# Diversity‐Oriented Catalytic Asymmetric Dearomatization of Indoles with *o*‐Quinone Diimides

**DOI:** 10.1002/advs.202305101

**Published:** 2023-10-23

**Authors:** Hao‐Jie Gao, Yu‐Hang Miao, Wen‐Na Sun, Rui Zhao, Xiao Xiao, Yuan‐Zhao Hua, Shi‐Kun Jia, Min‐Can Wang, Guang‐Jian Mei

**Affiliations:** ^1^ College of Chemistry Pingyuan Laboratory Zhengzhou University Zhengzhou 450001 China; ^2^ Collaborative Innovation Center of Yangtze River Delta Region Green Pharmaceuticals Zhejiang University of Technology Hangzhou 310014 China

**Keywords:** catalytic asymmetric dearomatization, chiral phosphoric acids, diversity‐oriented‐synthesis, indoles, *o*‐quinone diimides

## Abstract

Herein, the first diversity‐oriented catalytic asymmetric dearomatization of indoles with *o*‐quinone diimides (*o*‐QDIs) is reported. The catalytic asymmetric dearomatization (CADA) of indoles is one of the research focuses in terms of the structural and biological importance of dearomatized indole derivatives. Although great achievements have been made in target‐oriented CADA reactions, diversity‐oriented CADA reactions are regarded as more challenging and remain elusive due to the lack of synthons featuring multiple reaction sites and the difficulty in precise control of chemo‐, regio‐, and enantio‐selectivity. In this work, *o*‐QDIs are employed as a versatile building block, enabling the chemo‐divergent dearomative arylation and [4 + 2] cycloaddition reactions of indoles. Under the catalysis of chiral phosphoric acid and mild conditions, various indolenines, furoindolines/pyrroloindolines, and six‐membered‐ring fused indolines are collectively prepared in good yields with excellent enantioselectivities. This diversity‐oriented synthesis protocol enriches the *o*‐quinone chemistry and offers new opportunities for CADA reactions.

## Graphical abstract

The first diversity‐oriented catalytic asymmetric dearomatization of indoles is accomplished. *o*‐Quinone diimides are employed as a versatile building block, enabling the chemo‐divergent dearomative arylation and [4 + 2] cycloaddition reactions of indoles. Under the catalysis of chiral phosphoric acid and mild conditions, various indolenines, furoindolines/pyrroloindolines, and six‐membered‐ring fused indolines are collectively prepared in good yields with excellent enantioselectivities.

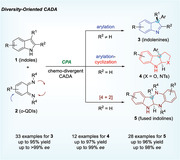

## 1 Introduction

**Figure 1:**
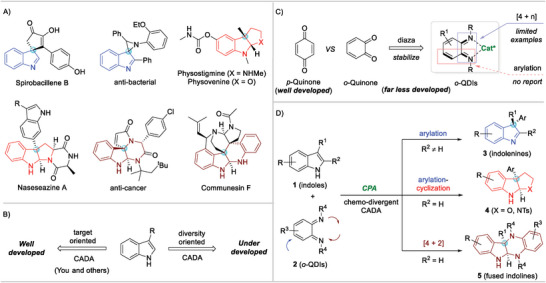
Diversity‐oriented CADA reaction of indoles with o‐QDIs. A) Representative molecules; B) CADA reactions; C) *o*‐QDIs; D) This work.

Dearomatized indole motifs bearing a C3‐quaternary stereocenter occur prevalently in natural alkaloids and pharmaceutical molecules. 3,3‐Disubstituted indolenines, pyrroloindolines/furoindolines, and six‐membered ring fused indolines are representative ones (**Figure** **1**A). Structurally, the C3‐quaternary stereocenters render rigid architectures, thus posing a daunting synthetic challenge. Biologically, they display a wide range of anti‐cancer, anti‐bacterial, and anti‐fungal properties.^[^ ^1^ ^]^ The combination of structural complexity and biological importance has inspired continuous interest from the chemical community in developing catalytic asymmetric approaches for these unique skeletons.^[^ ^2^ ^]^ Among many strategies, the catalytic asymmetric dearomatization (CADA) reaction has emerged as the most powerful one preparing enantio‐enriched 3D molecules directly from planar indoles (Figure 1B).^[^ ^3^ ^]^ To date, significant progresses have been made by You and others, which include the well‐investigated tandem C3‐functionalization/cyclization^[^ ^4^ ^]^ and [3 + 2] cycloaddition reactions^[^ ^5^ ^]^, and the less studied arylation^[^ ^6^ ^]^ and [4 + 2] cycloaddition reactions.^[^ ^7^ ^]^ While impressive, these methods are usually target‐oriented and provide the specified type of products. To our knowledge, the diversity‐oriented CADA reaction of indoles that allows facile access to structurally diversified scaffolds from the same starting materials, however, still remains elusive.^[^ ^8^ ^]^ The key to the success of such a protocol is the use of a synthon featuring multiple reaction sites and the precise control of chemo‐, regio‐, and enantio‐selectivity. Therefore, developing diversity‐oriented CADA reactions of indoles for rapid synthesis of indolenines and indolines is a highly desirable yet challenging subject.

Quinones, known for biological relevance, chemical versatility, and industrial importance, have attracted increasing attention in asymmetric synthesis.^[^ ^9^ ^]^ *p*‐Quinones and their imine derivatives are a typical class, which render substantial catalytic enantioselective arylations and annulations on the basis of 1,4‐conjugate addition reaction on quinone sp^2^ hybridized carbon.^[^ ^10^, ^11^ ^]^ Their ‘ortho’‐analogs *o*‐quinones, in stark contrast, have been dismissed for a long time due to the structural characteristics of high electrophilicity and easy dimerization/aromatization.^[^ ^12^, ^13^ ^]^ In fact, *o*‐quinones with multiple reaction sites show the potential to become a kind of versatile synthons. For example, the N‐protected *o*‐quinone diimide (*o*‐QDI) is a structurally stable variant and possesses imine, diene, *α,β*‐unsaturated system, and 1,4‐diazadiene etc. structural motifs.^[^ ^14^ ^]^ In this regard, *o*‐QDIs could serve as either arylation reagents via 1,4‐conjugate addition or heterodienes via [4 + n] cycloaddition (Figure 1C). However, catalytic asymmetric reactions involving *o*‐QDIs have not yet been developed with only limited examples which are focused on the synthesis of dihydroquinoxalines.^[^ ^15^ ^]^ This is the case that Lectka disclosed the asymmetric inverse electron demand hetero‐Diels–Alder reaction of *o*‐QDIs with ketene enolates through a cooperative catalytic strategy.^[^ ^15c^ ^]^ Furthermore, the enantioselective arylation reaction of QDIs has never been reported. Our team has a long‐term program to develop new reaction modes of traditional aza‐dienes.^[^ ^16^ ^]^ Herein, we envision that chemo‐divergent dearomative arylation and [4 + 2] cycloaddition reactions of indoles **1** with *o*‐QDIs **2** are feasible under the catalysis of chiral phosphoric acid (CPA) (Figure 1D). Namely, the dearomative C3‐arylation of indoles **1** via the 1,4‐conjugate addition of *o*‐QDIs **2** on quinone sp^2^ hybridized carbon allows the enantioselective synthesis of indolenines **3**; the dearomative arylation‐cyclization cascade of tryptophols/tryptamines leads to the formation of furoindolines/pyrroloindolines **4**; the chemo‐selective dearomative [4 + 2] cycloaddition of indoles with *o*‐QDIs on imine nitrogen delivers various six‐membered ring fused indolines **5**. This chemo‐divergent protocol not only provides a diversity‐oriented synthesis (DOS) of dearomatized indole derivatives but also enriches the quinone chemistry.

## 2 Results and Discussion

### 2.1 Reaction Optimization

We commenced our investigation with the model reaction between indole **1a** and *o*‐QDI **2a** under the catalysis of CPAs (**Table** **1**). To our delight, with **CPA‐1** in CH_2_Cl_2_, asymmetric dearomatization of indole **1a** with *o*‐QDI **2a** readily took place in 10 min, affording arylation product **3a** and [4 + 2] cycloaddition product **5a** with excellent enantioselectivities, respectively (entry 1). To investigate the chemo‐selectivity, the solvent effect was studied (entries 2−4). In all solvents with **CPA‐1**, arylation product **3a** dominated the dearomatization process. In CH_3_CN, the ratio of **3a** increased, but the enantioselectivity decreased (entry 3). Then, various commercially available CPAs were screened (entries 5−8). The results indicated that TRIP‐**CPA‐1** preferred the formation of **3a** (entry 1), while SiPh_3_‐derived **CPA‐5** furnished an equivalent amount of arylation product and [4 + 2] cycloaddition product (entry 8). Besides, in toluene with **CPA‐5**, the ratio of **3a:5a** could be reversed (entries 9−11). At 0 °C, [4 + 2] cycloaddition product **5a** was obtained as the major product (entry 10). Using 5 Å molecular sieve (MS) as an additive, the *ee* value was increased to 92% (entry 11).

**Table 1:** Reaction optimization.

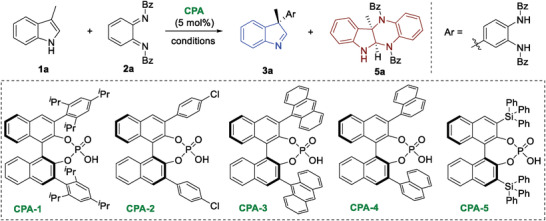							
Entry^a^ ^)^	CPA	solvent	3a:5a	3a		5a	
				Yield [%]^b^ ^)^	*ee* [%]^c^ ^)^	Yield [%]^b^ ^)^	*ee* [%])^c)^
1	**CPA‐1**	CH_2_Cl_2_	3.1:1	68	98	22	96
2	**CPA‐1**	toluene	4:1	72	96	18	94
3	**CPA‐1**	CH_3_CN	6.9:1	83	72	12	67
4	**CPA‐1**	THF	2.1:1	61	95	29	30
5	**CPA‐2**	CH_2_Cl_2_	1.9:1	66	43	35	62
6	**CPA‐3**	CH_2_Cl_2_	2.4:1	66	92	28	93
7	**CPA‐4**	CH_2_Cl_2_	1.8:1	59	73	32	84
8	**CPA‐5**	CH_2_Cl_2_	1:1	46	73	44	84
9	**CPA‐5**	toluene	1:1	46	82	48	88
10^d^ ^)^	**CPA‐5**	toluene	1:1.4	41	91	56	90
11^d)^, ^e)^ ^)^	**CPA‐5**	toluene	1:1.4	40	91	55	92

^a)^ Unless indicated otherwise, reaction conditions: **1a** (0.05 mmol), **2a** (0.05 mmol) added in five portions, **CPA** (5 mol%) in the specified solvent (1 mL) at room temperature (r.t.) for 10 min, the dr of **5a** was >20:1;

^b)^ Isolated yields;

^c)^ Determined by chiral HPLC analysis;

^d)^ At 0 °C.;

^e)^ With 5Å MS (50 mg).

**Figure 2:**
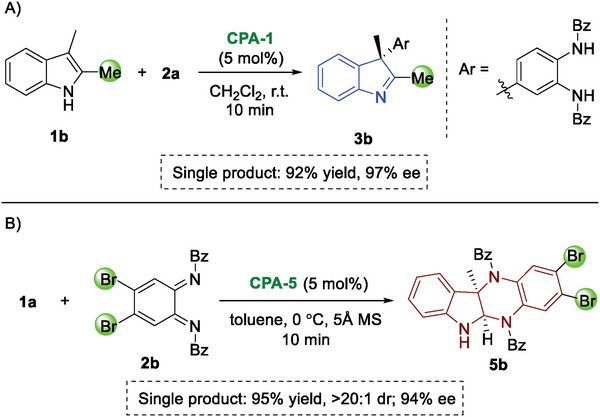
Further optimization by using disubstituted substrates. A) Dearomative arylation; B) Dearomative [4 + 2] cycloaddition.

Considering that substituents could affect selectivity, further optimization was carried out by employing disubstituted indole **1b** and *o*‐QDI **2b** (**Figure** **2**). Notably, under the conditions in entry 1 (Table 1), the reaction between 2,3‐dimethyl indole **1b** and *o*‐QDI **2a** occurred in a chemo‐specific manner, giving the only dearomative arylation product **3b** in 92% yield with 97% enantioselectivity (Figure 2A). In this regard, the steric hindrance had a significant influence on the dearomative [4 + 2] cycloaddition. On the other hand, by using dibromo‐substituted *o*‐QDI **2b**, the arylation reaction was avoided to give the reversed chemo‐selectivity (Figure 2B). In the presence of **CPA‐5** in toluene (Table 1, entry 11), only the dearomative [4 + 2] cycloaddition reaction of **1a** with **2b** took place, affording product **5b** in 95% yield with 94% *ee*. Consequently, chemo‐divergent CADA reactions of indoles with *o*‐QDIs were accomplished via the substrate‐control strategy.

### 2.2 Substrate Scope

**Figure 3:**
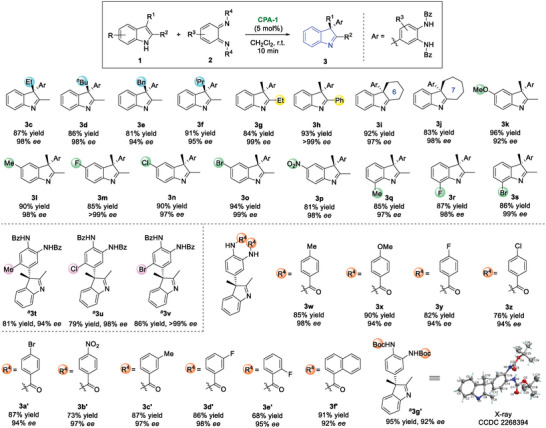
CADA arylation for the synthesis of indolenines. [a] Performed at 0 °C.

With the best conditions, we subsequently examined the substrate generality of CADA arylation of 2,3‐disubstituted indoles with *o*‐QDIs (**Figure** **3**). Considering that the variation of the C3‐quarternary carbon center is of great value in organic synthesis, the tolerance of different substituents at indole C3‐position was explored. We were pleased to find that substituents, such as ethyl (**3c**), *n*‐butyl (**3d**), benzyl (**3e**) as well as *i*‐propyl (**3f**) groups, were well compatible with the optimal conditions. It was also feasible to change the methyl group at indole C2‐position to ethyl (**3g**) and phenyl (**3h**) groups. Notably, fused indolenines (**3i−j**) were readily prepared with equally excellent yields and *ee* values. Various indole partners containing substituents on the phenyl ring were then evaluated. The consistently good results (**3k−s**) implied that neither the electronic nature nor position of these substituents affected the reaction efficiency and enantioselectivity. Furthermore, the reaction was applicable to a wide range of substituted *o*‐QDIs **2**. Although substituents on cyclohexadiene ring reduced the enantioselectivity, this could be simply addressed by lowering the reaction temperature (**3t−v**). In addition, the N‐protecting group of *o*‐QDIs could be altered, delivering a variety of synthetically interesting dearomative arylation products (**3w−f′**) in excellent yields and enantioselectivities. Of particular note is the use of *o*‐QDI with an easily removable Boc group. A low reaction temperature was employed to ensure a high *ee* value (**3g′**). The absolute configuration of compound **3g′** was unambiguously determined by X‐ray crystallography, and other arylation products were assigned by analogy.^[^ ^17^ ^]^

**Figure 4:**
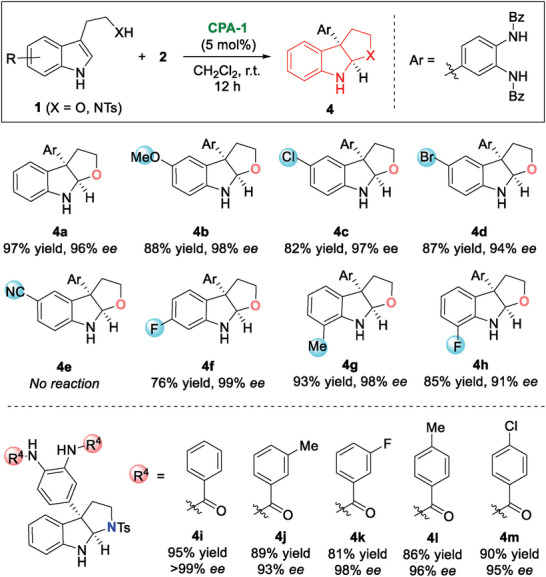
CADA arylation‐cyclization for the synthesis of furoindolines/pyrroloindolines.

Furoindolines/pyrroloindolines are important and privileged polycyclic indoline motifs, which can be derived from tryptophols/tryptamines via a dearomatization‐cyclization cascade.^[^ ^2a^ ^]^ In this context, we assumed the CADA arylation of tryptophols/tryptamines with *o*‐QDIs followed by intramolecular cyclization was feasible (**Figure** **4**). To demonstrate this concept, tryptophol was directly subjected to the reaction with *o*‐QDI **2a** under standard conditions. Pleasingly, furoindoline **4a** was created with 97% yield and 96% *ee*. Then, various tryptophols bearing substituents on the phenyl ring were employed to explore the electronic effect (**4b−h**). The electron‐donating and halogen groups afforded the corresponding products in good yields with excellent *ee* values, while the strong electron‐withdrawing ‐CN group gave no reaction result (**4e**). Additionally, the reaction cascade could be extended to tryptamine. A series of structurally important pyrroloindolines (**4i−m**) were prepared via the reaction of NTs tryptamine with *o*‐QDIs **2**. The observed excellent chemo‐selectivity could be attributed to the ‐XH group, which acted as a directing group via hydrogen‐bonding interaction.

**Figure 5:**
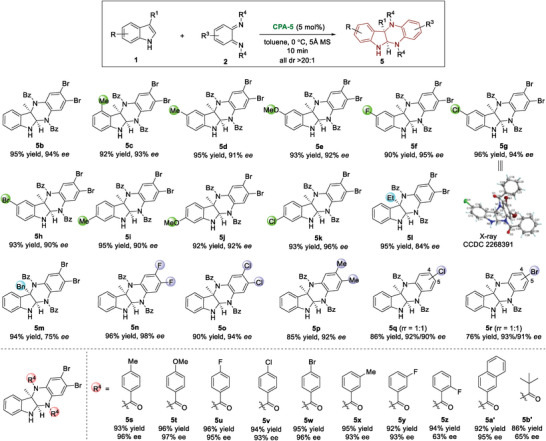
CADA [4 + 2] cycloaddition for synthesis of six‐membered ring fused indolines.

Next, we turned our attention to the substrate scope of dearomative [4 + 2] cycloaddition reaction (**Figure** **5**). The tolerance of the substituents at the indole ring was tested by reacting with *o*‐QDI **2b** under standard conditions. All patterns of substituents on the phenyl ring were well tolerated, and cycloaddition products (**5c−k**) were obtained in excellent yields with excellent enantioselectivities. However, the substituents at the C3‐position had some influence on the enantio‐control. As the steric hindrance increased, the *ee* value significantly decreased (**5l−m**). This result once again indicated that the dearomative [4 + 2] cycloaddition reaction was sensitive to the steric effect. Besides, the optimized conditions were well compatible to other disubstituted *o*‐QDIs (**5n−p**). When monosubstituted *o*‐QDIs were employed, regioisomers (**5q−r**) appeared. Finally, the N‐protecting groups of *o*‐QDIs were varied (**5s−b′**), whose steric hindrance deteriorated the enantioselectivity as shown in the results of **5z** and **5b′**. The absolute configuration of compound **5g** was unambiguously determined by X‐ray crystallography, and other [4 + 2] cycloaddition products were assigned by analogy.^[^ ^17^ ^]^

**Figure 6:**
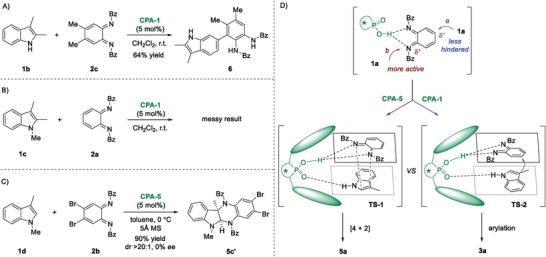
Control experiments and mechanism considerations. A) Reaction of 2,3‐dimethyl indole **1b** with disubstituted *o*‐QDI; B) The use of *N*‐methylated indoles **1c** for arylation; C) The use of *N*‐methylated indoles **1d** for [4 + 2] cycloaddition; D) Mechanism considerations.

To explore the mechanism, control experiments were conducted (**Figure** **6**). Under standard conditions, the reaction of 2,3‐dimethyl indole **1b** with disubstituted *o*‐QDI **2c** delivered indole C6‐arylation product **6** rather than the dearomative C3‐arylation or [4 + 2] cycloaddition product (Figure 6A).^[^ ^18^ ^]^ This chemical event should involve an interesting 1,6‐addition process, which will be investigated in detail in the future. The use of *N*‐methylated indoles **1c** and **1d** as substrates gave rise to messy results and racemic [4 + 2] product **5c′** respectively (Figure 6B,C). These outcomes underlined the importance of indole NH for the reactivity and stereoselectivity. On the basis of these experimental results, plausible transition states were proposed for this divergent dearomatization reaction (Figure 6D). First of all, the CPA catalyst simultaneously activated indoles and *o*‐QDIs via H‐bonding interaction. Similar to quinone methides,^[^ ^19^ ^]^ the driving force of aromatization and the profound electrophilicity of imine nitrogen make *o*‐QDIs as good 1,4‐diazadienes (N═C─C═N). Therefore, dearomative [4 + 2] cycloaddition occurred via transition state **TS‐1**, facilitating the formation of a six‐membered ring fused indoline **5a**. Nevertheless, electronic effects can be overwhelmed by steric interactions. In this event, 1,4‐addition reaction of *o*‐QDI took place via **TS‐2**, leading to the dearomative arylation product indolenine **3a**. Dual H‐bonding interaction, plausible π–π interaction, as well as steric hindrance might contribute to the observed excellent enantio‐control.

**Figure 7:**
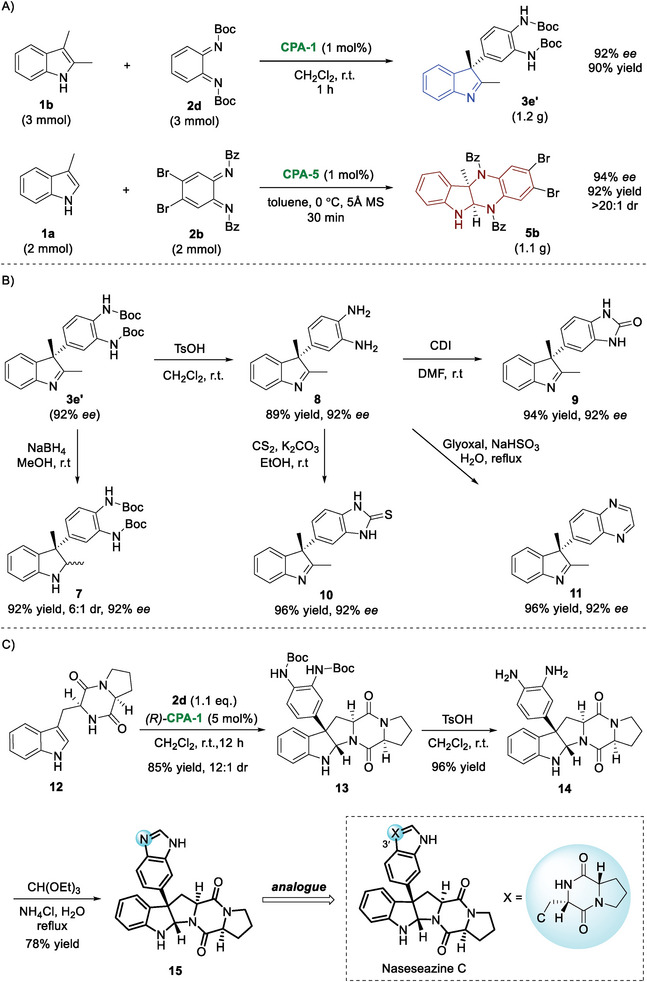
Further elaborations. A) Large‐scale synthesis; B) Further transformations; C) Facile synthesis of (+)‐Naseseazines C analog.

Finally, to demonstrate the synthetic utility of this diversity‐oriented dearomatization protocol, large‐scale preparations of indolenine **3e′** and six‐membered ring fused indoline **5b** were carried out. As shown in **Figure** **7**A, in the presence of 1 mol% CPA, the reactions readily occurred without erosion of enantioselectivity. Furthermore, the prepared indolenine **3e′** can be transformed into some interesting indole derivatives (Figure 7B). Reduction of the imine motif with NaBH_4_ afforded indoline **7**. TsOH‐triggered Boc deprotection furnished compound **8** in a good yield. The vicinal NH_2_ groups can undergo further cyclization reactions with CDI, CS_2_, and glyoxal to form other useful indolenines **9−11** bearing a heteroaromatic group at the C3‐position. Additionally, the facile synthesis of (+)‐Naseseazines C analog was accomplished (Figure 7C). The synthetic route commenced with the CPA‐catalyzed diastereoselective dearomatization of cyclo(L‐Pro‐L‐Trp‐) **12** with *o*‐QDI **2d**. In the presence of (*R*)‐**CPA‐1**, the pentacyclic product **13** was obtained in 85% yield with 12:1 dr. The following deprotection and cyclization delivered the corresponding compound **15**, a 3′‐aza analog of (+)‐Naseseazines C.^[^ ^20^ ^]^

## 3 Conclusion

In conclusion, we have established the first diversity‐oriented CADA of indoles with *o*‐QDIs. Given the importance of dearomatized indole derivatives, CADA reaction of indoles is a hot topic in organic synthesis. As opposite to the well‐developed target‐oriented CADA reactions, diversity‐oriented CADA reactions are more challenging and remain elusive due to the lack of synthons featuring multiple reaction sites and the difficulty in precise control of chemo‐, regio‐, and enantio‐selectivity. In this work, *o*‐QDIs were employed as a versatile building block, enabling the chemo‐divergent dearomative arylation and [4 + 2] cycloaddition reactions of indoles. Under the catalysis of CPA, various indolenines, furoindolines/pyrroloindolines, and six‐membered ring fused indolines were collectively prepared in a DOS manner. This chemo‐divergent protocol not only enriches the CADA reaction of indoles but also offers new reaction modes for *o*‐QDIs. Further investigations along this line are ongoing in our laboratory and will be reported in due course.

## 4 Experimental Section

### Typical Procedure for Arylation Reaction

To a solution of 2,3‐disubstituted indoles **1** (0.1 mmol, 1 equiv.) and catalyst **CPA‐1** (5 mol%) in anhydrous CH_2_Cl_2_ (1 mL), was added *o‐*QDIs **2** (1 equiv.) in five portions. The reaction mixture was stirred for 10 min at room temperature. After completion (monitored by TLC), the solvent was removed under reduced pressure and the crude product was directly purified by flash chromatography on silica gel employing mixtures of petroleum and ethyl acetate (petroleum ether/ethyl acetate 4:1–2:1) as eluents to afford the desired product **3**.

### Typical Procedure for [4 + 2] Reaction

To a solution of 3‐substituted indoles **1** (0.1 mmol, 1 equiv.) and catalyst **CPA‐5** (5 mol%) in anhydrous toluene (1 mL), was added 50 mg 5Å MS and *o*‐QDIs **2** (1 equiv.) in five portions. The reaction mixture was stirred for 10 min at 0 °C. After completion (monitored by TLC), the solvent was removed under reduced pressure, and the crude product was directly purified by flash chromatography on silica gel employing mixtures of petroleum and ethyl acetate (petroleum ether/ethyl acetate 4:1) as eluents to afford the desired product **5**.

[CCDC 2268394 for **3g′** and 2268391 for **5g** contain the supplementary crystallographic data for this paper. These data can be obtained free of charge from The Cambridge Crystallographic Data Centre via www.ccdc.cam.ac.uk/data_request/cif.]

## Conflict of Interest

The authors declare no conflict of interest.

## Supporting information

Supporting Information

## Data Availability

The data that support the findings of this study are available in the supplementary material of this article.
