# Supplementary material for: Diversity‐Oriented Catalytic Asymmetric Dearomatization of Indoles with o‐Quinone Diimides
Source: Adv Sci (Weinh). 2023 Oct 23;10(35):2305101. doi: 10.1002/advs.202305101 (PMC10724437; doi:10.1002/advs.202305101)
Supplement: Supplementary file 1 — Supporting Information [file ADVS-10-2305101-s001.pdf]

ADVANCED SCIENCE
Open Access

Supporting Information

for *Adv. Sci.*, DOI 10.1002/advs.202305101

Diversity-Oriented Catalytic Asymmetric Dearomatization of Indoles with *o*-Quinone Diimides

*Hao-Jie Gao, Yu-Hang Miao, Wen-Na Sun, Rui Zhao, Xiao Xiao, Yuan-Zhao Hua, Shi-Kun Jia, Min-Can Wang and Guang-Jian Mei**

Supporting Information

Diversity-Oriented Catalytic Asymmetric Dearomatization of Indoles with *o*-Quinone Diimides

Hao-Jie Gao, Yu-Hang Miao, Wen-Na Sun, Rui Zhao, Xiao Xiao, Yuan-Zhao Hua, Shi-Kun Jia, Min-Can Wang, and Guang-Jian Mei*

Table of Contents

1. General Information S2

2. Representative Procedures S3

3. Characterization of Products S8

4. Crystallographic data collections for compounds **3g'** & **5g** S42

5. NMR Spectra of compounds S46

6. HPLC spectra of compounds S146

7. References S226

1. General Information

Unless otherwise specified, all reactions were conducted under an inert atmosphere and anhydrous conditions. All the solvents were purified according to the standard procedures. All chemicals which are commercially available were employed without further purification. Thin-layer chromatography (TLC) was performed on silica gel plates using UV-light (254 and 365 nm). Flash chromatography was conducted on silica gel (200–300 mesh). NMR spectra were recorded on a 400 MHz NMR spectrometer with $CDCl_3$ or d_6-DMSO as the solvent and TMS as an internal standard (400 MHz for ^{1}H and 100 MHz for ^{13}C). All high-resolution mass spectra were obtained on a Q-TOF Micro LC/MS System ESI spectrometer to be given in m/z. Enantiomeric excesses values were determined with HPLC (chiral column; mobile phase hexane/i-PrOH). Optical rotations were measured using an Anton Paar MCP-4100 digital polarimeter. Indoles **1** were either employed directly from commercial sources or prepared according to the literature[1], *o*-QDIs **2** were synthesized according to modified literature-reported procedures[2].

2. Representative Procedures

2.1 Optimization of the reaction conditions

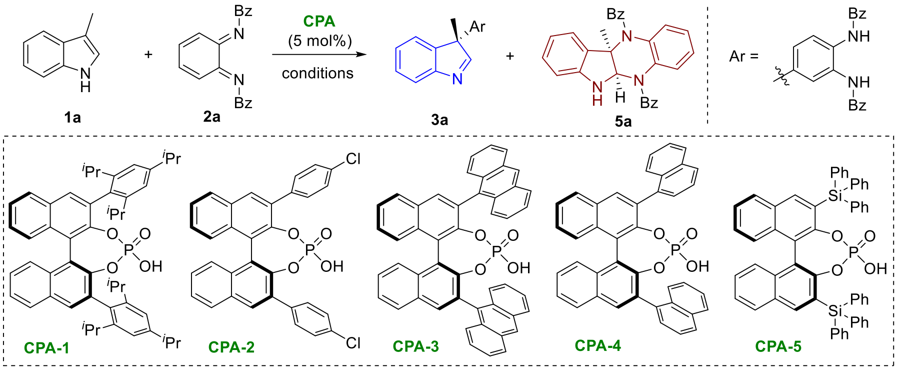

Entry[a]	CPA	solvent	3a:5a	3a		5a	
				Yiled (%)[b]	*ee* (%)[c]	Yiled (%)[b]	*ee* (%)[c]
1	**CPA-1**	CH_2Cl_2	3.1:1	68	98	22	96
2	**CPA-1**	toluene	4:1	72	96	18	94
3	**CPA-1**	CH_3CN	6.9:1	83	72	12	67
4	**CPA-1**	THF	2.1:1	61	95	29	30
5	**CPA-2**	CH_2Cl_2	1.9:1	66	43	35	62
6	**CPA-3**	CH_2Cl_2	2.4:1	66	92	28	93
7	**CPA-4**	CH_2Cl_2	1.8:1	59	73	32	84
8	**CPA-5**	CH_2Cl_2	1:1	46	73	44	84
9	**CPA-5**	toluene	1:1	46	82	48	88
10[d]	**CPA-5**	toluene	1:1.4	41	91	56	90
11[d,e]	**CPA-5**	toluene	1:1.4	40	91	55	92

[a] Unless indicated otherwise, reaction conditions: **1a** (0.05 mmol), **2a** (0.05 mmol) added in 5 portions, **CPA** (5 mol%) in the specified solvent (1 mL) at room temperature (r.t.) for 10 min, the dr of **5a** was >20:1. [b] Isolated yields. [c] Determined by chiral HPLC analysis. [d] At 0 °C. [e] With 5Å MS (50 mg).

2.2 General Procedures for the Synthesis of 3

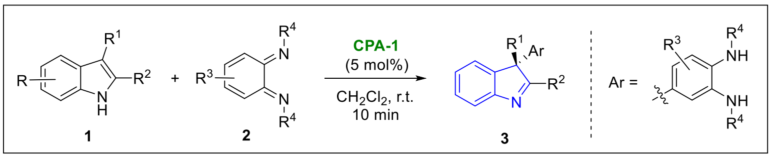

To a solution of indoles **1** (0.1 mmol, 1 equiv) and catalyst **CPA-1** (5 mol%) in anhydrous DCM (1 mL), was added *o*-QDIs **2** (1 equiv) in 5 portions. The reaction mixture was stirred for 10 min at rt. After completion (monitored by TLC), the solvent was removed under reduced pressure and the

crude product was directly purified by flash chromatography on silica gel employing mixtures of petroleum and ethyl acetate (petroleum ether/ethyl acetate 4:1–2:1) as eluents to afford the desired product **3**.

2.3 General Procedures for the Synthesis of 4

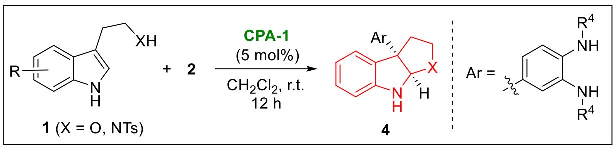

To a solution of indoles **1** (0.1 mmol, 1 equiv) and catalyst **CPA-1** (5 mol%) in anhydrous DCM (1 mL), was added *o*-QDIs **2** (1 equiv) in one portion. The reaction mixture was stirred for 12 h at rt. After completion (monitored by TLC), the solvent was removed under reduced pressure and the crude product was directly purified by flash chromatography on silica gel employing mixtures of petroleum and ethyl acetate (petroleum ether/ethyl acetate 4:1–2:1) as eluents to afford the desired product **4**.

2.4 General Procedures for the Synthesis of 5

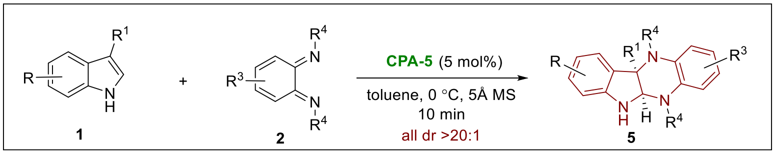

To a solution of indoles **1** (0.1 mmol, 1 equiv) and catalyst **CPA-5** (5 mol%) in anhydrous toluene (1 mL), was added 50 mg 5Å MS and *o*-QDIs **2** (1 equiv) in 5 portions. The reaction mixture was stirred for 10 min at 0 °C After completion (monitored by TLC), the solvent was removed under reduced pressure and the crude product was directly purified by flash chromatography on silica gel employing mixtures of petroleum and ethyl acetate (petroleum ether/ethyl acetate 4:1) as eluents to afford the desired product **5**.

2.5 Detailed Procedures for further transformation of 3g'and facile synthesis of (+)-Naseseazines C analogue 15:

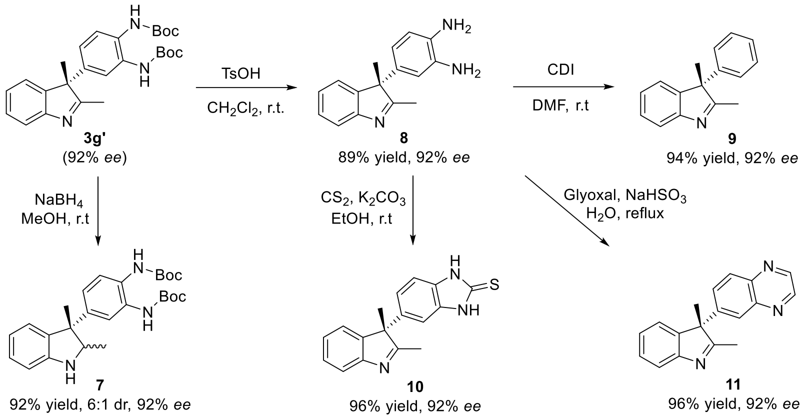

For compound **7:** To a solution of the **3g'** (0.1 mmol) in MeOH (2 mL) was added $NaBH_4$ (0.4 mmol). Then the mixture was stirred at rt overnight. After completion, the reaction mixture was quenched with saturated $NaHCO_3$ solution and extracted with DCM. The organic layer was washed with brine and dried over anhydrous Na_2SO_4. After removal of solvent, product **7** was obtained through purification by flash column chromatography over a short plug of silica gel using AcOEt and petroleum ether as eluent.

For compound **8**: TsOH was added to the solution of **3g'** (45.1 mg; 0.10 mmol) in dichloromethane (2 mL), which was stirred at rt. After the completion of the reaction indicated by TLC, saturated $NaHCO_3$ aqueous solution was added to the reaction mixture to adjust the pH, which was then extracted by DCM. The resultant organic layer was dried by anhydrous Na_2SO_4 and purified by flash column chromatography to afford product **8**.

For compound **9:** To a flask containing a solution of **8** (0.1 mmol) in DMF (2 mL) were added 1,1'-carbonyldiimidazole (0.1 mmol). Then the mixture was stirred at room temperature until TLC showed complete consumption of starting material. Water was added and the mixture was extracted with AcOEt. The combined organic layer was washed with brine, dried over Na_2SO_4 and filtered. After the solvent was removed under reduced pressure, the residue was purified by silica gel column chromatography to afford **9.**

For compound **10:** A suspension of **8** (0.1 mmol) and potassium carbonate (0.15 mmol) in ethanol (2 mL) was stirred at room temperature, and then carbon disulfide (0.2 mmol) added dropwise. The reaction mixture was heated under reflux. After completion of the reaction (TLC monitoring), the mixture was cooled to room temperature and diluted with water (10 mL). Then the mixture was extracted with AcOEt and the organic layers were combined and dried (Na_2SO_4). After concentration under reduced pressure, the pure product was obtained by flash column chromatography.

For compound **11:** A solution of 40% aqueous glyoxal and sodium hydrogen sulfite in water (16 mL) was heated to 70 ^{o}C. The suspense on of **8** was added to the reaction mixture. Then the reaction mixture was allowed to reach room temperature. After completion of the reaction (TLC monitoring), the reaction mixture was basified to pH 7.5 with solid sodium carbonate at room temperature; which

was then extracted by AcOEt. The resultant organic layer was dried by anhydrous Na_2SO_4 and purified by flash column chromatography to afford product **11**.

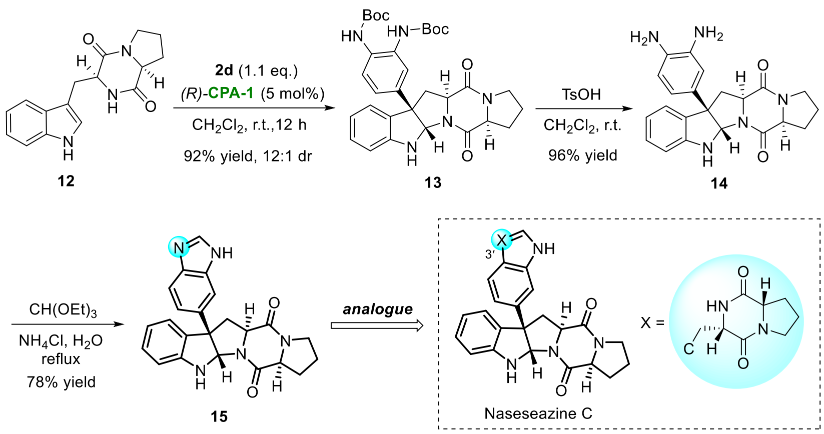

For compound **13:** To a solution of indoles **12** (0.3 mmol, 1 equiv) and catalyst (*R*)-**CPA-1** (5 mol%) in anhydrous DCM (3 mL), was added *o*-QDI **2d** (1.1 equiv) in one portion. The reaction mixture was stirred for 12 h at rt. After completion (monitored by TLC), the solvent was removed under reduced pressure and the crude product was directly purified by flash chromatography on silica gel employing mixtures of petroleum and ethyl acetate (CH_2Cl_2/ethyl acetate 3:1–2:1) as eluents to afford the desired product **13**.

For compound **14**: TsOH (0.11 mmol, 1.1 equiv) was added to the solution of **13** (0.10 mmol) in dichloromethane (2 mL), which was stirred at rt. After the completion of the reaction indicated by TLC, saturated $NaHCO_3$ aqueous solution was added to the reaction mixture to adjust the pH, which was then extracted by DCM. The resultant organic layer was dried by anhydrous Na_2SO_4 and purified by flash column chromatography to afford product **14**.

For compound **15:** Charge a 10 mL one-necked round-bottomed flask, equipped with a magnetic stirrer and a condenser with **14** (0.1 mmol) , (0.015 mmol, 15 mol%) of NH_4Cl, (18 μL, 15.6 mg) of triethyl orthoformate and 2 mL of H_2O. The reaction mixture was refluxed (oil bath) for 30 min. At this time, TLC indicated the reaction was complete. The reaction mixture was cooled to 23°C and some of the product crystallized. The mixture was extracted with ethyl acetate (3 × 5 mL). The resultant organic layer was dried by anhydrous Na_2SO_4 and purified by flash column chromatography to afford product **15**.

2.6 Large-scale synthesis

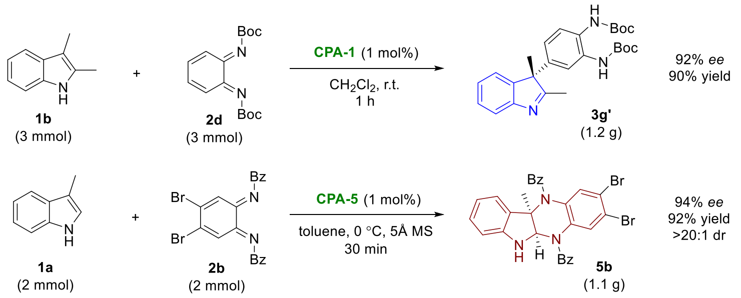

For compound **3g':** To a stirring anhydrous DCM solution (15 mL) of indoles **1b** (3 mmol) and **2d** (3 mmol) was added **CPA-1** (1 mol%) at rt. The reaction mixture was stirred until completion of reaction (1 h, as monitored by TLC). Water was added and the mixture was extracted with AcOEt (3 × 20 mL). The combined organic layer was washed with brine, separated, dried over Na_2SO_4 and filtered. The solvent was then removed under reduced pressure and the residue was purified by flash column chromatography on silica gel (PE:EtOAc = 2:1) to afford product **3g'** (1.2 g) in 90% yield with 92% ee. Moreover, catalyst **CPA-1** was recycled (purified by column chromatography, followed by acidification with 1N HCl), which was demonstrated to possess same catalytic activity.

For compound **5b:** To a stirring anhydrous toluene solution (15 mL) of indoles **1a** (2 mmol) and **2b** (2 mmol) was added **CPA-5** (1 mol%) and 2 g 5Å MS at 0 ºC. The reaction mixture was stirred until completion of reaction (30 min, as monitored by TLC). Water was added and the mixture was extracted with AcOEt (3 × 20 mL). The combined organic layer was washed with brine, separated, dried over Na_2SO_4 and filtered. The solvent was then removed under reduced pressure and the residue was purified by flash column chromatography on silica gel (PE:EtOAc = 4:1) to afford product **5b** (1.1 g) in 92% yield with 94% ee. Moreover, catalyst **CPA-5** was recycled (purified by column chromatography, followed by acidification with 1N HCl), which was demonstrated to possess same catalytic activity.

3. Characterization of Products

(*S*)-*N,N'*-(4-(3-methyl-3*H*-indol-3-yl)-1,2-phenylene)dibenzamide **3a:**

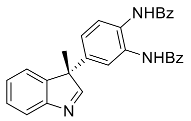

3a

Yellow oil; 30.6 mg; isolated yield = 68%; $[\alpha]_D^{20}$ = -132 (*c* 0.48, MeOH); HPLC (Chiralpak OD, *i*-propanol/hexane = 20/80, flow rate 1.0 mL/min, λ = 254 nm), t_1 = 11.12 min (major), t_2 = 13.74 min (minor), *ee* = 98%; ^{1}H NMR (400 MHz, $CDCl_3$) δ ppm: 9.75 (s, 1H), 9.52 (s, 1H), 8.15 – 8.03 (m, 2H), 8.01 – 7.95 (m, 2H), 7.59 – 7.43 (m, 7H), 7.42 (s, 1H), 7.32 – 7.24 (m, 3H), 7.13 – 7.05 (m, 1H), 6.82 (d, *J* = 7.3 Hz, 1H), 6.41 – 6.33 (m, 1H), 1.33 (s, 3H); ^{13}C NMR (100 MHz, $CDCl_3$) δ ppm: 177.6, 166.7, 166.4, 154.2, 144.5, 136.0, 133.3, 133.2, 132.5, 132.4, 131.0, 130.2, 128.9, 128.8, 128.0, 127.9, 127.6, 126.8, 126.4, 124.4, 123.1, 122.4, 121.3, 60.4, 19.4; HRMS (ESI) Calcd. For $C_{29}H_{24}N_3O_2^+$ $[M+H]^+$ 446.1863, found 446.1870.

(*S*)-*N,N'*-(4-(2,3-dimethyl-3*H*-indol-3-yl)-1,2-phenylene)dibenzamide **3b:**

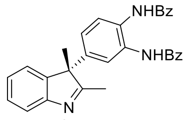

3b

White solid; 42.2 mg; isolated yield = 92%; M.p.: 152 – 153 °C; $[\alpha]_D^{20}$ = -129 (*c* 0.15, MeOH); HPLC (Chiralpak IC, *i*-propanol/hexane = 30/70, flow rate 1.0 mL/min, λ = 254 nm), t_1 = 18.87 min (major), t_2 = 26.98 min (minor), *ee* = 97%; ^{1}H NMR (400 MHz, d_6-DMSO) δ ppm: 10.13 (s, 1H), 9.99 (s, 1H), 7.97 – 7.87 (m, 4H), 7.65 (d, *J* = 8.5 Hz, 1H), 7.59 – 7.47 (m, 7H), 7.37 – 7.30 (m, 2H), 7.26 – 7.17 (m, 2H), 7.04 – 7.00 (m, 1H), 2.15 (s, 3H), 1.68 (s, 3H); ^{13}C NMR (100 MHz, d_6-DMSO) δ ppm: 186.7, 166.1, 165.8, 154.7, 147.2, 137.3, 134.6, 134.4, 132.4, 131.9, 130.8, 129.1, 129.0, 128.3, 128.1, 127.9, 126.7, 126.0, 123.7, 123.6, 123.0, 120.2, 61.5, 20.6, 16.1; HRMS (ESI) Calcd. For $C_{30}H_{25}N_3O_2Na^+$ $[M+Na]^+$ 482.1839, found 482.1845.

(*S*)-*N,N'*-(4-(3-ethyl-2-methyl-3*H*-indol-3-yl)-1,2-phenylene)dibenzamide **3c:**

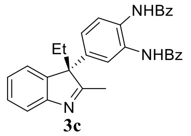

3c

White solid; 41.2 mg; isolated yield = 87%; M.p.: 174 – 175 °C; $[\alpha]_D^{20}$ = -19 (*c* 0.12, MeOH); HPLC (Chiralpak IC, *i*-propanol/hexane = 30/70, flow rate 1.0 mL/min, λ = 254 nm), t_1 = 16.26 min (major), t_2 = 25.03 min (minor), *ee* = 98%; ^{1}H NMR (400 MHz, d_6-DMSO) δ ppm: 10.14 (s, 1H), 10.01 (s, 1H), 7.95 – 7.89 (m, 4H), 7.65 (d, *J* = 8.5 Hz, 1H), 7.59 – 7.47 (m, 7H), 7.38 – 7.32 (m, 2H), 7.24 – 7.18 (m, 2H), 7.03 (dd, *J* = 8.5, 2.1 Hz, 1H), 2.50 – 2.41 (m, 1H), 2.33 – 2.22 (m, 1H), 2.12 (s, 3H), 0.41 (t, *J* = 7.2

Hz, 3H); ^{13}C NMR (100 MHz, d_6-DMSO) δ ppm: 185.3, 166.1, 165.8, 155.8, 144.6, 137.3, 134.6, 134.4, 132.4, 131.9, 130.7, 129.1, 129.0, 128.4, 128.1, 127.9, 126.7, 126.0, 123.9, 123.3, 120.0, 66.5, 26.3, 16.4, 8.4; HRMS (ESI) Calcd. For $C_{31}H_{28}N_3O_2^+$ $[M+H]^+$ 474.2176, found 474.2189.

(*S*)-*N,N'*-(4-(3-butyl-2-methyl-3*H*-indol-3-yl)-1,2-phenylene)dibenzamide **3d:**

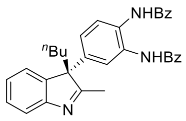

3d

Yellow oil;, 43.1 mg; isolated yield = 86%; $[\alpha]_D^{20}$ = -68 (*c* 0.25, MeOH); HPLC (Chiralpak IC, *i*-propanol/hexane = 30/70, flow rate 1.0 mL/min, λ = 254 nm), t_1 = 12.73 min (major), t_2 = 16.24 min (minor), *ee* = 98%; ^{1}H NMR (400 MHz, d_6-DMSO) δ ppm: 10.12 (s, 1H), 9.98 (s, 1H), 8.00 – 7.85 (m, 4H), 7.65 – 7.55 (m, 3H), 7.54 – 7.46 (m, 5H), 7.38 – 7.31 (m, 2H), 7.23 – 7.16 (m, 2H), 7.03 – 6.95 (m, 1H), 2.47 – 2.38 (m, 1H), 2.32 – 2.19 (m, 1H), 2.12 (s, 3H), 1.29 – 1.16 (m, 4H), 0.77 (t, *J* = 7.3 Hz, 3H); ^{13}C NMR (100 MHz, d_6-DMSO) δ ppm: 185.6, 166.1, 165.7, 155.6, 145.0, 137.4, 134.6, 134.4, 132.4, 131.8, 130.7, 129.1, 129.0, 128.3, 128.1, 127.9, 126.7, 126.0, 123.9, 123.2, 120.1, 65.9, 33.1, 25.9, 22.7, 16.4, 14.2; HRMS (ESI) Calcd. For $C_{33}H_{32}N_3O_2^+$ $[M+H]^+$ 502.2489, found 502.2497.

(*S*)-*N,N'*-(4-(3-benzyl-2-methyl-3*H*-indol-3-yl)-1,2-phenylene)dibenzamide **3e:**

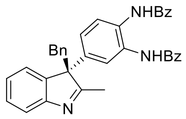

3e

White solid; 43.3 mg; isolated yield = 81%; M.p.: 109 - 110 °C; $[\alpha]_D^{20}$ = -123 (*c* 0.67, MeOH); HPLC (Chiralpak IC, *i*-propanol/hexane = 30/70, flow rate 1.0 mL/min, λ = 254 nm), t_1 = 17.07 min (major), t_2 = 49.02 min (minor), *ee* = 94%;^{1}H NMR (400 MHz, $CDCl_3$) δ ppm: 9.83 (s, 1H), 9.65 (s, 1H), 8.12 (d, *J* = 7.4 Hz, 2H), 7.98 (d, *J* = 7.4 Hz, 2H), 7.53 – 7.31 (m, 8H), 7.13 (dd, *J* = 7.2, 5.8 Hz, 2H), 7.03 – 6.96 (m, 2H), 6.94 – 6.87 (m, 2H), 6.70 (d, *J* = 7.3 Hz, 1H), 6.43 (d, *J* = 7.3 Hz, 2H), 6.36 (dd, *J* = 8.5, 1.8 Hz, 1H), 3.50 – 3.18 (m, 2H), 1.84 (s, 3H); ^{13}C NMR (100 MHz, $CDCl_3$) δ ppm: 184.4, 166.6, 166.4, 154.9, 143.7, 137.2, 134.6, 133.2, 133.2, 132.5, 132.4, 131.2, 130.3, 129.1, 128.9, 128.8, 128.0, 127.9, 127.6, 127.5, 126.7, 126.5, 125.4, 124.9, 123.2, 119.6, 66.8, 39.1, 16.7; HRMS (ESI) Calcd. For $C_{36}H_{30}N_3O_2^+$ $[M+H]^+$ 536.2333, found 536.2330.

(*S*)-*N,N'*-(4-(3-isopropyl-2-methyl-3*H*-indol-3-yl)-1,2-phenylene)dibenzamide **3f:**

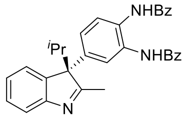

3f

White solid; 44.3 mg; isolated yield = 91%; M.p.: 138 – 139 °C; $[\alpha]_D^{20}$ = -139 (*c* 0.58, MeOH); HPLC (Chiralpak IC, *i*-propanol/hexane = 30/70, flow rate 1.0 mL/min, λ =

254 nm), t_1 = 9.95 min (major), t_2 = 16.22 min (minor), *ee* = 95%; ^{1}H NMR (400 MHz, d_6-DMSO) δ ppm: 10.09 (s, 1H), 10.00 (s, 1H), 7.95 – 7.86 (m, 4H), 7.65 – 7.48 (m, 8H), 7.44 – 7.35 (m, 3H), 7.31 – 7.23 (m, 1H), 7.05 – 6.98 (m, 1H), 3.06 – 2.89 (m, 1H), 2.14 (s, 3H), 1.09 (d, *J* = 6.6 Hz, 3H), 0.42 (d, *J* = 6.6 Hz, 3H); ^{13}C NMR (100 MHz, d_6-DMSO) δ ppm: 185.1, 166.1, 165.9, 156.5, 141.3, 136.5, 134.6, 134.4, 132.4 131.8, 130.8, 129.1, 129.0, 128.7, 128.0, 127.9, 126.5, 125.5, 125.2, 125.1, 124.8, 120.2, 70.7, 30.7, 18.2, 16.9, 16.7; HRMS (ESI) Calcd. For $C_{32}H_{30}N_3O_2^+$ [M+H]$^+$ 488.2333, found 488.2339.

(*S*)-*N,N'*-(4-(2-ethyl-3-methyl-3*H*-indol-3-yl)-1,2-phenylene)dibenzamide **3g:**

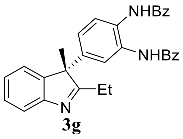

3g

White solid; 39.7 mg; isolated yield = 84%; M.p.: 170 – 171 °C; $[\alpha]_D^{20}$ = +17 (*c* 0.10, MeOH); HPLC (Chiralpak IC, *i*-propanol/hexane = 30/70, flow rate 1.0 mL/min, λ = 254 nm), t_1 = 12.42 min (major), t_2 = 17.46 min (minor), *ee* = 99%; ^{1}H NMR (400 MHz, d_6-DMSO) δ ppm: 10.13 (s, 1H), 10.00 (s, 1H), 7.96 – 7.88 (m, 4H), 7.63 (d, *J* = 8.5 Hz, 1H), 7.60 – 7.54 (m, 3H), 7.53 – 7.48 (m, 4H), 7.36 – 7.28 (m, 2H), 7.25 – 7.15 (m, 2H), 6.98 (dd, *J* = 8.5, 1.6 Hz, 1H), 2.63 – 2.53 (m, 1H), 2.38 – 2.26 (m, 1H), 1.67 (s, 3H), 1.21 (t, *J* = 7.3 Hz, 3H); ^{13}C NMR (100 MHz, d_6-DMSO) δ ppm: 190.7, 166.1, 165.8, 154.6, 147.3, 137.5, 134.6, 134.4, 132.4, 131.9, 130.7, 129.1, 129.0, 128.3, 128.1, 127.9, 126.7, 126.1, 123.7, 123.6, 122.9, 120.4, 61.5, 22.5, 20.7, 10.9; HRMS (ESI) Calcd. For $C_{31}H_{28}N_3O_2^+$ [M+H]$^+$ 474.2176, found 474.2182.

(*S*)-*N,N'*-(4-(3-methyl-2-phenyl-3*H*-indol-3-yl)-1,2-phenylene)dibenzamide **3h:**

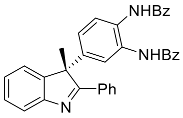

3h

White solid; 48.5 mg; isolated yield = 93%; M.p.: 90 – 91 °C; $[\alpha]_D^{20}$ = -43 (*c* 0.48, MeOH); HPLC (Chiralpak IC, *i*-propanol/hexane = 20/80, flow rate 1.0 mL/min, λ = 254 nm), t_1 = 16.27 min (major), t_2 = 44.49 min (minor), *ee* = >99%; ^{1}H NMR (400 MHz, d_6-DMSO) δ ppm: 10.16 (s, 1H), 9.99 (s, 1H), 7.98 – 7.85 (m, 6H), 7.74 (d, *J* = 7.7 Hz, 1H), 7.64 (d, *J* = 8.5 Hz, 1H), 7.60 – 7.55 (m, 2H), 7.54 – 7.42 (m, 8H), 7.42 – 7.36 (m, 1H), 7.24 (d, *J* = 4.2 Hz, 2H), 7.06 (d, *J* = 8.1 Hz, 1H), 1.86 (s, 3H); ^{13}C NMR (100 MHz, d_6-DMSO) δ ppm: 181.9, 166.1, 165.7, 153.3, 149.0, 138.1, 134.6, 134.4, 132.4, 132.1, 131.5, 130.7, 129.2, 129.1, 129.0, 128.5, 128.1, 127.9, 127.0, 126.9, 123.2, 123.0, 122.6, 121.4, 60.3, 22.3; HRMS (ESI) Calcd. For $C_{35}H_{28}N_3O_2^+$ [M+H]$^+$ 522.2176, found 522.2188.

(*S*)-*N,N'*-(4-(1,2,3,4-tetrahydro-4a*H*-carbazol-4a-yl)-1,2-phenylene)dibenzamide **3i:**

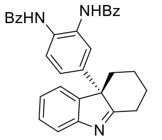

3i

Colorless oil; 44.6 mg; isolated yield = 92%; $[\alpha]_D^{20}$ = +23 (*c* 0.50, MeOH); HPLC (Chiralpak IC, *i*-propanol/hexane = 30/70, flow rate 1.0 mL/min, λ = 254 nm), t_1 = 20.37 min (minor), t_2 = 55.72 min (major), *ee* = 97%; ^{1}H NMR (400 MHz, d_6-DMSO) δ ppm: 10.12 (s, 1H), 10.01 (s, 1H), 8.00 – 7.88 (m, 4H), 7.68 (d, *J* = 8.5 Hz, 1H), 7.61 – 7.49 (m, 7H), 7.37 – 7.25 (m, 3H), 7.21 – 7.11 (m, 2H), 3.28 – 3.07 (m, 1H), 2.97 – 2.83 (m, 1H), 2.68 – 2.55 (m, 1H), 2.24 – 2.03 (m, 1H), 1.77 – 1.64 (m, 1H), 1.59 – 1.39 (m, 2H), 1.25 – 1.11 (m, 1H); ^{13}C NMR (100 MHz, d_6-DMSO) δ ppm: 188.4, 166.1, 165.8, 154.3, 147.4, 136.5, 134.6, 134.4, 132.4, 132.2, 130.5, 129.1, 129.0, 128.1, 128.0, 127.9, 127.1, 125.6, 123.8, 123.7, 122.9, 120.6, 62.5, 37.0, 30.5, 29.1, 22.0; HRMS (ESI) Calcd. For $C_{32}H_{28}N_3O_2^+$ [M+H]$^+$ 486.2176, found 486.2185.

(*S*)-*N,N'*-(4-(7,8,9,10-tetrahydrocyclohepta[*b*]indol-10a(6*H*)-yl)-1,2-phenylene)dibenz amide **3j:**

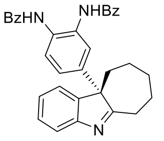

3j

Colorless oil; 41.4 mg; isolated yield = 83%; $[\alpha]_D^{20}$ = +100 (*c* 0.10, MeOH); HPLC (Chiralpak IA, *i*-propanol/hexane = 20/80, flow rate 1.0 mL/min, λ = 254 nm), t_1 = 14.21 min (major), t_2 = 19.29 min (minor), *ee* = 98%; ^{1}H NMR (400 MHz, d_6-DMSO) δ ppm: 10.11 (s, 1H), 10.01 (s, 1H), 7.96 – 7.89 (m, 4H), 7.64 (d, *J* = 8.5 Hz, 1H), 7.62 – 7.56 (m, 2H), 7.54 – 7.45 (m, 6H), 7.31 – 7.24 (m, 2H), 7.21 – 7.11 (m, 2H), 3.07 – 2.78 (m, 2H), 2.64 – 2.54 (m, 1H), 2.08 – 1.97 (m, 1H), 1.79 – 1.51 (m, 5H), 1.19 – 1.05 (m, 1H); ^{13}C NMR (100 MHz, d_6-DMSO) δ ppm: 189.9, 166.1, 165.8, 154.0, 146.6, 137.8, 134.6, 134.5, 132.4, 132.0, 130.4, 129.1, 129.0, 128.1, 127.9, 126.9, 125.9, 123.4, 123.2, 122.7, 120.0, 66.1, 34.4, 32.9, 29.7, 26.9, 25.4; HRMS (ESI) Calcd. For $C_{33}H_{30}N_3O_2^+$ [M+H]$^+$ 500.2333, found 500.2332.

(*S*)-*N,N'*-(4-(5-methoxy-2,3-dimethyl-3*H*-indol-3-yl)-1,2-phenylene)dibenzamide **3k:**

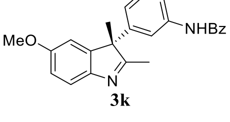

3k

White solid; 46.9 mg; isolated yield = 96%; M.p.: 126 – 127 °C; $[\alpha]_D^{20}$ = -264 (*c* 0.20, MeOH); HPLC (Chiralpak Ik-3, *i*-propanol/hexane = 30/70, flow rate 1.0 mL/min, λ = 254 nm), t_1 = 14.42 min (major), t_2 = 22.31 min (minor), *ee* = 94%; ^{1}H NMR (400 MHz, d_6-DMSO) δ ppm: 10.15 (s, 1H), 9.99 (s, 1H), 8.02 – 7.86 (m, 4H), 7.65 (d, *J* = 8.5 Hz, 1H), 7.61 – 7.55 (m, 2H), 7.55 – 7.47 (m, 4H), 7.41 (d, *J* = 7.8 Hz, 1H), 7.30 (d, *J* = 1.2 Hz, 1H), 7.12 (d, *J* = 7.8 Hz, 1H), 7.07 – 6.97 (m, 2H), 2.29 (s, 3H), 2.12 (s, 3H), 1.66 (s, 3H); ^{13}C NMR (100 MHz, d_6-DMSO) δ ppm: 189.1, 170.9, 170.5, 163.0, 153.5,

152.9, 142.2, 139.4, 139.2, 137.2, 137.1, 136.6, 135.5, 133.8, 132.8, 132.6, 131.4, 128.5, 128.4, 125.3, 118.0, 114.0, 66.4, 60.7, 25.4, 20.7; HRMS (ESI) Calcd. For $C_{31}H_{28}N_3O_3^+$ $[M+H]^+$ 490.2125, found 490.2128.

(*S*)-*N,N'*-(4-(2,3,5-trimethyl-3*H*-indol-3-yl)-1,2-phenylene)dibenzamide **3l:**

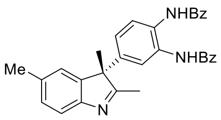

3l

Yellow oil; 42.6 mg; isolated yield = 90%; $[\alpha]_D^{20}$ = -16 (*c* 0.80, MeOH); HPLC (Chiralpak IE, *i*-propanol/hexane = 20/80, flow rate 1.0 mL/min, λ = 254 nm), t_1 = 16.68 min (minor), t_2 = 18.83 min (major), *ee* = 98%; ^{1}H NMR (400 MHz, d_6-DMSO) δ ppm: 10.15 (s, 1H), 9.99 (s, 1H), 8.02 – 7.86 (m, 4H), 7.65 (d, *J* = 8.5 Hz, 1H), 7.61 – 7.55 (m, 2H), 7.55 – 7.47 (m, 4H), 7.41 (d, *J* = 7.8 Hz, 1H), 7.30 (d, *J* = 1.2 Hz, 1H), 7.12 (d, *J* = 7.8 Hz, 1H), 7.07 – 6.97 (m, 2H), 2.29 (s, 3H), 2.12 (s, 3H), 1.66 (s, 3H); ^{13}C NMR (100 MHz, d_6-DMSO) δ ppm: 185.6, 166.1, 165.7, 152.5, 147.5, 137.5, 135.3, 134.6, 134.4, 132.4, 131.9, 130.7, 129.1, 129.0, 128.8, 128.1, 127.9, 126.8, 123.6, 119.8, 61.3, 21.4, 20.6, 16.0; HRMS (ESI) Calcd. For $C_{31}H_{28}N_3O_2^+$ $[M+H]^+$ 474.2176, found 474.2186.

(*S*)-*N,N'*-(4-(5-fluoro-2,3-dimethyl-3*H*-indol-3-yl)-1,2-phenylene)dibenzamide **3m:**

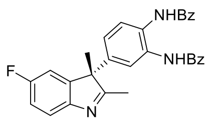

3m

Yellow oil; 40.5 mg; isolated yield = 85%; $[\alpha]_D^{20}$ = -69 (*c* 0.50, MeOH); HPLC (Chiralpak IC, *i*-propanol/hexane = 30/70, flow rate 1.0 mL/min, λ = 254 nm), t_1 = 19.23 min (major), t_2 = 44.31 min (minor), *ee* = >99%; ^{1}H NMR (400 MHz, d_6-DMSO) δ ppm: 10.13 (s, 1H), 10.01 (s, 1H), 7.96 – 7.86 (m, 4H), 7.66 (d, *J* = 8.5 Hz, 1H), 7.60 – 7.47 (m, 7H), 7.29 (s, 1H), 7.20 – 7.11 (m, 2H), 7.08 – 7.01 (m, 1H), 2.13 (s, 3H), 1.69 (s, 3H); ^{13}C NMR (100 MHz, d_6-DMSO) δ ppm: 187.0, 166.2, 165.8, 161.1 (d, *J* = 240 Hz), 150.8, 149.3, 149.2, 136.7, 134.6, 134.4, 132.5, 132.4, 131.9, 130.9, 129.1, 129.0, 128.1, 127.9, 126.8, 123.7, 123.6, 121.2 (d, *J* = 9 Hz), 121.1, 114.9, 110.8 (d, *J* = 25 Hz), 62.1, 20.4, 16.1; ^{19}F NMR (376 MHz, d_6-DMSO) δ -116.86; HRMS (ESI) Calcd. For $C_{30}H_{25}FN_3O_2^+$ $[M+H]^+$ 478.1925, found 478.1929.

(*S*)-*N,N'*-(4-(5-chloro-2,3-dimethyl-3*H*-indol-3-yl)-1,2-phenylene)dibenzamide **3n:**

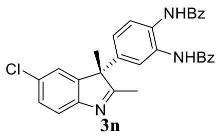

3n

White solid; 44.4 mg; isolated yield = 90%; M.p.: 136 – 137 °C; $[\alpha]_D^{20}$ = -139 (*c* 0.20, MeOH); HPLC (Chiralpak IC, *i*-propanol/hexane = 30/70, flow rate 1.0 mL/min, λ = 254 nm), t_1 = 15.46 min (major), t_2 = 22.88 min (minor), *ee* = 97%; ^{1}H NMR (400 MHz,

d_6-DMSO) δ ppm: 10.13 (s, 1H), 10.01 (s, 1H), 7.94 – 7.88 (m, 4H), 7.66 (d, *J* = 8.5 Hz, 1H), 7.59 – 7.47 (m, 7H), 7.29 (s, 1H), 7.20 – 7.12 (m, 2H), 7.07 – 7.01 (m, 1H), 2.13 (s, 3H), 1.69 (s, 3H); ^{13}C NMR (100 MHz, d_6-DMSO) δ ppm: 187.8, 166.2, 165.8, 153.4, 149.2, 136.5, 134.6, 134.4, 132.5, 132.4, 131.9, 131.0, 130.6, 129.1, 129.0, 128.4, 128.1, 127.9, 126.8, 123.6, 123.4, 121.6, 62.0, 20.2, 16.1; HRMS (ESI) Calcd. For $C_{30}H_{25}ClN_3O_2^+$ [M+H]$^+$ 494.1630, found 494.1641.

(*S*)-*N,N'*-(4-(5-bromo-2,3-dimethyl-3*H*-indol-3-yl)-1,2-phenylene)dibenzamide **3o:**

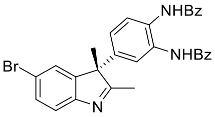

3o

White solid; 50.5 mg; isolated yield = 94%; M.p.: 125 – 126 °C; $[\alpha]_D^{20}$ = -187 (*c* 0.20, MeOH); HPLC (Chiralpak IC, *i*-propanol/hexane = 30/70, flow rate 1.0 mL/min, λ = 254 nm), t_1 = 17.79 min (major), t_2 = 23.51 min (minor), *ee* = 99%; ^{1}H NMR (400 MHz, d_6-DMSO) δ ppm: 10.15 (s, 1H), 10.01 (s, 1H), 7.98 – 7.85 (m, 4H), 7.68 (d, *J* = 8.5 Hz, 1H), 7.61 – 7.55 (m, 2H), 7.55 – 7.41 (m, 7H), 7.30 (d, *J* = 1.4 Hz, 1H), 7.08 – 7.00 (m, 1H), 2.15 (s, 3H), 1.69 (s, 3H); ^{13}C NMR (100 MHz, d_6-DMSO) δ ppm: 187.8, 165.8, 153.8, 149.6, 136.4, 134.6, 134.4, 132.4, 131.9, 131.3, 131.0, 129.1, 129.0, 128.1, 127.9, 126.8, 126.2, 123.6, 122.1, 118.9, 62.0, 20.2, 16.2; HRMS (ESI) Calcd. For $C_{30}H_{25}BrN_3O_2^+$ [M+H]$^+$ 538.1125, found 538.1136.

(*S*)-*N,N'*-(4-(2,3-dimethyl-5-nitro-3*H*-indol-3-yl)-1,2-phenylene)dibenzamide **3p:**

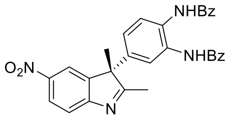

3p

Yellow solid; 40.8 mg; isolated yield = 81%; M.p.: 102 – 103 °C ; $[\alpha]_D^{20}$ = -237 (*c* 0.17, MeOH); HPLC (Chiralpak Ik-3, *i*-propanol/hexane = 30/70, flow rate 1.0 mL/min, λ = 254 nm), t_1 = 24.82 min (major), t_2 = 46.80 min (minor), *ee* = 98%; ^{1}H NMR (400 MHz, d_6-DMSO) δ ppm: 10.13 (s, 1H), 10.02 (s, 1H), 8.36 – 8.23 (m, 1H), 8.14 (d, *J* = 2.2 Hz, 1H), 8.03 – 7.84 (m, 4H), 7.79 (d, *J* = 8.5 Hz, 1H), 7.70 (d, *J* = 8.5 Hz, 1H), 7.65 – 7.43 (m, 6H), 7.33 (s, 1H), 7.17 – 7.08 (m, 1H), 2.25 (s, 3H), 1.79 (s, 3H); ^{13}C NMR (100 MHz, d_6-DMSO) δ ppm: 198.4, 170.9, 170.6, 164.8, 153.1, 150.6, 140.3, 139.3, 139.1, 137.2, 136.8, 136.0, 133.8, 132.8, 132.6, 131.7, 130.0, 128.5, 128.3, 125.6, 123.3, 67.1, 24.7, 21.5; HRMS (ESI) Calcd. For $C_{30}H_{25}N_4O_4^+$ [M+H]$^+$ 505.1870, found 505.1876.

(*S*)-*N,N'*-(4-(2,3,7-trimethyl-3*H*-indol-3-yl)-1,2-phenylene)dibenzamide **3q:**

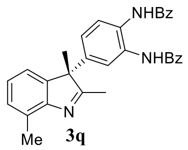

3q

White solid; 40.2 mg; isolated yield = 85%; M.p.: 101 – 102 °C; $[\alpha]_D^{20}$ = -38 (*c* 0.90, MeOH); HPLC (Chiralpak IC, *i*-propanol/hexane = 30/70, flow rate 1.0 mL/min, λ = 254 nm), t_1 = 16.34 min (major), t_2 = 21.46 min (minor), *ee* = 97%; ^{1}H NMR (400 MHz, d_6-DMSO) δ ppm: 10.15 (s, 1H), 9.99 (s, 1H), 7.97 – 7.88 (m, 4H), 7.64 (d, *J* = 8.5 Hz, 1H), 7.61 – 7.55 (m, 2H), 7.55 – 7.47 (m, 4H), 7.28 (d, *J* = 1.2 Hz, 1H), 7.14 (d, *J* = 7.3 Hz, 1H), 7.11 – 6.98 (m, 3H), 2.52 (s, 3H), 2.15 (s, 3H), 1.65 (s, 3H); ^{13}C NMR (100 MHz, d_6-DMSO) δ ppm: 185.6, 165.8, 153.0, 147.0, 137.5, 134.6, 134.4, 132.5, 132.4, 131.8, 130.7, 129.5, 129.4, 129.1, 129.0, 128.1, 127.9, 126.7, 126.0, 123.7, 123.6, 120.4, 61.8, 20.7, 17.1, 16.1; HRMS (ESI) Calcd. For $C_{31}H_{28}N_3O_2^+$ $[M+H]^+$ 474.2176, found 474.2186.

(*S*)-*N,N'*-(4-(7-fluoro-2,3-dimethyl-3*H*-indol-3-yl)-1,2-phenylene)dibenzamide **3r:**

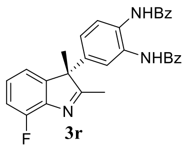

White solid; 41.5 mg; isolated yield = 87%; M.p.: 110 – 111 °C; $[\alpha]_D^{20}$ = -83 (*c* 0.68, MeOH); HPLC (Chiralpak IC, *i*-propanol/hexane = 30/70, flow rate 1.0 mL/min, λ = 254 nm), t_1 = 34.46 min (major), t_2 = 54.29 min (minor), *ee* = 98%; ^{1}H NMR (400 MHz, d_6-DMSO) δ ppm: 10.13 (s, 1H), 10.00 (s, 1H), 7.98 – 7.85 (m, 4H), 7.67 (d, *J* = 8.5 Hz, 1H), 7.63 – 7.56 (m, 2H), 7.55 – 7.46 (m, 4H), 7.31 (d, *J* = 1.5 Hz, 1H), 7.26 – 7.15 (m, 2H), 7.12 – 6.98 (m, 2H), 2.18 (s, 3H), 1.70 (s, 3H); ^{13}C NMR (100 MHz, d_6-DMSO) δ ppm: 187.9, 166.2, 165.8, 153.4 (d, *J* = 250 Hz), 150.7, 150.6, 141.0, 140.9, 136.5, 134.5, 132.4, 131.9, 131.0, 130.1, 129.1 (d, *J* = 8 Hz), 128.1, 127.8, 126.8, 123.6, 123.5, 119.2, 115.5 (d, *J* = 18 Hz), 62.4, 20.5, 16.3; ^{19}F NMR (376 MHz, d_6-DMSO) δ ppm: -127.34; HRMS (ESI) Calcd. For $C_{30}H_{25}FN_3O_2^+$ $[M+H]^+$ 478.1925, found 478.1930.

(*S*)-*N,N'*-(4-(7-bromo-2,3-dimethyl-3*H*-indol-3-yl)-1,2-phenylene)dibenzamide **3s:**

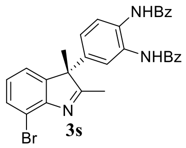

White solid; 46.2 mg; isolated yield = 86%; M.p.: 112 – 113 °C ; $[\alpha]_D^{20}$ = -33 (*c* 0.15, MeOH); HPLC (Chiralpak IC, *i*-propanol/hexane = 30/70, flow rate 1.0 mL/min, λ = 254 nm), t_1 = 18.56 min (major), t_2 = 26.37 min (minor), *ee* = 99%; ^{1}H NMR (400 MHz, d_6-DMSO) δ ppm: 10.15 (s, 1H), 10.01 (s, 1H), 7.98 – 7.85 (m, 4H), 7.68 (d, *J* = 8.5 Hz, 1H), 7.61 – 7.55 (m, 2H), 7.55 – 7.41 (m, 7H), 7.30 (d, *J* = 1.4 Hz, 1H), 7.08 – 7.00 (m, 1H), 2.15 (s, 3H), 1.70 (s, 3H); ^{13}C NMR (100 MHz, d_6-DMSO) δ ppm: 187.8, 166.2, 165.8, 153.8, 149.6, 136.4, 134.6, 134.4, 132.4, 131.9, 131.3, 1301.0, 129.1, 129.0, 128.1, 127.9, 126.8, 126.2, 123.6 , 122.1, 118.9, 62.0, 20.2, 16.2; HRMS (ESI) Calcd. For $C_{30}H_{25}BrN_3O_2^+$ $[M+H]^+$ 538.1125, found 538.1122.

(*S*)-*N,N'*-(4-(2,3-dimethyl-3*H*-indol-3-yl)-5-methyl-1,2-phenylene)dibenzamide **3t:**

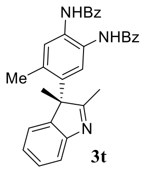

3t

White solid; 38.3 mg; isolated yield = 81%; M.p.: 155 – 156 °C; $[\alpha]_D^{20}$ = +2 (*c* 0.33, MeOH); HPLC (Chiralpak IC-3, *i*-propanol/hexane = 30/70, flow rate 1.0 mL/min, λ = 254 nm), t_1 = 9.21 min (major), t_2 = 10.83 min (minor), *ee* = 94%; ^{1}H NMR (400 MHz, d_6-DMSO) δ ppm: 10.29 (s, 1H), 10.04 (s, 1H), 8.03 (s, 3H), 7.93 (d, *J* = 7.6 Hz, 2H), 7.68 – 7.47 (m, 7H), 7.42 – 7.33 (m, 2H), 7.23 – 7.15 (m, 1H), 7.06 (d, *J* = 7.3 Hz, 1H), 2.08 (s, 3H), 1.59 (s, 3H), 1.34 (s, 3H); ^{13}C NMR (100 MHz, d_6-DMSO) δ ppm: 186.3, 166.2, 165.7, 155.0, 146.1, 134.6, 134.1, 132.4, 130.7, 129.5, 129.1, 128.7, 128.3, 128.1, 127.8, 126.1, 125.6, 122.1, 120.5, 61.6, 24.8, 18.0, 16.2; HRMS (ESI) Calcd. For $C_{31}H_{28}N_3O_2^+$ $[M+H]^+$ 474.2176, found 474.2185.

(*R*)-*N,N'*-(4-chloro-5-(2,3-dimethyl-3*H*-indol-3-yl)-1,2-phenylene)dibenzamide **3u:**

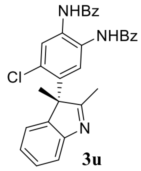

3u

White solid; 38.9 mg; isolated yield = 79%; M.p.: 162 – 163 °C; $[\alpha]_D^{20}$ = +40 (*c* 0.28, MeOH); HPLC (Chiralpak IK-3, *i*-propanol/hexane = 30/70, flow rate 1.0 mL/min, λ = 254 nm), t_1 = 5.60 min (minor), t_2 = 6.21 min (major), *ee* = 98%; ^{1}H NMR (400 MHz, d_6-DMSO) δ ppm: 10.34 (s, 1H), 10.11 (s, 1H), 8.17 (s, 1H), 8.03 (d, *J* = 7.3 Hz, 2H), 7.95 – 7.89 (m, 2H), 7.72 (s, 1H), 7.68 – 7.47 (m, 7H), 7.37 – 7.31 (m, 1H), 7.20 – 7.14 (m, 1H), 7.05 (d, *J* = 7.2 Hz, 1H), 2.11 (s, 3H), 1.58 (s, 3H); ^{13}C NMR (100 MHz, d_6-DMSO) δ ppm: 185.2, 166.5, 166.0, 155.4, 145.5, 134.4, 133.8, 132.6, 132.2, 130.5, 129.1, 128.8, 128.2, 128.0, 127.2, 125.9, 121.8, 120.4, 61.1, 24.8, 16.3; HRMS (ESI) Calcd. For $C_{30}H_{25}ClN_3O_2^+$ $[M+H]^+$ 494.1630, found 494.1641.

(*R*)-*N,N'*-(4-bromo-5-(2,3-dimethyl-3*H*-indol-3-yl)-1,2-phenylene)dibenzamide **3v:**

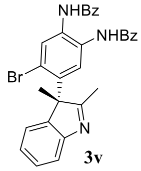

3v

White solid; 46.2 mg; isolated yield = 86%; M.p.: 166 – 167 °C; $[\alpha]_D^{20}$ = +16 (*c* 0.60, MeOH); HPLC (Chiralpak IK-3, *i*-propanol/hexane = 30/70, flow rate 1.0 mL/min, λ = 254 nm), t_1 = 5.32 min (minor), t_2 = 6.24 min (major), *ee* = >99%; ^{1}H NMR (400 MHz, d_6-DMSO) δ ppm: 10.32 (s, 1H), 10.12 (s, 1H), 8.18 (s, 1H), 8.02 (d, *J* = 7.3 Hz, 2H), 7.95 – 7.87 (m, 3H), 7.65 – 7.51 (m, 7H), 7.39 – 7.31 (m, 1H), 7.22 – 7.12 (m, 1H), 7.03 (d, *J* = 7.2 Hz, 1H), 2.13 (s, 3H), 1.58 (s, 3H); ^{13}C NMR (100 MHz, d_6-DMSO) δ ppm: 185.2, 166.4, 166.0, 155.7, 145.6, 135.2, 134.4, 132.6, 132.3, 131.0, 130.9, 129.1,

128.2, 128.0, 127.5, 125.9, 121.8, 120.5, 118.6, 62.4, 25.6, 16.4; HRMS (ESI) Calcd. For $C_{30}H_{25}BrN_3O_2^+$ [M+H]$^+$ 538.1125, found 538.1133.

(*S*)-*N,N'*-(4-(2,3-dimethyl-3*H*-indol-3-yl)-1,2-phenylene)bis(4-methylbenzamide) **3w:**

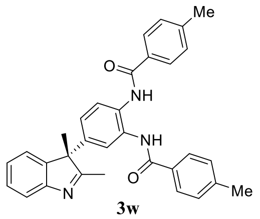

3w

White solid; 41.4 mg; isolated yield = 85%; M.p.: 154 – 155 °C; $[\alpha]_D^{20}$ = -123 (*c* 0.50, MeOH); HPLC (Chiralpak IC, *i*-propanol/hexane = 30/70, flow rate 1.0 mL/min, λ = 254 nm), t_1 = 24.62 min (major), t_2 = 34.61 min (minor), *ee* = 98%; ^{1}H NMR (400 MHz, DMSO) δ ppm: 10.09 (s, 1H), 9.93 (s, 1H), 7.86 – 7.78 (m, 4H), 7.63 (d, *J* = 8.5 Hz, 1H), 7.54 (d, *J* = 7.7 Hz, 1H), 7.35 – 7.28 (m, 6H), 7.25 – 7.16 (m, 2H), 7.03 – 6.98 (m, 1H), 2.36 (s, 6H), 2.14 (s, 3H), 1.67 (s, 3H); ^{13}C NMR (100 MHz, DMSO) δ ppm: 186.7, 166.0, 165.6, 154.7, 147.2, 142.6, 142.5, 137.2, 131.9, 131.7, 131.5, 130.8, 129.6, 128.3, 128.1, 127.9, 126.6, 126.0, 123.6, 123.5, 123.0, 120.2, 61.5, 21.5, 21.4, 20.6, 16.1; HRMS (ESI) Calcd. For $C_{32}H_{30}N_3O_2^+$ [M+H]$^+$ 488.2333, found 488.2347.

(*S*)-*N,N'*-(4-(2,3-dimethyl-3*H*-indol-3-yl)-1,2-phenylene)bis(4-methoxybenzamide) **3x:**

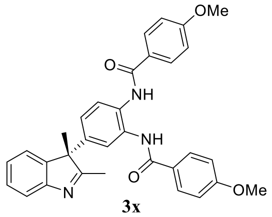

3x

Colorless oil; 46.7 mg; isolated yield = 90%; $[\alpha]_D^{20}$ = -107 (*c* 0.53, MeOH); HPLC (Chiralpak IC-3, *i*-propanol/hexane = 30/70, flow rate 1.0 mL/min, λ = 254 nm), t_1 = 44.85 min (major), t_2 = 58.82 min (minor), *ee* = 94%; ^{1}H NMR (400 MHz, d_6-DMSO) δ ppm: 10.05 (s, 1H), 9.91 (s, 1H), 7.97 – 7.87 (m, 4H), 7.63 (d, *J* = 8.5 Hz, 1H), 7.54 (d, *J* = 7.7 Hz, 1H), 7.36 – 7.28 (m, 2H), 7.26 – 7.15 (m, 2H), 7.08 – 7.03 (m, 4H), 7.02 – 6.97 (m, 1H), 3.82 – 3.81 (m, 6H), 2.14 (s, 3H), 1.67 (s, 3H); ^{13}C NMR (100 MHz, d_6-DMSO) δ ppm: 186.7, 165.6, 165.2, 162.7, 162.6, 154.7, 147.2, 137.0, 131.9, 130.8, 130.0, 129.8, 128.3, 126.6, 126.4, 126.0, 123.5, 123.0, 120.2, 114.4, 114.3, 61.5, 55.9, 20.6, 16.1; HRMS (ESI) Calcd. For $C_{32}H_{30}N_3O_4^+$ [M+H]$^+$ 520.2231, found 520.2236.

(*S*)-*N,N'*-(4-(2,3-dimethyl-3*H*-indol-3-yl)-1,2-phenylene)bis(4-fluorobenzamide) **3y:**

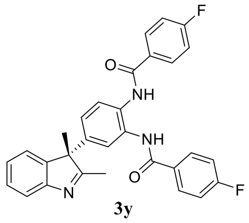

3y

Yellow solid; 40.6 mg; isolated yield = 82%; M.p.: 189 – 190 °C; $[\alpha]_D^{20}$ = -142 (*c* 0.70, MeOH); HPLC (Chiralpak IC-3, *i*-propanol/hexane = 30/70, flow rate 1.0 mL/min, λ = 254 nm), t_1 = 7.59 min (major), t_2 = 9.43 min (minor), *ee* = 94%; ^{1}H NMR (400 MHz, d_6-DMSO) δ ppm: 10.15 (s, 1H), 10.02 (s, 1H), 8.07 – 7.94 (m, 4H), 7.66 (d, *J* = 8.5 Hz, 1H), 7.56 (d, *J* = 7.7 Hz, 1H), 7.41 – 7.30 (m, 6H), 7.27 – 7.16 (m, 2H), 7.07 – 6.99 (m, 1H), 2.16 (s, 3H), 1.69 (s, 3H); ^{13}C NMR (100 MHz, d_6-DMSO) δ ppm: 186.7, 165.9, 164.8, 164.7 (d, *J* = 248 Hz), 164.3 (d, *J* = 248 Hz), 154.7, 147.2, 137.3, 131.9, 131.1, 130.9 (d, *J* = 9 Hz), 130.8 (d, *J* = 8 Hz), 130.6, 128.3, 126.8, 126.0, 123.7, 123.0, 120.2, 116.1 (d, *J* = 22 Hz), 116.0 (d, *J* = 21 Hz), 61.5, 20.6, 16.1; ^{19}F NMR (376 MHz, d_6-DMSO) δ ppm: -108.21, -108.29; HRMS (ESI) Calcd. For $C_{30}H_{23}F_2N_3O_2Na^+$ $[M+Na]^+$ 518.1651, found 518.1649.

(*S*)-*N,N'*-(4-(2,3-dimethyl-3*H*-indol-3-yl)-1,2-phenylene)bis(4-chlorobenzamide) **3z:**

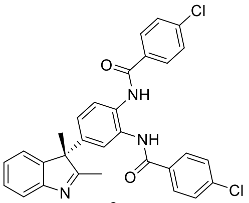

3z

White solid; 40.1 mg; isolated yield = 76%; M.p.: 201 – 202 °C; $[\alpha]_D^{20}$ = -104 (*c* 0.63, MeOH); HPLC (Chiralpak IC-3, *i*-propanol/hexane = 30/70, flow rate 1.0 mL/min, λ = 254 nm), t_1 = 6.93 min (major), t_2 = 8.34 min (minor), *ee* = 94%;^{1}H NMR (400 MHz, d_6-DMSO) δ ppm: 10.16 (s, 1H), 10.03 (s, 1H), 7.98 – 7.91 (m, 4H), 7.66 – 7.53 (m, 6H), 7.36 – 7.28 (m, 2H), 7.25 – 7.15 (m, 2H), 7.06 – 6.99 (m, 1H), 2.14 (s, 3H), 1.67 (s, 3H); ^{13}C NMR (100 MHz, d_6-DMSO) δ ppm: 186.7, 165.1, 164.8, 154.7, 147.2, 137.4, 137.2, 137.1, 133.5, 133.3, 131.9, 130.8, 130.1, 129.9, 129.1, 129.0, 128.3, 126.9, 126.0, 123.7, 123.0, 120.2, 61.5, 20.6, 16.1; HRMS (ESI) Calcd. For $C_{30}H_{24}Cl_2N_3O_2^+$ $[M+H]^+$ 528.1240, found 528.1245.

(*S*)-*N,N'*-(4-(2,3-dimethyl-3*H*-indol-3-yl)-1,2-phenylene)bis(4-bromobenzamide) **3a':**

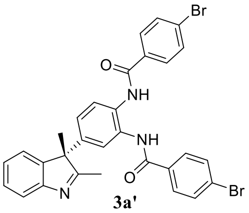

3a'

Yellow solid; 53.7 mg; isolated yield = 87%; M.p.: 216 – 217 °C; $[\alpha]_D^{20}$ = -55 (*c* 0.80, MeOH); HPLC (Chiralpak IC-3, *i*-propanol/hexane = 30/70, flow rate 1.0 mL/min, λ = 254 nm), t_1 = 7.03 min (major), t_2 = 8.35 min (minor), *ee* = 94%;^{1}H NMR (400 MHz, d_6-DMSO) δ ppm: 10.16 (s, 1H), 10.03 (s, 1H), 7.92 – 7.83 (m, 4H), 7.75 – 7.69 (m, 4H), 7.64 (d, *J* = 8.5 Hz, 1H), 7.54 (d, *J* = 7.7 Hz, 1H), 7.37 – 7.27 (m, 2H), 7.24 – 7.15 (m, 2H), 7.05 – 6.99 (m, 1H), 2.14 (s, 3H), 1.67 (s, 3H);^{13}C NMR (100 MHz, d_6-DMSO) δ ppm: 186.7, 165.2, 164.9, 154.7, 147.2, 137.4, 133.9, 133.7, 132.0, 131.9, 130.7,

130.3, 130.1, 128.3, 126.9, 126.1, 126.0, 123.7, 123.0, 120.2, 61.5, 20.6, 16.1; HRMS (ESI) Calcd. For $C_{30}H_{24}Br_2N_3O_2^+$ $[M+H]^+$ 618.0209, found 618.0222.

(*S*)-*N,N*'-(4-(2,3-dimethyl-3*H*-indol-3-yl)-1,2-phenylene)bis(4-nitrobenzamide) **3b':**

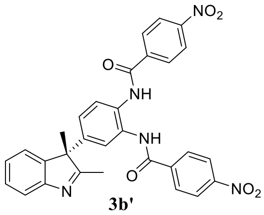

3b'

White solid; 40.1 mg; isolated yield = 73%; M.p.: 90 – 91 °C; $[\alpha]_D^{20}$ = -63 (*c* 0.33, MeOH); HPLC (Chiralpak IF, *i*-propanol/hexane = 20/80, flow rate 1.0 mL/min, λ = 254 nm), t_1 = 22.40 min (minor), t_2 = 26.24 min (major), *ee* = 97%; ^{1}H NMR (400 MHz, d_6-DMSO) δ ppm: 10.33 (s, 1H), 10.23 (s, 1H), 8.40 – 8.30 (m, 4H), 8.20 – 8.09 (m, 4H), 7.65 (d, *J* = 8.4 Hz, 1H), 7.54 (d, *J* = 7.6 Hz, 1H), 7.38 – 7.29 (m, 2H), 7.27 – 7.16 (m, 2H), 7.08 – 7.02 (m, 1H), 2.14 (s, 3H), 1.68 (s, 3H); ^{13}C NMR (100 MHz, d_6-DMSO) δ ppm: 186.6, 164.5, 164.4, 154.7, 149.7, 147.1, 140.6, 137.7, 131.9, 130.7, 129.8, 129.6, 128.3, 127.1, 126.1, 124.1, 124.0, 123.0, 120.2, 61.5, 20.6, 16.1; HRMS (ESI) Calcd. For $C_{30}H_{24}N_5O_6^+$ $[M+H]^+$ 550.1721, found 550.1728.

(*S*)-*N,N*'-(4-(2,3-dimethyl-3*H*-indol-3-yl)-1,2-phenylene)bis(3-methylbenzamide) **3c':**

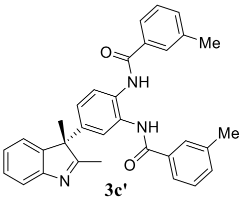

3c'

White solid; 42.4 mg; isolated yield = 87%; M.p.: 102 – 103 °C; $[\alpha]_D^{20}$ = -165 (*c* 0.60, MeOH); HPLC (Chiralpak IC-3, *i*-propanol/hexane = 30/70, flow rate 1.0 mL/min, λ = 254 nm), t_1 = 26.16 min (major), t_2 = 38.76 min (minor), *ee* = 97%; ^{1}H NMR (400 MHz, DMSO) δ ppm: 10.13 (s, 1H), 9.97 (s, 1H), 7.80 – 7.70 (m, 4H), 7.67 (d, *J* = 8.5 Hz, 1H), 7.55 (d, *J* = 7.7 Hz, 1H), 7.45 – 7.30 (m, 6H), 7.27 – 7.15 (m, 2H), 7.07 – 7.01 (m, 1H), 2.35 – 2.34 (m, 6H), 2.15 (s, 3H), 1.68 (s, 3H); ^{13}C NMR (100 MHz, DMSO) δ ppm: 186.7, 166.2, 165.9, 154.7, 147.2, 138.4, 138.3, 137.3, 134.6, 134.4, 133.0, 131.9, 130.8, 129.0, 128.9, 128.6, 128.4, 128.3, 126.6, 126.0, 125.2, 125.0, 123.7, 123.5, 123.0, 120.2, 61.5, 21.4, 20.6, 16.1; HRMS (ESI) Calcd. For $C_{32}H_{30}N_3O_2^+$ $[M+H]^+$ 488.2333, found 488.2329.

(*S*)-*N,N*'-(4-(2,3-dimethyl-3*H*-indol-3-yl)-1,2-phenylene)bis(3-fluorobenzamide) **3d':**

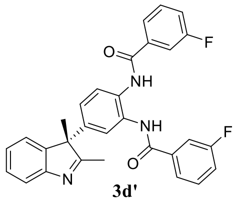

3d'

White solid; 42.6 mg; isolated yield = 86%; M.p.: 96 – 97 °C; $[\alpha]_D^{20}$ = -124 (*c* 0.53, MeOH); HPLC (Chiralpak IC, *i*-propanol/hexane = 30/70, flow rate 1.0 mL/min, λ = 254 nm), t_1 = 7.80 min (major), t_2 = 9.68 min (minor), *ee* = 98%; ^{1}H NMR (400 MHz, d_6-DMSO) δ ppm: 10.13 (s, 1H), 10.02 (s, 1H), 7.80 – 7.67 (m, 4H), 7.64 (d, *J* = 8.5 Hz, 1H), 7.60 – 7.51 (m, 3H), 7.46 – 7.39 (m, 2H), 7.37 – 7.27 (m, 2H), 7.26 – 7.15 (m, 2H), 7.06 – 6.99 (m, 1H), 2.14 (s, 3H), 1.67 (s, 3H); ^{13}C NMR (100 MHz, d_6-DMSO) δ ppm: 186.7, 164.8, 164.6, 162.3 (d, *J* = 243 Hz), 162.4 (d, *J* = 243 Hz), 154.7, 147.2, 137.5, 137.2, 137.1, 137.0, 136.9, 131.9, 131.3, 131.2 (d, *J* = 8 Hz), 131.1 (d, *J* = 8 Hz), 130.7, 128.3, 126.9, 126.1, 124.2, 123.8, 123.0, 120.2, 119.2, 115.2, 115.0, 114.8 (d, *J* = 23 Hz), 61.5, 20.6, 16.1; ^{19}F NMR (376 MHz, d_6-DMSO) δ ppm: -112.46, -112.53; HRMS (ESI) Calcd. For $C_{30}H_{24}F_2N_3O_2^+$ $[M+H]^+$ 496.1831, found 496.1837.

(*S*)-*N*,*N*'-(4-(2,3-dimethyl-3*H*-indol-3-yl)-1,2-phenylene)bis(2-fluorobenzamide) **3e':**

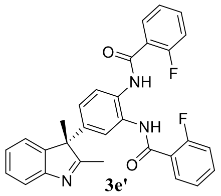

3e'

White solid; 33.7 mg; isolated yield = 68%; M.p.: 165 – 166 °C; $[\alpha]_D^{20}$ = -86 (*c* 0.73, MeOH); HPLC (Chiralpak IC-3, *i*-propanol/hexane = 30/70, flow rate 1.0 mL/min, λ = 254 nm), t_1 = 34.06 min (major), t_2 = 48.37 min (minor), *ee* = 95%; ^{1}H NMR (400 MHz, d_6-DMSO) δ ppm: 9.97 (s, 1H), 9.83 (s, 1H), 7.86 – 7.77 (m, 2H), 7.70 (d, *J* = 8.4 Hz, 1H), 7.63 – 7.54 (m, 3H), 7.40 – 7.29 (m, 6H), 7.27 – 7.16 (m, 2H), 7.05 (d, *J* = 8.1 Hz, 1H), 2.15 (s, 3H), 1.68 (s, 3H); ^{13}C NMR (100 MHz, d_6-DMSO) δ ppm: 186.7, 163.2, 163.0, 159.8 (d, *J* = 249 Hz), 159.8 (d, *J* = 249 Hz), 154.7, 147.2, 137.7, 133.9 (d, *J* = 8 Hz), 133.8 (d, *J* = 8 Hz), 131.4, 131.0, 130.1, 128.3, 126.5, 126.0, 125.3, 125.2, 123.9, 123.6, 123.5, 123.4, 123.2, 123.0, 120.2, 116.8 (d, *J* = 23 Hz), 61.5, 20.6, 16.1; ^{19}F NMR (376 MHz, d_6-DMSO) δ ppm: -113.54, -113.74; HRMS (ESI) Calcd. For $C_{30}H_{24}F_2N_3O_2^+$ $[M+H]^+$ 496.1831, found 496.1841.

(*S*)-*N*,*N*'-(4-(2,3-dimethyl-3*H*-indol-3-yl)-1,2-phenylene)bis(1-naphthamide) **3f':**

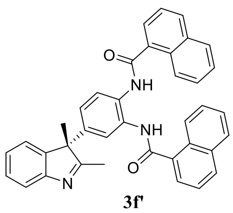

3f'

White solid; 50.9 mg; isolated yield = 91%; M.p.: 177 – 178 °C; $[\alpha]_D^{20}$ = -94 (*c* 0.67, MeOH); HPLC (Chiralpak IC-3, *i*-propanol/hexane = 30/70, flow rate 1.0 mL/min, λ = 254 nm), t_1 = 25.57 min (major), t_2 = 32.46 min (minor), *ee* = 92%; ^{1}H NMR (400 MHz, d_6-DMSO) δ ppm: 10.20 (s, 1H), 10.10 (s, 1H), 8.27 – 8.15 (m, 2H), 8.06 (d, *J* = 8.2 Hz, 2H), 7.99 (d, *J* = 8.2 Hz, 2H), 7.90 – 7.79 (m, 3H), 7.62 – 7.50 (m, 6H), 7.45 – 7.34 (m, 3H), 7.30 (d, *J* = 7.1 Hz, 1H), 7.27 – 7.20 (m, 1H), 7.09 (d, *J* = 8.0 Hz, 1H), 2.22 (s, 3H), 1.74 (s, 3H); ^{13}C NMR (100 MHz, d_6-DMSO) δ ppm: 186.8, 167.9, 167.8, 154.8,

147.2, 137.5, 134.6, 134.3, 133.6, 131.8, 131.1, 131.0, 130.5, 130.1, 128.8, 128.3, 127.4, 126.9, 126.8, 126.7, 126.4, 126.1, 125.5, 125.4, 123.7, 123.5, 123.1, 120.3, 61.6, 20.8, 16.2; HRMS (ESI) Calcd. For $C_{38}H_{30}N_3O_2^+$ $[M+H]^+$ 560.2333, found 560.2342.

Di-*tert*-butyl (4-(2,3-dimethyl-3*H*-indol-3-yl)-1,2-phenylene) (*S*)-dicarbamate **3g':**

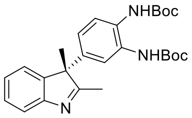

3g'

White solid; 42.8 mg; isolated yield = 95%; M.p.: 96 – 97 °C; $[\alpha]_D^{20}$ = -78 (*c* 0.75, MeOH); HPLC (Chiralpak IC, *i*-propanol/hexane = 30/70, flow rate 1.0 mL/min, λ = 254 nm), t_1 = 4.88 min (major), t_2 = 5.81 min (minor), *ee* = 92%; ^{1}H NMR (400 MHz, d_6-DMSO) δ ppm: 8.4 – 8.46 (m, 2H), 7.51 (m, 1H), 7.40 (d, *J* = 8.5 Hz, 1H), 7.33 – 7.27 (m, 1H), 7.15 (d, *J* = 4.1 Hz, 3H), 6.79 – 6.72 (m, 1H), 2.07 (s, 3H), 1.59 (s, 3H), 1.45 (s, 9H), 1.42 (s, 9H); ^{13}C NMR (100 MHz, d_6-DMSO) δ ppm: 186.8, 154.7, 153.7, 153.6, 147.3, 135.7, 130.8, 129.4, 128.2, 125.9, 124.8, 122.9, 122.1, 121.7, 120.1, 80.0, 79.9, 61.4, 28.5, 20.6, 16.0; HRMS (ESI) Calcd. For $C_{26}H_{34}N_3O_4^+$ $[M+H]^+$ 452.2544, found 452.2550.

N,*N*'-(4-((3a*S*,8a*R*)-2,3,8,8a-tetrahydro-3a*H*-furo[2,3-*b*]indol-3a-yl)-1,2-phenylene)dibenzamide **4a:**

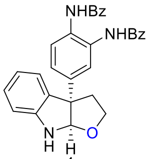

4a

White solid; 46.1 mg; isolated yield = 97%; M.p.: 192 – 193 °C; $[\alpha]_D^{20}$ = +236 (*c* 0.25, MeOH); HPLC (Chiralpak IF-3, *i*-propanol/hexane = 20/80, flow rate 1.0 mL/min, λ = 254 nm), t_1 = 35.78 min (minor), t_2 = 47.95 min (major), *ee* = 96%; ^{1}H NMR (400 MHz, $CDCl_3$) δ ppm: 9.16 (s, 1H), 9.11 (s, 1H), 8.02 – 7.88 (m, 4H), 7.58 – 7.39 (m, 8H), 7.08 – 7.00 (m, 2H), 6.84 (d, *J* = 7.1 Hz, 1H), 6.71 – 6.66 (m, 1H), 6.61 (d, *J* = 7.8 Hz, 1H), 5.40 (s, 1H), 4.54 (s, 1H), 4.08 – 3.97 (m, 1H), 3.61 – 3.49 (m, 1H), 2.63 – 2.47 (m, 1H), 2.38 – 2.27 (m, 1H); ^{13}C NMR (100 MHz, $CDCl_3$) δ ppm: 161.8, 161.5, 144.5, 137.5, 129.0, 128.8, 127.5, 127.4, 125.9, 124.8, 124.1, 124.0, 123.7, 122.9, 122.8, 121.4, 119.8, 118.2, 114.6, 104.0, 95.4, 63.4, 57.0, 35.3; HRMS (ESI) Calcd. For $C_{30}H_{25}N_3O_3Na^+$ $[M+Na]^+$ 498.1788, found 498.1790.

N,*N*'-(4-((3a*S*,8a*R*)-5-methoxy-2,3,8,8a-tetrahydro-3a*H*-furo[2,3-*b*]indol-3a-yl)-1,2-phenylene)dibenzamide **4b:**

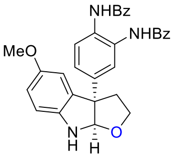

4b

White solid; 44.4 mg; isolated yield = 88%; M.p.: 129 – 130 °C; $[\alpha]_D^{20}$ = +91 (*c* 0.20, MeOH); HPLC (Chiralpak IK-3, *i*-propanol/hexane = 40/60, flow rate 1.0 mL/min, λ = 254 nm), t_1 = 16.15 min (major), t_2 = 28.09 min (minor), *ee* = 98%; ^{1}H NMR (400 MHz, $CDCl_3$) δ ppm: 9.42 – 9.02 (m, 2H), 8.07 – 7.84 (m, 4H), 7.63 – 7.40 (m, 7H), 7.36 (d, *J* = 1.8 Hz, 1H), 7.03 – 6.95 (m, 1H), 6.66 – 6.60 (m, 1H), 6.55 (d, *J* = 8.5 Hz, 1H), 6.48 (d, *J* = 2.5 Hz, 1H), 5.28 (s, 1H), 4.01 – 3.92 (m, 1H), 3.67 (s, 3H), 3.60 – 3.48 (m, 1H), 2.51 – 2.39 (m, 1H), 2.35 – 2.23 (m, 1H); ^{13}C NMR (100 MHz, $CDCl_3$) δ ppm: 166.6, 166.3, 153.9, 143.2, 141.9, 133.9, 133.7, 133.5, 132.3, 132.2, 130.6, 129.7, 128.8, 127.7, 127.5, 126.2, 124.6, 123.0, 113.7, 110.9, 109.8, 100.9, 68.1, 62.1, 55.9, 39.6; HRMS (ESI) Calcd. For $C_{31}H_{28}N_3O_4^+$ $[M+H]^+$ 506.2074, found 506.2076.

N,N'-(4-((3a*S*,8a*R*)-5-chloro-2,3,8,8a-tetrahydro-3a*H*-furo[2,3-*b*]indol-3a-yl)-1,2-phenylene)dibenzamide **4c:**

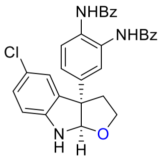

4c

White solid; 41.7 mg; isolated yield = 82%; M.p.: 180 – 181 °C; $[\alpha]_D^{20}$ = +22 (*c* 0.17, MeOH); HPLC (Chiralpak IG-3, *i*-propanol/hexane = 30/70, flow rate 1.0 mL/min, λ = 254 nm), t_1 = 14.96 min (minor), t_2 = 19.08 min (major), *ee* = 97%; ^{1}H NMR (400 MHz, d_6-DMSO) δ ppm: 10.16 (s, 1H), 10.00 (s, 1H), 7.99 – 7.88 (m, 4H), 7.68 – 7.48 (m, 8H), 7.33 – 7.21 (m, 1H), 7.11 – 6.85 (m, 3H), 6.57 (d, *J* = 8.3 Hz, 1H), 5.71 – 5.53 (m, 1H), 4.25 – 3.96 (m, 1H), 3.51 – 3.43 (m, 1H), 2.68 – 2.53 (m, 2H); ^{13}C NMR (100 MHz, d_6-DMSO) δ ppm: 166.1, 165.8, 149.4, 141.9, 135.3, 134.6, 134.5, 132.4, 131.7, 130.5, 129.1, 129.0, 128.4, 128.1, 127.9, 126.5, 124.5, 123.6, 123.5, 121.2, 109.2, 100.0, 67.5, 61.8, 40.0; HRMS (ESI) Calcd. For $C_{30}H_{25}ClN_3O_3^+$ $[M+H]^+$ 510.1579, found 510.1584.

N,N'-(4-((3a*S*,8a*R*)-5-bromo-2,3,8,8a-tetrahydro-3a*H*-furo[2,3-*b*]indol-3a-yl)-1,2-phenylene)dibenzamide **4d:**

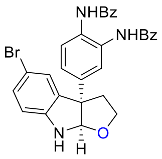

4d

White solid; 48.1 mg; isolated yield = 87%; M.p.: 115 – 116 °C; $[\alpha]_D^{20}$ = +56 (*c* 0.27, MeOH); HPLC (Chiralpak IG-3, *i*-propanol/hexane = 30/70, flow rate 1.0 mL/min, λ = 254 nm), t_1 = 15.48 min (minor), t_2 = 19.22 min (major), *ee* = 94%; ^{1}H NMR (400 MHz, d_6-DMSO) δ ppm: 10.15 (m, 1H), 9.99 (m, 1H), 8.00 – 7.85 (m, 4H), 7.67 – 7.48 (m, 8H), 7.30 – 7.23 (m, 1H), 7.19 – 6.84 (m, 3H), 6.63 – 6.52 (m, 1H), 5.64 – 5.60 (m, 1H), 4.13 – 3.99 (m, 1H), 3.50 – 3.44 (m, 1H), 2.68 – 2.52 (m, 2H); ^{13}C NMR (100 MHz, d_6-DMSO) δ ppm: 166.1, 165.8, 149.7, 141.9, 135.9, 134.6, 134.5, 132.9, 132.4, 132.4, 131.7, 131.6, 131.2, 130.5, 130.2, 129.1, 129.1, 128.6, 128.0, 127.9, 127.2, 126.5,

126.4, 124.6, 123.8, 123.6, 123.5, 118.1, 109.9, 108.5, 108.2, 100.0, 67.5, 61.7, 39.5;HRMS (ESI) Calcd. For $C_{30}H_{25}BrN_3O_3^+$ $[M+H]^+$ 554.1074, found 554.1073.

N,N'-(4-((3a*S*,8a*R*)-6-fluoro-2,3,8,8a-tetrahydro-3a*H*-furo[2,3-*b*]indol-3a-yl)-1,2-phenylene)dibenzamide **4f:**

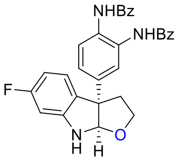

4f

White solid; 37.5 mg; isolated yield = 76%; M.p.: 178 – 179 °C; $[\alpha]_D^{20}$ = +216 (*c* 0.33, MeOH); HPLC (Chiralpak IB-3, *i*-propanol/hexane = 30/70, flow rate 1.0 mL/min, λ = 254 nm), t_1 = 14.37 min (minor), t_2 = 16.94 min (major), *ee* = 99%; ^{1}H NMR (400 MHz, $CDCl_3$) δ ppm: 9.35 (s, 1H), 9.24 (s, 1H), 8.13 – 7.89 (m, 4H), 7.60 – 7.31 (m, 8H), 6.88 – 6.77 (m, 2H), 6.60 – 6.53 (m, 1H), 6.47 (d, *J* = 7.3 Hz, 1H), 5.28 (s, 1H), 4.52 (s, 1H), 4.03 – 3.85 (m, 1H), 3.63 – 3.44 (m, 1H), 2.43 – 2.29 (m, 1H), 2.25 – 2.14 (m, 1H); ^{13}C NMR (100 MHz, $CDCl_3$) δ ppm: 166.6, 166.4, 147.7 (d, *J* = 240 Hz), 141.6, 136.5, 136.4, 135.7, 133.5, 133.3, 132.3, 130.7, 129.7, 128.8 (d, *J* = 4 Hz), 127.8, 127.6, 126.3, 124.5, 122.8, 119.8, 114.9 (d, *J* = 17 HZ), 100.5, 68.2, 62.3, 62.2, 39.5; ^{19}F NMR (376 MHz, $CDCl_3$) δ -135.60; HRMS (ESI) Calcd. For $C_{30}H_{25}FN_3O_3^+$ $[M+H]^+$ 494.1874, found 494.1884.

N,N'-(4-((3a*S*,8a*R*)-7-methyl-2,3,8,8a-tetrahydro-3a*H*-furo[2,3-*b*]indol-3a-yl)-1,2-phenylene)dibenzamide **4g:**

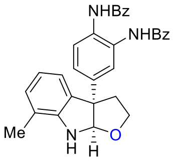

4g

White solid; 45.5 mg; isolated yield = 93%; M.p.: 161 – 162 °C; $[\alpha]_D^{20}$ = +41 (*c* 0.30, MeOH); HPLC (Chiralpak IF-3, *i*-propanol/hexane = 30/70, flow rate 1.0 mL/min, λ = 254 nm), t_1 = 20.37 min (minor), t_2 = 23.90 min (major), *ee* = 98%; ^{1}H NMR (400 MHz, $CDCl_3$) δ ppm: 9.42 (s, 1H), 9.31 (s, 1H), 8.03 (d, *J* = 7.3 Hz, 2H), 7.94 (d, *J* = 7.4 Hz, 2H), 7.59 – 7.37 (m, 7H), 7.32 (d, *J* = 1.5 Hz, 1H), 6.92 – 6.80 (m, 2H), 6.62 – 6.49 (m, 2H), 5.23 (s, 1H), 4.29 (s, 1H), 3.93 – 3.83 (m, 1H), 3.55 – 3.41 (m, 1H), 2.38 – 2.24 (m, 1H), 2.18 – 2.06 (m, 4H); ^{13}C NMR (100 MHz, $CDCl_3$) δ ppm: 166.7, 166.4, 147.9, 142.4, 133.6, 133.4, 132.3, 132.1, 131.5, 130.6, 129.5, 129.3, 128.8, 127.8, 127.6, 126.2, 124.7, 122.8, 121.9, 119.5, 118.1, 100.0, 68.1, 61.9, 39.9, 16.8; HRMS (ESI) Calcd. For $C_{31}H_{28}N_3O_3^+$ $[M+H]^+$ 490.2125, found 490.2137.

N,N'-(4-((3a*S*,8a*R*)-7-fluoro-2,3,8,8a-tetrahydro-3a*H*-furo[2,3-*b*]indol-3a-yl)-1,2-phenylene)dibenzamide **4h:**

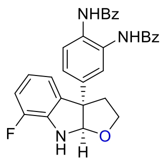

4h

White solid; 41.9 mg; isolated yield = 85%; M.p.: 185 – 186 °C; $[\alpha]_D^{20}$ = +109 (*c* 0.25, MeOH); HPLC (Chiralpak IF-3, *i*-propanol/hexane = 20/80, flow rate 1.0 mL/min, λ = 254 nm), t_1 = 37.08 min (minor), t_2 = 42.64 min (major), *ee* = 91%; ^{1}H NMR (400 MHz, $CDCl_3$) δ ppm: 9.42 (s, 1H), 9.26 (s, 1H), 8.10 – 7.90 (m, 4H), 7.58 – 7.31 (m, 8H), 6.88 – 6.79 (m, 1H), 6.53 – 6.44 (m, 1H), 6.31 – 6.18 (m, 2H), 5.22 (s, 1H), 4.54 (s, 1H), 3.97 – 3.86 (m, 1H), 3.56 – 3.42 (m, 1H), 2.43 – 2.24 (m, 1H), 2.14 – 2.04 (m, 1H); ^{13}C NMR (100 MHz, $CDCl_3$) δ ppm: 166.6, 166.4, 163.6 (d, *J* = 242 HZ), 150.7, 142.1, 133.4, 132.3, 130.7, 129.6, 128.8 (d, *J* = 4 Hz), 127.7, 127.5, 126.3, 125.1, 125.0, 124.6, 122.8, 105.5 (d, *J* = 23 HZ), 100.7, 96.4, 96.2, 68.1, 60.9, 39.8; ^{19}F NMR (376 MHz, $CDCl_3$) δ ppm: -114.27; HRMS (ESI) Calcd. For $C_{30}H_{25}FN_3O_3^+$ $[M+H]^+$ 494.1874, found 494.1884.

N,*N'*-(4-((3a*S*,8a*R*)-1-tosyl-2,3,8,8a-tetrahydropyrrolo[2,3-*b*]indol-3a(1*H*)-yl)-1,2-phenylene)dibenzamide **4i:**

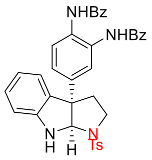

4i

White solid; 59.7 mg; isolated yield = 95%; M.p.: 125 – 126 °C; $[\alpha]_D^{20}$ = +229 (*c* 0.25, MeOH); HPLC (Chiralpak IF-3, *i*-propanol/hexane = 30/70, flow rate 1.0 mL/min, λ = 254 nm), t_1 = 72.76 min (major), t_2 = 89.04 min (minor), *ee* = >99%; ^{1}H NMR (400 MHz, $CDCl_3$) δ ppm: 9.25 – 9.23 (m, 2H), 8.00 – 7.86 (m, 4H), 7.67 – 7.65 (d, *J* = 8.2 Hz, 2H), 7.56 – 7.35 (m, 7H), 7.25 – 7.21 (m, 3H), 7.07 – 7.00 (m, 1H), 6.83 – 6.76 (m, 1H), 6.71 – 6.56 (m, 3H), 5.27 (s, 1H), 4.85 (s, 1H), 3.52 – 3.37 (m, 1H), 3.19 – 3.01 (m, 1H), 2.33 (s, 3H), 2.22 – 2.15 (m, 1H), 2.11 – 2.02 (m, 1H); ^{13}C NMR (100 MHz, $CDCl_3$) δ ppm: 166.6, 166.3, 148.6, 143.8, 141.2, 136.2, 133.6, 133.4, 132.4, 132.2, 131.2, 130.5, 130.0, 129.9, 128.9, 128.8, 128.8, 127.7, 127.5, 127.0, 126.1, 124.1, 123.8, 122.5, 119.8, 110.2, 85.4, 61.2, 48.0, 36.6, 21.5; HRMS (ESI) Calcd. For $C_{37}H_{32}N_4O_4SNa^+$ $[M+Na]^+$ 651.2036, found 651.2040.

N,*N'*-(4-((3a*S*,8a*R*)-1-tosyl-2,3,8,8a-tetrahydropyrrolo[2,3-*b*]indol-3a(1*H*)-yl)-1,2-phenylene)bis(3-methylbenzamide) **4j:**

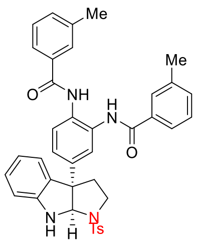

4j

White solid; 58.4 mg; isolated yield = 89%; M.p.: 83 – 84 °C; $[\alpha]_D^{20}$ = +203 (*c* 0.50, MeOH); HPLC (Chiralpak IF-3, *i*-propanol/hexane = 30/70, flow rate 1.0 mL/min, λ = 254 nm), t_1 = 63.39 min (major), t_2 = 69.94 min (minor), *ee* = 93%; ^{1}H NMR (400 MHz, d_6-DMSO) δ ppm: 10.05 (s, 1H), 9.90 (s, 1H), 7.81 – 7.66 (m, 6H), 7.54 (d, *J* = 8.5 Hz, 1H), 7.45 – 7.32 (m, 7H), 7.08 – 6.96 (m, 3H), 6.75 (s, 1H), 6.68 – 6.59 (m, 2H), 5.54 (s, 1H), 3.71 – 3.62 (m, 1H), 3.46 (s, 1H), 3.01 – 2.90 (m, 1H), 2.37 (s, 3H), 2.34 (s, 3H), 2.32 (s, 3H), 2.06 (m, 1H); ^{13}C NMR (100 MHz, d_6- DMSO) δ ppm: 166.1, 165.9, 149.8, 143.9, 141.5, 138.4, 136.4, 134.6, 134.4, 133.0, 131.7, 131.5, 130.6, 130.4, 129.0, 128.9, 128.6, 128.4, 127.5, 126.1, 125.2, 125.0, 124.2, 123.3, 118.7, 109.7, 85.8, 61.7, 48.4, 37.7, 21.4; HRMS (ESI) Calcd. For $C_{39}H_{37}N_4O_4S^+$ $[M+H]^+$ 657.2530, found 657.2539.

N,*N'*-(4-((3a*S*,8a*R*)-1-tosyl-2,3,8,8a-tetrahydropyrrolo[2,3-*b*]indol-3a(1*H*)-yl)-1,2-phenylene)bis(3-fluorobenzamide) **4k:**

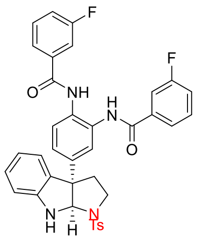

4k

Yellow oil; 53.8 mg; isolated yield = 81%; $[\alpha]_D^{20}$ = +164 (*c* 0.39, MeOH); HPLC (Chiralpak IK-3, *i*-propanol/hexane = 30/70, flow rate 1.0 mL/min, λ = 254 nm), t_1 = 38.94 min (minor), t_2 = 46.15 min (major), *ee* = 98%; ^{1}H NMR (400 MHz, d_6-DMSO) δ ppm: 10.09 (s, 1H), 9.98 (s, 1H), 7.82 – 7.66 (m, 6H), 7.62 – 7.51 (m, 3H), 7.48 – 7.34 (m, 5H), 7.08 – 6.97 (m, 3H), 6.76 (s, 1H), 6.70 – 6.60 (m, 2H), 5.54 (s, 1H), 3.73 – 3.61 (m, 1H), 3.44 (s, 1H), 2.99 – 2.90 (m, 1H), 2.32 (s, 3H), 2.13 – 2.00 (m, 1H); ^{13}C NMR (100 MHz, d_6-DMSO) δ ppm: 164.7, 164.6, 162.4 (d, *J* = 244 Hz), 149.8, 143.9, 141.7, 137.2, 137.1, 137.0, 136.4, 131.7, 131.6, 131.2 (d, *J* = 8 Hz), 130.6, 130.3, 128.9, 127.5, 126.4, 124.3, 124.2, 124.1, 123.6, 123.4, 119.3, 119.2, 119.1, 118.7, 115.1 (d, *J* = 23 Hz), 115.0 (d, *J* = 24 Hz), 109.7, 85.8, 61.7, 48.3, 37.7, 21.4; ^{19}F NMR (376 MHz, d_6-DMSO) δ ppm: -112.50, -112.52; HRMS (ESI) Calcd. For $C_{37}H_{31}F_2N_4O_4S^+$ $[M+H]^+$ 665.2029, found 665.2032.

N,*N'*-(4-((3a*S*,8a*R*)-1-tosyl-2,3,8,8a-tetrahydropyrrolo[2,3-*b*]indol-3a(1*H*)-yl)-1,2-phenylene)bis(4-methylbenzamide) **4l:**

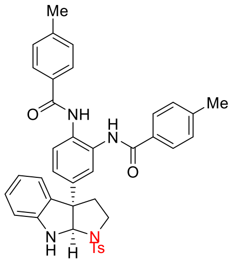

4l

White solid; 56.4 mg; isolated yield = 86%; M.p.: 237 – 238 °C; $[\alpha]_D^{20}$ = +169 (*c* 0.20, MeOH); HPLC (Chiralpak IF-3, *i*-propanol/hexane = 30/70, flow rate 1.0 mL/min, λ = 254 nm), t_1 = 87.45 min (major), t_2 = 101.29 min (minor), *ee* = 96%; ^{1}H NMR (400 MHz, d_6-DMSO) δ ppm: 10.05 (s, 1H), 9.90 (s, 1H), 7.92 – 7.75 (m, 6H), 7.53 (d, *J* = 8.5 Hz, 1H), 7.40 (d, *J* = 1.6 Hz, 1H), 7.38 – 7.28 (m, 6H), 7.11 – 6.95 (m, 3H), 6.75 (s, 1H), 6.70 – 6.60 (m, 2H), 5.54 (s, 1H), 3.70 – 3.58 (m, 1H), 3.51 – 3.42 (m, 1H), 3.02 – 2.87 (m, 1H), 2.37 (s, 3H), 2.35 (s, 3H), 2.32 (s, 3H), 2.14 – 1.98 (m, 1H); ^{13}C NMR (100 MHz, DMSO) δ ppm: 165.9, 165.6, 149.8, 143.9, 142.6, 142.5, 141.4, 136.4, 131.7, 131.5, 130.6, 130.3, 129.6, 128.9, 128.0, 127.9, 127.5, 126.2, 124.2, 123.3, 123.2, 118.7, 109.7, 85.8, 61.7, 48.4, 37.7, 21.5, 21.4; HRMS (ESI) Calcd. For $C_{39}H_{37}N_4O_4S^+$ $[M+H]^+$ 657.2530, found 657.2540.

N,*N'*-(4-((3a*S*,8a*R*)-1-tosyl-2,3,8,8a-tetrahydropyrrolo[2,3-*b*]indol-3a(1*H*)-yl)-1,2-phenylene)bis(4-chlorobenzamide) **4m:**

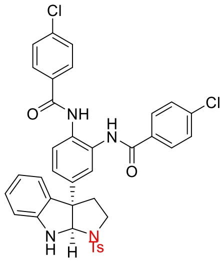

4m

White solid; 62.6 mg; isolated yield = 90%; M.p.: 77 – 78 °C; $[\alpha]_D^{20}$ = +187 (*c* 0.25, MeOH); HPLC (Chiralpak IK-3, *i*-propanol/hexane = 30/70, flow rate 1.0 mL/min, λ = 254 nm), t_1 = 41.70 min (major), t_2 = 49.66 min (minor), *ee* = 95%; ^{1}H NMR (400 MHz, d_6-DMSO) δ ppm: 10.11 (s, 1H), 9.99 (s, 1H), 8.01 – 7.88 (m, 4H), 7.78 (d, *J* = 8.2 Hz, 2H), 7.63 – 7.51 (m, 5H), 7.42 – 7.33 (m, 3H), 7.08 – 6.97 (m, 3H), 6.76 (s, 1H), 6.70 – 6.59 (m, 2H), 5.54 (s, 1H), 3.74 – 3.60 (m, 1H), 3.45 (s, 1H), 3.03 – 2.90 (m, 1H), 2.32 (s, 3H), 2.15 – 1.98 (m, 1H); ^{13}C NMR (100 MHz, d_6-DMSO) δ ppm: 165.0, 164.8, 149.8, 143.9, 141.6, 137.2, 137.1, 136.4, 133.5, 133.4, 131.7, 131.5, 130.6, 130.3, 130.1, 129.9, 129.1, 128.9, 127.5, 126.4, 124.2, 123.6, 123.4, 118.7, 109.7, 85.8, 61.7, 48.3, 37.7, 21.4; HRMS (ESI) Calcd. For $C_{37}H_{31}Cl_2N_4O_4S^+$ $[M+H]^+$ 697.1438, found 697.1447.

((5a*R*,10b*R*)-10b-methyl-6,10b-dihydro-5*H*-indolo[2,3-*b*]quinoxaline-5,11(5a*H*)-diyl) bis(phenylmethanone) **5a**:

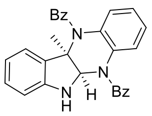

5a

White solid; 24.5 mg; isolated yield: 55%; M.p.: 164 – 165 °C; $[\alpha]_D^{20}$ = -73 (*c* 0.1, MeOH); HPLC (Chiralpak IB-3, *i*-propanol/hexane = 30/70, flow rate 1.0 mL/min, λ = 254 nm), t_1= 6.84 min (minor), t_2 = 9.14 min (major), *ee* = 92%; ^{1}H NMR (400 MHz, d_6-DMSO) δ ppm: 7.68 – 7.51 (m, 10H), 7.48 – 7.40 (m, 3H), 6.97 – 6.93 (m, 1H), 6.82 – 6.79 (m, 1H), 6.67 – 6.60 (m, 2H), 6.40 – 6.32 (m, 2H), 6.17 (s, 1H), 2.09 (s, 3H); ^{13}C NMR (100 MHz, d_6-DMSO) δ ppm: 169.8, 168.8, 149.5, 136.9, 135.9, 133.7, 133.5, 132.4, 132.2, 132.0, 131.6, 131.0, 130.6, 130.4, 130.0, 129.8, 126.3, 119.9, 119.6, 118.8, 109.6, 81.6, 74.1, 26.0; HRMS (ESI) Calcd. For $C_{29}H_{24}N_3O_2^+$ $[M+H]^+$ 446.1863, found 446.1872.

((5a*R*,10b*R*)-2,3-dibromo-10b-methyl-6,10b-dihydro-5*H*-indolo[2,3-*b*]quinoxaline-5, 11(5a*H*)-diyl)bis(phenylmethanone) **5b**:

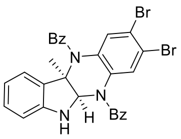

5b

White solid; 57.2 mg; isolated yield: 95%; M.p.: 158 – 159 °C; $[\alpha]_D^{20}$ = -86 (*c* 0.1, MeOH); HPLC (Chiralpak IB, *i*-propanol/hexane = 30/70, flow rate 1.0 mL/min, λ = 254 nm), t_1= 12.22 min (major), t_2 = 18.35 min (minor), *ee* = 94%; ^{1}H NMR (400 MHz, d_6-DMSO) δ ppm: 7.70 – 7.67 (m, 3H), 7.61 – 7.41 (m, 8H), 7.17 – 6.96 (m, 2H), 6.76 (s, 1H), 6.70 – 6.63 (m, 2H), 6.44 – 6.37 (m, 1H), 6.20 (s, 1H), 2.11 (s, 3H); ^{13}C NMR (100 MHz, d_6-DMSO) δ ppm: 170.8, 169.6, 149.5, 137.3, 136.8, 134.5, 133.7, 132.0, 131.9, 130.8, 130.6, 130.0, 129.9, 129.6, 129.2, 129.0, 128.8, 128.2, 126.3, 119.6, 119.2, 118.7, 109.5, 81.7, 73.9, 26.0; HRMS (ESI) Calcd. For $C_{29}H_{22}Br_2N_3O_2^+$ $[M+H]^+$ 602.0073, found 602.0077.

((5a*R*,10b*R*)-2,3-dibromo-10,10b-dimethyl-6,10b-dihydro-5*H*-indolo[2,3-*b*]quinoxaline-5,11(5a*H*)-diyl)bis(phenylmethanone) **5c**:

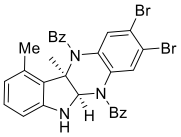

5c

White solid; 56.7 mg; isolated yield: 92%; M.p.: 126 – 127 °C; $[\alpha]_D^{20}$ = -75 (*c* 0.1, MeOH); HPLC (Chiralpak IB, *i*-propanol/hexane = 30/70, flow rate 1.0 mL/min, λ = 254 nm), t_1= 8.19 min (major), t_2 = 12.90 min (minor), *ee* = 93%; ^{1}H NMR (400 MHz, d_6-DMSO) δ ppm: 7.59 – 7.40 (m, 11H), 6.88 (s, 1H), 6.80 – 6.75 (m, 2H), 6.41 – 6.23 (m, 2H), 5.80 (s, 1H), 2.37 (s, 3H), 2.12 (s, 3H); ^{13}C NMR (100 MHz, d_6-DMSO) δ ppm: 170.1, 169.5, 166.2, 149.3, 137.6, 135.3, 134.6, 134.3, 134.0, 131.7, 131.4, 131.1, 129.6, 129.1, 129.0, 129.0, 128.7, 128.2, 126.8, 120.8, 120.0, 107.0, 84.3, 74.5, 25.2, 18.1; HRMS (ESI) Calcd. For $C_{30}H_{24}Br_2N_3O_2^+$ $[M+H]^+$ 616.0230, found 616.0235.

((5a*R*,10b*R*)-2,3-dibromo-9,10b-dimethyl-6,10b-dihydro-5*H*-indolo[2,3-*b*]quinoxaline-5,11(5a*H*)-diyl)bis(phenylmethanone) **5d**:

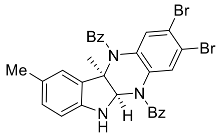

5d

White solid; 58.5 mg; isolated yield: 95%; M.p.: 133 – 134 °C; $[\alpha]_D^{20}$ = -58 (*c* 0.1, MeOH); HPLC (Chiralpak IB, *i*-propanol/hexane = 30/70, flow rate 1.0 mL/min, λ = 254 nm), t_1= 9.62 min (major), t_2 = 16.89 min (minor), *ee* = 91%; ^{1}H NMR (400 MHz, d_6-DMSO) δ ppm: 7.70 – 7.60 (m, 2H), 7.58 – 7.41 (m, 10H), 6.81 – 6.78 (m, 2H), 6.48 (s, 1H), 6.35 – 6.33 (m, 1H), 6.18 (s, 1H), 2.18 (s, 3H), 2.08 (s, 3H); ^{13}C NMR (100 MHz, d_6-DMSO) δ ppm: 170.8, 169.6, 147.2, 137.3, 136.9, 134.6, 133.7, 131.9, 130.8, 130.5, 130.5, 130.0, 129.6, 129.2, 129.0, 128.8, 128.2, 127.3, 126.8, 119.6, 119.1, 109.6, 82.0, 74.0, 25.9, 21.1; HRMS (ESI) Calcd. For $C_{30}H_{24}Br_2N_3O_2^+$ $[M+H]^+$ 616.0230, found 616.0233.

((5a*R*,10b*R*)-2,3-dibromo-9-methoxy-10b-methyl-6,10b-dihydro-5*H*-indolo[2,3-*b*]quinoxaline-5,11(5a*H*)-diyl)bis(phenylmethanone) **5e**:

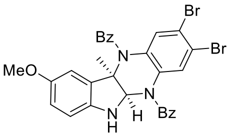

5e

White solid; 58.8 mg; isolated yield: 93%; M.p.: 162 – 163 °C; $[\alpha]_D^{20}$ = -71 (*c* 0.1, MeOH); HPLC (Chiralpak IB, *i*-propanol/hexane = 30/70, flow rate 1.0 mL/min, λ = 254 nm), t_1= 6.40 min (major), t_2 = 7.91 min (minor), *ee* = 92%; ^{1}H NMR (400 MHz, d_6-DMSO) δ ppm: 7.70 – 7.62 (m, 2H), 7.58 – 7.22 (m, 6H), 716-7.12 (m, 3H), 6.81 (s, 1H), 6.61 – 6.44 (m, 1H), 6.38 – 6.32 (m, 1H), 6.31 – 6.28 (m, 2H), 6.22 (s, 1H), 3.66 (s, 3H), 2.10 (s, 3H); ^{13}C NMR (100 MHz, d_6-DMSO) δ ppm: 170.6, 169.4, 162.4, 149.8, 137.8, 134.4, 131.9, 131.3, 130.7, 130.6, 130.2, 130.0, 128.8, 126.5, 126.3, 119.5, 119.0, 118.7, 114.7, 114.6, 109.5, 82.1, 74.0, 56.1, 26.5; HRMS (ESI) Calcd. For $C_{30}H_{24}Br_2N_3O_3^+$ $[M+H]^+$ 632.0179, found 632.0182.

((5a*R*,10b*R*)-2,3-dibromo-9-fluoro-10b-methyl-6,10b-dihydro-5*H*-indolo[2,3-*b*]quinoxaline-5,11(5a*H*)-diyl)bis(phenylmethanone) **5f**:

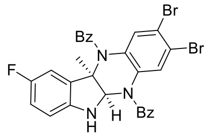

5f

White solid; 55.8 mg; yield: 90%; M.p.: 156 – 157 °C; $[\alpha]_D^{20}$ = -68 (*c* 0.1, MeOH); HPLC (Chiralpak IA, *i*-propanol/hexane = 30/70, flow rate 1.0 mL/min, λ = 254 nm), t_1= 3.84 min (minor), t_2 = 5.87 min (major), *ee* = 95%; ^{1}H NMR (400 MHz, d_6-DMSO) δ ppm: 7.67 – 7.63 (m, 11H), 7.01 – 6.82 (m, 3H), 6.59 – 6.58 (m, 1H), 6.42 – 6.39 (m, 1H), 6.22 (s, 1H), 2.09 (s, 3H); ^{13}C NMR (100 MHz, d_6-DMSO) δ ppm: 170.7, 169.4,

155.7 (d, J = 232 Hz), 145.6, 136.4, 134.2, 133.3, 131.8 (d, J = 8 Hz), 130.9, 130.6, 130.3, 129.4, 129.0, 128.8, 128.6, 119.4, 119.0, 116.3, 116.1, 113.2 (d, J = 24 Hz), 110.1, 110.0, 80.2, 73.5, 25.3; ^{19}F NMR (376 MHz, DMSO) δ ppm: -125.80; HRMS (ESI) Calcd. For $C_{29}H_{21}Br_2FN_3O_2^+$ $[M+H]^+$ 619.9979, found 619.9979.

((5a*R*,10b*R*)-2,3-dibromo-9-chloro-10b-methyl-6,10b-dihydro-5*H*-indolo[2,3-*b*]quinoxaline-5,11(5a*H*)-diyl)bis(phenylmethanone) **5g**:

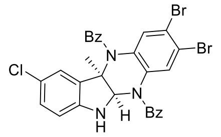

5g

White solid; 61.1 mg; isolated yield: 96%; M.p.: 141 – 142 °C; $[\alpha]_D^{20}$ = -56 (c 0.1, MeOH); HPLC (Chiralpak IB, i-propanol/hexane = 30/70, flow rate 1.0 mL/min, λ = 254 nm), t_1= 7.30 min (major), t_2 = 8.69 min (minor), ee = 94%; ^{1}H NMR (400 MHz, d_6-DMSO) δ ppm: 7.67 – 7.64 (m, 3H), 7.61 – 7.43 (m, 9H), 7.04 – 7.02 (m, 1H), 6.87 – 6.84 (m, 2H), 6.44 – 6.42 (m, 1H), 6.23 (s, 1H), 2.09 (s, 3H); ^{13}C NMR (100 MHz, d_6-DMSO) δ ppm: 171.0, 169.6, 148.5, 136.6, 134.4, 133.4, 132.1, 131.8, 130.8, 130.5, 129.8, 129.7, 129.2, 129.0, 128.8, 128.2, 126.2, 121.6, 119.7, 119.3, 81.0, 73.5, 25.6; HRMS (ESI) Calcd. For $C_{29}H_{21}Br_2ClN_3O_2^+$ $[M+H]^+$ 635.9684, found 635.9684.

((5a*R*,10b*R*)-2,3,9-tribromo-10b-methyl-6,10b-dihydro-5*H*-indolo[2,3-*b*]quinoxaline-5,11(5a*H*)-diyl)bis(phenylmethanone) **5h:**

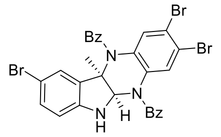

5h

White solid; 58.8 mg; isolated yield: 93%; M.p.: 162 – 163 °C; $[\alpha]_D^{20}$ = -65 (c 0.1, MeOH); HPLC (Chiralpak IB, i-propanol/hexane = 30/70, flow rate 1.0 mL/min, λ = 254 nm), t_1= 6.94 min (major), t_2 = 8.06 min (minor), ee = 90%; ^{1}H NMR (400 MHz, d_6-DMSO) δ ppm: 7.48 – 7.47 (m, 2H), 7.44 – 7.34 (m, 6H), 7.32-7.25 (m, 3H), 6.98 – 6.67 (m, 2H), 6.59 (s, 1H), 6.48 – 6.46 (m, 1H), 6.23 – 6.22 (m, 1H), 6.03 (s, 1H), 1.91 (s, 3H); ^{13}C NMR (100 MHz, d_6-DMSO) δ ppm: 170.9, 169.7, 149.6, 137.4, 136.9, 134.6, 133.7, 132.0, 130.9, 130.6, 130.0, 130.0, 129.6, 129.3, 129.1, 128.9, 128.3, 126.4, 119.7, 119.3, 118.8, 109.6, 81.8, 74.0, 26.1; HRMS (ESI) Calcd. For $C_{29}H_{21}Br_2N_3O_2^+$ $[M+H]^+$ 679.9178, found 679.9181.

((5a*R*,10b*R*)-2,3-dibromo-8,10b-dimethyl-6,10b-dihydro-5*H*-indolo[2,3-*b*]quinoxaline-5,11(5a*H*)-diyl)bis(phenylmethanone) **5i**:

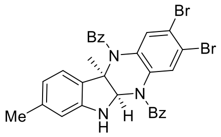

5i

White solid; 58.5 mg; isolated yield: 95%; M.p.: 155 – 156 °C; $[\alpha]_D^{20}$ = -67 (c 0.1, MeOH); HPLC (Chiralpak IB, i-propanol/hexane = 30/70, flow rate 1.0 mL/min, λ =

254 nm), t_1= 6.97 min (minor), t_2 = 7.78 min (major), *ee* = 90%; ^{1}H NMR (400 MHz, d_6-DMSO) δ ppm: 7.70 – 7.60 (m, 2H), 7.58 – 7.41 (m, 10H), 6.81 – 6.78 (m, 2H), 6.48 (s, 1H), 6.36 – 6.34 (m, 1H), 6.19 (s, 1H), 2.18 (s, 3H), 2.08 (s, 3H); ^{13}C NMR (100 MHz, d_6-DMSO) δ ppm: 170.8, 169.7, 147.3, 137.4, 136.9, 134.6, 133.7, 131.9, 130.8, 130.5, 130.5, 130.0, 129.6, 129.2, 129.0, 128.8, 128.2, 127.3, 126.8, 119.6, 119.1, 109.6, 82.0, 74.0, 26.0, 21.2; HRMS (ESI) Calcd. For $C_{30}H_{24}Br_2N_3O_2^+$ [M+H]$^+$ 616.0230, found 616.0231.

((5a*R*,10b*R*)-2,3-dibromo-8-methoxy-10b-methyl-6,10b-dihydro-5*H*-indolo[2,3-*b*]quinoxaline-5,11(5a*H*)-diyl)bis(phenylmethanone) **5j**:

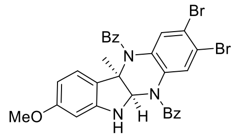

5j

White solid; 58.1 mg; isolated yield: 92%; M.p.: 121 – 122 °C; $[\alpha]_D^{20}$ = -47 (*c* 0.1, MeOH); HPLC (Chiralpak IC-3, *i*-propanol/hexane = 30/70, flow rate 1.0 mL/min, λ = 254 nm), t_1= 12.03 min (minor), t_2 = 15.36 min (minor), *ee* = 92%; ^{1}H NMR (400 MHz, d_6-DMSO) δ ppm: 7.70 – 7.62 (m, 2H), 7.58 – 7.22 (m, 6H), 7.16 – 6,81 (m, 4H), 6.61 – 6.44 (m, 1H), 6.38 – 6.32 (m, 1H), 6.31 – 6.28 (m, 2H), 6.22 (s, 1H), 3.67 (s, 3H), 2.11 (s, 3H); ^{13}C NMR (100 MHz, d_6-DMSO) δ ppm: 170.6, 169.3, 162.3, 149.7, 137.8, 134.3, 131.8, 131.3, 130.6, 130.5, 130.1, 129.9, 128.7, 126.4, 126.3, 119.5, 118.9, 118.6, 114.6, 114.5, 109.4, 82.1, 73.9, 56.0, 26.4; HRMS (ESI) Calcd. For $C_{30}H_{24}Br_2N_3O_3^+$ [M+H]$^+$ 632.0179, found 632.0179.

((5a*R*,10b*R*)-2,3-dibromo-8-chloro-10b-methyl-6,10b-dihydro-5*H*-indolo[2,3-*b*]quinoxaline-5,11(5a*H*)-diyl)bis(phenylmethanone) **5k**:

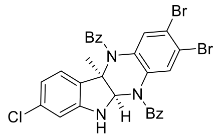

5k

White solid; 59.1 mg; isolated yield: 93%; M.p.: 168 – 169 °C; $[\alpha]_D^{20}$ = -84 (*c* 0.1, MeOH); HPLC (Chiralpak IB-3, *i*-propanol/hexane = 30/70, flow rate 1.0 mL/min, λ = 254 nm), t_1= 7.10 min (major), t_2 = 9.24 min (minor), *ee* = 96%; ^{1}H NMR (400 MHz, d_6-DMSO) δ ppm: 7.67 – 7.65 (m, 2H), 7.63 – 7.41 (m, 9H), 7.11 – 6.88 (m, 2H), 6.78 (s, 1H), 6.67 – 6.65 (m, 1H), 6.42 – 6.41 (m, 1H), 6.22 (s, 1H), 2.10 (s, 3H); ^{13}C NMR (100 MHz, d_6-DMSO) δ ppm: 171.0, 169.6, 151.0, 136.7, 134.5, 134.4, 133.5, 132.1, 132.0, 130.8, 130.5, 129.6, 129.2, 129.1, 128.9, 128.9, 127.6, 119.7, 119.3, 118.1, 108.9, 81.7, 73.2, 25.8; HRMS (ESI) Calcd. For $C_{29}H_{21}Br_2ClN_3O_2^+$ [M+H]$^+$ 635.9684, found 635.9686.

((5a*R*,10b*R*)-2,3-dibromo-10b-ethyl-6,10b-dihydro-5*H*-indolo[2,3-*b*]quinoxaline-5,11(5a*H*)-diyl)bis(phenylmethanone) **5l**:

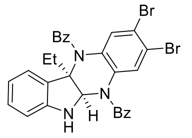

5l

White solid; 58.5 mg; isolated yield: 95%; M.p.: 155 – 156 °C; $[\alpha]_D^{20}$ = -67 (*c* 0.1, MeOH); HPLC (Chiralpak IB-3, *i*-propanol/hexane = 30/70, flow rate 1.0 mL/min, λ = 254 nm), t_1= 5.82 min (major), t_2 = 7.42 min (minor), *ee* = 84%; ^{1}H NMR (400 MHz, d_6-DMSO) δ ppm: 7.74 – 7.64 (m, 3H), 7.59–7.44 (m, 9H), 7.01 – 6.97 (m, 1H), 6.83 – 6.73 (m, 2H), 6.67 – 6.59 (m, 1H), 6.52 – 6.21 (m, 2H), 3.24 – 3.19 (m, 1H), 1.98 – 1.88 (m, 1H), 0.97 (s, 3H); ^{13}C NMR (100 MHz, d_6-DMSO) δ ppm: 170.5, 169.1, 149.7, 136.2, 134.5, 133.5, 132.1, 131.0, 130.9, 130.1, 129.9, 129.6, 129.3, 129.2, 128.7, 126.5, 119.8, 119.3, 118.7, 109.4, 79.0, 77.9, 30.7, 8.3; HRMS (ESI) Calcd. For $C_{30}H_{24}Br_2N_3O_2^+$ $[M+H]^+$ 616.0230, found 616.0227.

((5a*R*,10b*R*)-10b-benzyl-2,3-dibromo-6,10b-dihydro-5*H*-indolo[2,3-*b*]quinoxaline-5,11(5a*H*)-diyl)bis(phenylmethanone) **5m**:

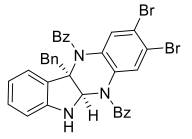

5m

White solid; 63.7 mg; isolated yield: 94%; M.p.: 177 – 178 °C; $[\alpha]_D^{20}$ = -31 (*c* 0.1, MeOH); HPLC (Chiralpak IB-3, *i*-propanol/hexane = 30/70, flow rate 1.0 mL/min, λ = 254 nm), t_1= 5.44 min (major), t_2 = 8,15 min (minor), *ee* = 75%; ^{1}H NMR (400 MHz, d_6-DMSO) δ ppm: 8.01 – 7.91 (m, 1H), 7.57 – 7.31 (m, 11H), 7.11-7.02 (m, 5H), 6.86 – 6.62 (m, 5H), 6.42 – 6.40 (m, 1H), 4.37 (s, 1H), 3.77 (s, 1H); ^{13}C NMR (100 MHz, d_6-DMSO) δ ppm: 171.1, 169.2, 150.1, 136.8, 136.1, 134.5, 133.8, 132.1, 131.9, 131.2, 130.7, 130.3, 129.8, 129.4, 129.0, 129.0, 128.8, 128.6, 127.3, 127.2, 119.4, 119.1, 118.7, 109.6, 78.3, 77.5, 41.3; HRMS (ESI) Calcd. For $C_{35}H_{26}Br_2N_3O_2^+$ $[M+H]^+$ 678.0386, found 678.0388.

((5a*R*,10b*R*)-2,3-difluoro-10b-methyl-6,10b-dihydro-5*H*-indolo[2,3-*b*]quinoxaline-5,11(5a*H*)-diyl)bis(phenylmethanone) **5n**:

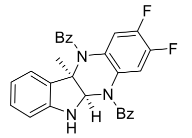

5n

White solid; 46.3 mg; isolated yield: 96%; M.p.: 143 – 144 °C; $[\alpha]^{20}{}_D$ = -112 (*c* 0.1, MeOH); HPLC (Chiralpak IB, *i*-propanol/hexane = 30/70, flow rate 1.0 mL/min, λ = 254 nm), t_1= 3.52 min (minor), t_2 = 4.74 min (major), *ee* = 98%; ^{1}H NMR (400 MHz, $CDCl_3$) δ ppm: 7.81 – 7.79 (m, 1H), 7.67 – 7.59 (m, 4H), 7.16 – 7.03 (m, 6H), 6.80 – 6.76 (m, 2H), 6.61 (s, 1H), 6.47 – 6.45 (m, 1H), 6.29 (s, 1H), 5.28 (s, 1H), 4.63 (s, 1H), 2.20 (s, 3H); ^{13}C NMR (100 MHz, $CDCl_3$) δ ppm: 169.7, 168.7, 164.1 (d, *J* = 248 Hz), 164.1 (d, *J* = 249 Hz),149.5, 137.2, 133.7, 133.3, 132.4, 132.3, 131.7 (d, *J* = 9 Hz), 131.1 (d, *J* = 8 Hz), 131.1, 131.0, 130.9, 130.9, 130.6, 129.9, 129.9, 126.3, 119.8, 119.3,

118.7, 116.3 (d, J = 21 Hz), 116.2 (d, J = 20 Hz)109.5, 81.8, 74.0, 25.9; ^{19}F NMR (376 MHz, DMSO) δ ppm: -139.51, -139.57; HRMS (ESI) Calcd. For $C_{29}H_{22}F_2N_3O_2^+$ $[M+H]^+$ 482.1675, found 482.1677.

((5a*R*,10b*R*)-2,3-dichloro-10b-methyl-6,10b-dihydro-5*H*-indolo[2,3-*b*]quinoxaline-5,11(5a*H*)-diyl)bis(phenylmethanone) **5o**:

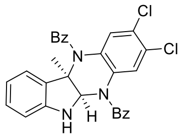

5o

White solid; 46.3 mg; isolated yield: 90%; M.p.: 147 – 148 °C; $[\alpha]_D^{20}$ = -76(c 0.1, MeOH); HPLC (Chiralpak IB, *i*-propanol/hexane = 30/70, flow rate 1.0 mL/min, λ = 254 nm), t_1= 6.04 min (major), t_2 = 7.65 min (minor), *ee* = 94%; ^{1}H NMR (400 MHz, d_6-DMSO) δ ppm: 7.72 – 7.69 (m, 3H), 7.63 – 7.58 (m, 2H), 7.57 – 7.51 (m, 3H), 7.48 – 7.40 (m, 3H), 6.99 – 6.85 (m, 2H), 6.72 – 6.63 (m, 3H), 6.51 – 6.44 (m, 1H), 6.22 (s, 1H), 2.11 (s, 3H); ^{13}C NMR (100 MHz, d_6-DMSO) δ ppm: 170.9, 169.7, 149.5, 136.9, 136.8, 134.5, 133.2, 132.0, 131.9, 129.9, 129.9, 129.6, 129.2, 129.0, 128.8, 128.2, 127.9, 127.6, 127.6, 127.5, 127.0, 126.3, 118.7, 109.5, 81.9, 74.0, 26.0; HRMS (ESI) Calcd. For $C_{29}H_{22}Cl_2N_3O_2^+$ $[M+H]^+$ 514.1084, found 514.1082.

((5a*R*,10b*R*)-2,3,10b-trimethyl-6,10b-dihydro-5*H*-indolo[2,3-*b*]quinoxaline-5,11(5a*H*)-diyl)bis(phenylmethanone) **5p**:

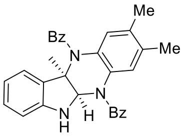

5p

White solid; 40.3 mg; isolated yield: 85%; M.p.: 172 – 173 °C; $[\alpha]_D^{20}$ = -66(c 0.1, MeOH); HPLC (Chiralpak IB-3, *i*-propanol/hexane = 30/70, flow rate 1.0 mL/min, λ = 254 nm), t_1= 12.58 min (major), t_2 = 22.68 min (minor), *ee* = 92%; ^{1}H NMR (400 MHz, d_6-DMSO) δ ppm: 7.67 – 7.58 (m, 3H), 7.55 – 7.33 (m, 10H), 6.94 – 6.90 (m, 1H), 6.61 – 6.57 (m, 2H), 6.39 – 6.30 (m, 1H), 6.14 (s, 1H), 2.09 (s, 3H), 1.77 (s, 3H), 1.66 (s, 3H); ^{13}C NMR (100 MHz, d_6-DMSO) δ ppm: 171.1, 169.6, 149.8, 137.8, 135.4, 133.6, 133.0, 131.5, 131.4, 130.5, 129.6, 129.4, 129.0, 128.8, 127.8, 127.4, 126.1, 118.3, 109.1, 81.9, 73.5, 26.8, 19.1, 19.0; HRMS (ESI) Calcd. For $C_{31}H_{28}N_3O_2^+$ $[M+H]^+$ 474.2176, found 474.2177.

((5a*R*,10b*R*)-(4 or 5)-chloro-10b-methyl-6,10b-dihydro-5*H*-indolo[2,3-*b*]quinoxaline-5,11(5a*H*)-diyl)bis(phenylmethanone) **5q**:

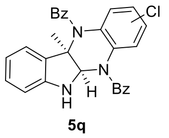

5q

White solid; 40.8 mg; isolated yield: 85%; M.p.: 172 – 173 °C;
Up: $[\alpha]_D^{20}$ = -66 (c 0.1, MeOH); HPLC (Chiralpak IB-3, *i*-propanol/hexane = 30/70,

flow rate 1.0 mL/min, λ = 254 nm), t_1= 12.08 min (major), t_2 = 13.32 min (minor), *ee* = 90%; ^{1}H NMR (400 MHz, d_6-DMSO) δ ppm: 7.69 – 7.52 (m, 9H), 7.48 – 7.40 (m, 3H), 6.97 – 6.93 (m, 1H), 6.70 – 6.60 (m, 3H), 6.43 – 6.38 (m, 2H), 6.18 (s, 1H), 2.10 (s, 3H); ^{13}C NMR (100 MHz, d_6-DMSO) δ ppm: 171.0, 169.7, 149.6, 137.1, 135.8, 134.7, 134.4, 131.8, 130.1, 129.8, 129.6, 129.1, 128.9, 128.8, 128.8, 128.2, 128.0, 126.3, 126.0, 125.6, 118.5, 109.3, 81.9, 73.9, 26.3; HRMS (ESI) Calcd. For $C_{29}H_{23}ClN_3O_2^+$ $[M+H]^+$ 480.1473, found 480.1476.
Down: $[\alpha]_D^{20}$ = -48 (*c* 0.1, MeOH); HPLC (Chiralpak IA, *i*-propanol/hexane = 30/70, flow rate 1.0 mL/min, λ = 254 nm), t_1= 4.75 min (minor), t_2 = 9.99 min (major), *ee* = 92%; ^{1}H NMR (400 MHz, d_6-DMSO) δ ppm: 7.66 – 7.42 (m, 11H), 6.98 – 6.91 (m, 2H), 6.69 – 6.53 (m, 4H), 6.40 – 6.39 (m, 1H), 6.23 (s, 1H), 2.12 (s, 3H); ^{13}C NMR (100 MHz, d_6-DMSO) δ ppm: 170.9, 169.7, 149.6, 137.4, 137.0, 134.7, 133.8, 132.1, 132.0, 130.9, 130.7, 130.1, 130.0, 129.7, 129.3, 129.1, 128.9, 126.4, 119.8, 119.3, 118.8, 109.6, 81.8, 74.0, 26.1; HRMS (ESI) Calcd. For $C_{29}H_{23}ClN_3O_2^+$ $[M+H]^+$ 480.1473, found 480.1474.

((5a*R*,10b*R*)-(4 or 5)-bromo-10b-methyl-6,10b-dihydro-5*H*-indolo[2,3-*b*]quinoxaline -5,11(5a*H*)-diyl)bis(phenylmethanone) **5r**:

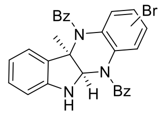

5r

White solid; 39.8 mg; isolated yield: 76%; M.p.: 145 – 146 °C;
Up: $[\alpha]_D^{20}$ = -65 (*c* 0.1, MeOH); HPLC (Chiralpak IB-3, *i*-propanol/hexane = 30/70, flow rate 1.0 mL/min, λ = 254 nm), t_1 = 10.25 min (major), t_2 = 13.11 min (minor), *ee* = 91%; ^{1}H NMR (400 MHz, d_6-DMSO) δ ppm: 7.69 – 7.52 (m, 9H), 7.49 – 7.40 (m, 3H), 6.97 – 6.93 (m, 1H), 6.82 – 6.79 (m, 1H), 6.67 – 6.60 (m, 2H), 6.40 – 6.32 (m, 2H), 6.18 (s, 1H), 2.10 (s, 3H); ^{13}C NMR (100 MHz, d_6-DMSO) δ ppm: 171.1, 169.7, 149.6, 137.1, 135.9, 134.7, 134.4, 131.8, 130.1, 129.8, 129.6, 129.1, 128.9, 128.8, 128.8, 128.2, 128.0, 126.3, 126.0, 125.6, 118.5, 109.3, 81.9, 74.0, 26.3; HRMS (ESI) Calcd. For $C_{29}H_{23}BrN_3O_2^+$ $[M+H]^+$ 524.0968, found 524.0972.
Down: $[\alpha]_D^{20}$ = -80 (*c* 0.1, MeOH); HPLC (Chiralpak IB-3, *i*-propanol/hexane = 30/70, flow rate 1.0 mL/min, λ = 254 nm), t_1= 17.72 min (major), t_2 = 22.81 min (minor), *ee* = 93%; ^{1}H NMR (400 MHz, d_6-DMSO) δ ppm: 7.68 – 7.62 (m, 2H), 7.58 – 7.52 (m, 6H), 7.48 – 7.39 (m, 3H), 6.97 – 6.93 (m, 1H), 6.70 – 6.60 (m, 4H), 6.40 – 6.38 (m, 2H), 6.18 (s, 1H), 2.10 (s, 3H); ^{13}C NMR (100 MHz, d_6-DMSO) δ ppm: 171.1, 169.7, 149.7, 137.2, 135.9, 134.8, 134.5, 131.9, 130.2, 129.9, 129.7, 129.2, 129.0, 128.9, 128.9, 128.3, 128.1, 126.4, 126.1, 125.7, 118.6, 109.3, 82.0, 74.0, 26.4; HRMS (ESI) Calcd. For $C_{29}H_{23}BrN_3O_2^+$ $[M+H]^+$ 524.0968, found 524.0969.

((5a*R*,10b*R*)-2,3-dibromo-10b-methyl-6,10b-dihydro-5*H*-indolo[2,3-*b*]quinoxaline-5, 11(5a*H*)-diyl)bis(*p*-tolylmethanone) **5s**:

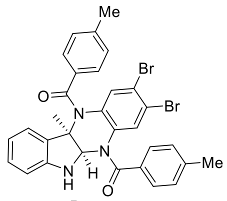

5s

White solid; 58.6 mg; isolated yield: 93%; M.p.: 122 – 123 °C; $[\alpha]_D^{20}$ = -69 (*c* 0.1, MeOH); HPLC (Chiralpak IB-3, *i*-propanol/hexane = 30/70, flow rate 1.0 mL/min, λ = 254 nm), t_1= 6.06 min (minor), t_2 = 6.66 min (major), *ee* = 96%; ^{1}H NMR (400 MHz, d_6-DMSO) δ ppm: 7.69 – 7.63 (m, 1H), 7.56 – 7.48 (m, 4H), 7.42 – 7.32 (m, 2H), 7.27 – 7.25 (m, 2H), 6.99 – 6.95 (m, 2H), 6.72 (s, 1H), 6.69 – 6.61 (m, 2H), 6.45 – 6.39 (m, 1H), 6.16 (s, 1H), 2.38 (s, 3H), 2.33 (s, 3H), 2.07 (s, 3H); ^{13}C NMR (100 MHz, d_6-DMSO) δ ppm: 170.9, 169.6, 149.6, 142.2, 142.1, 137.4, 134.0, 131.6, 130.6, 130.5, 129.9, 129.9, 129.7, 129.7, 129.6, 129.0, 126.2, 119.5, 119.0, 118.6, 109.4, 81.7, 73.9, 26.1, 21.5, 21.4; HRMS (ESI) Calcd. For $C_{31}H_{26}Br_2N_3O_2^+$ [M+H]$^+$ 630.0386, found 630.0385.

((5a*R*,10b*R*)-2,3-dibromo-10b-methyl-6,10b-dihydro-5*H*-indolo[2,3-*b*]quinoxaline-5,11(5a*H*)-diyl)bis((4-methoxyphenyl)methanone) **5t**:

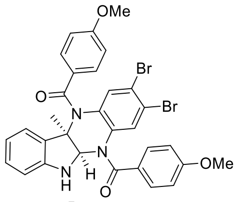

5t

White solid; 63.6 mg; isolated yield: 96%; M.p.: 148 – 149 °C; $[\alpha]_D^{20}$ = -73 (*c* 0.1, MeOH); HPLC (Chiralpak IB-3, *i*-propanol/hexane = 30/70, flow rate 1.0 mL/min, λ = 254 nm), t_1= 26.76 min (minor), t_2 = 28.80 min (major), *ee* = 97%; ^{1}H NMR (400 MHz, d_6-DMSO) δ ppm: 7.66 – 7.55 (m, 5H), 7.13 – 7.08 (m, 2H), 7.03 – 6.95 (m, 4H), 6.73 (s, 1H), 6.66 – 6.61 (m, 2H), 6.45 – 6.40 (m, 1H), 6.21 – 6.14 (m, 1H), 3.85 (s, 3H), 3.82 (s, 3H), 2.05 (s, 3H); ^{13}C NMR (100 MHz, d_6-DMSO) δ ppm: 170.5, 169.3, 162.3, 162.3, 149.6, 137.7, 134.2, 131.8, 131.2, 130.5, 130.4, 130.0, 129.8, 128.7, 126.3, 126.2, 119.4, 118.8, 118.5, 114.5, 114.4, 109.3, 82.0, 73.8, 56.0, 56.0, 26.3; HRMS (ESI) Calcd. For $C_{31}H_{26}Br_2N_3O_4^+$ [M+H]$^+$ 662.0285, found 662.0289.

((5a*R*,10b*R*)-2,3-dibromo-10b-methyl-6,10b-dihydro-5*H*-indolo[2,3-*b*]quinoxaline-5,11(5a*H*)-diyl)bis((4-fluorophenyl)methanone) **5u**:

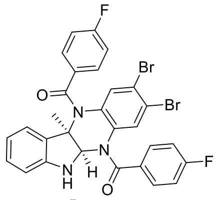

5u

White solid; 61.2 mg; isolated yield: 96%; M.p.: 152 – 153 °C; $[\alpha]_D^{20}$ = -104 (*c* 0.1,

MeOH); HPLC (Chiralpak IB-3, *i*-propanol/hexane = 30/70, flow rate 1.0 mL/min, λ = 254 nm), t_1= 6.14 min (minor), t_2 = 6.64 min (major), *ee* = 95%; ^{1}H NMR (400 MHz, d_6-DMSO) δ ppm: 7.77 – 7.65 (m, 5H), 7.45 – 7.41 (m, 2H), 7.34 – 7.29 (m, 2H), 7.08 – 6.95 (m, 2H), 6.81 (s, 1H), 6.68 – 6.60 (m, 2H), 6.44 – 6.38 (m, 1H), 6.17 (s, 1H), 2.11 (s, 3H); ^{13}C NMR (100 MHz, d_6-DMSO) δ ppm: 169.9, 168.9, 164.3 (d, *J* = 248 Hz), 164.3 (d, *J* = 249 Hz), 149.7, 137.4, 133.8, 133.5, 133.4, 132.6, 132.5, 131.9 (d, *J* = 9 Hz), 131.2 (d, *J* = 9 Hz), 131.1, 130.8, 130.1, 130.1, 126.5, 119.9, 119.5, 118.9, 116.5 (d, *J* = 22 Hz), 116.1 (d, *J* = 17 Hz), 109.7, 82.0, 74.2, 26.1; ^{19}F NMR (376 MHz, d_6-DMSO) δ ppm: -105.36, -106.08; HRMS (ESI) Calcd. For $C_{29}H_{20}Br_2F_2N_3O_2^+$ $[M+H]^+$ 637.9885, found 637.9887.

((5a*R*,10b*R*)-2,3-dibromo-10b-methyl-6,10b-dihydro-5*H*-indolo[2,3-*b*]quinoxaline-5, 11(5a*H*)-diyl)bis((4-chlorophenyl)methanone) **5v**:

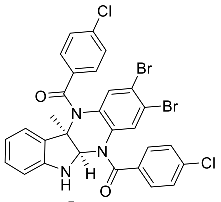

5v

White solid; 63.0 mg; isolated yield: 94%; M.p.: 166 – 167 °C; $[\alpha]_D^{20}$ = -86 (*c* 0.1, MeOH); HPLC (Chiralpak IB-3, *i*-propanol/hexane = 30/70, flow rate 1.0 mL/min, λ = 254 nm), t_1= 5.92 min (minor), t_2 = 6.95 min (major), *ee* = 93%; ^{1}H NMR (400 MHz, d_6-DMSO) δ ppm: 7.71 – 7.54 (m, 10H), 6.99 – 6.95 (m, 1H), 6.84 (s, 1H), 6.72 – 6.58 (m, 2H), 6.45 – 6.43 (m, 1H), 6.19 (s, 1H), 2.12 (s, 3H); ^{13}C NMR (100 MHz, d_6-DMSO) δ ppm: 169.7, 168.6, 149.5, 136.7, 135.6, 133.5, 133.3, 131.5, 130.0, 129.8, 129.4, 129.2, 126.3, 119.9, 119.6, 118.8, 109.6, 81.6, 74.1, 25.9; HRMS (ESI) Calcd. For $C_{29}H_{20}Br_2Cl_2N_3O_2^+$ $[M+H]^+$ 669.9294, found 669.9294.

((5a*R*,10b*R*)-2,3-dibromo-10b-methyl-6,10b-dihydro-5*H*-indolo[2,3-*b*]quinoxaline-5, 11(5a*H*)-diyl)bis((4-bromophenyl)methanone) **5w**:

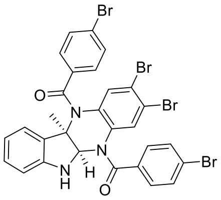

5w

White solid; 72.0 mg; isolated yield: 95%; M.p.: 173 – 174 °C; $[\alpha]_D^{20}$ = -98 (*c* 0.1, MeOH); HPLC (Chiralpak IB-3, *i*-propanol/hexane = 30/70, flow rate 1.0 mL/min, λ = 254 nm), t_1= 6.19 min (minor), t_2 = 7.34 min (major), *ee* = 96%; ^{1}H NMR (400 MHz, d_6-DMSO) δ ppm: 7.82 – 7.80 (m, 2H), 7.74 – 7.51 (m, 7H), 7.24 – 6.95 (m, 2H), 6.83 (s, 1H), 6.66 – 6.62 (m, 2H), 6.44 – 6.39 (m, 1H), 6.18 (s, 1H), 2.11 (s, 3H); ^{13}C NMR (100 MHz, d_6-DMSO) δ ppm: 169.8, 168.7, 149.5, 136.9, 135.9, 133.6, 133.5, 132.3, 132.1, 132.0, 131.6, 130.9, 130.6, 130.4, 130.0, 129.8, 126.3, 125.7, 125.6, 119.9, 119.6, 118.8, 109.6, 81.6, 74.1, 25.9; HRMS (ESI) Calcd. For $C_{29}H_{20}Br_4N_3O_2^+$ $[M+H]^+$

757.8284, found 757.8285.

((5aR,10bR)-2,3-dibromo-10b-methyl-6,10b-dihydro-5H-indolo[2,3-b]quinoxaline-5, 11(5aH)-diyl)bis(m-tolylmethanone) **5x**:

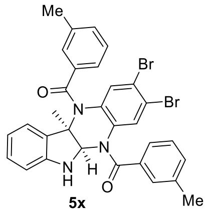

5x

White solid; 59.9 mg; isolated yield: 95%; M.p.: 188 – 189 °C; $[\alpha]_D^{20}$ = -75 (c 0.1, MeOH); HPLC (Chiralpak IB-3, i-propanol/hexane = 30/70, flow rate 1.0 mL/min, λ = 254 nm), t_1= 5.68 min (major), t_2 = 6.41 min (minor), ee = 93%; ^{1}H NMR (400 MHz, $CDCl_3$) δ ppm: 7.84 – 7.82 (m, 1H), 7.54 – 7.48 (m, 2H), 7.40 – 7.23 (m, 6H), 7.07 – 7.03 (m, 1H), 6.80 – 6.72 (m, 2H), 6.62 (s, 1H), 6.48 – 6.46 (m, 1H), 6.35 (s, 1H), 4.59 (s, 1H), 2.41-2.37 (m, 6H), 2.23 (s, 3H); ^{13}C NMR (100 MHz, $CDCl_3$) δ ppm: 171.2, 170.5, 148.0, 138.8, 138.6, 136.2, 136.1, 133.5, 133.2, 133.0, 132.6, 130.7, 129.9, 129.8, 129.7, 129.7, 128.4, 128.4, 126.7, 126.3, 125.9, 120.0, 119.9, 119.6, 109.5, 81.2, 73.8, 26.4, 21.5, 21.4; HRMS (ESI) Calcd. For $C_{31}H_{26}Br_2N_3O_2^+$ $[M+H]^+$ 630.0386, found 630.0385.

((5aR,10bR)-2,3-dibromo-10b-methyl-6,10b-dihydro-5H-indolo[2,3-b]quinoxaline-5, 11(5aH)-diyl)bis((3-fluorophenyl)methanone) **5y**:

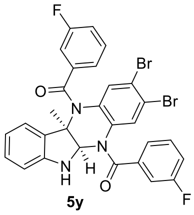

5y

White solid;; 58.7 mg; isolated yield: 92%; M.p.: 145 – 146 °C $[\alpha]_D^{20}$ = -58 (c 0.1, MeOH); HPLC (Chiralpak IB-3, i-propanol/hexane = 30/70, flow rate 1.0 mL/min, λ = 254 nm), t_1= 7.23 min (major), t_2 = 7.87 min (minor), ee = 93%; ^{1}H NMR (400 MHz, d_6-DMSO) δ ppm: 7.66 – 7.59 (m, 1H), 7.57 – 7.29 (m, 9H), 7.00 – 6.96 (m, 1H), 6.91 (s, 1H), 6.69 – 6.63 (m, 2H), 6.51 – 6.35 (m, 1H), 6.15 (s, 1H), 2.12 (s, 3H); ^{13}C NMR (100 MHz, d_6-DMSO) δ ppm: 169.3, 168.2, 162.2 (d, J = 244 Hz), 162.1 (d, J = 244 Hz), 149.4, 139.4, 139.2, 136.9, 136.8, 133.3, 131.4 (d, J = 8 Hz), 131.0 (d, J = 8 Hz), 130.9, 130.6, 130.0, 129.8, 126.2, 125.6, 124.9, 120.0, 119.6, 118.7,118.7, 116.6 (d, J = 22 Hz), 115.9 (d, J = 23 Hz), 109.6, 81.8, 74.1, 25.7; ^{19}F NMR (376 MHz, d_6-DMSO) δ ppm: -105.79, -106.50; HRMS (ESI) Calcd. For $C_{29}H_{20}Br_2F_2N_3O_2^+$ $[M+H]^+$ 637.9885, found 637.9888.

((5aR,10bR)-2,3-dibromo-10b-methyl-6,10b-dihydro-5H-indolo[2,3-b]quinoxaline-5, 11(5aH)-diyl)bis((2-fluorophenyl)methanone) **5z**:

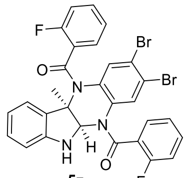

White solid; 60.0 mg; isolated yield: 94%; M.p.: 180 – 181 °C; $[\alpha]_D^{20}$ = -14 (*c* 0.1, MeOH); HPLC (Chiralpak IB-3, *i*-propanol/hexane = 30/70, flow rate 1.0 mL/min, λ = 254 nm), t_1= 7.16 min (minor), t_2 = 9.57 min (major), *ee* = 63%; ^{1}H NMR (400 MHz, d_6-DMSO) δ ppm: 7.69 – 7.59 (m, 5H), 7.52 – 7.33 (m, 4H), 7.28 – 7.20 (m, 2H), 7.03 – 6.99 (m, 1H), 6.86 (s, 1H), 6.70 – 6.67 (m, 1H), 6.58 (s, 1H), 6.46 – 6.44 (m, 1H), 2.09 (s, 3H); ^{13}C NMR (100 MHz, d_6-DMSO) δ ppm: 169.4, 168.2, 162.2 (d, *J* = 248 Hz), 162.2 (d, *J* = 248 Hz), 149.5, 139.4, 139.3, 137.0, 136.9, 133.4, 131.4 (d, *J* = 9 Hz), 131.1 (d, *J* = 8 Hz), 131.0, 130.9, 130.7, 130.1, 129.8, 126.2, 119.7, 118.9, 118.7, 116.7 (d, *J* = 21 Hz), 115.9 (d, *J* = 24 Hz), 109.6, 81.8, 74.1, 25.8; ^{19}F NMR (376 MHz, d_6-DMSO) δ ppm: -113.48, -114.78; HRMS (ESI) Calcd. For $C_{29}H_{20}Br_2F_2N_3O_2^+$ $[M+H]^+$ 637.9885, found 637.9885.

((5a*R*,10b*R*)-2,3-dibromo-10b-methyl-6,10b-dihydro-5*H*-indolo[2,3-*b*]quinoxaline-5,11(5a*H*)-diyl)bis(naphthalen-2-ylmethanone) **5a'**:

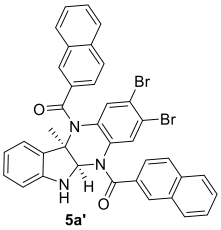

White solid; 64.6 mg; isolated yield: 92%; M.p.: 203 – 204 °C; $[\alpha]_D^{20}$ = -76 (*c* 0.1, MeOH); HPLC (Chiralpak IB-3, *i*-propanol/hexane = 30/70, flow rate 1.0 mL/min, λ = 254 nm), t_1= 10.99 min (major), t_2 = 13.87 min (minor), *ee* = 95%; ^{1}H NMR (400 MHz, d_6-DMSO) δ ppm: 8.43 – 8.37 (m, 2H), 8.09 – 7.99 (m, 6H), 7.77 – 7.63 (m, 7H), 7.06 – 6.93 (m, 2H), 6.85 – 6.80 (m, 2H), 6.71 – 6.67 (m, 1H), 6.49 – 6.47 (m, 1H), 6.33 (s, 1H), 2.20 (s, 3H); ^{13}C NMR (100 MHz, d_6-DMSO) δ ppm: 170.9, 169.6, 149.6, 137.4, 134.4, 133.9, 132.6, 132.5, 132.0, 130.8, 130.7, 130.4, 130.0, 129.7, 129.3, 129.2, 128.6, 128.3, 128.3, 127.6, 127.6, 126.3, 125.9, 125.4, 119.7, 119.2, 118.7, 109.5, 82.0, 74.1, 26.1; HRMS (ESI) Calcd. For $C_{37}H_{26}Br_2N_3O_2^+$ $[M+H]^+$ 702.0386, found 702.0388.

1,1'-((5a*R*,10b*R*)-2,3-dibromo-10b-methyl-6,10b-dihydro-5*H*-indolo[2,3-*b*]quinoxaline-5,11(5a*H*)-diyl)bis(2,2-dimethylpropan-1-one) **5b'**:

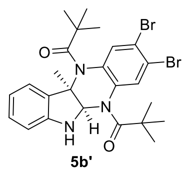

White solid; 44.8 mg; isolated yield: 86%; M.p.: 104 – 105 °C; $[\alpha]_D^{20}$ = -26 (*c* 0.1, MeOH); HPLC (Chiralpak IB-3, *i*-propanol/hexane = 30/70, flow rate 1.0 mL/min, λ =

254 nm), t_1= 11.03 min (major), t_2 = 15.00 min (minor), *ee* = 65%; ^{1}H NMR (400 MHz, d_6-DMSO) δ ppm: 7.64 – 7.45 (m, 2H), 6.92 – 6.86 (m, 2H), 6.58 – 6.45 (m, 2H), 6.36 – 6.32 (m, 1H), 6.16 – 6.10 (m, 1H), 1.87 (s, 3H), 1.37 (s, 9H), 1.06 (s, 9H); ^{13}C NMR (100 MHz, d_6-DMSO) δ ppm: 183.5, 177.3, 149.5, 138.6, 135.2, 132.4, 129.7, 129.6, 127.8, 126.2, 120.0, 119.4, 118.2, 108.7, 81.9, 75.6, 43.6, 29.9, 29.4, 26.5; HRMS (ESI) Calcd. For $C_{25}H_{30}Br_2F_2N_3O_2^+$ $[M+H]^+$ 522.0699, found 522.0696.

(Racemic) (2,3-dibromo-6,10b-dimethyl-6,10b-dihydro-5*H*-indolo[2,3-*b*]quinoxaline -5,11(5a*H*)-diyl)bis(phenylmethanone) **5c'**:

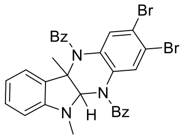

5c'

White solid; 48.0 mg; isolated yield: 90%; M.p.: 157 – 158 °C; ^{1}H NMR (400 MHz, d_6-DMSO) δ 7.85 – 7.82 (m, 1H), 7.75 – 7.74 (m, 2H), 7.57 – 7.55 (m, 5H), 7.46 – 7.41 (m, 3H), 7.11 – 7.09 (m, 2H), 6.75 – 6.72 (m, 2H), 6.45 – 6.43 (m, 1H), 6.14 (s, 1H), 2.70 (s, 3H), 2.17 (s, 3H); ^{13}C NMR (100 MHz, d_6-DMSO) δ ppm: 170.6, 169.8, 150.6, 137.1, 136.9, 134.6, 133.1, 131.8, 131.7, 130.6, 130.4, 130.4, 129.7, 129.4, 129.0, 127.0, 119.7, 119.2, 119.0, 108.1, 85.6, 72.5, 32.3, 24.6; HRMS (ESI) Calcd. For $C_{30}H_{23}Br_2N_3O_2Na^+$ $[M+Na]^+$ 638.0049, found 638.0053.

N,N'-(3-(2,3-dimethyl-1*H*-indol-6-yl)-4,5-dimethyl-1,2-phenylene)dibenzamide **6:**

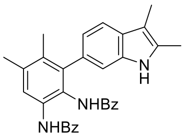

6

Brown oil; 31.2 mg; isolated yield = 64%; ^{1}H NMR (400 MHz, d_6-DMSO) δ ppm: 10.60 (s, 1H), 9.51 (s, 1H), 7.77 – 7.66 (m, 2H), 7.51 – 7.44 (m, 1H), 7.43 – 7.33 (m, 5H), 7.23 – 7.08 (m, 5H), 6.87 – 6.78 (m, 2H), 2.23 (s, 6H), 2.10 (s, 3H), 2.06 (s, 3H); ^{13}C NMR (100 MHz, d_6-DMSO) δ ppm: 170.2, 166.2, 137.4, 135.1, 135.0, 133.1, 131.8, 131.5, 130.1, 129.1, 128.6, 128.0, 127.6, 117.6, 105.6, 19.4, 19.3, 11.6, 8.6; HRMS (ESI) Calcd. For $C_{32}H_{30}N_3O_2^+$ $[M+H]^+$ 488.2333, found 488.2337.

Di-*tert*-butyl (4-((3*S*)-2,3-dimethylindolin-3-yl)-1,2-phenylene)dicarbamate **7:**

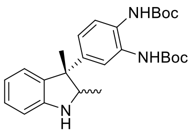

7

Brown solid; 41.7 mg; isolated yield = 92%; M.p.: 67 – 68 °C; dr = 6:1; $[\alpha]_D^{20}$ = -95 (*c* 0.10, MeOH); HPLC (Chiralpak IE-3, *i*-propanol/hexane = 20/80, flow rate 1.0 mL/min, λ = 254 nm), major product: t_1 = 6.88 min (minor), t_2 = 8.00 min (major), ee = 92%; minor product: t_1 = 9.43 min (minor), t_2 = 10.84 min (major), ee = 87%; ^{1}H NMR (400 MHz, d_6-DMSO) δ ppm: 8.41 (d, 2H), 7.48 – 7.28 (m, 1H), 7.16 (s, 1H), 7.08 – 6.93

(m, 1H), 6.84 (d, J = 7.2 Hz, 1H), 6.77 – 6.68 (m, 1H), 6.65 – 6.54 (m, 2H), 5.61 (s, 1H), 3.77 – 3.60 (m, 1H), 1.64 – 1.31 (m, 3H), 1.45 (t, J = 8.1 Hz, 18H), 1.10 – 0.66 (m, 3H); ^{13}C NMR (100 MHz, d_6-DMSO) δ ppm: 153.8, 153.7, 151.6, 151.0, 140.0, 137.8, 129.7, 128.8, 127.9, 124.4, 124.0, 123.3, 118.0, 117.9, 1093, 109.2, 79.7 67.2, 66.4, 51.8, 51.1, 28.5, 25.9, 17.3; HRMS (ESI) Calcd. For $C_{26}H_{36}N_3O_4^+$ $[M+H]^+$ 454.2700, found 454.2703.

(*S*)-4-(2,3-dimethyl-3*H*-indol-3-yl)benzene-1,2-diamine **8:**

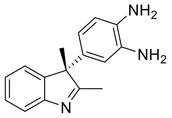

8

Red oil; 22.3 mg; isolated yield = 89%; $[\alpha]_D^{20}$ = -33 (*c* 0.33, MeOH); HPLC (Chiralpak IG-3, *i*-propanol/hexane = 30/70, flow rate 1.0 mL/min, λ = 254 nm), t_1 = 10.85 min (major), t_2 = 12.73 min (minor), *ee* = 92%; ^{1}H NMR (400 MHz, d_6-DMSO) δ ppm: 7.45 (d, J = 7.6 Hz, 1H), 7.32 – 7.18 (m, 1H), 7.18 – 7.01 (m, 2H), 6.44 (d, J = 8.0 Hz, 1H), 6.28 – 6.19 (m, 1H), 6.02 (d, J = 1.9 Hz, 1H), 4.42 (s, 4H), 2.02 (s, 3H), 1.49 (s, 3H); ^{13}C NMR (100 MHz, d_6-DMSO) δ ppm: 188.1, 154.7, 148.3, 135.6, 134.5, 128.0, 127.7, 125.5, 122.8, 119.8, 115.0, 112.2, 61.3, 20.6, 16.0; HRMS (ESI) Calcd. For $C_{16}H_{18}N_3^+$ $[M+H]^+$ 252.1495, found 252.1501.

(*S*)-5-(2,3-dimethyl-3*H*-indol-3-yl)-1,3-dihydro-2*H*-benzo[*d*]imidazol-2-one **9:**

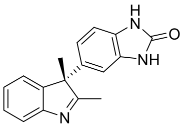

9

Yellow oil; 26.0 mg; isolated yield = 94%; $[\alpha]_D^{20}$ = -29 (*c* 0.25, MeOH); HPLC (Chiralpak IG-3, *i*-propanol/hexane = 30/70, flow rate 1.0 mL/min, λ = 254 nm), t_1= 4.82 min (major), t_2 = 5.48 min (minor), *ee* = 92%; ^{1}H NMR (400 MHz, d_6-DMSO) δ ppm: 10.58 (s, 1H), 10.45 (s, 1H), 7.50 (d, J = 7.7 Hz, 1H), 7.33 – 7.25 (m, 1H), 7.19 – 7.10 (m, 2H), 6.86 (d, J = 8.1 Hz, 1H), 6.71 – 6.64 (m, 1H), 6.42 (d, J = 1.5 Hz, 1H), 2.02 (s, 3H), 1.61 (s, 3H); ^{13}C NMR (100 MHz, d_6-DMSO) δ ppm: 187.5, 155.8, 154.7, 148.0, 132.0, 130.5, 129.3, 128.1, 125.9, 123.0, 120.0, 118.5, 109.1, 106.3, 61.7, 20.5, 15.9; HRMS (ESI) Calcd. For $C_{17}H_{16}N_3O^+$ $[M+H]^+$ 278.1288, found 278.1294.

(*S*)-5-(2,3-dimethyl-3*H*-indol-3-yl)-1,3-dihydro-2*H*-benzo[*d*]imidazole-2-thione **10 :**

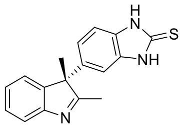

10

Brown solid; 28.1 mg; isolated yield = 96%; M.p.: 75 – 76 °C; $[\alpha]_D^{20}$ = -70 (*c* 0.23, MeOH); HPLC (Chiralpak IG-3, *i*-propanol/hexane = 30/70, flow rate 1.0 mL/min, λ = 254 nm), t_1 = 10.67 min (major), t_2 = 12.07 min (minor), *ee* = 92%; ^{1}H NMR (400 MHz, d_6-DMSO) δ ppm: 12.54 (s, 1H), 12.45 (s, 1H), 7.53 (d, J = 7.7 Hz, 1H), 7.35 – 7.27

(m, 1H), 7.18 – 7.12 (m, 2H), 7.08 (s, 1H), 6.80 – 6.70 (m, 2H), 2.03 (s, 3H), 1.64 (s, 3H); ^{13}C NMR (100 MHz, d_6-DMSO) δ ppm: 187.3, 169.0, 154.6, 147.8, 134.2, 133.1, 131.8, 128.2, 126.0, 123.0, 120.7, 120.1, 110.3, 107.1, 61.7, 20.5, 15.9; HRMS (ESI) Calcd. For $C_{17}H_{16}N_3S^+$ [M+H]$^+$ 294.1059, found 294.1060.

(*S*)-6-(2,3-dimethyl-3*H*-indol-3-yl)quinoxaline **11:**

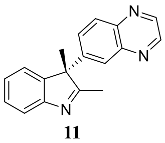

11

Yellow oil; 26.2 mg; isolated yield = 96%; $[\alpha]_D^{20}$ = -119 (*c* 0.35, MeOH); HPLC (Chiralpak IC-3, *i*-propanol/hexane = 30/70, flow rate 1.0 mL/min, λ = 254 nm), t_1 = 4.98 min (major), t_2 = 6.00 min (minor), *ee* = 92%; ^{1}H NMR (400 MHz, d_6-DMSO) δ ppm: 8.97 (d, *J* = 1.8 Hz, 1H), 8.93 (d, *J* = 1.8 Hz, 1H), 8.07 (d, *J* = 2.0 Hz, 1H), 7.96 (d, *J* = 8.8 Hz, 1H), 7.60 (d, *J* = 7.7 Hz, 1H), 7.42 – 7.34 (m, 1H), 7.24 – 7.16 (m, 2H), 7.04 – 6.95 (m, 1H), 2.09 (s, 3H), 1.81 (s, 3H); ^{13}C NMR (100 MHz, d_6-DMSO) δ ppm: 186.5, 154.9, 147.0, 146.5, 146.3, 142.9, 142.4, 141.9, 130.2, 129.0, 128.7, 126.4, 126.3, 123.2, 120.3, 62.0, 20.0, 16.1; HRMS (ESI) Calcd. For $C_{18}H_{16}N_3{}^+$ [M+H]$^+$ 274.1339, found 274.1344.

Di-*tert*-butyl (4-((5a*S*,6a*R*,11a*S*,13a*S*)-5,13-dioxo-2,3,5a,6,11,11a,13,13a-octahydro-1*H*-pyrrolo[1'',2'':4',5']pyrazino[1',2':1,5]pyrrolo[2,3-b]indol-6a(5*H*)-yl)-1,2-phenylene)dicarbamate **13:**

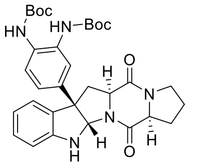

13

White solid; 150.3 mg; isolated yield = 85%; 12:1; M.p.: 88 – 89 °C; dr = 12:1; $[\alpha]_D^{20}$ = -388.3 (*c* 0.34, MeOH); ^{1}H NMR (400 MHz, d_6-DMSO) δ ppm: 8.47 – 8.43 (m, 2H), 7.48 – 7.38 (m, 2H), 7.16 – 7.09 (m, 2H), 7.03 – 6.99 (m, 1H), 6.67 – 6.63 (m, 3H), 5.55 (s, 1H), 4.30 – 4.26 (m, 1H), 4.17 – 4.13 (m, 1H), 3.40 – 3.32 (m, 2H), 3.04 – 2.99 (m, 1H), 2.58 – 2.52 (m, 1H), 2.17 – 2.11 (m, 1H), 2.00 – 1.76 (m, 3H), 1.45 (s, 18H); ^{13}C NMR (100 MHz, d_6-DMSO) δ ppm: 166.5, 165.6, 153.7, 149.8, 140.3, 132.1, 130.5, 129.3, 128.9, 124.5, 124.2, 121.9, 118.7, 110.1, 81.3, 80.0, 79.9, 60.5, 60.1, 58.6, 45.2, 40.3, 28.5, 27.8, 23.0; HRMS (ESI) Calcd. For $C_{32}H_{40}N_5O_6{}^+$ [M+H]$^+$ 590.2973, found 590.2985.

Di-*tert*-butyl (4-((5a*S*,6a*S*,11a*R*,13a*S*)-5,13-dioxo-2,3,5a,6,11,11a,13,13a-octahydro-1*H*-pyrrolo[1'',2'':4',5']pyrazino[1',2':1,5]pyrrolo[2,3-b]indol-6a(5*H*)-yl)-1,2-phenylen e)dicarbamate **13′:**

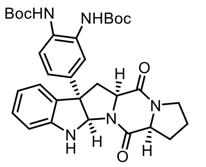

13'

White solid; 12 mg; isolated yield = 7%; M.p.: 136 – 137 °C; dr = 1:12; $[\alpha]_D^{20}$ = +89 (*c* 0.50, MeOH); ^{1}H NMR (400 MHz, $CDCl_3$) δ ppm: 7.58 – 7.39 (m, 2H), 7.13 – 7.00 (m, 4H), 6.89 (d, *J* = 7.3 Hz, 1H), 6.77 – 6.58 (m, 2H), 5.75 (s, 1H), 5.41 (s, 1H), 4.48 (t, *J* = 8.4 Hz, 1H), 4.13 (t, *J* = 8.0 Hz, 1H), 3.57 – 3.43 (m, 2H), 3.27 – 3.09 (m, 1H), 2.83 – 2.67 (m, 1H), 2.41 – 2.23 (m, 1H), 2.21 – 1.79 (m, 3H), 1.62 – 1.35 (m, 18H); ^{13}C NMR (100 MHz, $CDCl_3$) δ ppm: 167.9, 165.8, 153.9, 147.2, 139.2, 133.3, 128.6, 124.2, 123.7, 119.6, 109.7, 85.2, 81.0, 80.8, 60.5, 60.2, 59.5, 45.2, 38.3, 28.3, 27.6, 23.3; HRMS (ESI) Calcd. For $C_{32}H_{40}N_5O_6^+$ $[M+H]^+$ 590.2973, found 590.2985.

(5a*S*,6a*R*,11a*S*,13a*S*)-6a-(3,4-diaminophenyl)-1,2,3,6,6a,11,11a,13a-octahydro-13*H*-pyrrolo[1",2":4',5']pyrazino[1',2':1,5]pyrrolo[2,3-b]indole-5,13(5a*H*)-dione **14:**

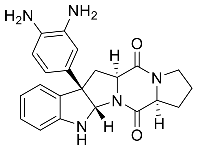

14

White solid; 37.4 mg; isolated yield = 96%; M.p.: 79 – 80 °C; $[\alpha]_D^{20}$ = -159.0 (*c* 0.2, MeOH); ^{1}H NMR (400 MHz, d_6-DMSO) δ ppm: 7.09 – 7.04 (m, 2H), 6.77 – 6.73 (m, 1H), 6.64 – 6.53 (m, 4H), 5.53 (s, 1H), 5.35 (s, 1H), 4.13 – 4.00 (m, 2H), 3.54 – 3.46 (m, 2H), 3.18 (s, 4H), 3.00 – 2.95 (m, 1H), 2.81 – 2.75 (m, 1H), 2.31 – 2.24 (m, 1H), 2.14 – 1.96 (m, 2H), 1.91 – 1.81 (m, 1H); ^{13}C NMR (100 MHz, d_6-DMSO) δ ppm: 167.0, 165.9, 148.6, 134.9, 134.3, 133.8, 132.2, 128.7, 124.1, 119.8, 117.1, 116.6, 114.1, 110.1, 82.2, 60.8, 60.5, 58.7, 45.2, 39.0, 27.8, 23.1; HRMS (ESI) Calcd. For $C_{22}H_{24}N_5O_2^+$ $[M+H]^+$ 390.1925, found 390.1928.

(5a*S*,6a*R*,11a*S*,13a*S*)-6a-(1*H*-benzo[*d*]imidazol-5-yl)-1,2,3,6,6a,11,11a,13a-octahydro-13*H*-pyrrolo[1",2":4',5']pyrazino[1',2':1,5]pyrrolo[2,3-*b*]indole-5,13(5a*H*)-dione **15:**

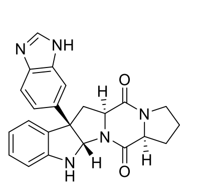

15

White solid; 31.2 mg; isolated yield = 78%; M.p.: 67 – 77 °C; $[\alpha]_D^{20}$ = -572.0 (*c* 0.15, MeOH); ^{1}H NMR (400 MHz, d_6-DMSO) δ ppm: 12.4 (s, 1H), 8.18 (s, 1H), 7.66 – 7.51 (m, 2H), 7.38 – 7.14 (m, 2H), 7.03 – 7.00 (m, 1H), 6.69 – 6.64 (m, 3H), 5.60 (s, 1H), 4.42 – 4.28 (m, 1H), 4.25 – 4.16 (m, 1H), 3.51 – 3.45 (m, 2H), 3.13 – 3.08 (m, 1H), 3.78 – 2.61 (m, 1H), 2.20 – 2.12 (m, 1H), 2.01 – 1.74 (m, 3H); ^{13}C NMR (100 MHz, d_6-DMSO) δ ppm: 166.5, 165.7, 149.9, 142.9, 138.3, 132.7, 128.8, 124.5, 120.2, 118.7,

110.1, 81.9, 60.7, 60.1, 59.2, 45.2, 40.4, 27.8, 23.0; HRMS (ESI) Calcd. For $C_{23}H_{22}N_5O_2^+$ $[M+H]^+$ 400.1768, found 400.1771.

4. Crystallographic data collections for compounds 3g' & 5g

4.1 X-ray single crystal data for compound 3g':

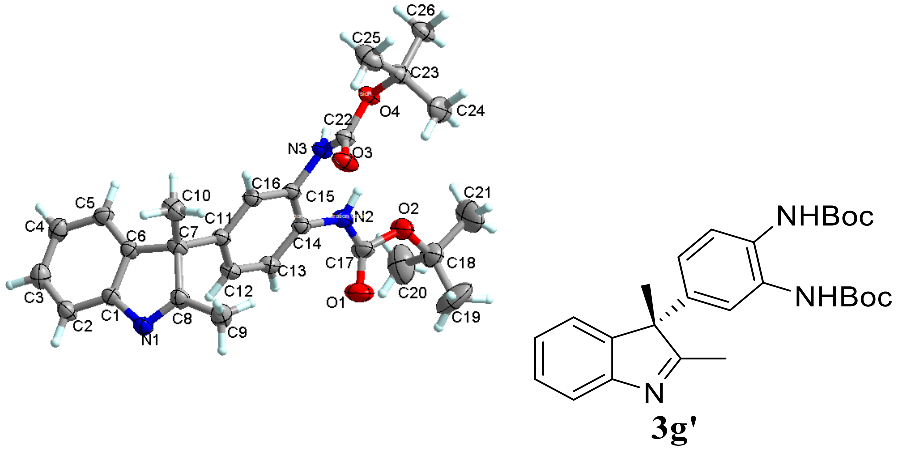

Figure S1. X ray structure of **3g'** (CCDC 2268394)

Table S1. Crystal data and structure refinement for cu_20230415_Zh_137566_0m:

Identification code	cu_20230415_Zh_137566_0m
Empirical formula	$C_{26}H_{33}N_3O_4$
Formula weight	451.55
Temperature/K	299.00
Crystal system	Trigonal
Space group	$P3_2$
a/Å	14.8240(3)
b/Å	14.8240(3)
c/Å	11.1410(4)
α/°	90
β/°	90
γ/°	120
Volume/Å^3	2120.24(11)
Z	3
ρ_{calc}g/cm^3	1.061
μ/mm^{-1}	0.579
F(000)	726.0
Crystal size/mm^3	0.13 × 0.12 × 0.1
Radiation	CuKα (λ = 1.54178)
2Θ range for data collection/°	6.886 to 159.138
Index ranges	$-18 \leq h \leq 18$, $-17 \leq k \leq 18$, $-12 \leq l \leq 14$
Reflections collected	35919
Independent reflections	5915 [R_{int} = 0.0655, R_{sigma} = 0.0361]
Data/restraints/parameters	5915/20/318
Goodness-of-fit on F^2	1.047
Final R indexes [I>=2σ (I)]	R_1 = 0.0459, wR_2 = 0.1277
Final R indexes [all data]	R_1 = 0.0543, wR_2 = 0.1357
Largest diff. peak/hole / e Å^{-3}	0.14/-0.15
Flack parameter	-0.1(3)

4.2 X-ray single crystal data for compound 5g:

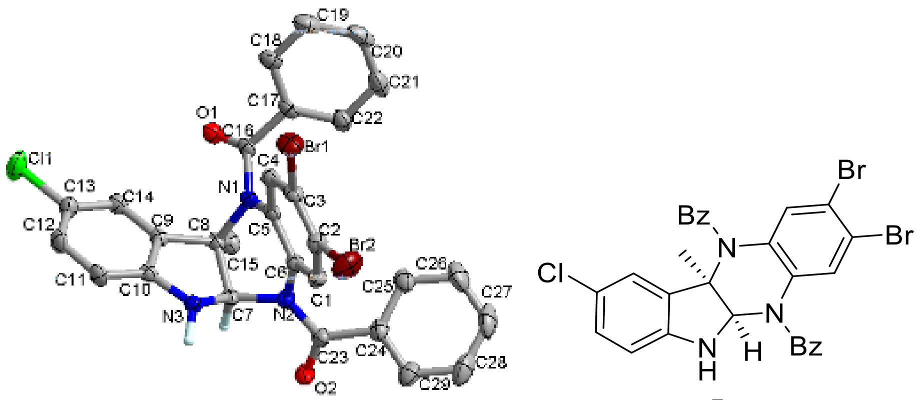

5g

Figure S2. X ray structure of **5g** (CCDC2268391)

Table S2. Crystal data and structure refinement for 202305105_auto.

Identification code	202305105_auto
Empirical formula	$C_{29}H_{20}Br_2ClN_3O_2$
Formula weight	637.75
Temperature/K	293(2)
Crystal system	monoclinic
Space group	$P2_1$
a/Å	12.3249(5)
b/Å	8.3514(4)
c/Å	14.0345(4)
α/°	90
β/°	109.247(4)
γ/°	90
Volume/Å^3	1363.84(10)
Z	2
ρ_{calc}g/cm^3	1.553
μ/mm^{-1}	4.925
F(000)	636.0
Crystal size/mm^3	0.14 × 0.09 × 0.07
Radiation	CuKα (λ = 1.54184)
2Θ range for data collection/°	6.67 to 141.604
Index ranges	$-12 \leq h \leq 14$, $-9 \leq k \leq 7$, $-16 \leq l \leq 17$
Reflections collected	10531
Independent reflections	4433 [R_{int} = 0.0377, R_{sigma} = 0.0490]
Data/restraints/parameters	4433/1/339
Goodness-of-fit on F^2	1.034
Final R indexes [I>=2σ (I)]	R_1 = 0.0453, wR_2 = 0.1176
Final R indexes [all data]	R_1 = 0.0491, wR_2 = 0.1217
Largest diff. peak/hole / e Å^{-3}	0.56/-0.39
Flack parameter	-0.04(3)

5. NMR Spectra of compounds

^{1}H NMR spectrum of compound **3a** ($CDCl_3$, 400 MHz)

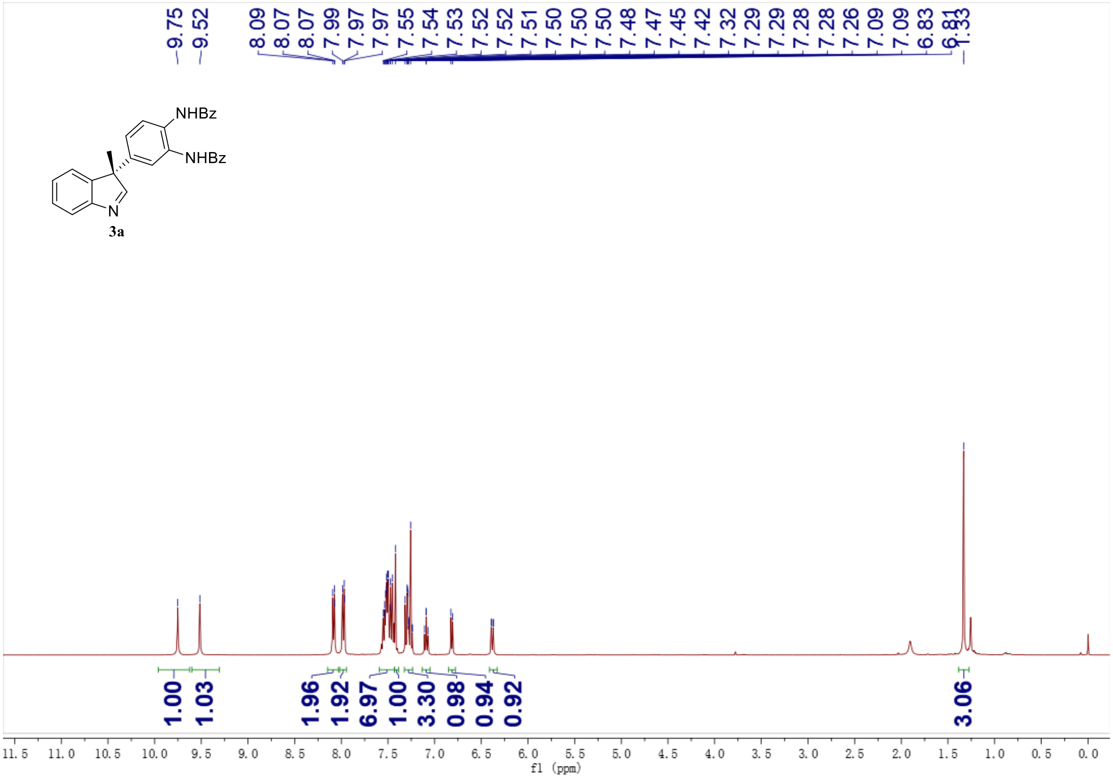

^{13}C NMR spectrum of compound **3a** ($CDCl_3$, 100 MHz)

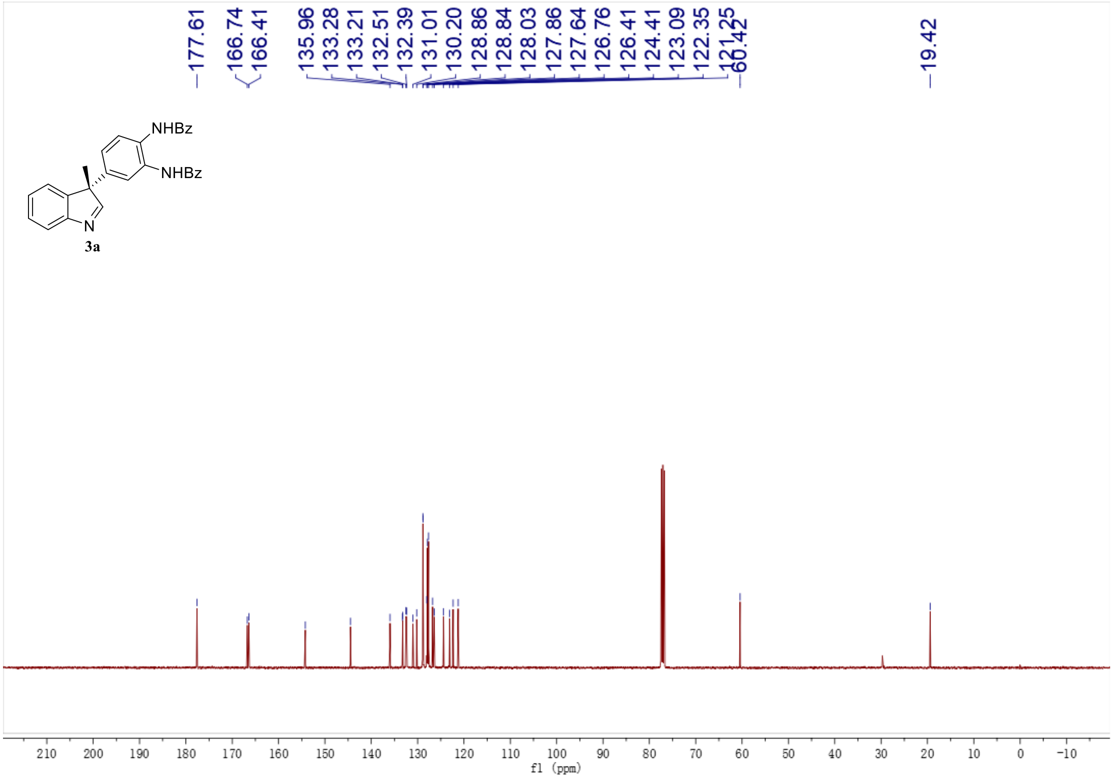

^{1}H NMR spectrum of compound **3b** ($(CD_3)_2SO$, 400 MHz)

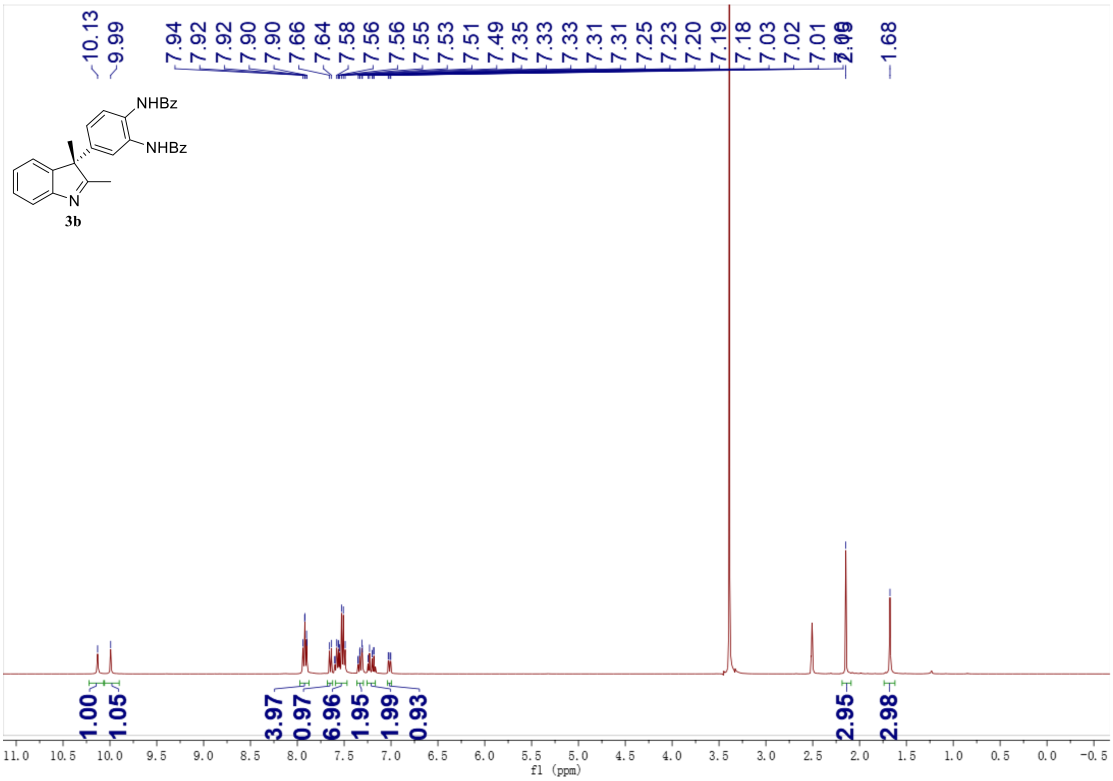

^{13}C NMR spectrum of compound **3b** ($(CD_3)_2SO$, 100 MHz)

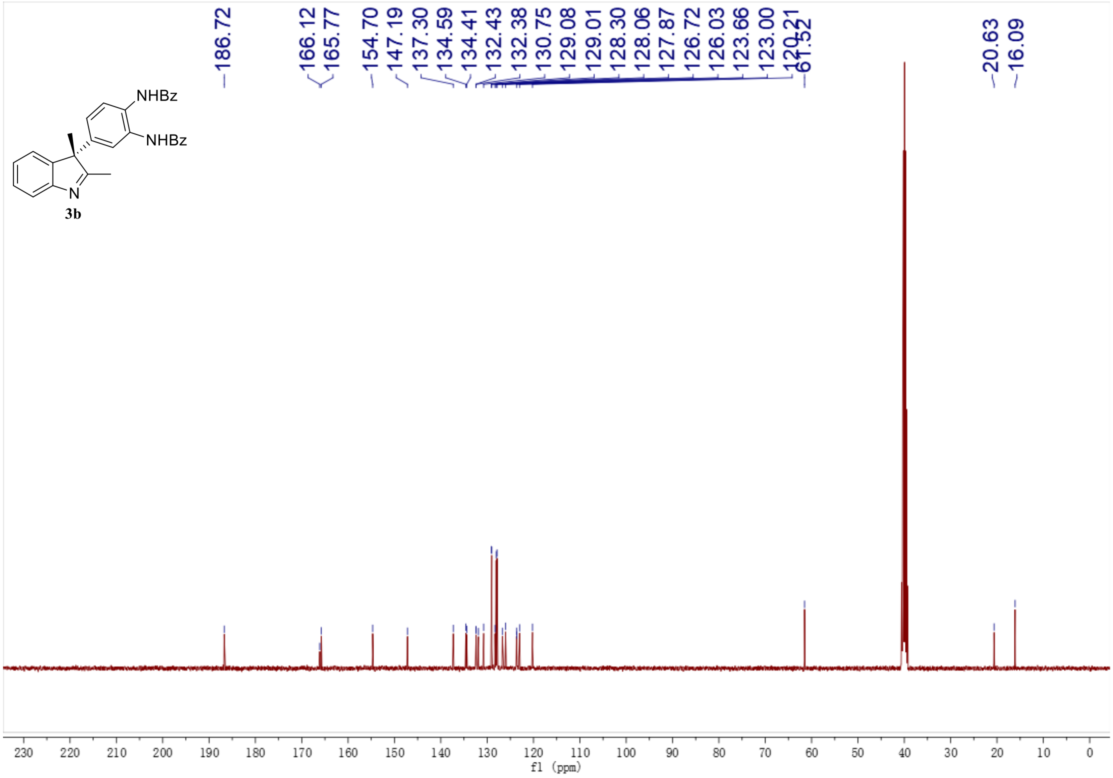

^{1}H NMR spectrum of compound **3c** ($(CD_3)_2SO$, 400 MHz)

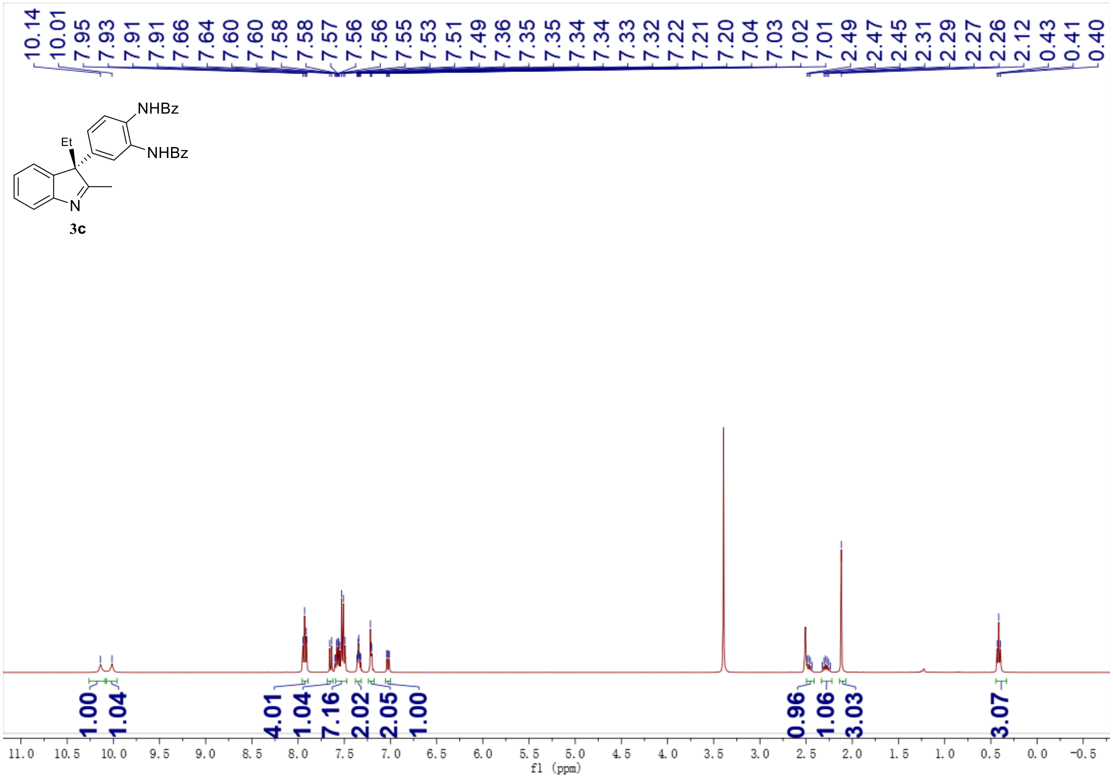

^{13}C NMR spectrum of compound **3c** ($(CD_3)_2SO$, 100 MHz)

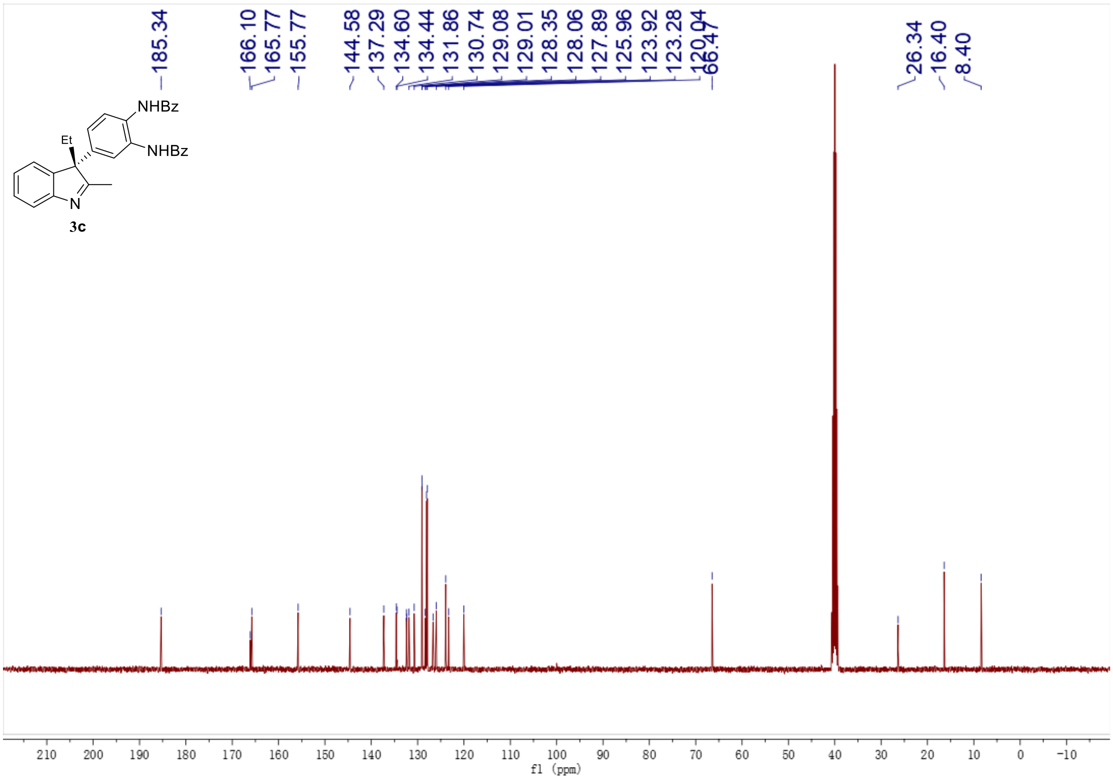

^{1}H NMR spectrum of compound **3d** ($(CD_3)_2SO$, 400 MHz)

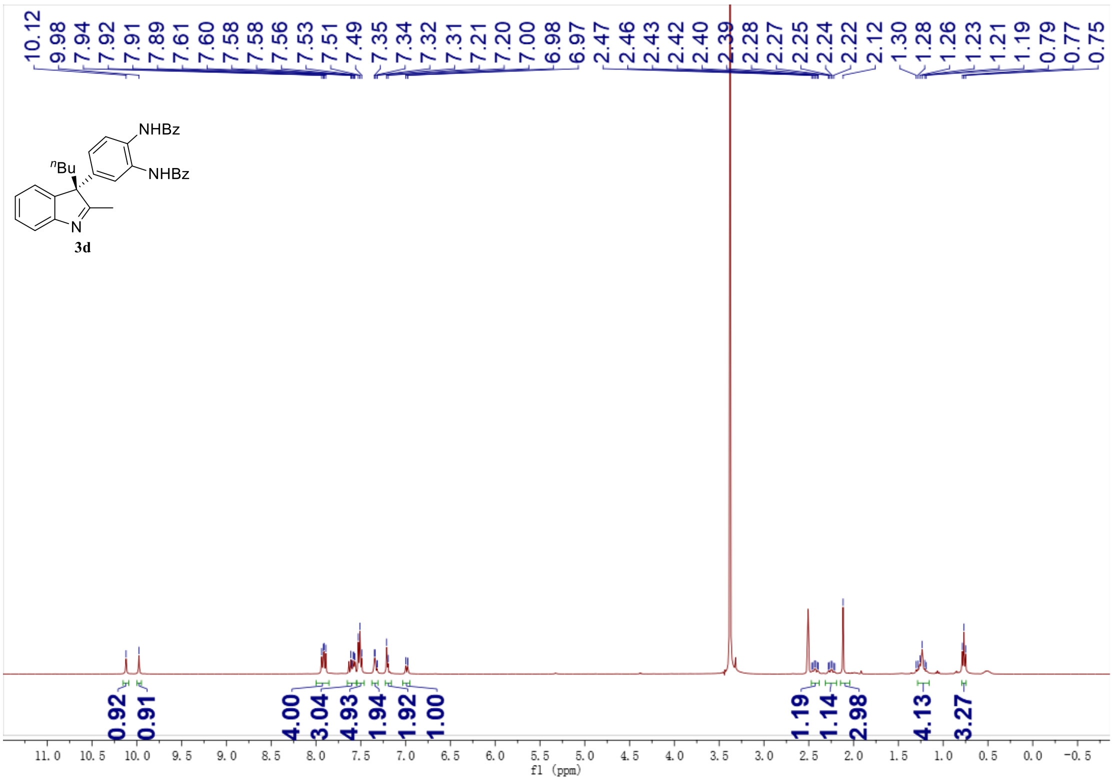

^{13}C NMR spectrum of compound **3d** ($(CD_3)_2SO$, 100 MHz)

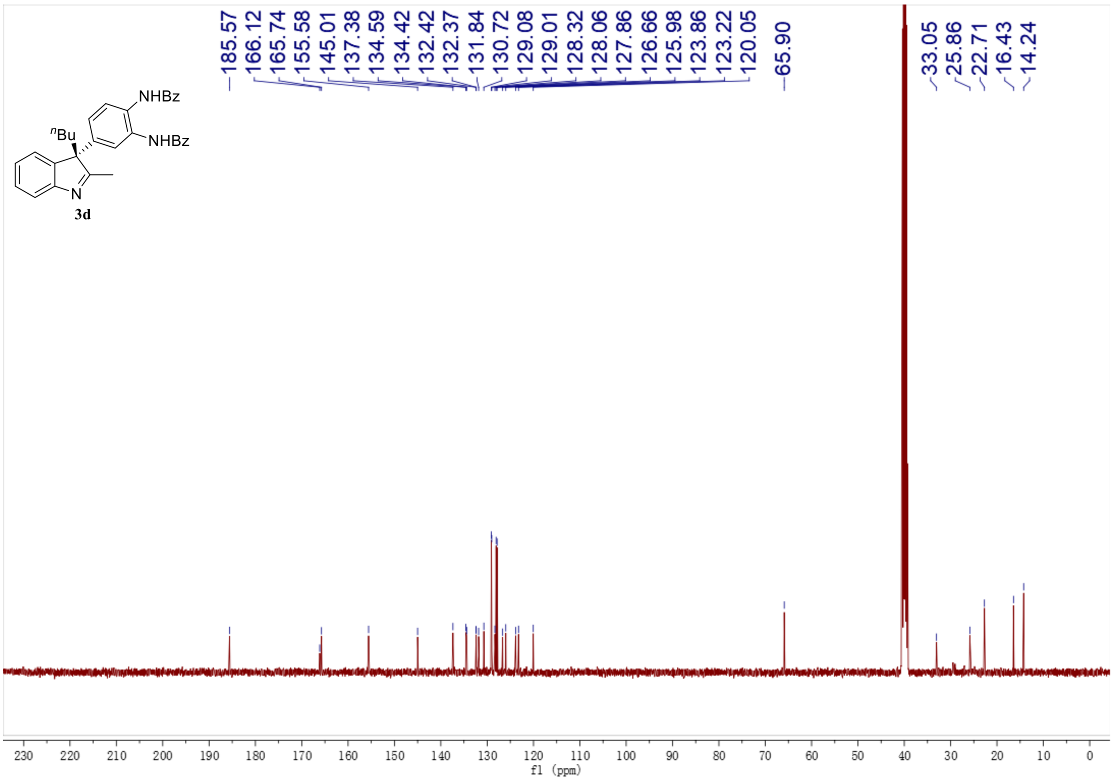

^{1}H NMR spectrum of compound **3e** ($CDCl_3$, 400 MHz)

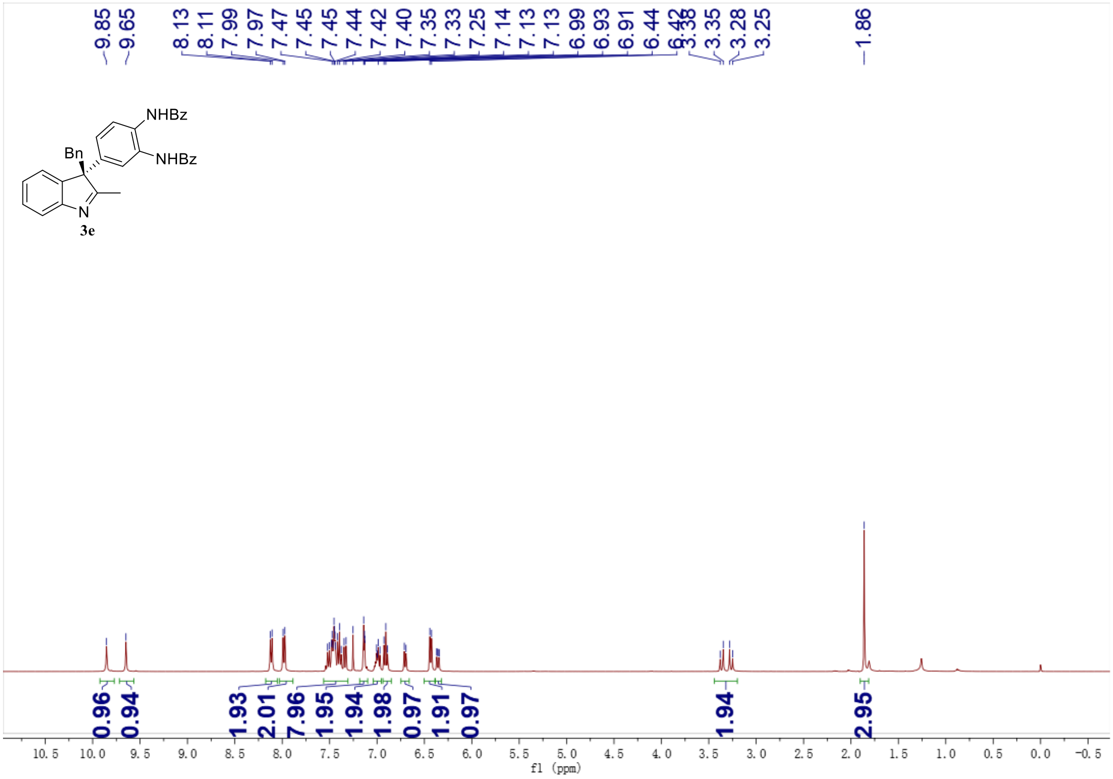

^{13}C NMR spectrum of compound **3e** ($CDCl_3$, 100 MHz)

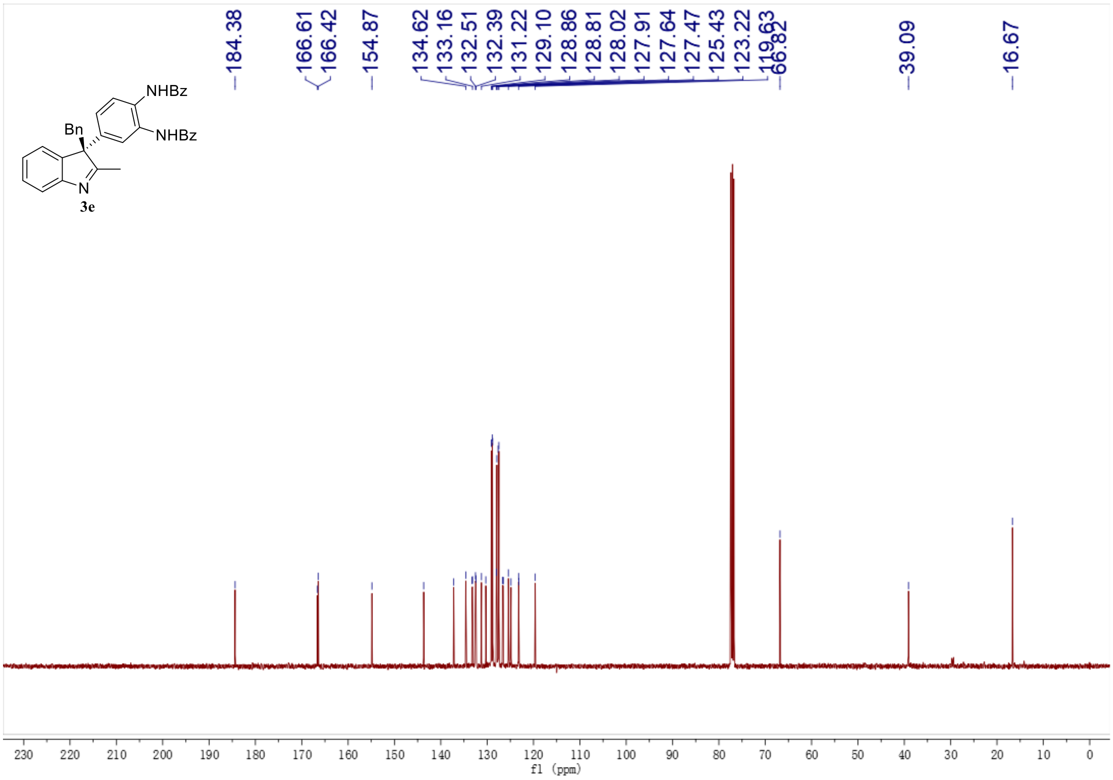

^{1}H NMR spectrum of compound **3f** ($(CD_3)_2SO$, 400 MHz)

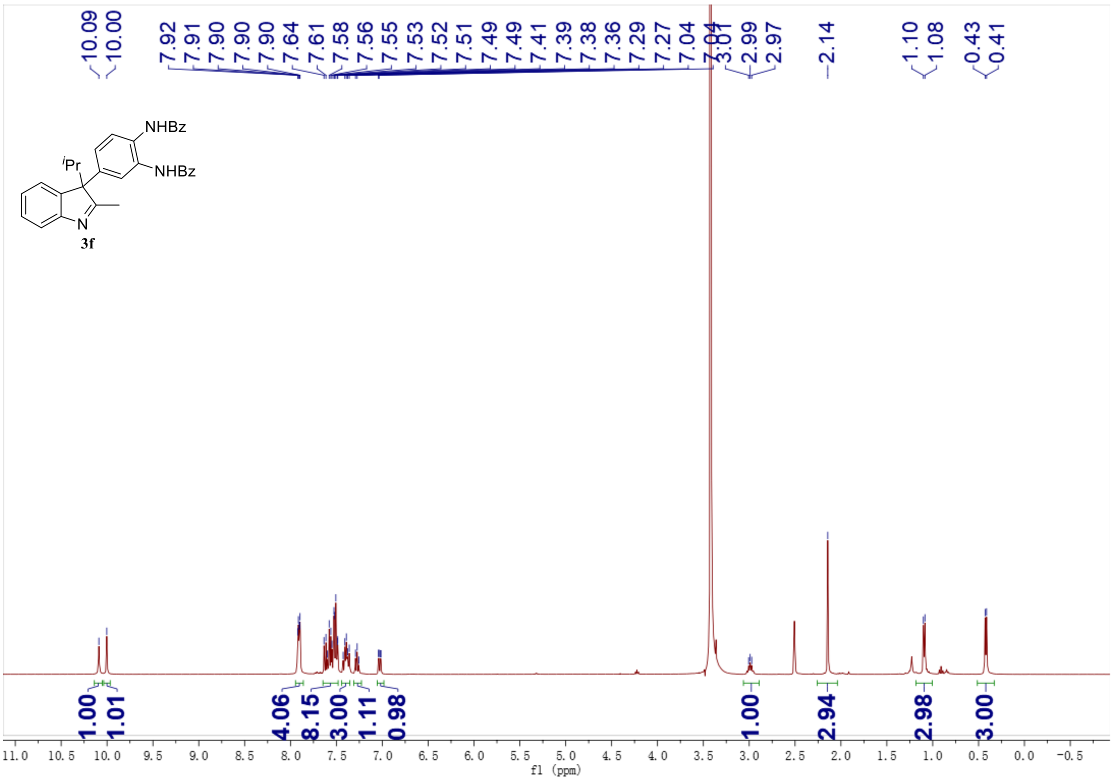

^{13}C NMR spectrum of compound **3f** ($(CD_3)_2SO$, 100 MHz)

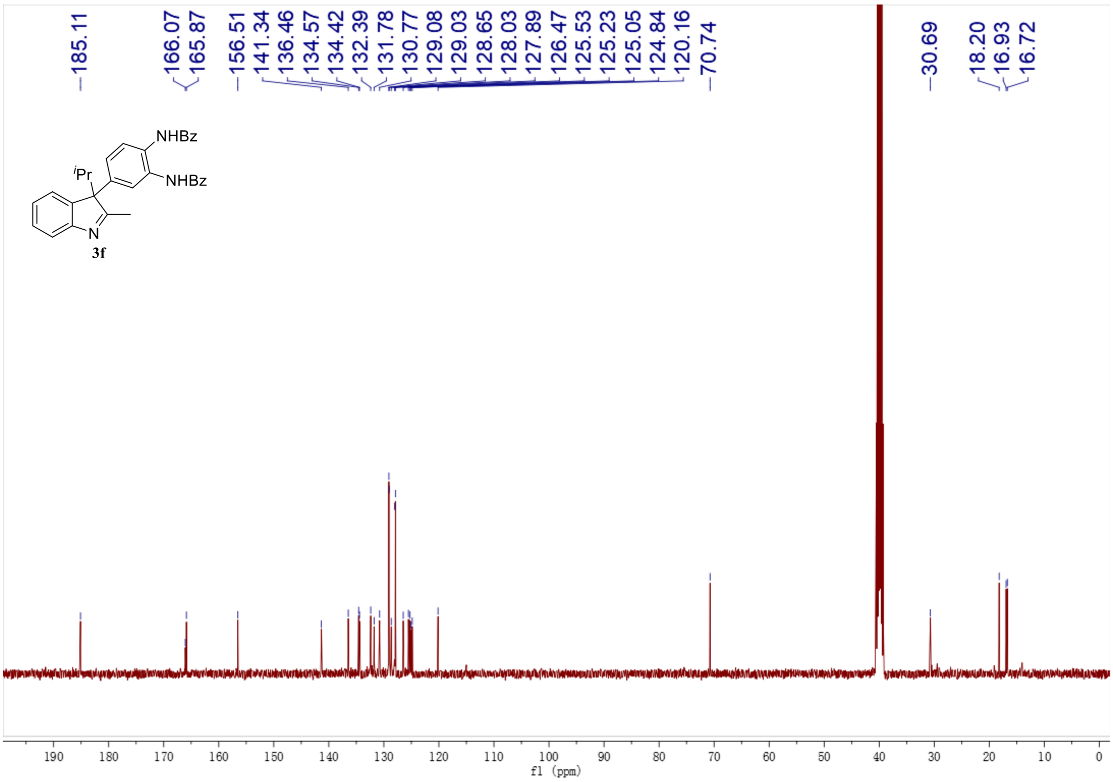

^{1}H NMR spectrum of compound **3g** ($(CD_3)_2SO$, 400 MHz)

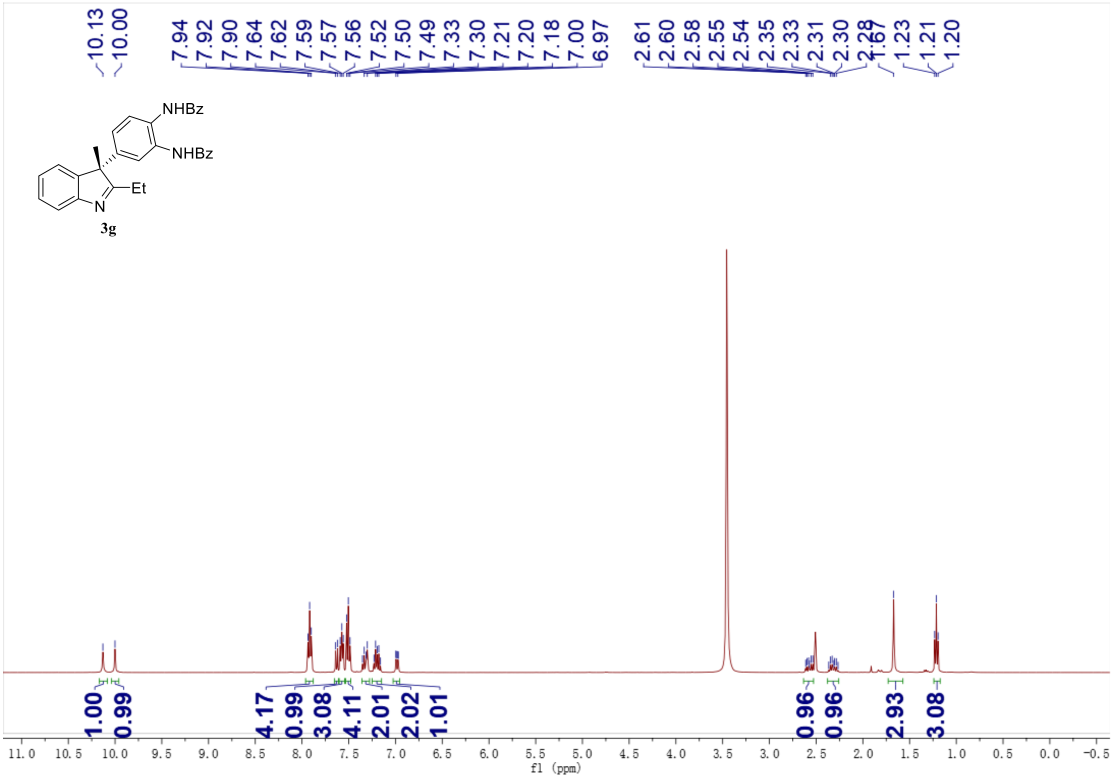

^{13}C NMR spectrum of compound **3g** ($(CD_3)_2SO$, 100 MHz)

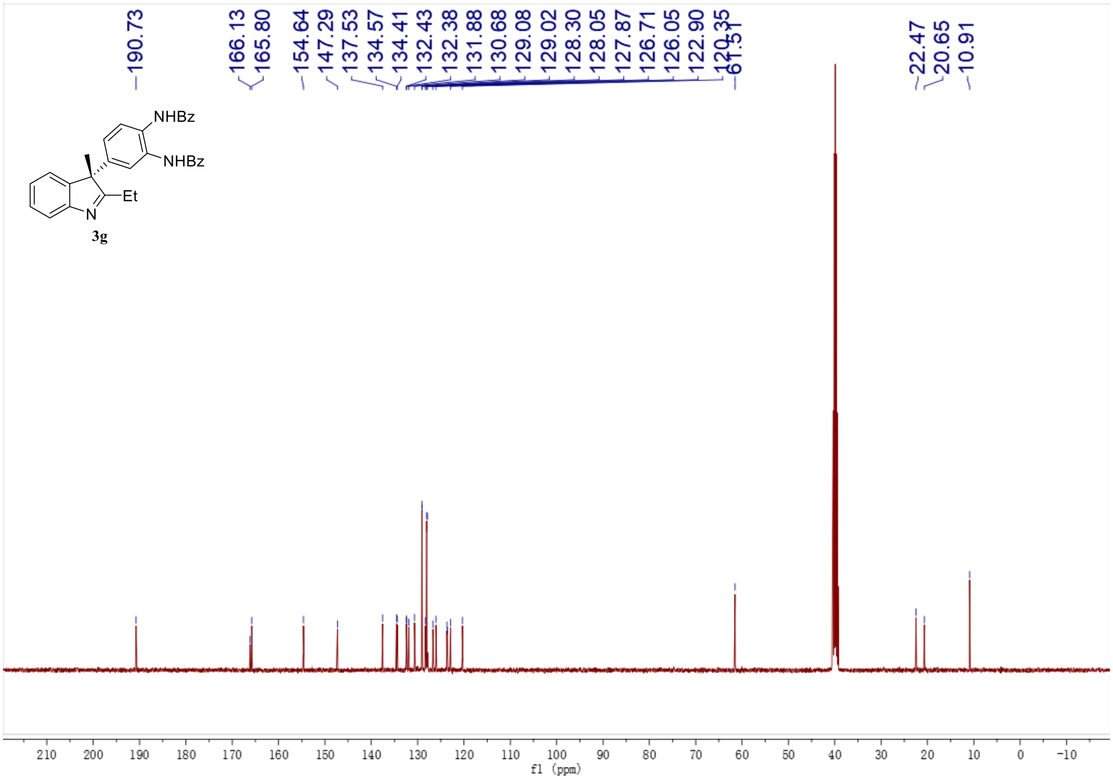

^{1}H NMR spectrum of compound **3h** ($(CD_3)_2SO$, 400 MHz)

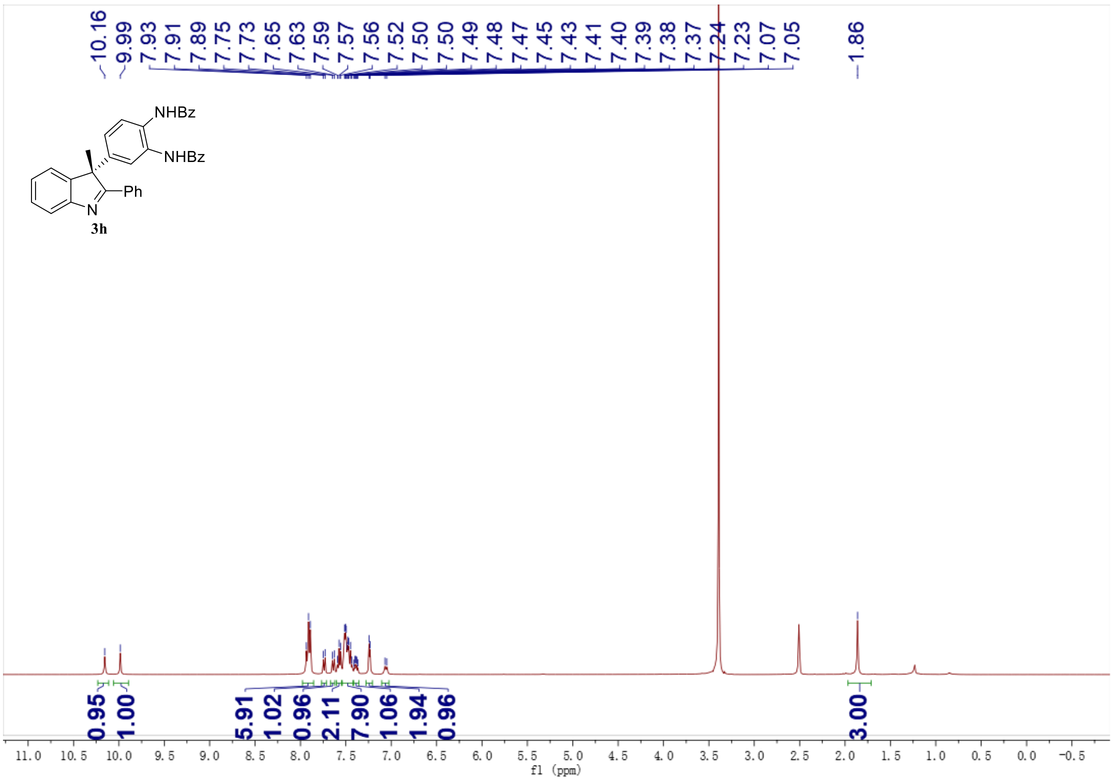

^{13}C NMR spectrum of compound **3h** ($(CD_3)_2SO$, 100 MHz)

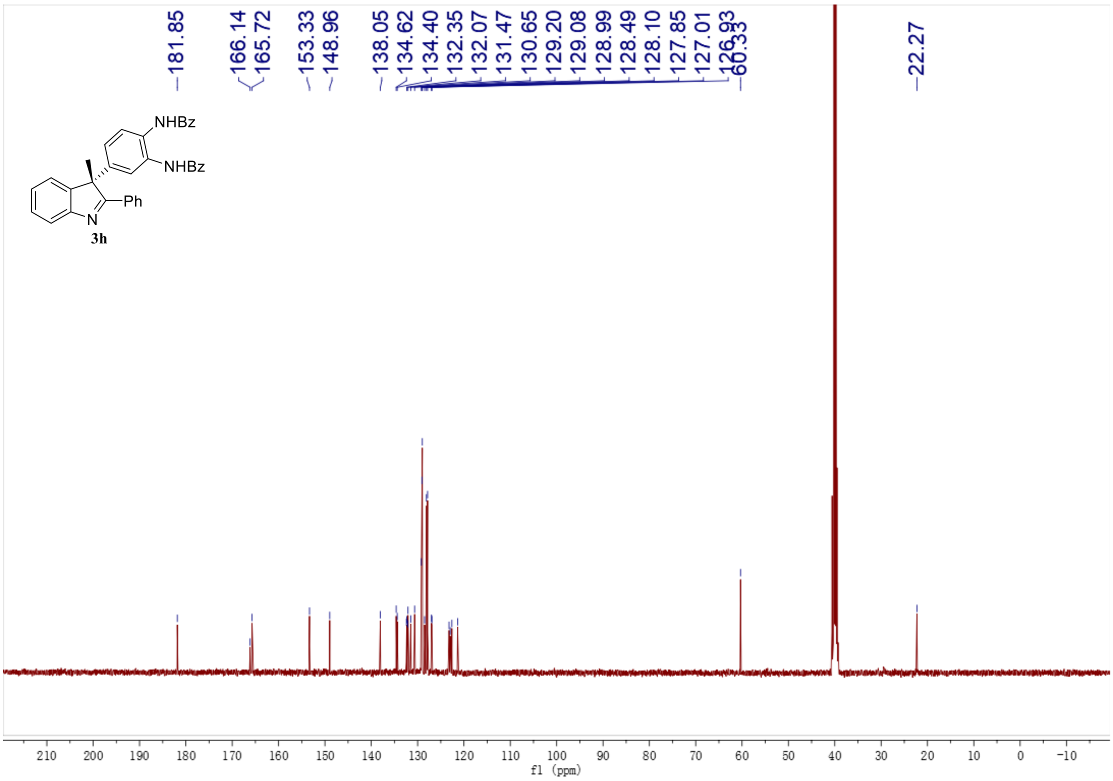

^{1}H NMR spectrum of compound **3i** ((CD$_3$)$_2$SO, 400 MHz)

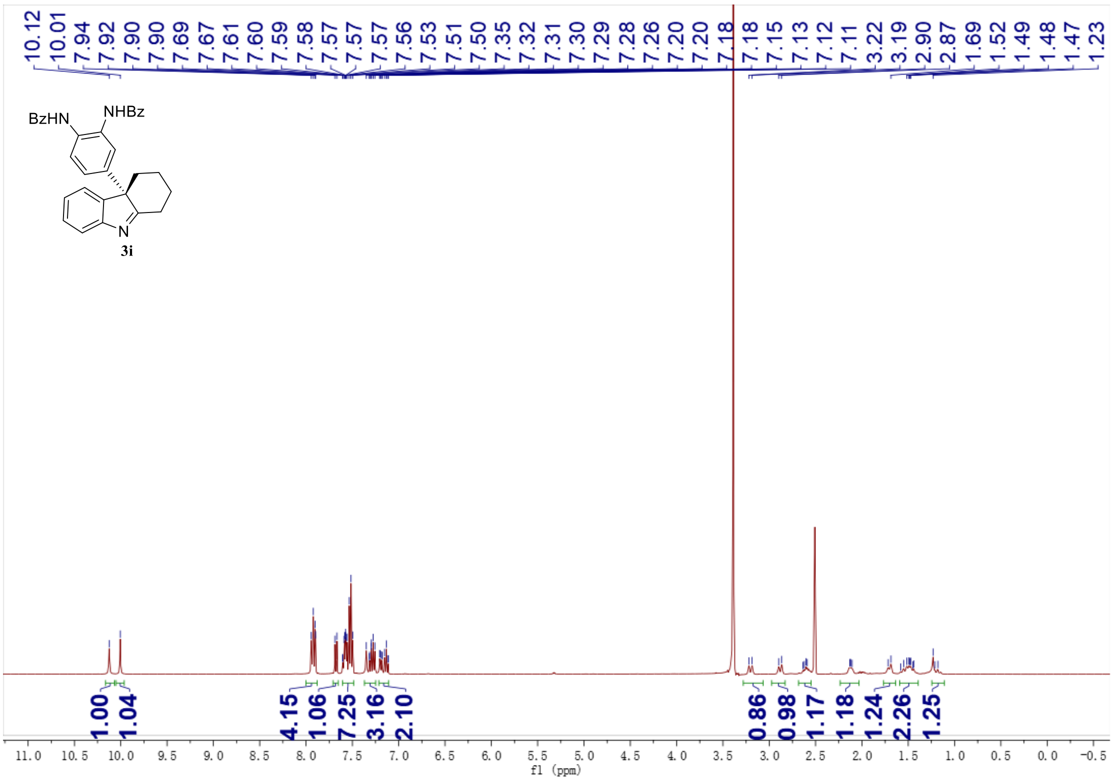

^{13}C NMR spectrum of compound **3i** ((CD$_3$)$_2$SO, 100 MHz)

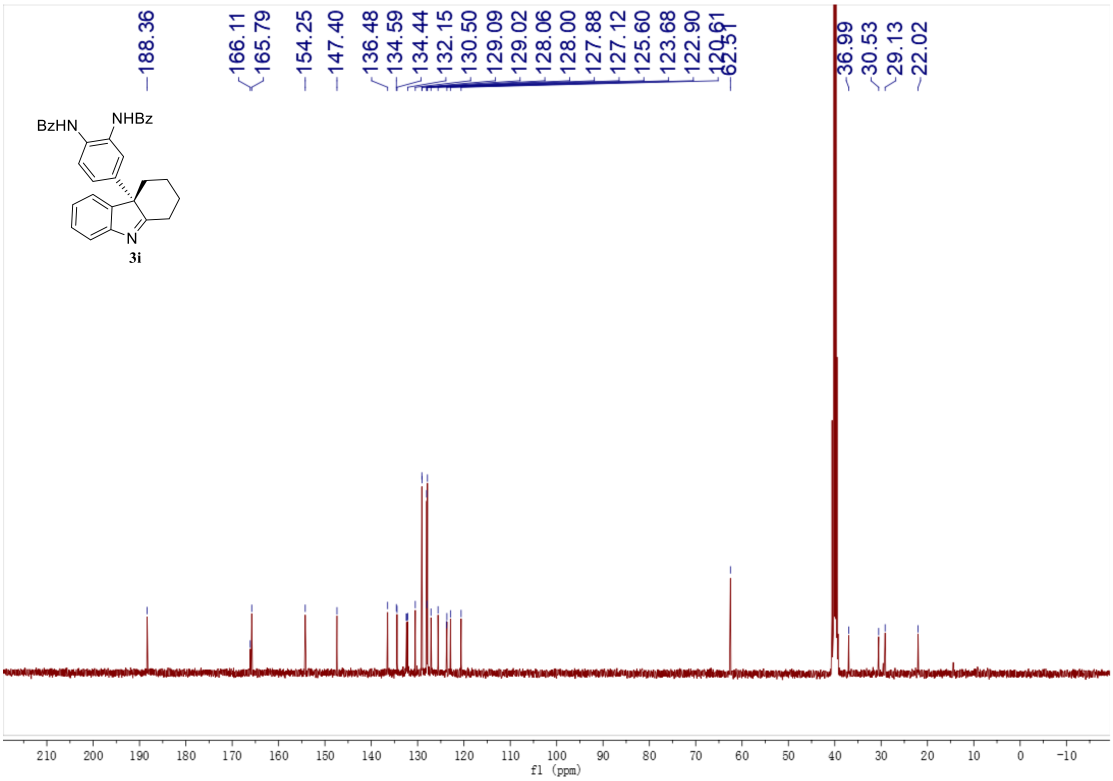

^{1}H NMR spectrum of compound **3j** ((CD$_3$)$_2$SO, 400 MHz)

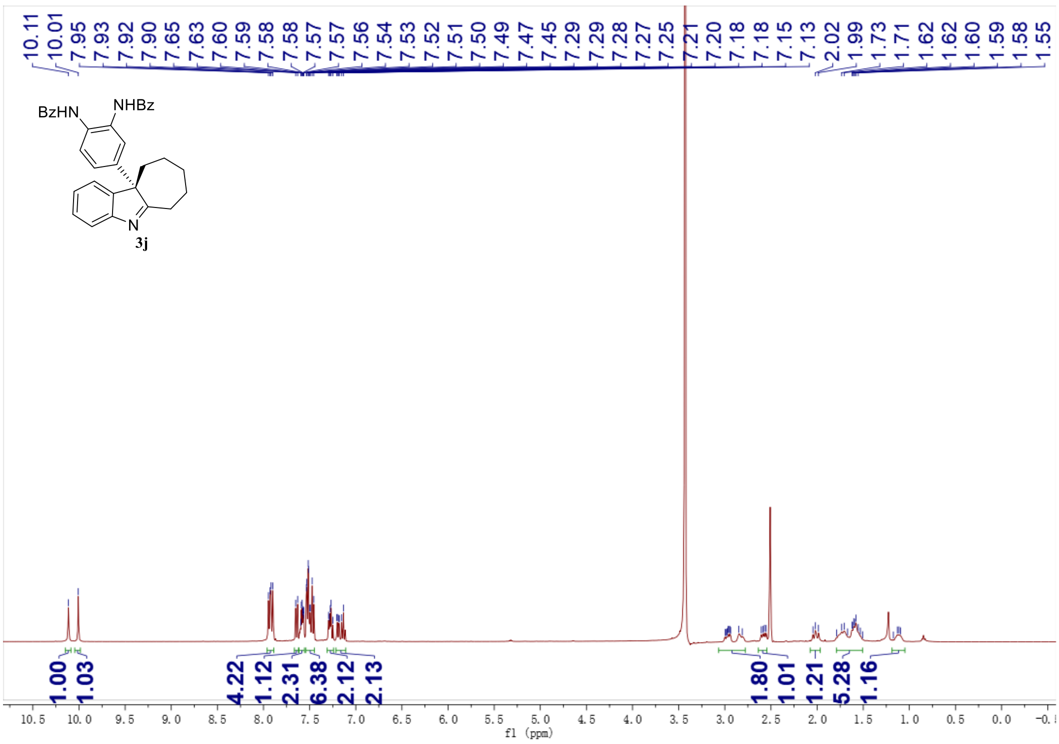

^{13}C NMR spectrum of compound **3j** ((CD$_3$)$_2$SO, 100 MHz)

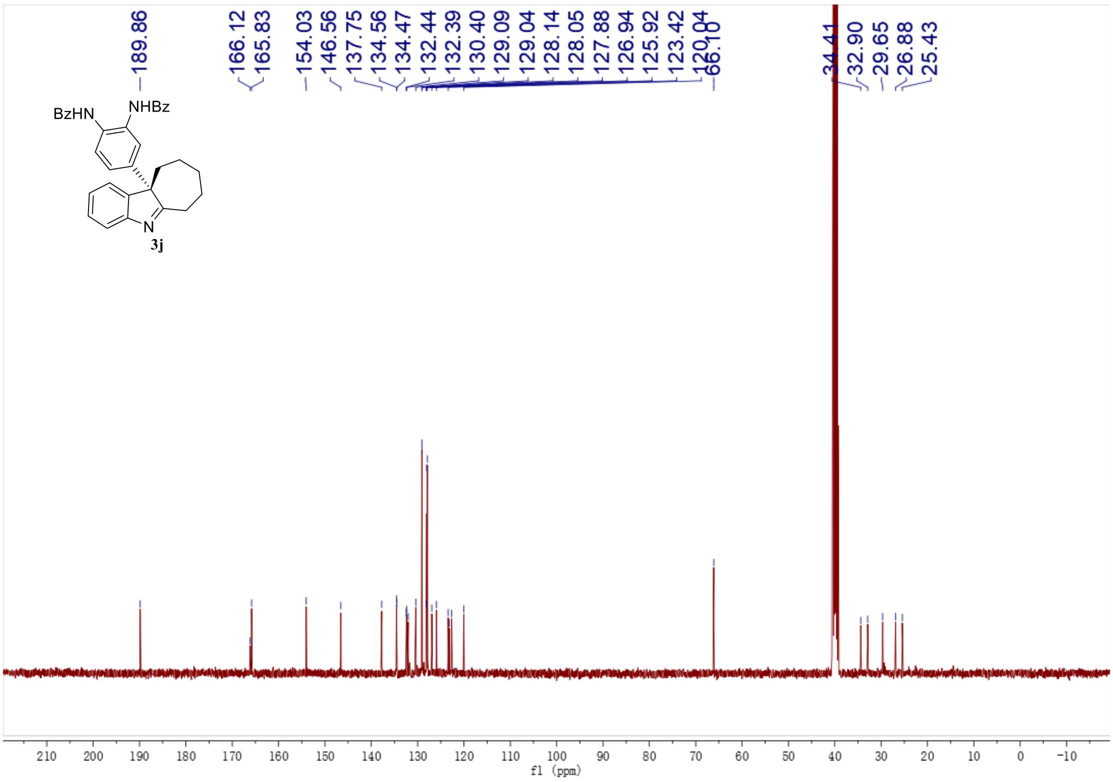

^{1}H NMR spectrum of compound **3k** ($(CD_3)_2SO$, 400 MHz)

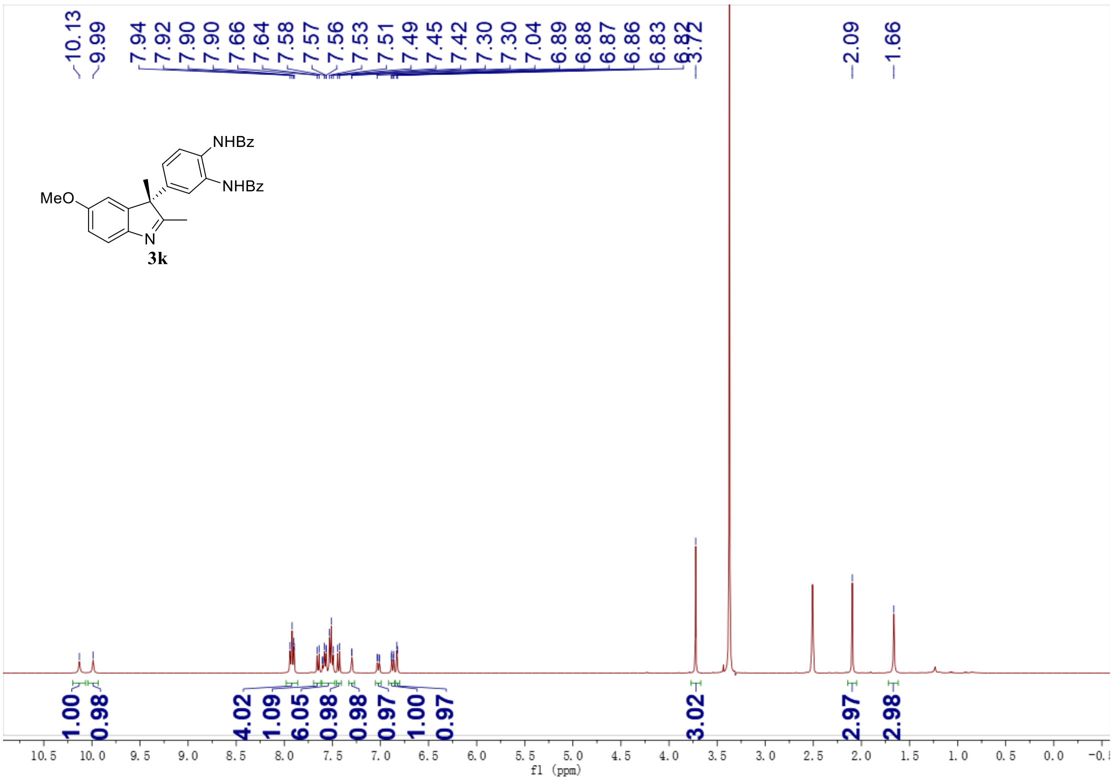

^{13}C NMR spectrum of compound **3k** ($(CD_3)_2SO$, 100 MHz)

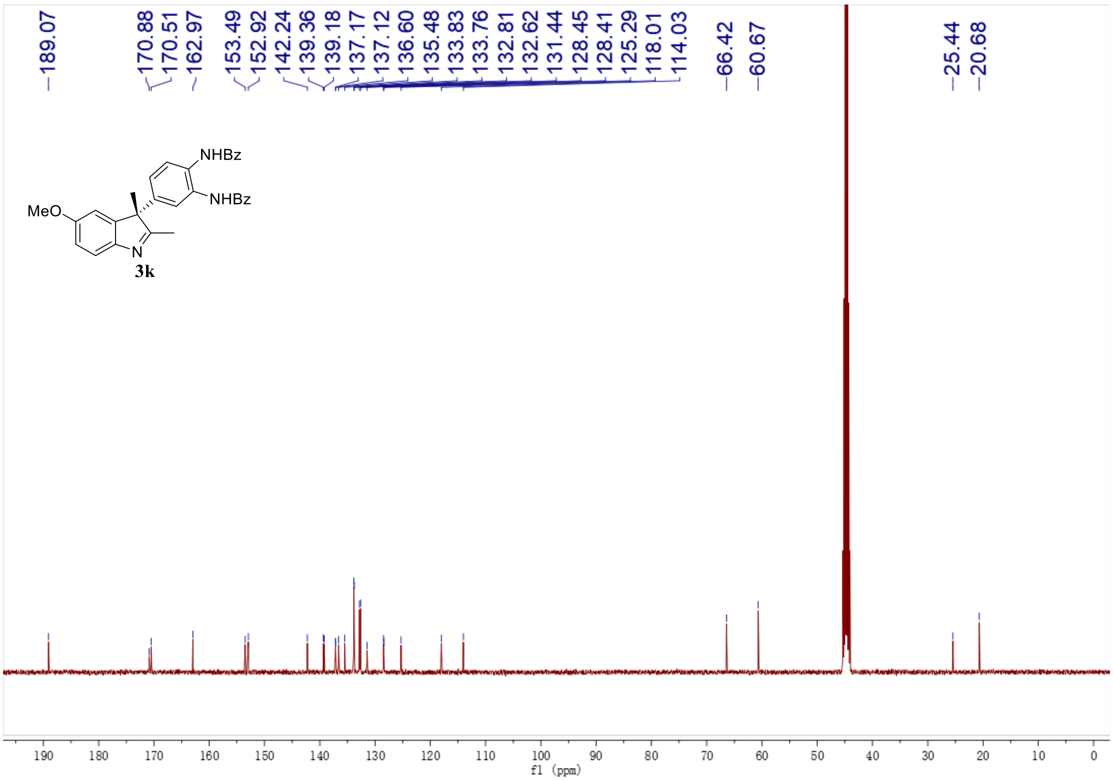

^{1}H NMR spectrum of compound **3l** ($(CD_3)_2SO$, 400 MHz)

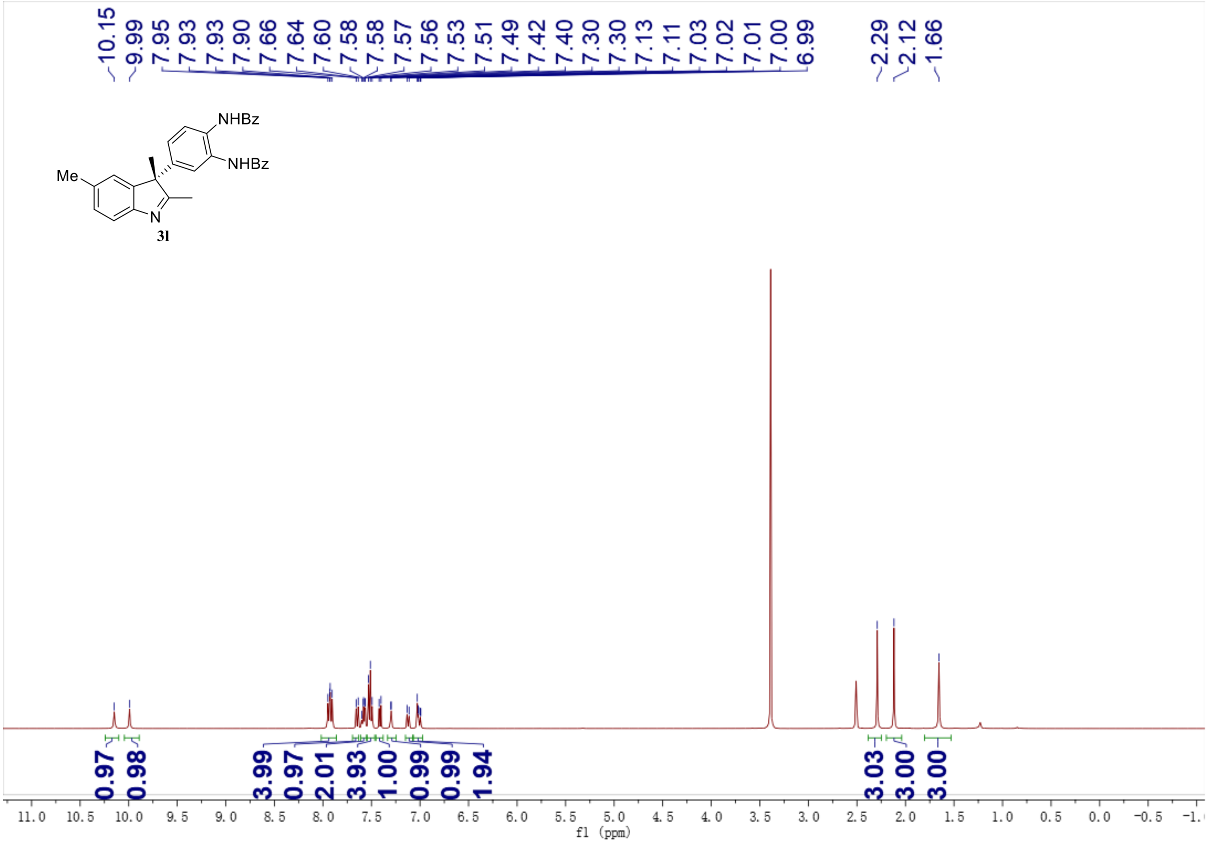

^{13}C NMR spectrum of compound **3l** ($(CD_3)_2SO$, 100 MHz)

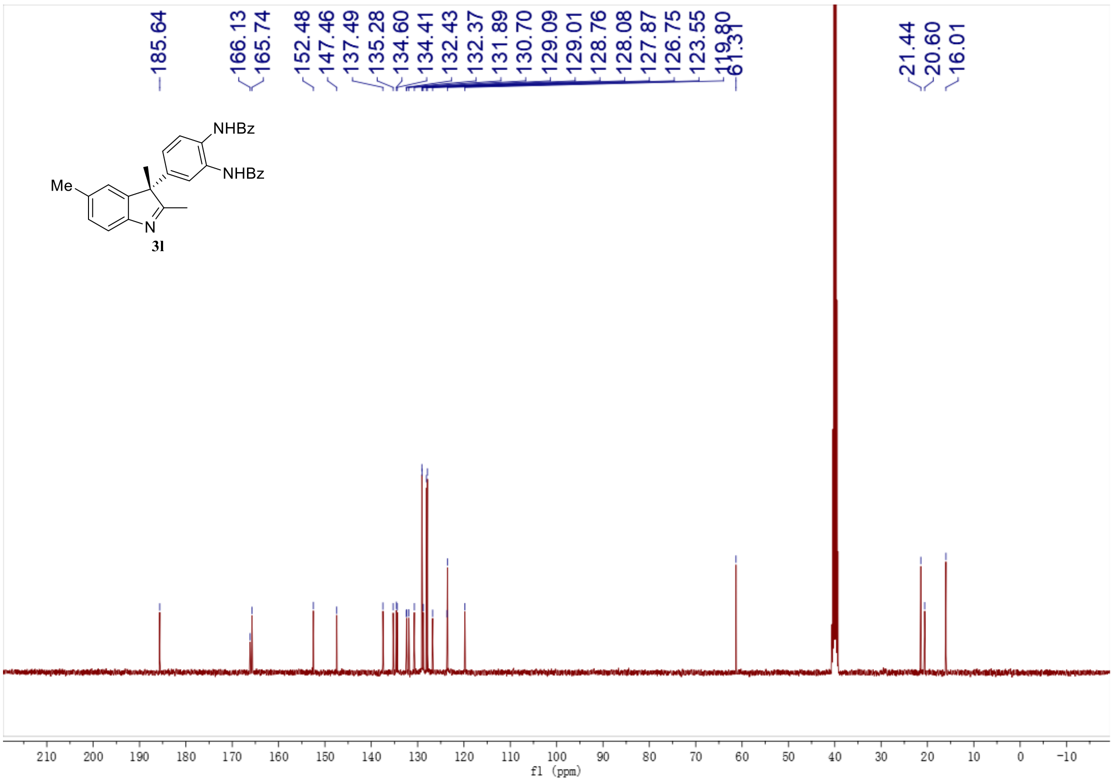

^{1}H NMR spectrum of compound **3m** ($(CD_3)_2SO$, 400 MHz)

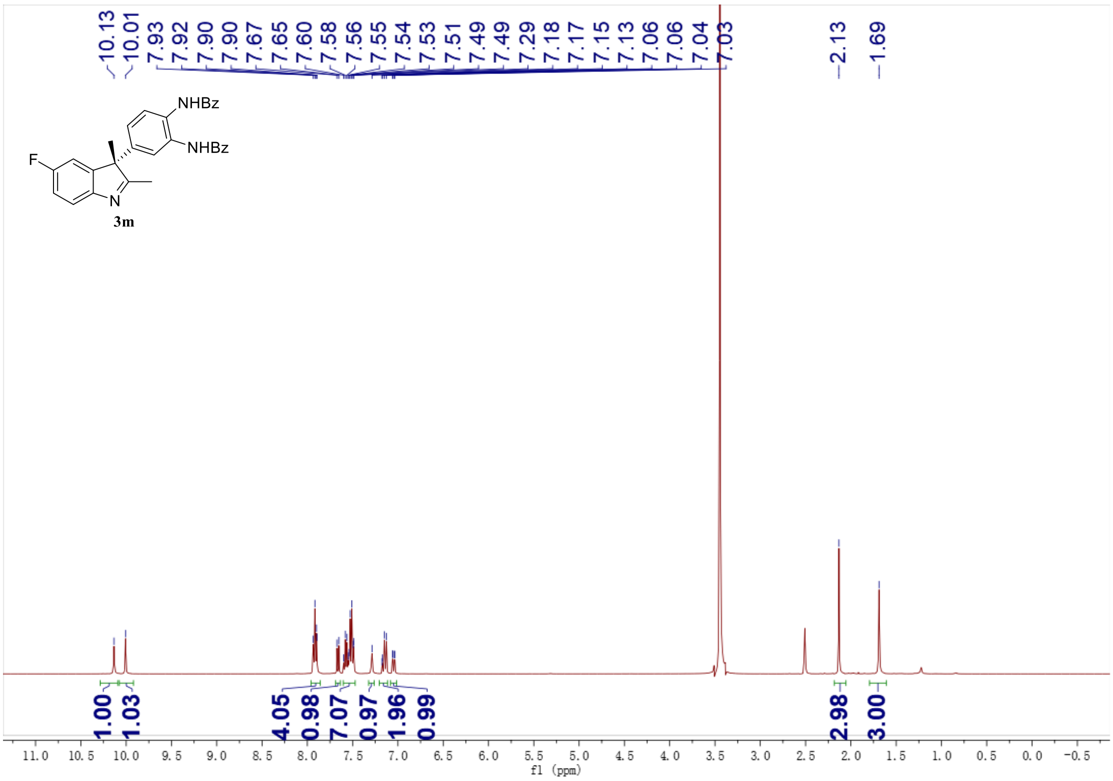

^{13}C NMR spectrum of compound **3m** ($(CD_3)_2SO$, 100 MHz)

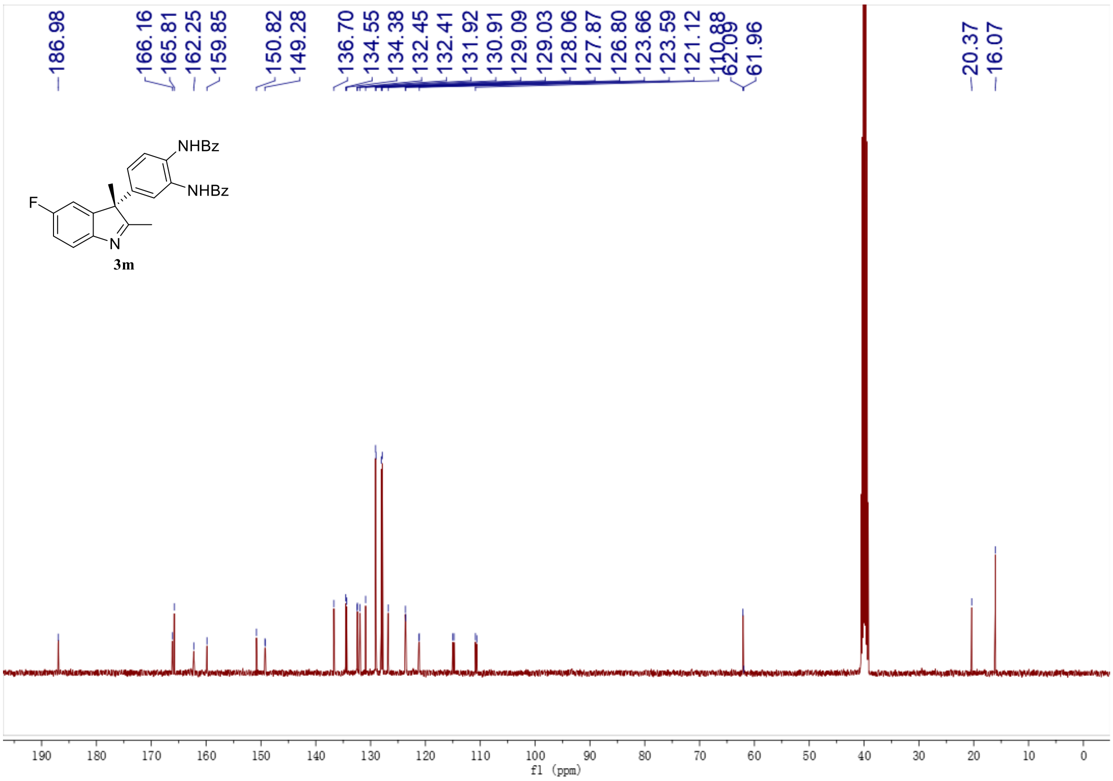

^{19}F NMR spectrum of compound **3m** ($(CD_3)_2SO$, 376 MHz)

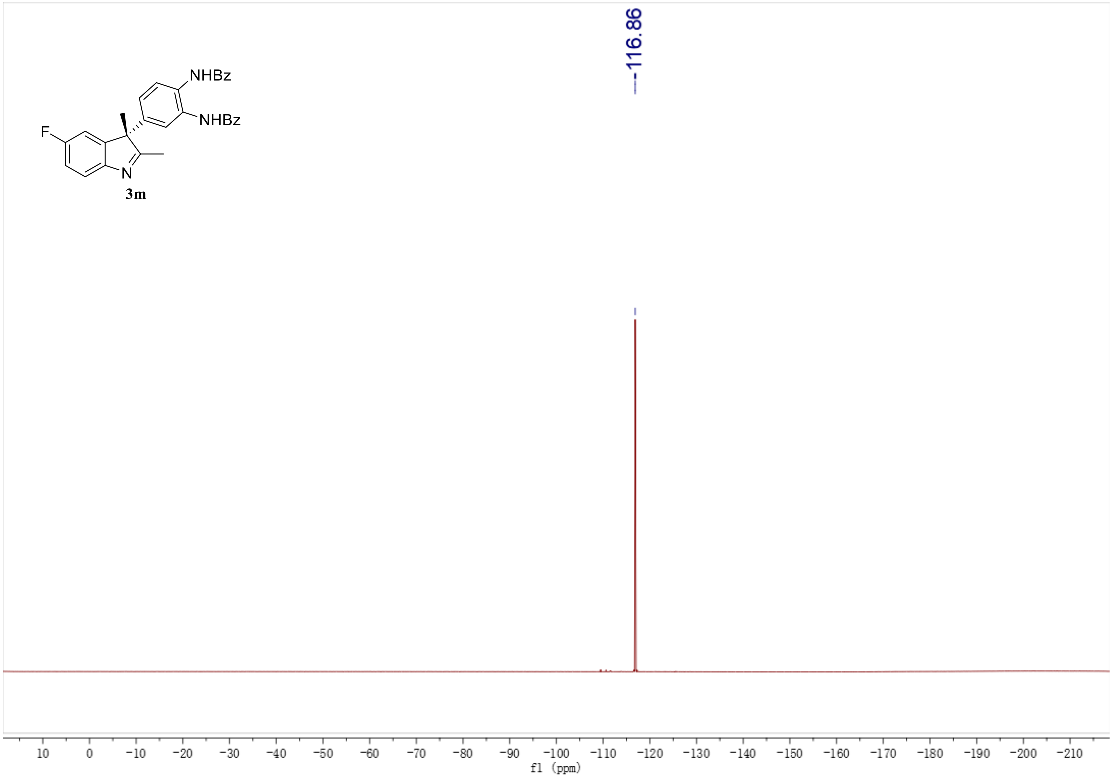

^{1}H NMR spectrum of compound **3n** (($(CD_3)_2SO$, 400 MHz)

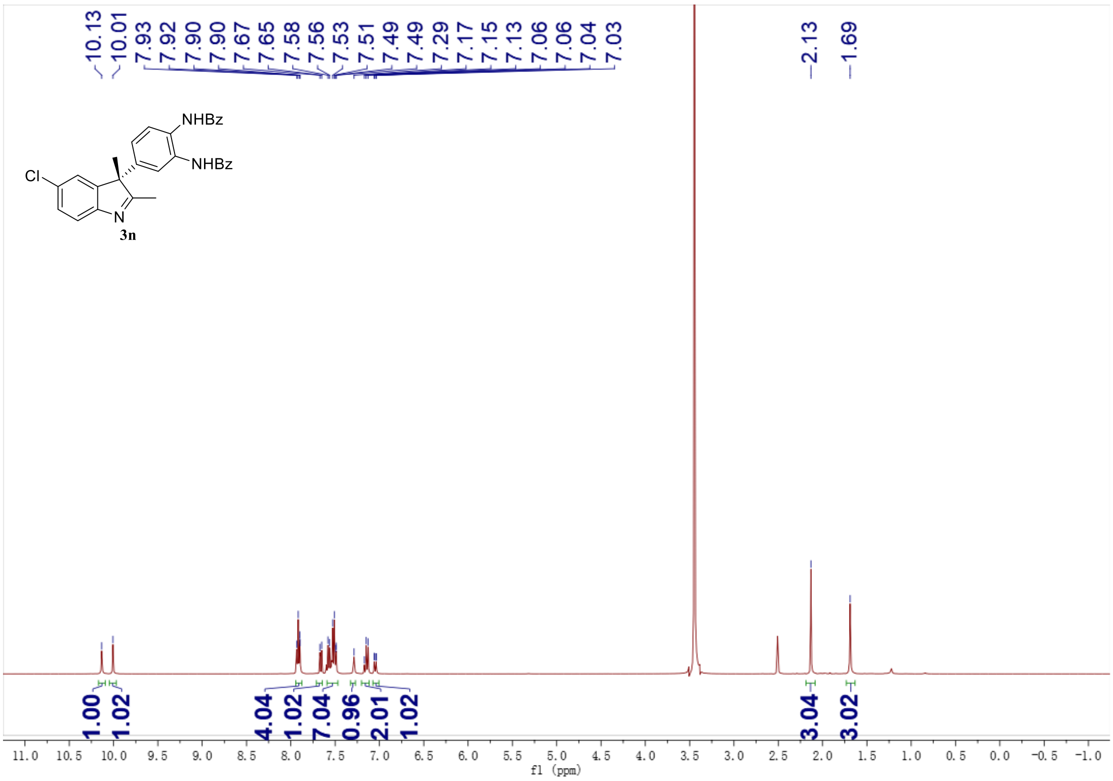

^{13}C NMR spectrum of compound **3n** (($(CD_3)_2SO$, 100 MHz)

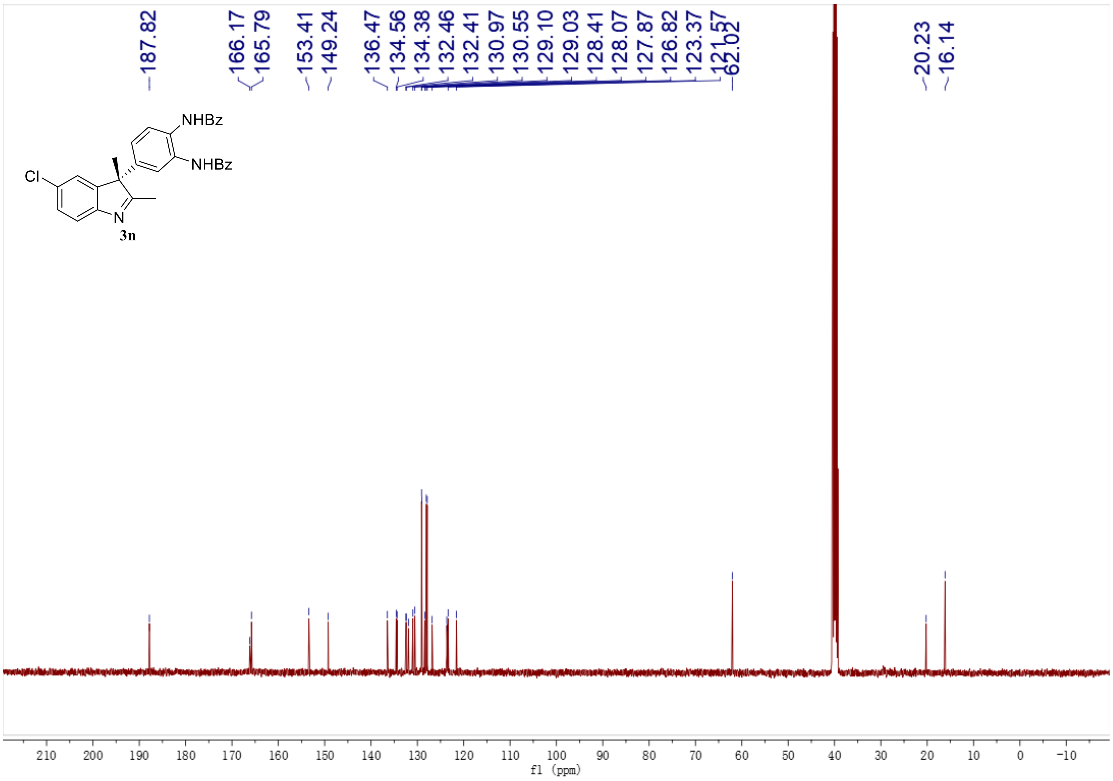

^{1}H NMR spectrum of compound **3o** (($(CD_3)_2SO$, 400 MHz)

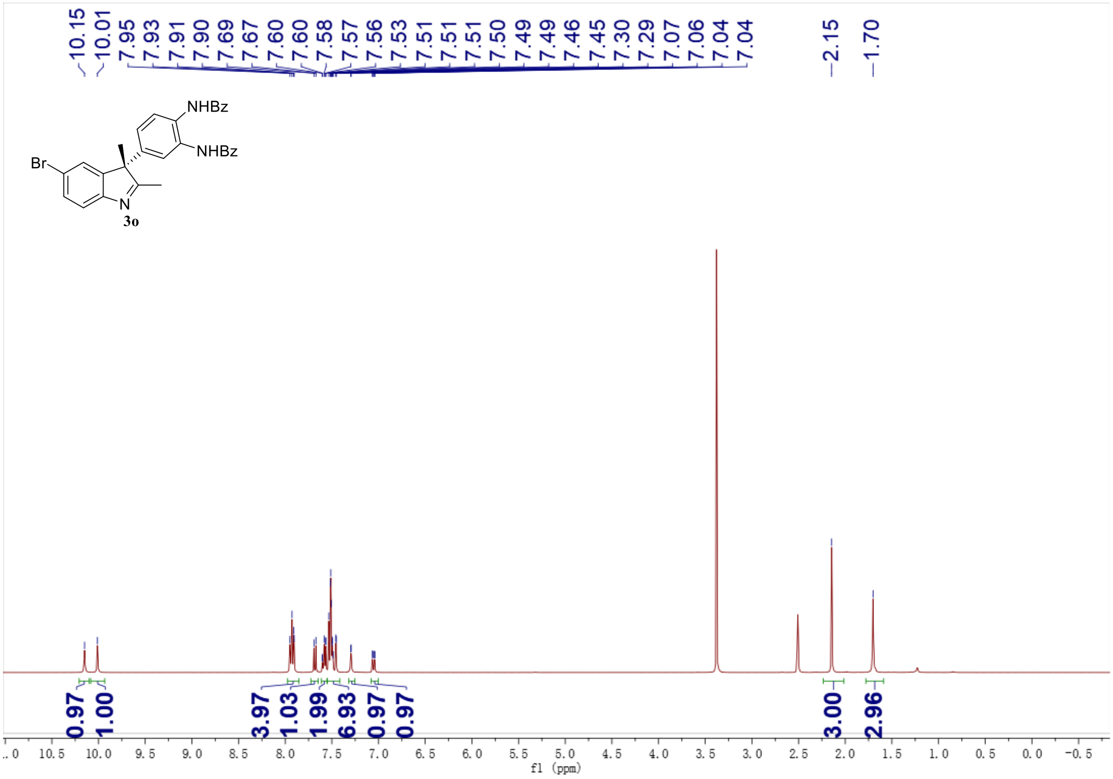

^{13}C NMR spectrum of compound **3o** (($(CD_3)_2SO$, 100 MHz)

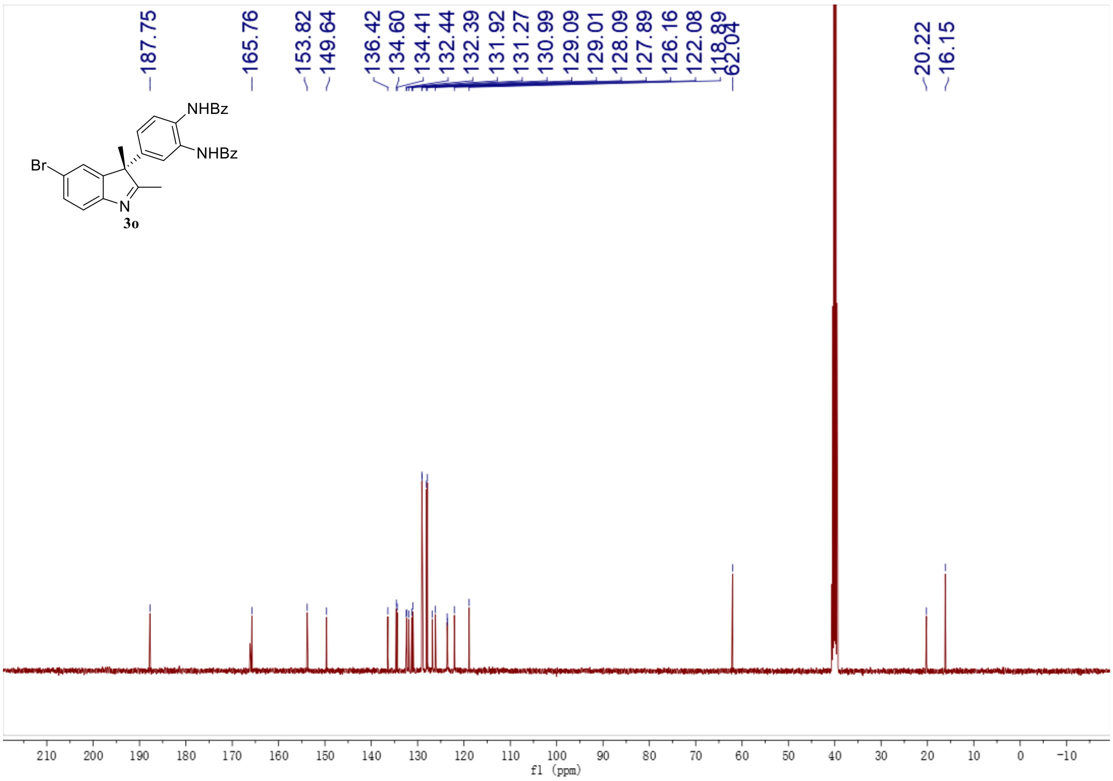

^{1}H NMR spectrum of compound **3p** ($(CD_3)_2SO$, 400 MHz)

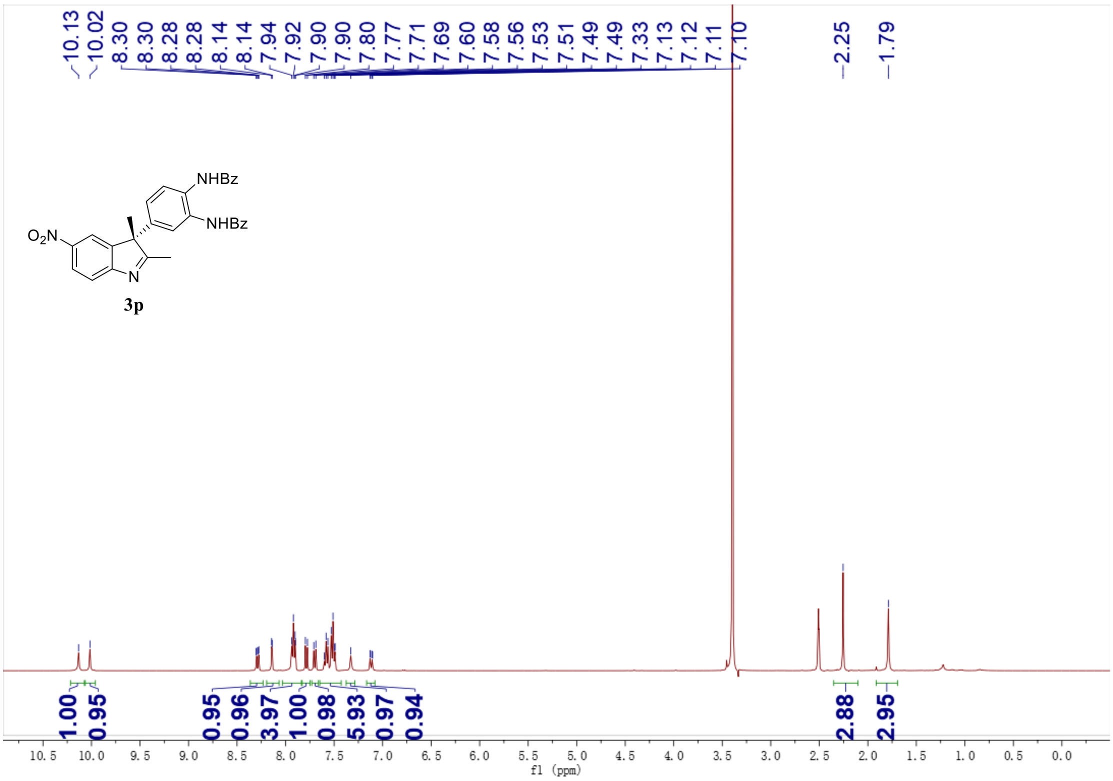

^{13}C NMR spectrum of compound **3p** ($(CD_3)_2SO$, 100 MHz)

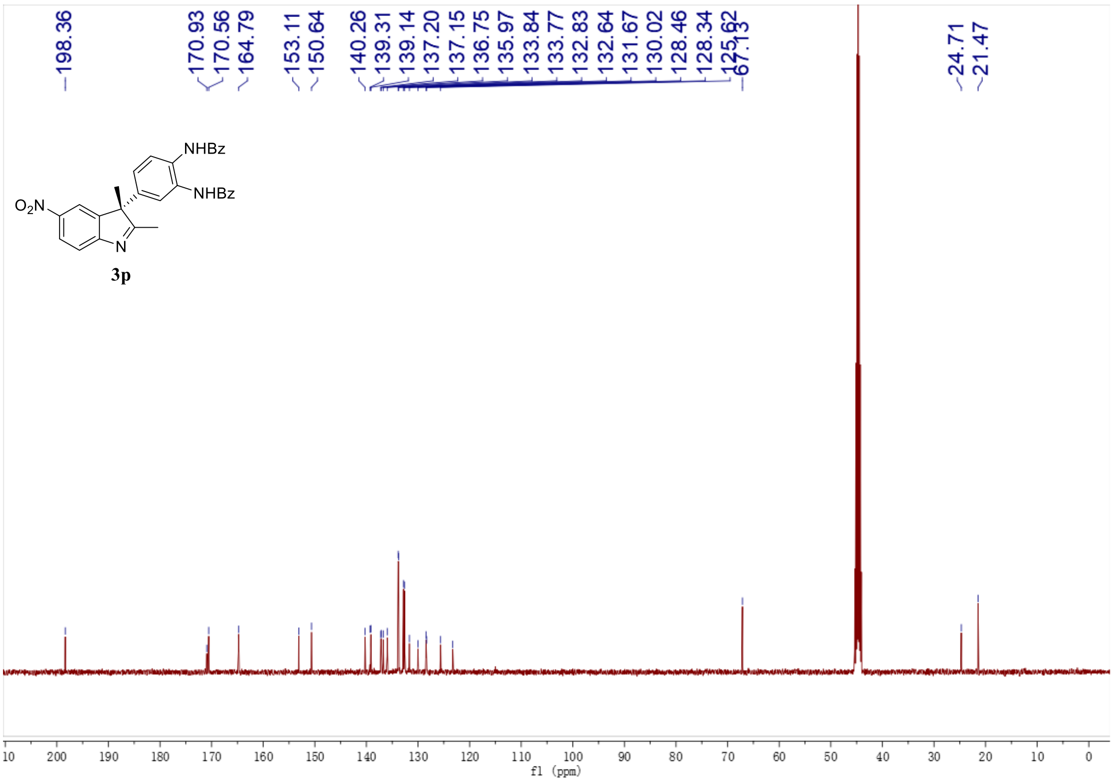

^{1}H NMR spectrum of compound **3q** ($(CD_3)_2SO$, 400 MHz)

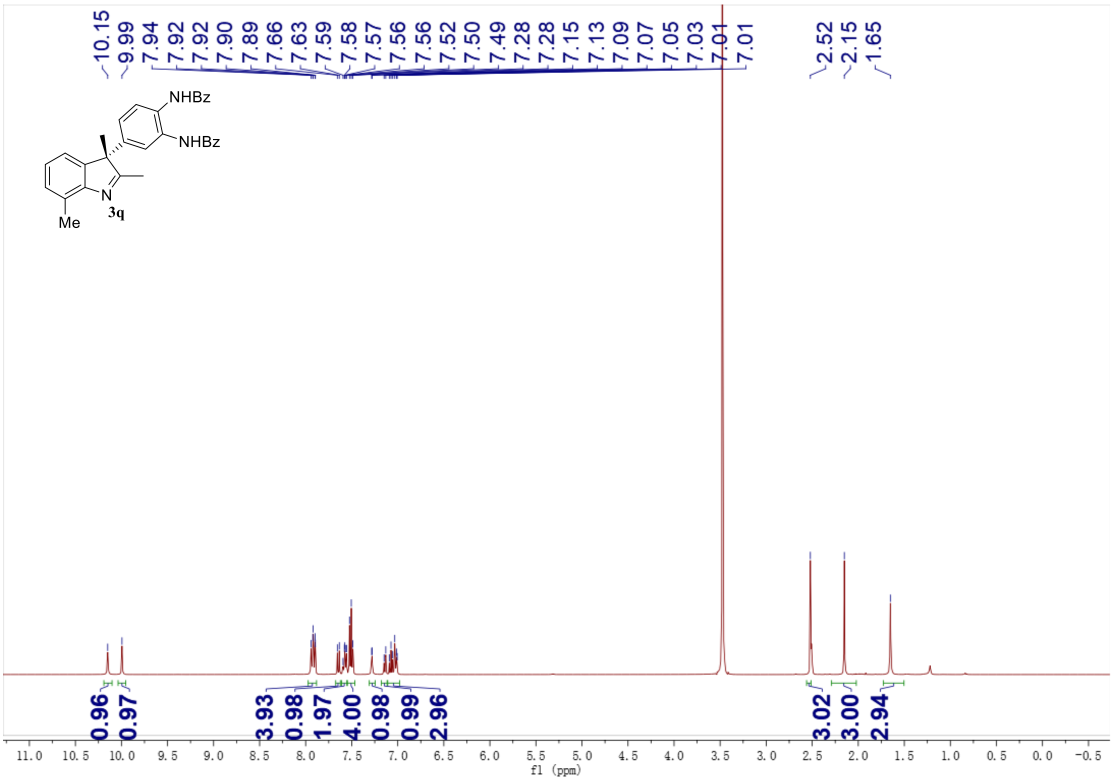

^{13}C NMR spectrum of compound **3q** ($(CD_3)_2SO$, 100 MHz)

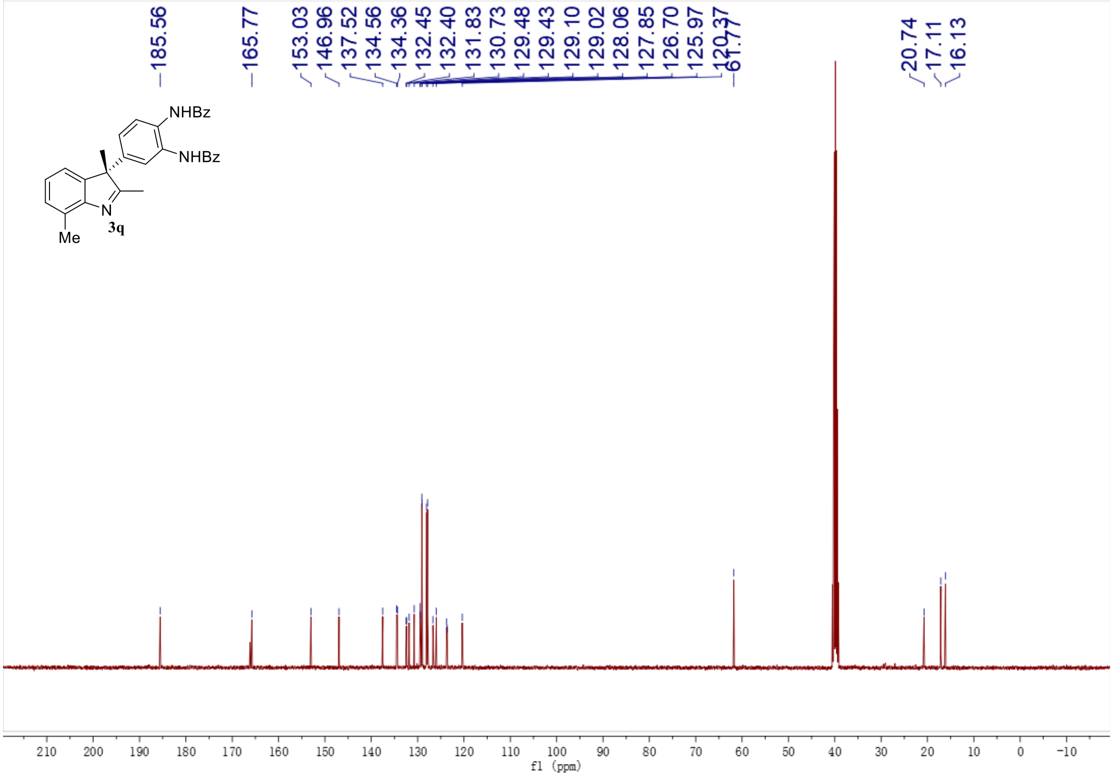

^{1}H NMR spectrum of compound **3r** ($(CD_3)_2SO$, 400 MHz)

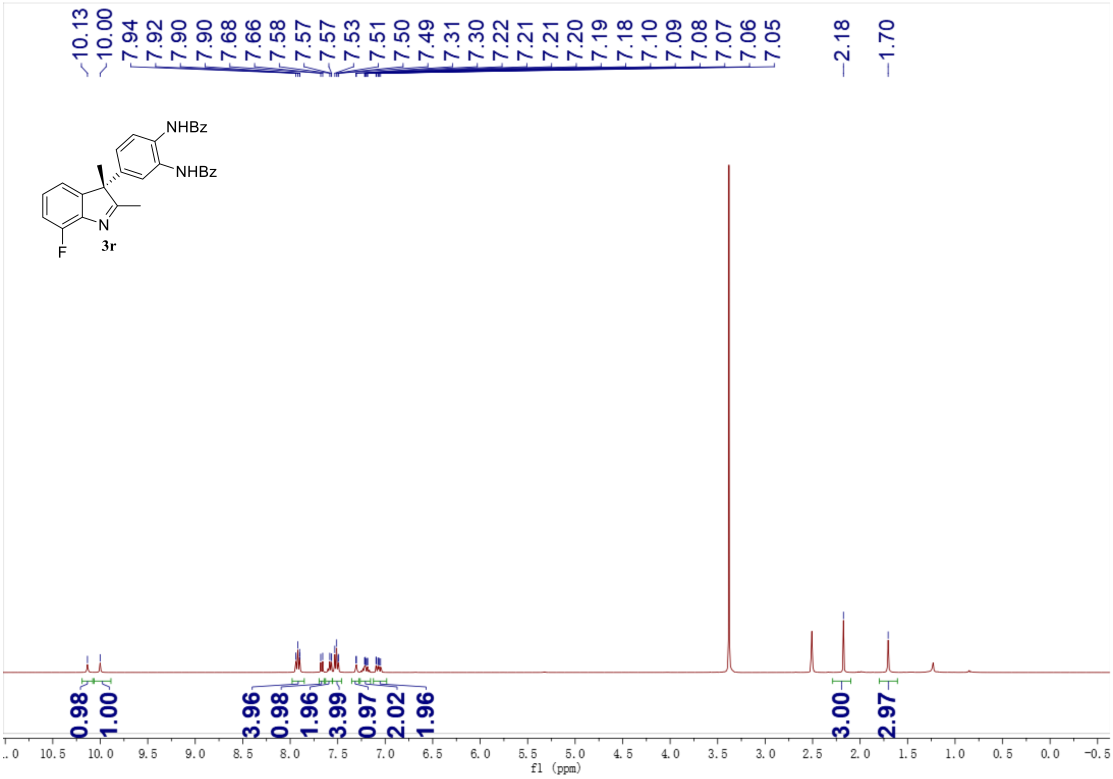

^{13}C NMR spectrum of compound **3r** ($(CD_3)_2SO$, 100 MHz)

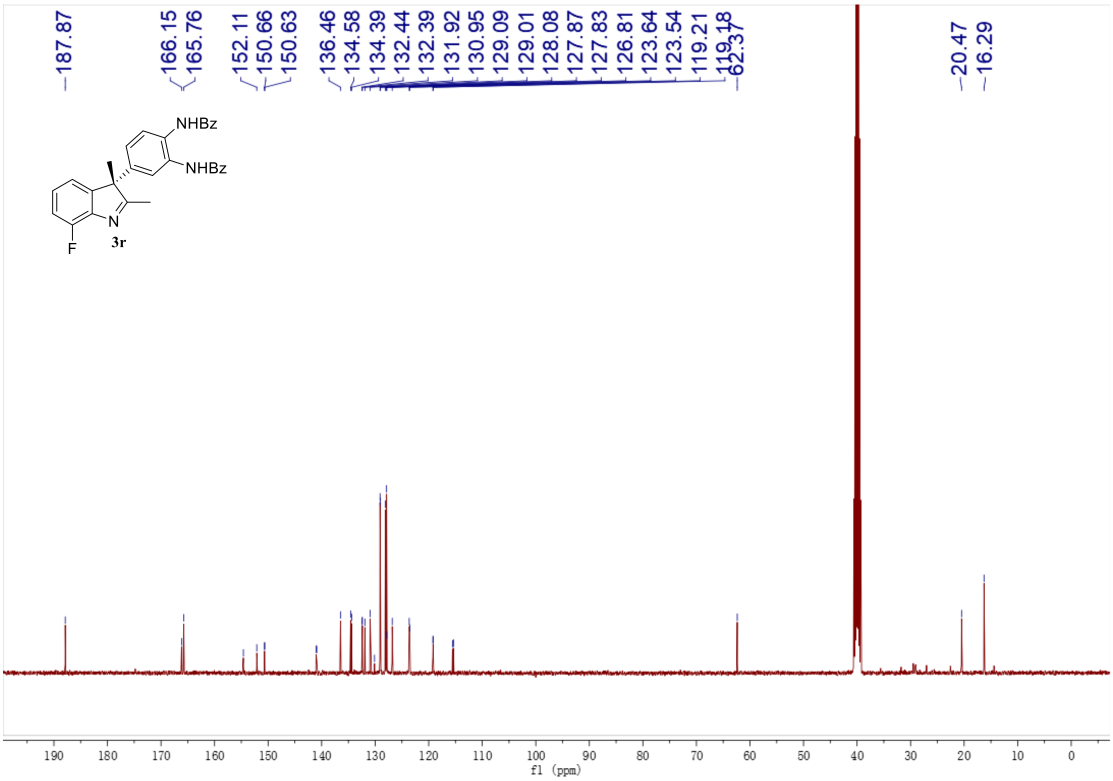

^{19}F NMR spectrum of compound **3r** ($(CD_3)_2SO$, 376 MHz)

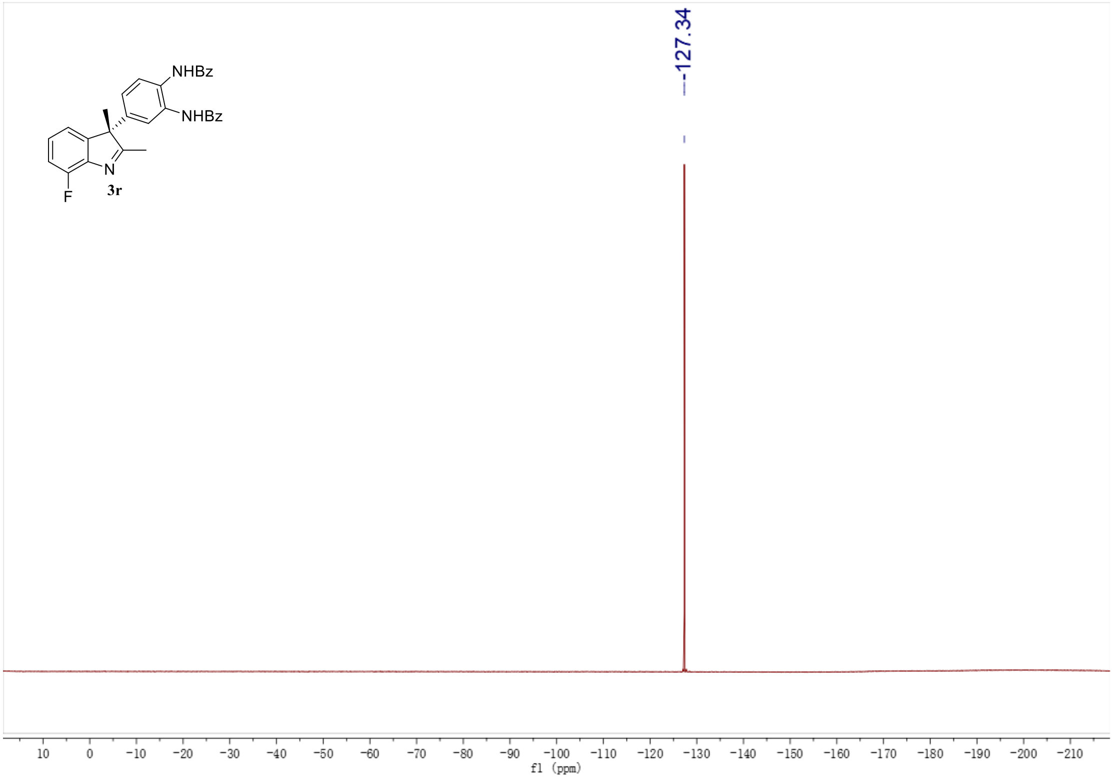

^{1}H NMR spectrum of compound **3s** ($(CD_3)_2SO$, 400 MHz)

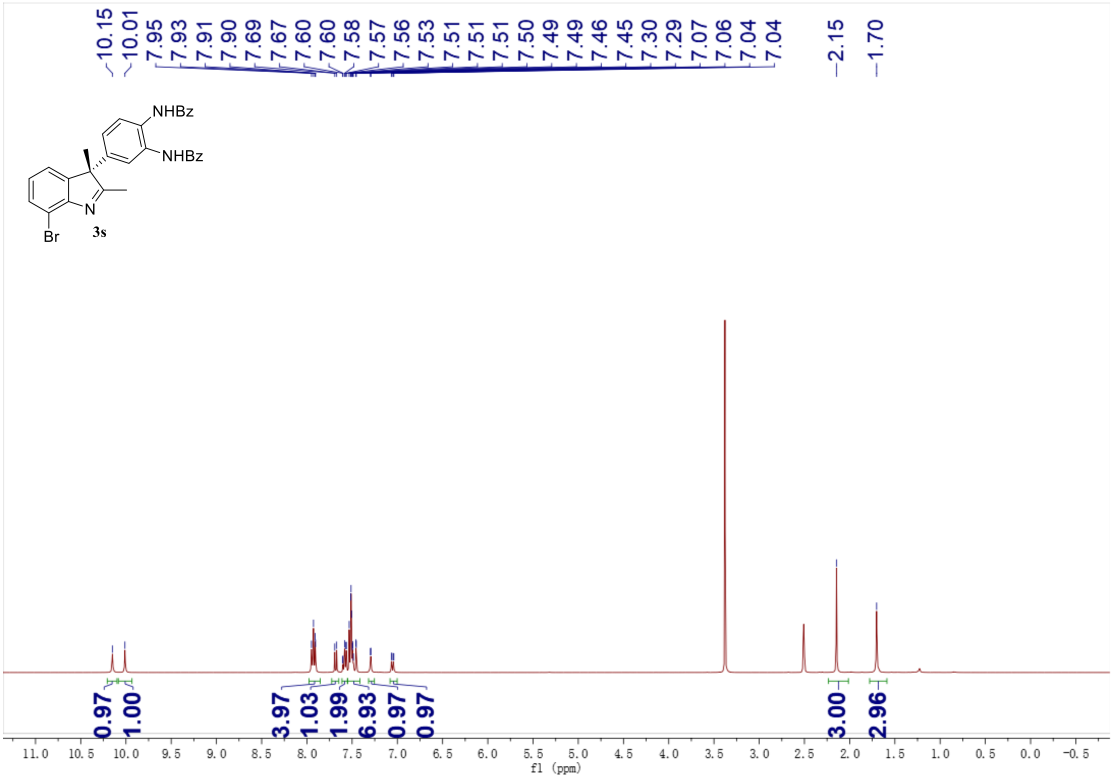

^{13}C NMR spectrum of compound **3s** ($(CD_3)_2SO$, 100 MHz)

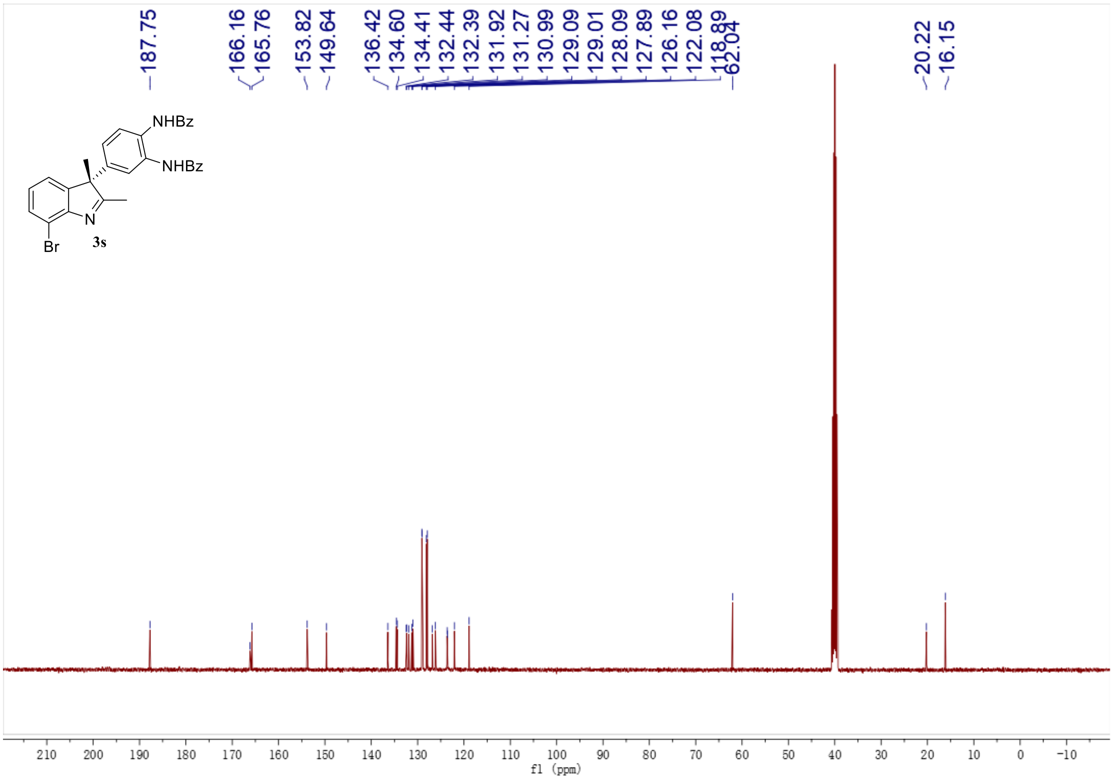

^{1}H NMR spectrum of compound **3t** ($(CD_3)_2SO$, 400 MHz)

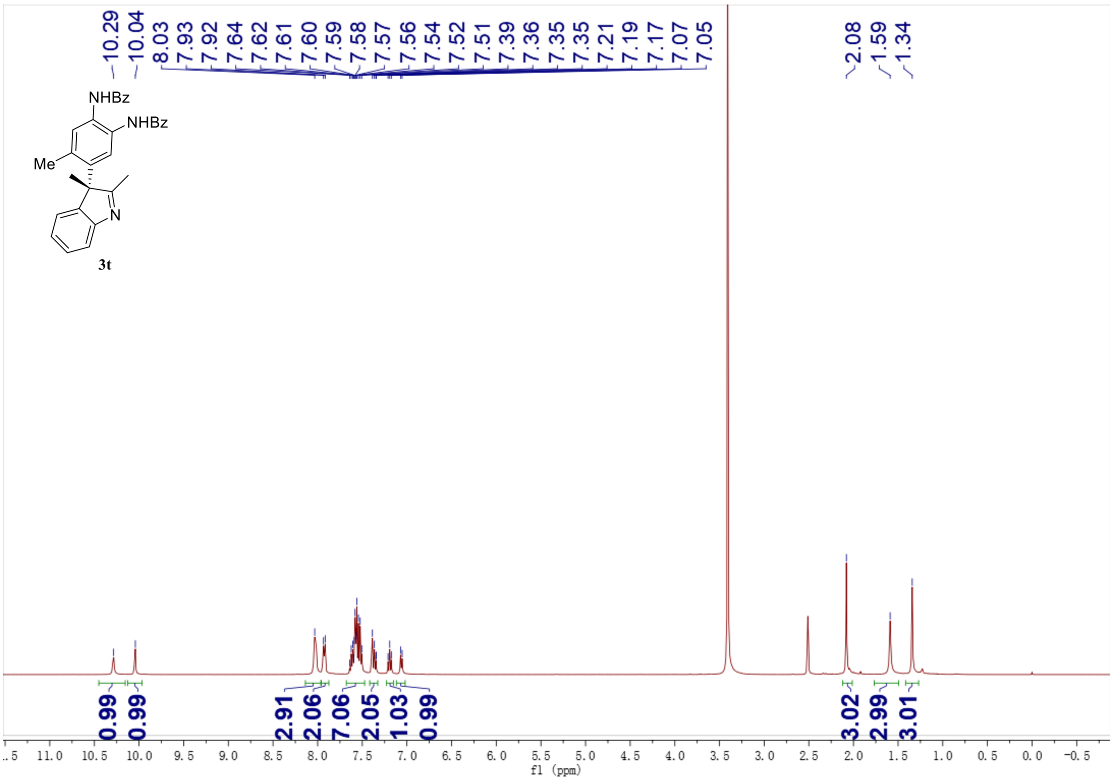

^{13}C NMR spectrum of compound **3t** ($(CD_3)_2SO$, 100 MHz)

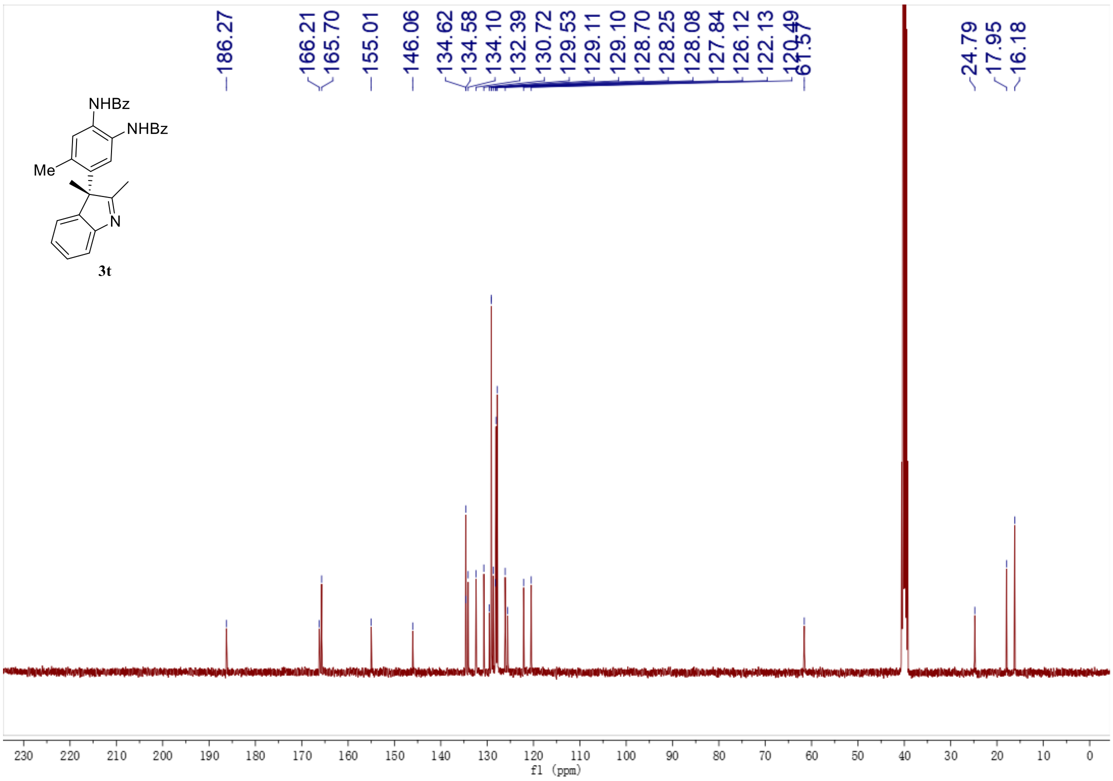

^{1}H NMR spectrum of compound **3u** ($(CD_3)_2SO$, 400 MHz)

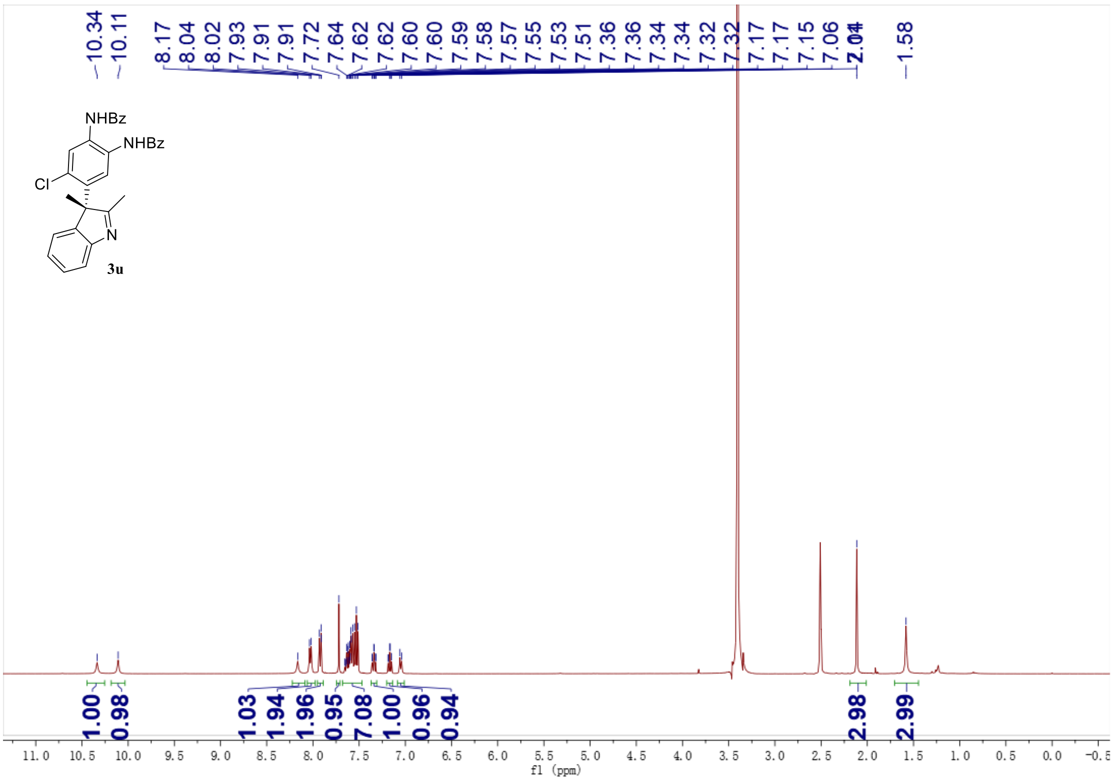

^{13}C NMR spectrum of compound **3u** ($(CD_3)_2SO$, 100 MHz)

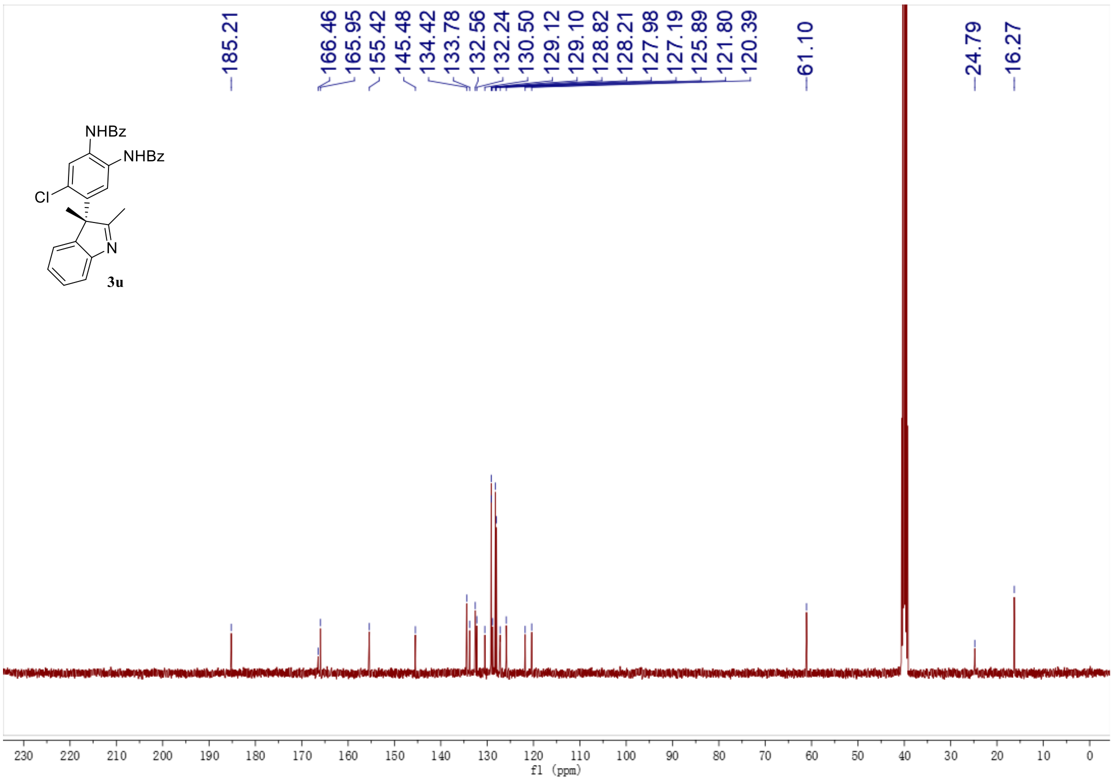

^{1}H NMR spectrum of compound **3v** ($(CD_3)_2SO$, 400 MHz)

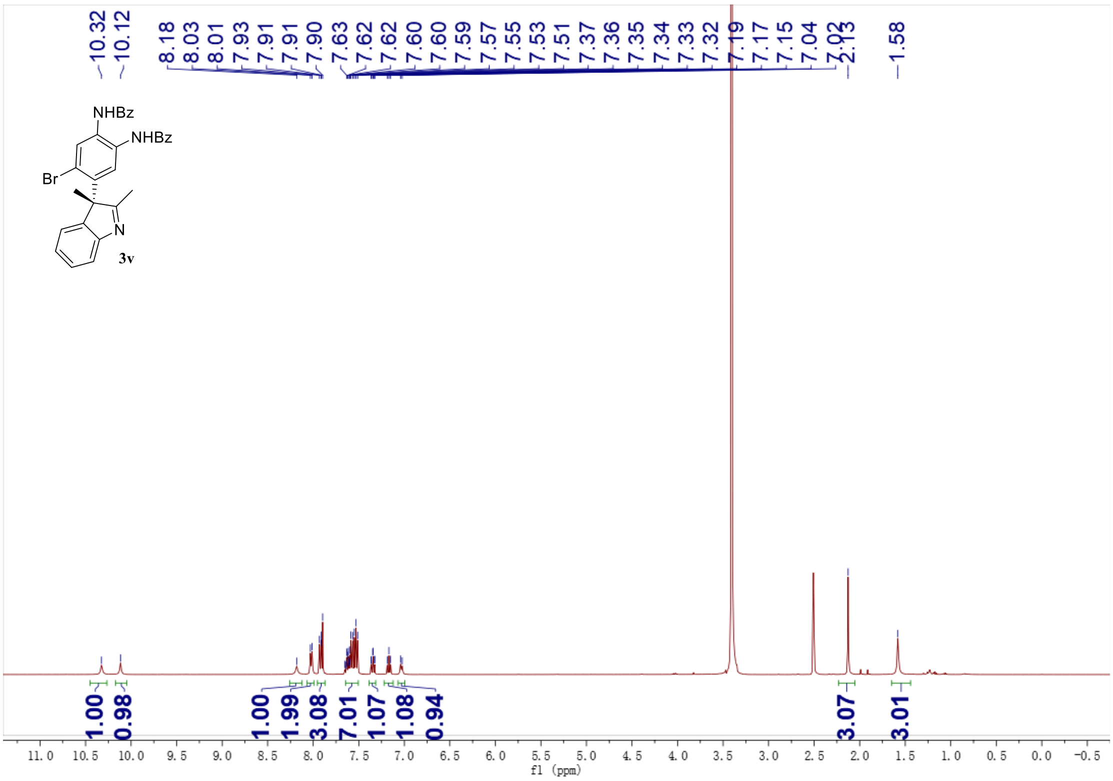

^{13}C NMR spectrum of compound **3v** ($(CD_3)_2SO$, 100 MHz)

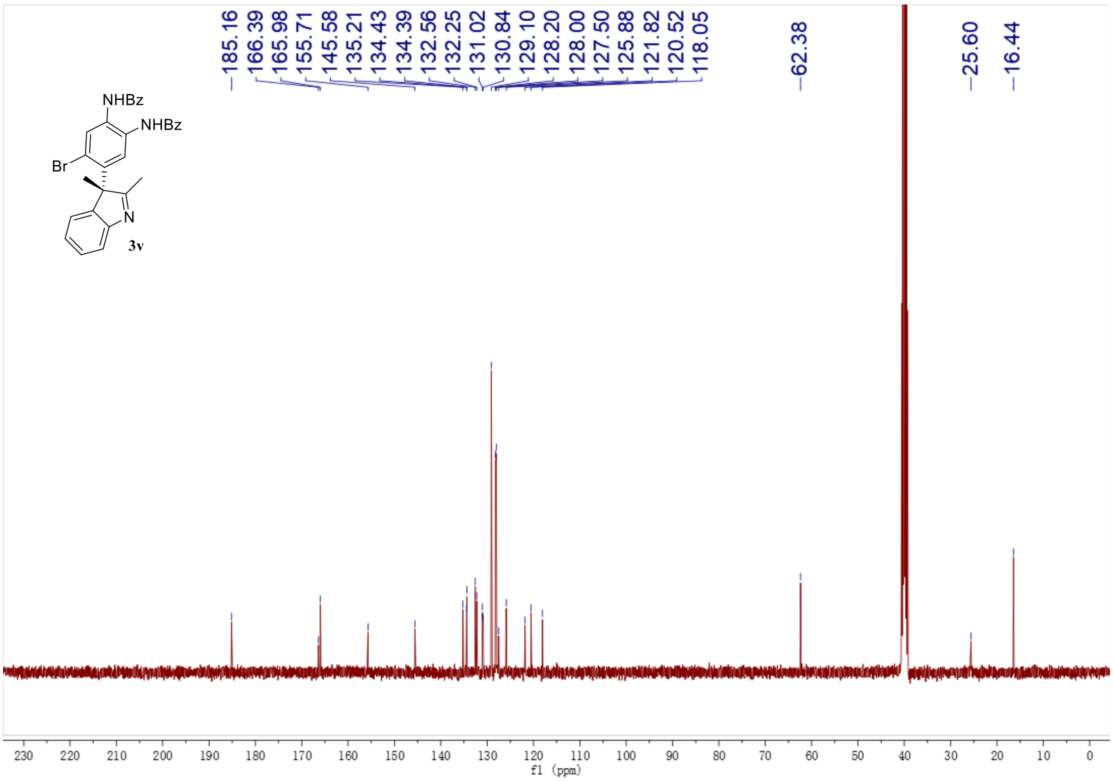

^{1}H NMR spectrum of compound **3w** ($(CD_3)_2SO$, 400 MHz)

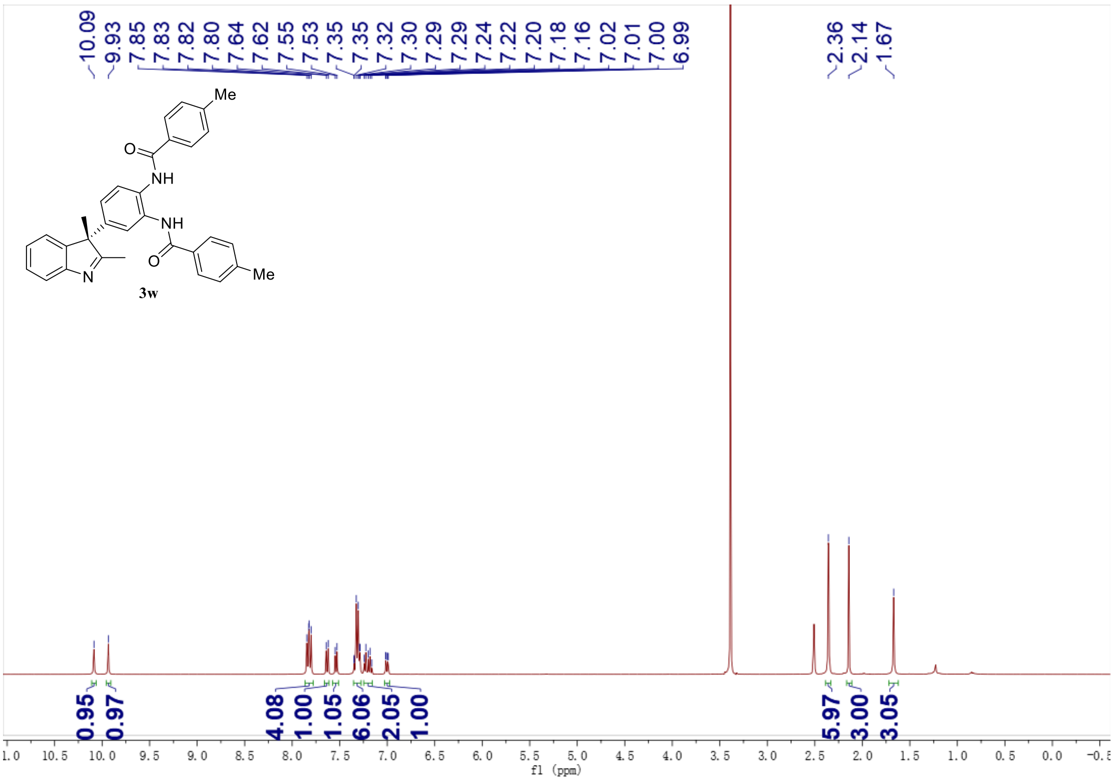

^{13}C NMR spectrum of compound **3w** ($(CD_3)_2SO$, 100 MHz)

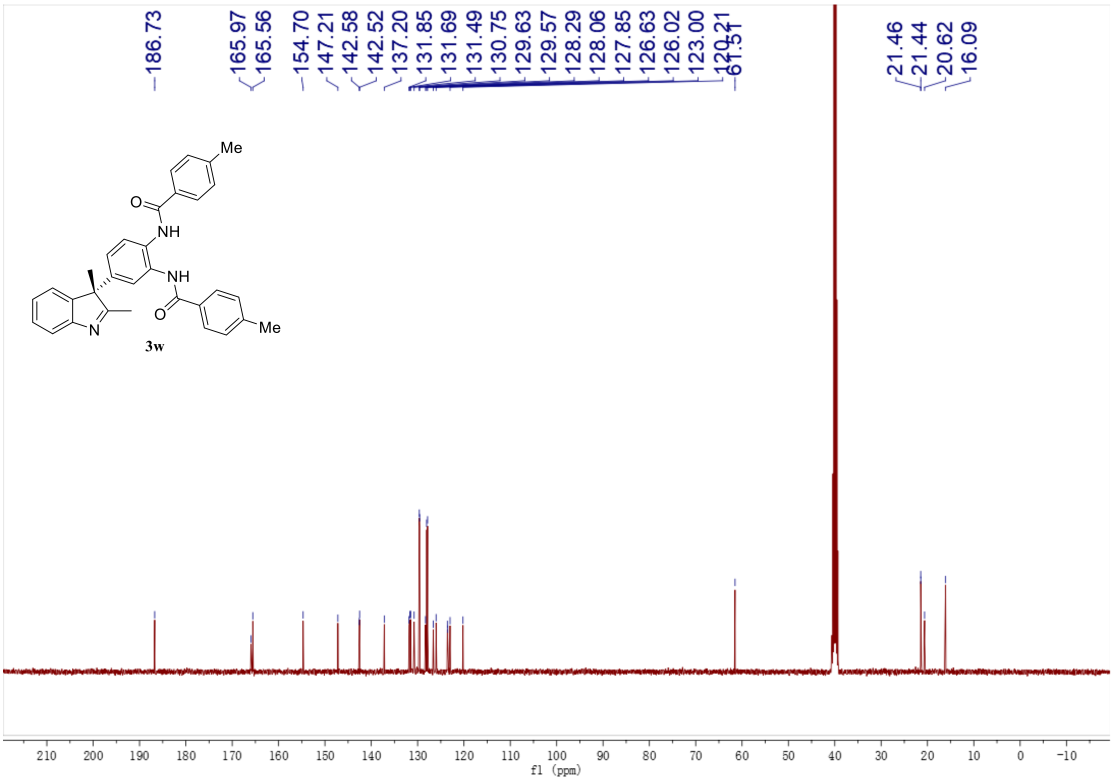

^{1}H NMR spectrum of compound **3x** (($(CD_3)_2SO$, 400 MHz)

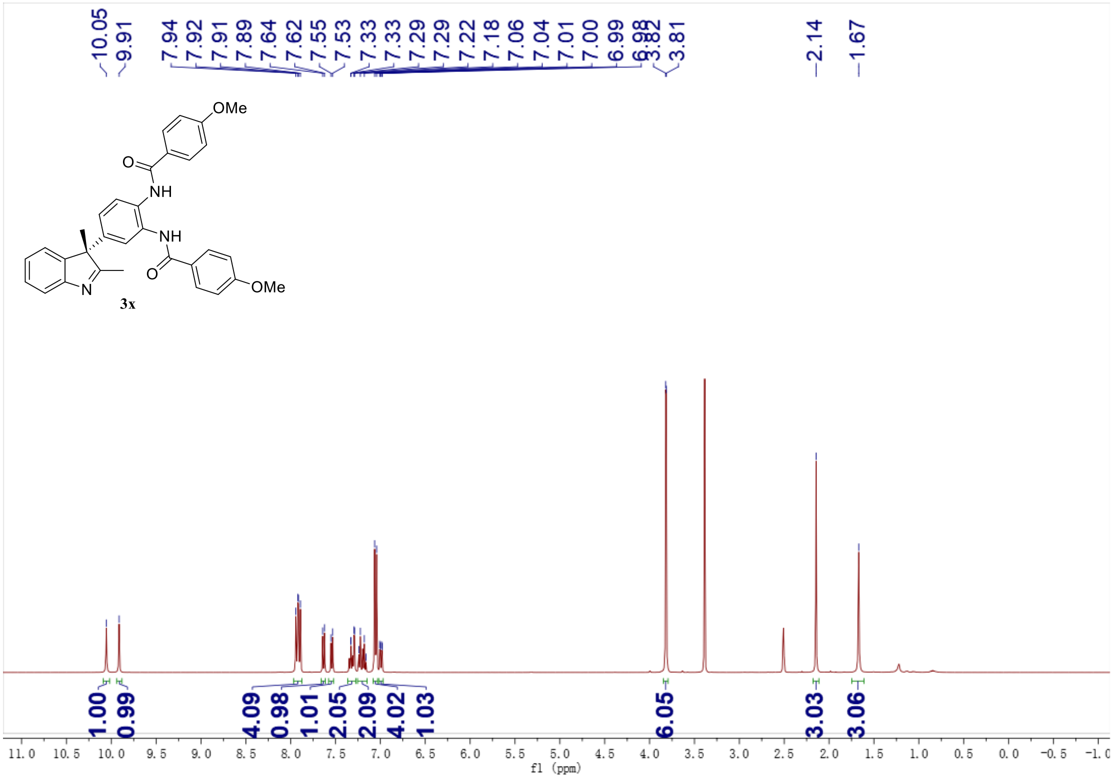

^{13}C NMR spectrum of compound **3x** (($(CD_3)_2SO$, 100 MHz)

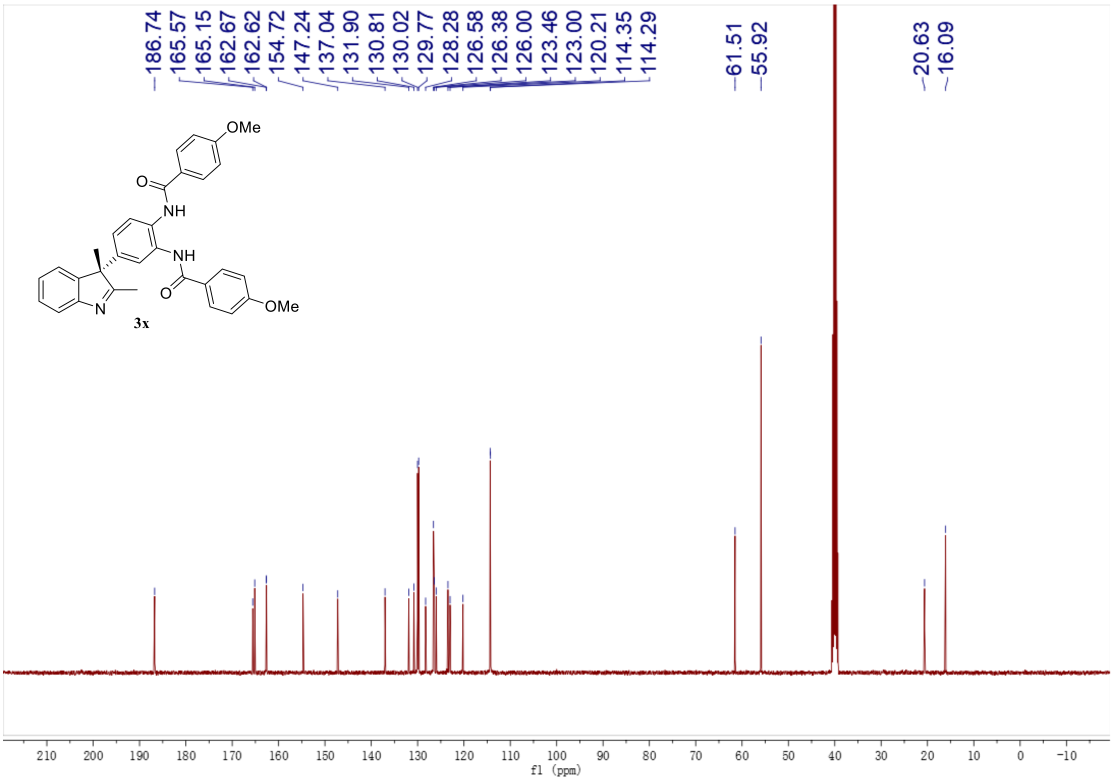

^{1}H NMR spectrum of compound **3y** ($(CD_3)_2SO$, 400 MHz)

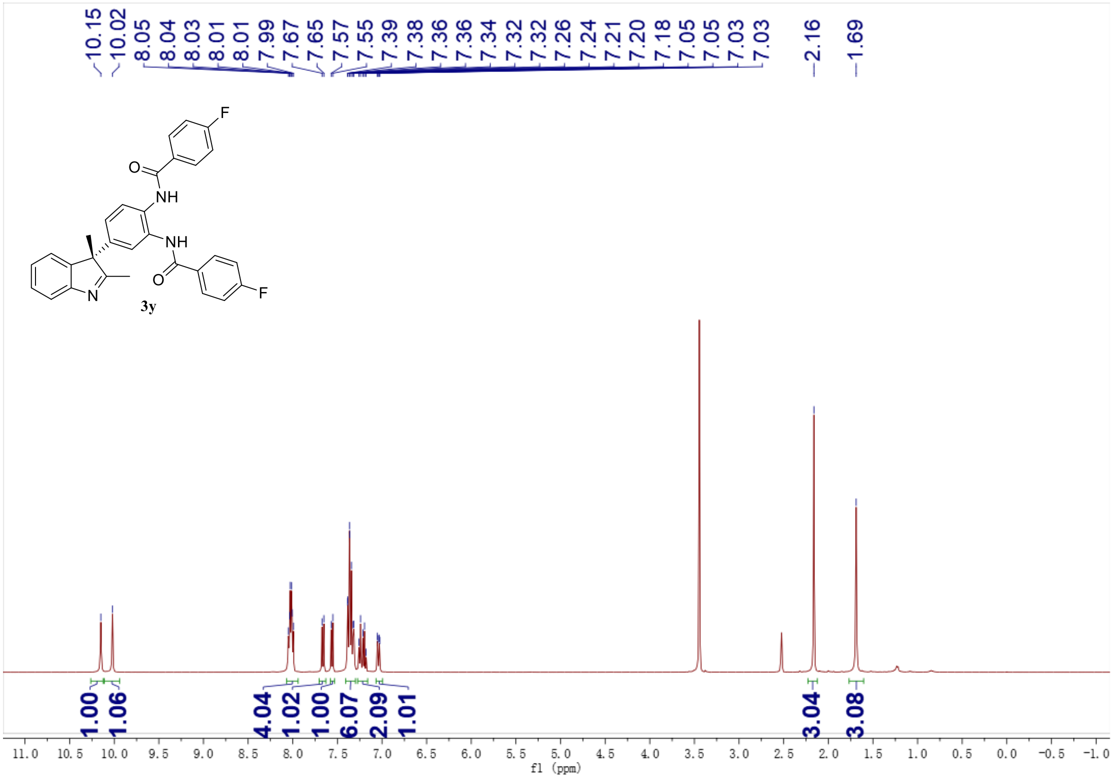

^{13}C NMR spectrum of compound **3y** ($(CD_3)_2SO$, 100 MHz)

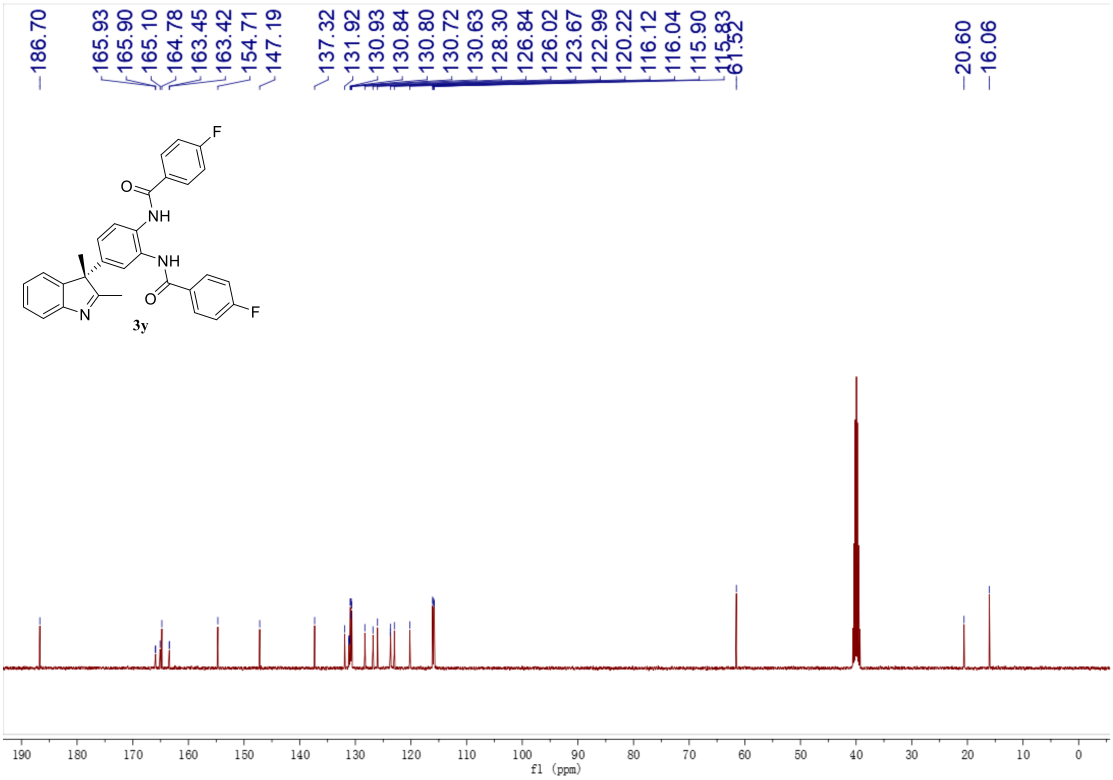

^{19}F NMR spectrum of compound **3y** ((CD_3)$_2$SO, 376 MHz)

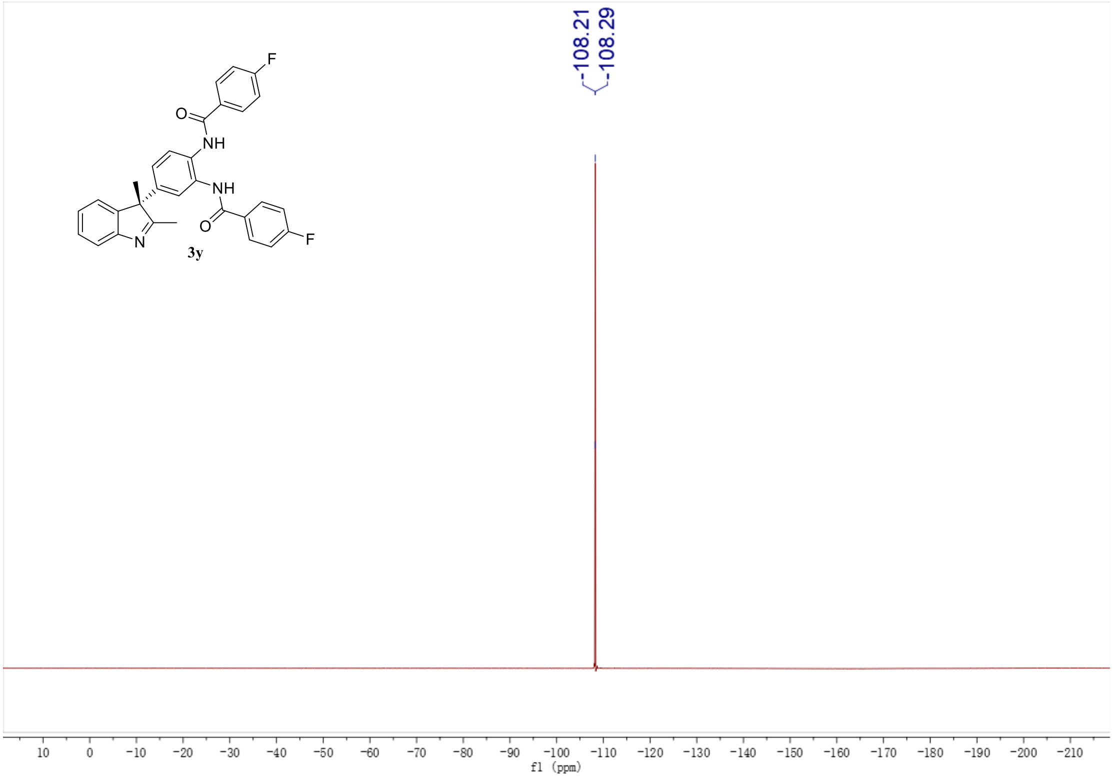

^{1}H NMR spectrum of compound **3z** ((CD$_3$)$_2$SO, 400 MHz)

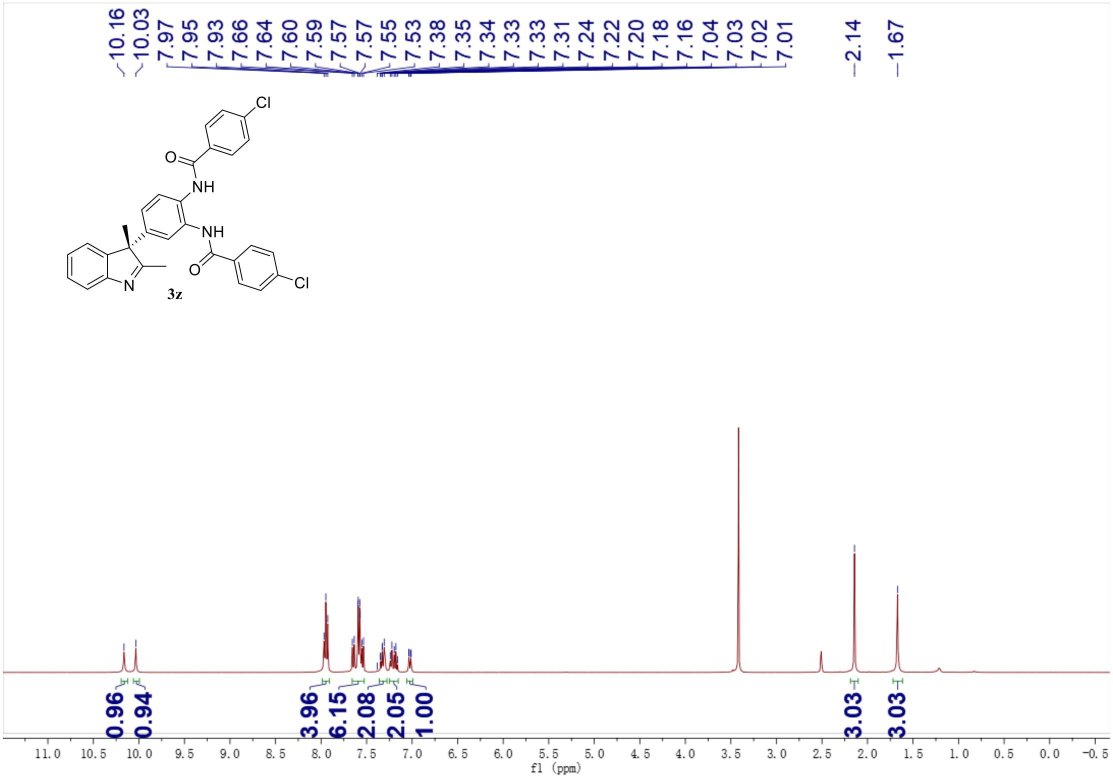

^{13}C NMR spectrum of compound **3z** ((CD$_3$)$_2$SO, 100 MHz)

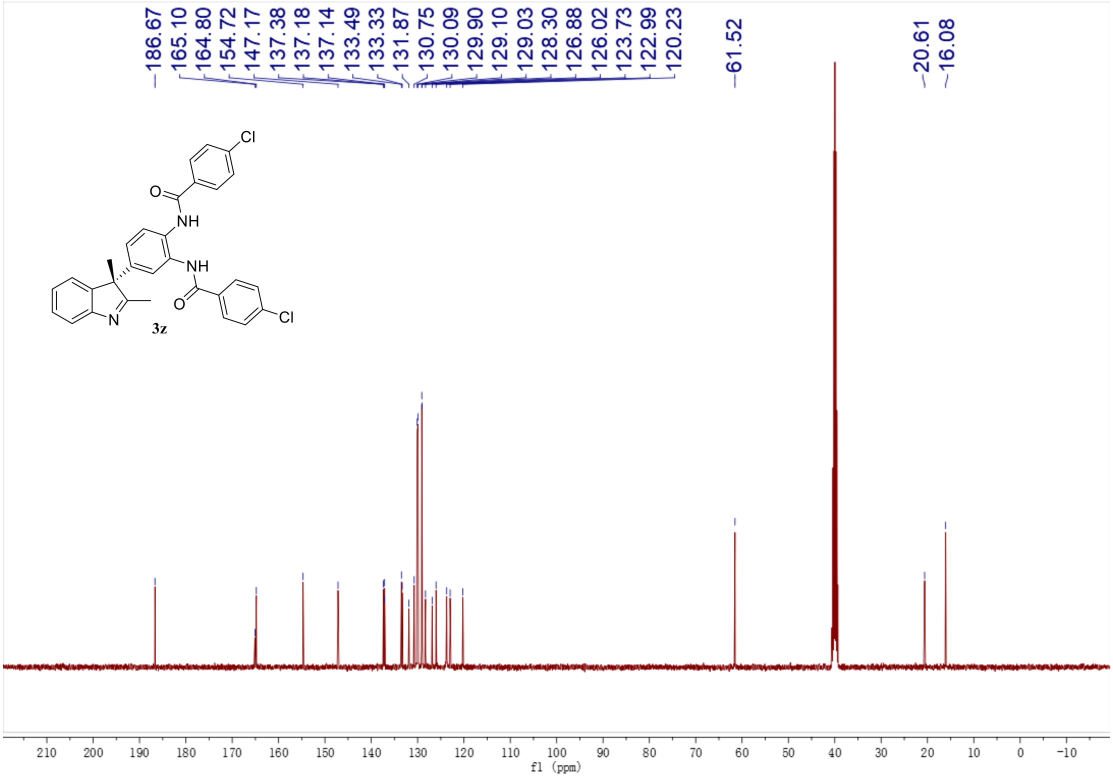

^{1}H NMR spectrum of compound **3a'** ($(CD_3)_2SO$, 400 MHz)

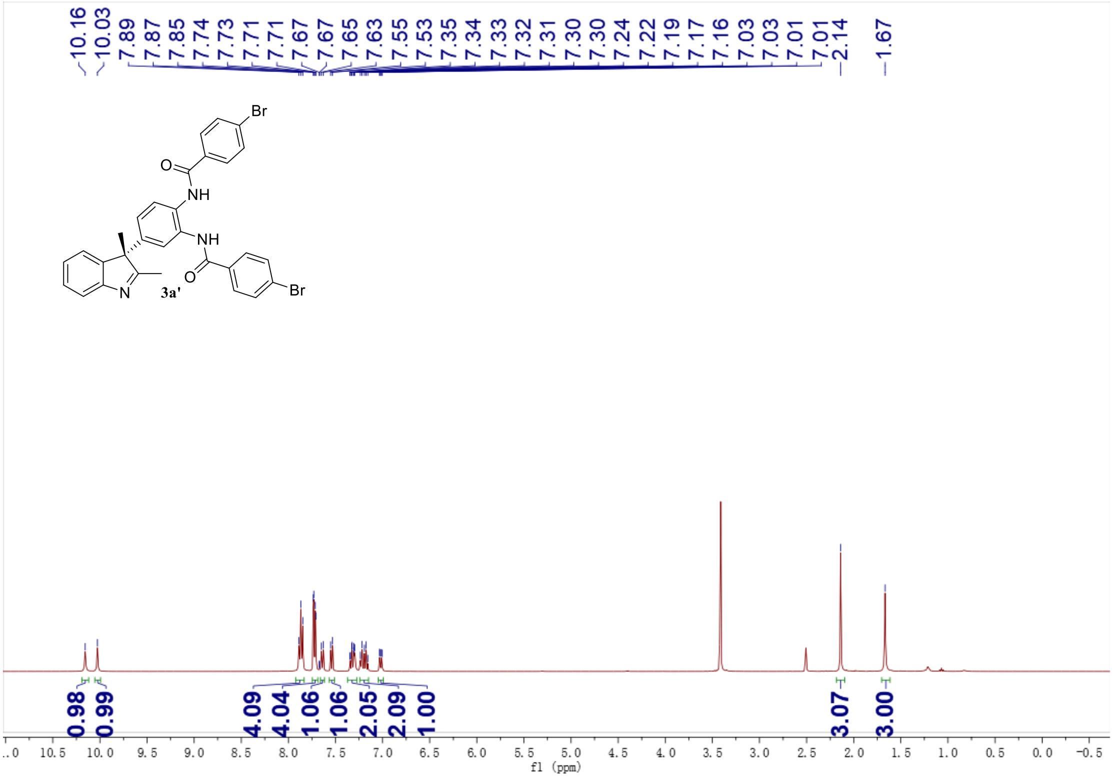

^{13}C NMR spectrum of compound **3a'** ($(CD_3)_2SO$, 100 MHz)

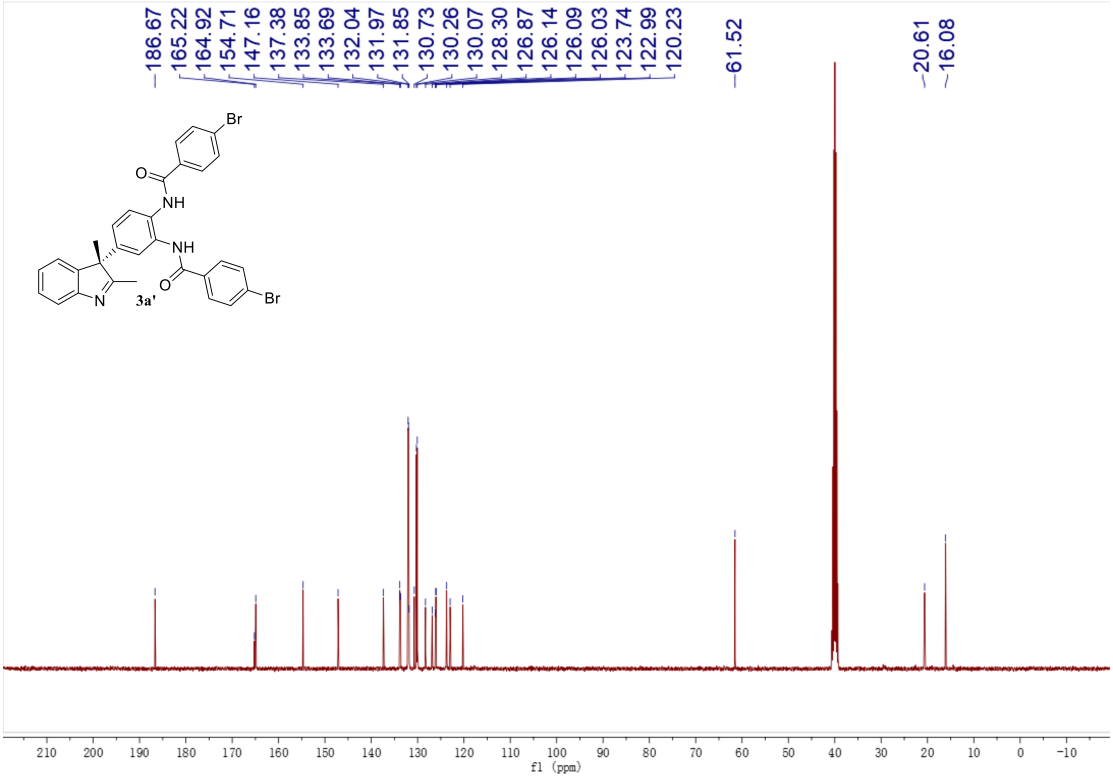

^{1}H NMR spectrum of compound **3b'** (($(CD_3)_2SO$, 400 MHz)

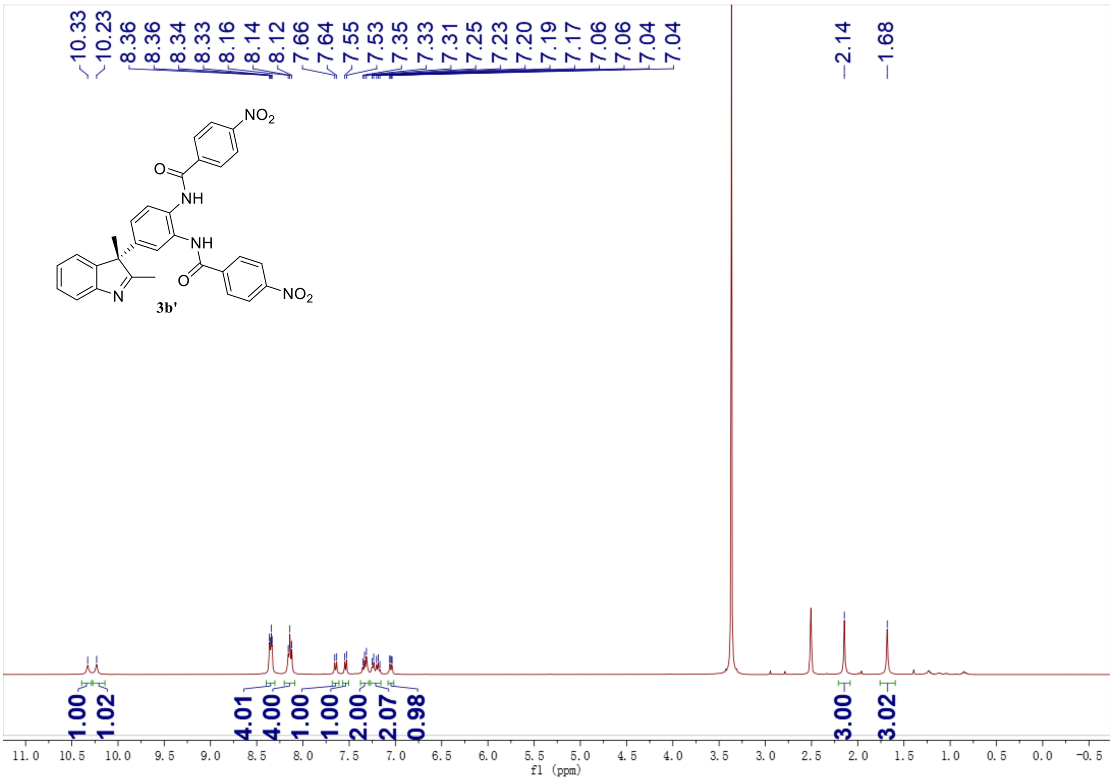

^{13}C NMR spectrum of compound **3b'** (($(CD_3)_2SO$, 100 MHz)

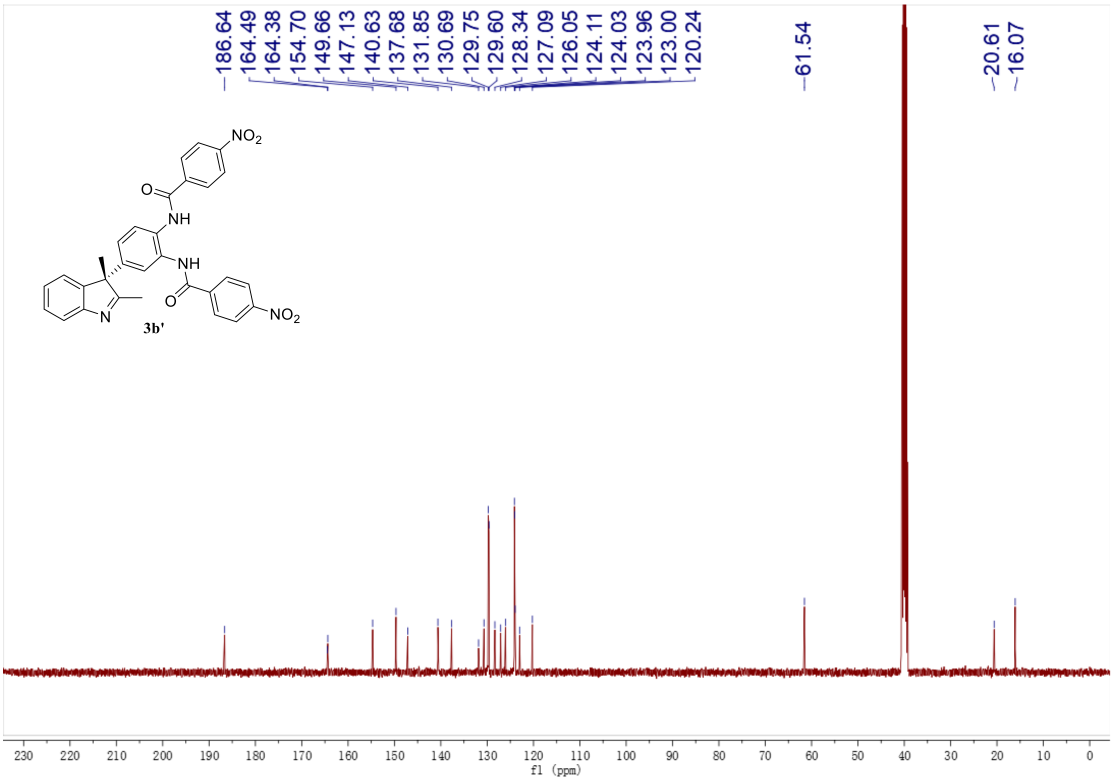

^{1}H NMR spectrum of compound **3c'** ((CD$_3$)$_2$SO, 400 MHz)

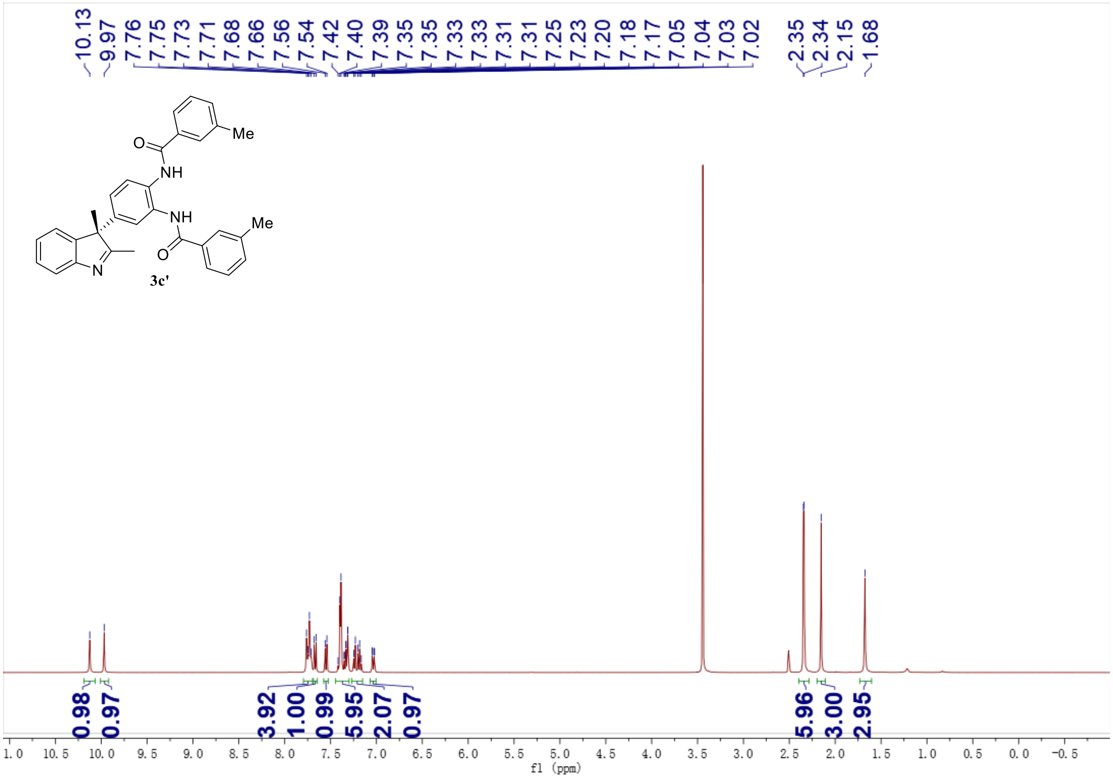

^{13}C NMR spectrum of compound **3c'** ((CD$_3$)$_2$SO, 100 MHz)

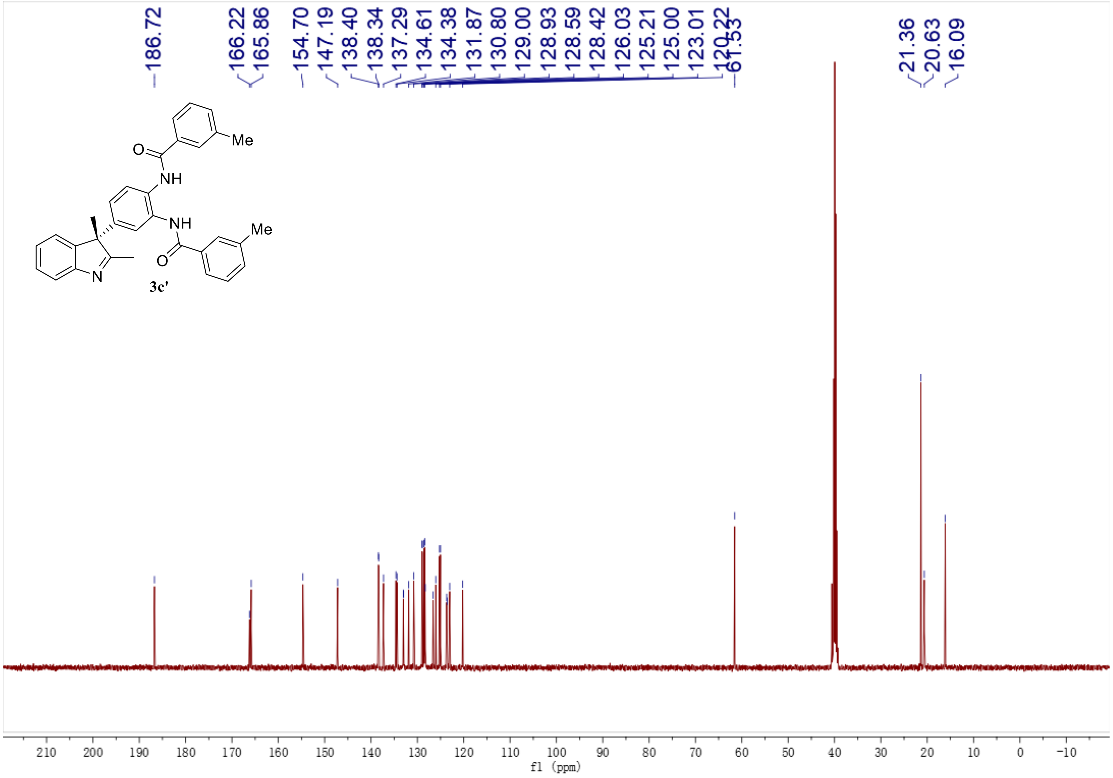

^{1}H NMR spectrum of compound **3d'** ($(CD_3)_2SO$, 400 MHz)

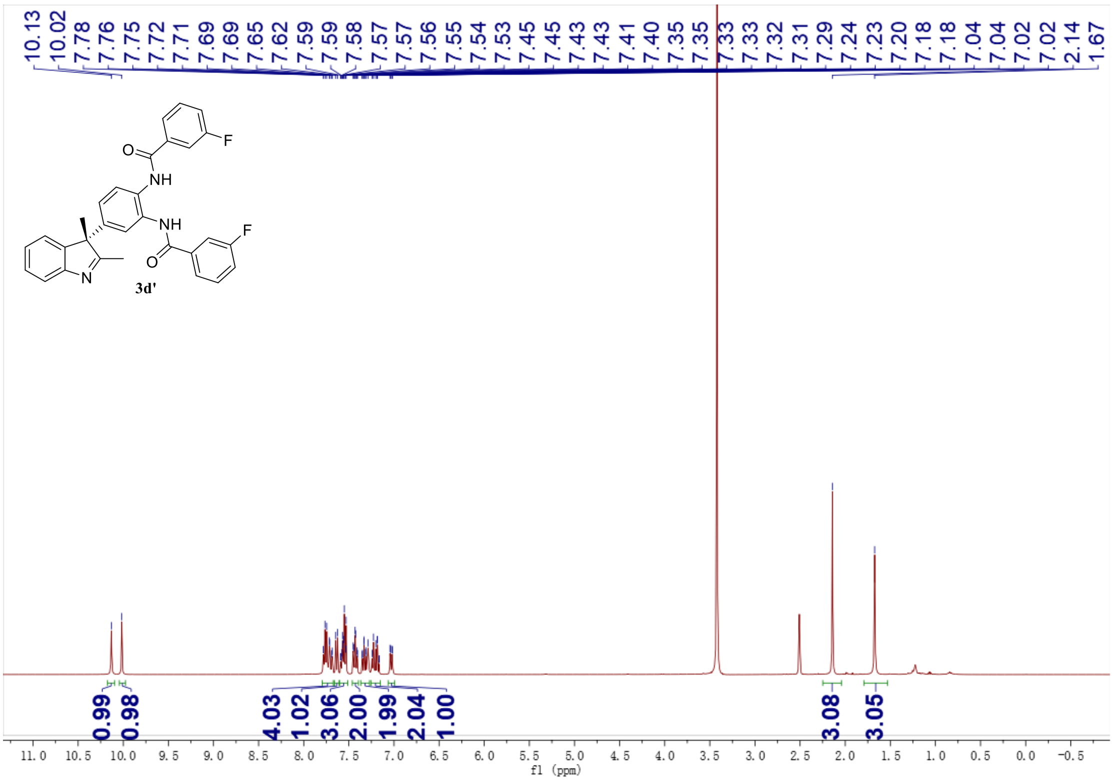

^{13}C NMR spectrum of compound **3d'** ($(CD_3)_2SO$, 100 MHz)

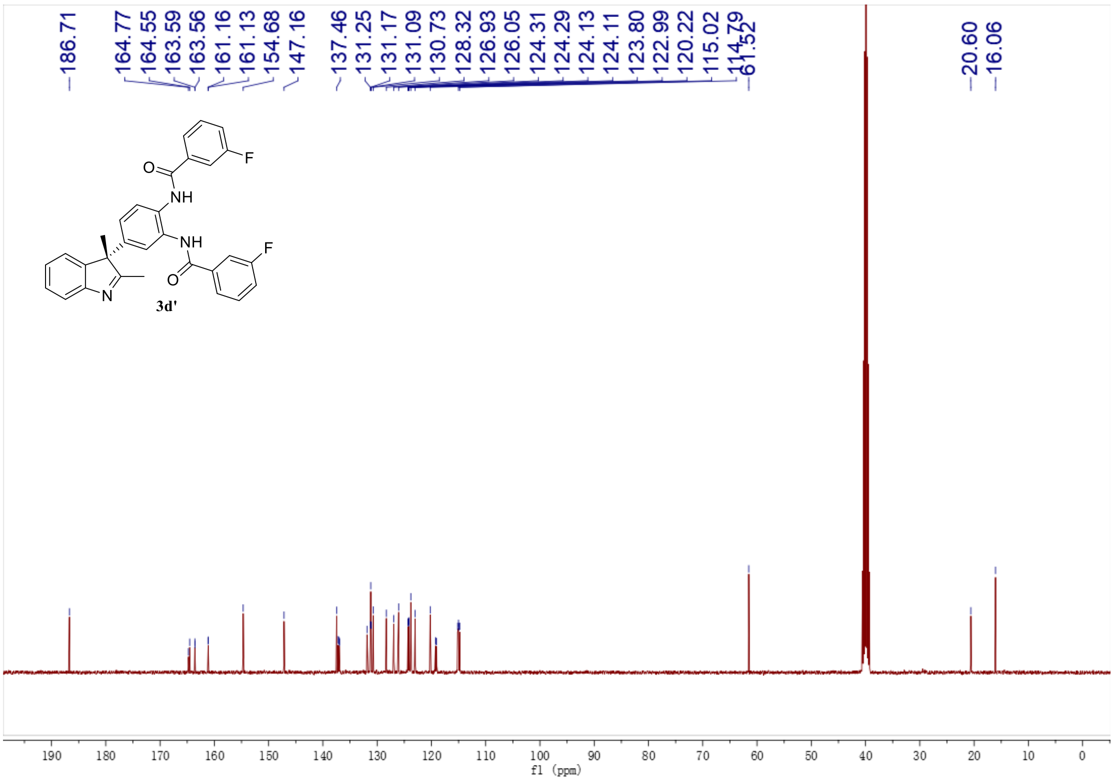

^{19}F NMR spectrum of compound **3d'** ($(CD_3)_2SO$, 376 MHz)

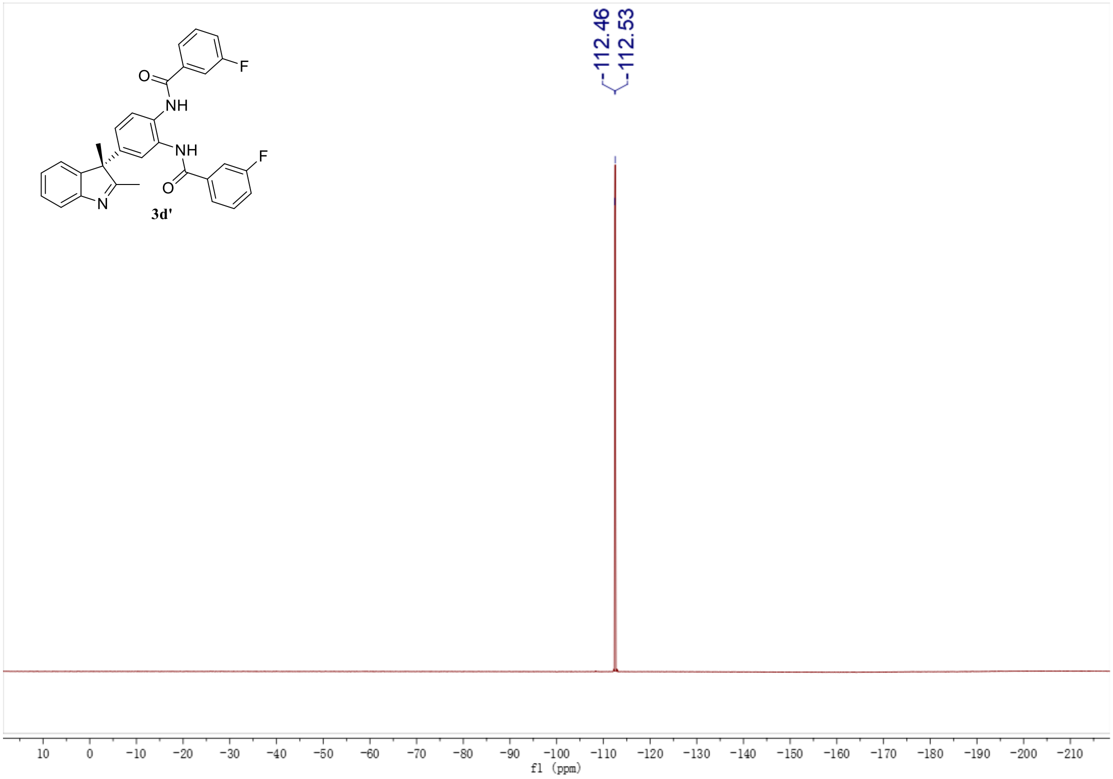

^{1}H NMR spectrum of compound **3e'** ((CD_3)$_2$SO, 400 MHz)

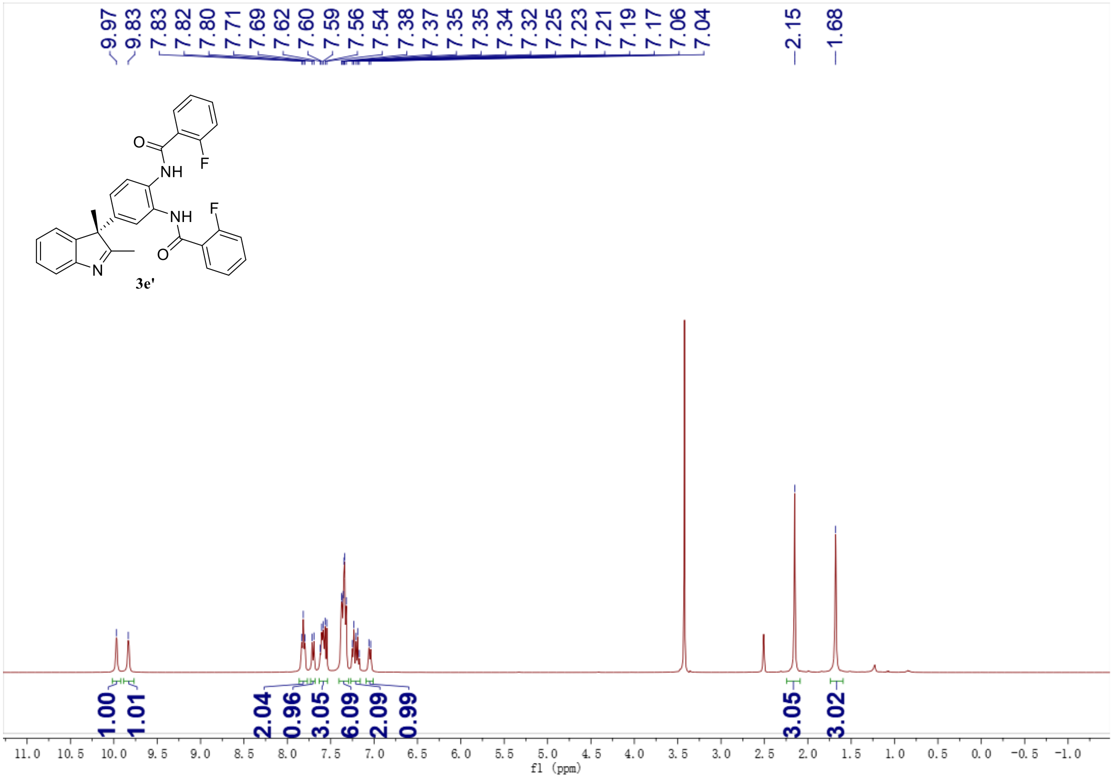

^{13}C NMR spectrum of compound **3e'** ((CD_3)$_2$SO, 100 MHz)

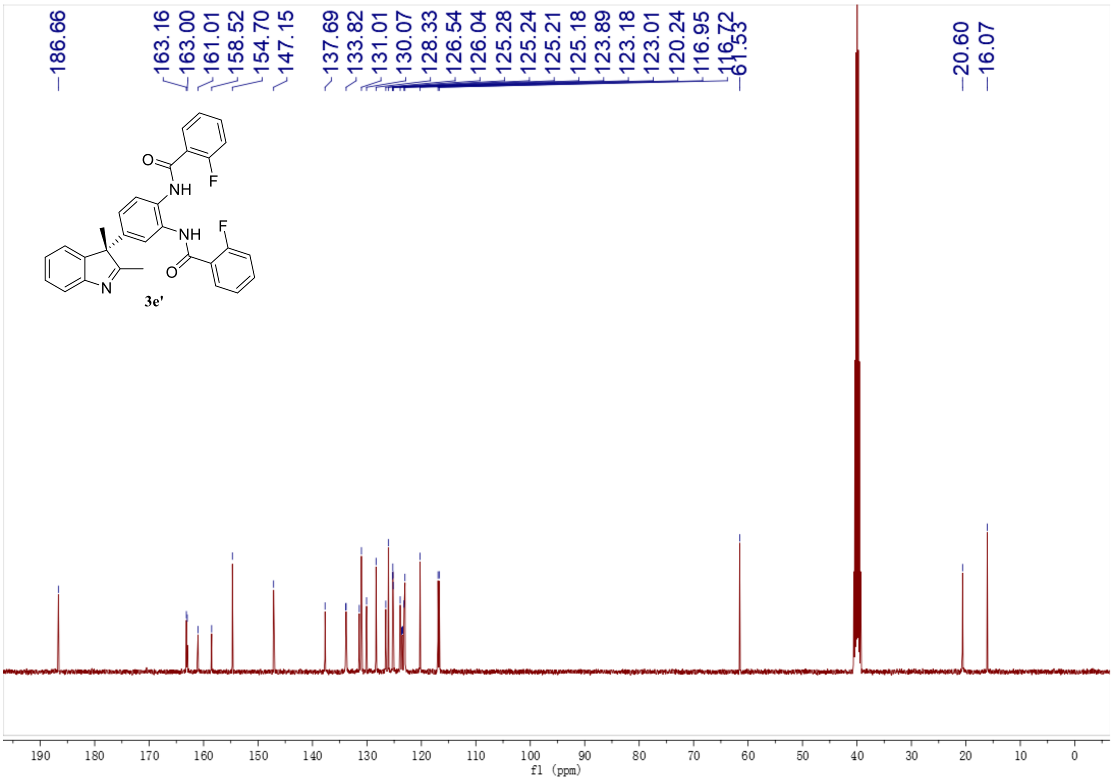

^{19}F NMR spectrum of compound **3e'** ($(CD_3)_2SO$, 376 MHz)

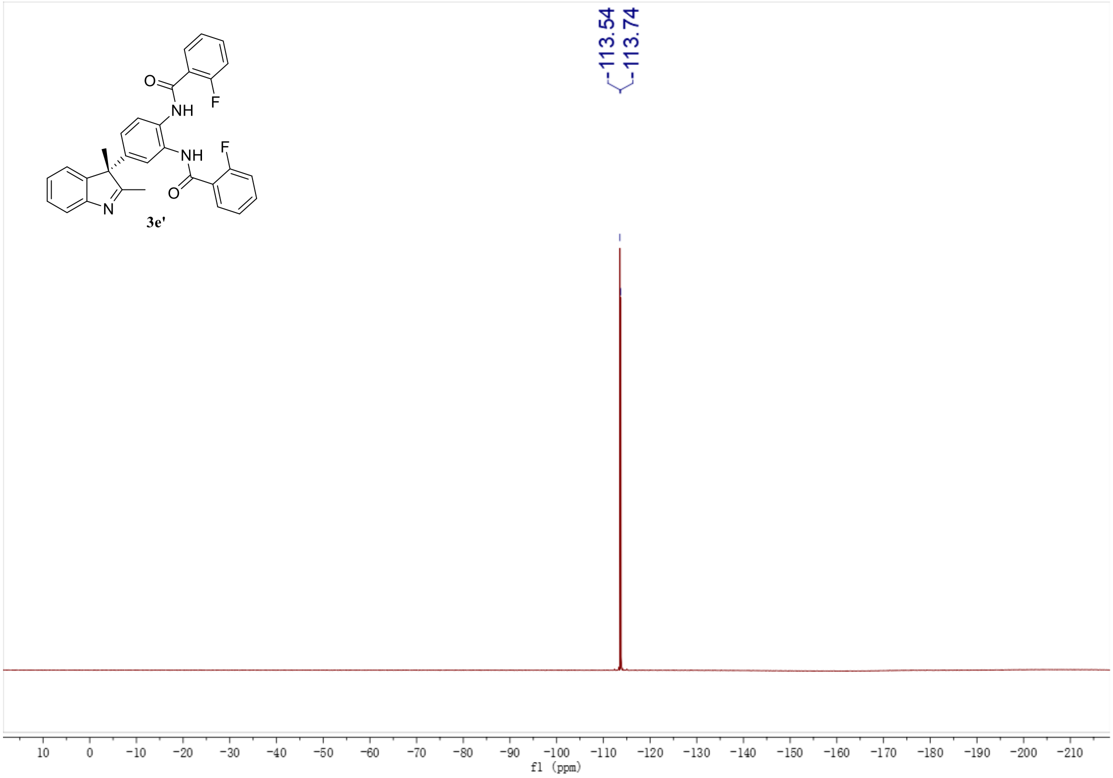

^{1}H NMR spectrum of compound **3f'** ($(CD_3)_2SO$, 400 MHz)

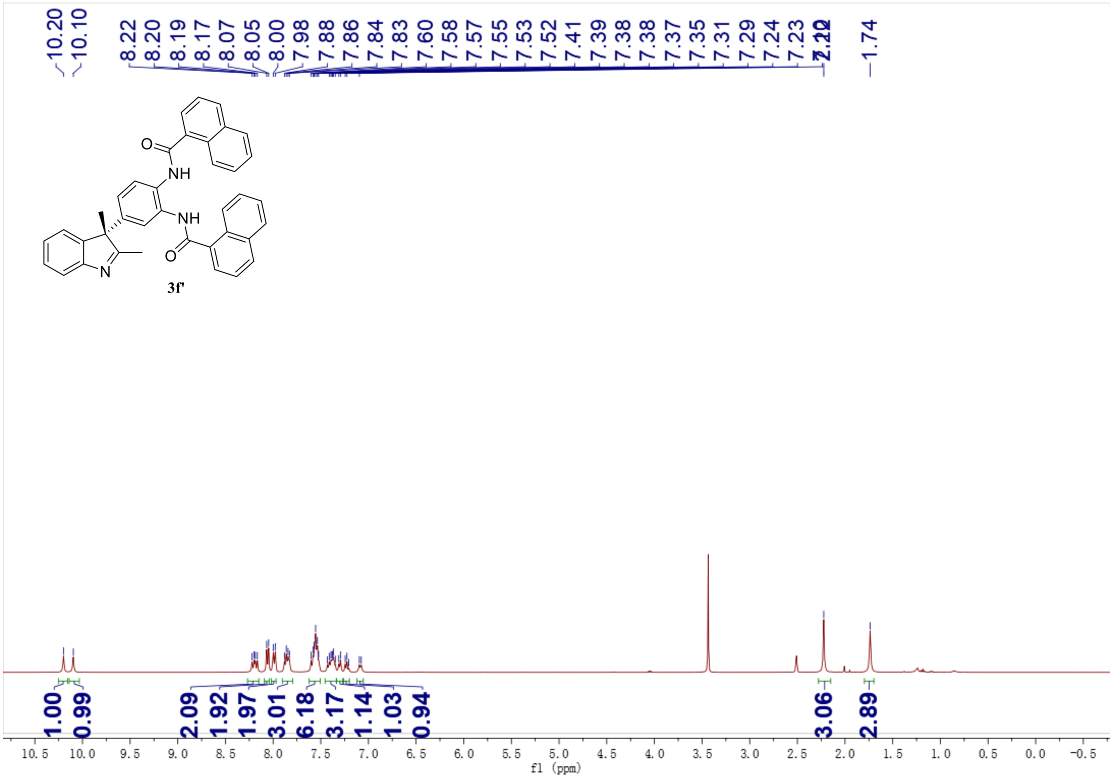

^{13}C NMR spectrum of compound **3f'** ($(CD_3)_2SO$, 100 MHz)

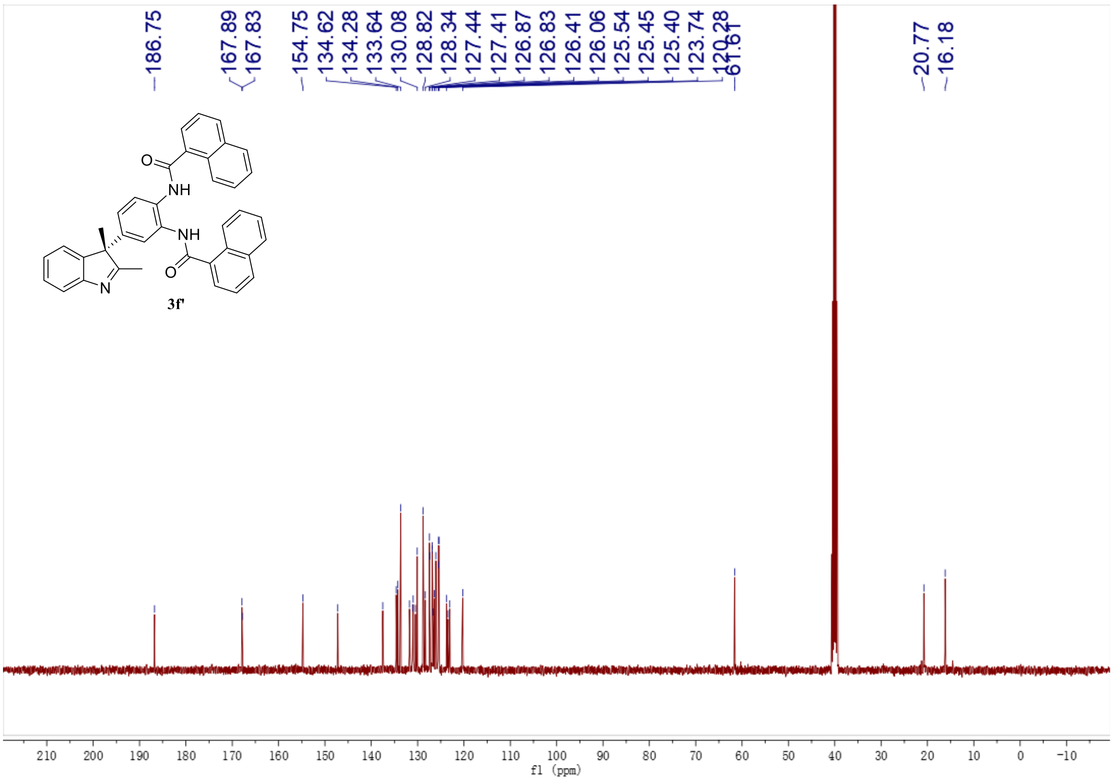

^{1}H NMR spectrum of compound **3g'** ($(CD_3)_2SO$, 400 MHz)

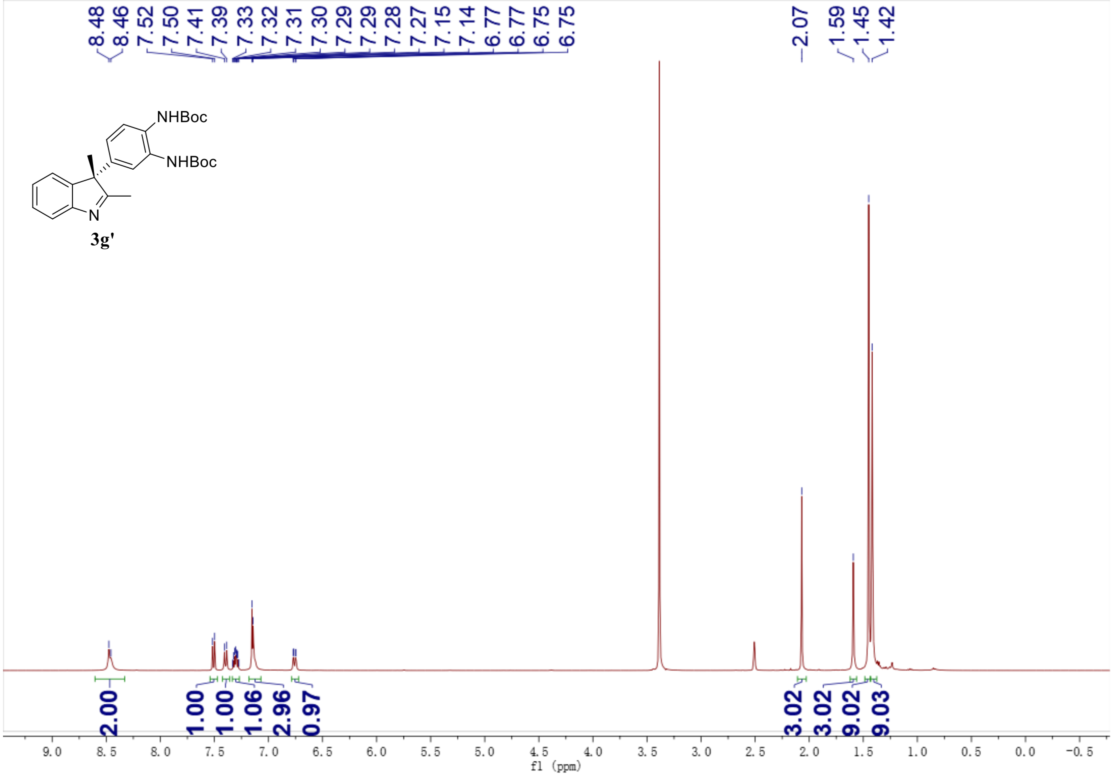

^{13}C NMR spectrum of compound **3g'** ($(CD_3)_2SO$, 100 MHz)

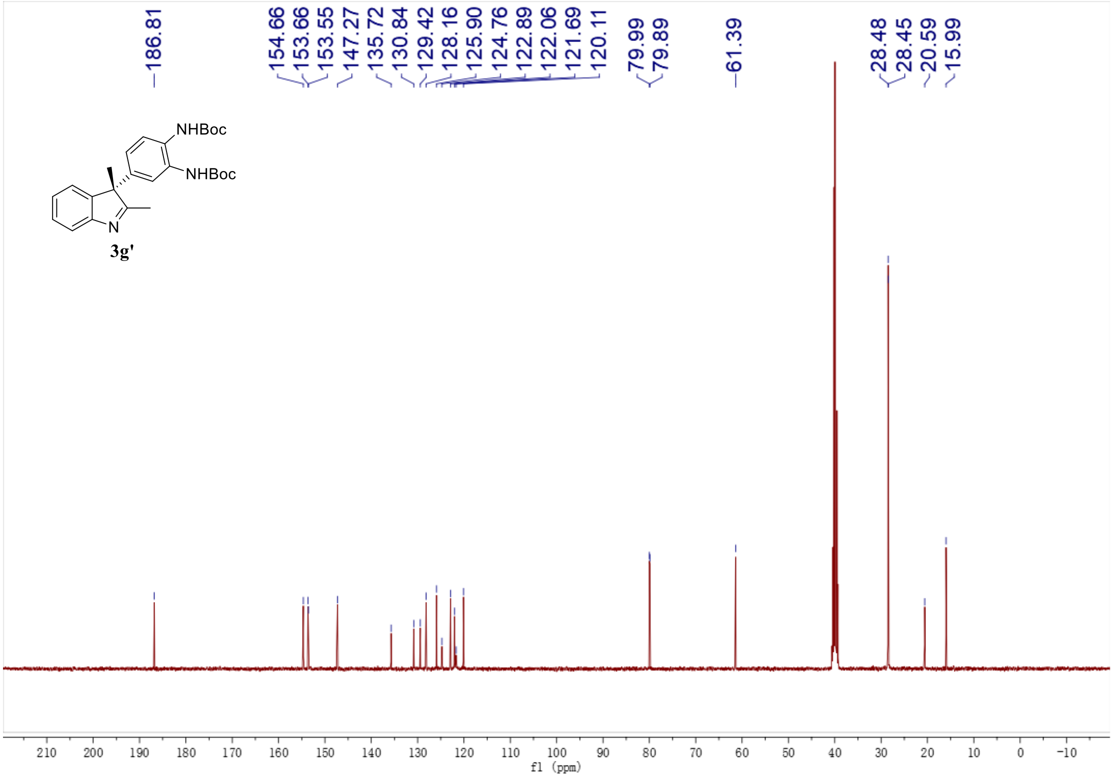

^{1}H NMR spectrum of compound **4a** ($CDCl_3$, 400 MHz)

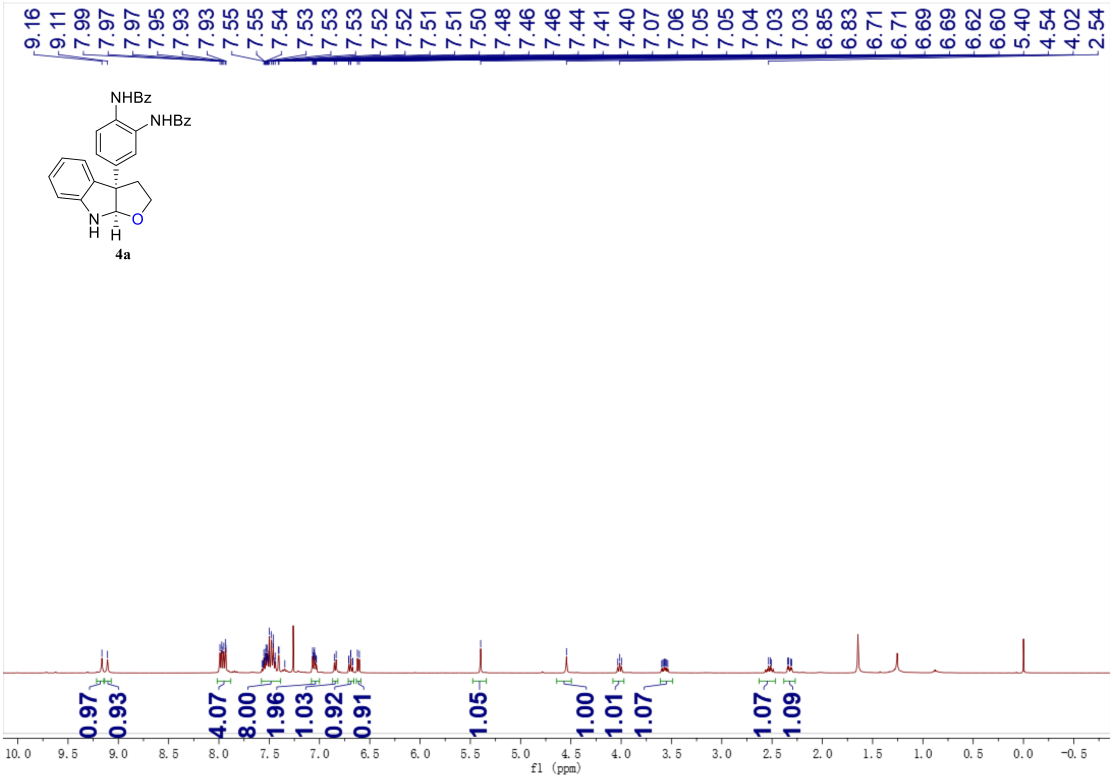

^{13}C NMR spectrum of compound **4a** ($CDCl_3$, 100 MHz)

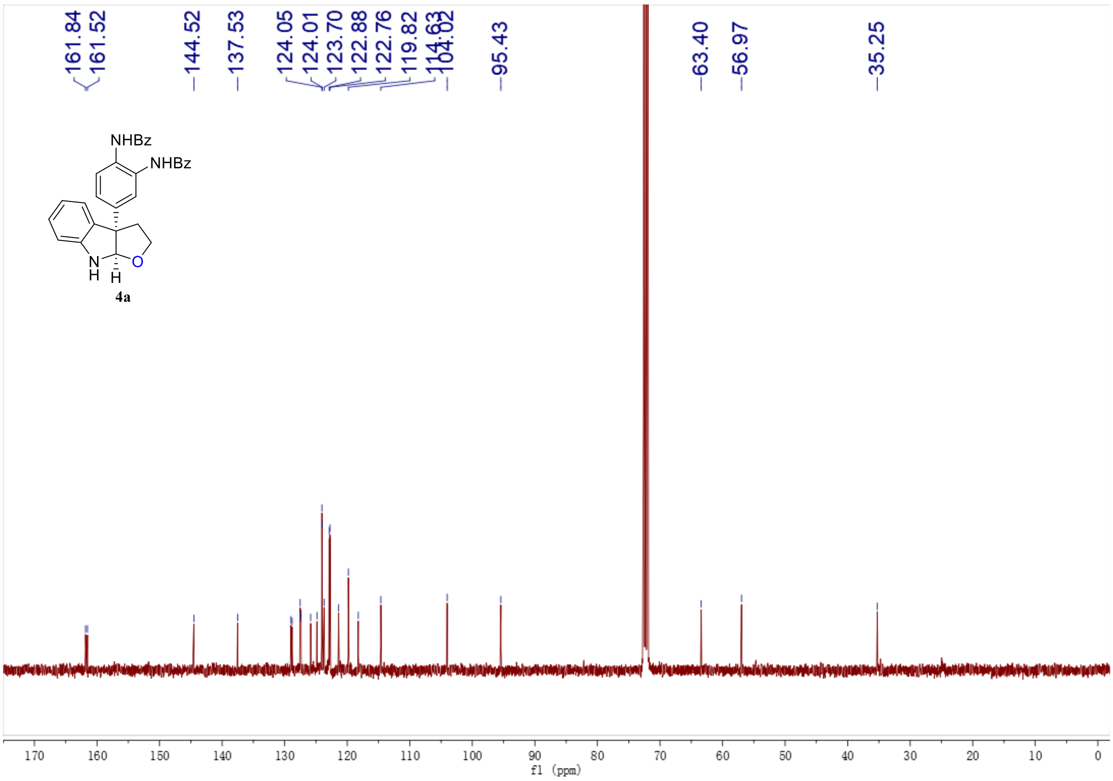

^{1}H NMR spectrum of compound **4b** ($CDCl_3$, 400 MHz)

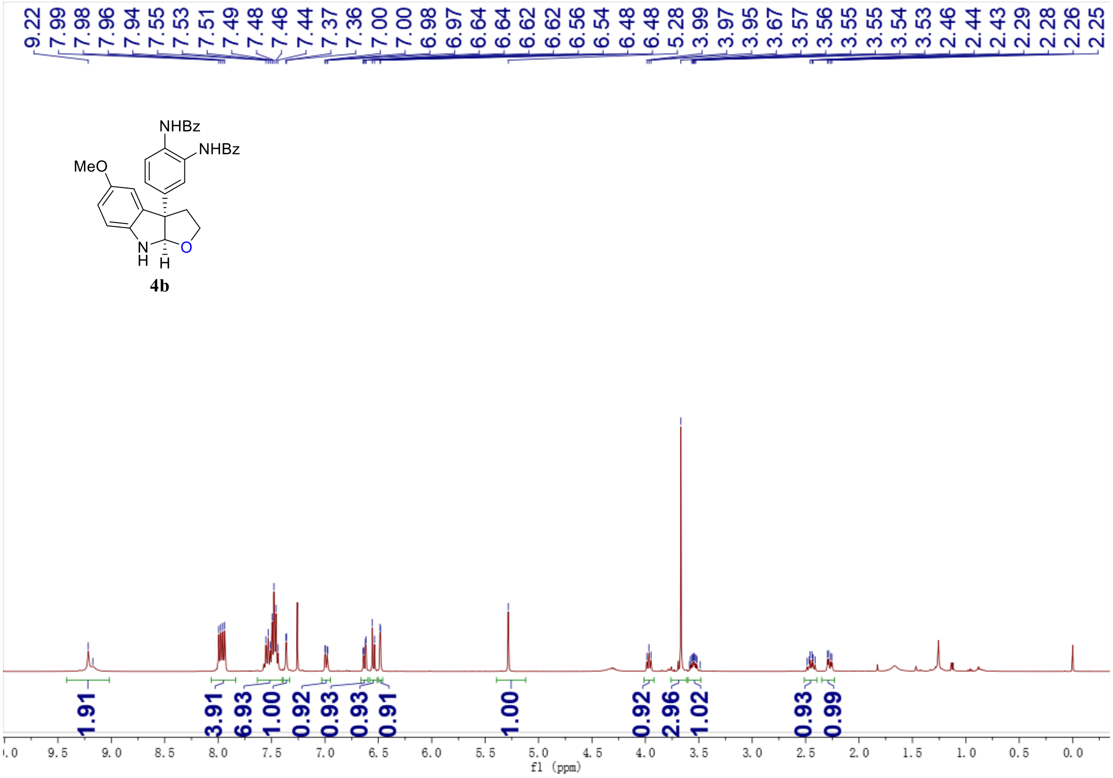

^{13}C NMR spectrum of compound **4b** ($CDCl_3$, 100 MHz)

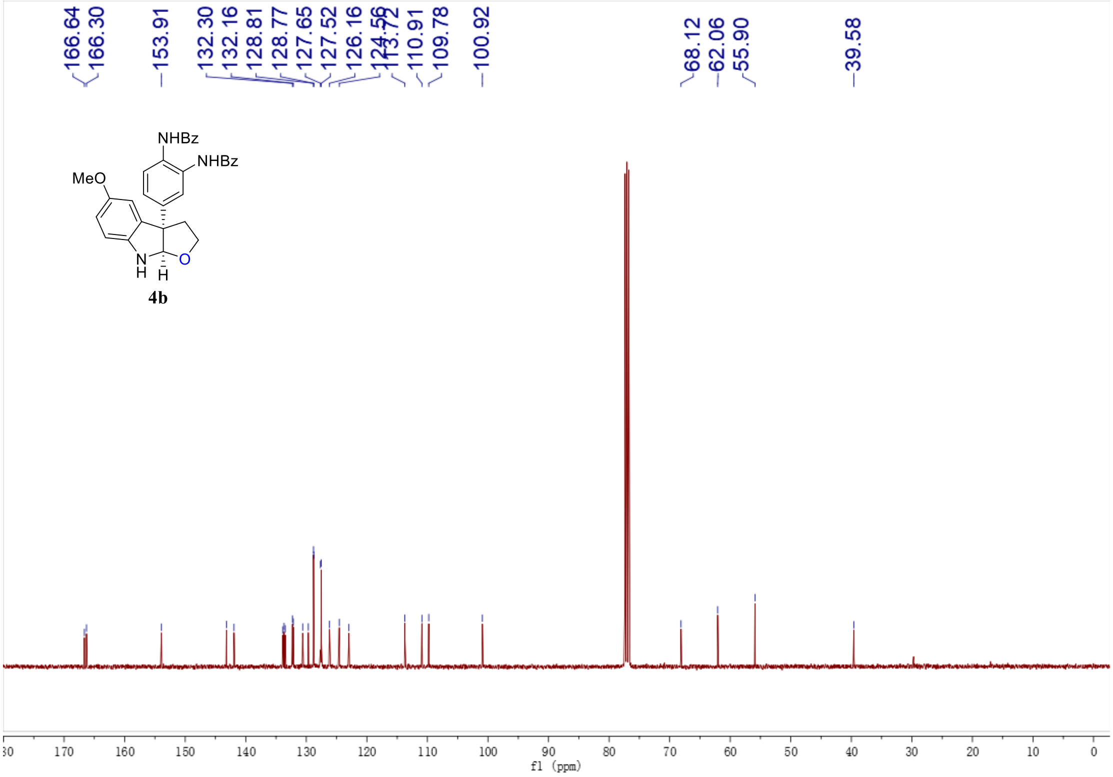

^{1}H NMR spectrum of compound **4c** ((CD_3)$_2$SO, 400 MHz)

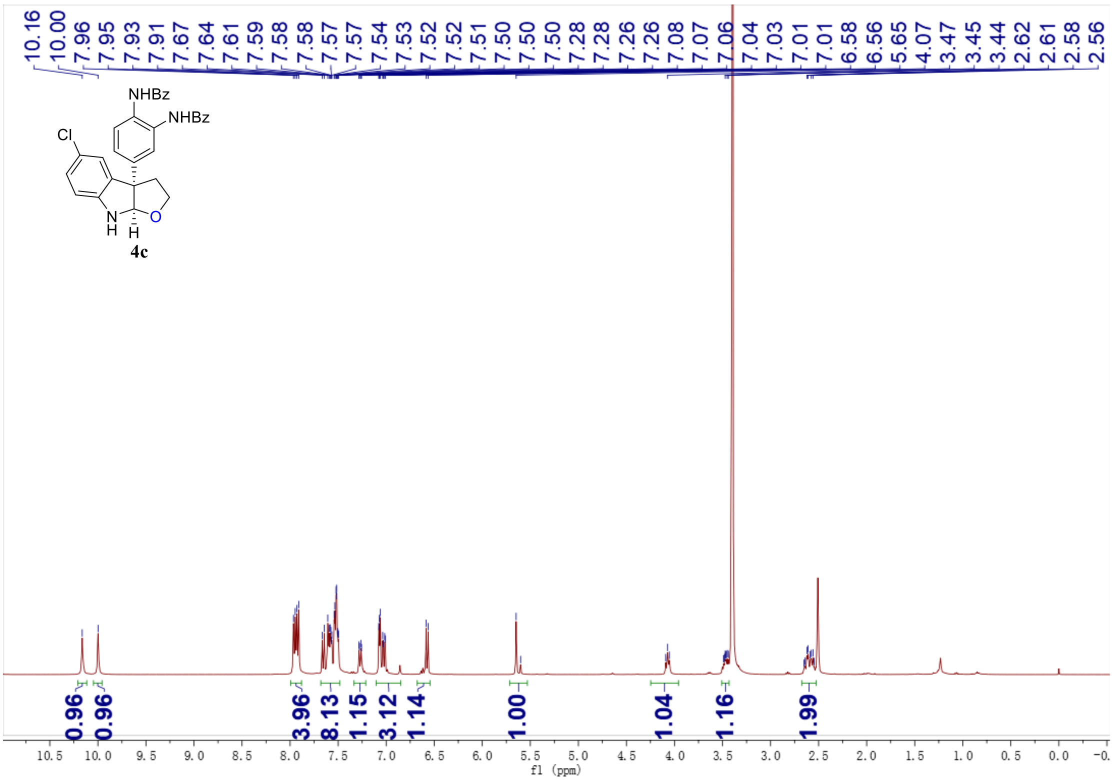

^{13}C NMR spectrum of compound **4c** ((CD_3)$_2$SO, 100 MHz)

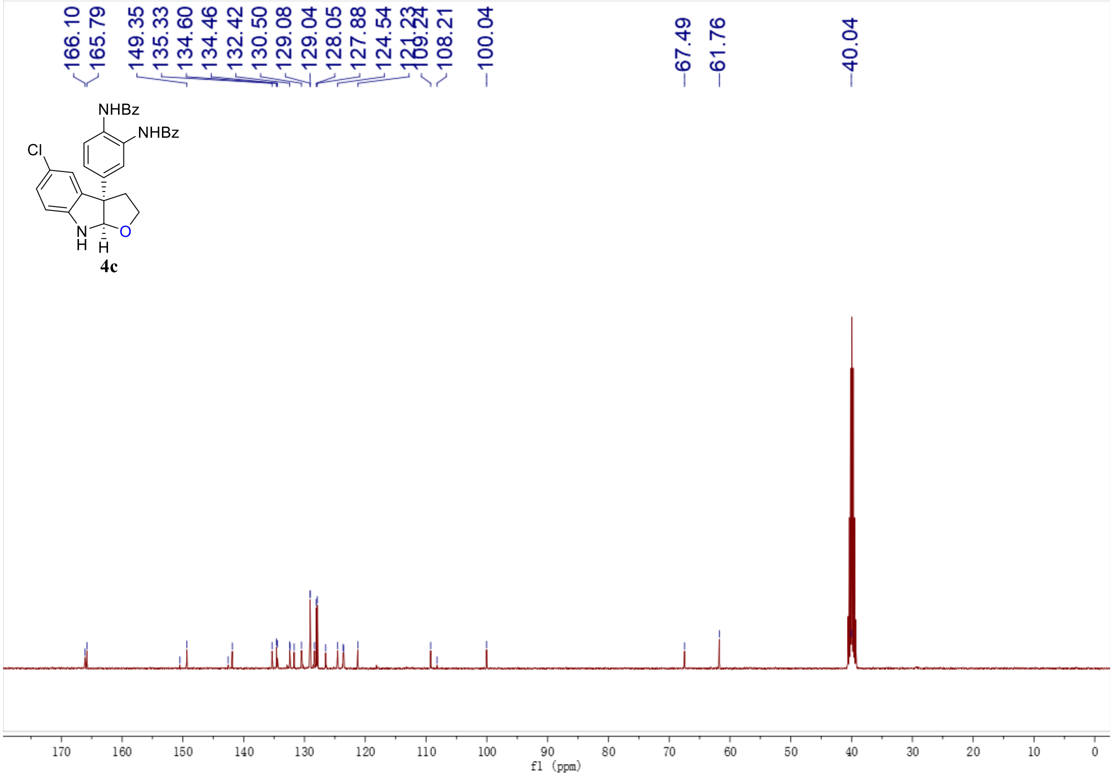

^{1}H NMR spectrum of compound **4d** ($(CD_3)_2SO$, 400 MHz)

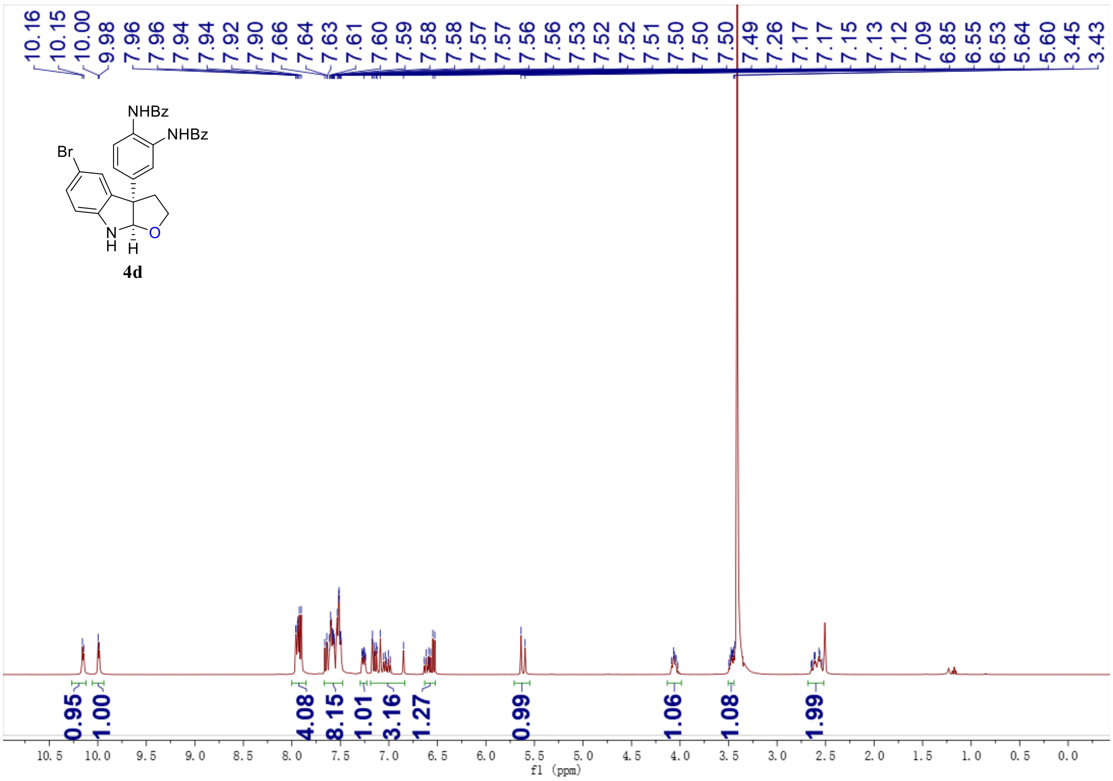

^{13}C NMR spectrum of compound **4d** ($(CD_3)_2SO$, 100 MHz)

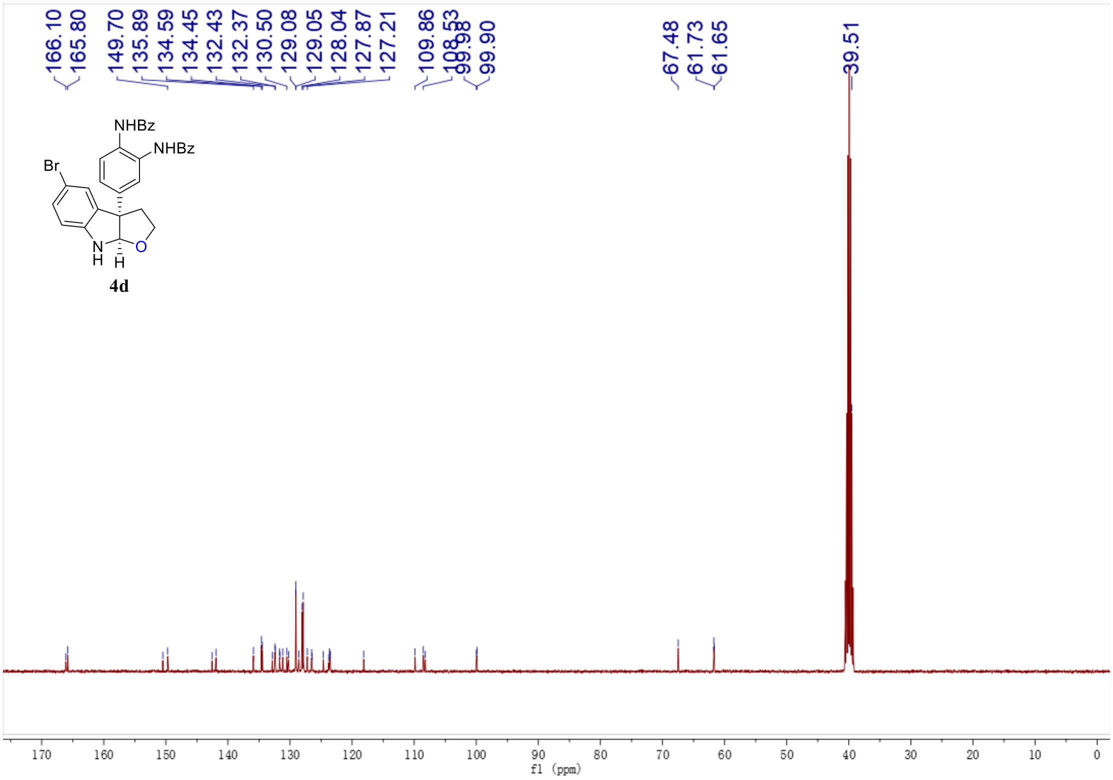

^{1}H NMR spectrum of compound **4f** ($CDCl_3$, 400 MHz)

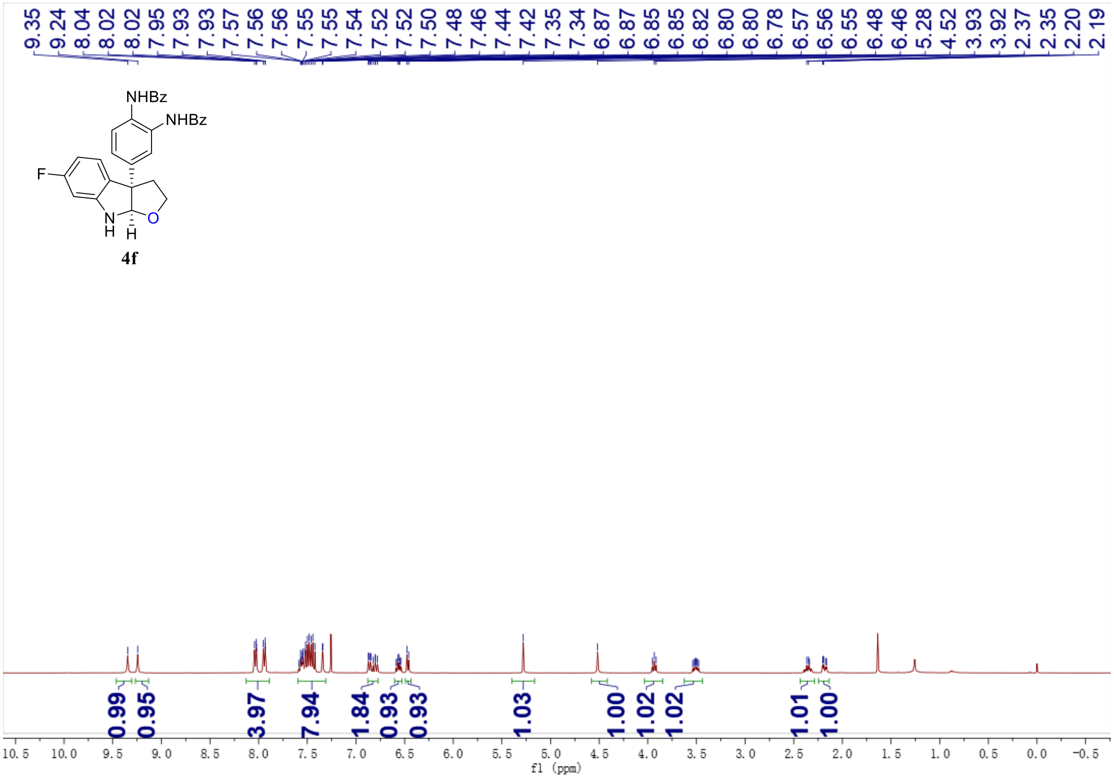

^{13}C NMR spectrum of compound **4f** ($CDCl_3$, 100 MHz)

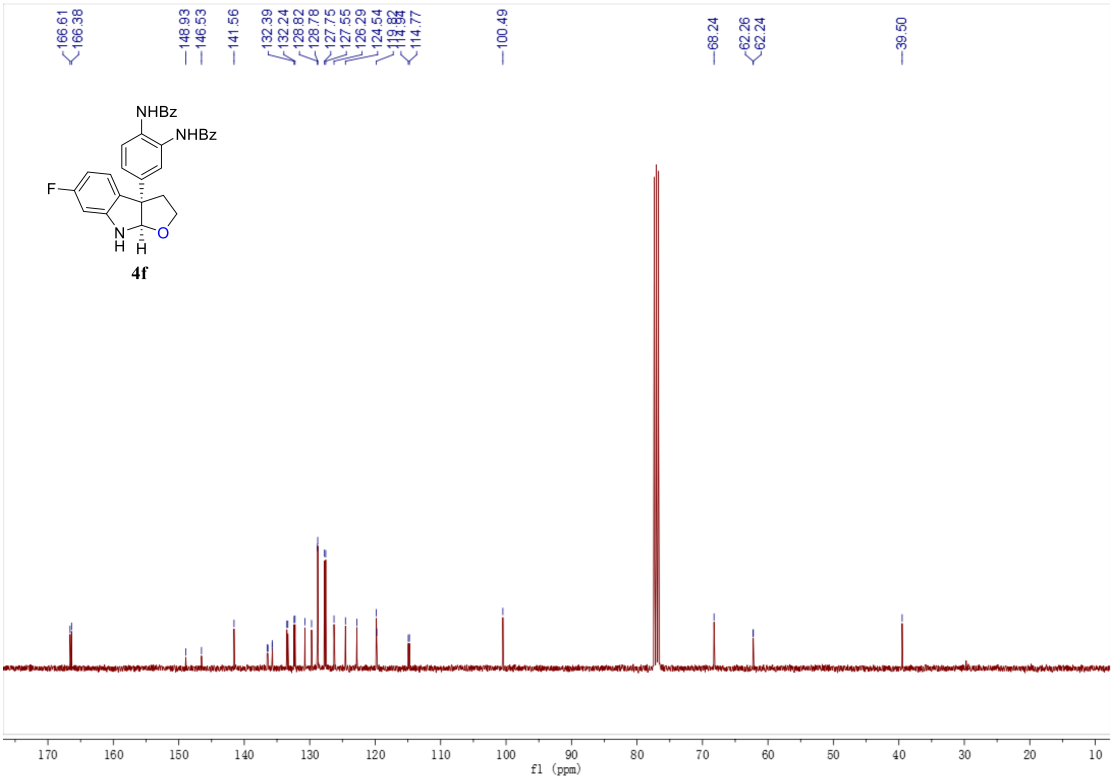

^{19}F NMR spectrum of compound **4f** ($CDCl_3$, 376 MHz)

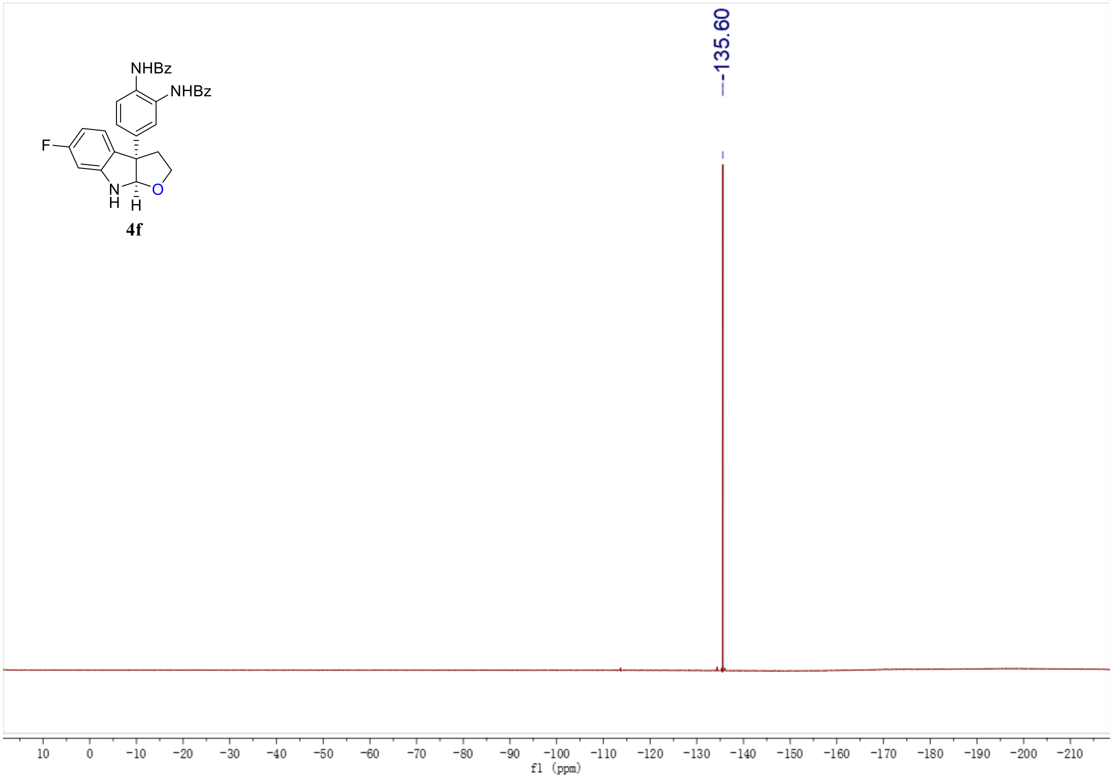

^{1}H NMR spectrum of compound **4g** ($CDCl_3$, 400 MHz)

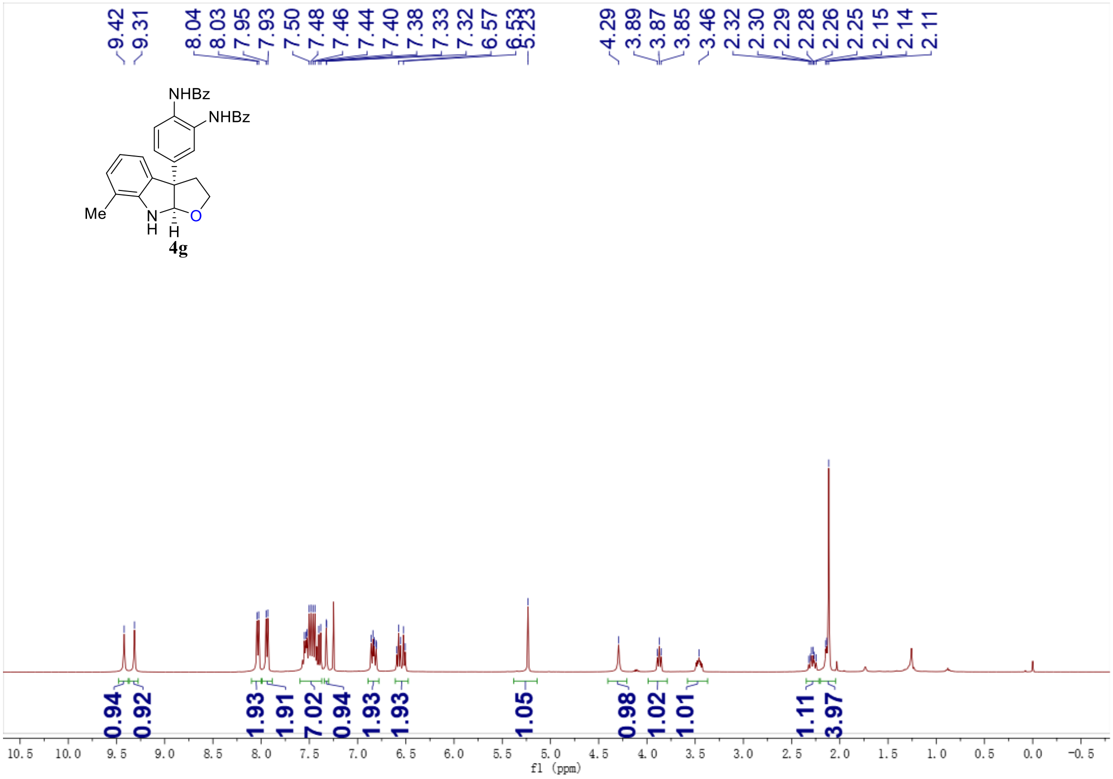

^{13}C NMR spectrum of compound **4g** ($CDCl_3$, 100 MHz)

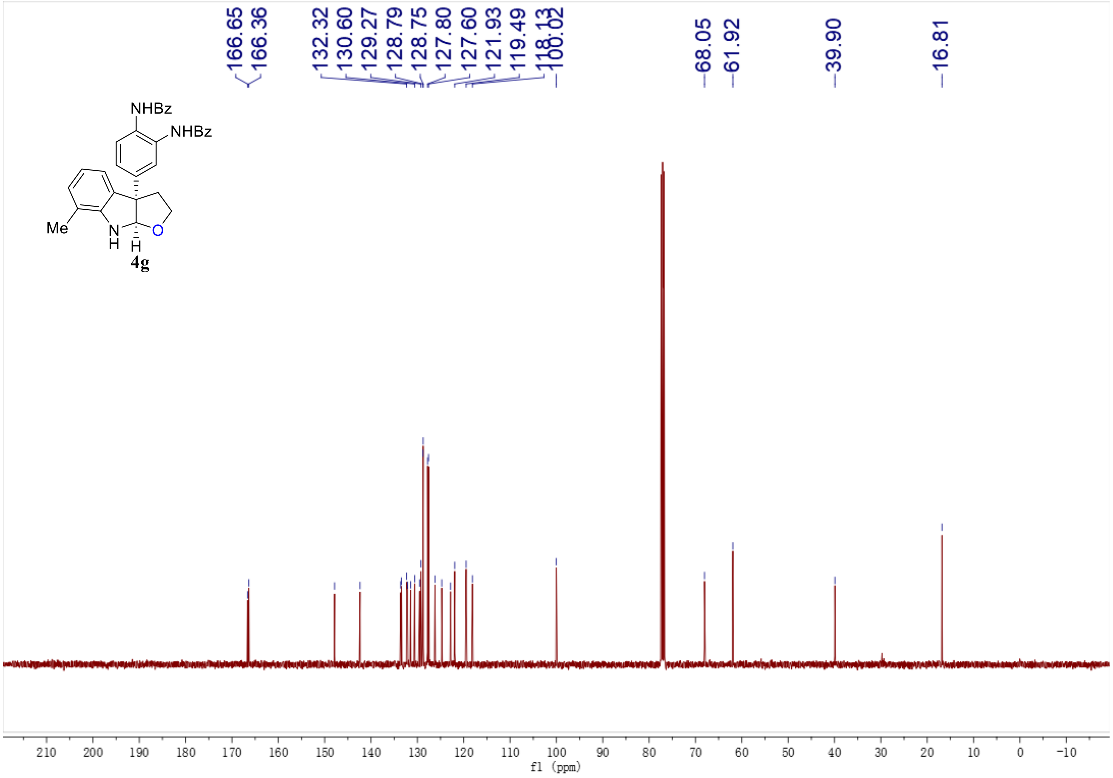

^{1}H NMR spectrum of compound **4h** ($CDCl_3$, 400 MHz)

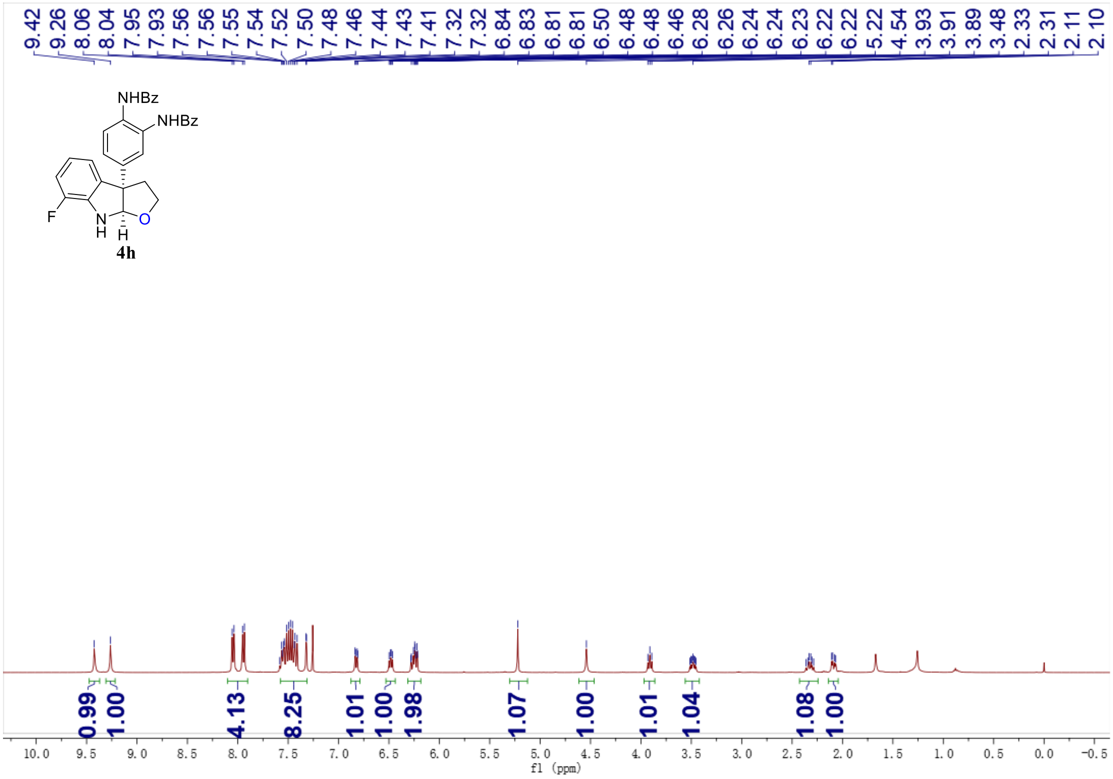

^{13}C NMR spectrum of compound **4h** ($CDCl_3$, 100 MHz)

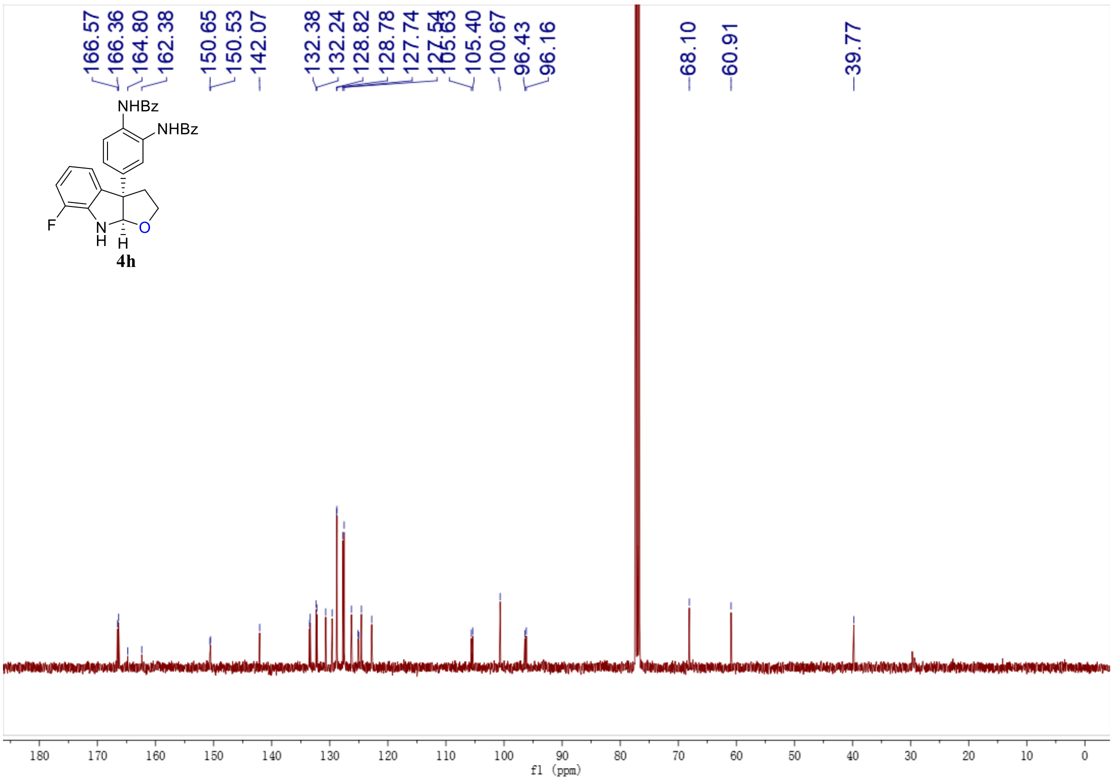

^{19}F NMR spectrum of compound **4h** ($CDCl_3$, 376 MHz)

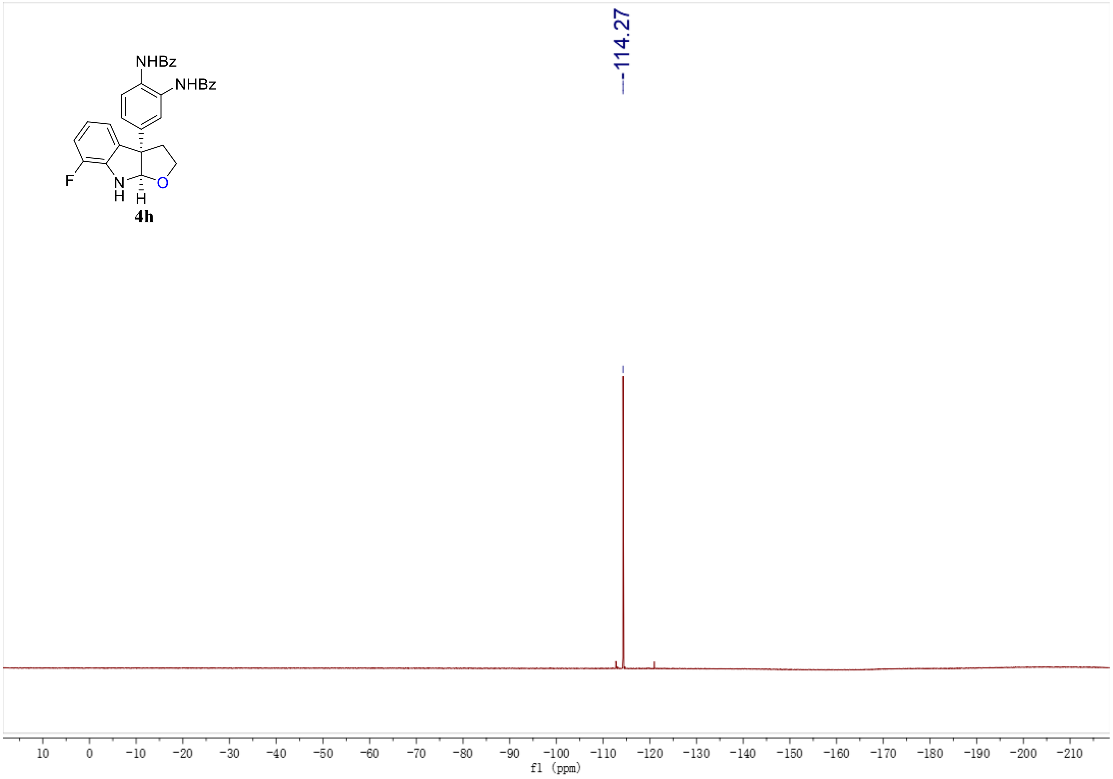

^{1}H NMR spectrum of compound **4i** ($CDCl_3$, 400 MHz)

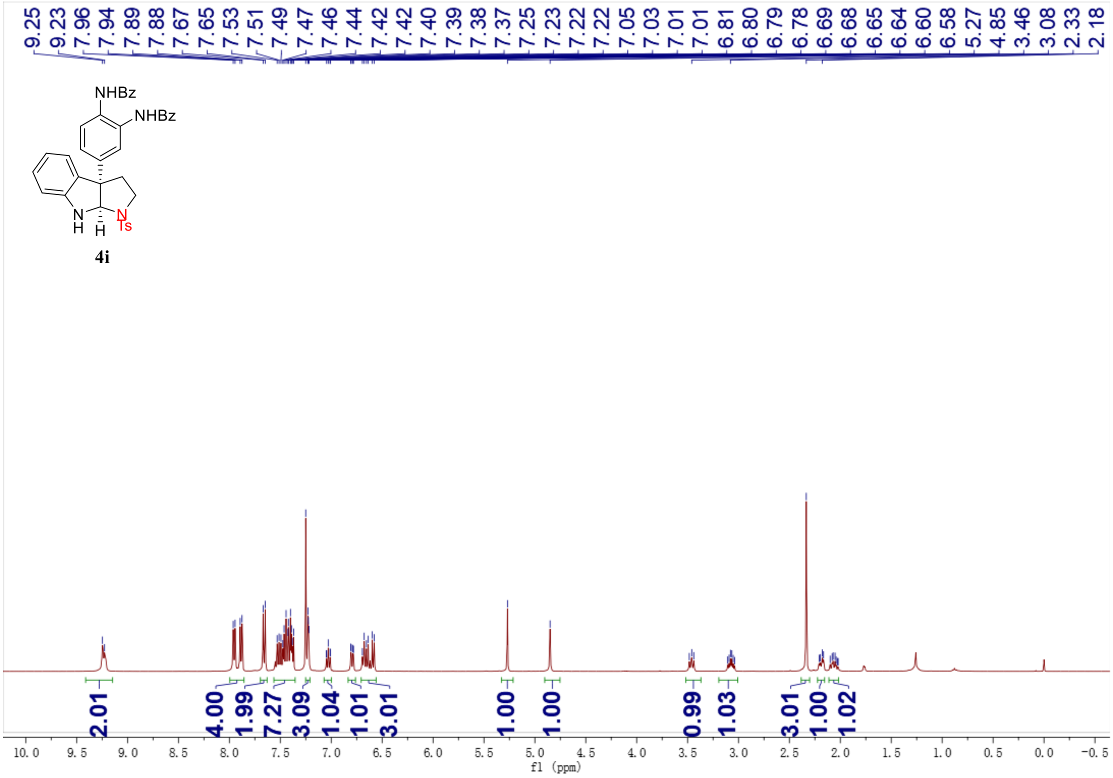

^{13}C NMR spectrum of compound **4i** ($CDCl_3$, 100 MHz)

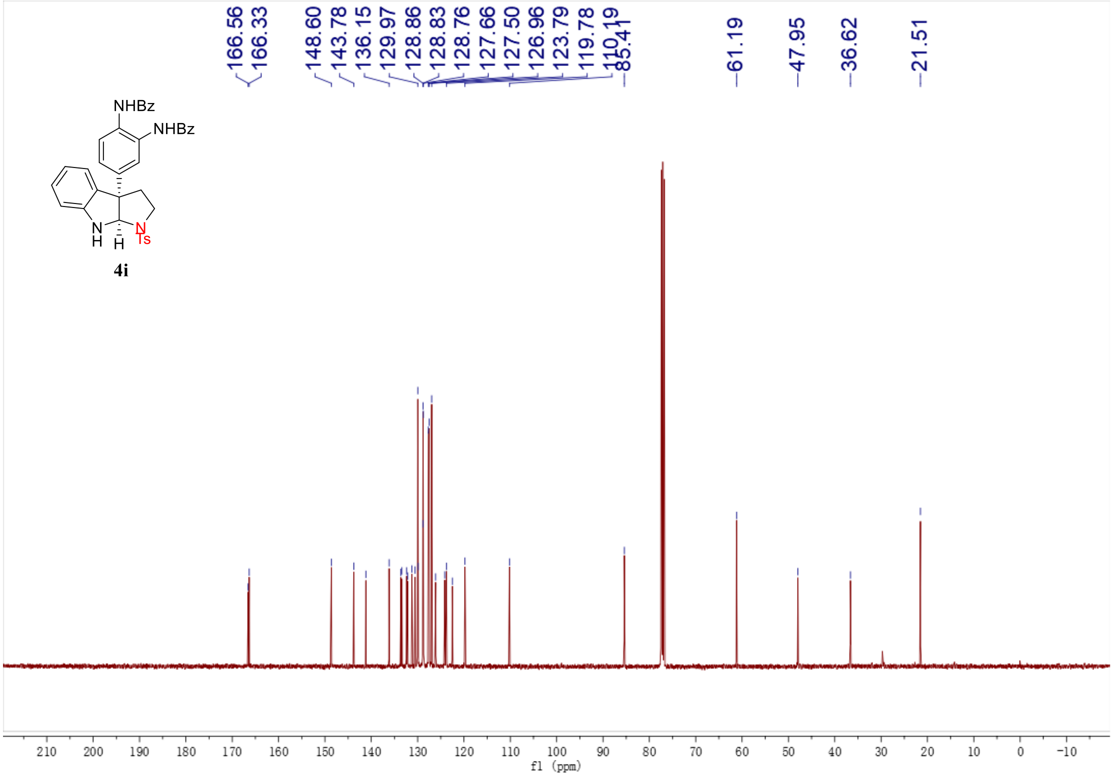

^{1}H NMR spectrum of compound **4j** ($(CD_3)_2SO$, 400 MHz)

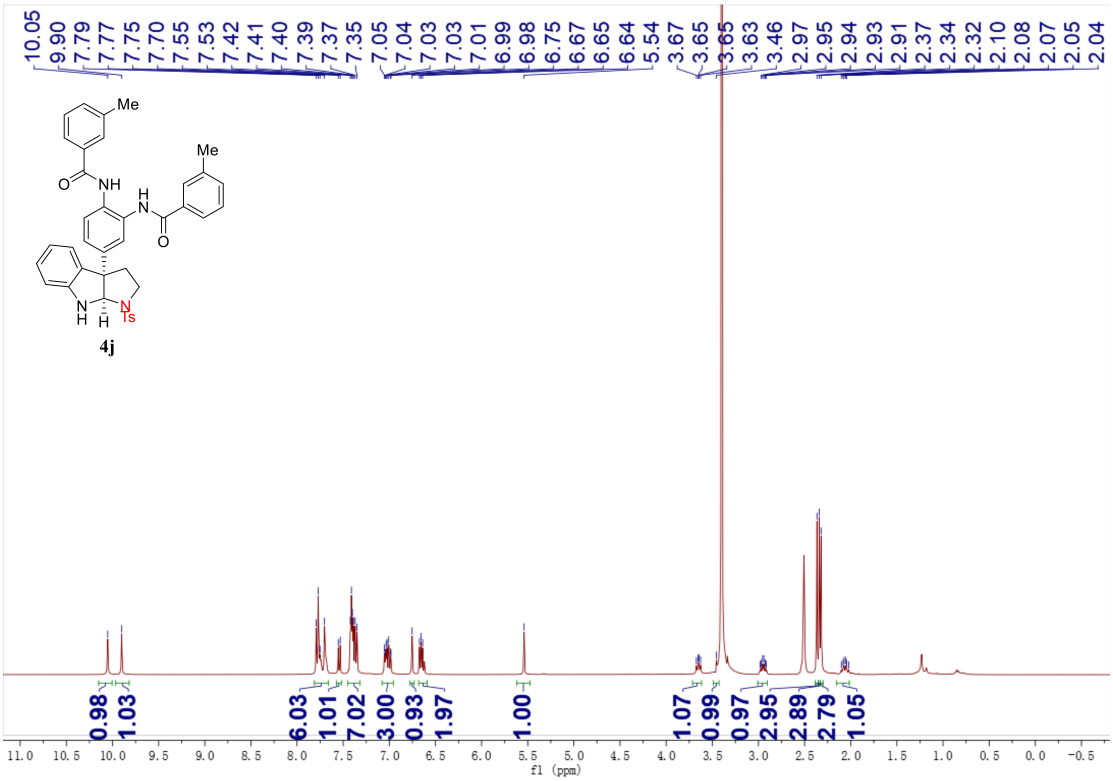

^{13}C NMR spectrum of compound **4j** ($(CD_3)_2SO$, 100 MHz)

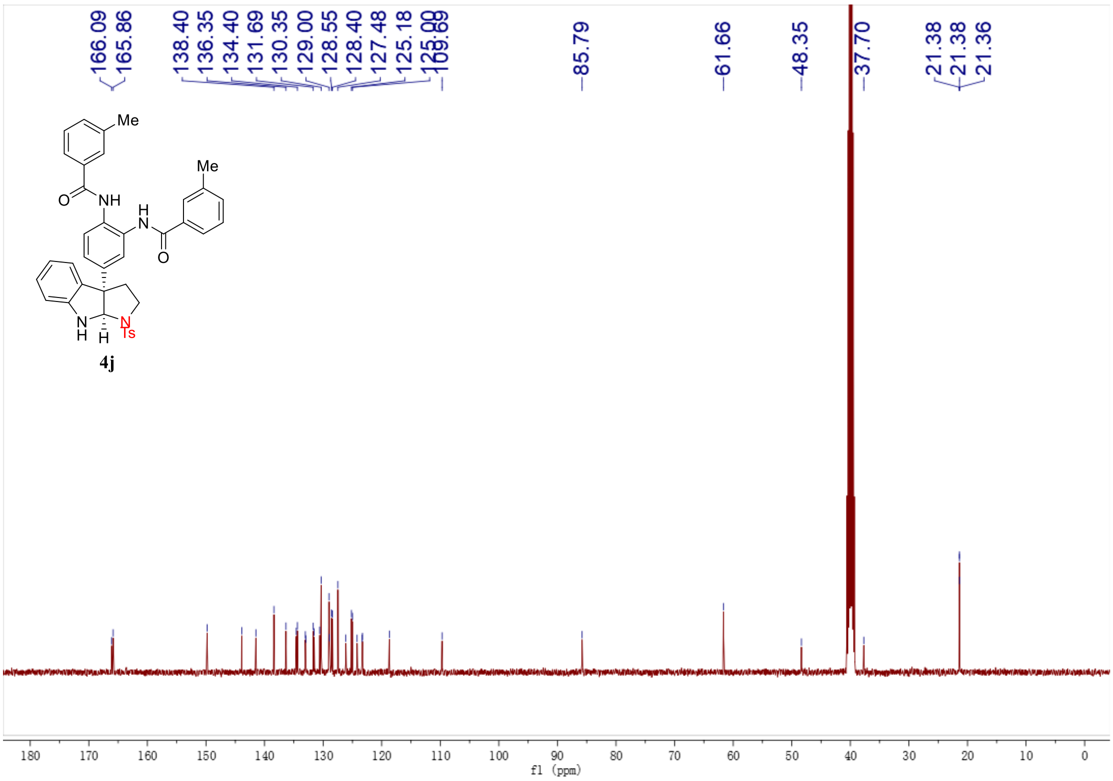

^{1}H NMR spectrum of compound **4k** ($(CD_3)_2SO$, 400 MHz)

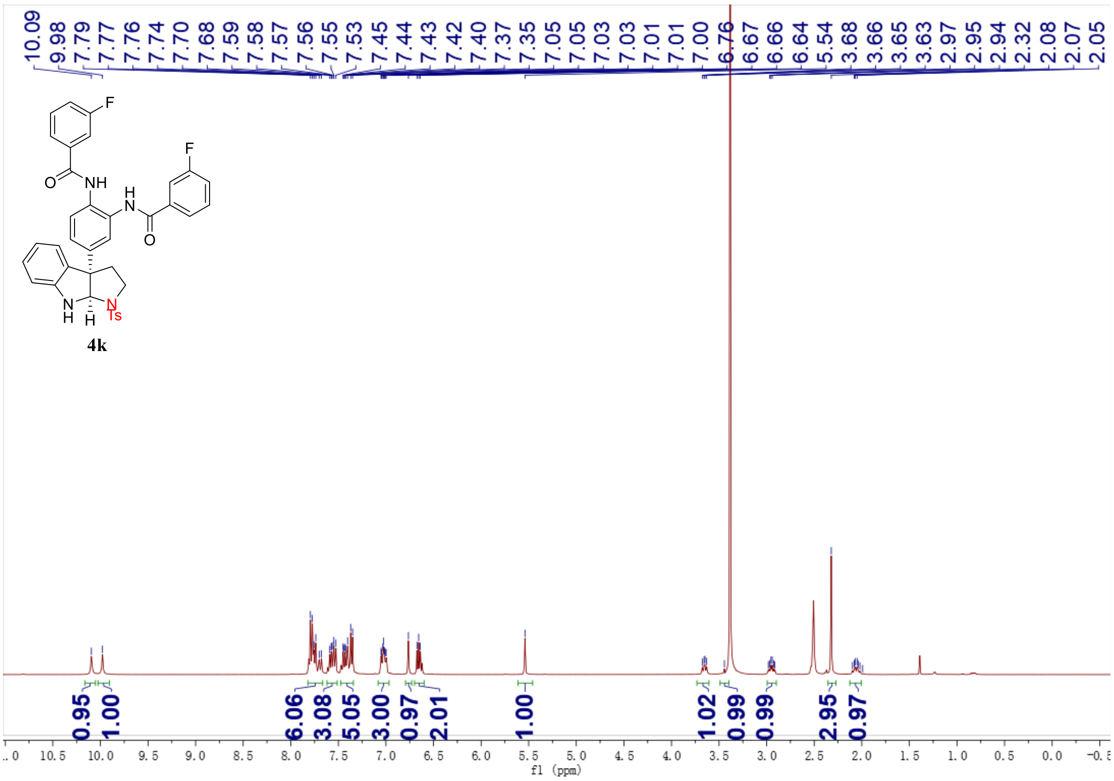

^{13}C NMR spectrum of compound **4k** ($(CD_3)_2SO$, 100 MHz)

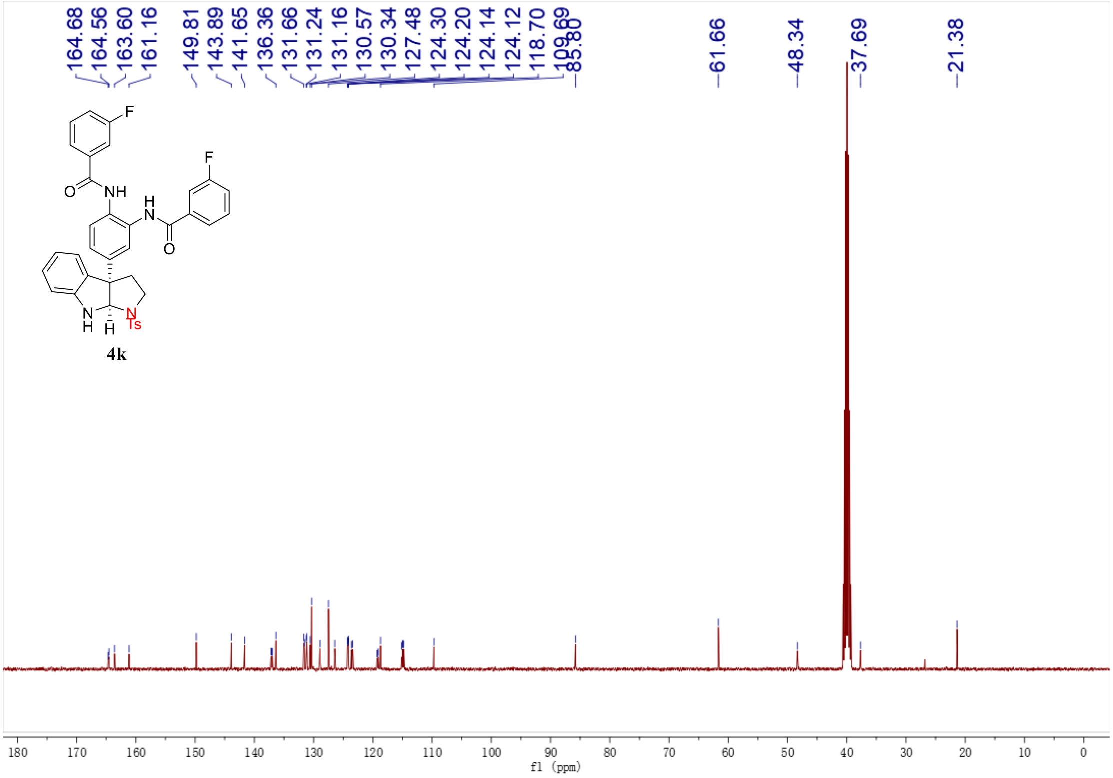

^{19}F NMR spectrum of compound **4k** ($(CD_3)_2SO$, 376 MHz)

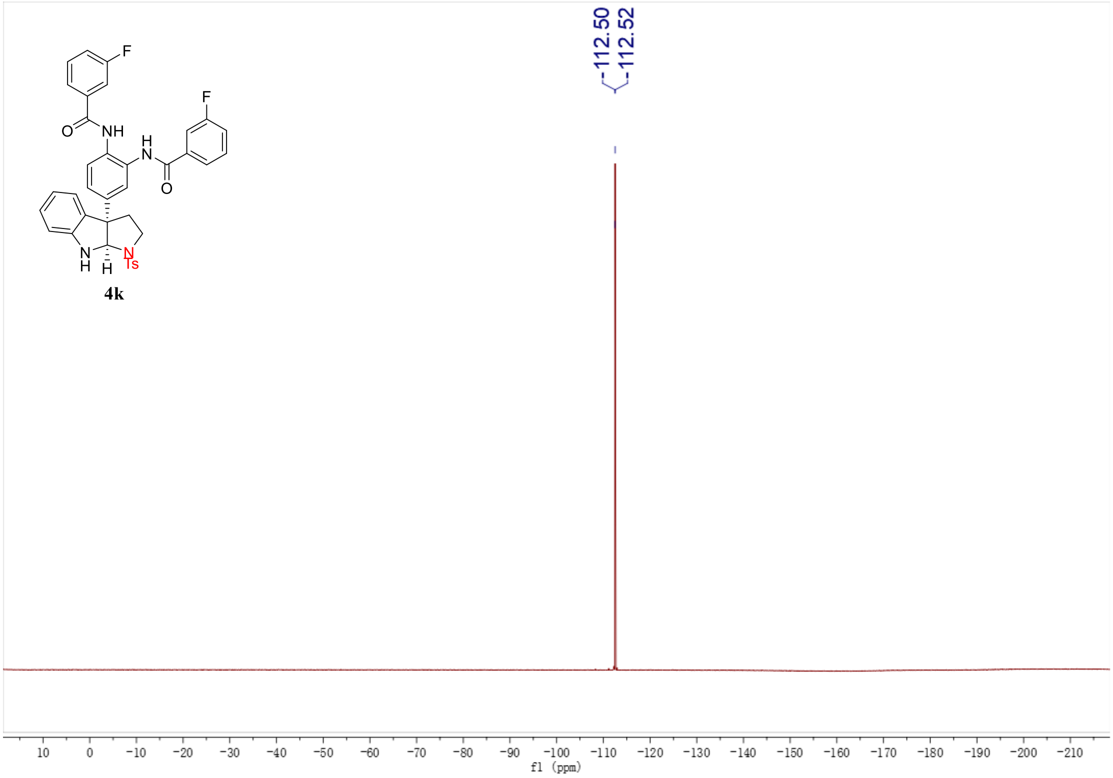

^{1}H NMR spectrum of compound **4l** ((CD$_3$)$_2$SO, 400 MHz)

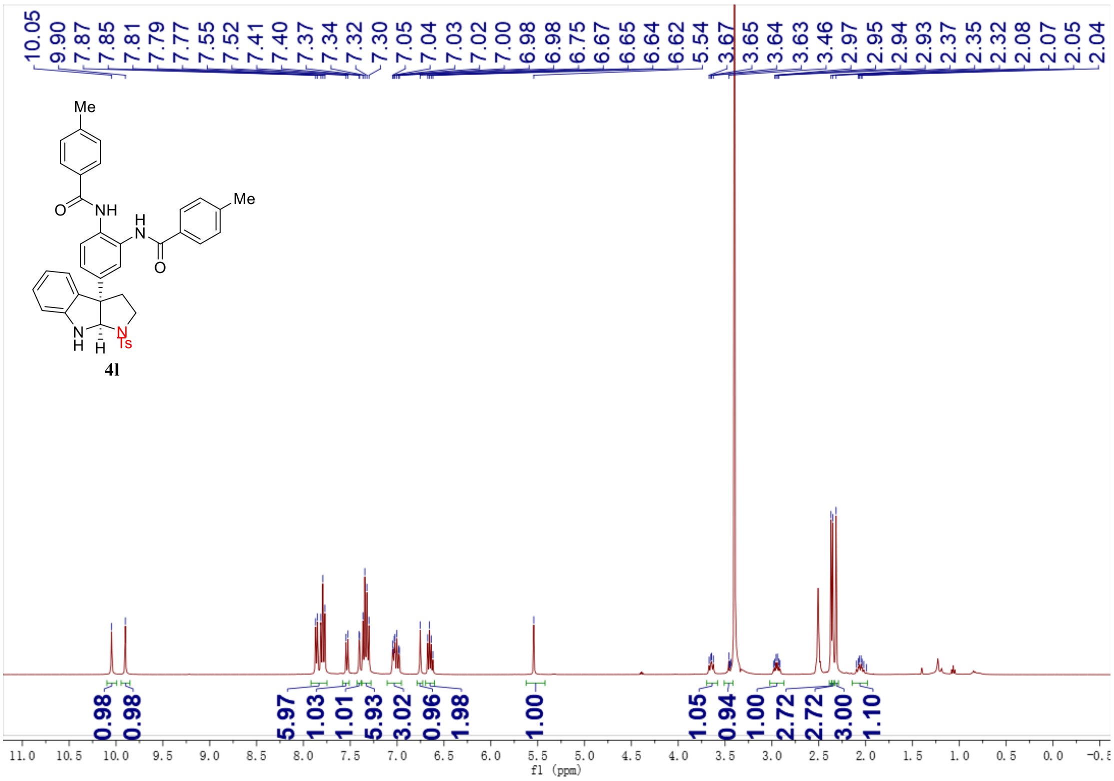

^{13}C NMR spectrum of compound **4l** ((CD$_3$)$_2$SO, 100 MHz)

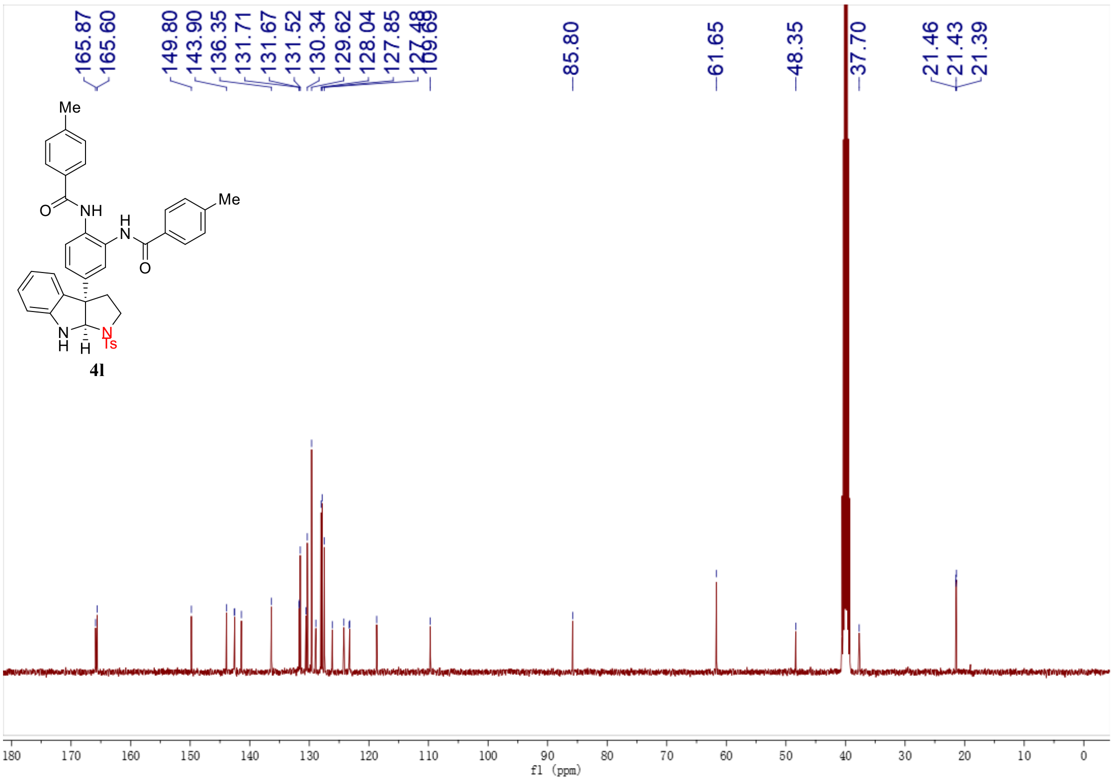

^{1}H NMR spectrum of compound **4m** ($(CD_3)_2SO$, 400 MHz)

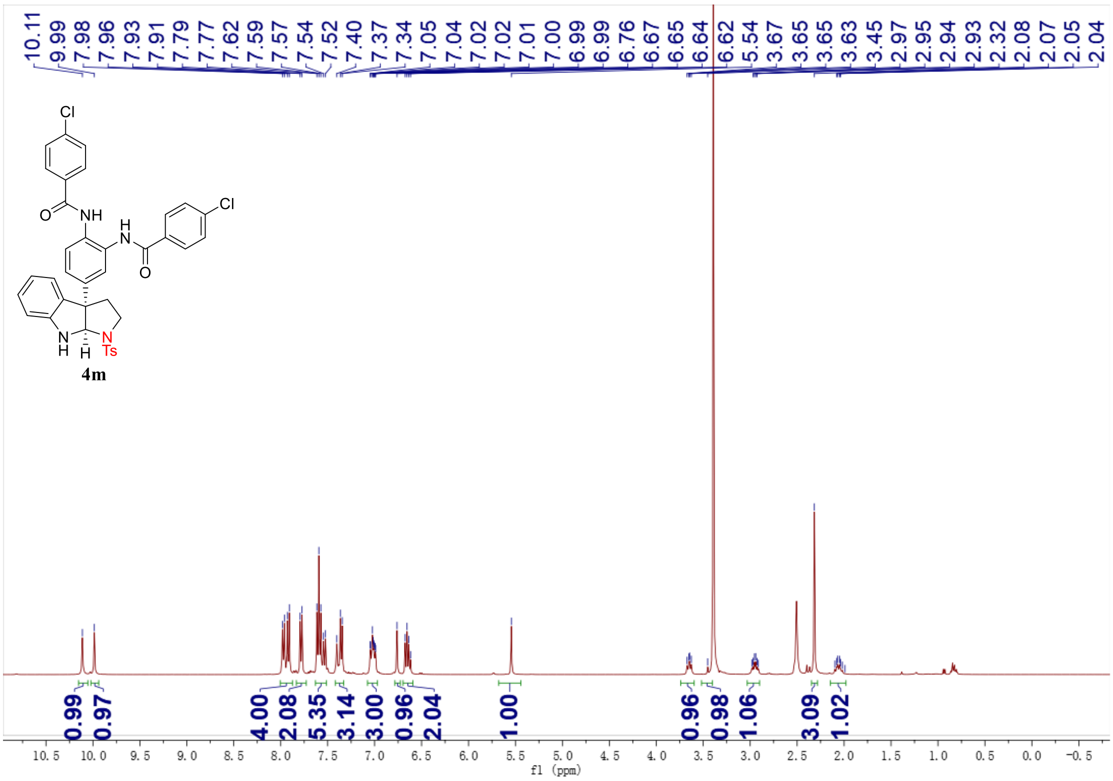

^{13}C NMR spectrum of compound **4m** ($(CD_3)_2SO$, 100 MHz)

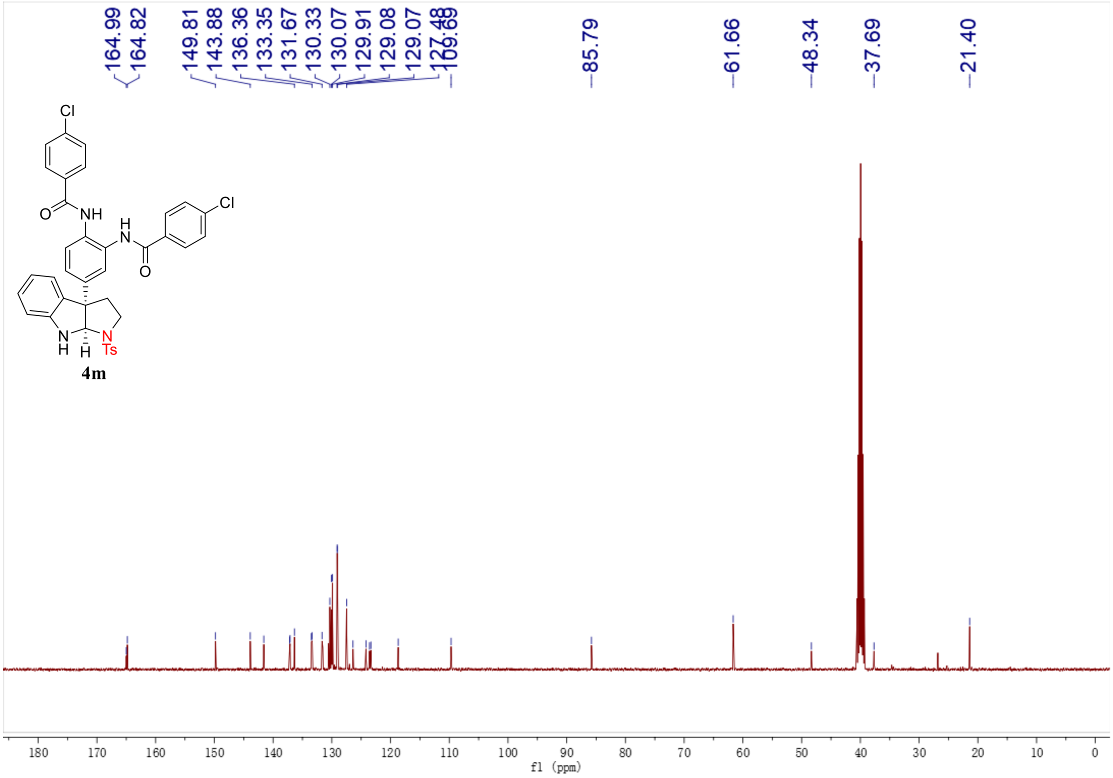

^{1}H NMR spectrum of compound **5a** (DMSO, 400 MHz)

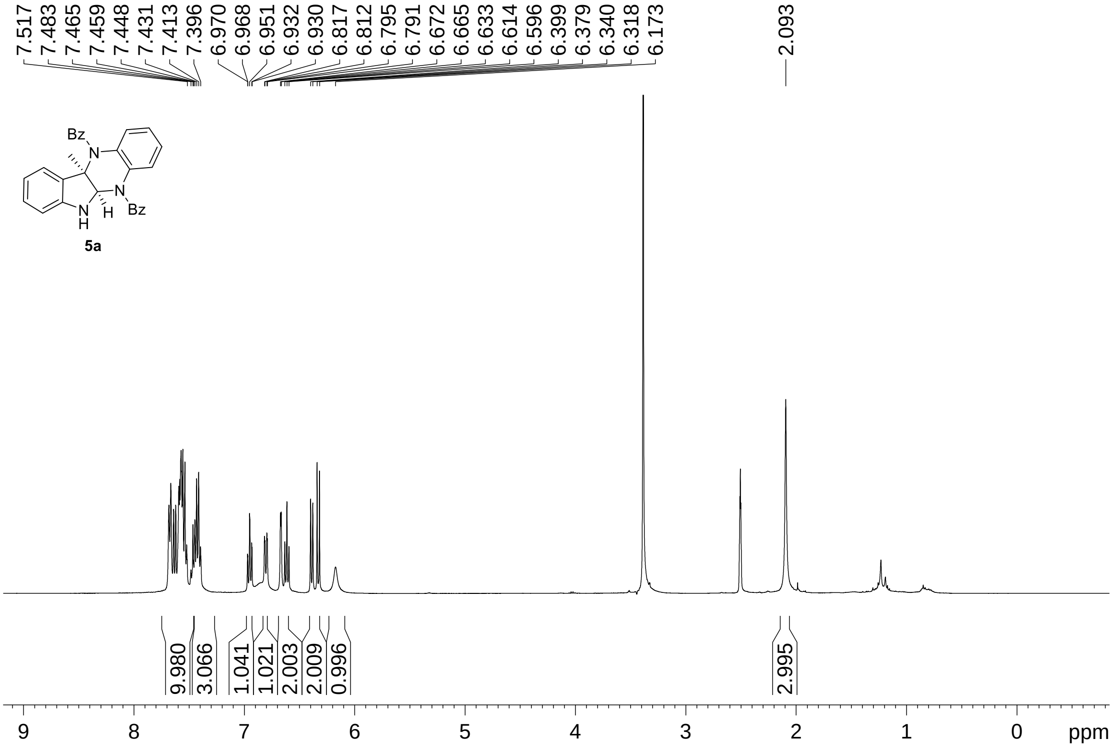

^{13}C NMR spectrum of compound **5a** (DMSO, 100 MHz)

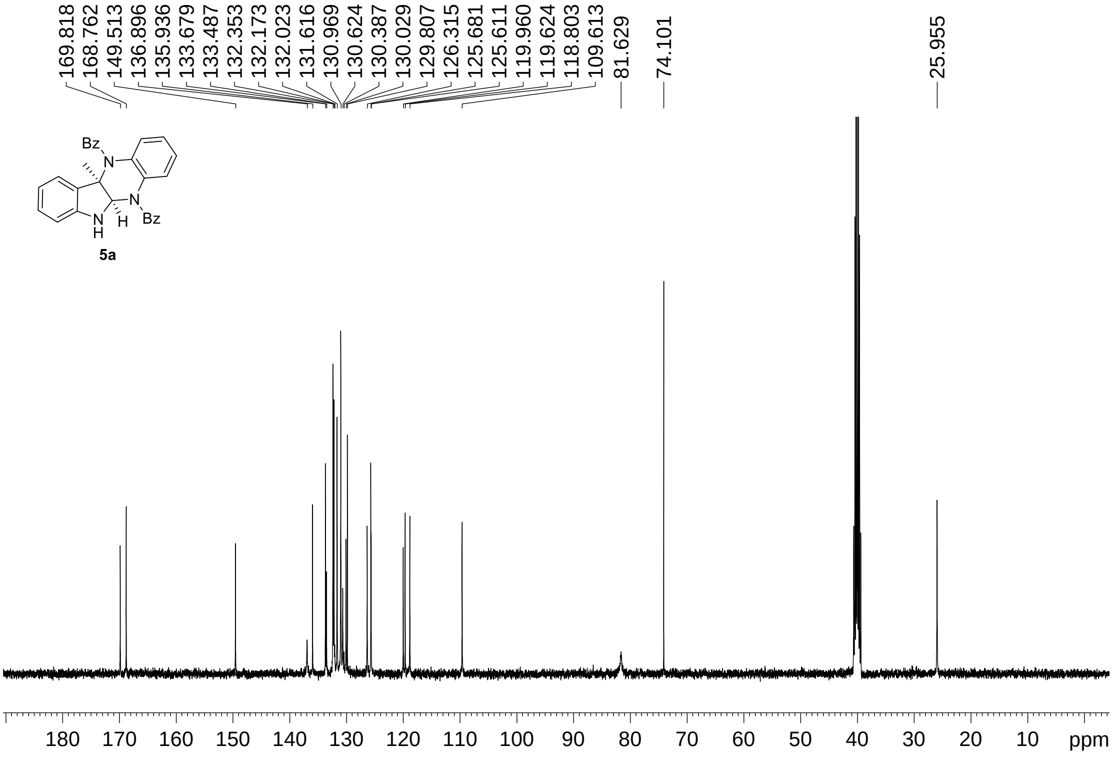

^{1}H NMR spectrum of compound **5b** (DMSO, 400 MHz)

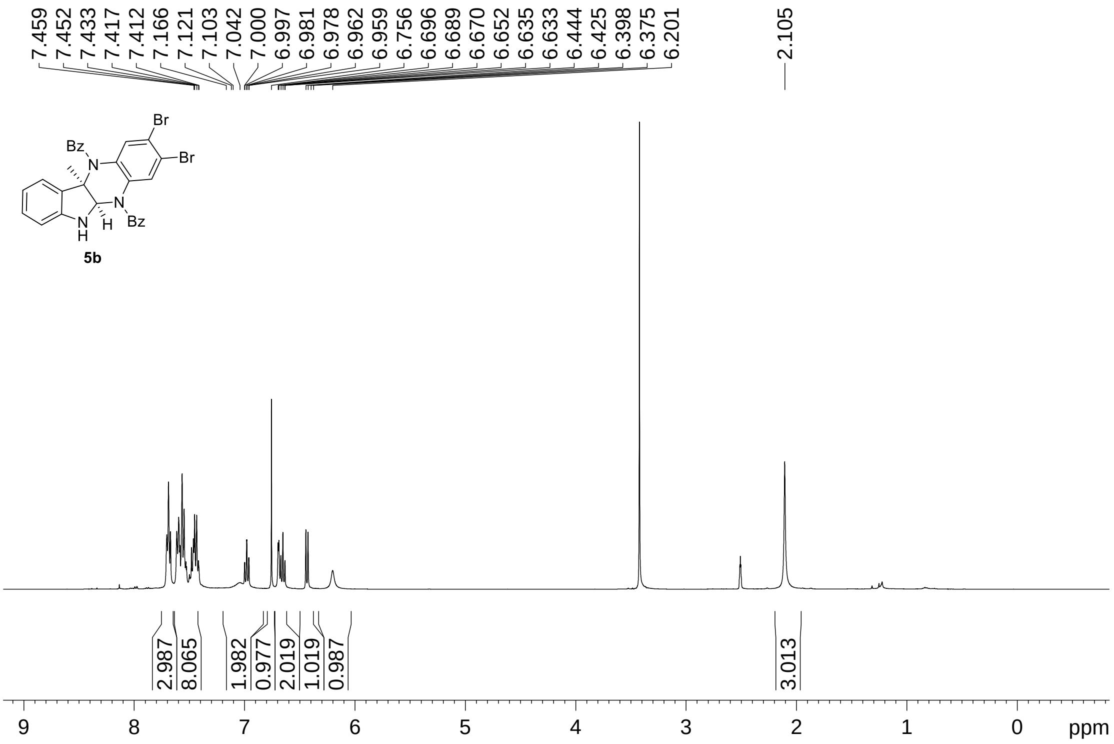

^{13}C NMR spectrum of compound **5b** (DMSO, 100 MHz)

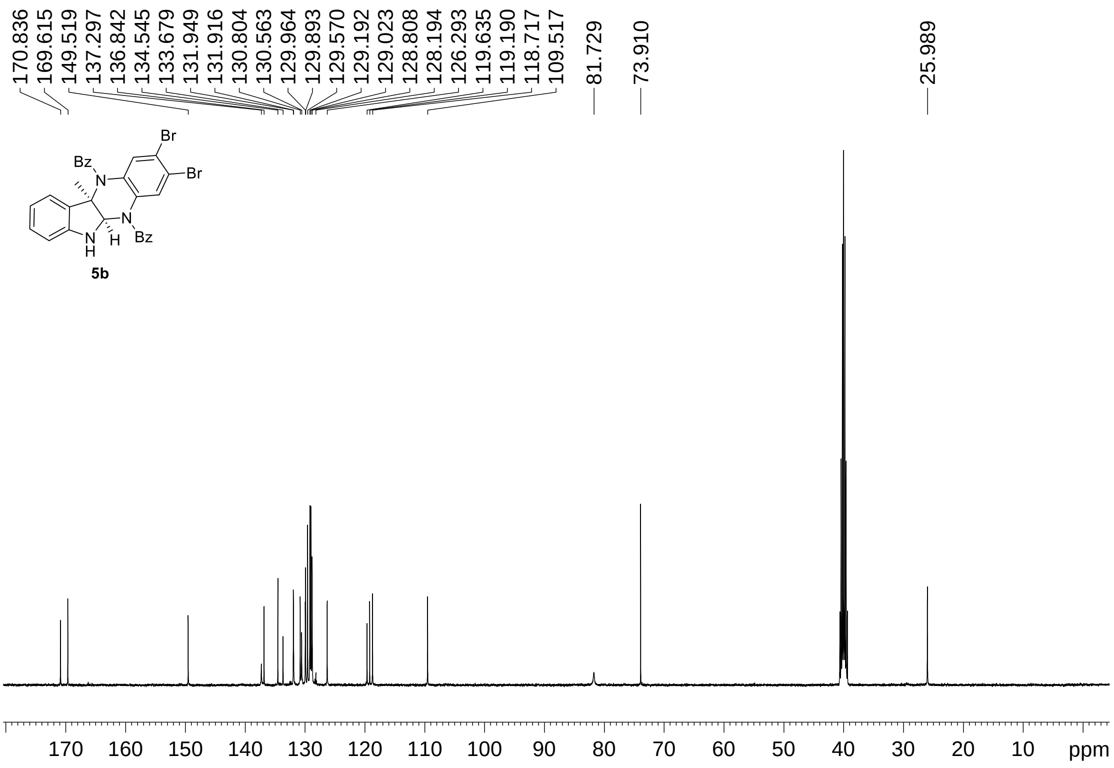

^{1}H NMR spectrum of compound **5c** (DMSO, 400 MHz)

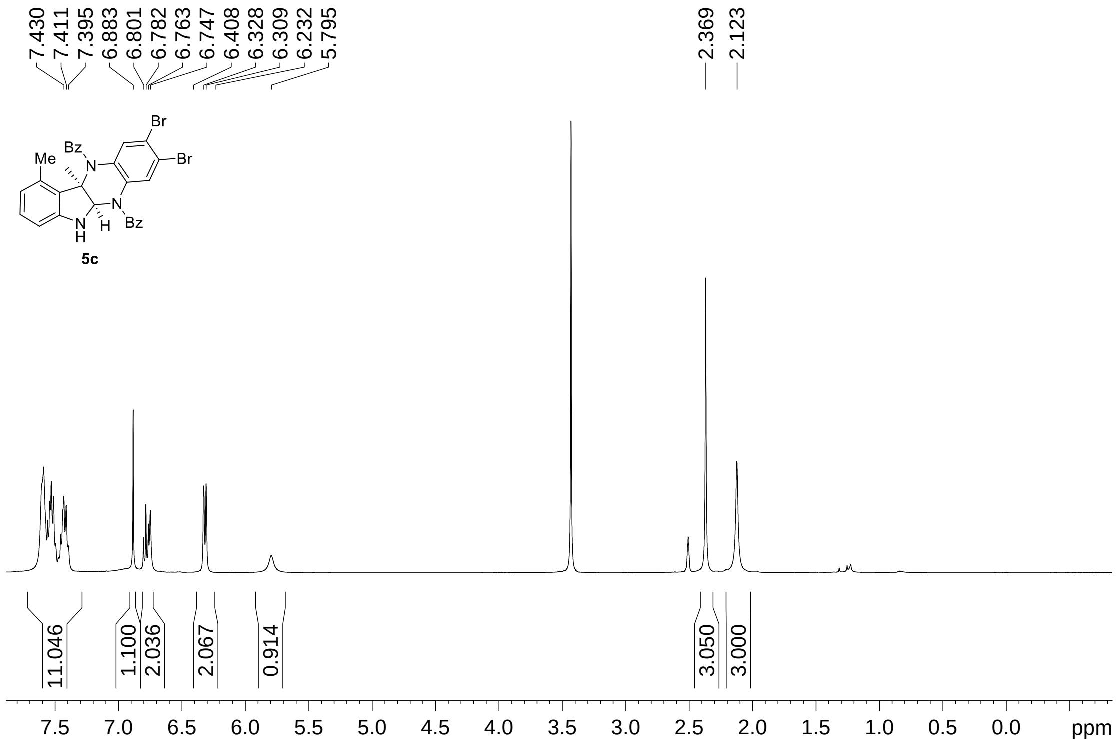

^{13}C NMR spectrum of compound **5c** (DMSO, 100 MHz)

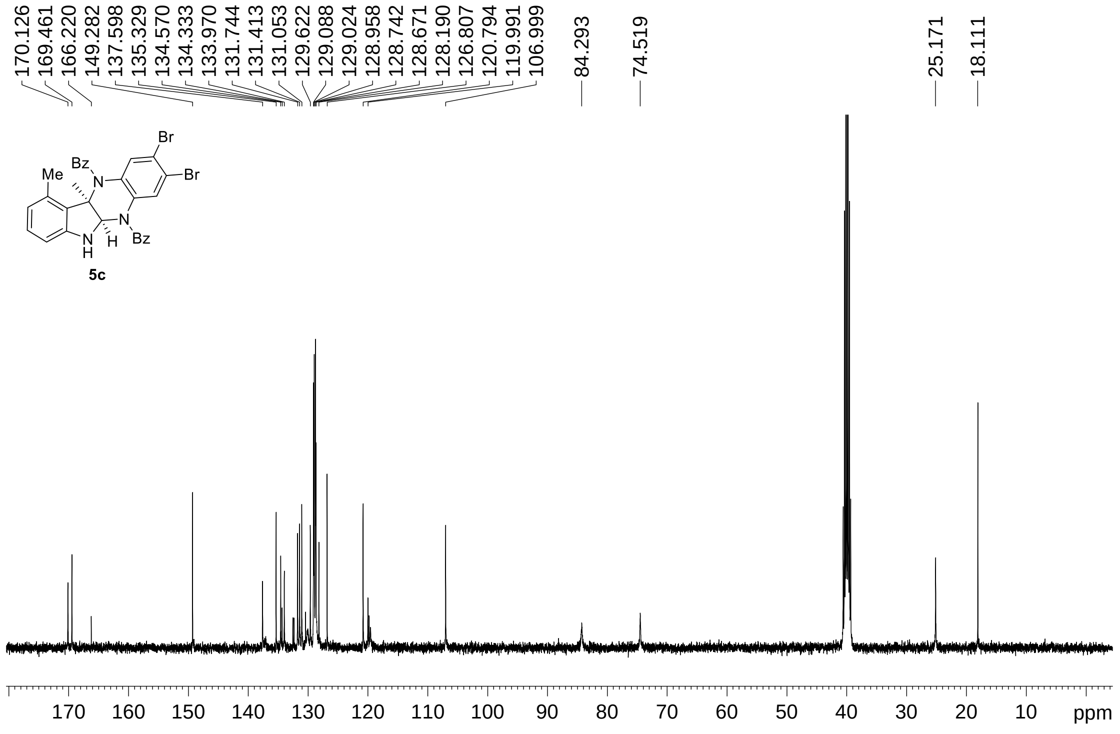

^{1}H NMR spectrum of compound **5d** (DMSO, 400 MHz)

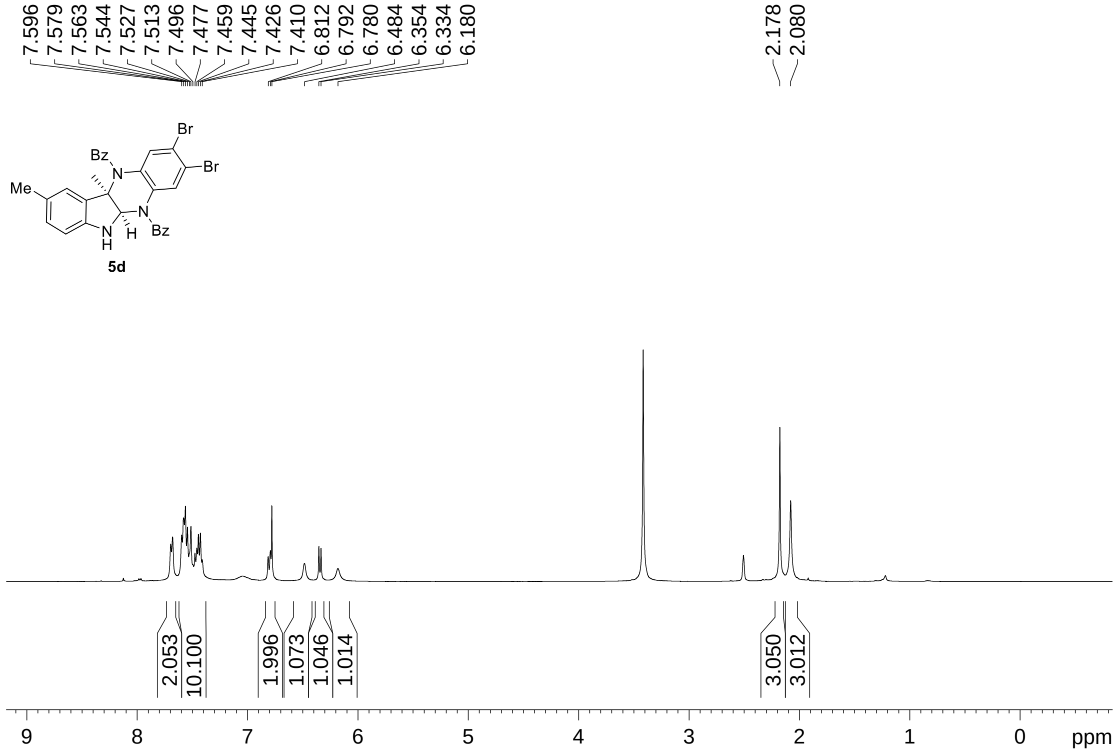

^{13}C NMR spectrum of compound **5d** (DMSO, 100 MHz)

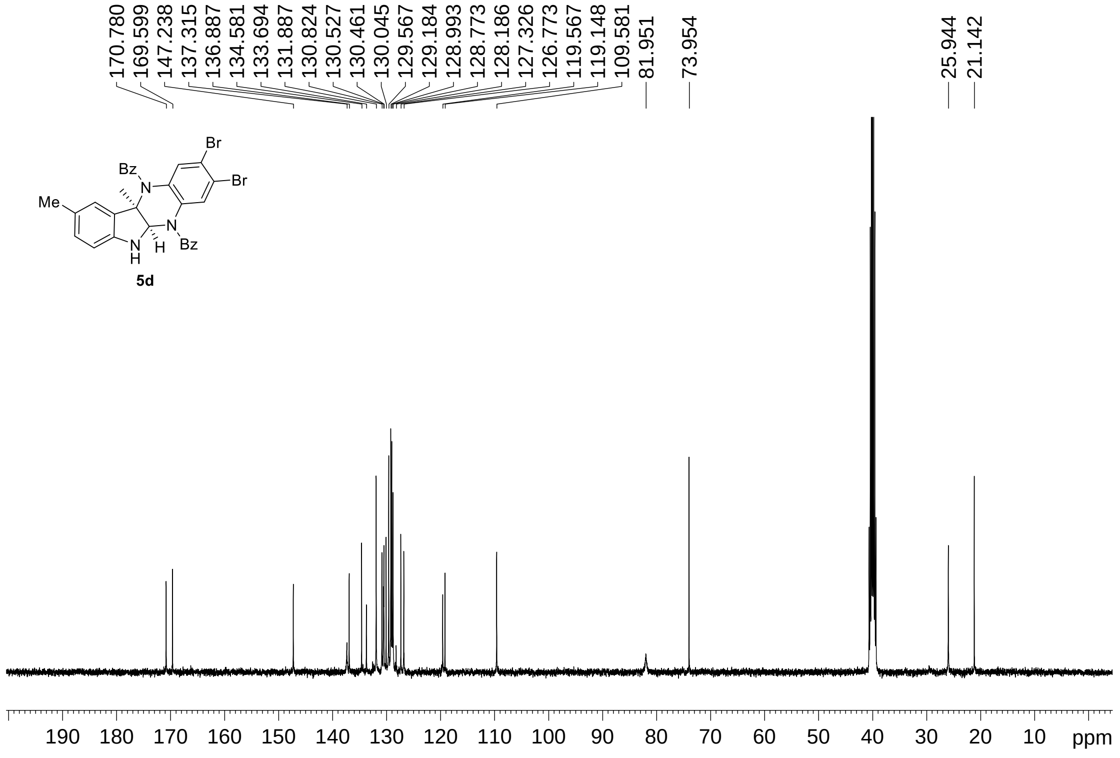

^{1}H NMR spectrum of compound **5e** (DMSO, 400 MHz)

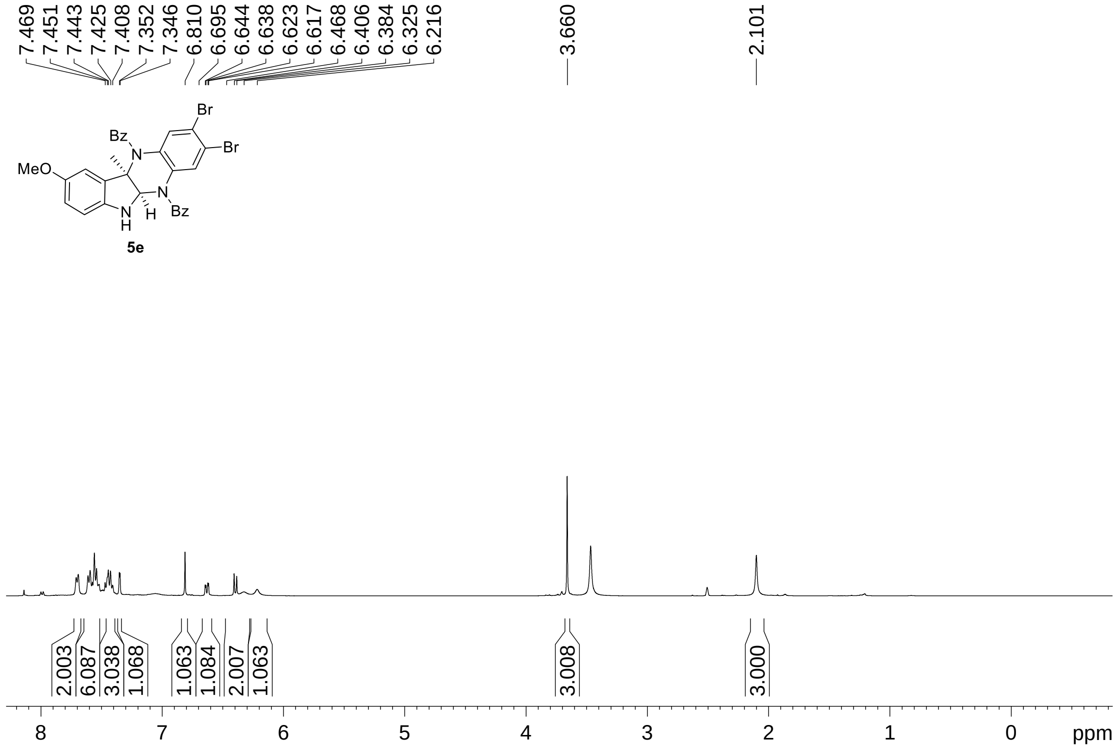

^{13}C NMR spectrum of compound **5e** (DMSO, 100 MHz)

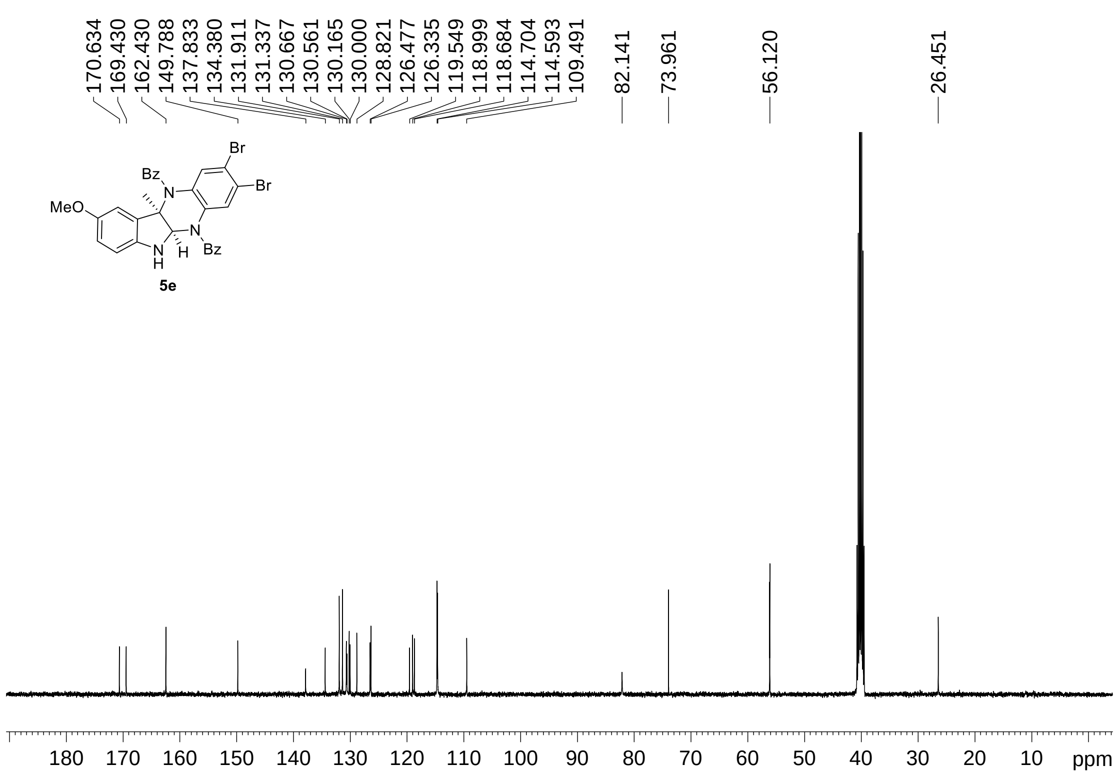

^{1}H NMR spectrum of compound **5f** (DMSO, 400 MHz)

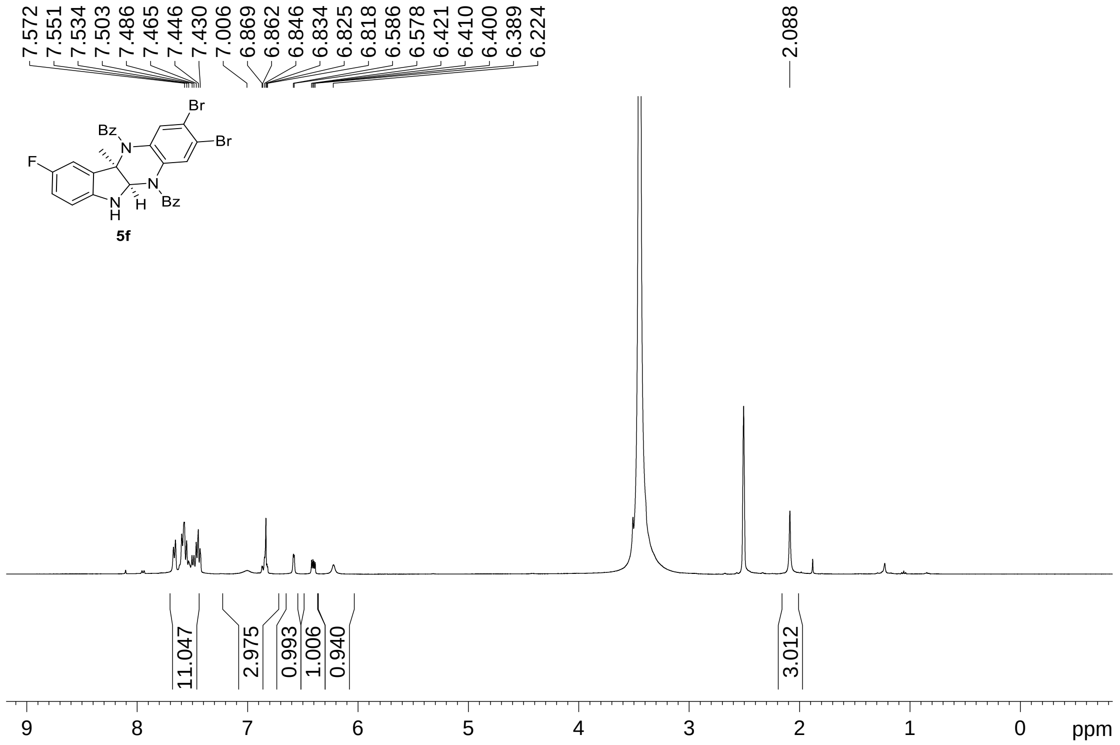

^{13}C NMR spectrum of compound **5f** (DMSO, 100 MHz)

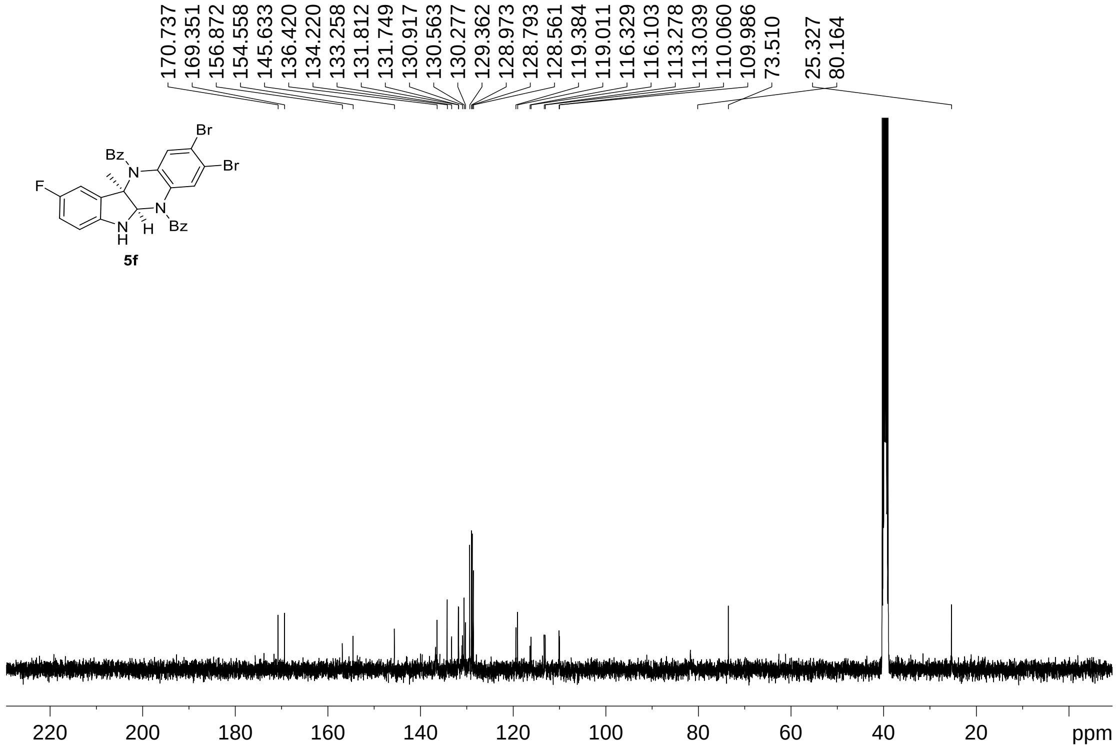

^{19}F NMR spectrum of compound **5f** (DMSO, 376 MHz)

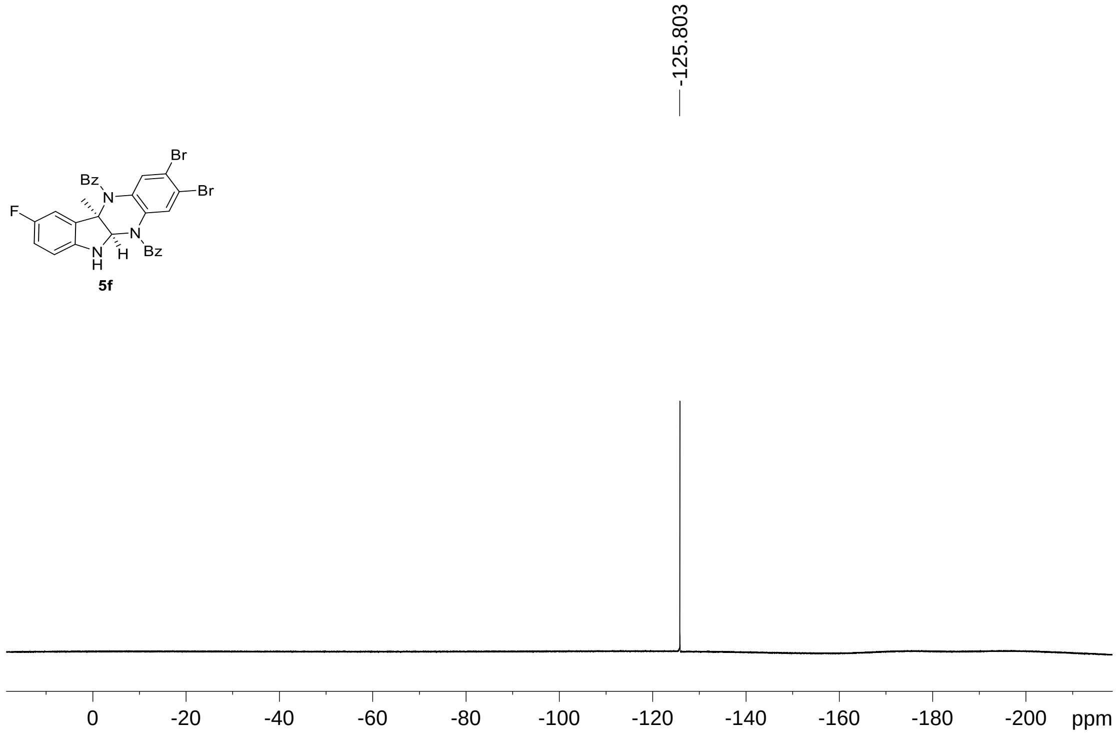

^{1}H NMR spectrum of compound **5g** (DMSO, 400 MHz)

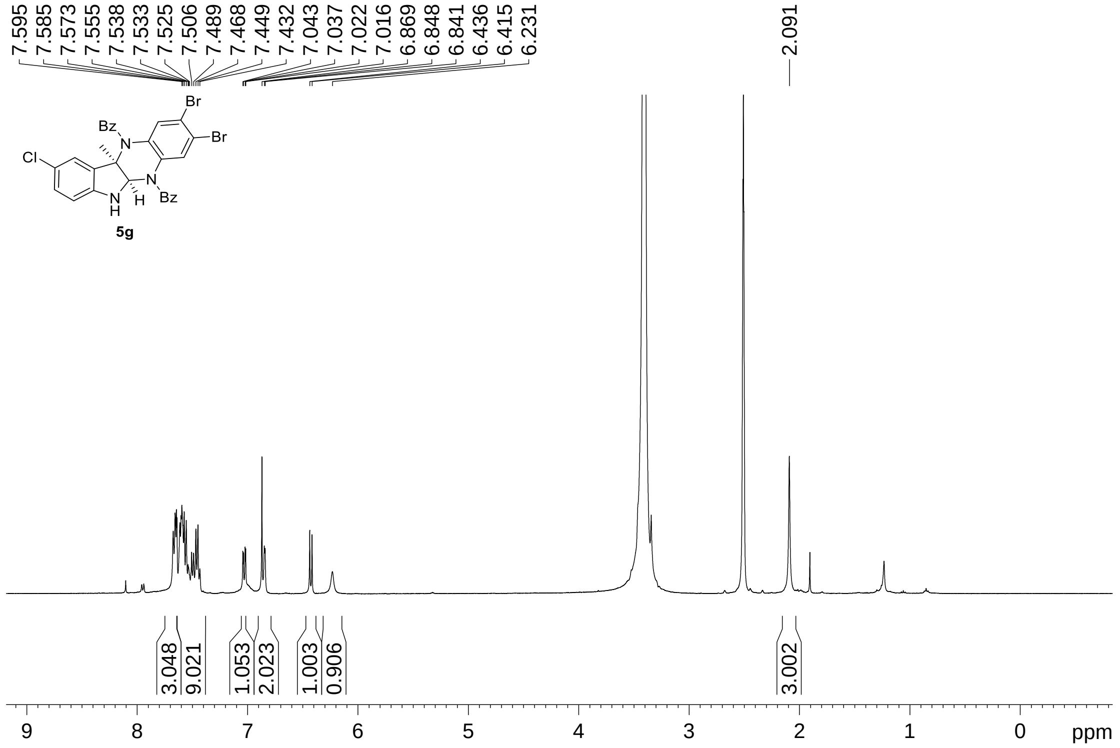

^{13}C NMR spectrum of compound **5g** (DMSO, 100 MHz)

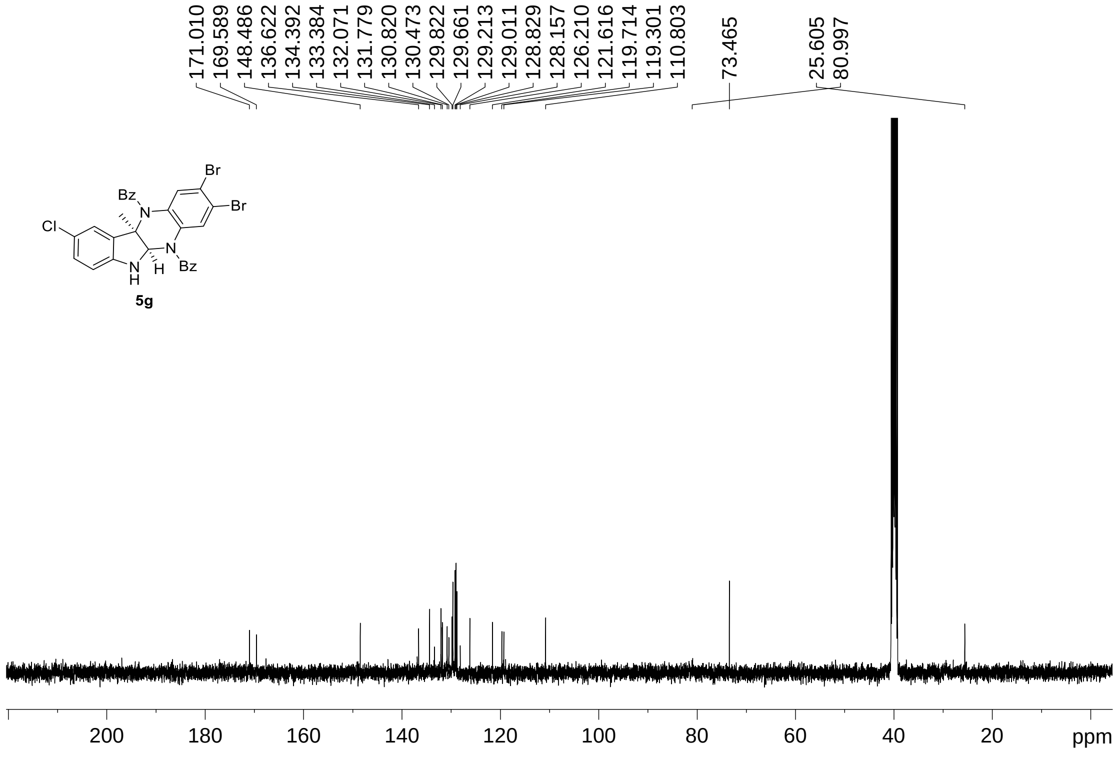

^{1}H NMR spectrum of compound **5h** (DMSO, 400 MHz)

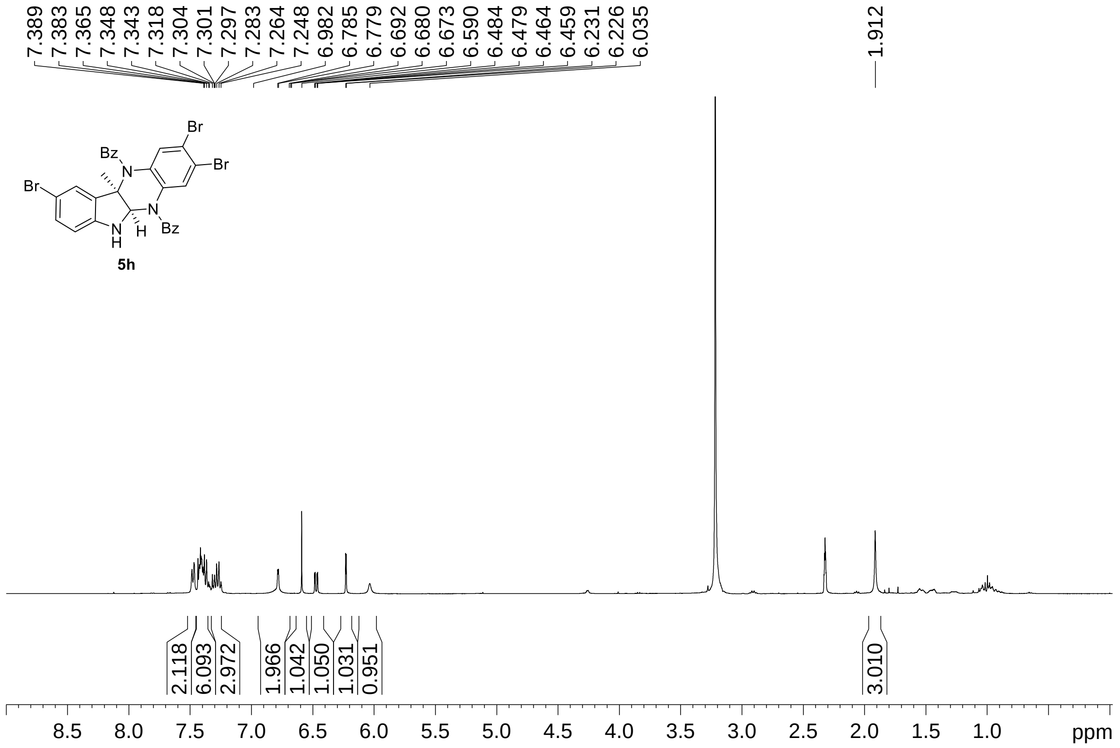

^{13}C NMR spectrum of compound **5h** (DMSO, 100 MHz)

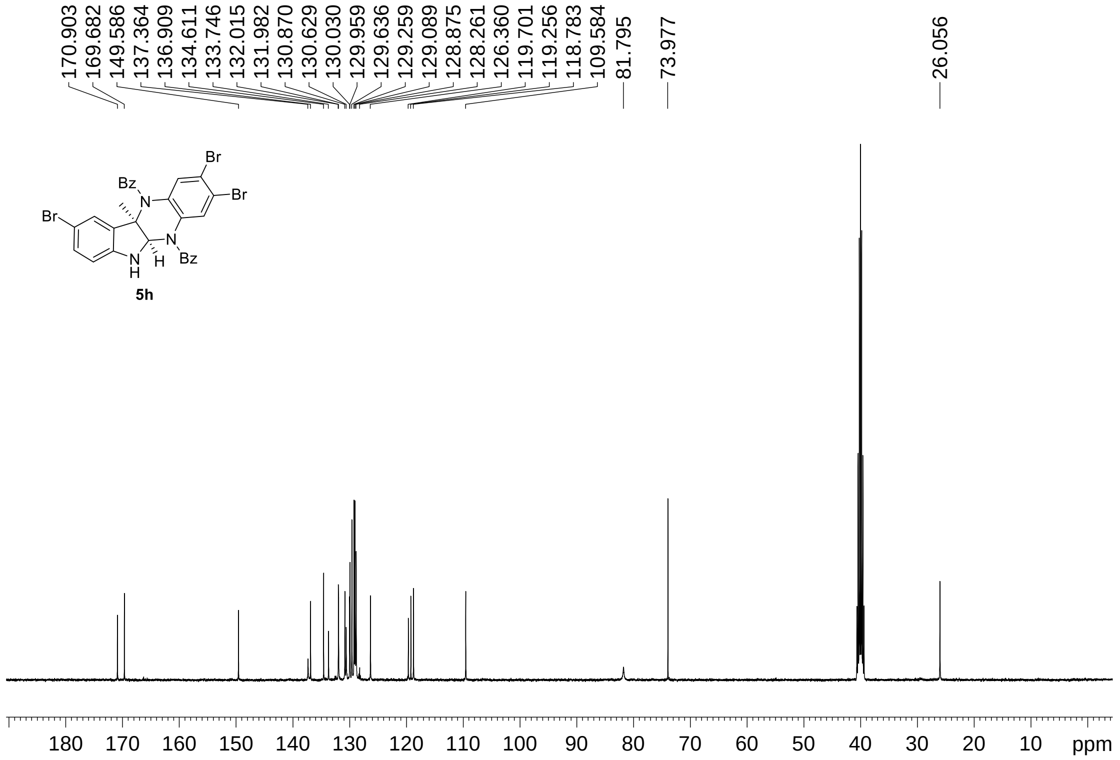

^{1}H NMR spectrum of compound **5i** (DMSO, 400 MHz)

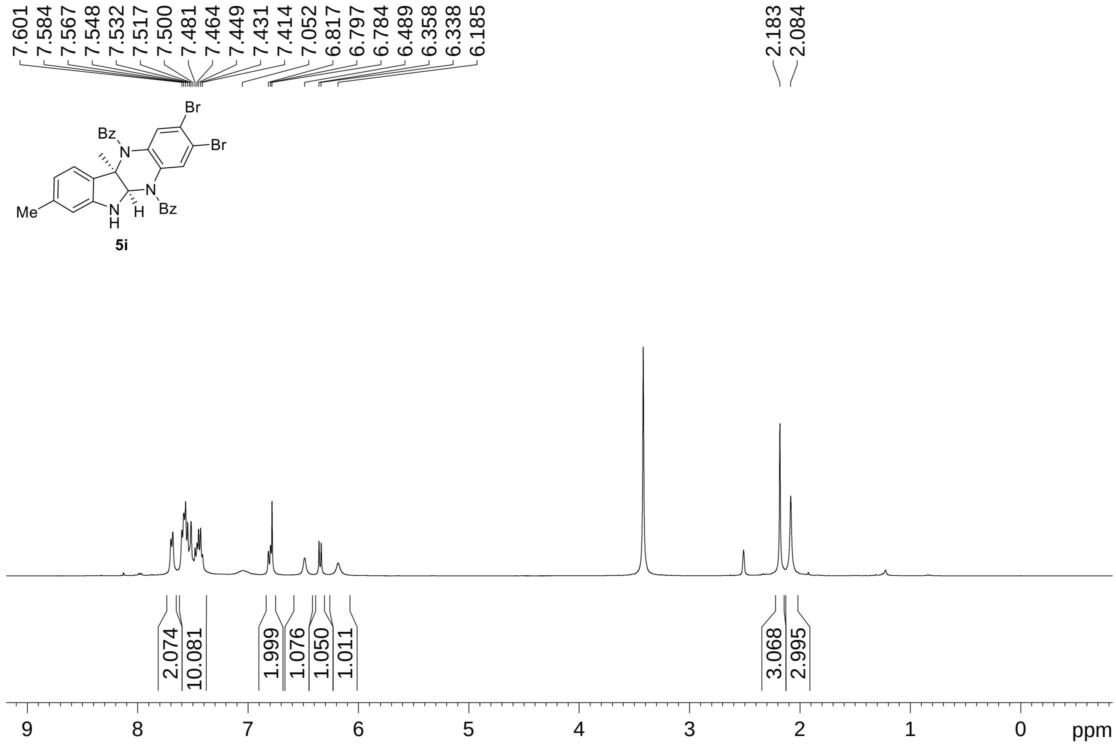

^{13}C NMR spectrum of compound **5i** (DMSO, 100 MHz)

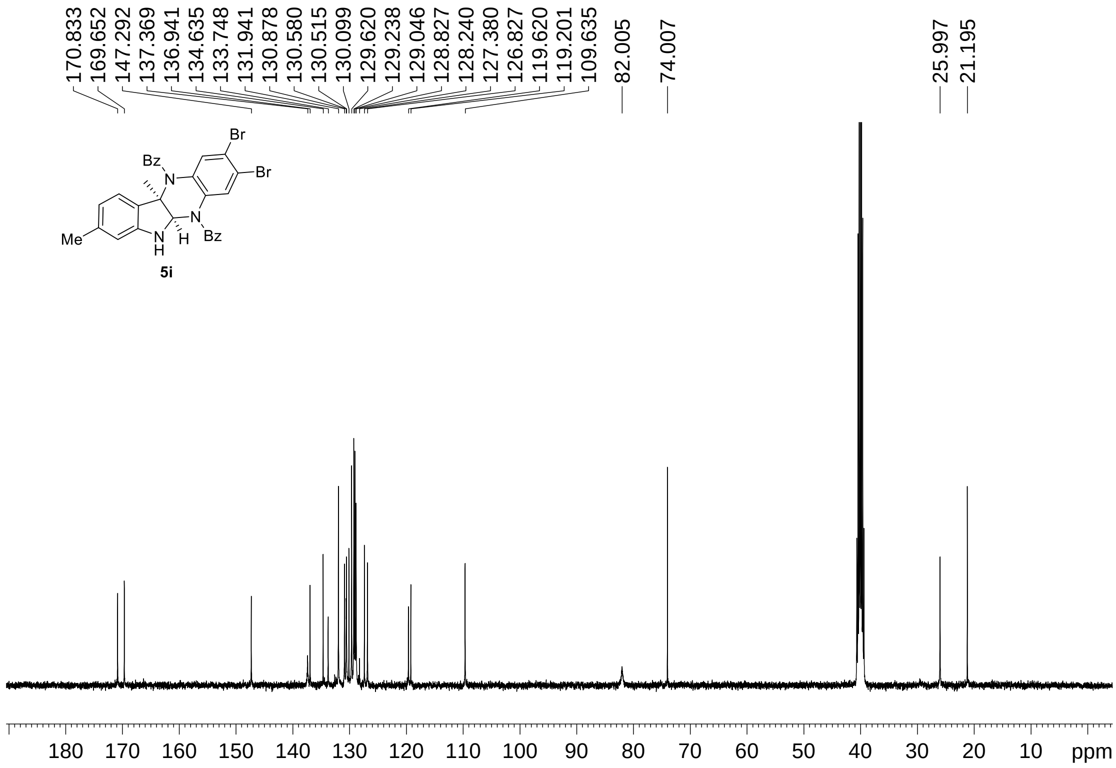

^{1}H NMR spectrum of compound **5j** (DMSO, 400 MHz)

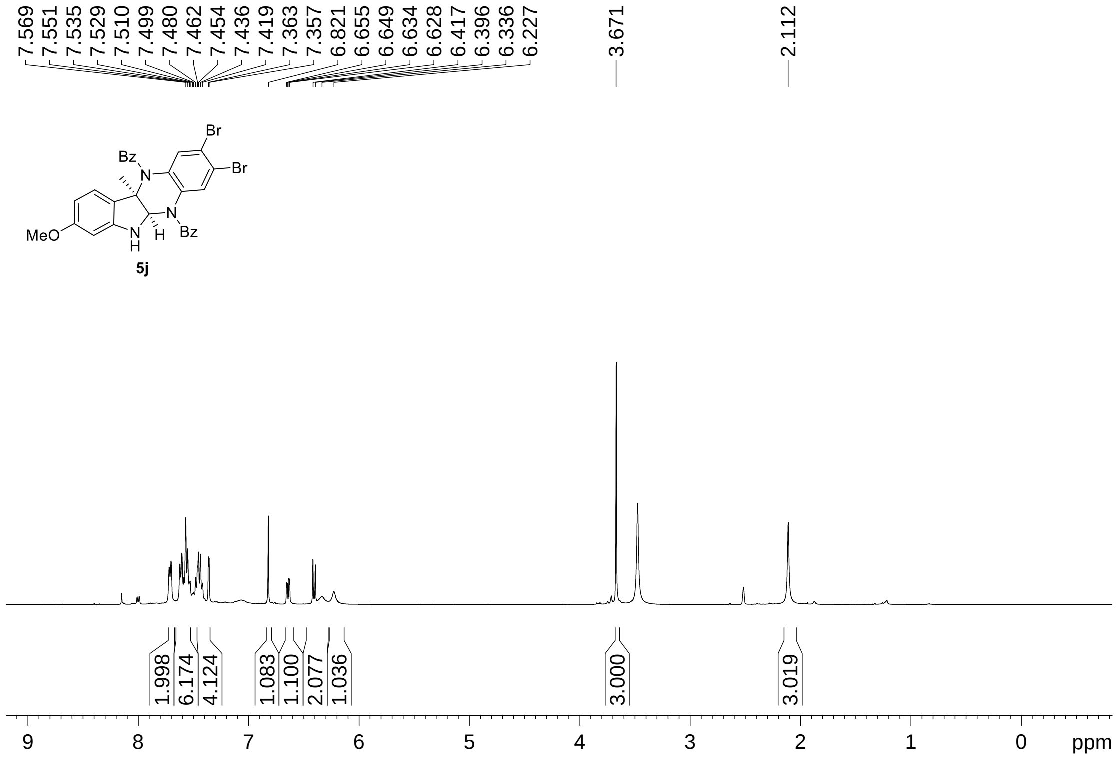

^{13}C NMR spectrum of compound **5j** (DMSO, 100 MHz)

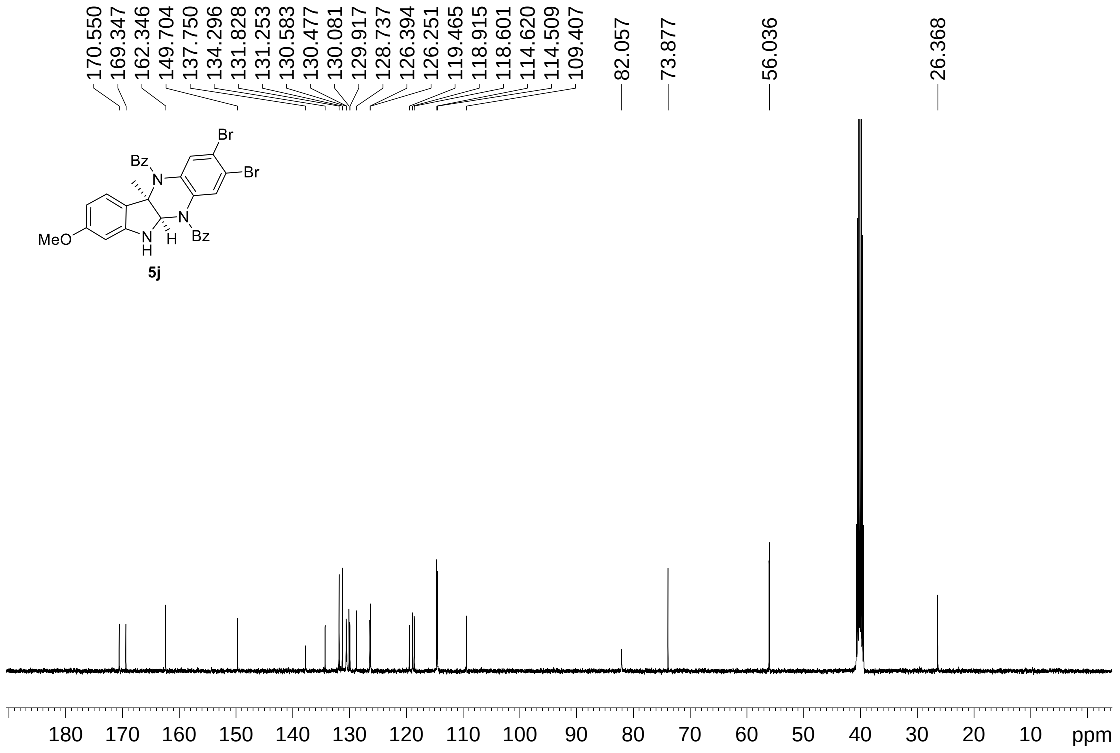

^{1}H NMR spectrum of compound **5k** (DMSO, 400 MHz)

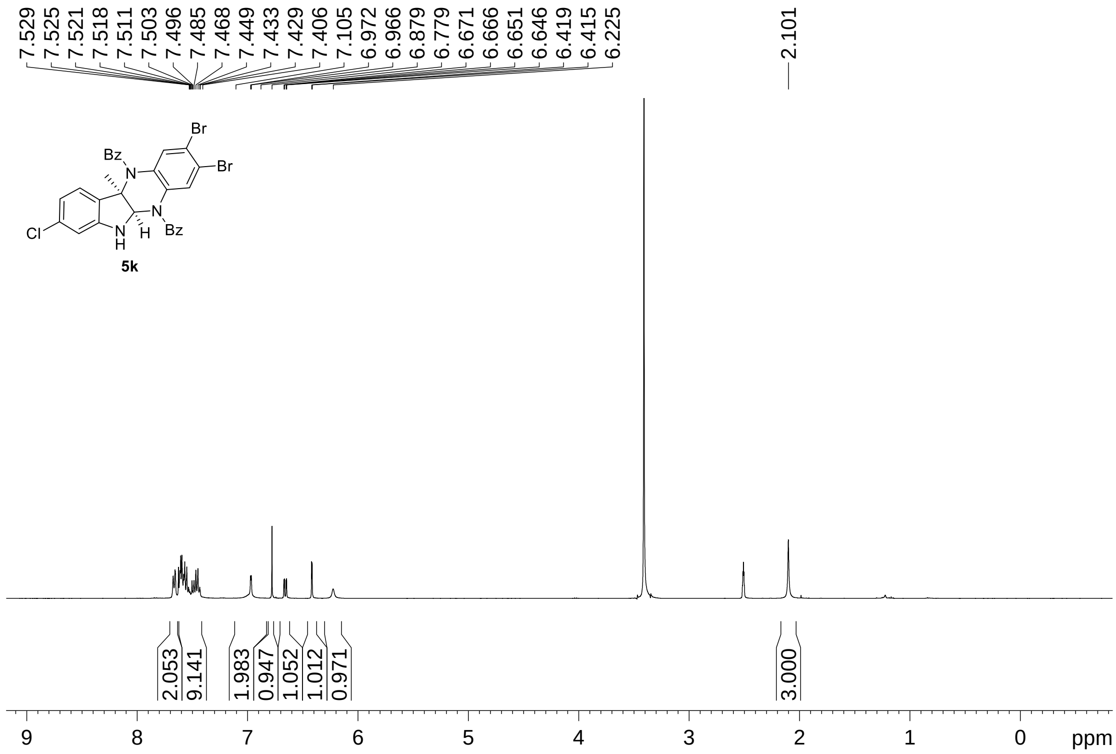

^{13}C NMR spectrum of compound **5k** (DMSO, 100 MHz)

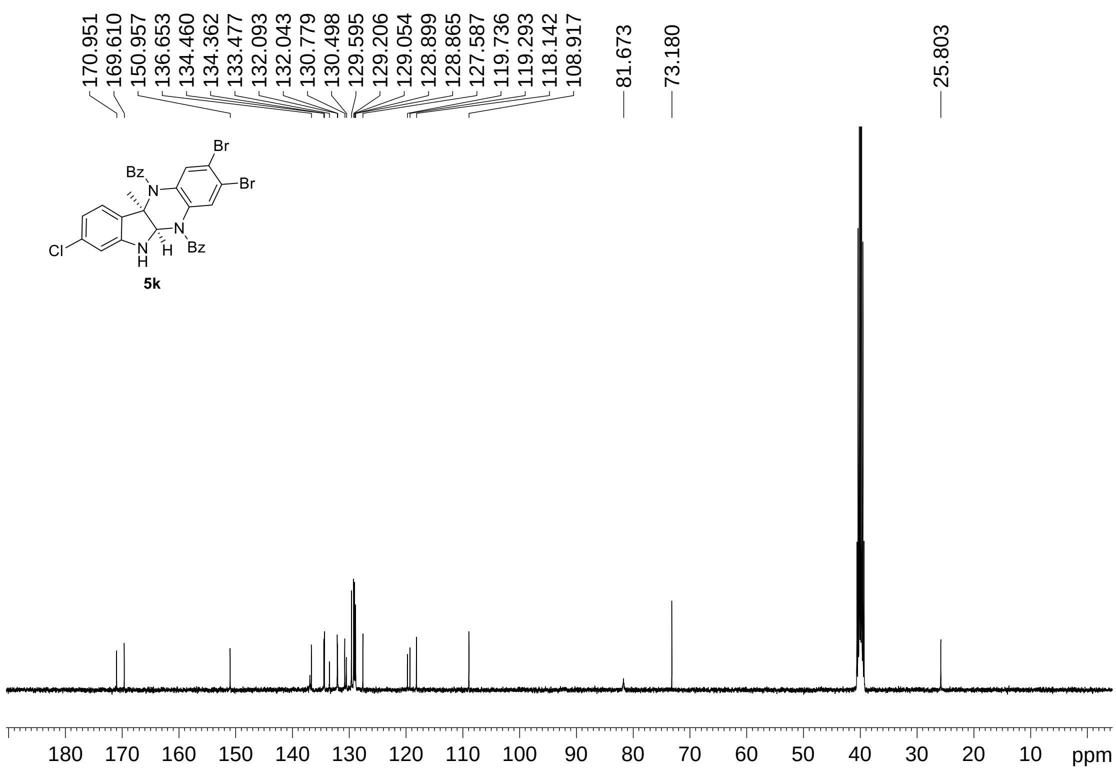

^{1}H NMR spectrum of compound **5l** (DMSO, 400 MHz)

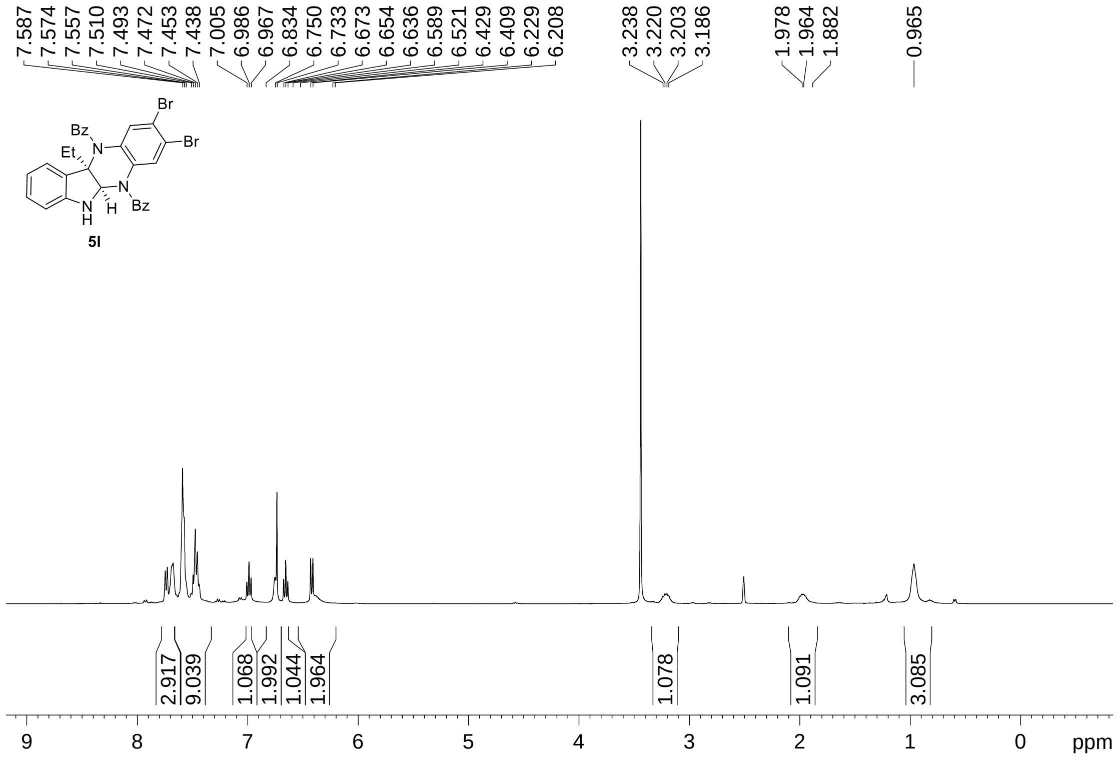

^{13}C NMR spectrum of compound **5l** (DMSO, 100 MHz)

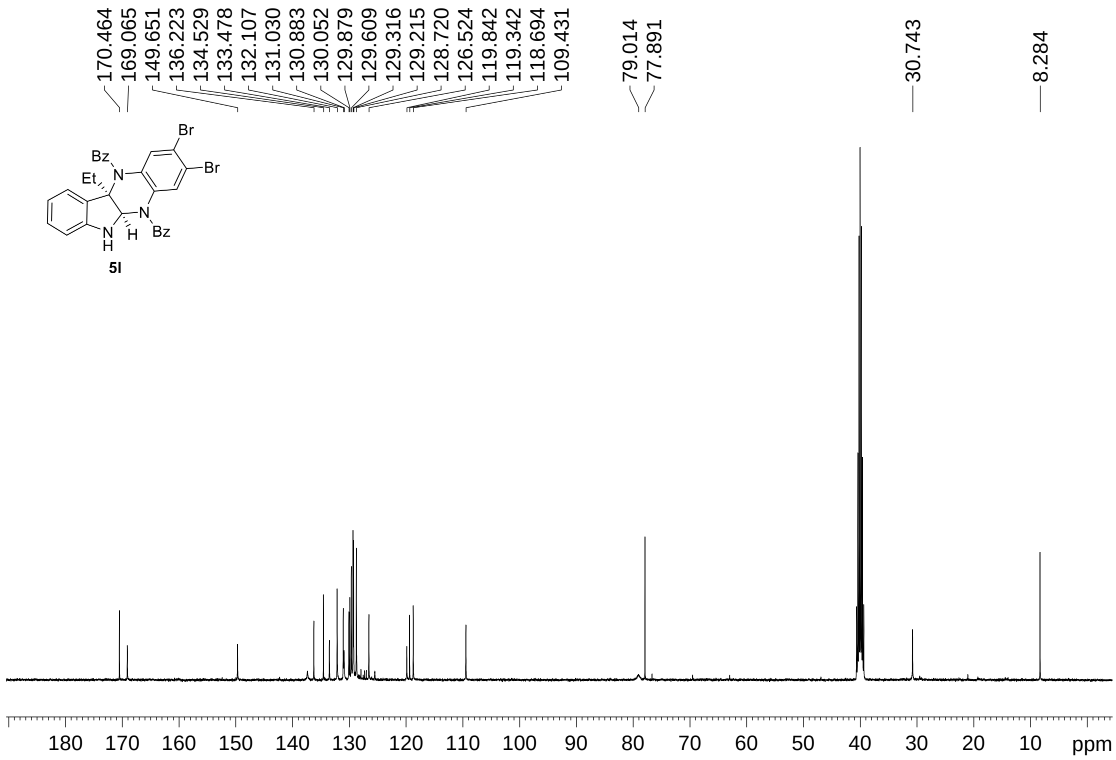

^{1}H NMR spectrum of compound **5m** (DMSO, 400 MHz)

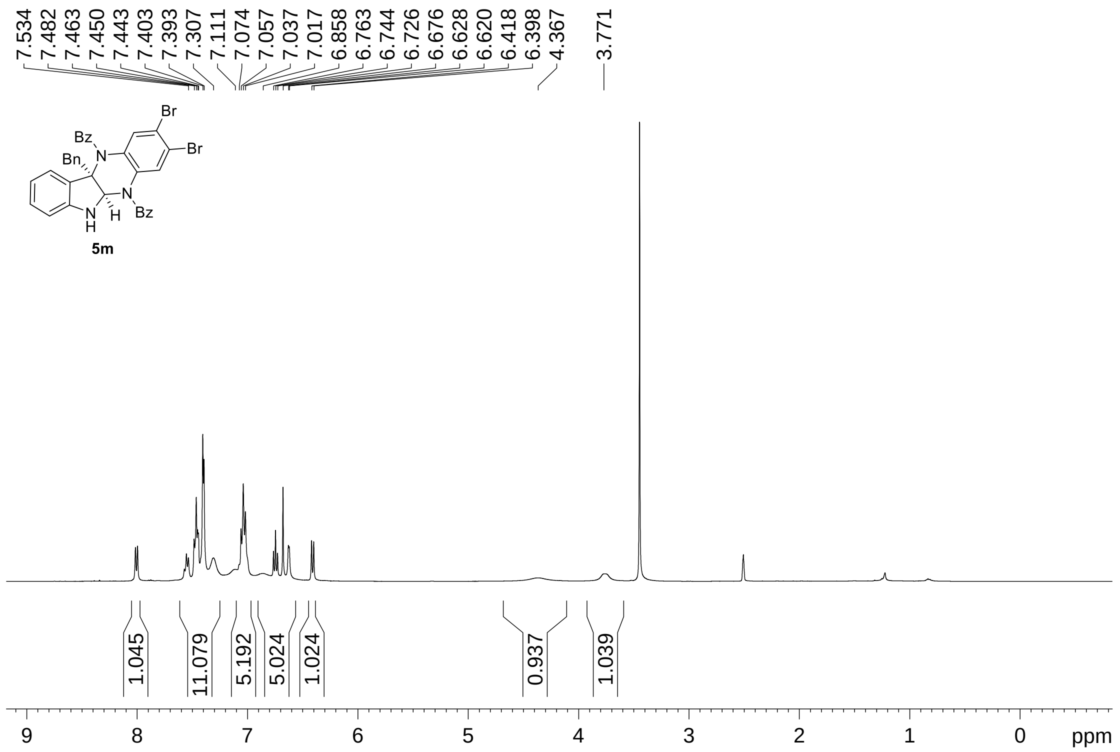

^{13}C NMR spectrum of compound **5m** (DMSO, 100 MHz)

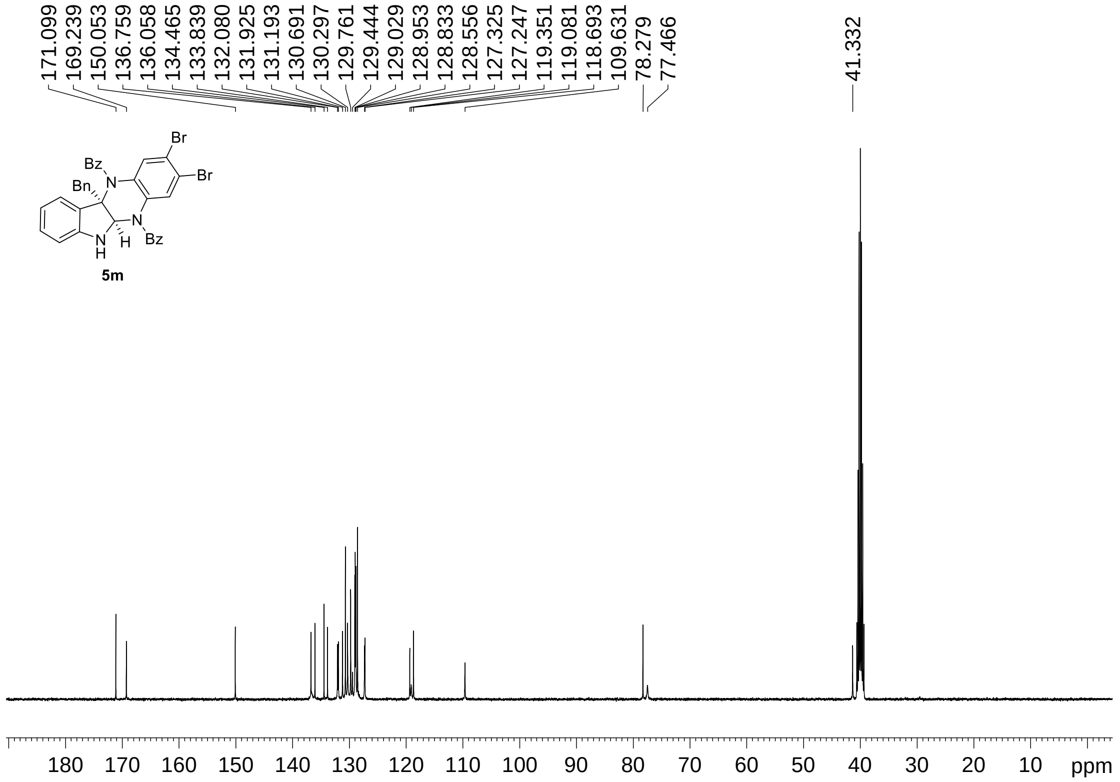

^{1}H NMR spectrum of compound **5n** ($CDCl_3$, 400 MHz)

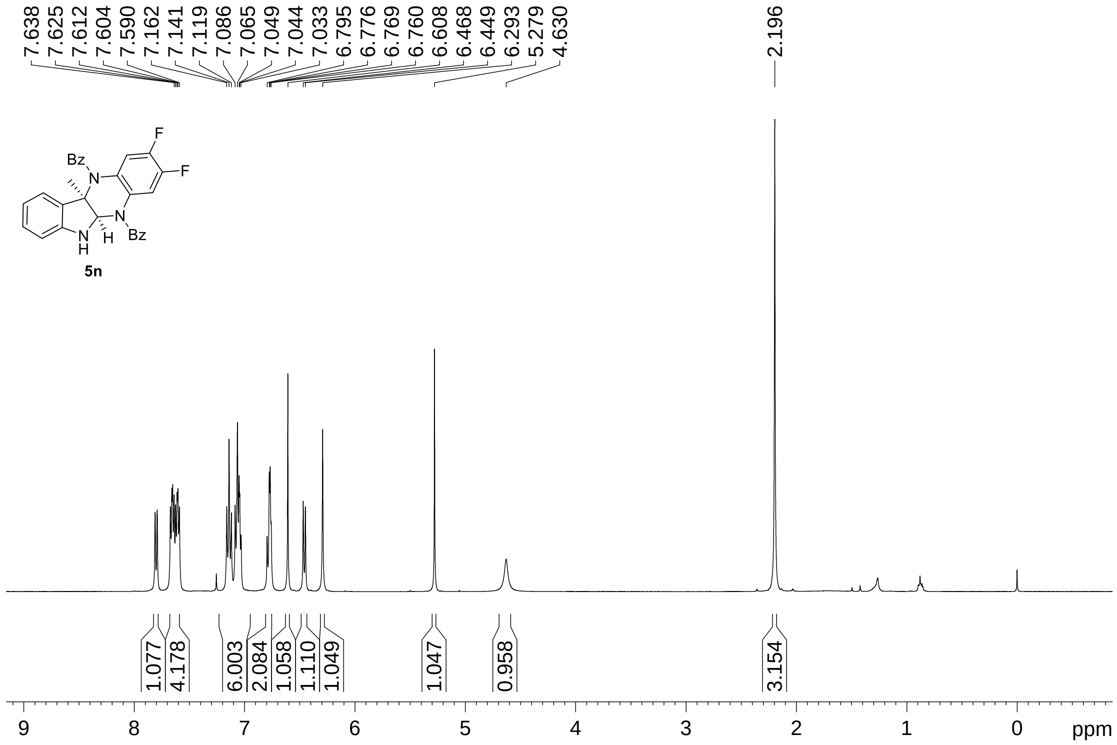

^{13}C NMR spectrum of compound **5n** ($CDCl_3$, 100 MHz)

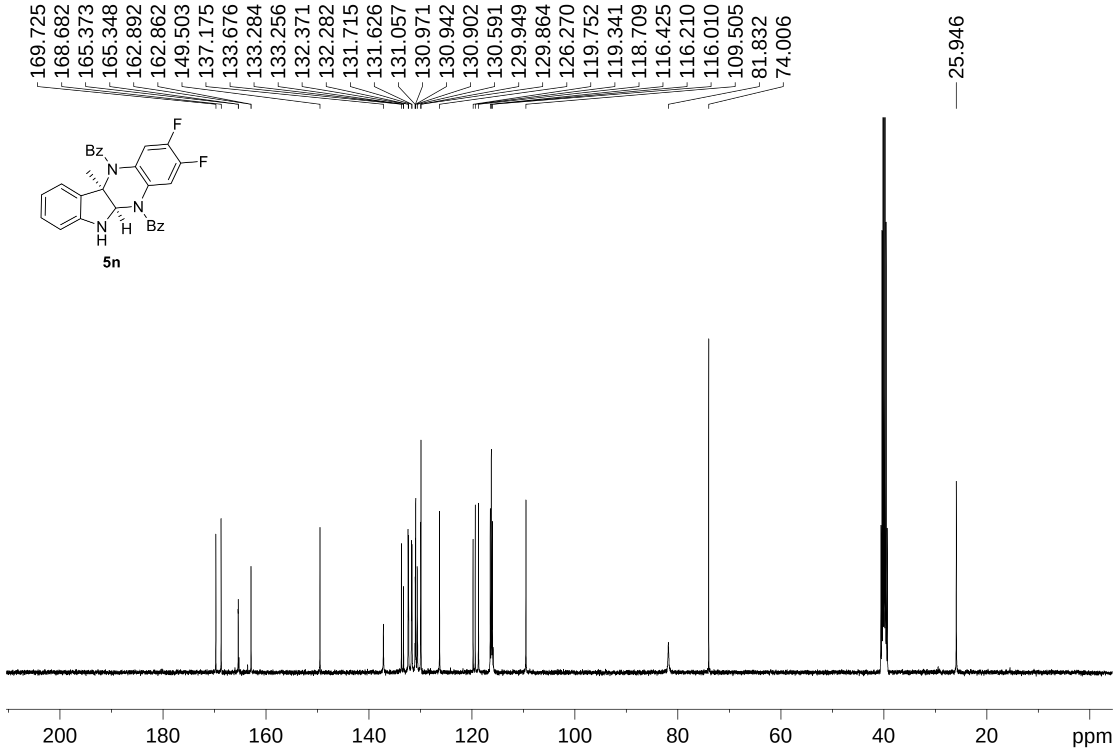

^{19}F NMR spectrum of compound **5n** ($CDCl_3$, 376 MHz)

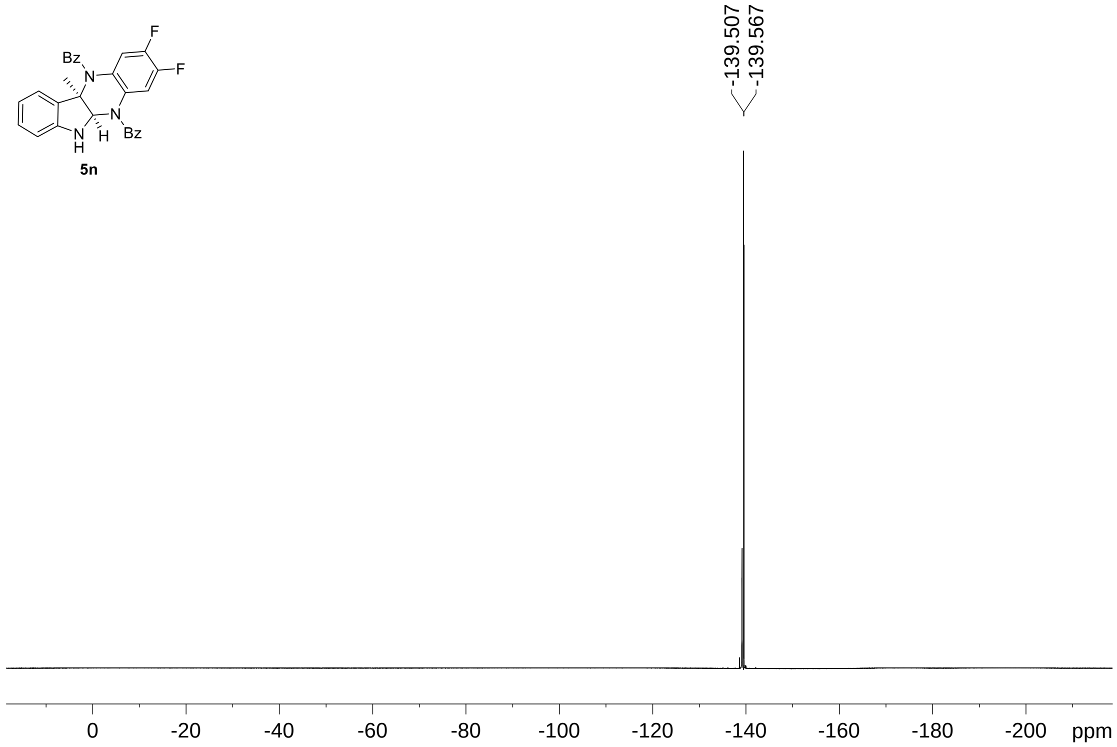

^{1}H NMR spectrum of compound **5o** (DMSO, 400 MHz)

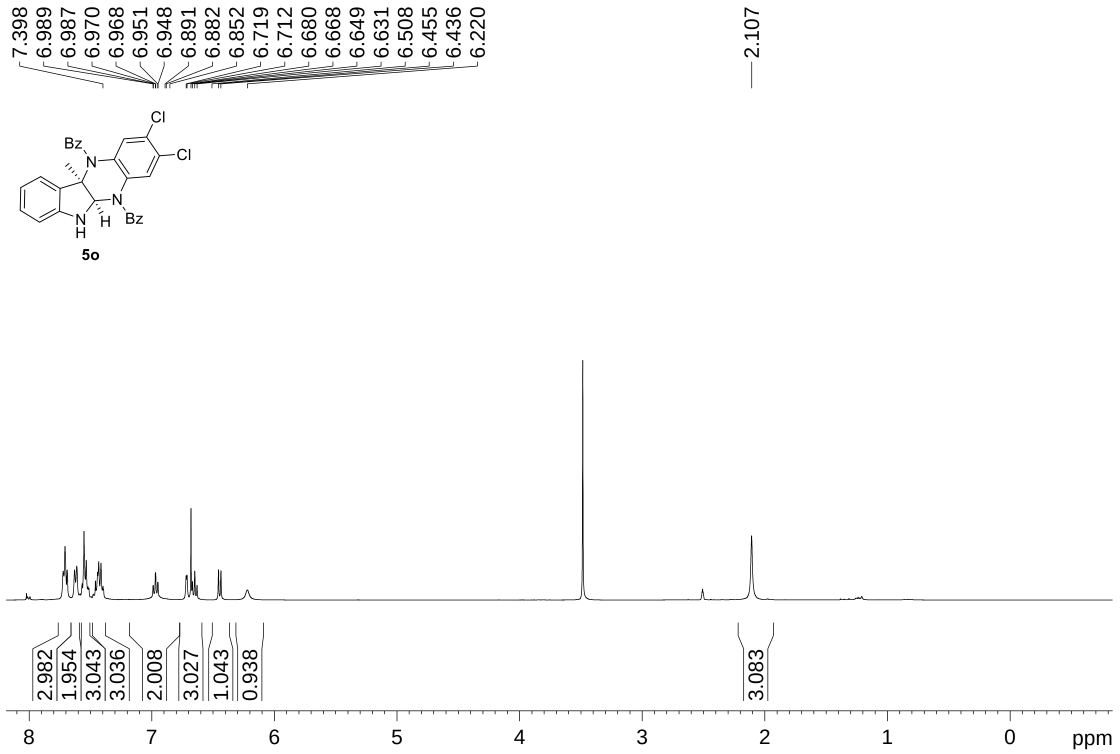

^{13}C NMR spectrum of compound **5o** (DMSO, 100 MHz)

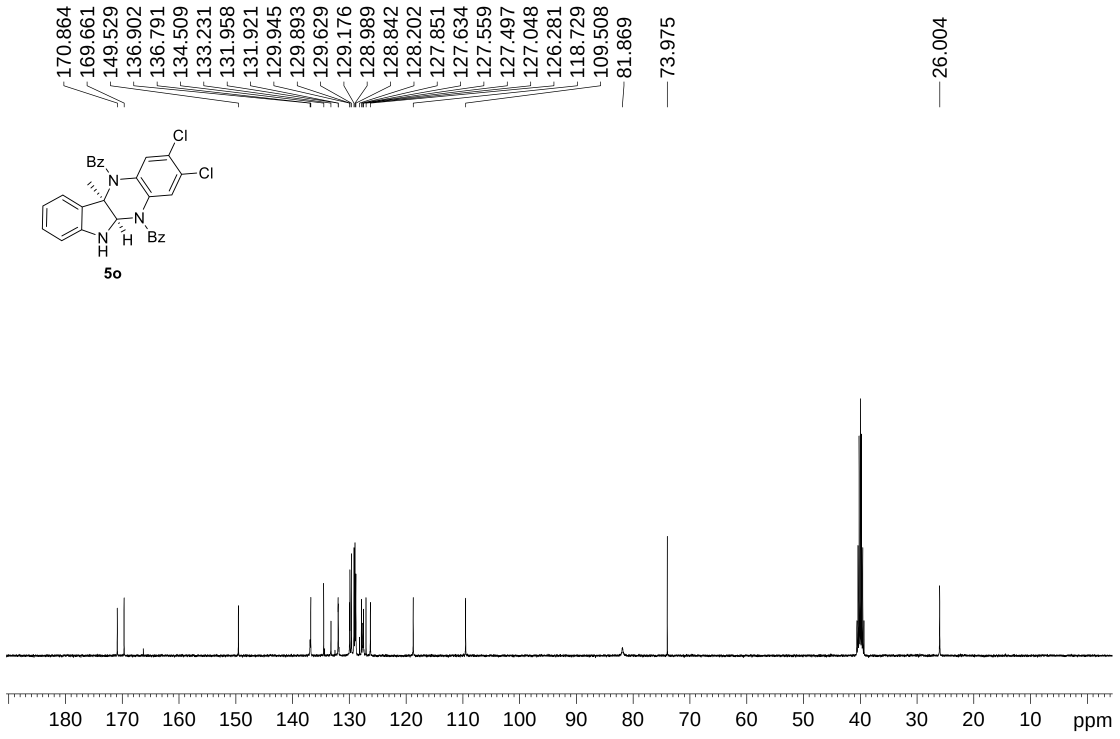

^{1}H NMR spectrum of compound **5p** (DMSO, 400 MHz)

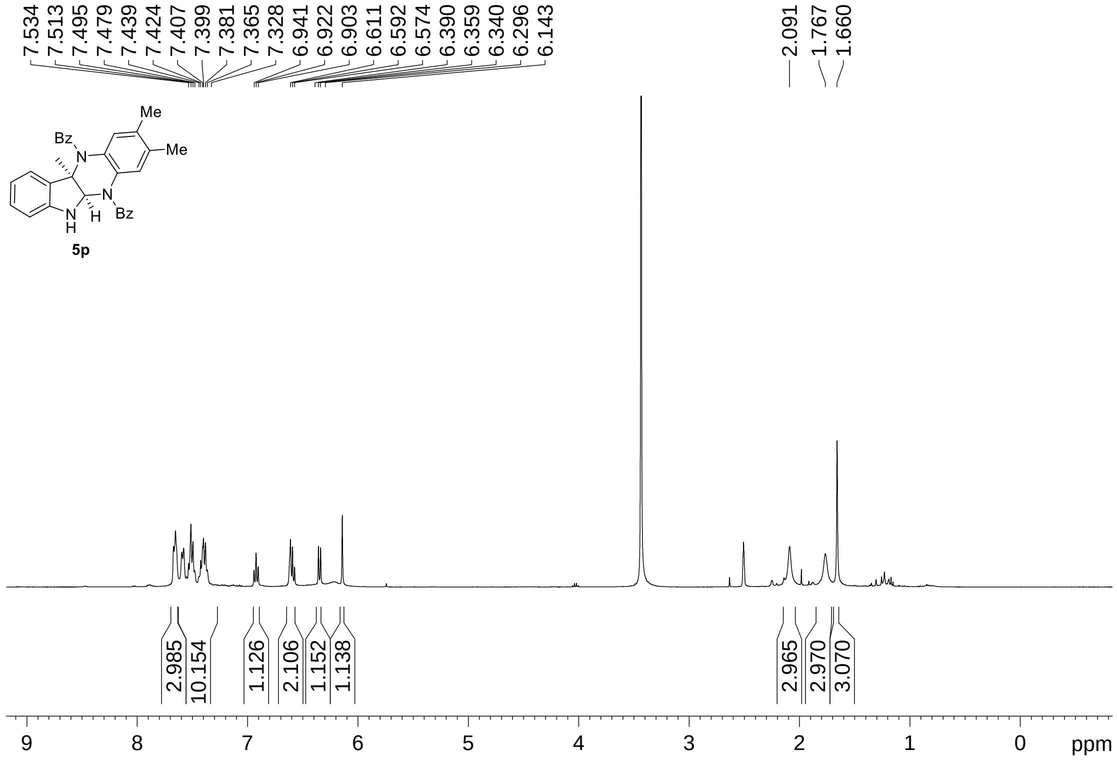

^{13}C NMR spectrum of compound **5p** (DMSO, 100 MHz)

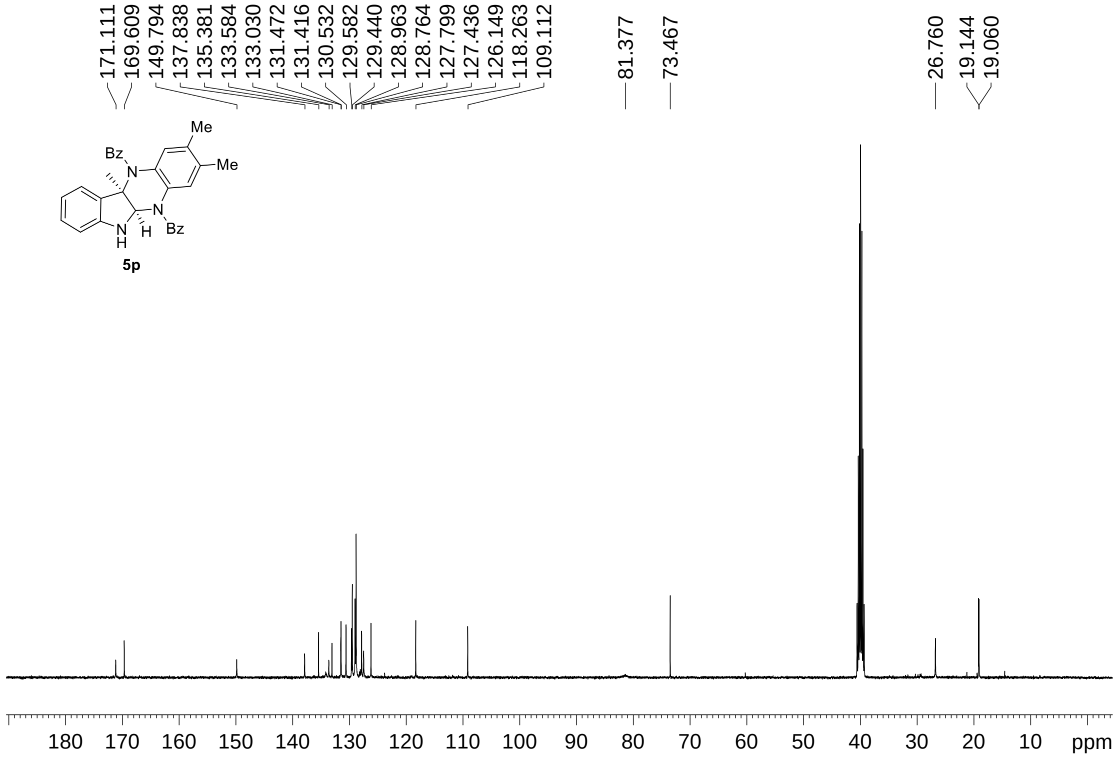

^{1}H NMR spectrum of compound **5q** (DMSO, 400 MHz)

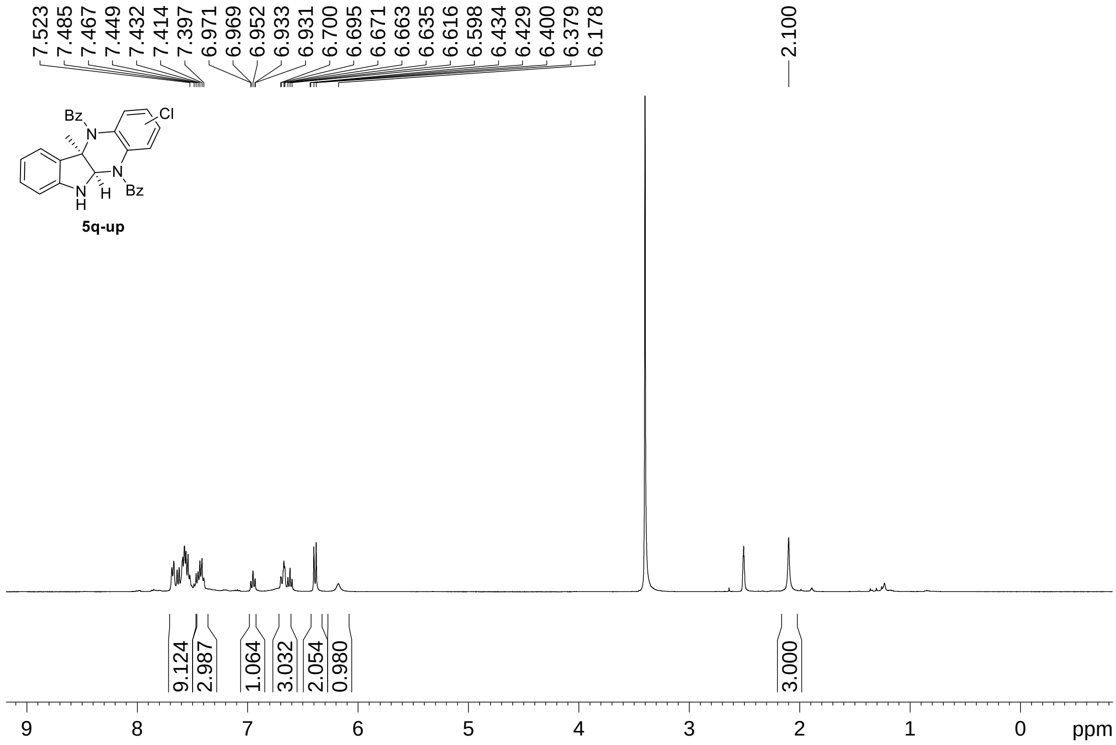

^{13}C NMR spectrum of compound **5q** (DMSO, 100 MHz)

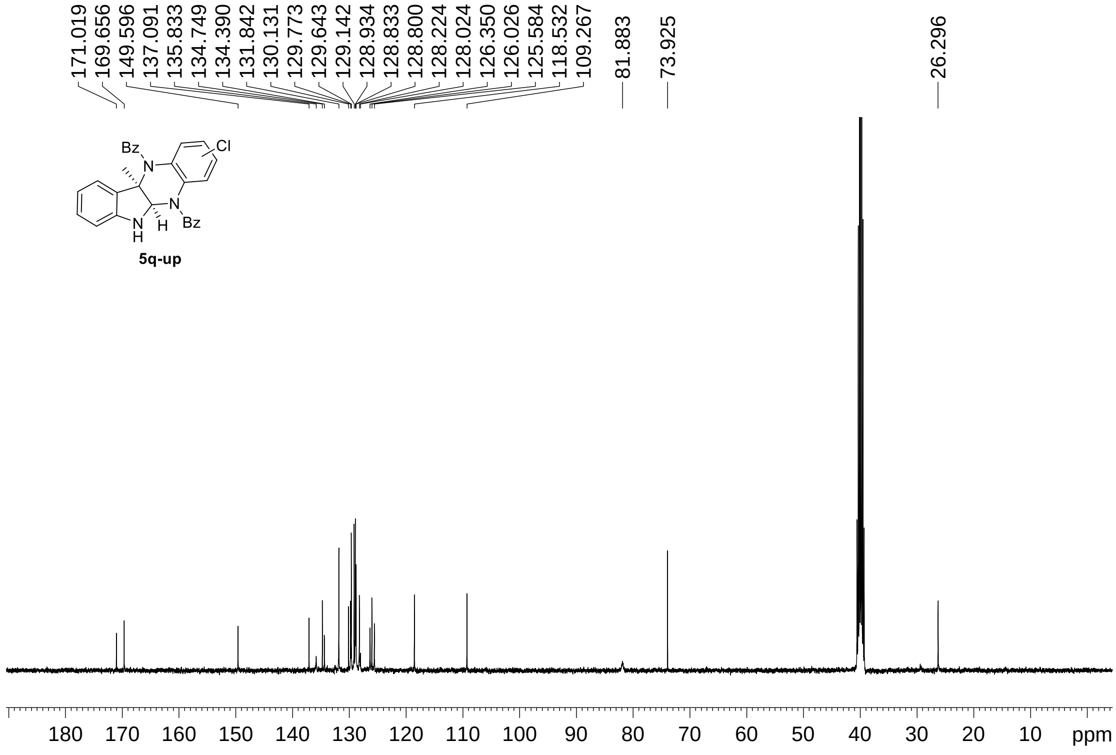

^{1}H NMR spectrum of compound **5q** (DMSO, 400 MHz)

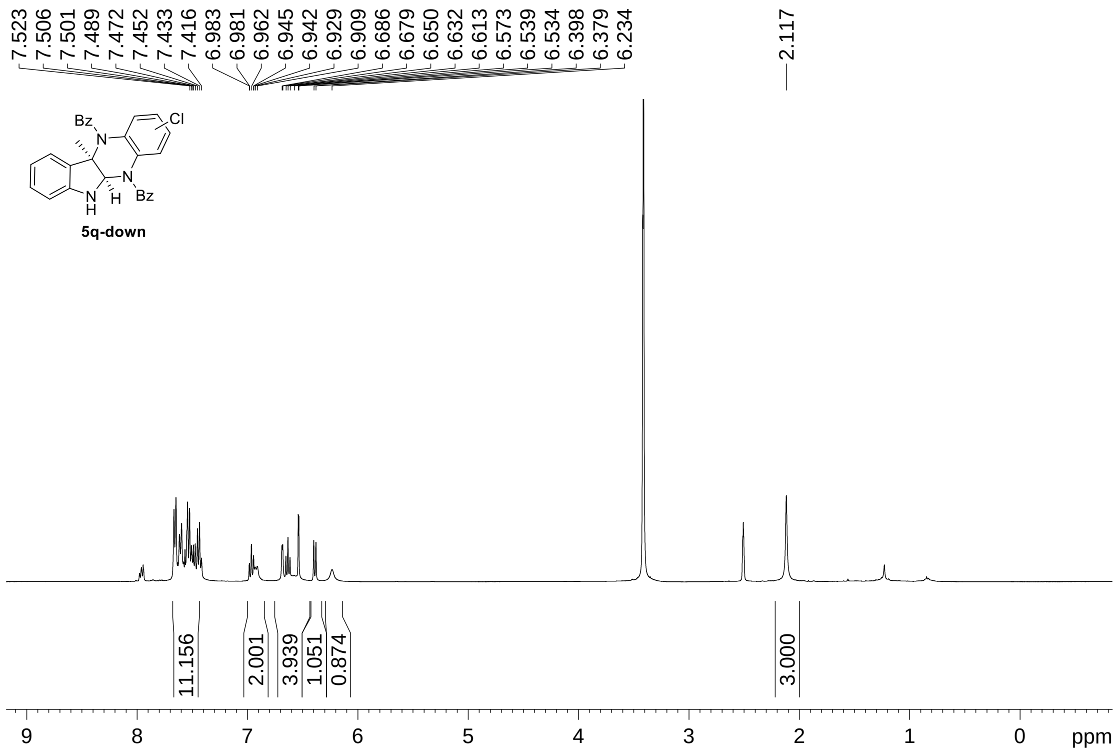

^{13}C NMR spectrum of compound **5q** (DMSO, 100 MHz)

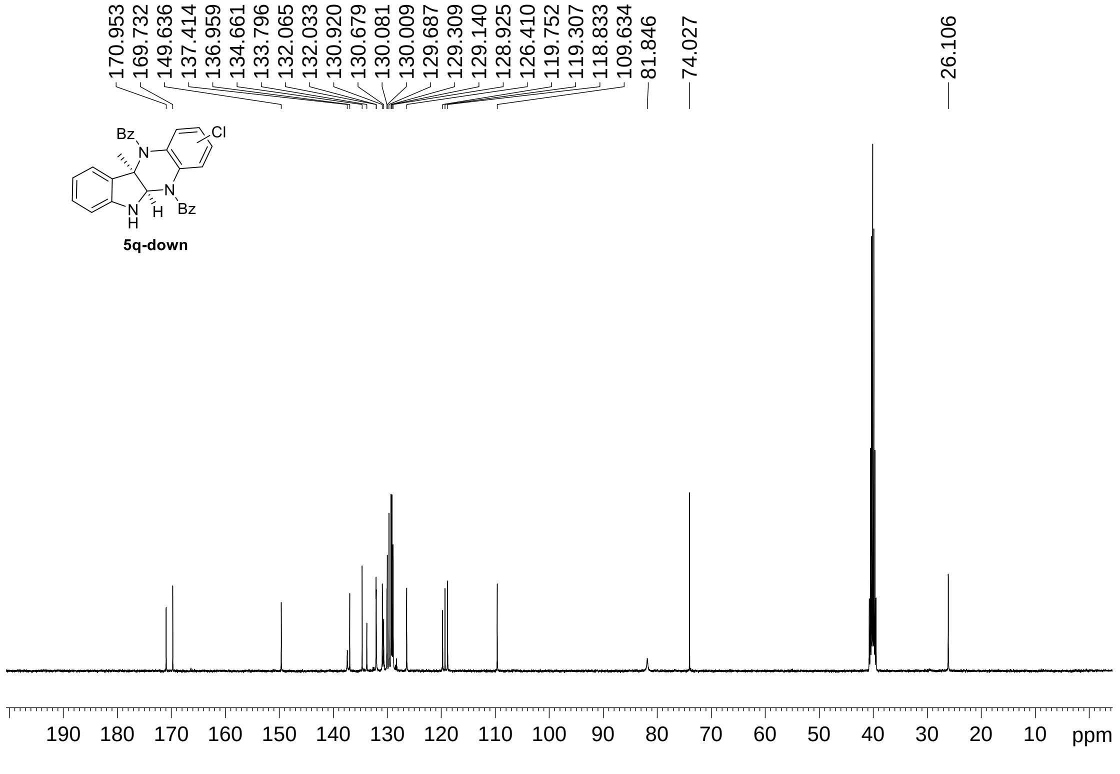

^{1}H NMR spectrum of compound **5r** (DMSO, 400 MHz)

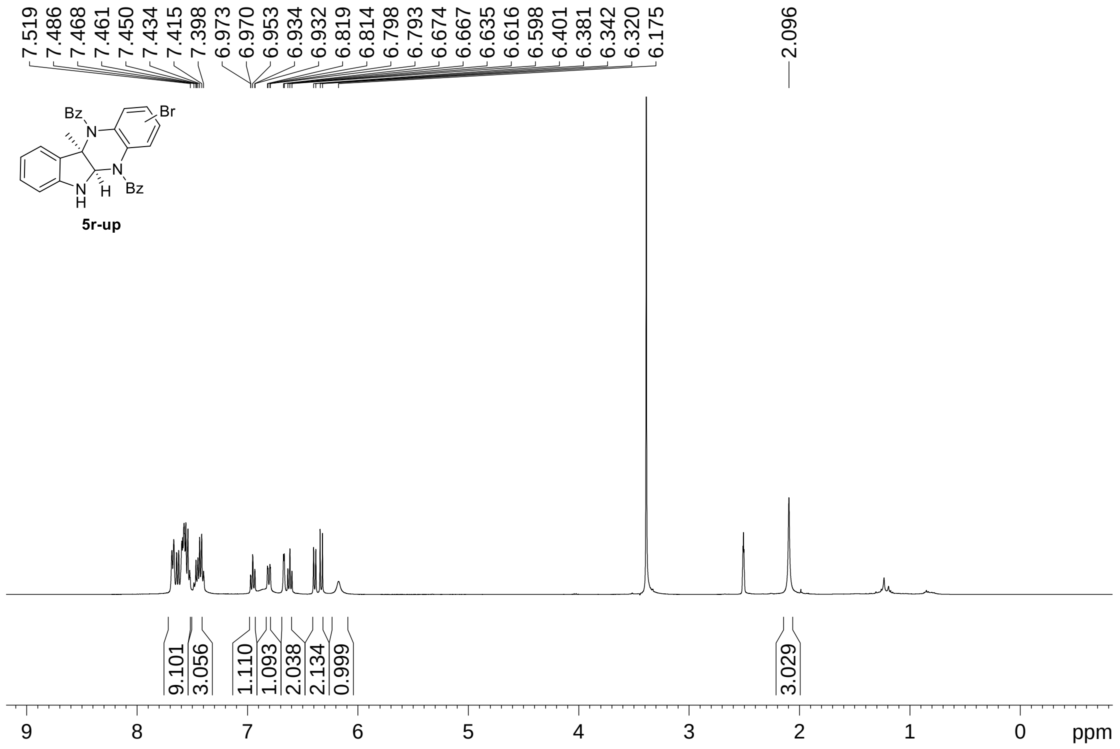

^{13}C NMR spectrum of compound **5r** (DMSO, 100 MHz)

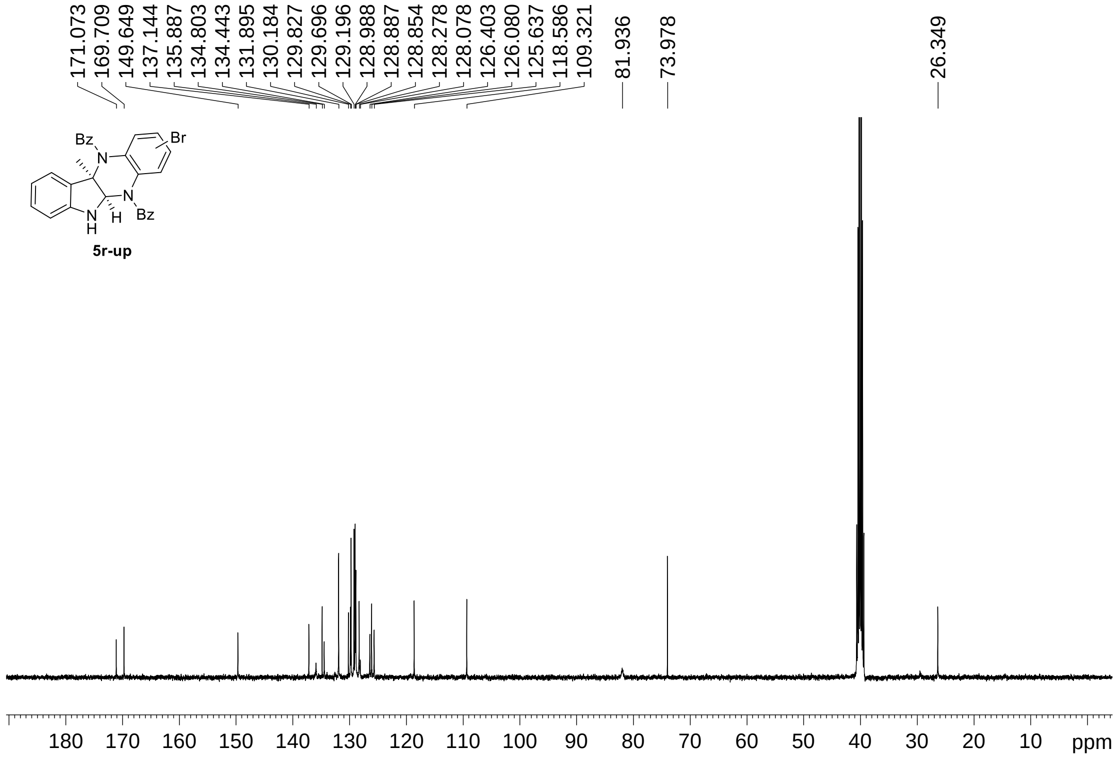

^{1}H NMR spectrum of compound **5r** (DMSO, 400 MHz)

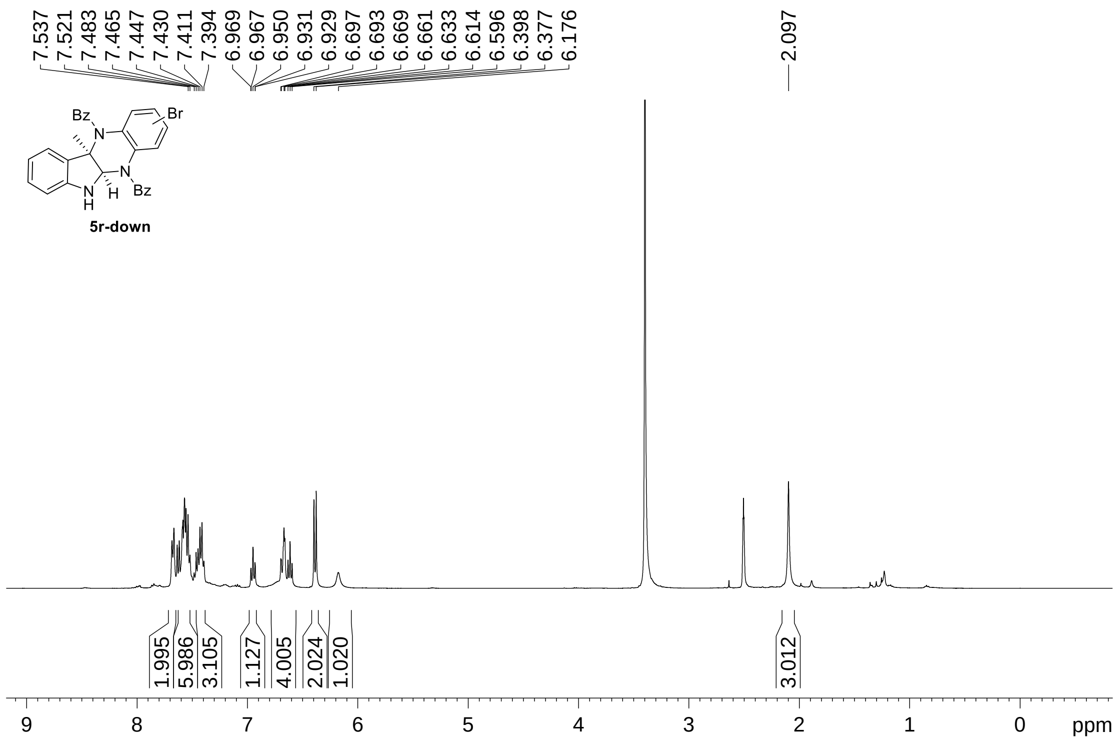

^{13}C NMR spectrum of compound **5r** (DMSO, 100 MHz)

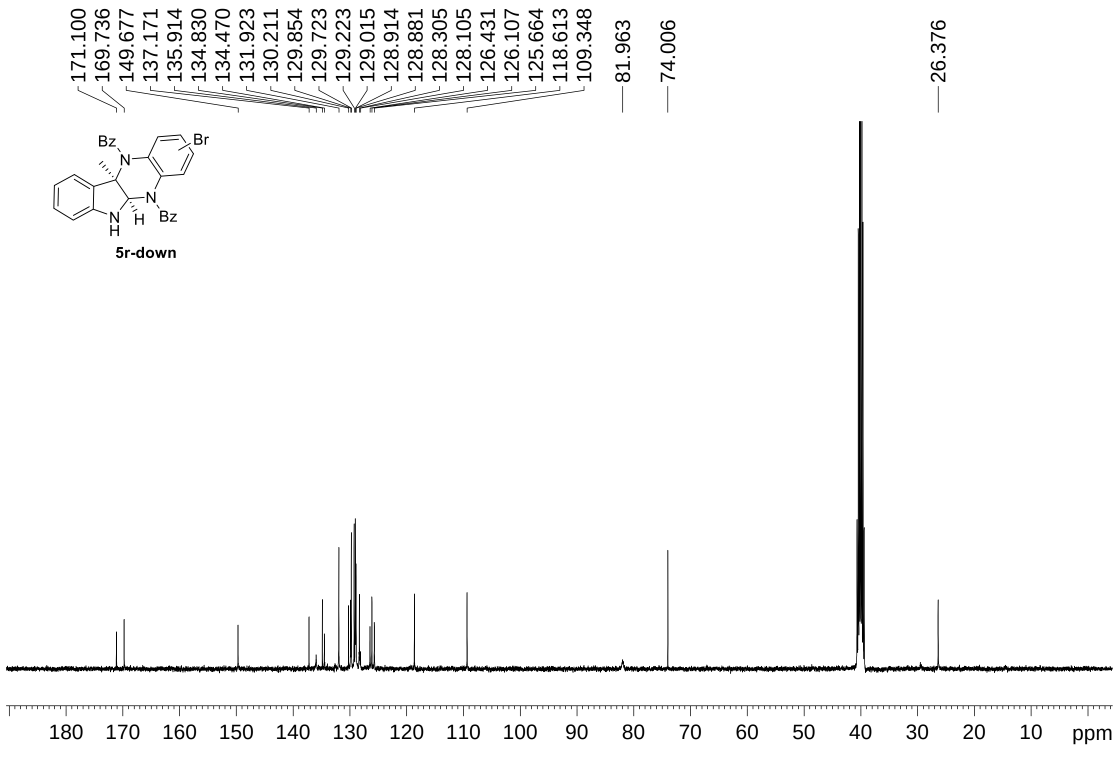

^{1}H NMR spectrum of compound **5s** (DMSO, 400 MHz)

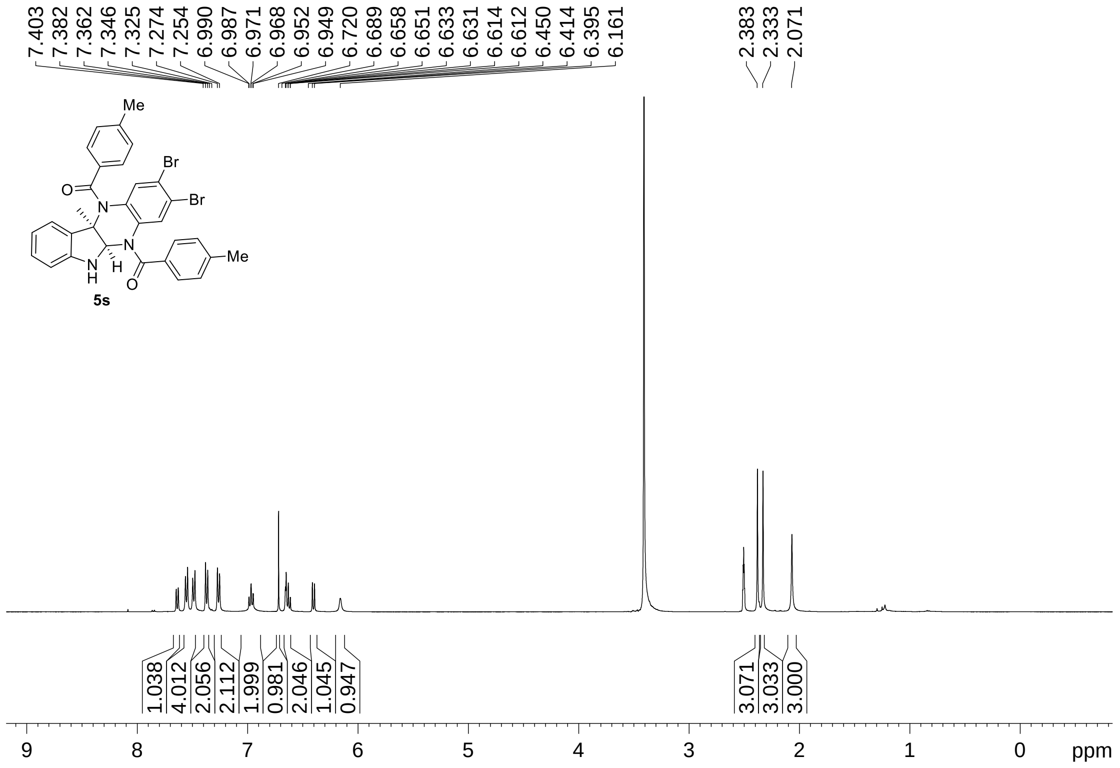

^{13}C NMR spectrum of compound **5s** (DMSO, 100 MHz)

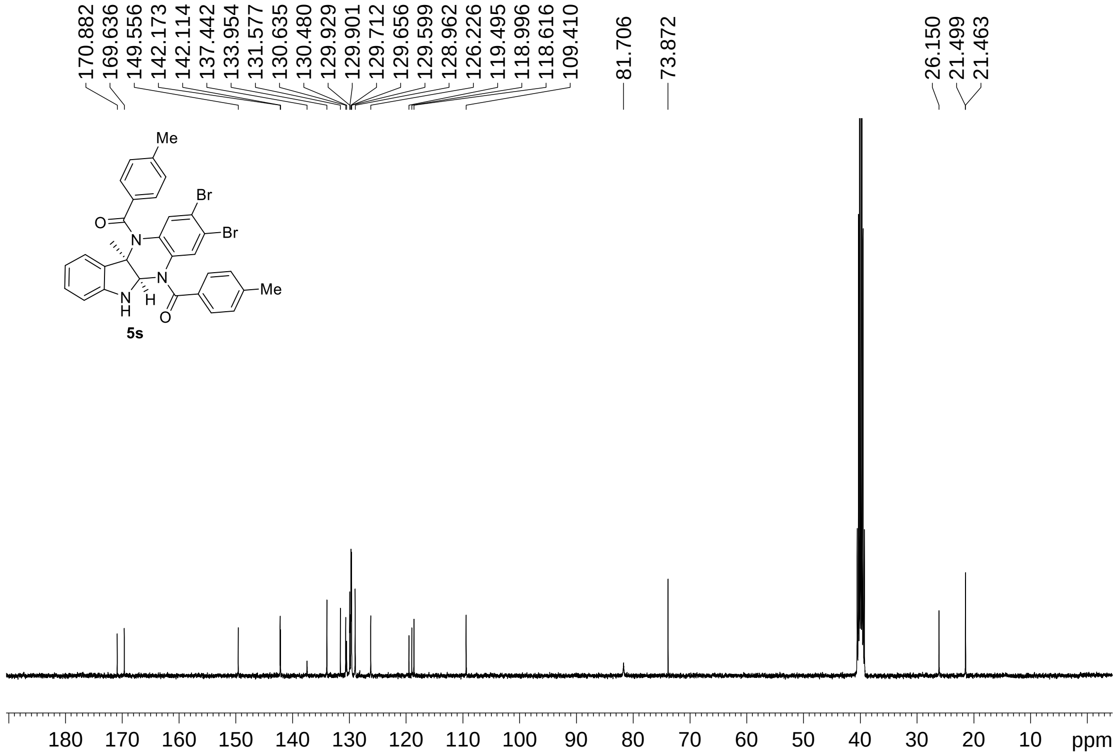

^{1}H NMR spectrum of compound **5t** (DMSO, 400 MHz)

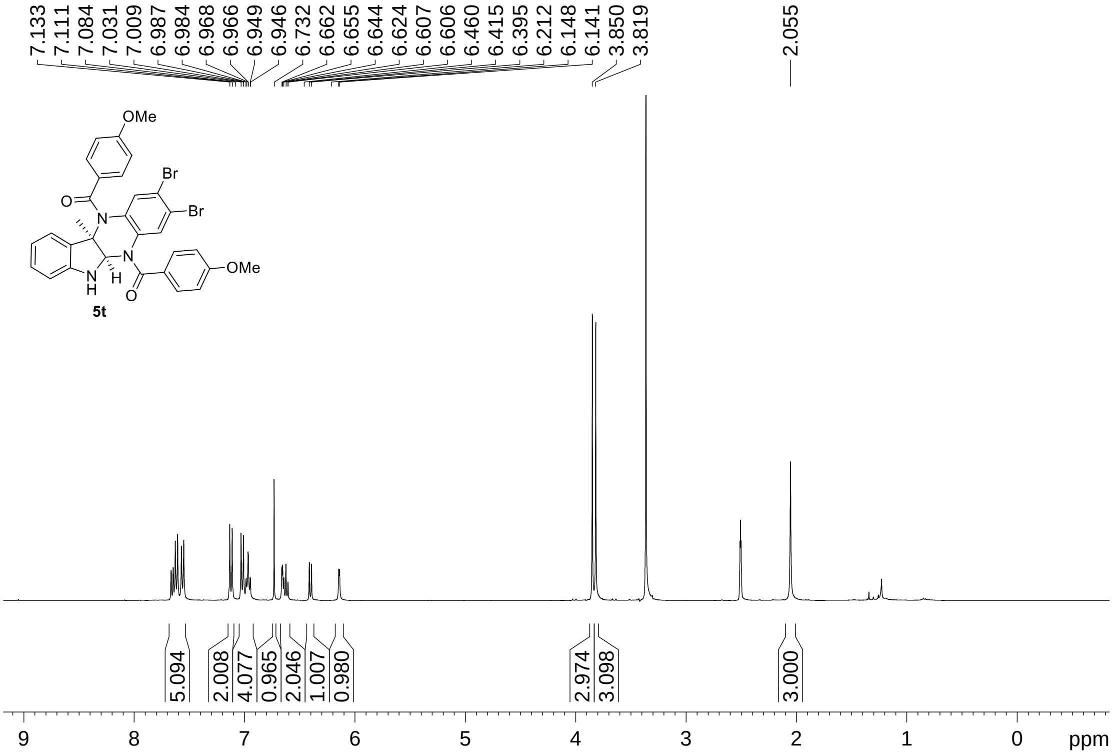

^{13}C NMR spectrum of compound **5t** (DMSO, 100 MHz)

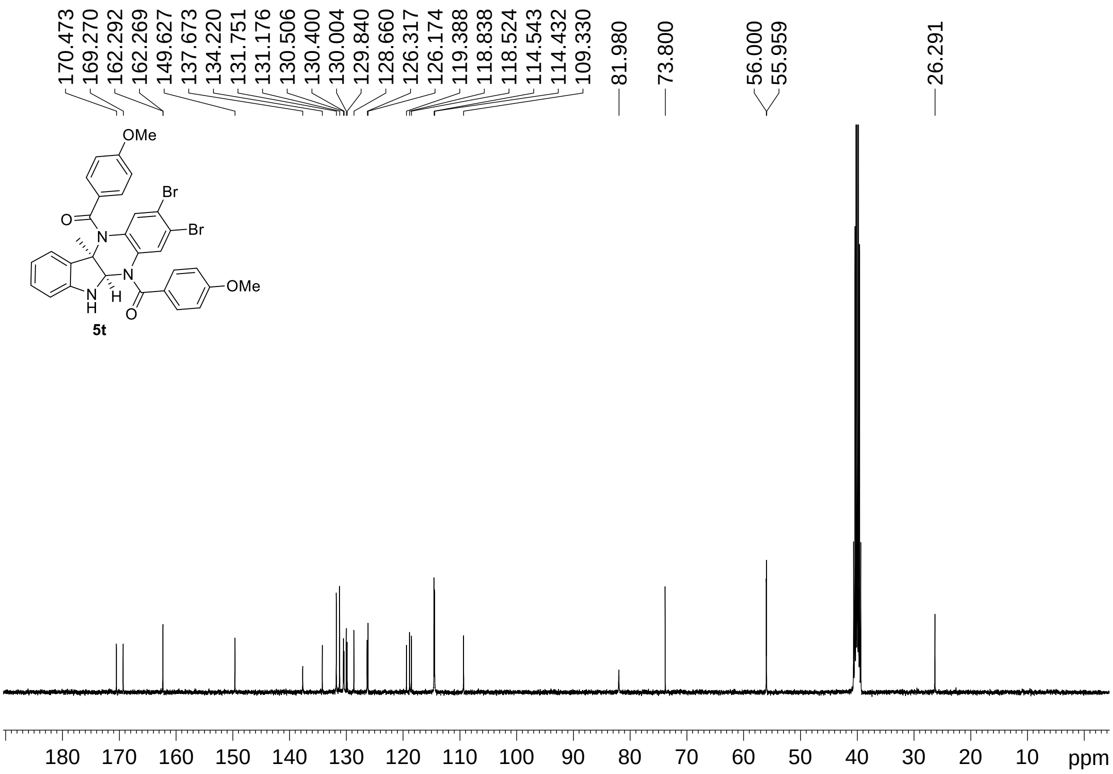

^{1}H NMR spectrum of compound **5u** (DMSO, 400 MHz)

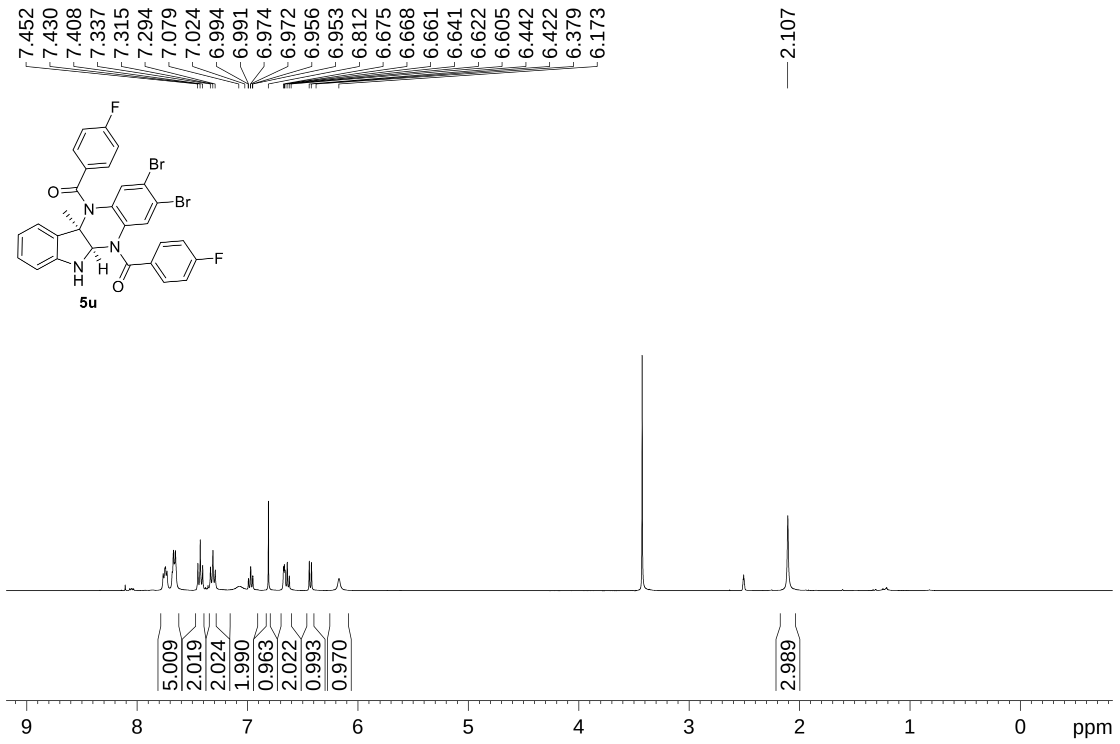

^{13}C NMR spectrum of compound **5u** (DMSO, 100 MHz)

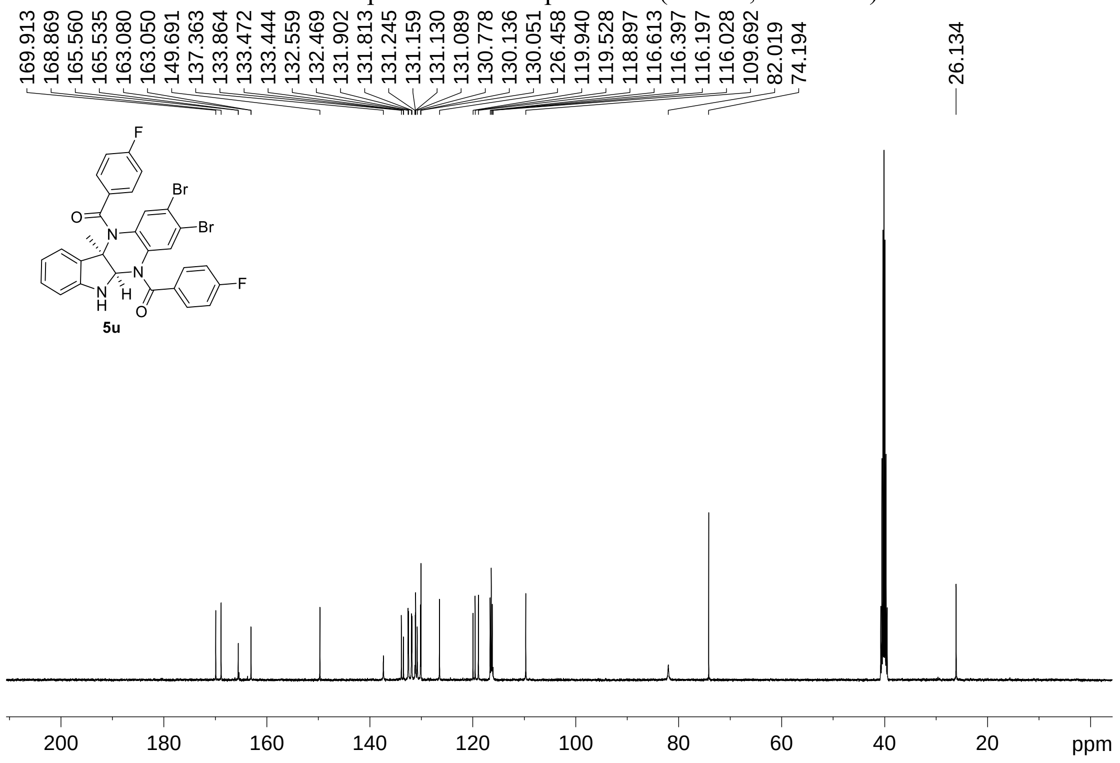

^{19}F NMR spectrum of compound **5u** (DMSO, 100 MHz)

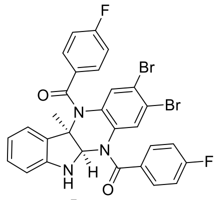

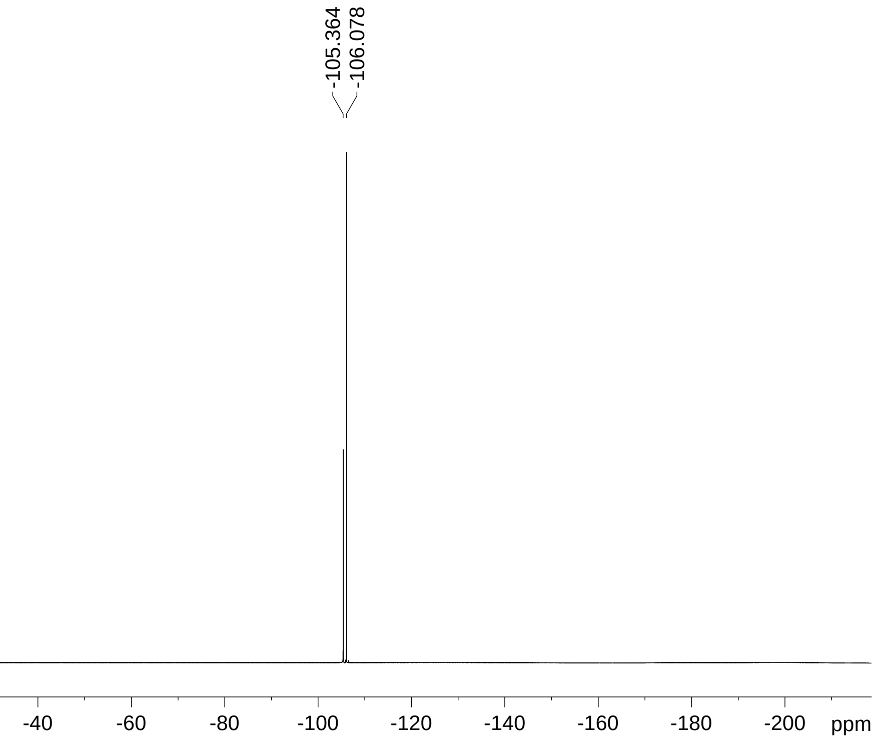

^{1}H NMR spectrum of compound **5v** (DMSO, 400 MHz)

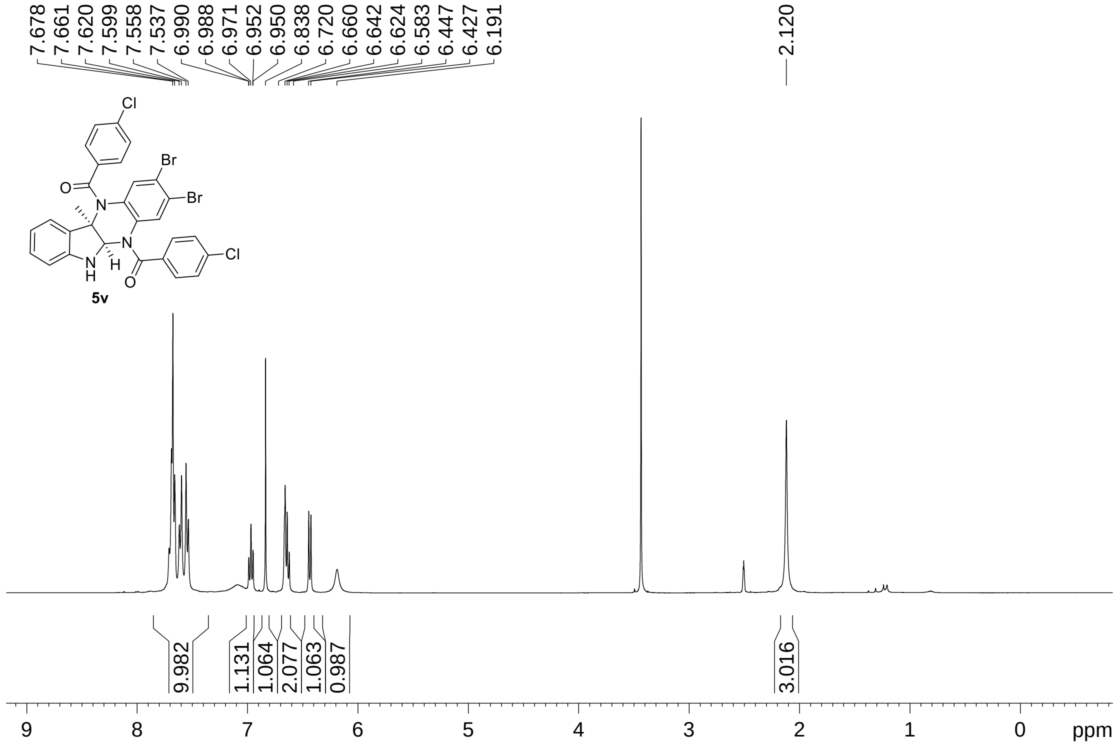

^{13}C NMR spectrum of compound **5v** (DMSO, 100 MHz)

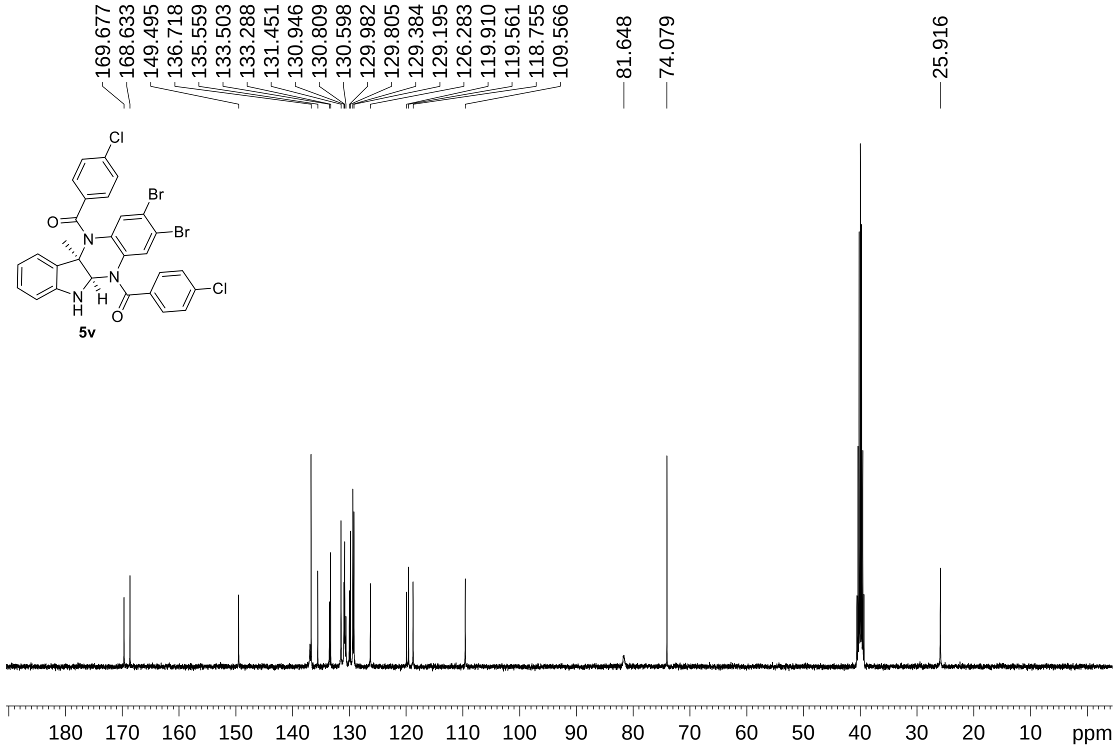

^{1}H NMR spectrum of compound **5w** (DMSO, 400 MHz)

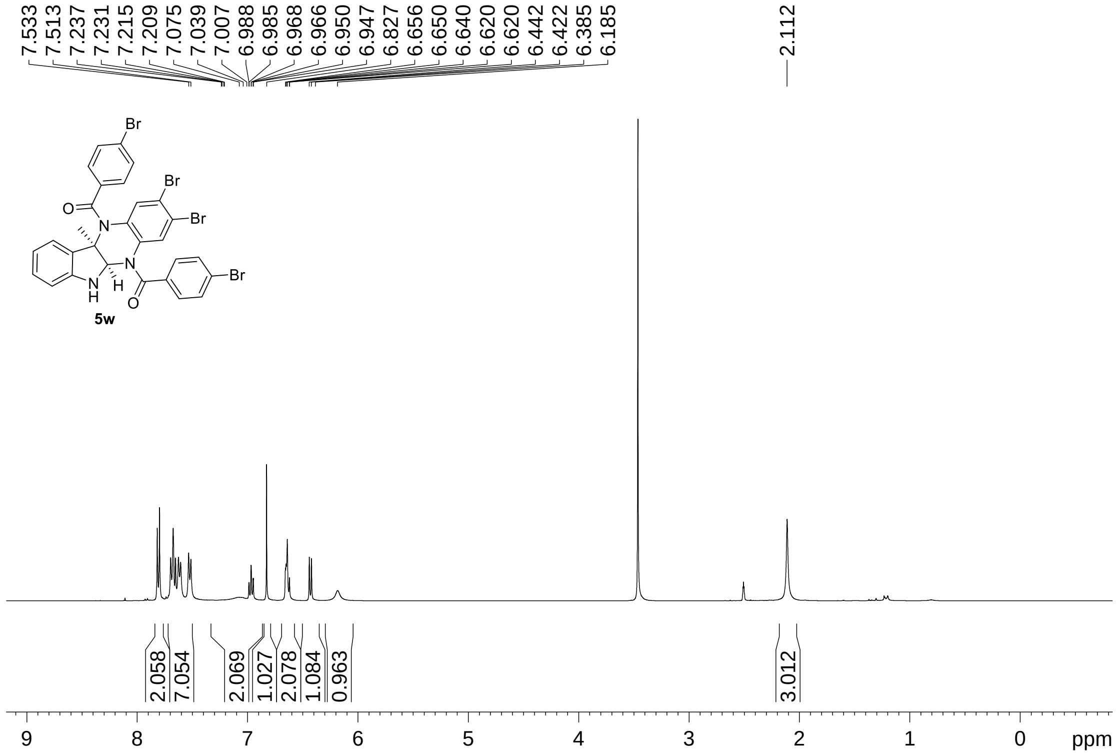

^{13}C NMR spectrum of compound **5w** (DMSO, 100 MHz)

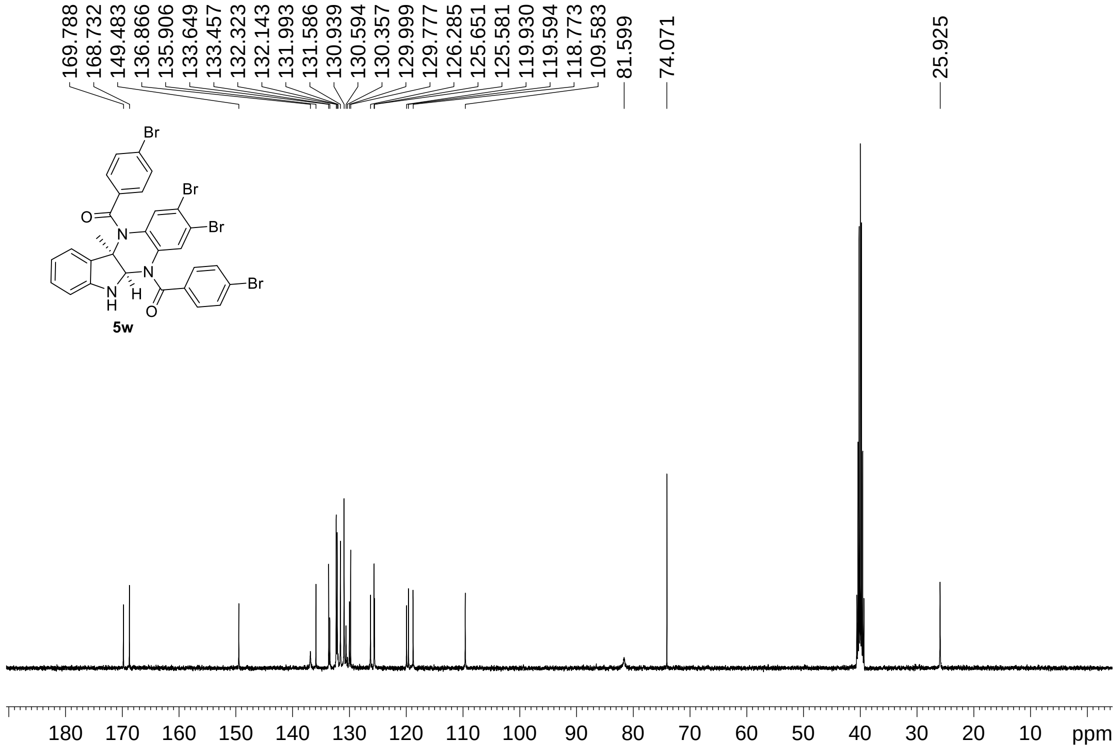

^{1}H NMR spectrum of compound **5x** ($CDCl_3$, 400 MHz)

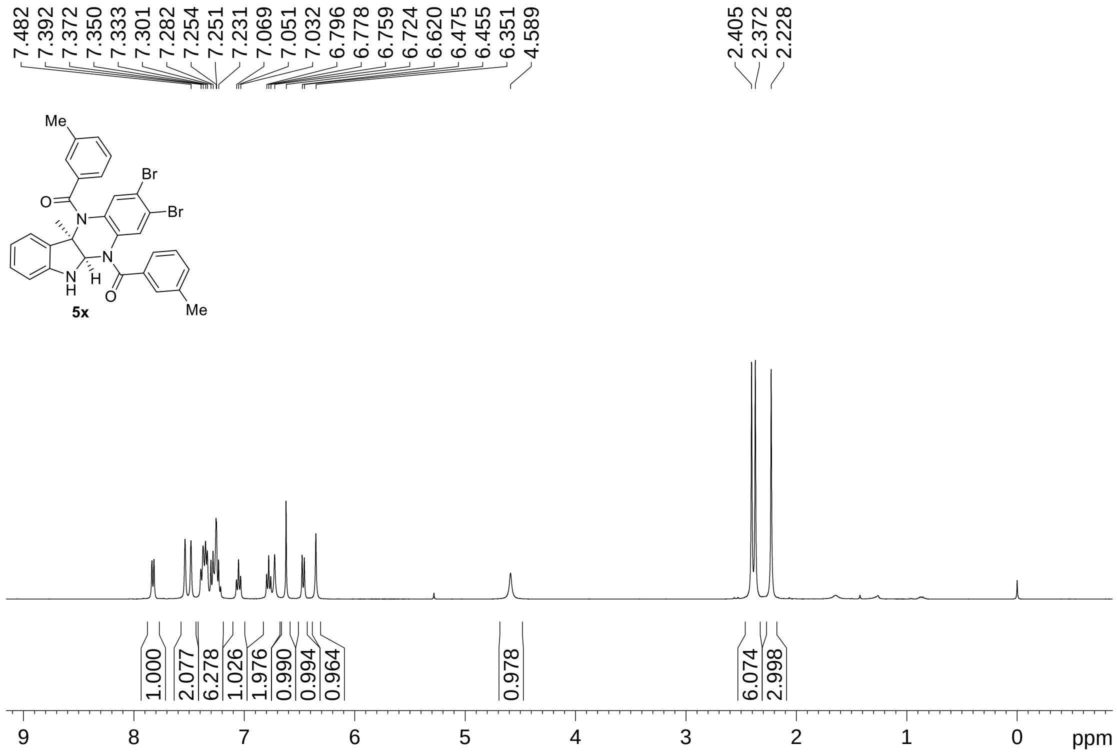

^{13}C NMR spectrum of compound **5w** ($CDCl_3$, 100 MHz)

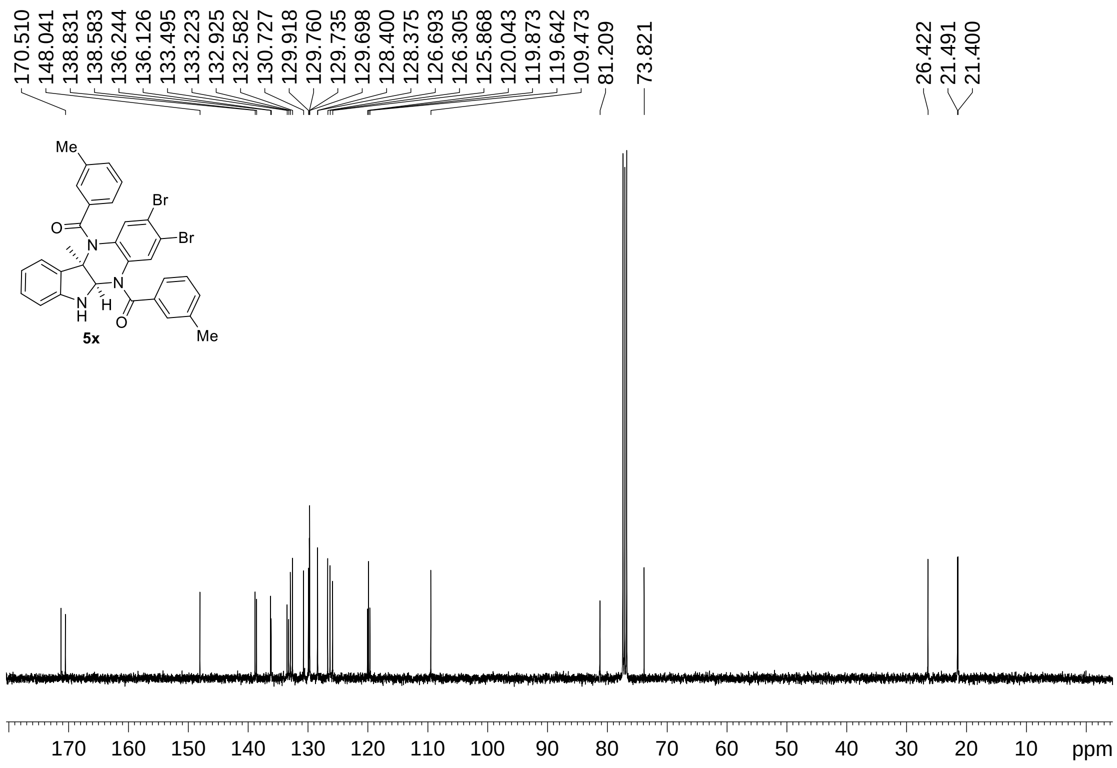

^{1}H NMR spectrum of compound **5y** (DMSO, 400 MHz)

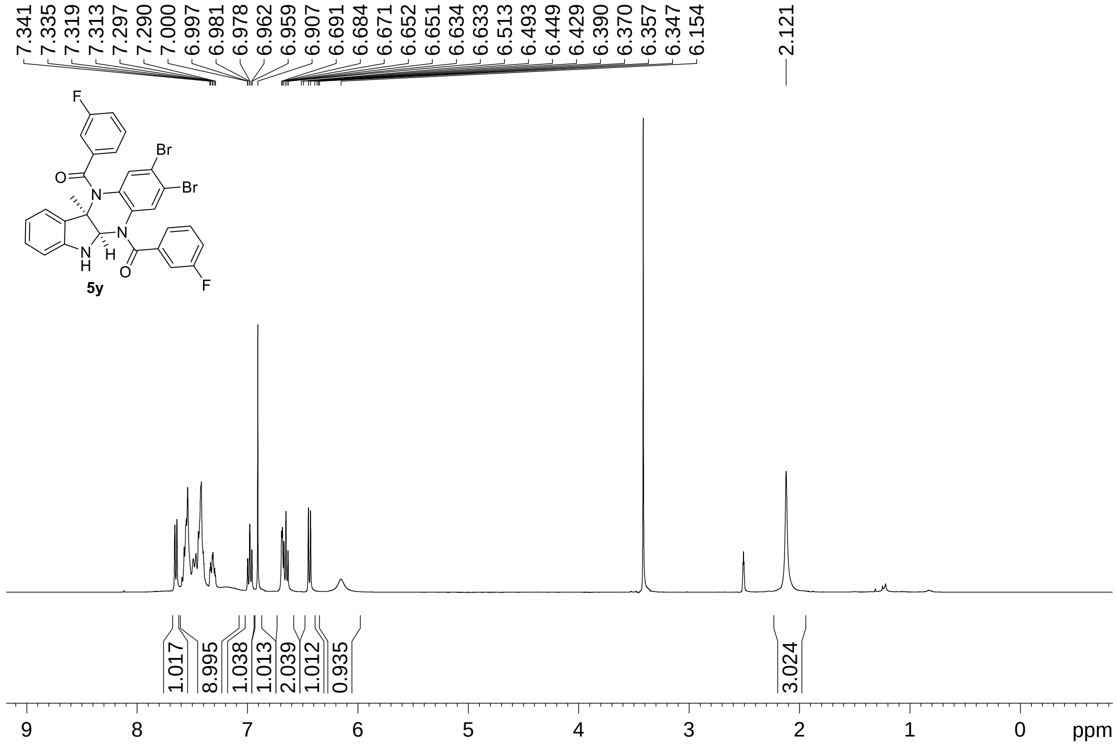

^{13}C NMR spectrum of compound **5y** (DMSO, 100 MHz)

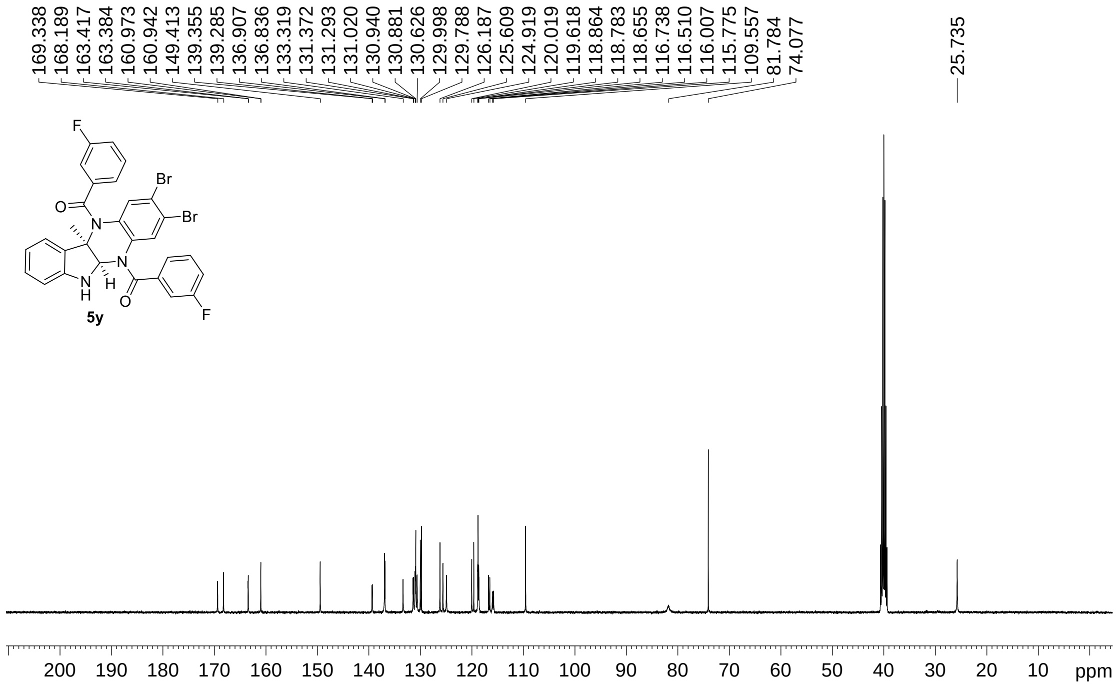

^{19}F NMR spectrum of compound **5y** (DMSO, 376 MHz)

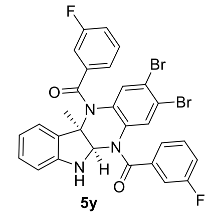

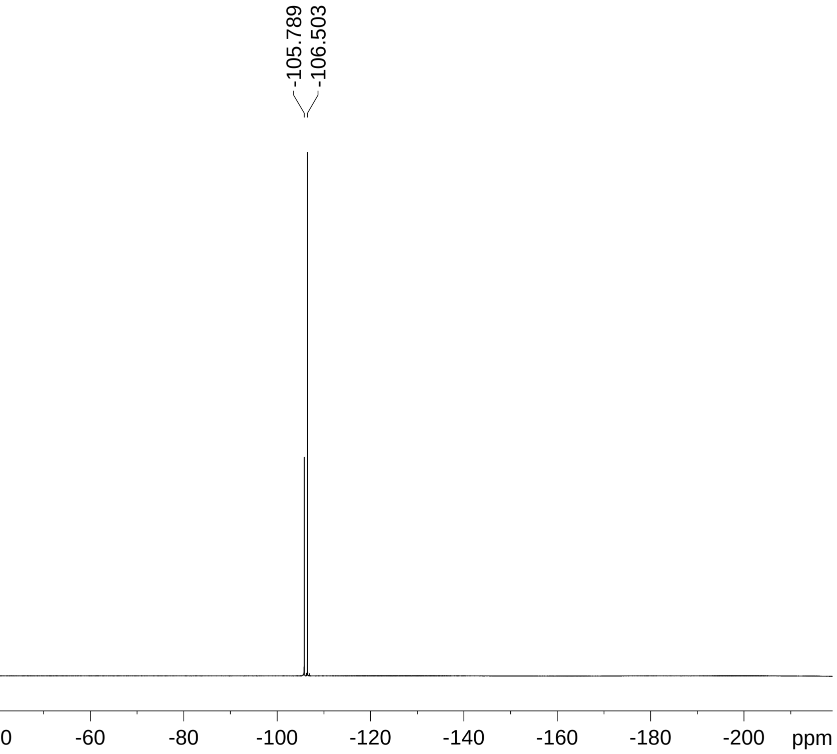

^{1}H NMR spectrum of compound **5z** (DMSO, 400 MHz)

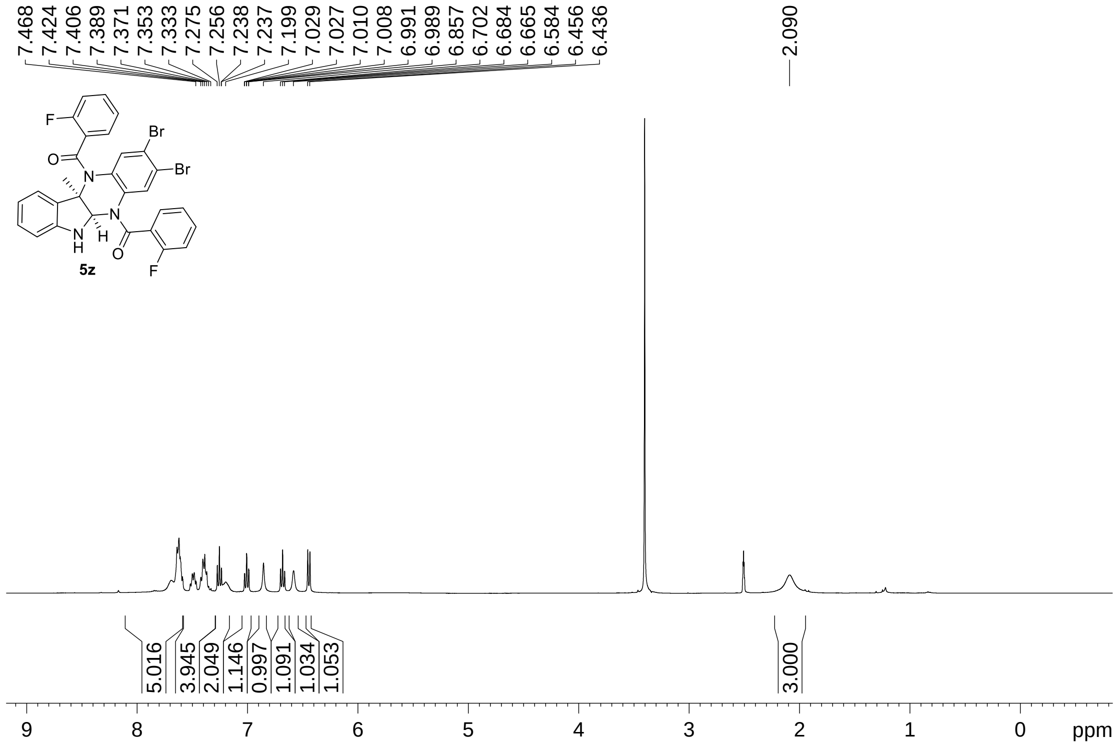

^{13}C NMR spectrum of compound **5z** (DMSO, 100 MHz)

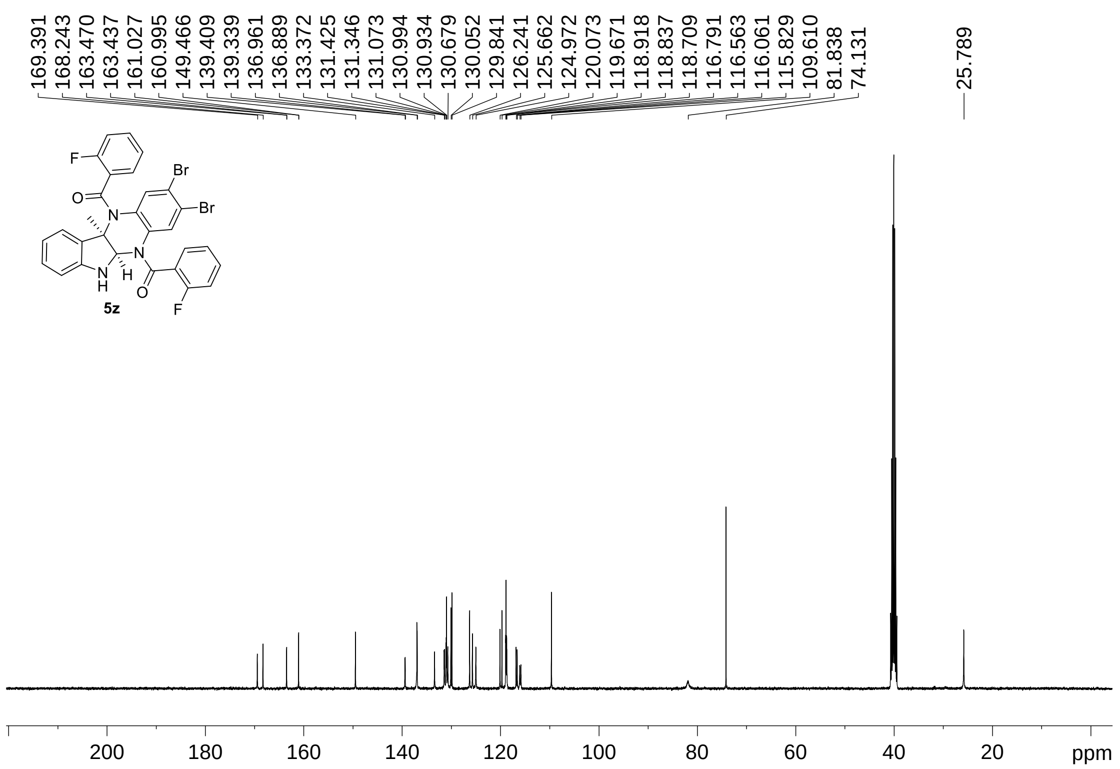

^{19}F NMR spectrum of compound **5z** ($(CD_3)_2SO$, 376 MHz)

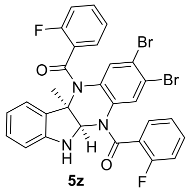

5z

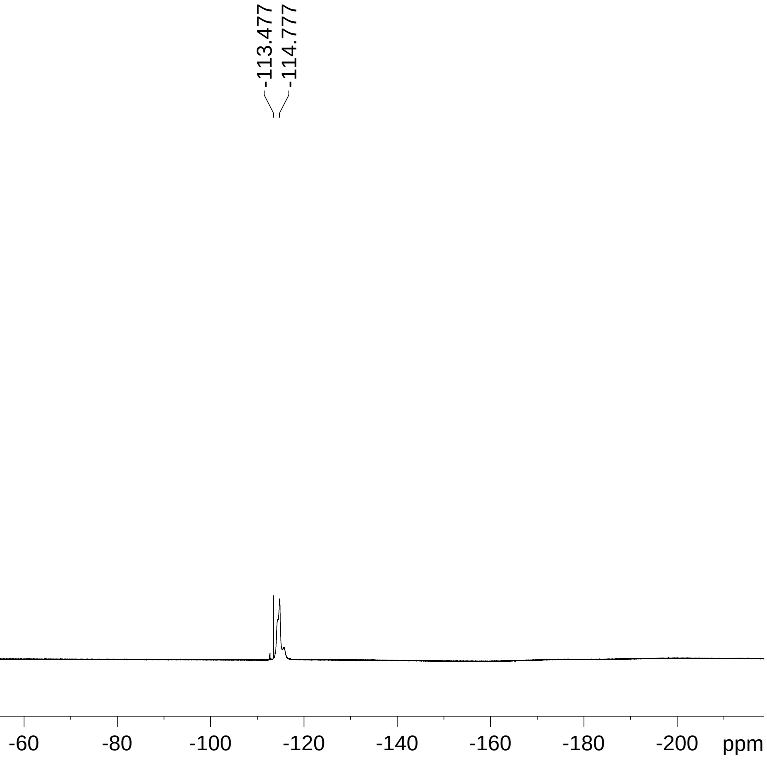

^{1}H NMR spectrum of compound **5a'** (DMSO, 400 MHz)

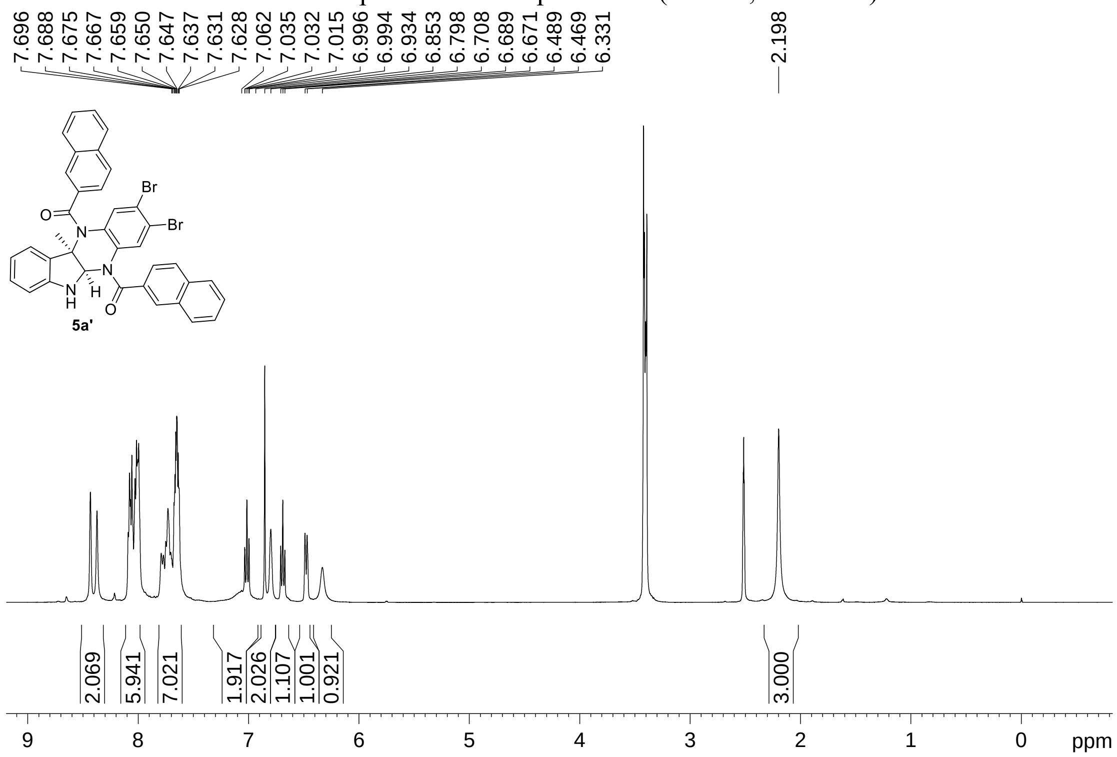

^{13}C NMR spectrum of compound **5a'** (DMSO, 100 MHz)

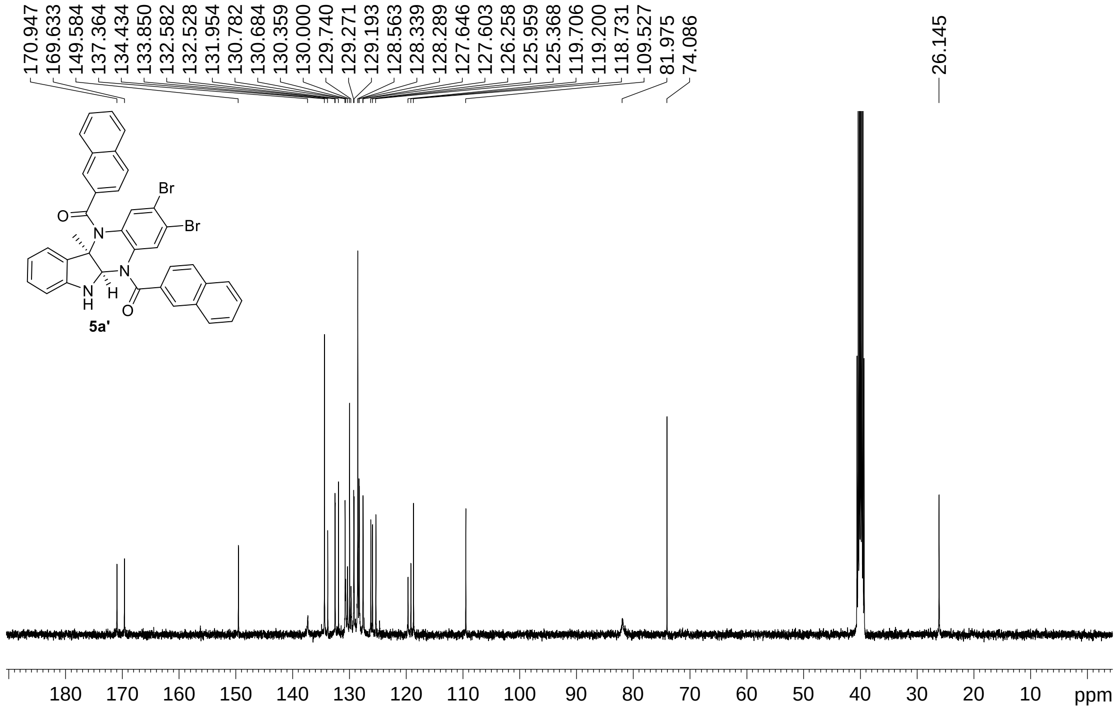

^{1}H NMR spectrum of compound **5b'** (DMSO, 400 MHz)

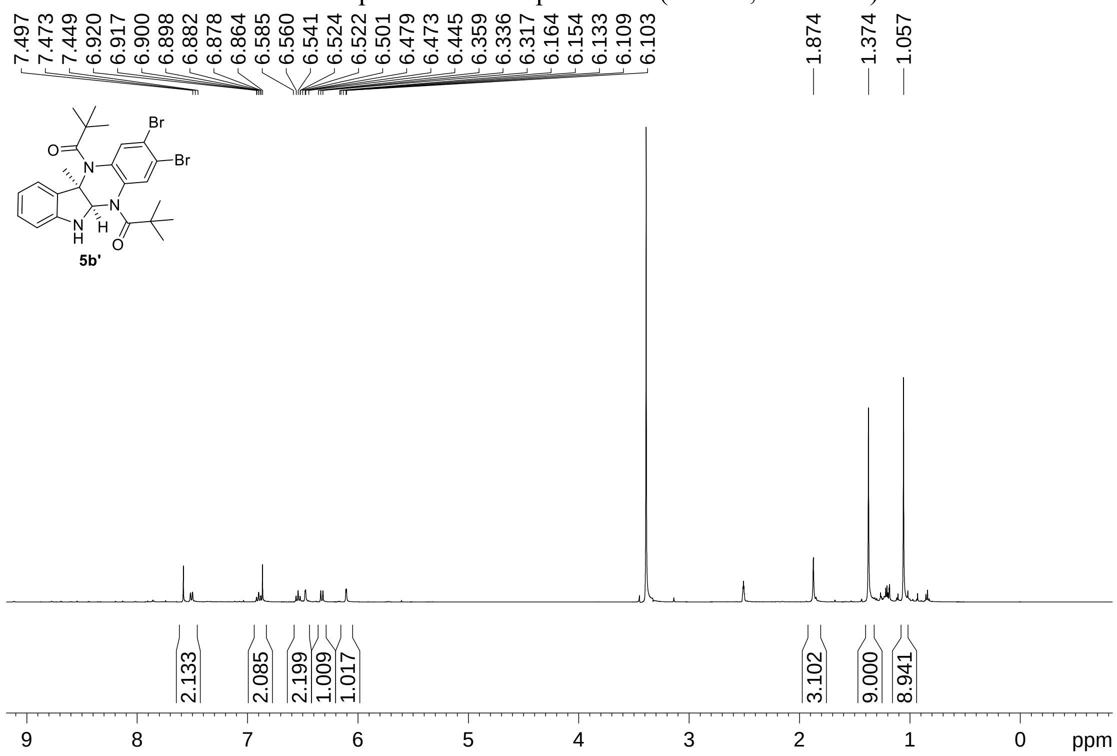

^{13}C NMR spectrum of compound **5b'** (DMSO, 100 MHz)

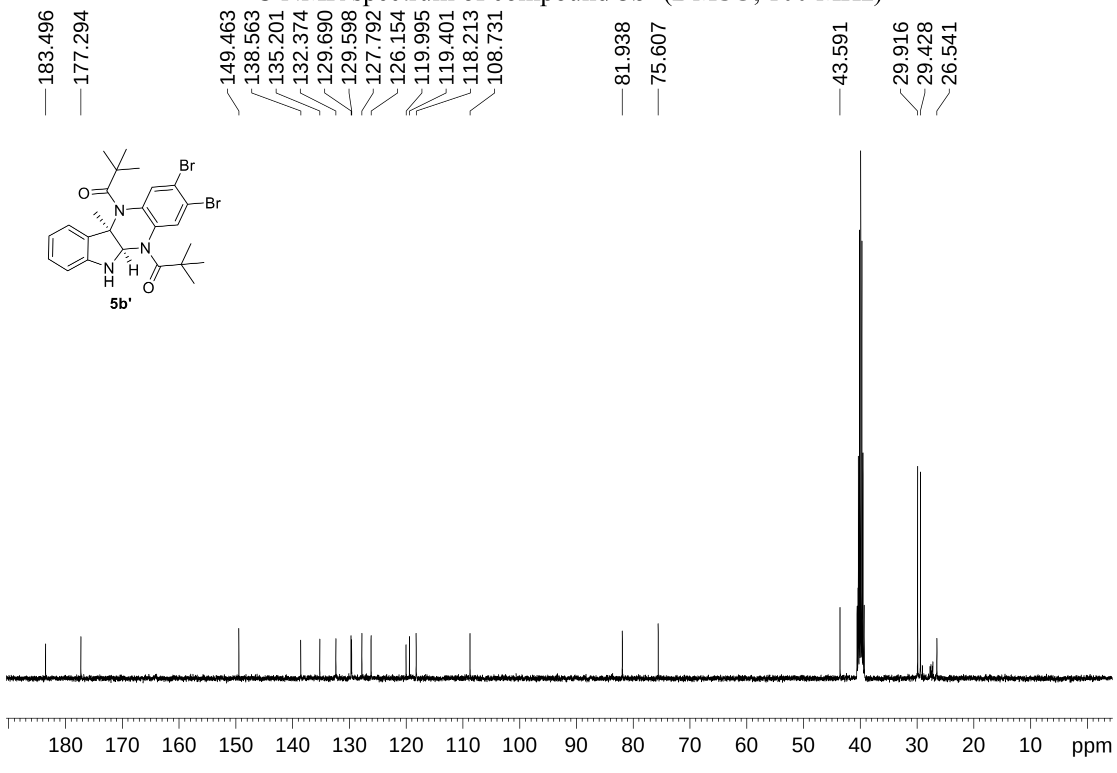

^{1}H NMR spectrum of compound **5c'** (DMSO, 400 MHz)

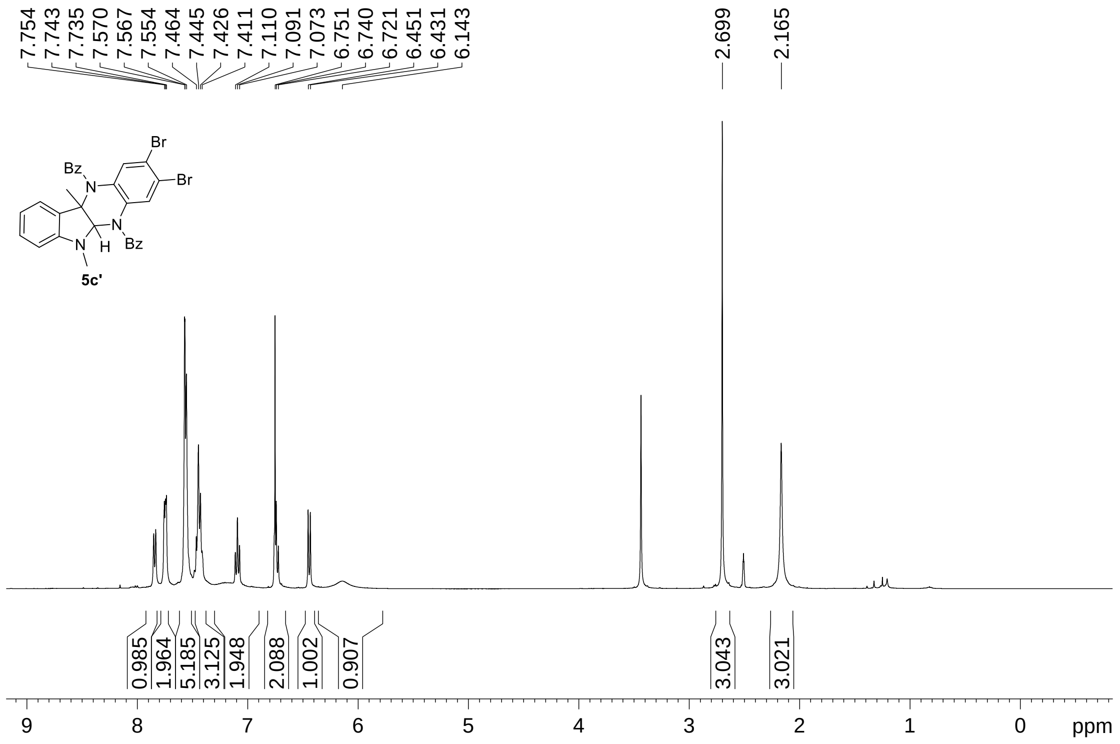

^{13}C NMR spectrum of compound **5c'** (DMSO, 100 MHz)

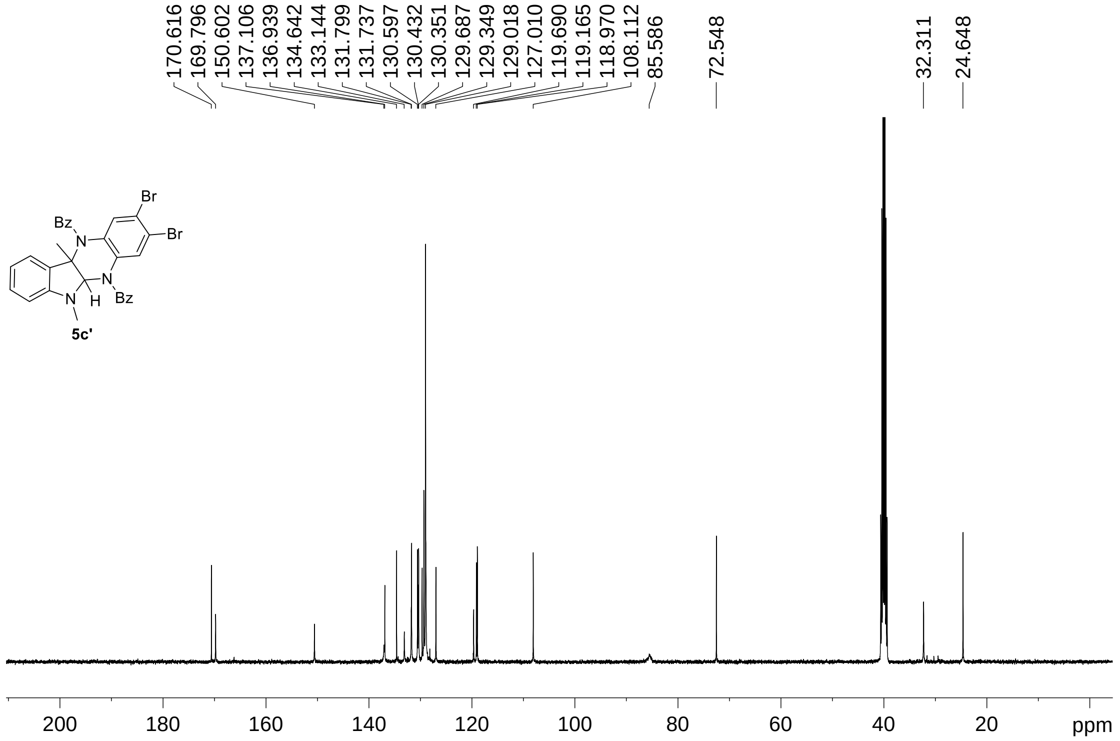

^{1}H NMR spectrum of compound **6** (($(CD_3)_2SO$, 400 MHz)

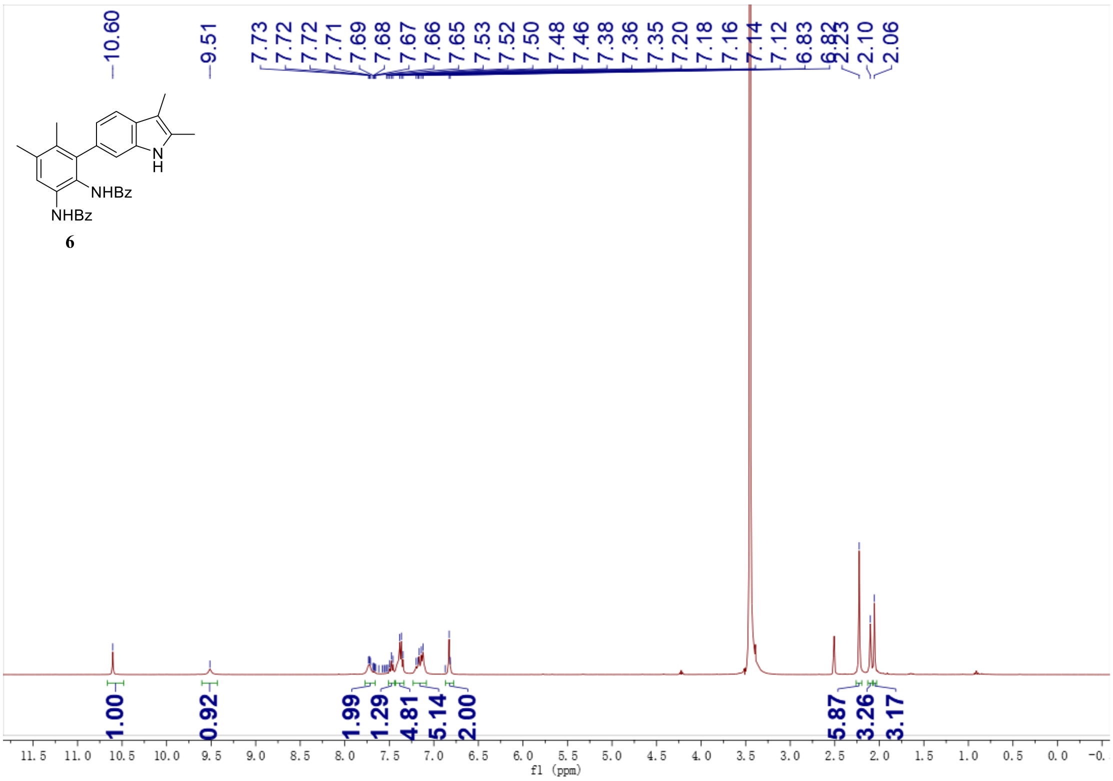

^{13}C NMR spectrum of compound **6** (($(CD_3)_2SO$, 100 MHz)

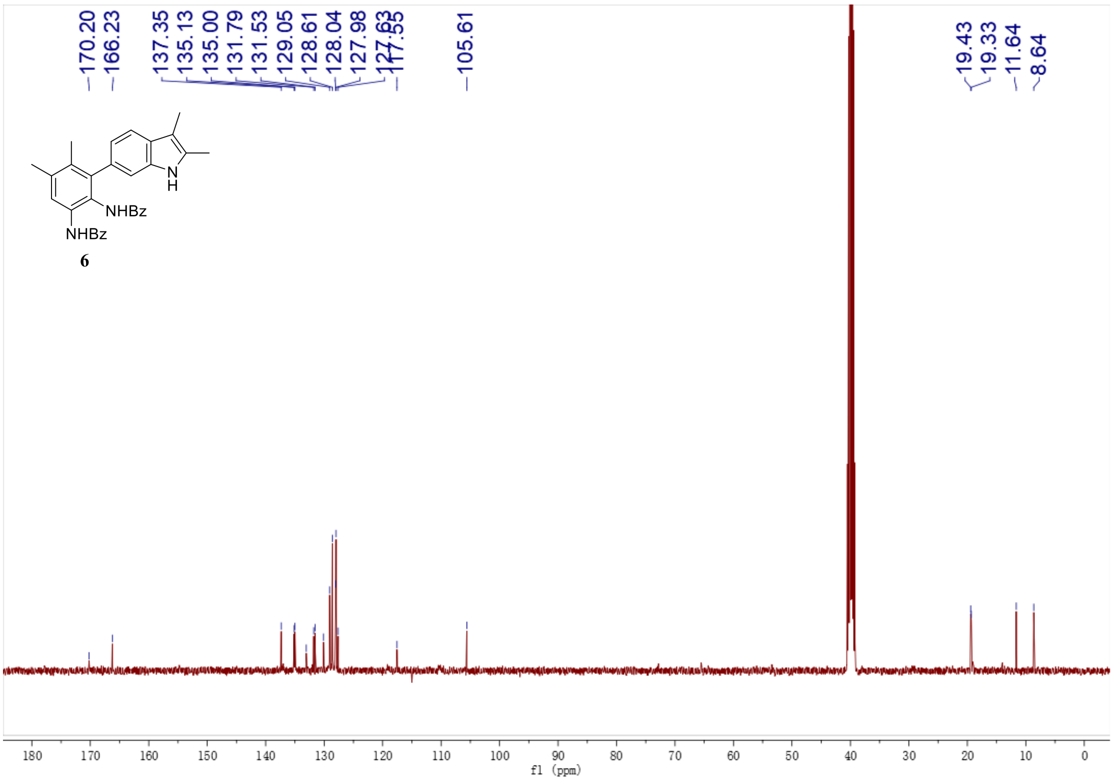

^{1}H NMR spectrum of compound **7** (dr = 6:1) (($(CD_3)_2SO$, 400 MHz)

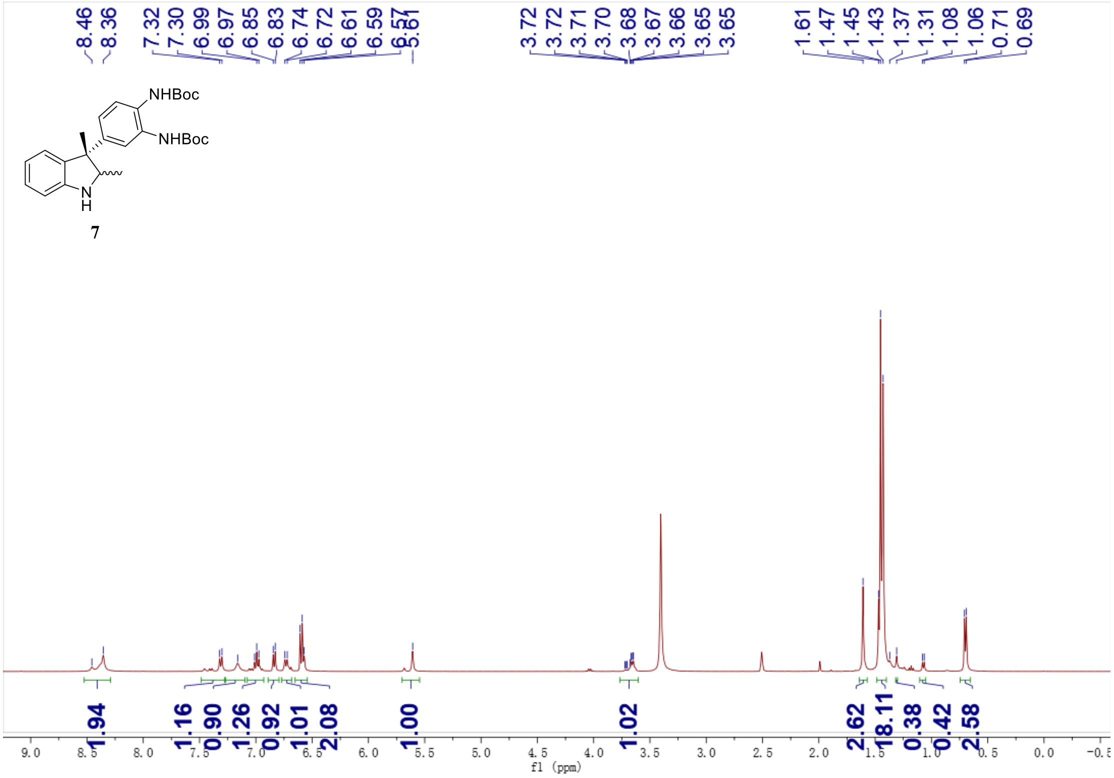

^{13}C NMR spectrum of compound **7** (dr = 6:1) (($(CD_3)_2SO$, 100 MHz)

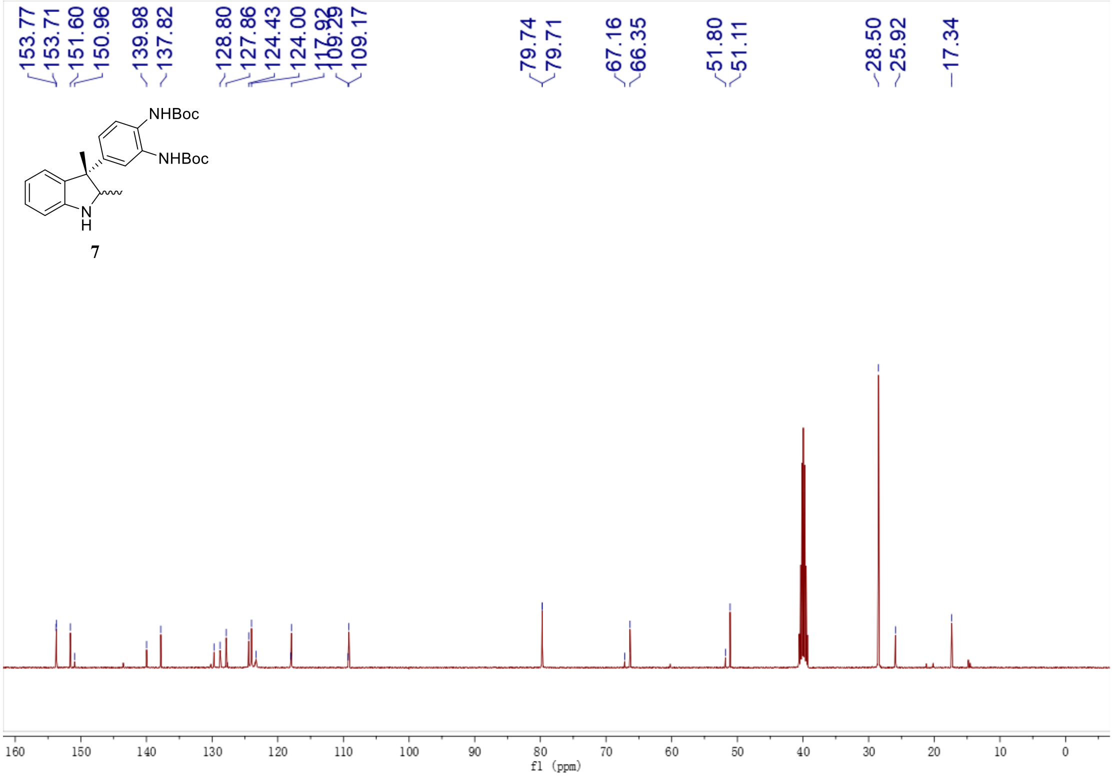

^{1}H NMR spectrum of compound **8** ($(CD_3)_2SO$, 400 MHz)

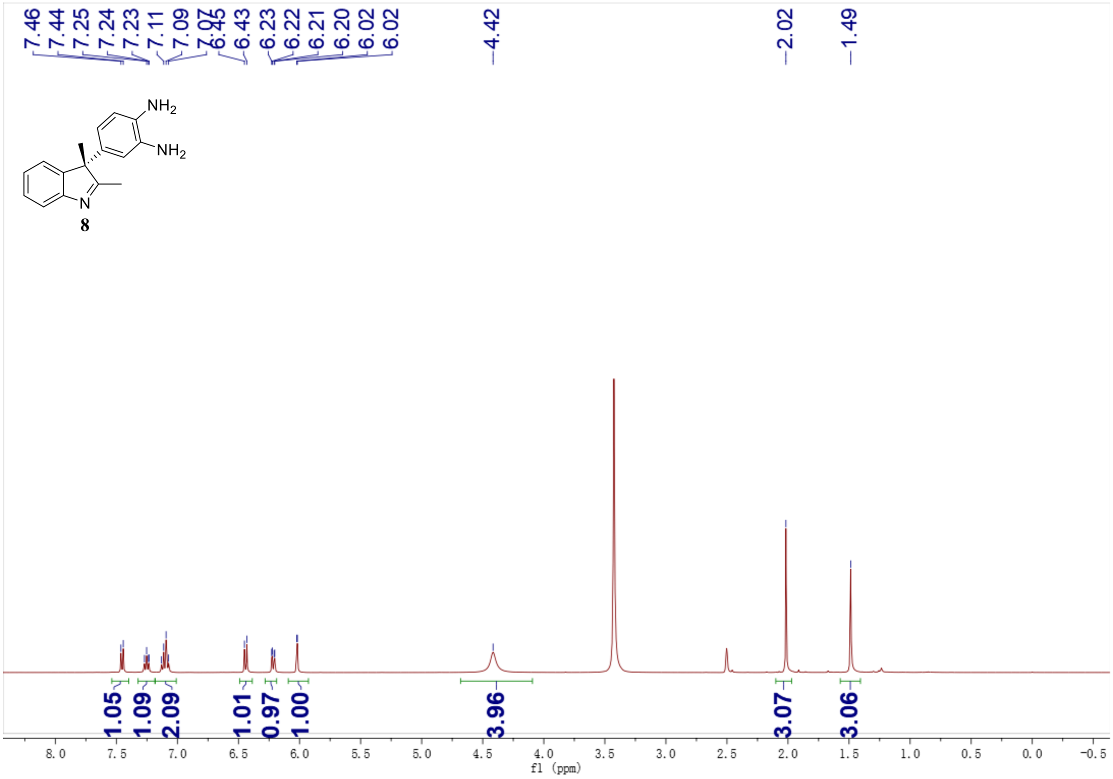

^{13}C NMR spectrum of compound **8** ($(CD_3)_2SO$, 100 MHz)

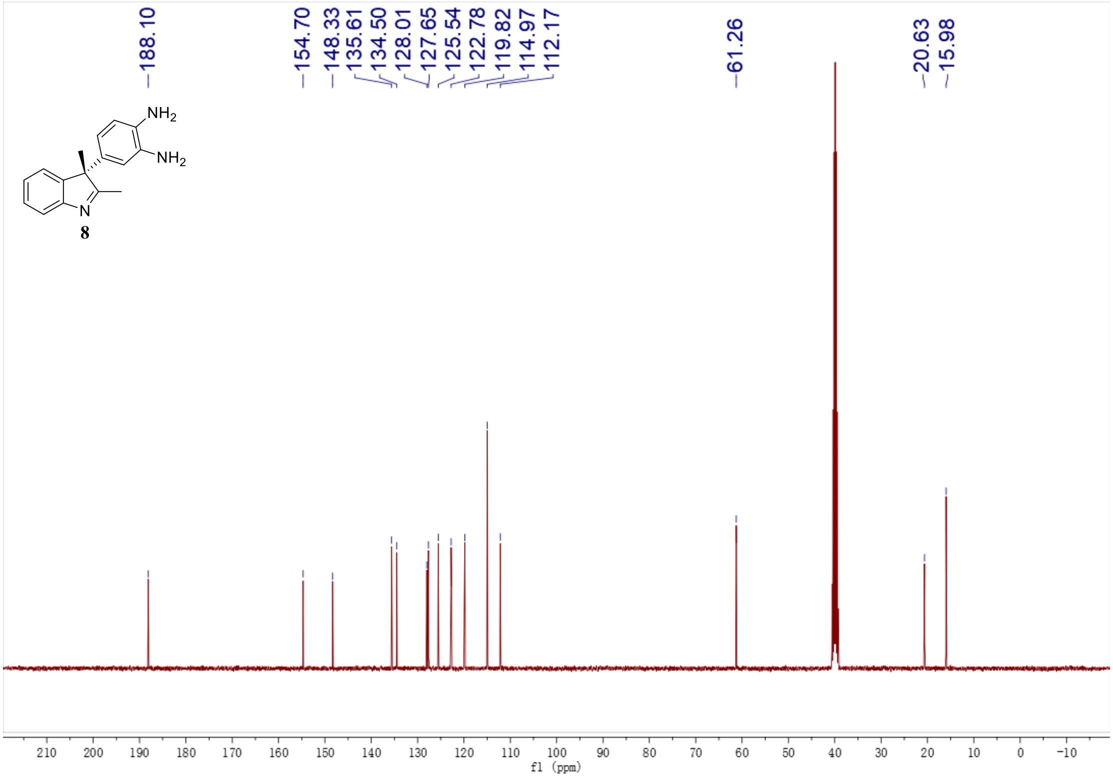

^{1}H NMR spectrum of compound **9** ($(CD_3)_2SO$, 400 MHz)

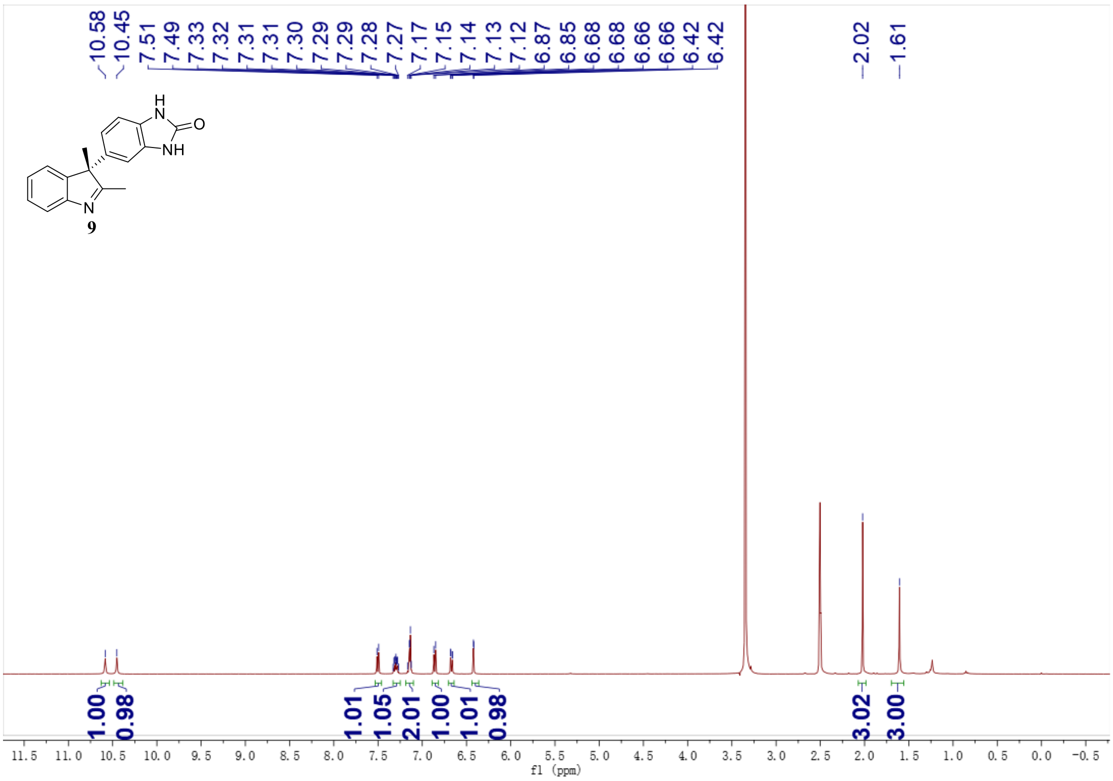

^{13}C NMR spectrum of compound **9** ($(CD_3)_2SO$, 100 MHz)

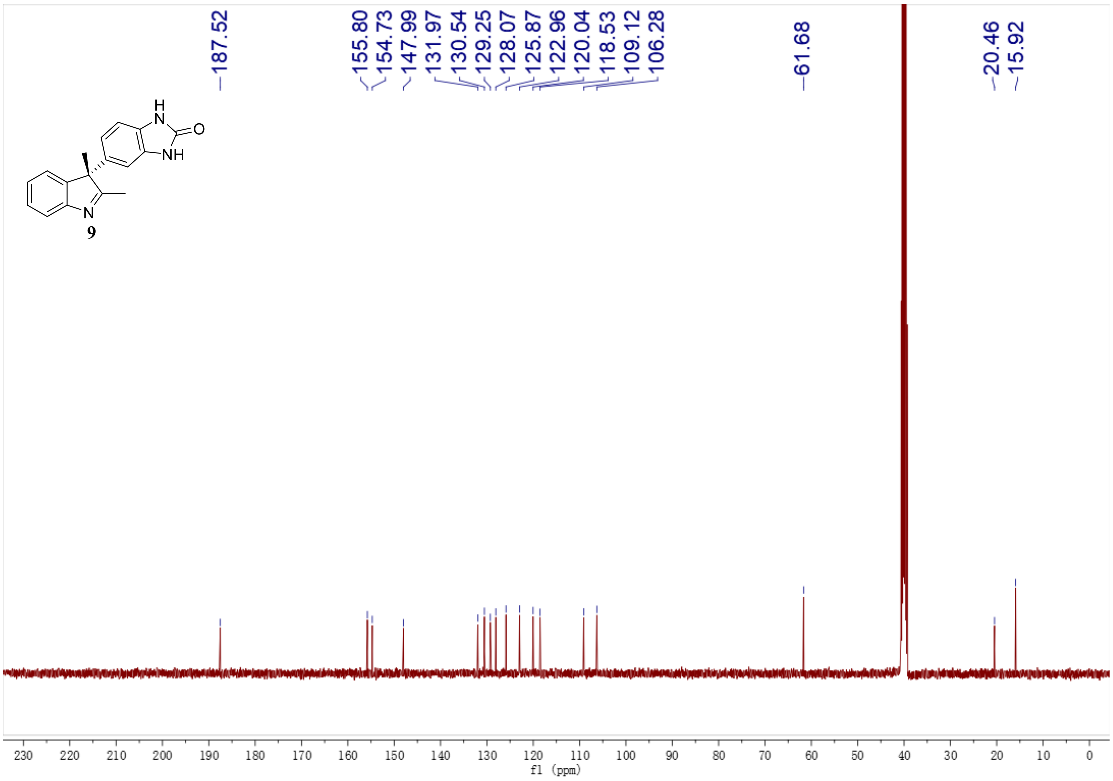

^1H NMR spectrum of compound **10** ($(CD_3)_2SO$, 400 MHz)

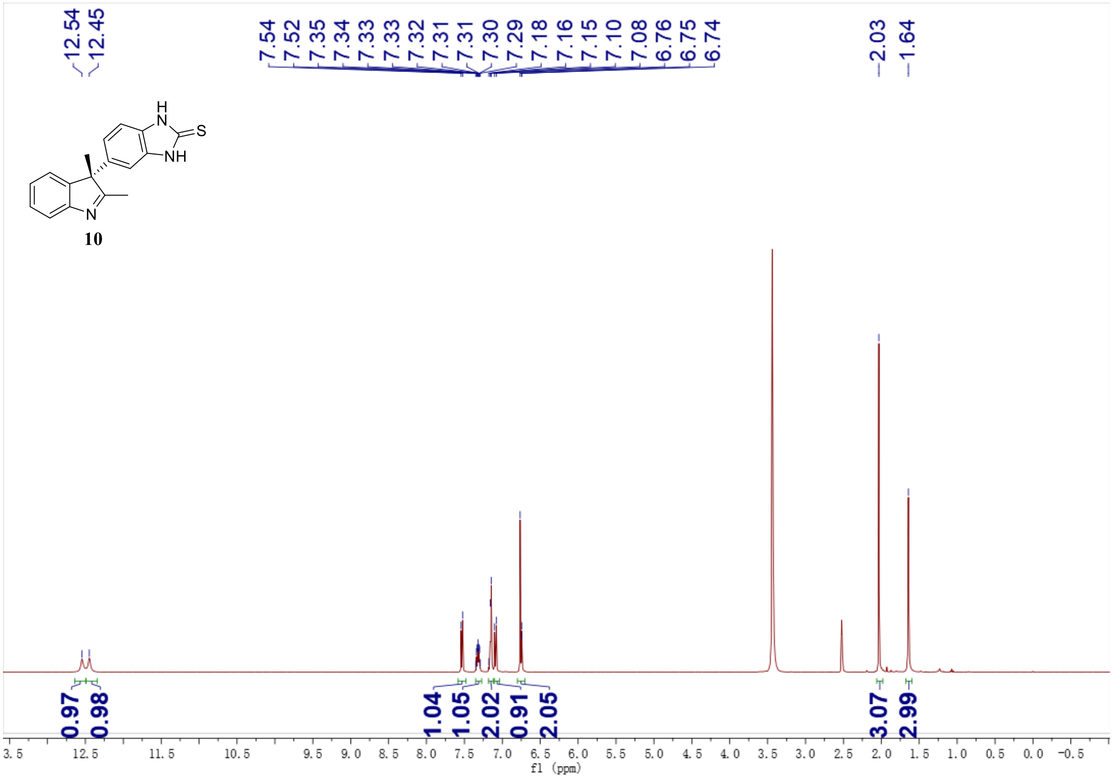

^{13}C NMR spectrum of compound **10** ($(CD_3)_2SO$, 100 MHz)

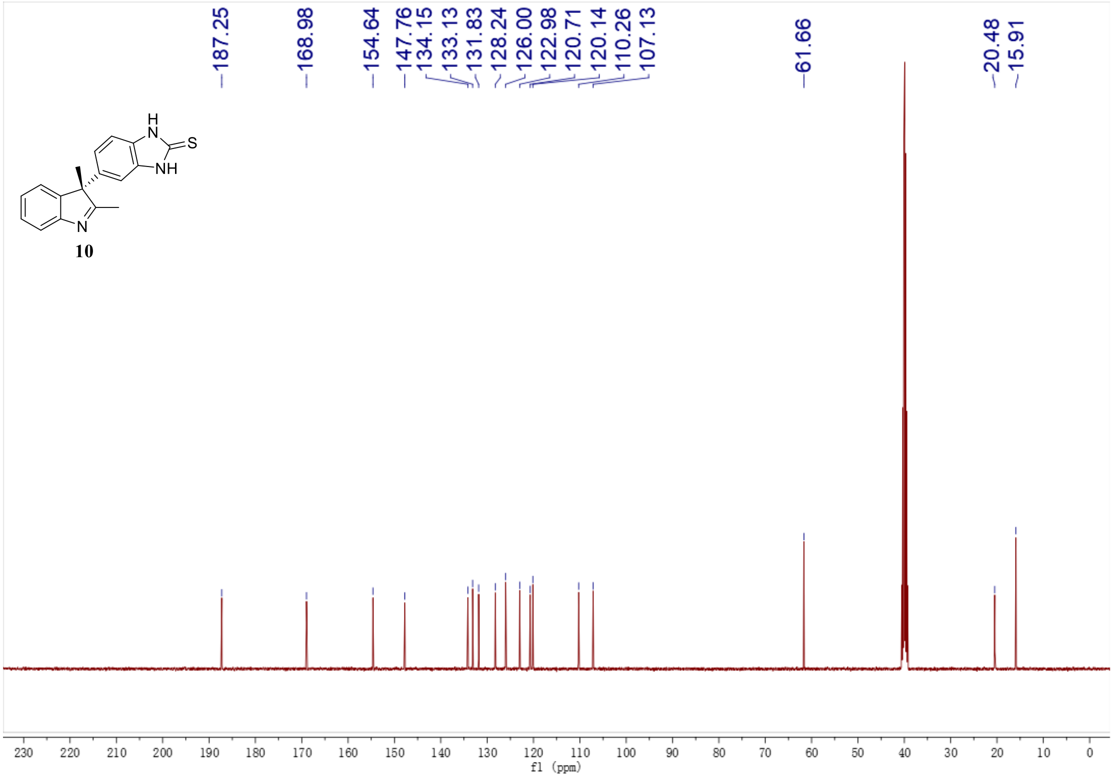

^{1}H NMR spectrum of compound **11** ($(CD_3)_2SO$, 400 MHz)

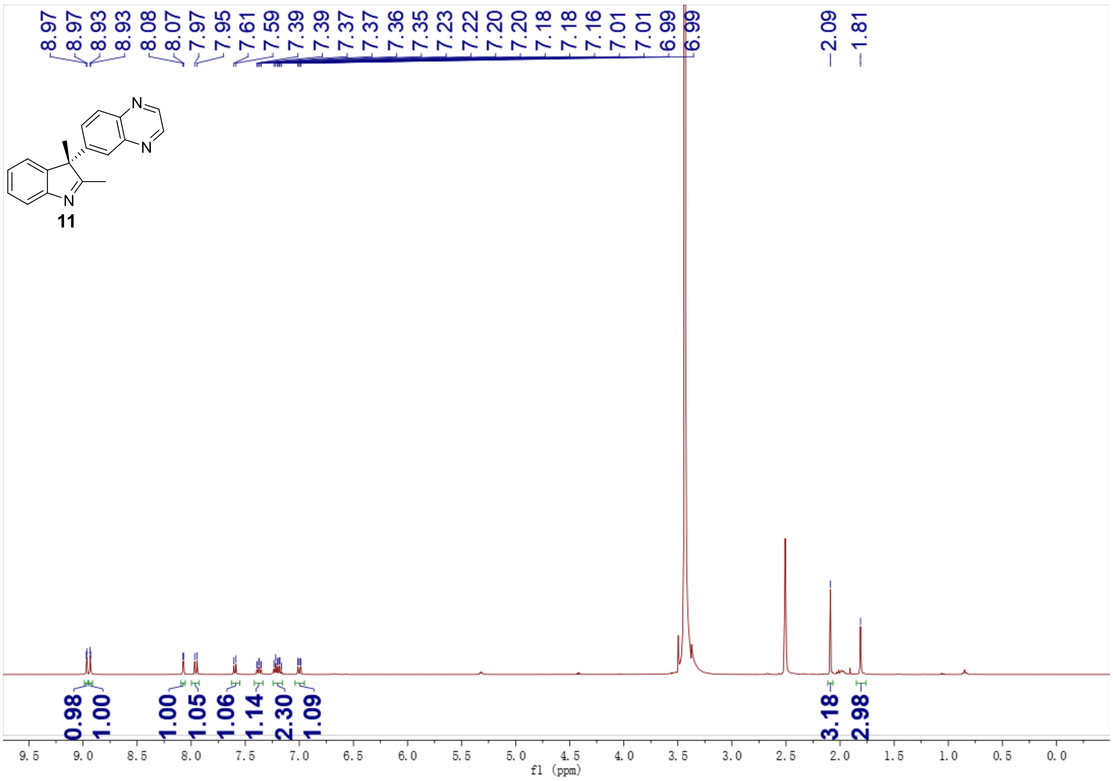

^{13}C NMR spectrum of compound **11** ($(CD_3)_2SO$, 100 MHz)

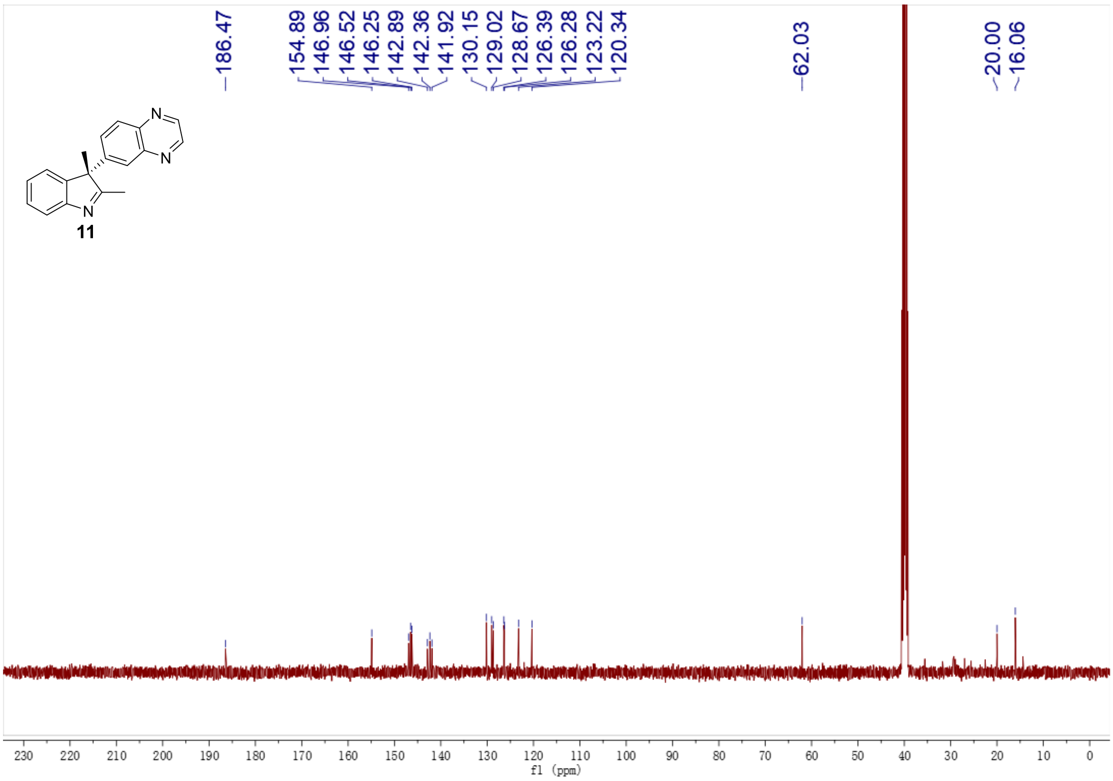

^{1}H NMR spectrum of compound **13** ($(CD_3)_2SO$, 400 MHz)

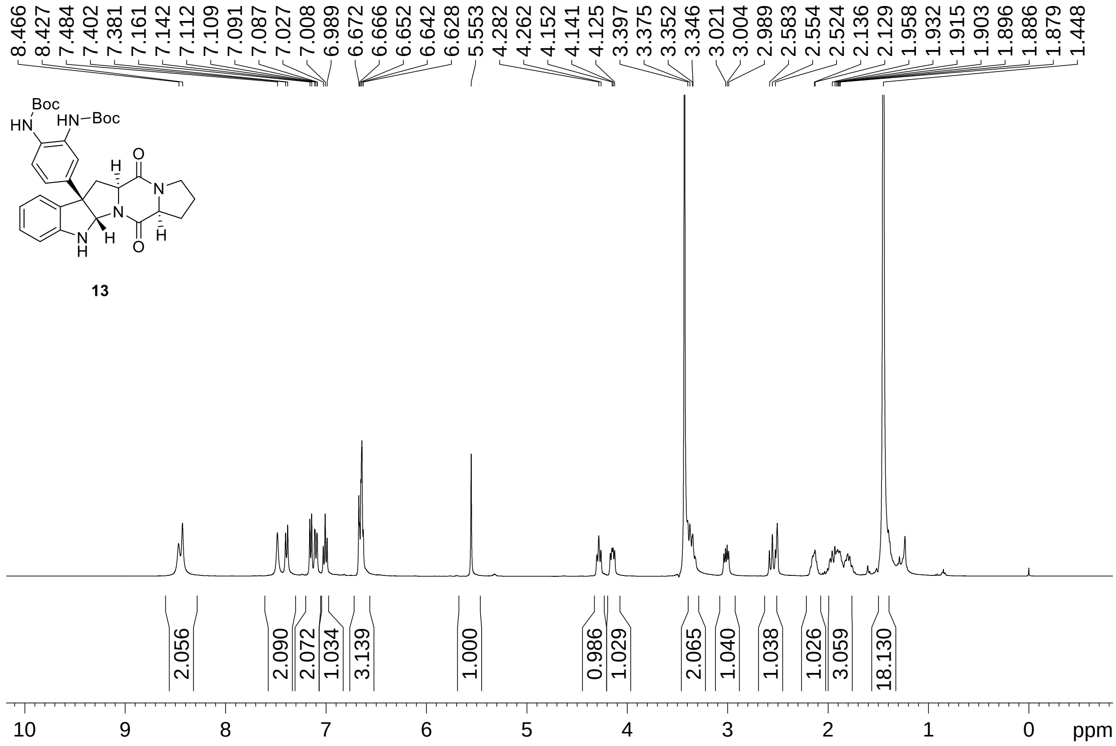

^{13}C NMR spectrum of compound **13** ($(CD_3)_2SO$, 100 MHz)

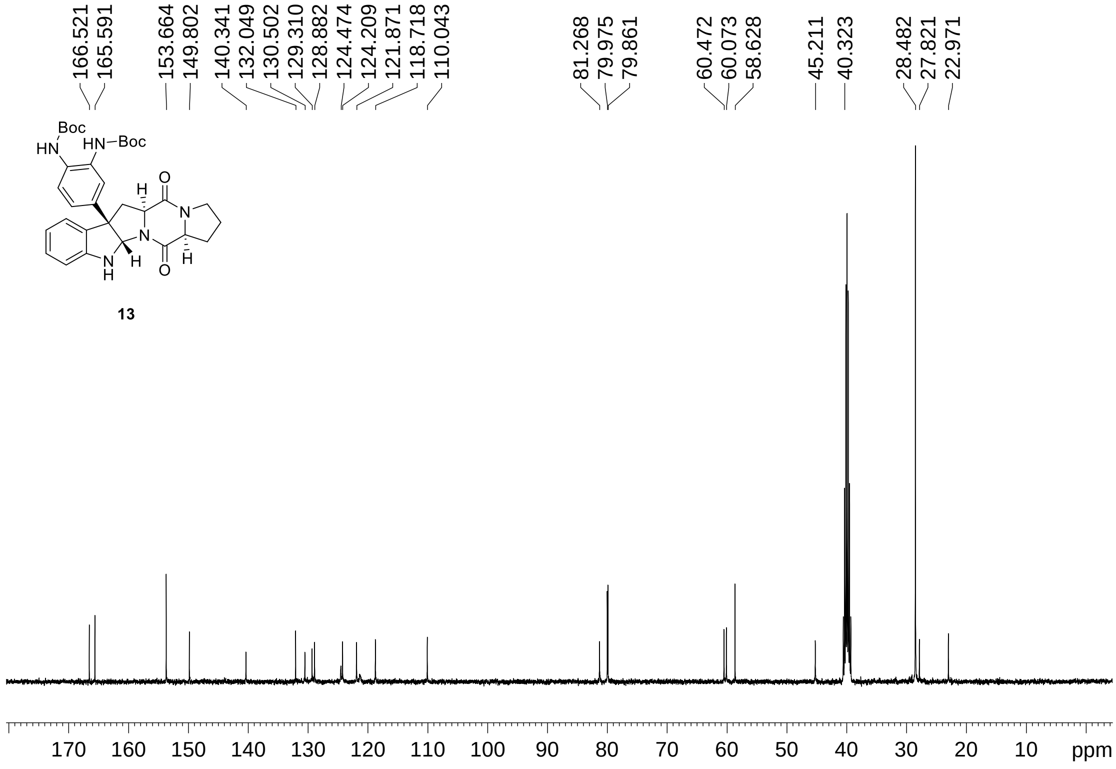

^{1}H NMR spectrum of compound **13′** ($CDCl_3$, 400 MHz)

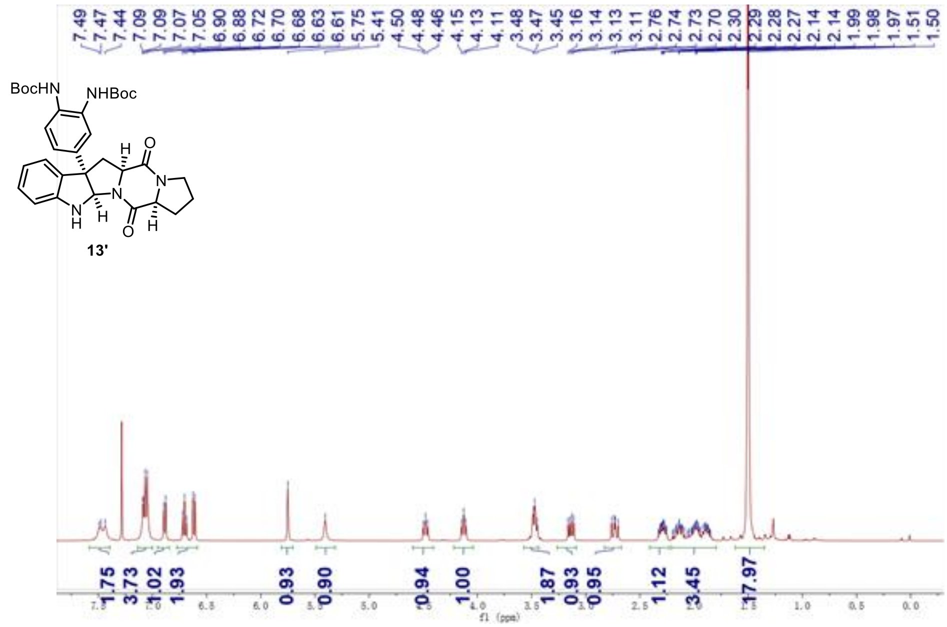

^{13}C NMR spectrum of compound **13′** ($CDCl_3$, 100 MHz)

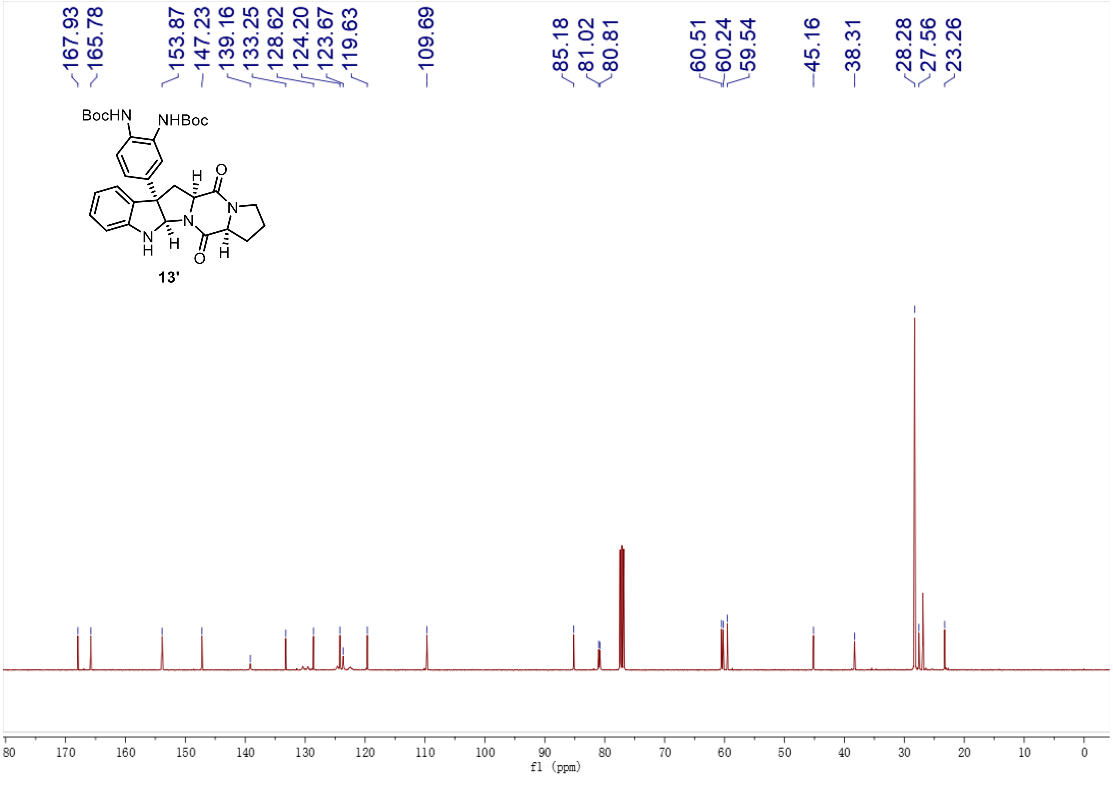

^{1}H NMR spectrum of compound **14** ($CDCl_3$, 400 MHz)

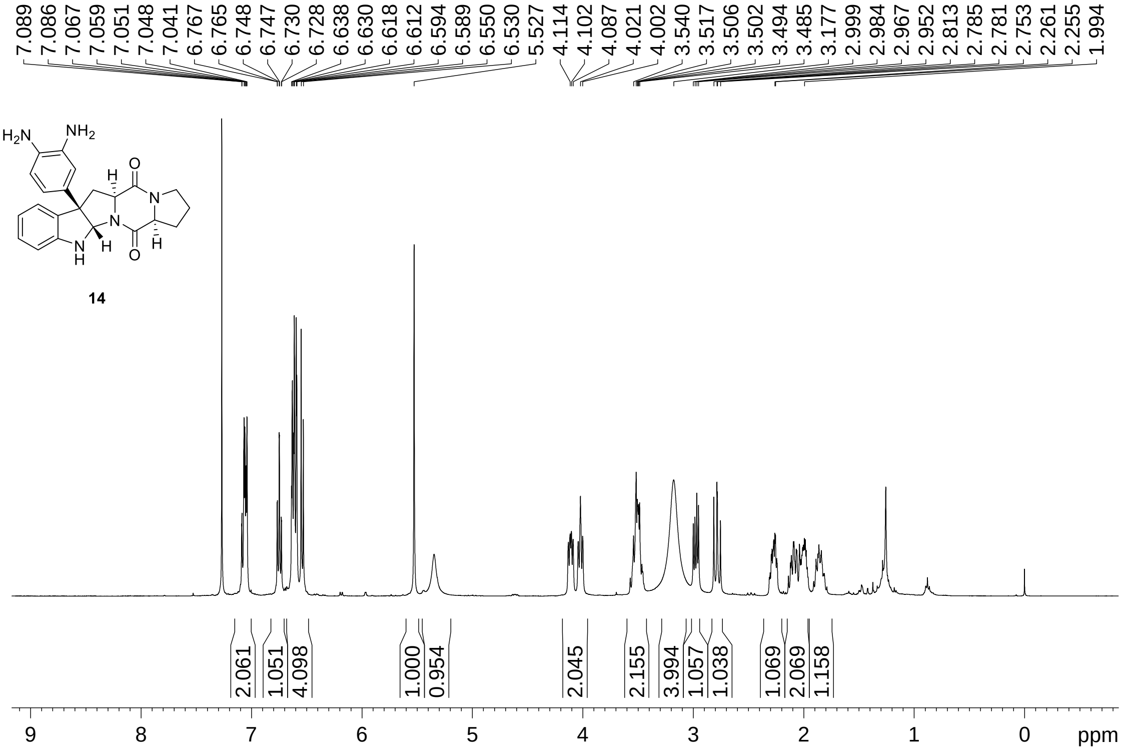

^{13}C NMR spectrum of compound **14** ($CDCl_3$, 100 MHz)

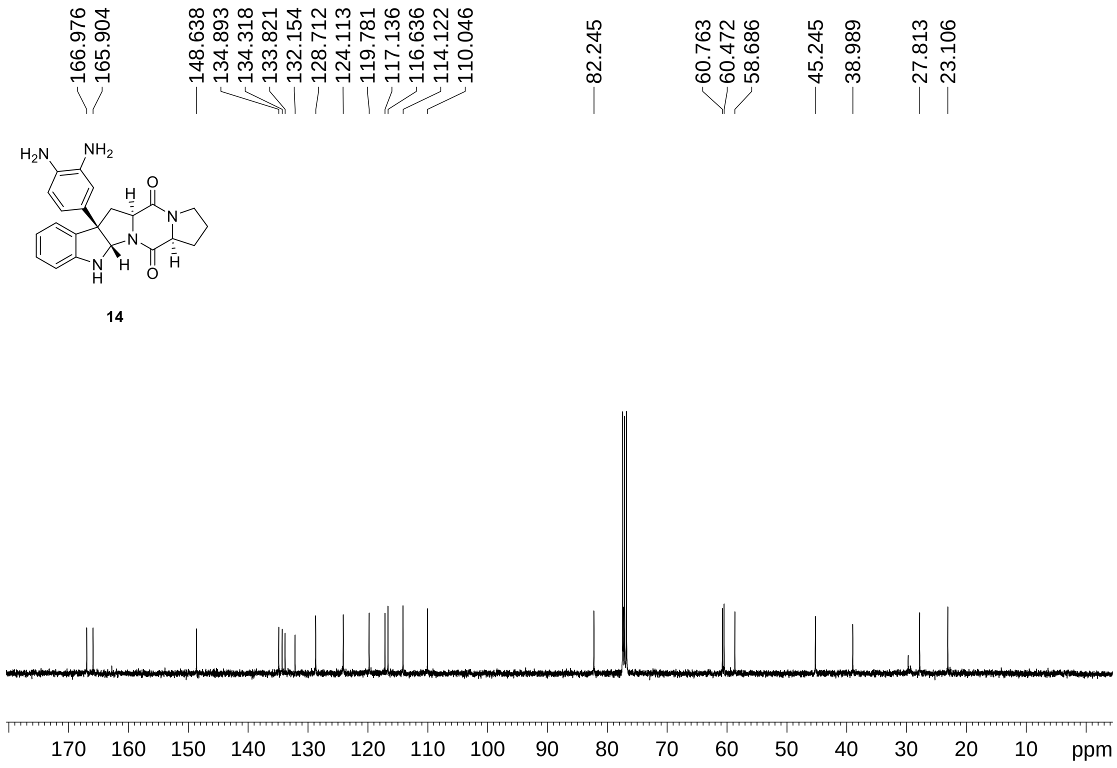

^{1}H NMR spectrum of compound **15** ($(CD_3)_2SO$, 400 MHz)

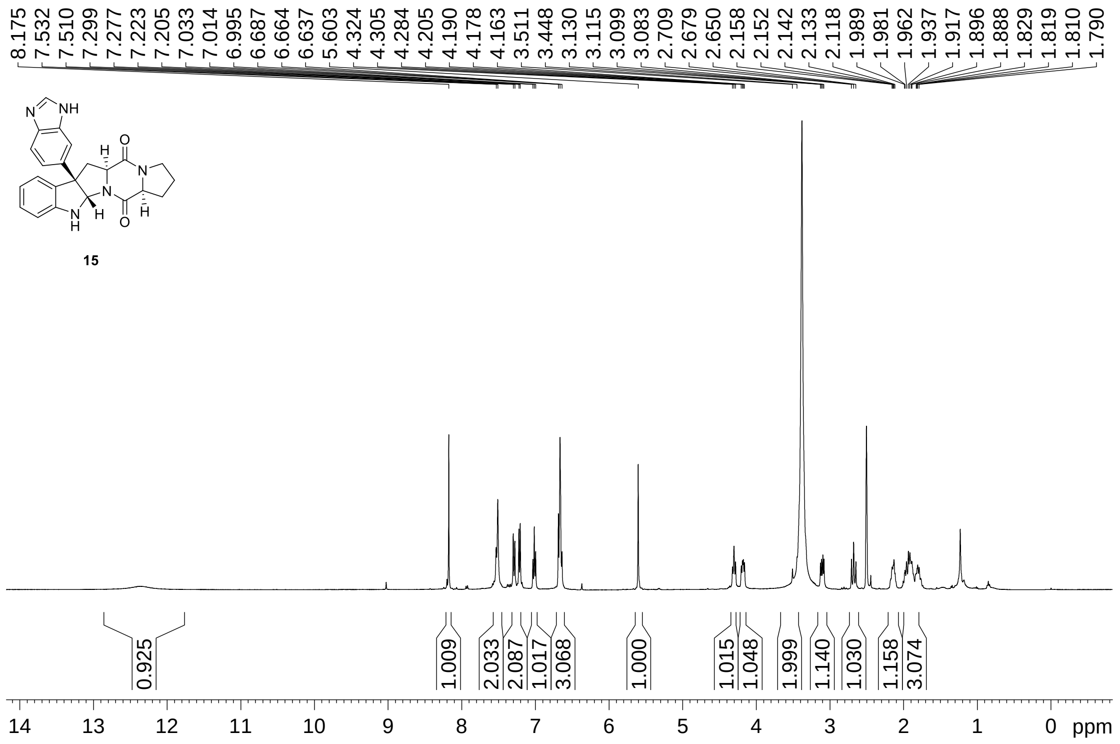

^{13}C NMR spectrum of compound **15** ($(CD_3)_2SO$, 100 MHz)

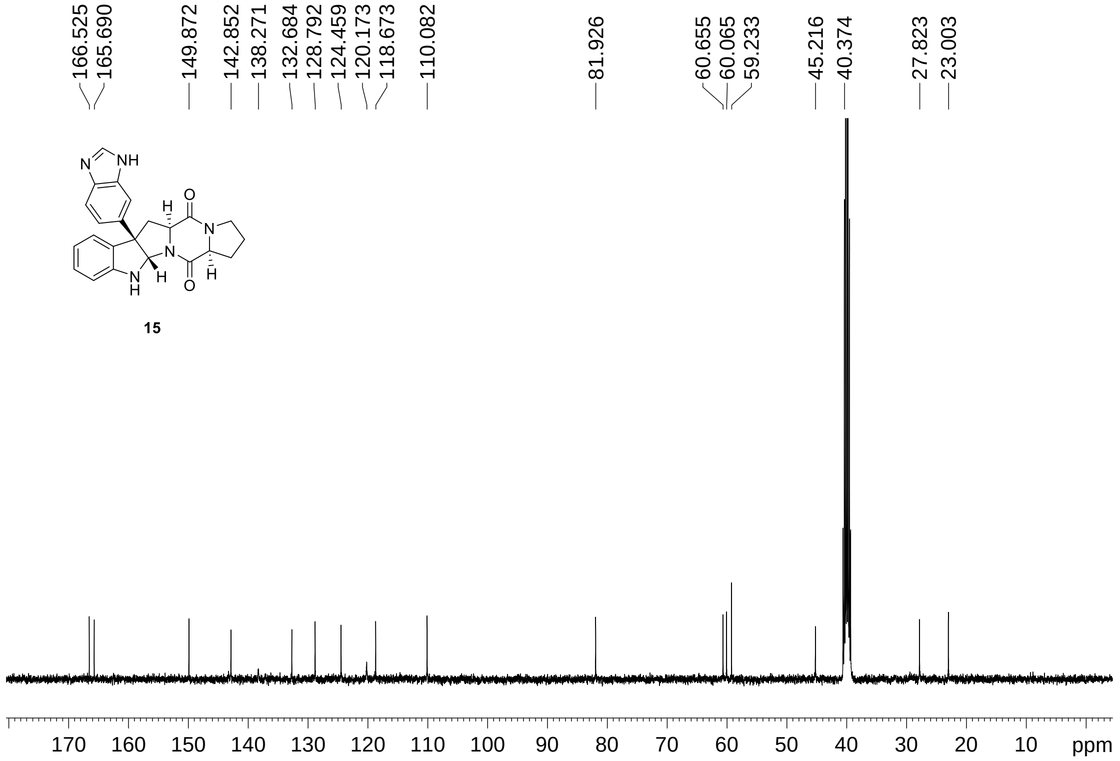

^{13}C-DEPT-135 NMR spectrum of compound **15** ((CD$_3$)$_2$SO, 100 MHz)

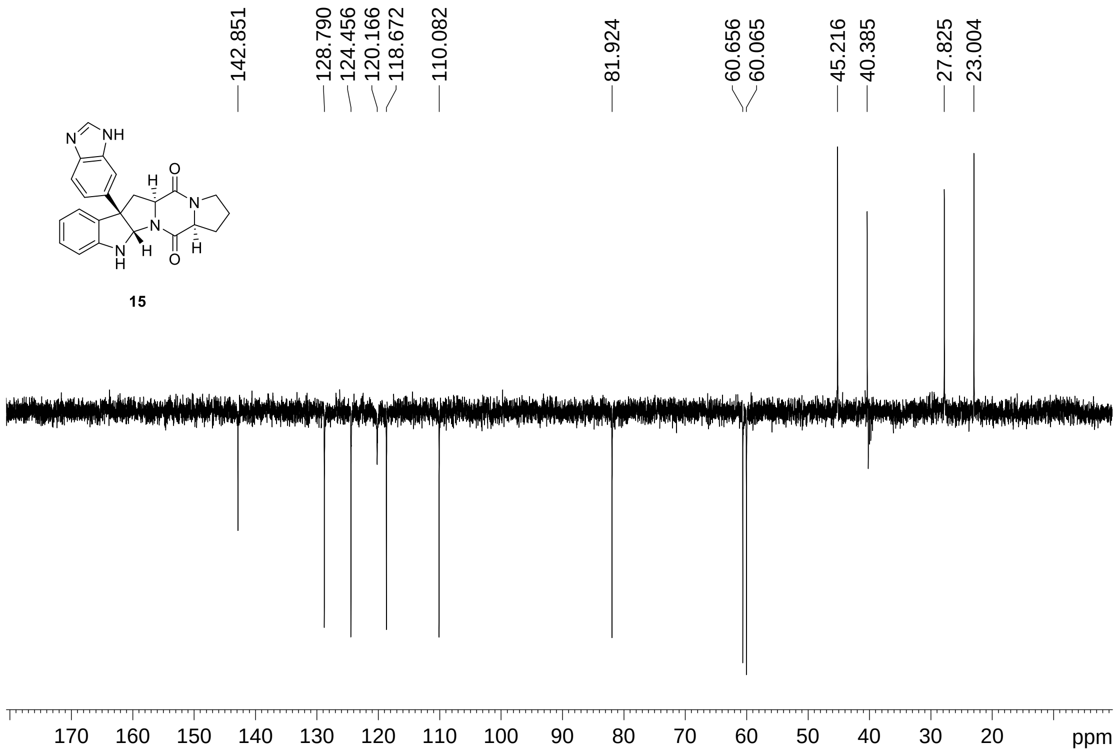

6. HPLC spectra of compounds

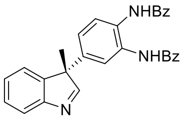

3a

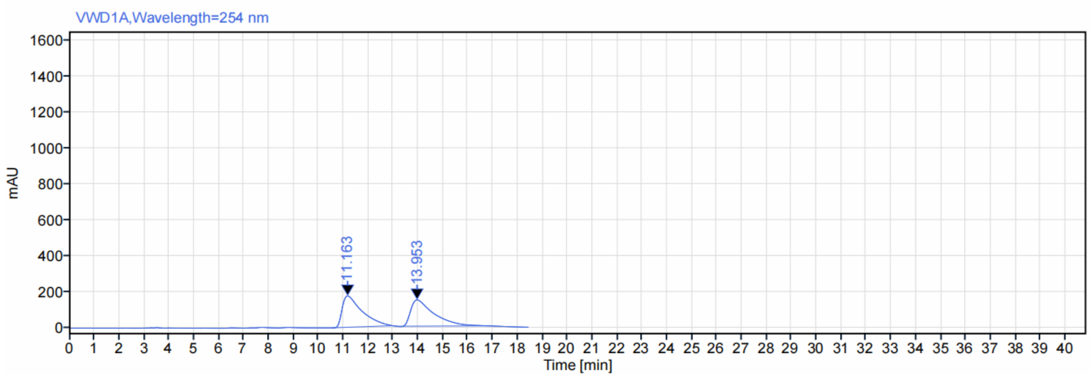

Signal: VWD1A,Wavelength=254 nm

RT [min]	Type	Width [min]	Area	Height	Area%	Name
11.163	MM m	2.44	9695.43	174.69	50.64	
13.953	MM m	3.73	9450.96	146.37	49.36	
		**Sum**	**19146.40**			

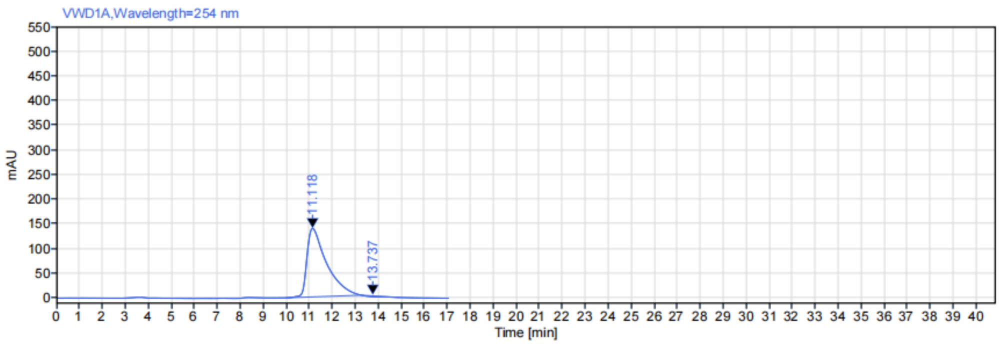

Signal: VWD1A,Wavelength=254 nm

RT [min]	Type	Width [min]	Area	Height	Area%	Name
11.118	MM m	3.28	8243.83	138.81	98.92	
13.737	MM n	1.66	90.04	1.78	1.08	
		**Sum**	**8333.87**			

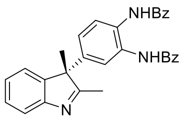

3b

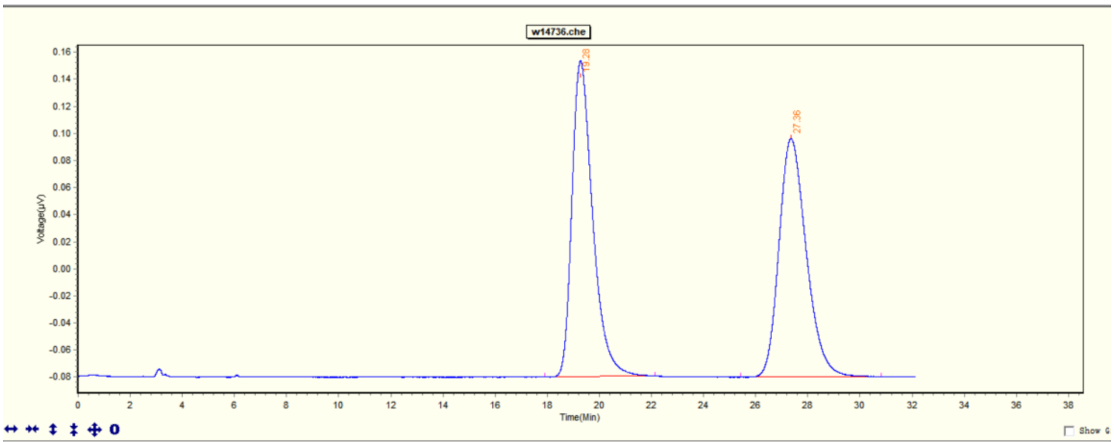

Integration Result | Calculation Result | TimeTable

No.	Retention Time	Peak Area	Peak Height	Peak Area(%)	Peak Width	Peak Type
1	19.28	6451144	116625	50.27%	4.227	BB
2	27.36	6382890	87964	49.73%	5.392	BB
Total		12,834,034	204,589	100.00%		

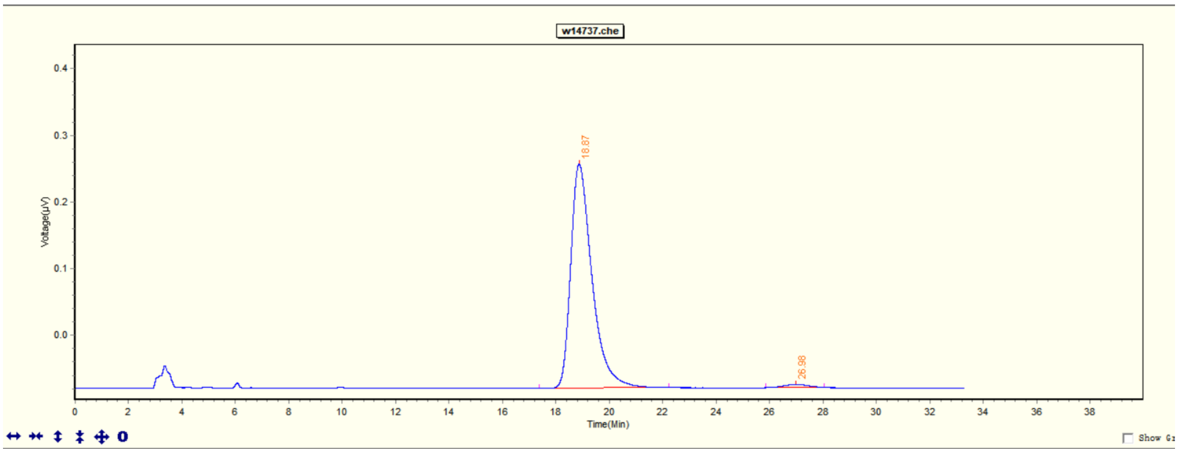

Integration Result | Calculation Result | TimeTable

No.	Retention Time	Peak Area	Peak Height	Peak Area(%)	Peak Width	Peak Type
1	18.87	9288693	167963	98.49%	4.857	BB
2	26.98	142278	2294	1.51%	2.189	BB
Total		9,430,971	170,257	100.00%		

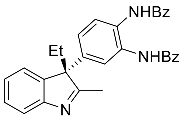

3c

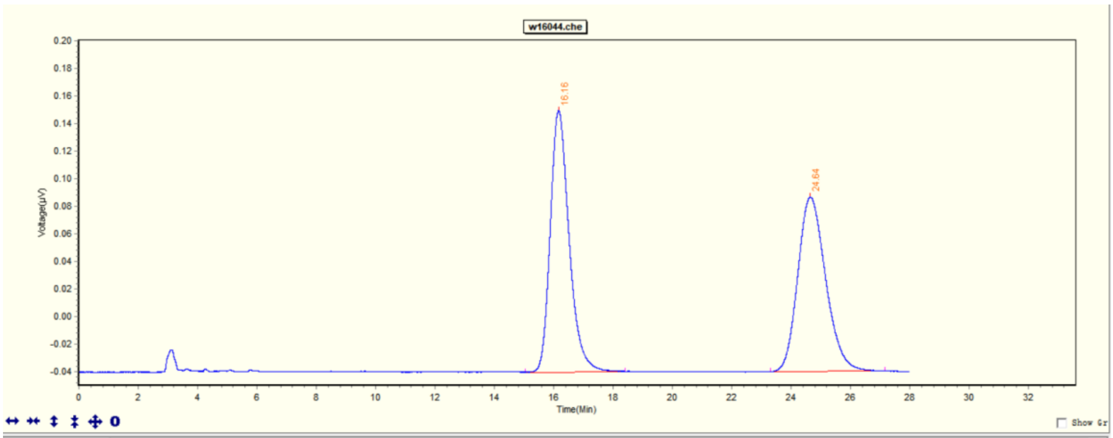

No.	Retention Time	Peak Area	Peak Height	Peak Area(%)	Peak Width	Peak Type
1	16.16	4171018	94647	50.27%	3.362	BB
2	24.64	4125751	63144	49.73%	3.855	BB
Total		8,296,769	157,791	100.00%		

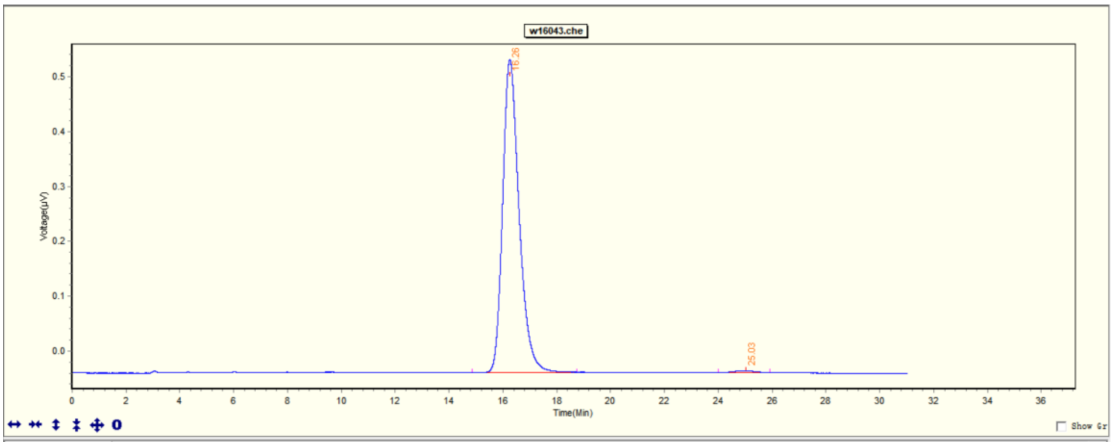

No.	Retention Time	Peak Area	Peak Height	Peak Area(%)	Peak Width	Peak Type
1	16.26	12065142	284846	99.14%	3.888	BB
2	25.03	105133	1857	0.86%	1.912	BB
Total		12,170,275	286,703	100.00%		

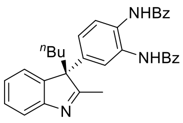

3d

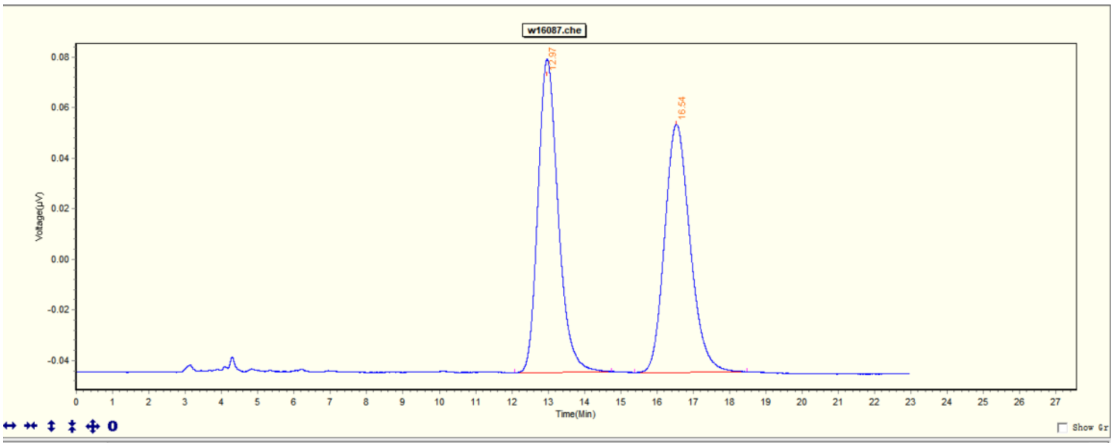

Integration Result | Calculation Result | TimeTable

No.	Retention Time	Peak Area	Peak Height	Peak Area(%)	Peak Width	Peak Type
1	12.97	2398476	61962	50.15%	2.663	BB
2	16.54	2384152	49029	49.85%	3.092	BB
Total		4,782,628	110,991	100.00%		

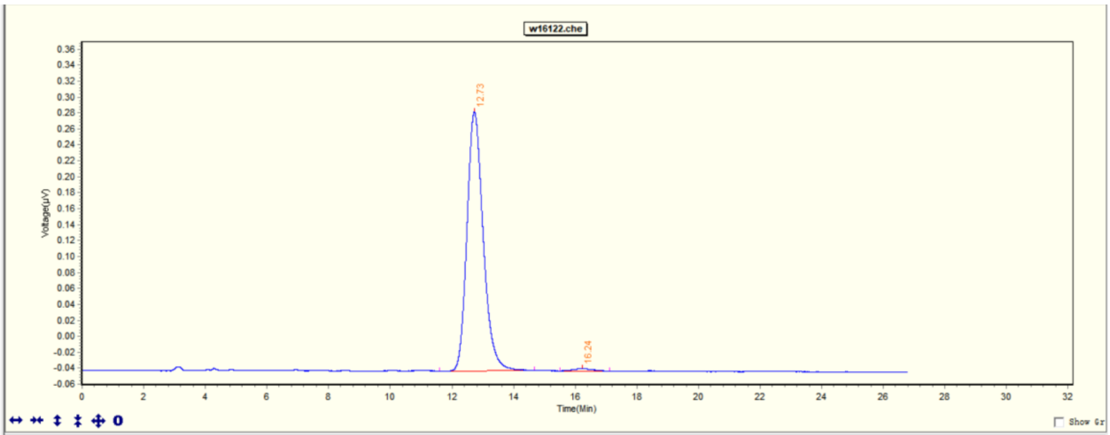

Integration Result | Calculation Result | TimeTable

No.	Retention Time	Peak Area	Peak Height	Peak Area(%)	Peak Width	Peak Type
1	12.73	5931192	162189	98.98%	3.079	BB
2	16.24	61289	1410	1.02%	1.609	BB
Total		5,992,481	163,599	100.00%		

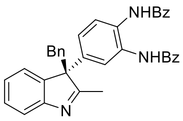

3e

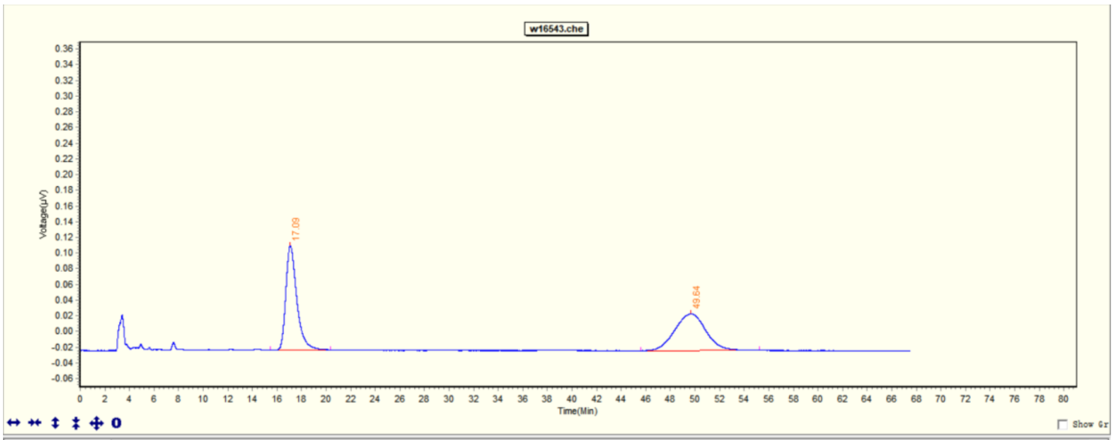

Integration Result | Calculation Result | TimeTable

No.	Retention Time	Peak Area	Peak Height	Peak Area(%)	Peak Width	Peak Type
1	17.09	4130440	66577	50.17%	4.895	BB
2	49.64	4103154	23364	49.83%	9.649	BB
Total		8,233,594	89,941	100.00%		

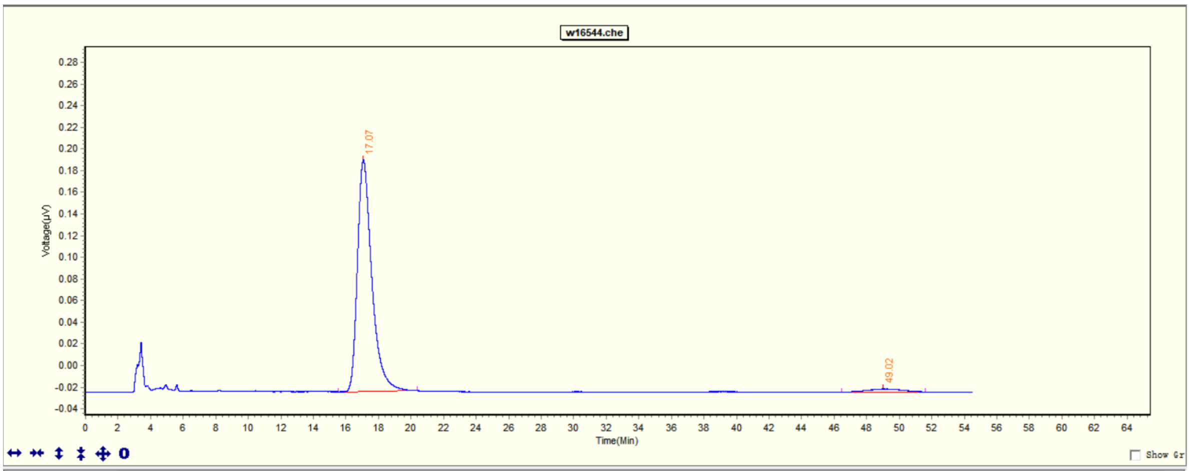

Integration Result | Calculation Result | TimeTable

No.	Retention Time	Peak Area	Peak Height	Peak Area(%)	Peak Width	Peak Type
1	17.07	6554823	106925	96.89%	4.856	BB
2	49.02	210204	1470	3.11%	5.137	BB
Total		6,765,027	108,395	100.00%		

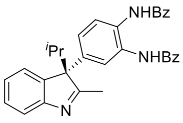

3f

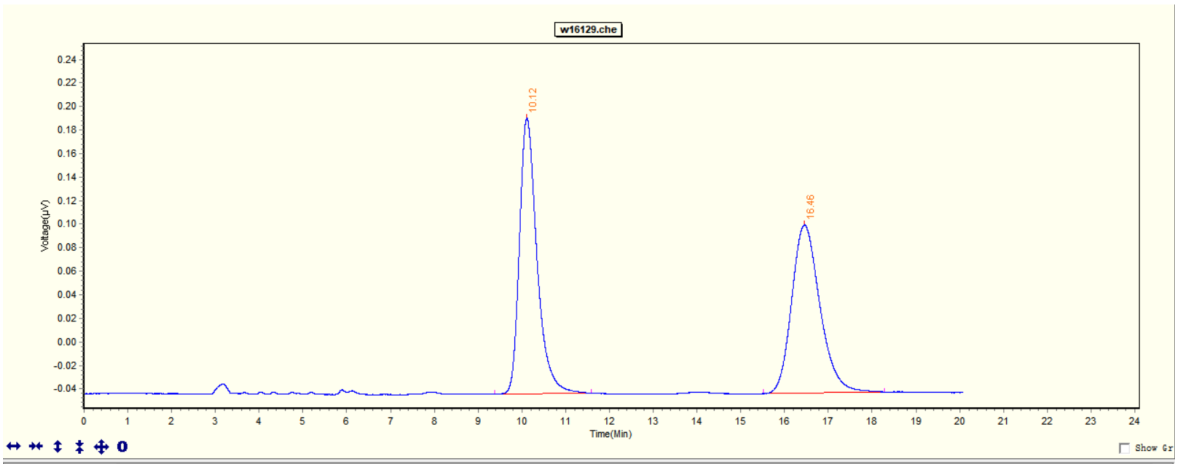

Integration Result | Calculation Result | TimeTable

No.	Retention Time	Peak Area	Peak Height	Peak Area(%)	Peak Width	Peak Type
1	10.12	3253637	117053	50.42%	2.204	BB
2	16.46	3199815	71413	49.58%	2.767	BB
Total		6,453,452	188,466	100.00%		

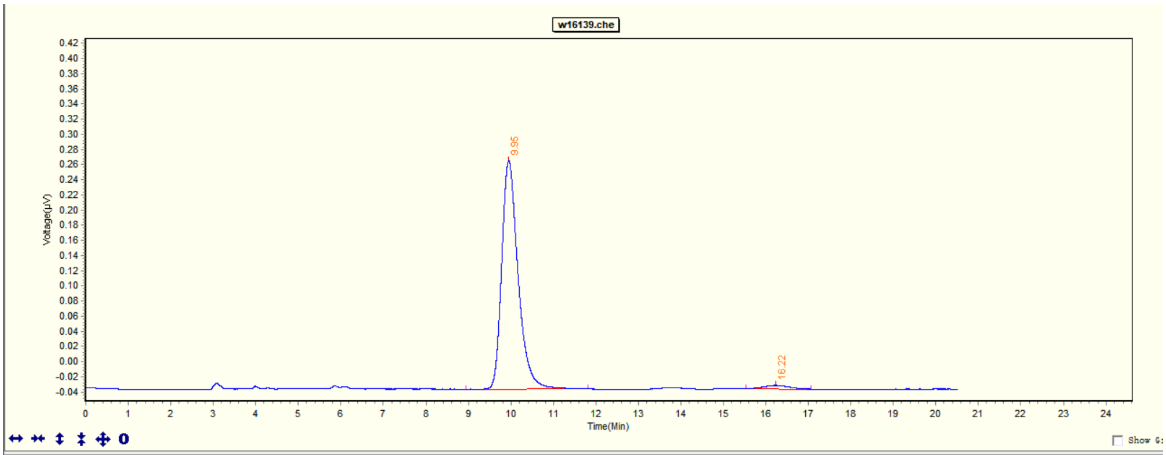

Integration Result | Calculation Result | TimeTable

No.	Retention Time	Peak Area	Peak Height	Peak Area(%)	Peak Width	Peak Type
1	9.95	4021803	150611	97.72%	2.868	BB
2	16.22	93894	2291	2.28%	1.53	BB
Total		4,115,697	152,902	100.00%		

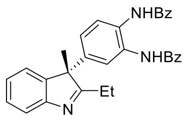

3g

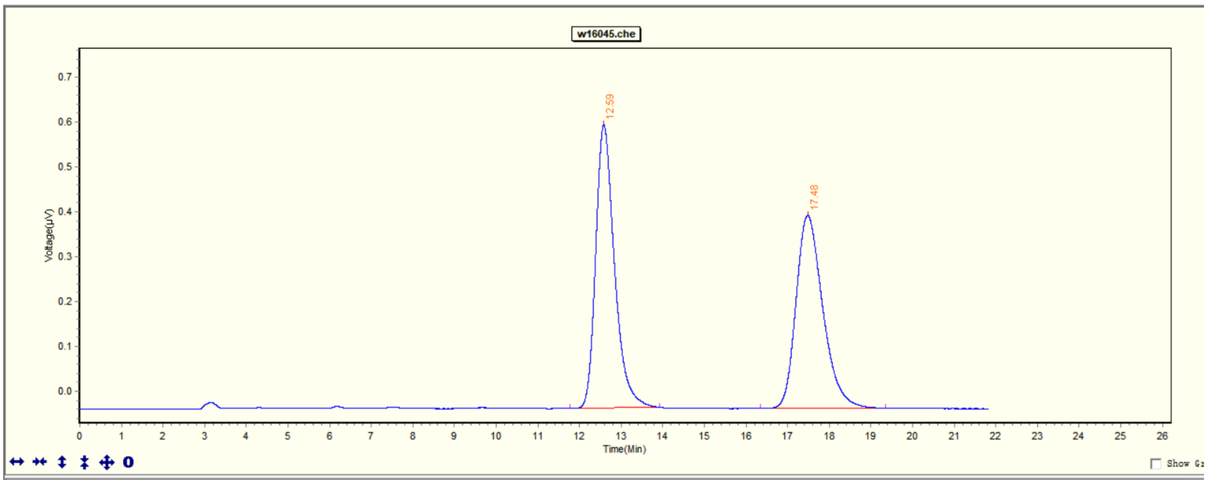

No.	Retention Time	Peak Area	Peak Height	Peak Area(%)	Peak Width	Peak Type
1	12.59	9899208	315545	50.78%	2.153	BB
2	17.48	9594651	215030	49.22%	3.005	BB
Total		19,493,859	530,575	100.00%		

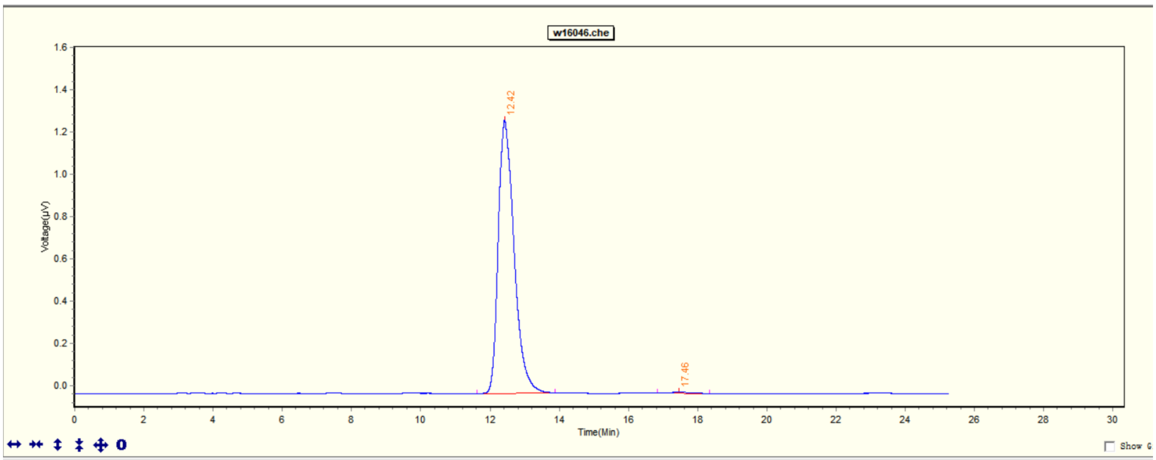

No.	Retention Time	Peak Area	Peak Height	Peak Area(%)	Peak Width	Peak Type
1	12.42	20570292	646942	99.48%	2.257	BB
2	17.46	108200	2722	0.52%	1.505	BB
Total		20,678,492	649,664	100.00%		

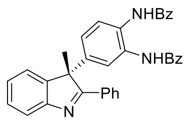

3h

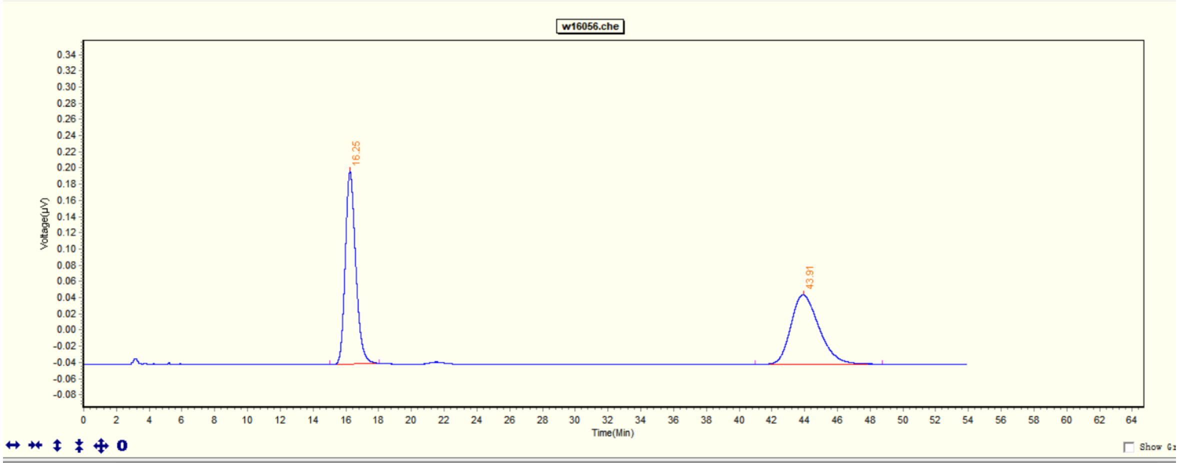

Integration Result | Calculation Result | TimeTable

No.	Retention Time	Peak Area	Peak Height	Peak Area(%)	Peak Width	Peak Type
1	16.25	5262811	118802	50.75%	3.015	BB
2	43.91	5107309	42920	49.25%	7.759	BB
Total		10,370,120	161,722	100.00%		

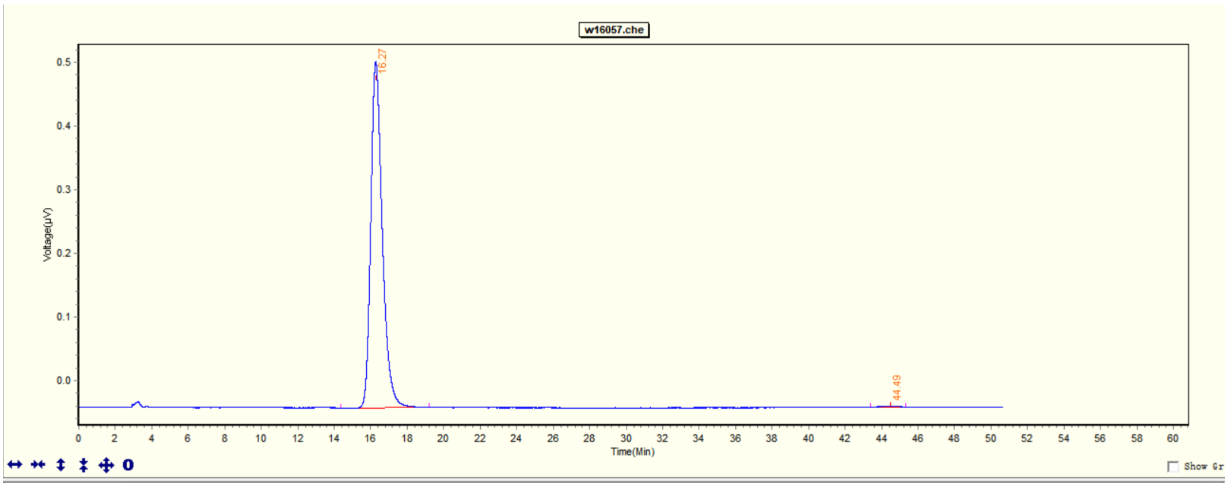

Integration Result | Calculation Result | TimeTable

No.	Retention Time	Peak Area	Peak Height	Peak Area(%)	Peak Width	Peak Type
1	16.27	11911213	271992	99.73%	4.839	BB
2	44.49	32352	468	0.27%	1.925	BB
Total		11,943,565	272,460	100.00%		

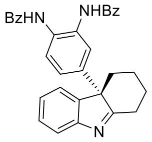

3i

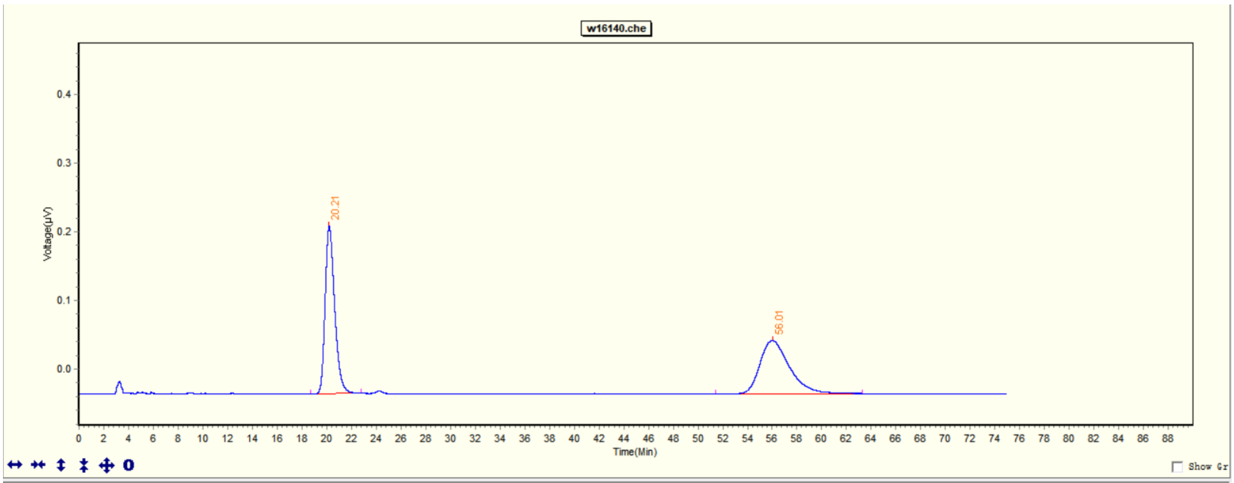

No.	Retention Time	Peak Area	Peak Height	Peak Area(%)	Peak Width	Peak Type
1	20.21	6533882	122078	50.62%	4.038	BB
2	56.01	6372684	38745	49.38%	11.879	BB
Total		12,906,566	160,823	100.00%		

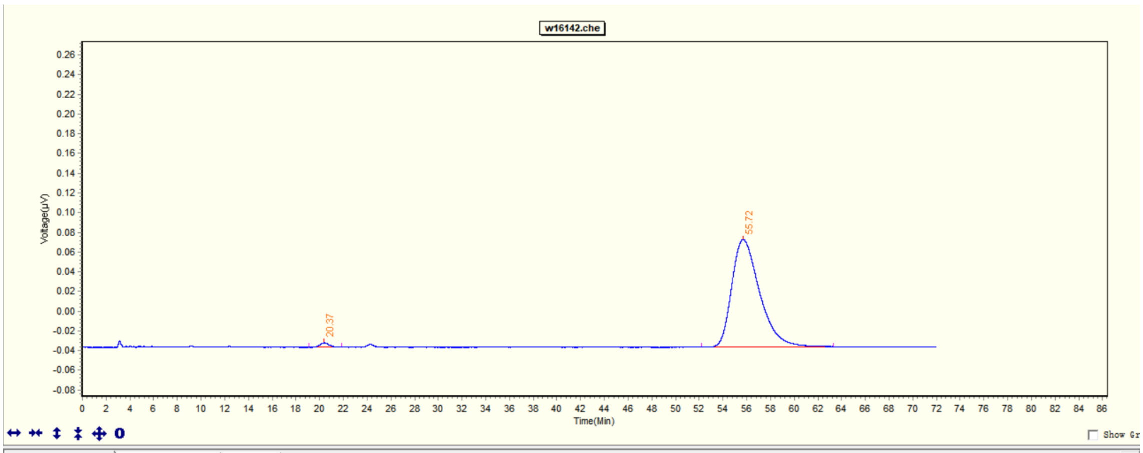

No.	Retention Time	Peak Area	Peak Height	Peak Area(%)	Peak Width	Peak Type
1	20.37	114760	2209	1.28%	2.758	BB
2	55.72	8878610	54521	98.72%	11.11	BB
Total		8,993,370	56,730	100.00%		

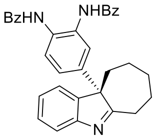

3j

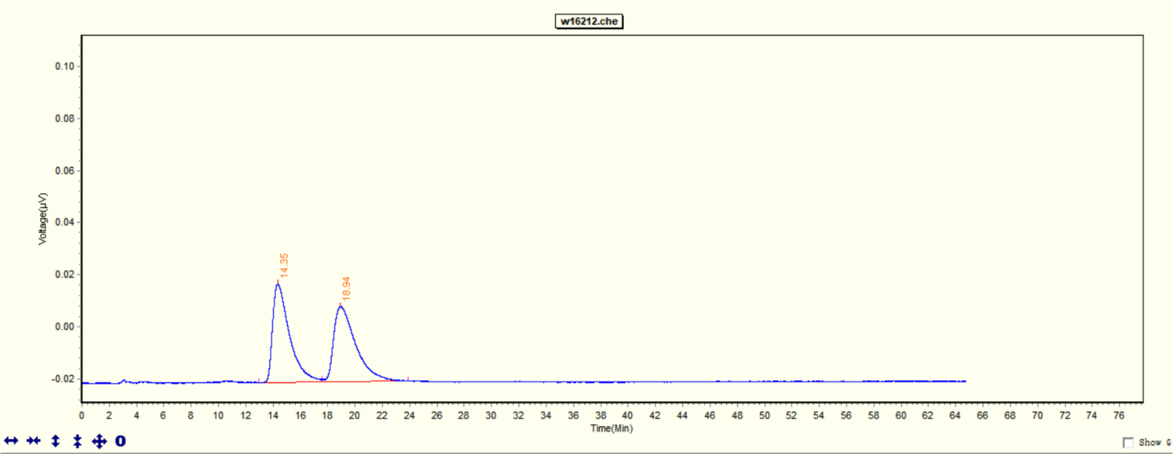

Integration Result | Calculation Result | TimeTable

No.	Retention Time	Peak Area	Peak Height	Peak Area(%)	Peak Width	Peak Type
1	14.35	1614645	18850	50.04%	4.628	BV
2	18.94	1611918	14469	49.96%	6.304	VB
Total		3,226,563	33,319	100.00%		

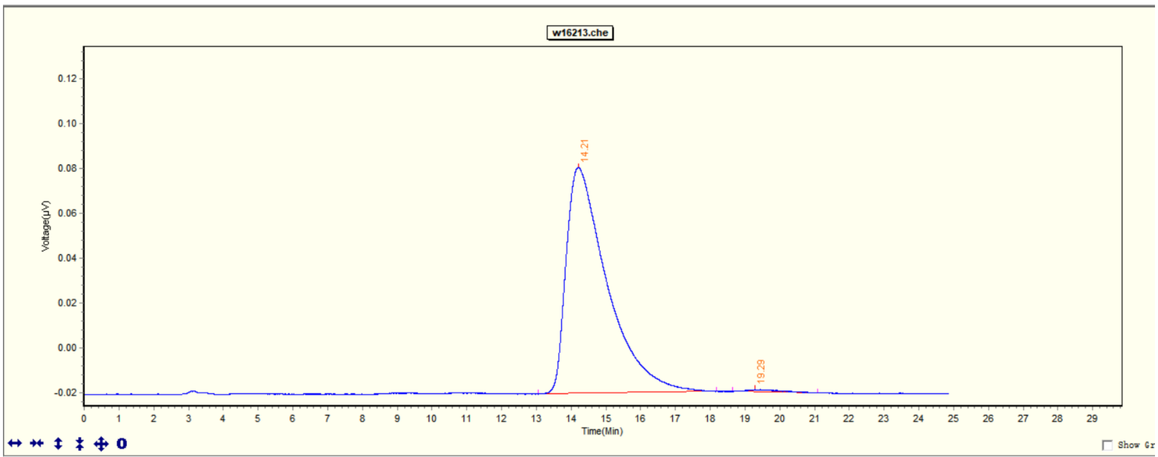

Integration Result | Calculation Result | TimeTable

No.	Retention Time	Peak Area	Peak Height	Peak Area(%)	Peak Width	Peak Type
1	14.21	4114324	50218	99.15%	5.123	BB
2	19.29	35247	485	0.85%	2.446	BB
Total		4,149,571	50,703	100.00%		

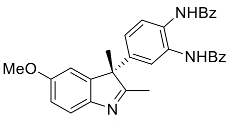

3k

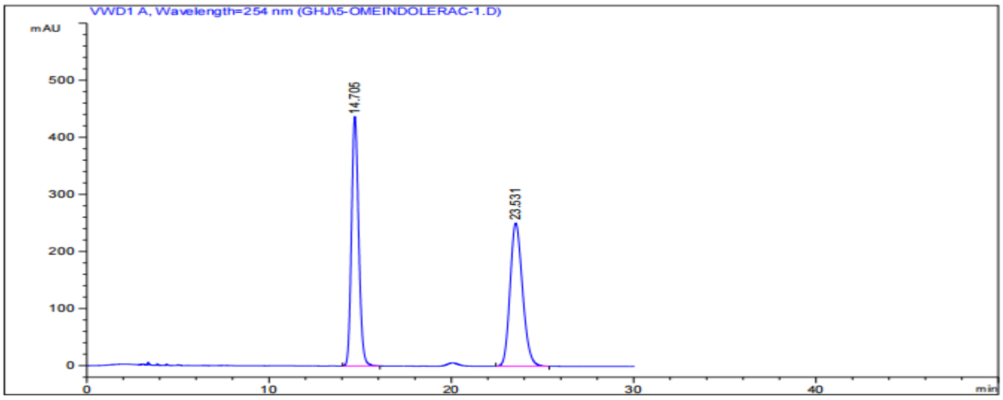

==
Area Percent Report
==

Sorted By : Signal
Multiplier : 1.0000
Dilution : 1.0000
Use Multiplier & Dilution Factor with ISTDs

Signal 1: VWD1 A, Wavelength=254 nm

Peak #	RetTime [min]	Type	Width [min]	Area mAU *s	Height [mAU]	Area %
1	14.705	BB	0.4219	1.19300e4	437.02902	50.0285
2	23.531	BB	0.7378	1.19164e4	250.78174	49.9715

Totals : 2.38464e4 687.81076

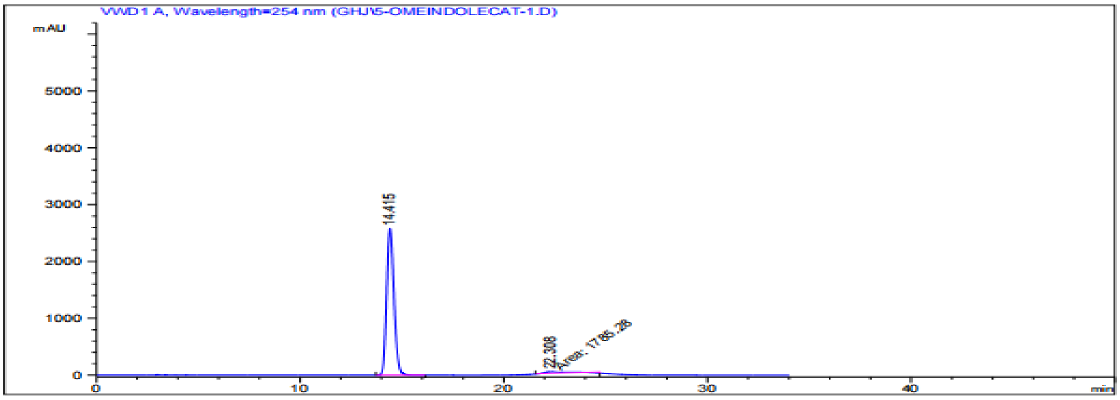

==
Area Percent Report
==

Sorted By : Signal
Multiplier : 1.0000
Dilution : 1.0000
Use Multiplier & Dilution Factor with ISTDs

Signal 1: VWD1 A, Wavelength=254 nm

Peak #	RetTime [min]	Type	Width [min]	Area mAU *s	Height [mAU]	Area %
1	14.415	VB	0.3900	6.47566e4	2583.21753	97.3171
2	22.308	MM	1.0655	1785.27722	27.92521	2.6829

Totals : 6.65418e4 2611.14274

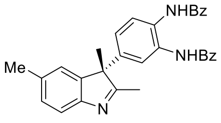

3l

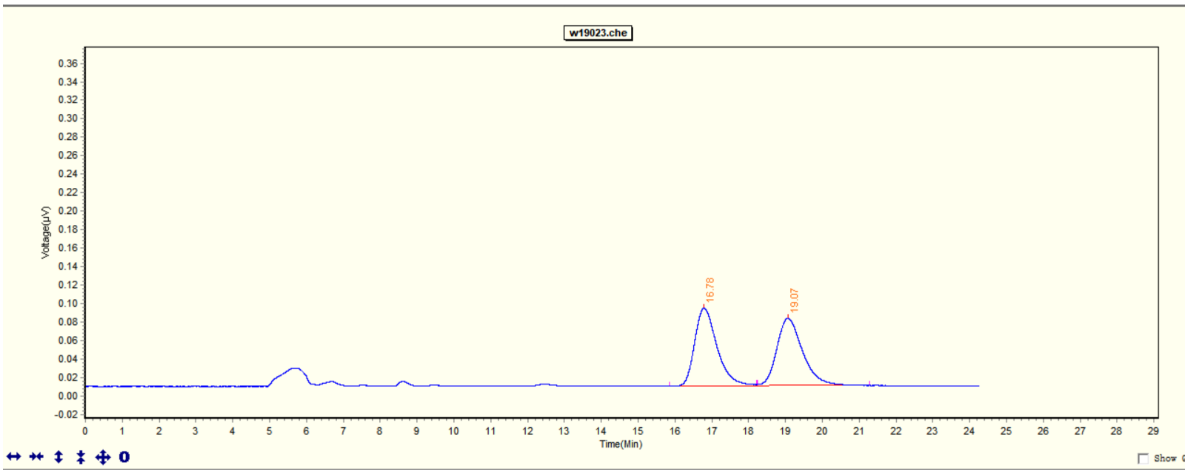

No.	Retention Time	Peak Area	Peak Height	Peak Area(%)	Peak Width	Peak Type
1	16.78	1776401	42166	49.95%	2.378	BV
2	19.07	1780239	36484	50.05%	3.054	VB
Total		3,556,640	78,650	100.00%		

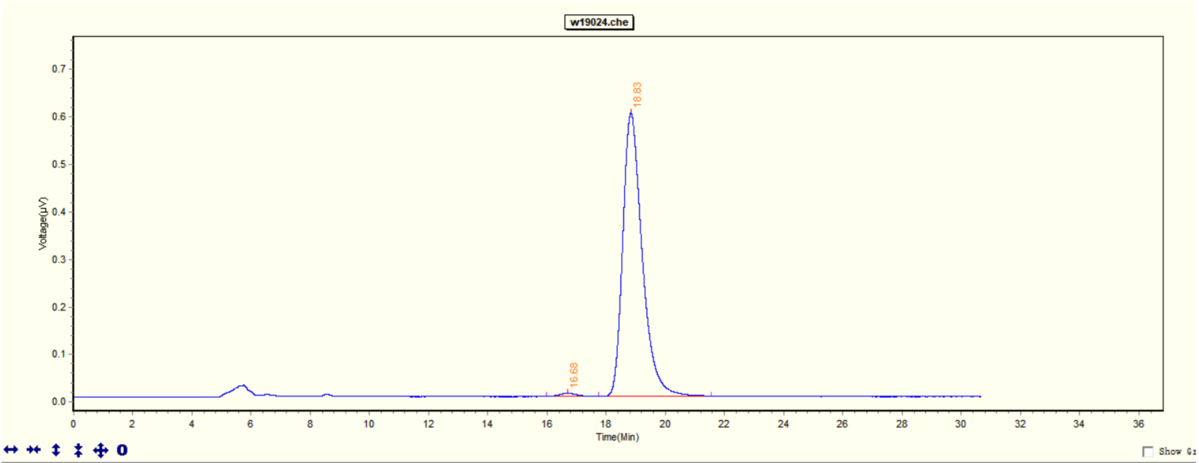

No.	Retention Time	Peak Area	Peak Height	Peak Area(%)	Peak Width	Peak Type
1	16.68	128832	3194	0.94%	1.763	BV
2	18.83	13568304	298094	99.06%	3.813	VB
Total		13,697,136	301,288	100.00%		

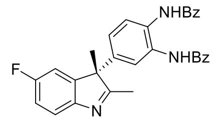

3m

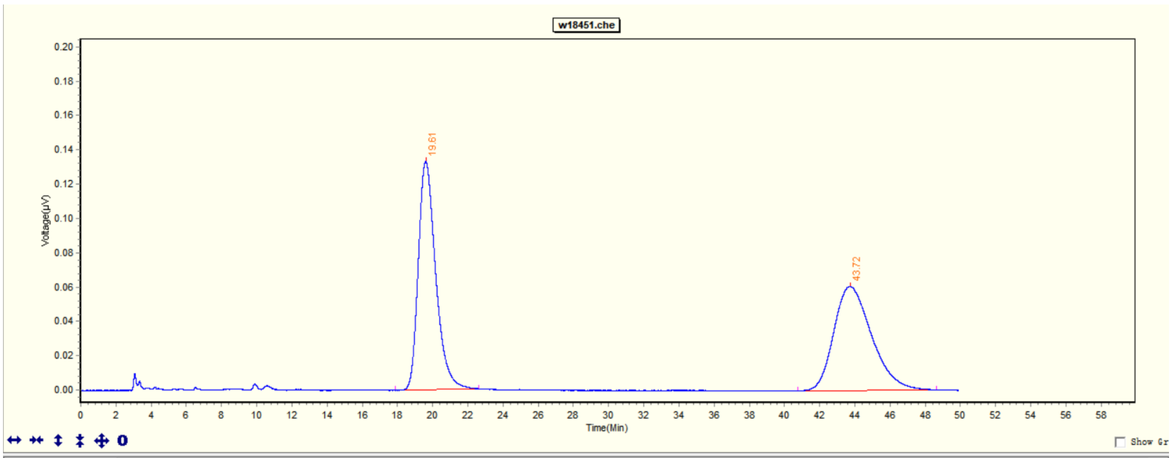

Integration Result | Calculation Result | TimeTable

No.	Retention Time	Peak Area	Peak Height	Peak Area(%)	Peak Width	Peak Type
1	19.61	4560862	66508	50.27%	4.74	BB
2	43.72	4512416	30312	49.73%	7.883	BB
Total		9,073,278	96,820	100.00%		

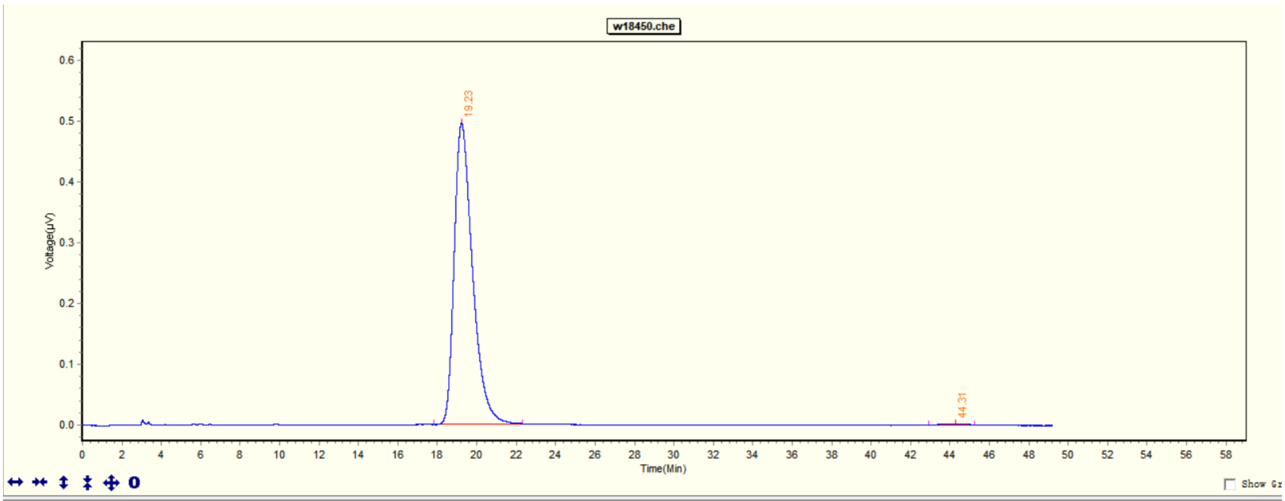

Integration Result | Calculation Result | TimeTable

No.	Retention Time	Peak Area	Peak Height	Peak Area(%)	Peak Width	Peak Type
1	19.23	15949953	247374	99.68%	4.493	BB
2	44.31	51460	625	0.32%	2.323	BB
Total		16,001,413	247,999	100.00%		

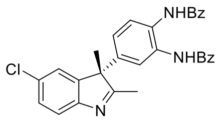

3n

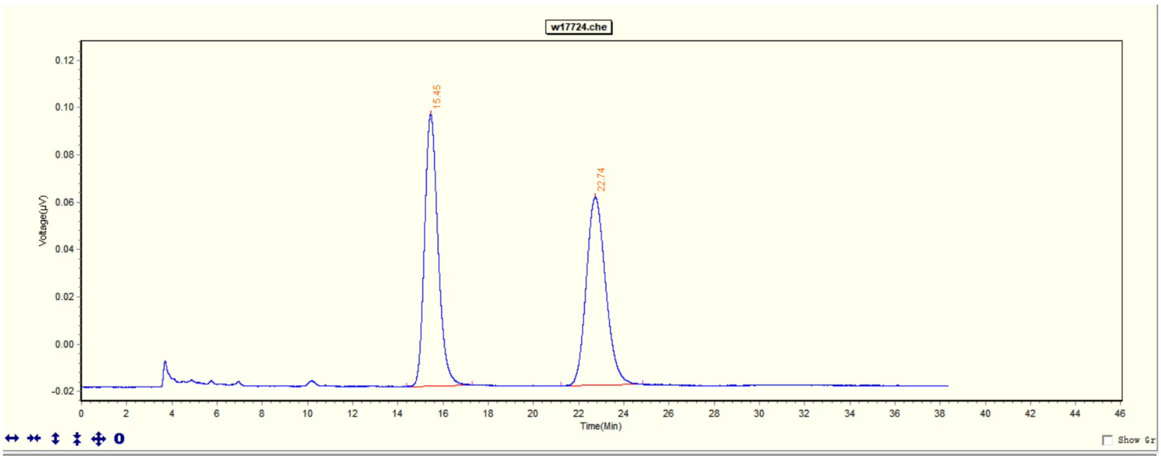

No.	Retention Time	Peak Area	Peak Height	Peak Area(%)	Peak Width	Peak Type
1	15.45	2366785	57387	49.97%	2.901	BB
2	22.74	2369537	39747	50.03%	3.617	BB
Total		4,736,322	97,134	100.00%		

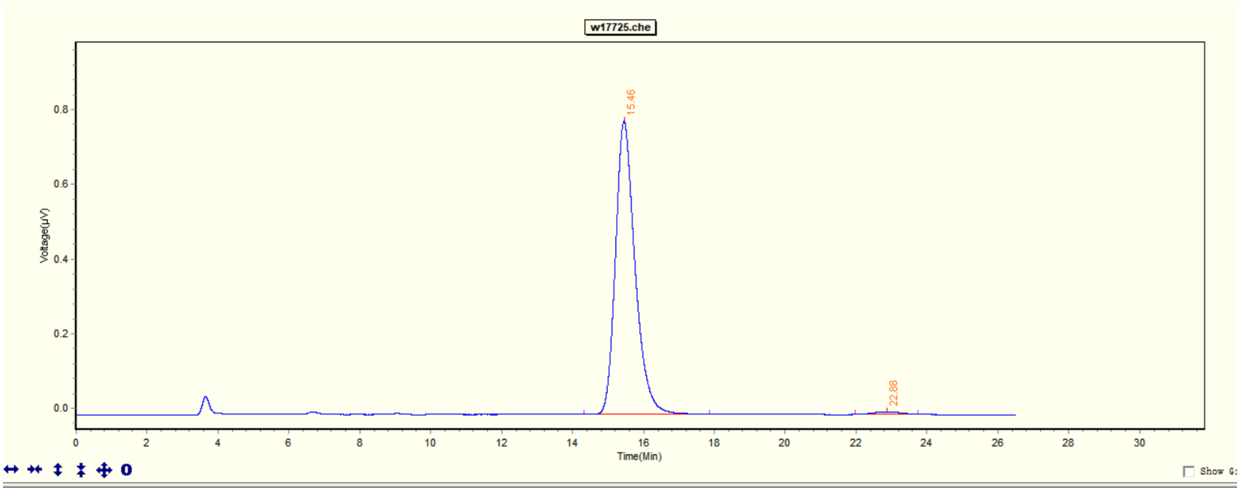

No.	Retention Time	Peak Area	Peak Height	Peak Area(%)	Peak Width	Peak Type
1	15.46	15035337	392823	98.79%	3.541	BB
2	22.88	184731	3535	1.21%	1.77	BB
Total		15,220,068	396,358	100.00%		

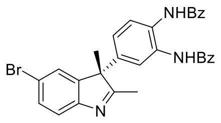

3o

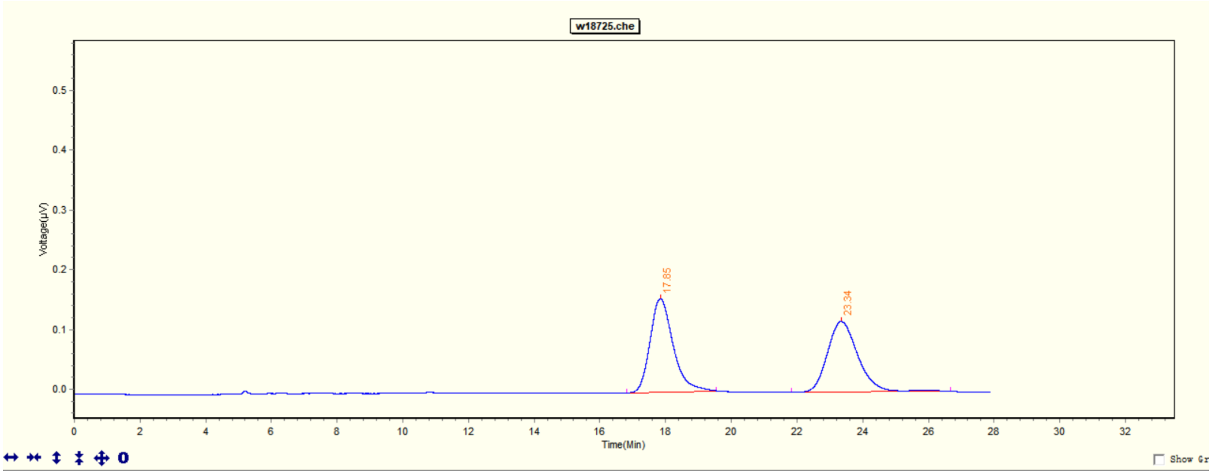

No.	Retention Time	Peak Area	Peak Height	Peak Area(%)	Peak Width	Peak Type
1	17.85	3787708	77826	49.89%	2.722	BB
2	23.34	3804520	58720	50.11%	4.838	BB
Total		7,592,228	136,546	100.00%		

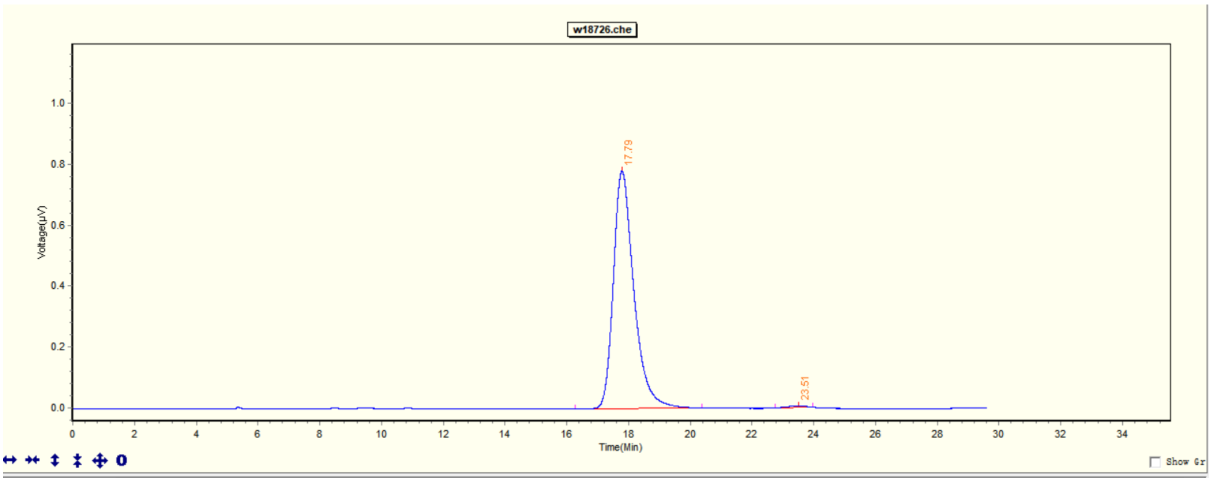

No.	Retention Time	Peak Area	Peak Height	Peak Area(%)	Peak Width	Peak Type
1	17.79	17958856	389796	99.43%	4.107	BB
2	23.51	102773	2411	0.57%	1.217	BB
Total		18,061,629	392,207	100.00%		

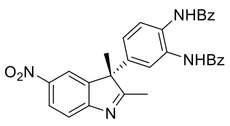

3p

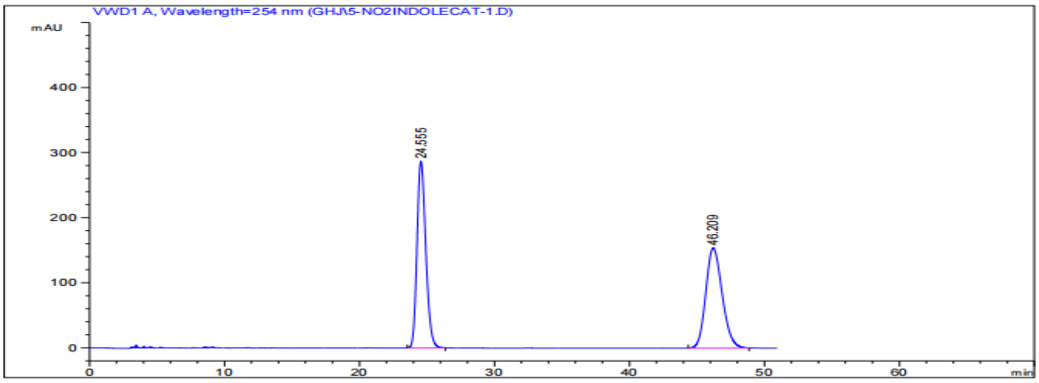

Area Percent Report

Sorted By : Signal
Multiplier : 1.0000
Dilution : 1.0000
Use Multiplier & Dilution Factor with ISTDs

Signal 1: VWD1 A, Wavelength=254 nm

Peak #	RetTime [min]	Type	Width [min]	Area mAU *s	Height [mAU]	Area %
1	24.555	BB	0.7016	1.29768e4	286.56149	50.0212
2	46.209	BB	1.3069	1.29658e4	153.78328	49.9788
Totals :				2.59427e4	440.34477	

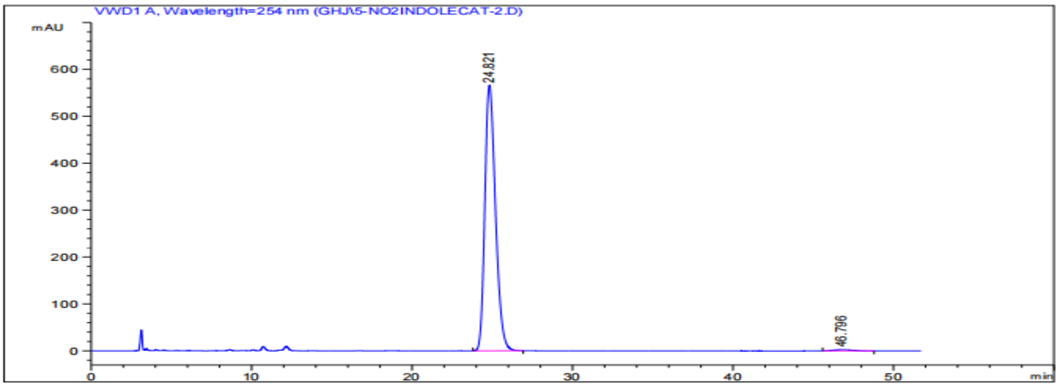

Area Percent Report

Sorted By : Signal
Multiplier : 1.0000
Dilution : 1.0000
Use Multiplier & Dilution Factor with ISTDs

Signal 1: VWD1 A, Wavelength=254 nm

Peak #	RetTime [min]	Type	Width [min]	Area mAU *s	Height [mAU]	Area %
1	24.821	BB	0.7117	2.60477e4	565.96448	99.1999
2	46.796	BB	0.9928	210.09012	2.60635	0.8001
Totals :				2.62578e4	568.57082	

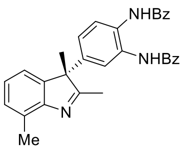

3q

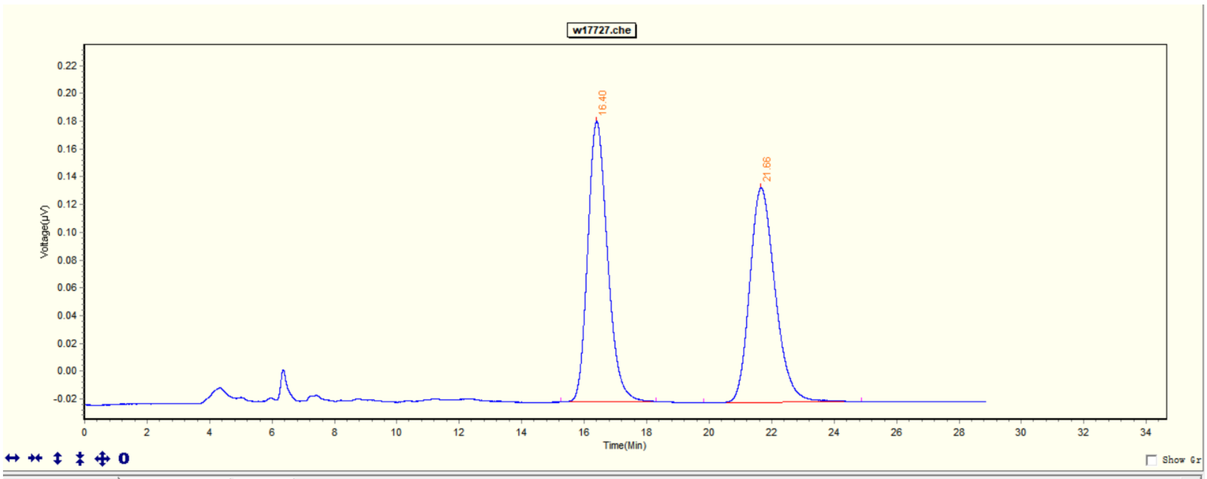

No.	Retention Time	Peak Area	Peak Height	Peak Area(%)	Peak Width	Peak Type
1	16.40	4438145	100949	50.31%	3.049	BB
2	21.66	4383426	77226	49.69%	5.053	BB
Total		8,821,571	178,175	100.00%		

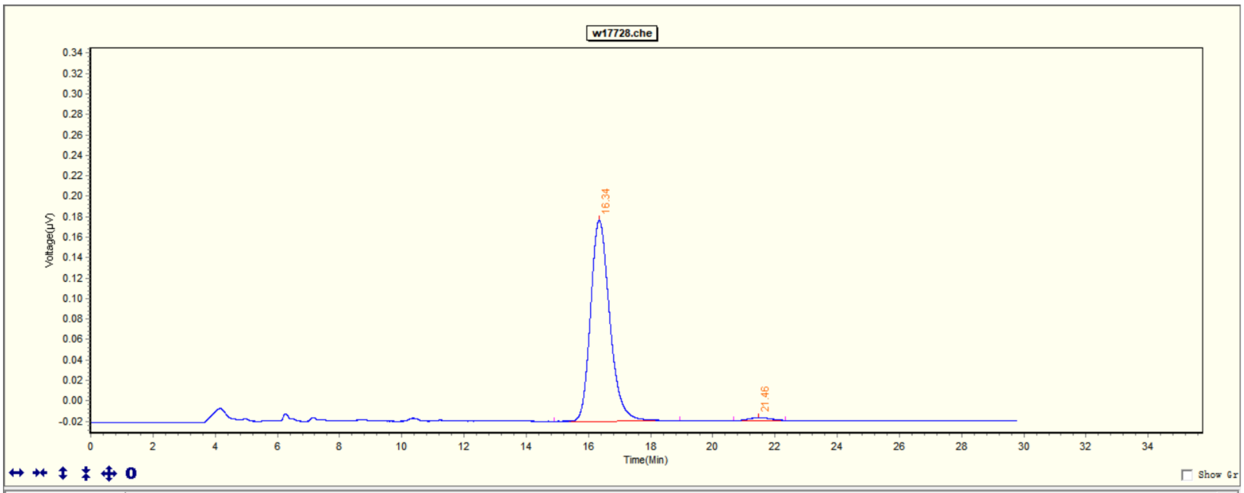

No.	Retention Time	Peak Area	Peak Height	Peak Area(%)	Peak Width	Peak Type
1	16.34	4193250	98138	98.34%	4.041	BB
2	21.46	70571	1448	1.66%	1.665	BB
Total		4,263,821	99,586	100.00%		

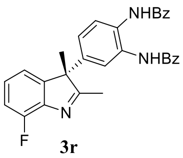

3r

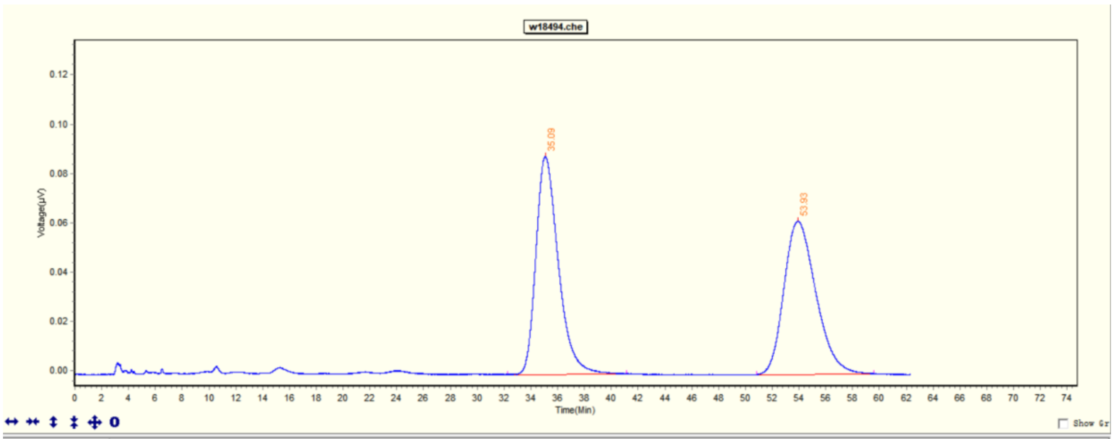

Integration Result | Calculation Result | TimeTable

No.	Retention Time	Peak Area	Peak Height	Peak Area(%)	Peak Width	Peak Type
1	35.09	5124871	44089	49.95%	8.871	BB
2	53.93	5135278	31015	50.05%	8.742	BB
Total		10,260,149	75,104	100.00%		

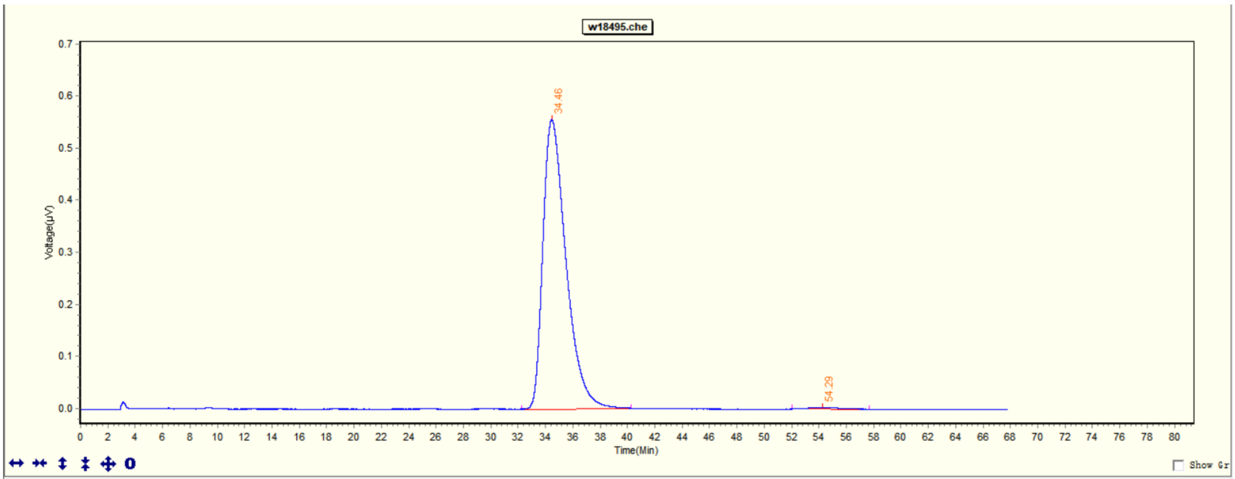

Integration Result | Calculation Result | TimeTable

No.	Retention Time	Peak Area	Peak Height	Peak Area(%)	Peak Width	Peak Type
1	34.46	32553234	277892	99.23%	8.01	BB
2	54.29	252493	1665	0.77%	5.642	BB
Total		32,805,727	279,557	100.00%		

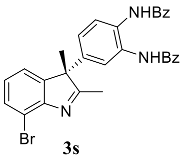

3s

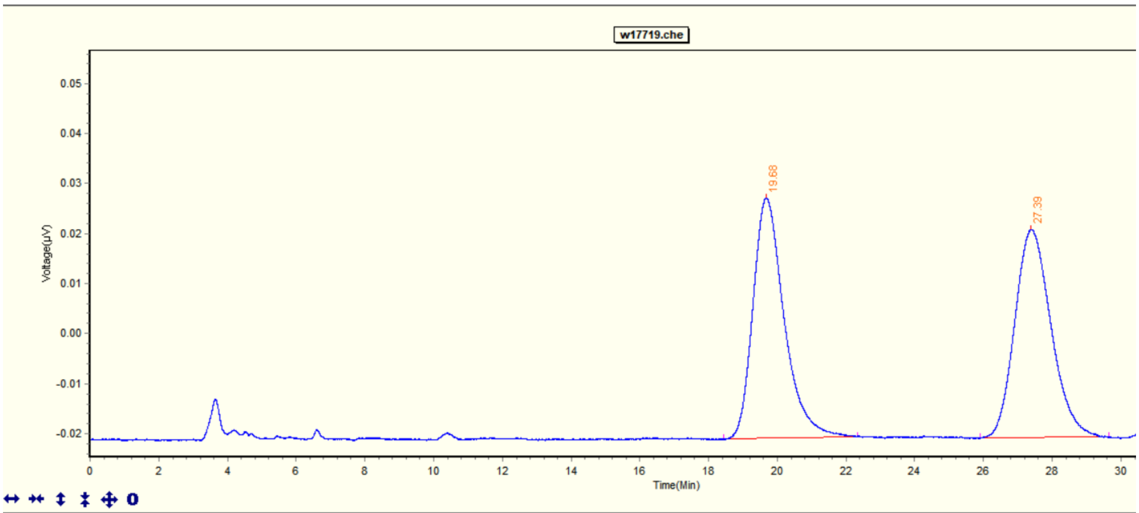

No.	Retention Time	Peak Area	Peak Height	Peak Area(%)	Peak Width	Peak Type
1	19.68	1512119	23967	49.81%	3.894	BB
2	27.39	1523408	20792	50.19%	3.747	BB
Total		3,035,527	44,759	100.00%		

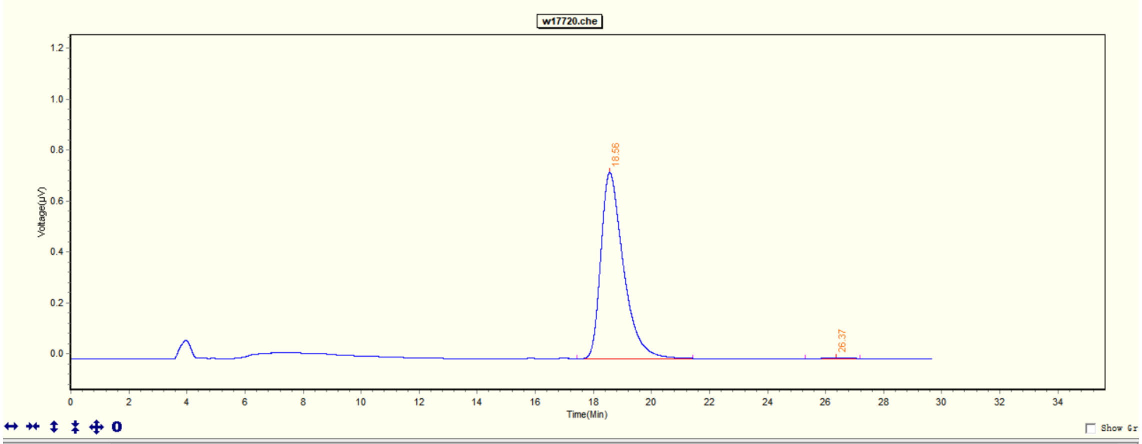

No.	Retention Time	Peak Area	Peak Height	Peak Area(%)	Peak Width	Peak Type
1	18.56	19680736	365793	99.38%	3.992	BB
2	26.37	123068	2283	0.62%	1.889	BB
Total		19,803,804	368,076	100.00%		

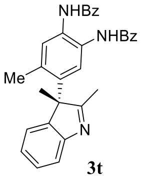

3t

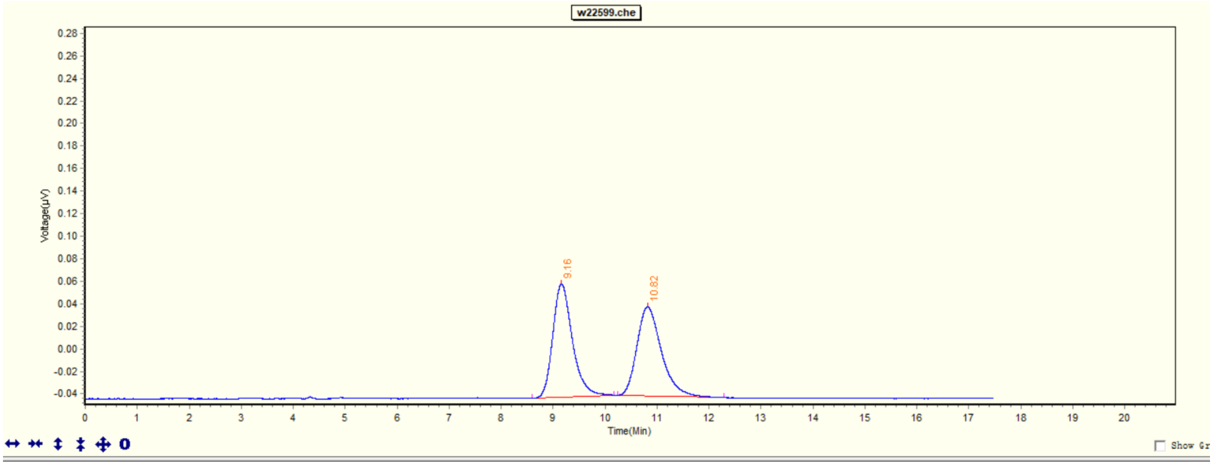

No.	Retention Time	Peak Area	Peak Height	Peak Area(%)	Peak Width	Peak Type
1	9.16	1304431	50118	50.04%	1.574	BB
2	10.82	1302601	39599	49.96%	2.045	BB
Total		2,607,032	89,717	100.00%		

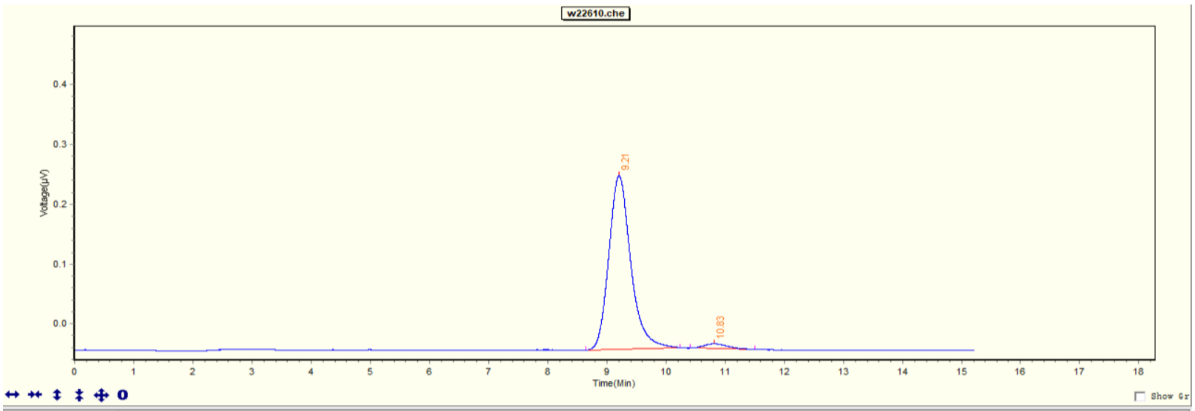

No.	Retention Time	Peak Area	Peak Height	Peak Area(%)	Peak Width	Peak Type
1	9.21	3729257	145002	97.03%	1.593	BB
2	10.83	114018	4053	2.97%	1.104	BB
Total		3,843,275	149,055	100.00%		

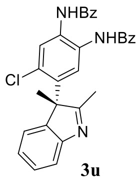

3u

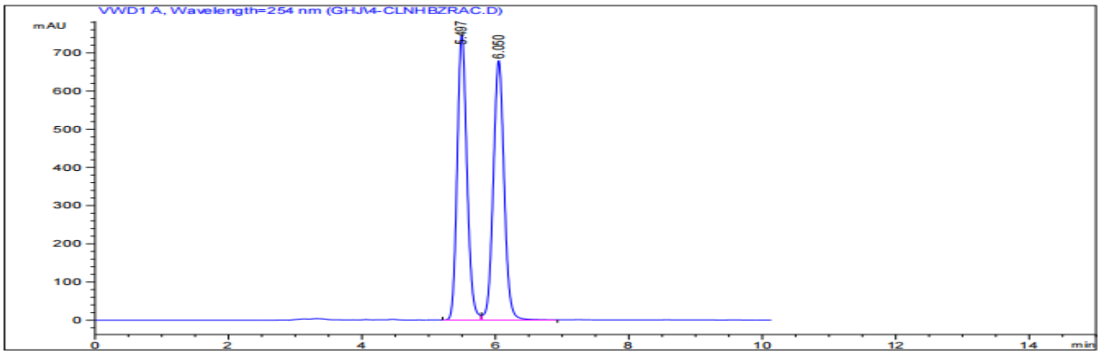

===
Area Percent Report
===

Sorted By : Signal
Multiplier : 1.0000
Dilution : 1.0000
Use Multiplier & Dilution Factor with ISTDs

Signal 1: VWD1 A, Wavelength=254 nm

Peak #	RetTime [min]	Type	Width [min]	Area mAU *s	Height [mAU]	Area %
1	5.497	VV	0.1567	7595.26367	745.69446	49.6686
2	6.050	VB	0.1745	7696.61133	678.64386	50.3314

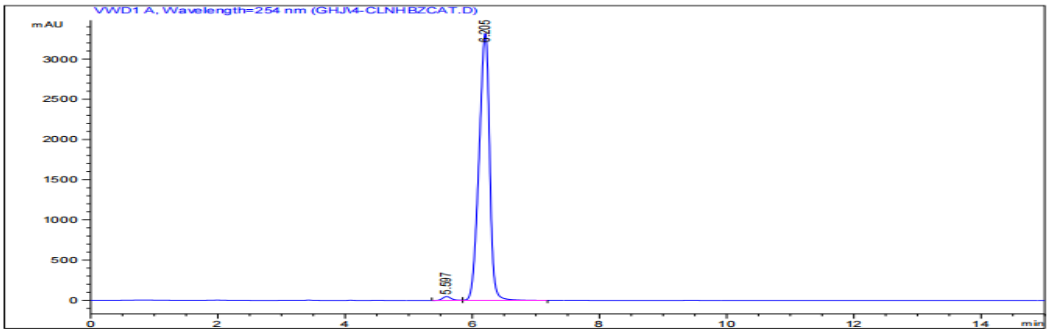

===
Area Percent Report
===

Sorted By : Signal
Multiplier : 1.0000
Dilution : 1.0000
Use Multiplier & Dilution Factor with ISTDs

Signal 1: VWD1 A, Wavelength=254 nm

Peak #	RetTime [min]	Type	Width [min]	Area mAU *s	Height [mAU]	Area %
1	5.597	BV	0.1470	452.41818	46.51910	1.1659
2	6.205	VB	0.1785	3.83517e4	3319.17261	98.8341

Totals : 3.88042e4 3365.69171

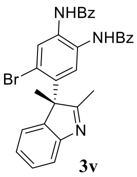

3v

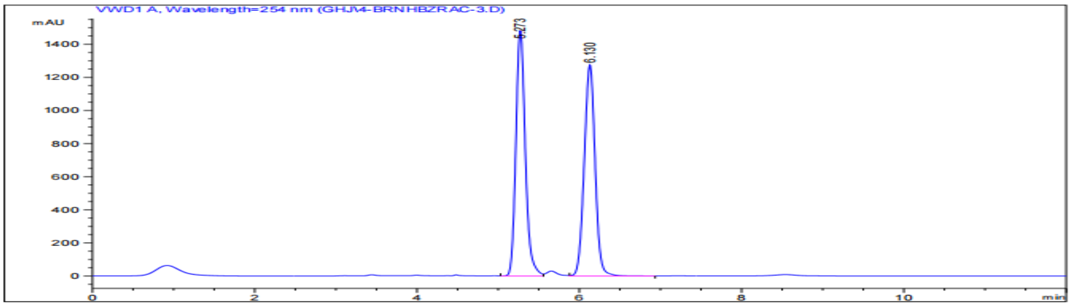


```
=====================================================================
                        Area Percent Report
=====================================================================

Sorted By             :        Signal
Multiplier            :        1.0000
Dilution              :        1.0000
Use Multiplier & Dilution Factor with ISTDs

Signal 1: VWD1 A, Wavelength=254 nm

Peak RetTime Type  Width     Area      Height     Area
  #   [min]        [min]   mAU   *s   [mAU   ]      %
----|-------|----|-------|----------|----------|--------|
   1   5.273 VV    0.1248 1.19796e4 1483.28882 50.2066
   2   6.130 VB    0.1436 1.18810e4 1275.54578 49.7934
```

```
Sample Info      : IK 7/3 1mL/min
```

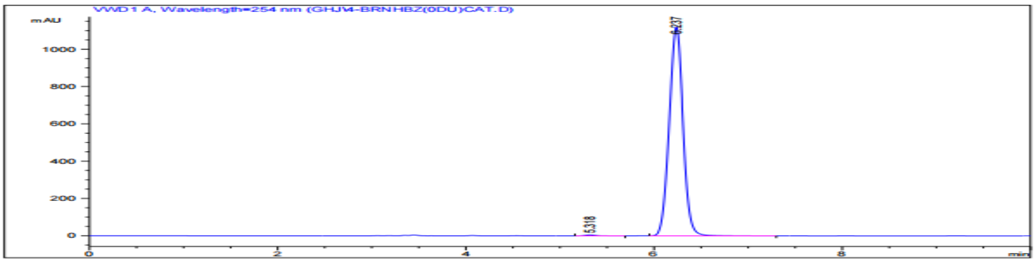


```
=====================================================================
                        Area Percent Report
=====================================================================

Sorted By             :        Signal
Multiplier            :        1.0000
Dilution              :        1.0000
Use Multiplier & Dilution Factor with ISTDs

Signal 1: VWD1 A, Wavelength=254 nm

Peak RetTime Type  Width     Area      Height     Area
  #   [min]        [min]   mAU   *s   [mAU   ]      %
----|-------|----|-------|----------|----------|--------|
   1   5.318 BB    0.1353   44.99626    5.08511   0.3927
   2   6.237 VB    0.1568 1.14122e4 1119.48132 99.6073

Totals :                  1.14572e4 1124.56643
```

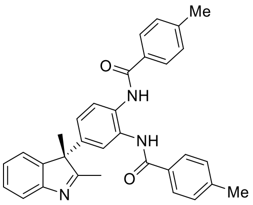

3w

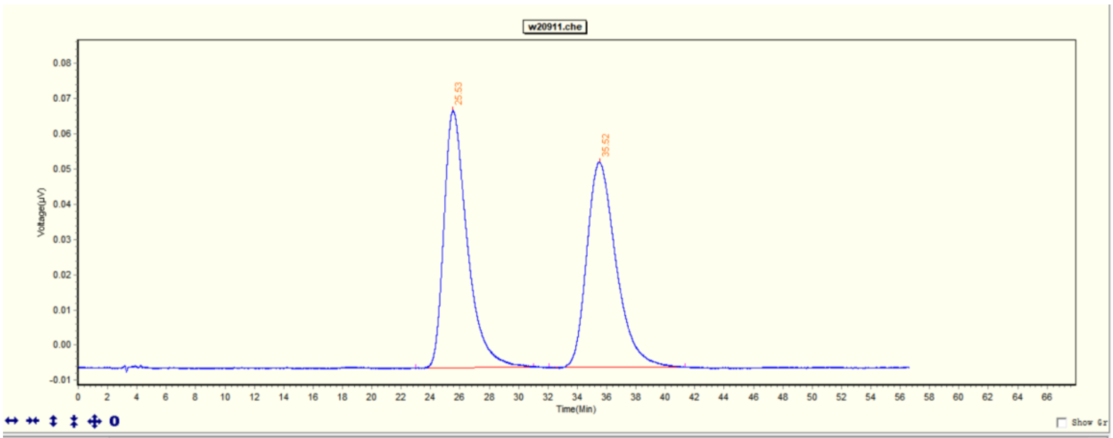

Integration Result | Calculation Result | TimeTable

No.	Retention Time	Peak Area	Peak Height	Peak Area(%)	Peak Width	Peak Type
1	25.53	4029973	36505	49.87%	8.032	BB
2	35.52	4050570	29176	50.13%	9.265	BB
Total		8,080,543	65,681	100.00%		

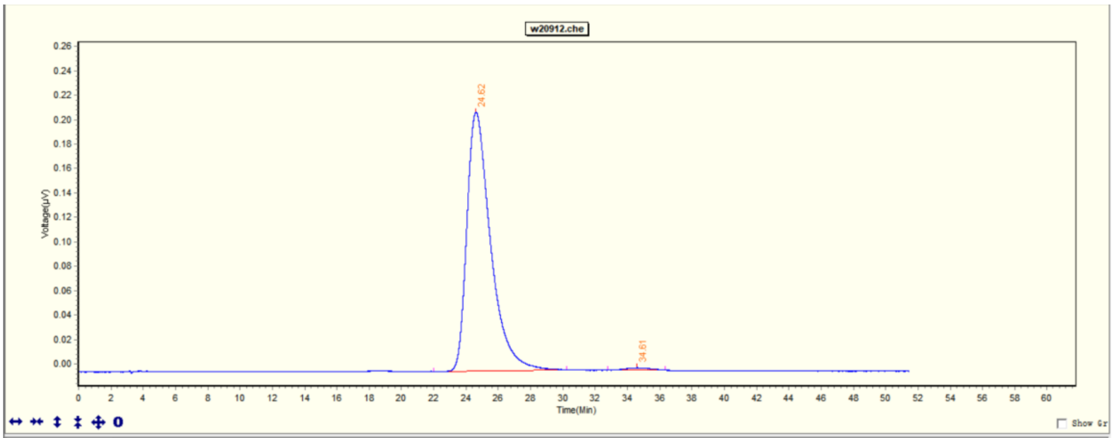

Integration Result | Calculation Result | TimeTable

No.	Retention Time	Peak Area	Peak Height	Peak Area(%)	Peak Width	Peak Type
1	24.62	10780437	105698	99.09%	8.233	BB
2	34.61	99033	974	0.91%	3.559	BB
Total		10,879,470	106,672	100.00%		

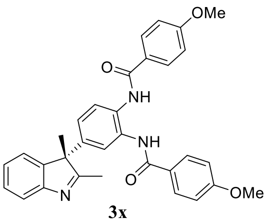

3x

Sample Info : IC-3 7/3 1mL/min

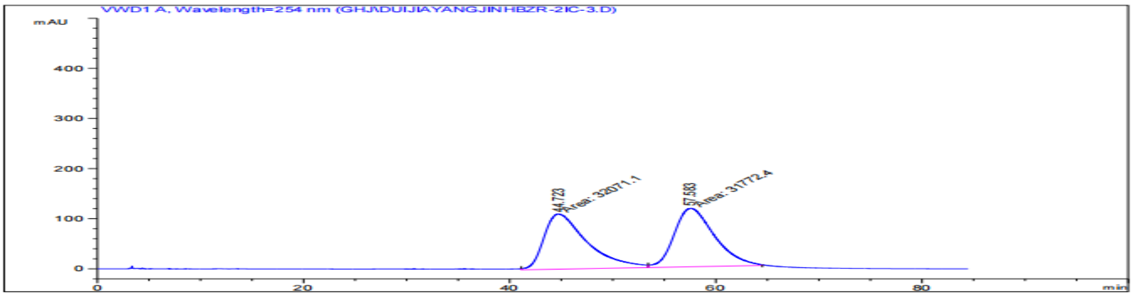

Area Percent Report

Sorted By : Signal
Multiplier : 1.0000
Dilution : 1.0000
Use Multiplier & Dilution Factor with ISTDs

Signal 1: VWD1 A, Wavelength=254 nm

Peak #	RetTime [min]	Type	Width [min]	Area mAU *s	Height [mAU]	Area %
1	44.723	MF	4.8392	3.20711e4	110.45690	50.2340
2	57.583	FM	4.5361	3.17724e4	116.73884	49.7660
Totals :				6.38434e4	227.19574	

Sample Info : IC-3 7/3 1mL/min

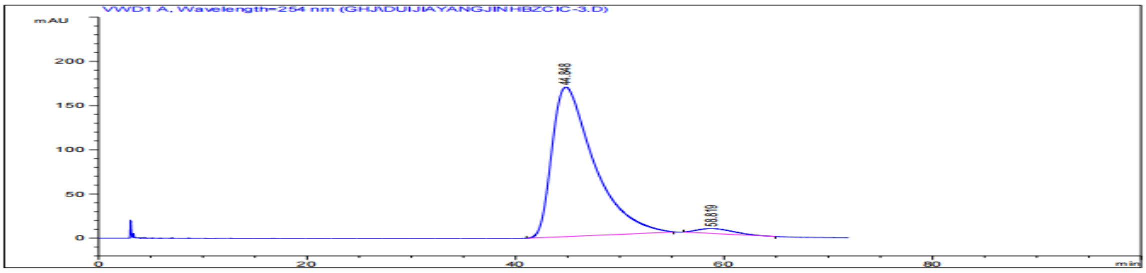

Area Percent Report

Sorted By : Signal
Multiplier : 1.0000
Dilution : 1.0000
Use Multiplier & Dilution Factor with ISTDs

Signal 1: VWD1 A, Wavelength=254 nm

Peak #	RetTime [min]	Type	Width [min]	Area mAU *s	Height [mAU]	Area %
1	44.848	BB	4.0105	4.74106e4	169.03383	97.2828
2	58.819	BB	2.7798	1324.19678	5.56925	2.7172
Totals :				4.87348e4	174.60308	

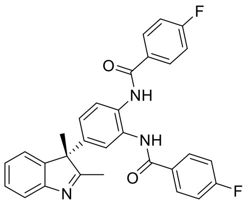

3y

Sample Info : IC-3 7/3 1mL/min

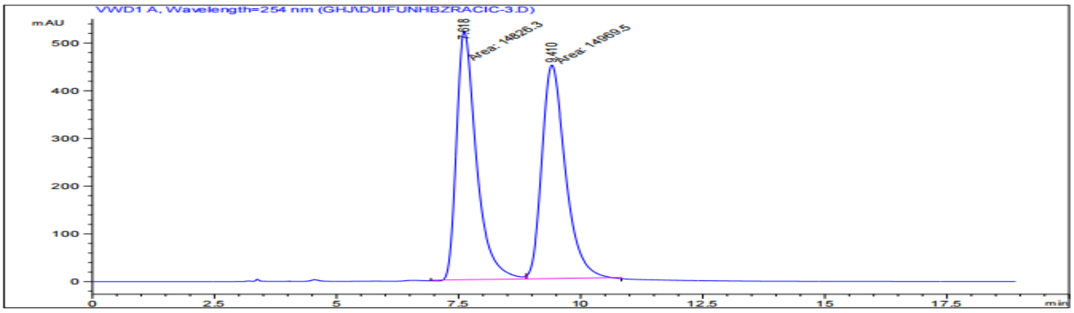

Area Percent Report

Sorted By : Signal
Multiplier : 1.0000
Dilution : 1.0000
Use Multiplier & Dilution Factor with ISTDs

Signal 1: VWD1 A, Wavelength=254 nm

Peak #	RetTime [min]	Type	Width [min]	Area mAU *s	Height [mAU]	Area %
1	7.618	MF	0.4746	1.48263e4	520.62152	49.7598
2	9.410	FM	0.5580	1.49695e4	447.13208	50.2402
Totals :				2.97958e4	967.75360	

Sample Info : IC-3 7/3 1mL/min

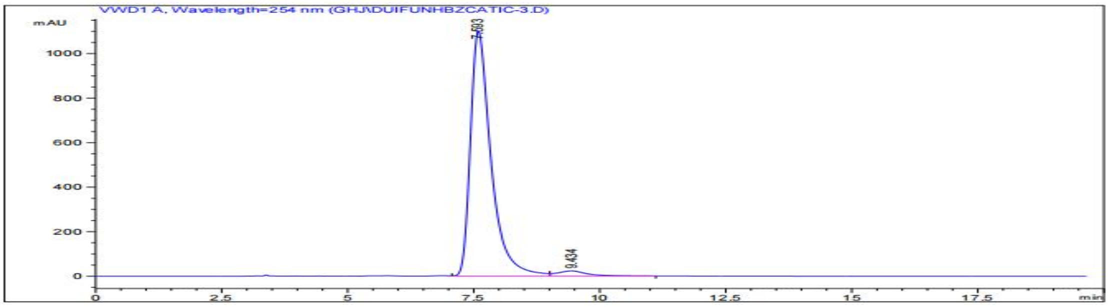

Area Percent Report

Sorted By : Signal
Multiplier : 1.0000
Dilution : 1.0000
Use Multiplier & Dilution Factor with ISTDs

Signal 1: VWD1 A, Wavelength=254 nm

Peak #	RetTime [min]	Type	Width [min]	Area mAU *s	Height [mAU]	Area %
1	7.593	VV	0.4280	3.13066e4	1100.48596	97.0826
2	9.434	VB	0.5946	940.78937	22.81537	2.9174
Totals :				3.22473e4	1123.30133	

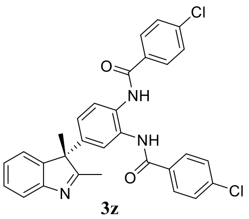

3z

Sample Info : IC-3 7/3 1mL/min

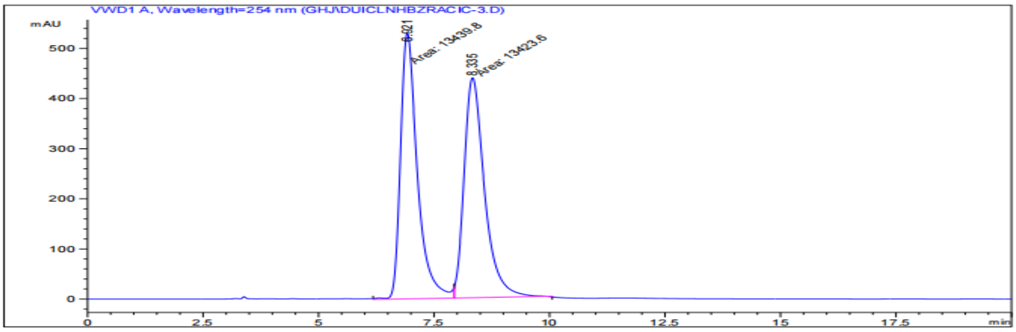

Area Percent Report

Sorted By : Signal
Multiplier : 1.0000
Dilution : 1.0000
Use Multiplier & Dilution Factor with ISTDs

Signal 1: VWD1 A, Wavelength=254 nm

Peak #	RetTime [min]	Type	Width [min]	Area mAU *s	Height [mAU]	Area %
1	6.921	MF	0.4223	1.34398e4	530.45667	50.0300
2	8.335	FM	0.5099	1.34236e4	438.74319	49.9700

Totals : 2.68634e4 969.19986

Sample Info : IC-3 7/3 1mL/min

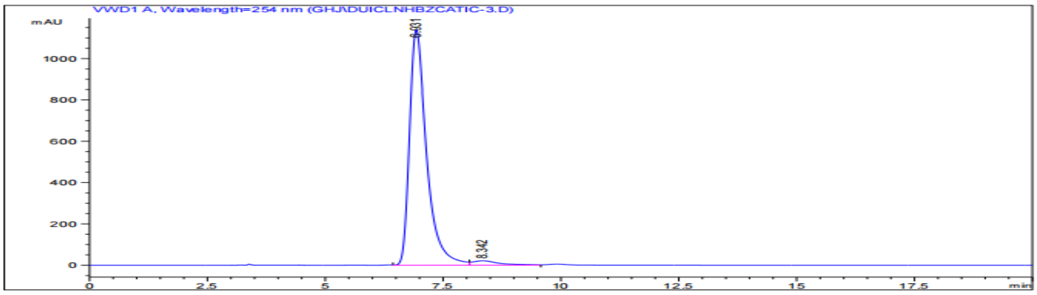

Area Percent Report

Sorted By : Signal
Multiplier : 1.0000
Dilution : 1.0000
Use Multiplier & Dilution Factor with ISTDs

Signal 1: VWD1 A, Wavelength=254 nm

Peak #	RetTime [min]	Type	Width [min]	Area mAU *s	Height [mAU]	Area %
1	6.931	VV	0.3777	2.86164e4	1138.36560	97.0462
2	8.342	VB	0.5775	871.01202	21.30415	2.9538

Totals : 2.94874e4 1159.66975

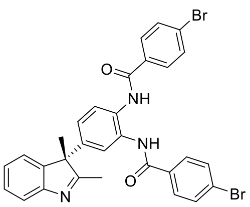

3a′

Sample Info : IC-3 7/3 1mL/min

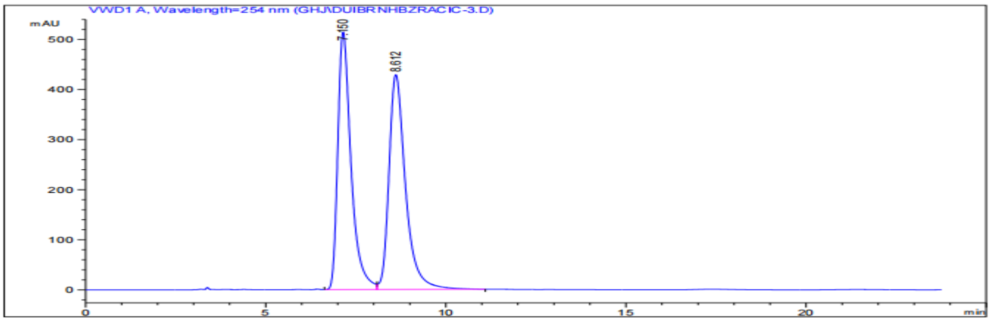

===

Area Percent Report

===

Sorted By : Signal
Multiplier : 1.0000
Dilution : 1.0000
Use Multiplier & Dilution Factor with ISTDs

Signal 1: VWD1 A, Wavelength=254 nm

Peak #	RetTime [min]	Type	Width [min]	Area mAU *s	Height [mAU]	Area %
1	7.150	VV	0.3861	1.31558e4	513.82544	48.9811
2	8.612	VB	0.4818	1.37031e4	429.06195	51.0189
Totals :				2.68589e4	942.88739	

Sample Info : IC-3 7/3 1mL/min

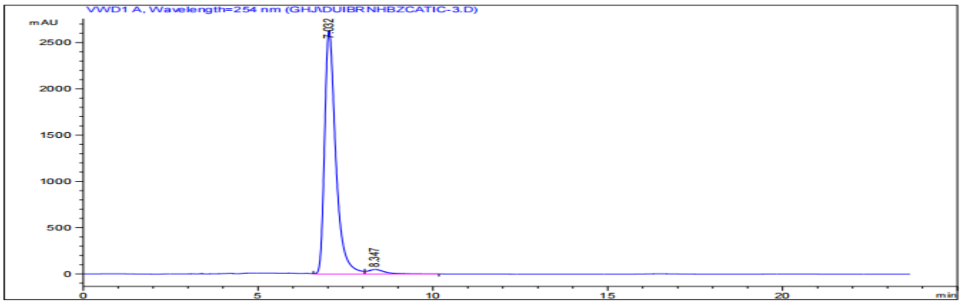

===

Area Percent Report

===

Sorted By : Signal
Multiplier : 1.0000
Dilution : 1.0000
Use Multiplier & Dilution Factor with ISTDs

Signal 1: VWD1 A, Wavelength=254 nm

Peak #	RetTime [min]	Type	Width [min]	Area mAU *s	Height [mAU]	Area %
1	7.032	VV	0.3355	5.85550e4	2629.88940	97.1709
2	8.347	VB	0.4956	1704.83569	49.42069	2.8291
Totals :				6.02598e4	2679.31010	

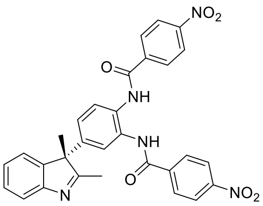

3b′

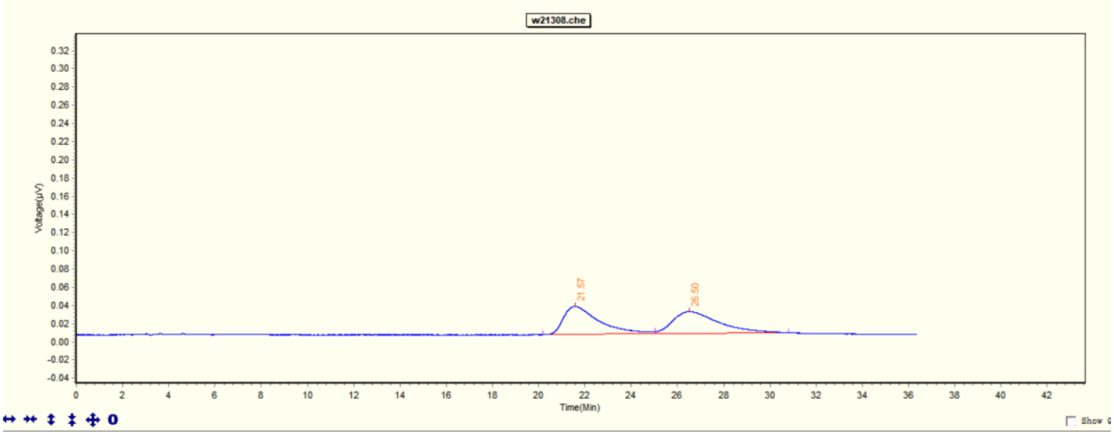

No.	Retention Time	Peak Area	Peak Height	Peak Area(%)	Peak Width	Peak Type
1	21.57	1671155	15507	50.09%	4.858	BV
2	26.50	1665396	12034	49.91%	5.76	VB
Total		3,336,551	27,541	100.00%		

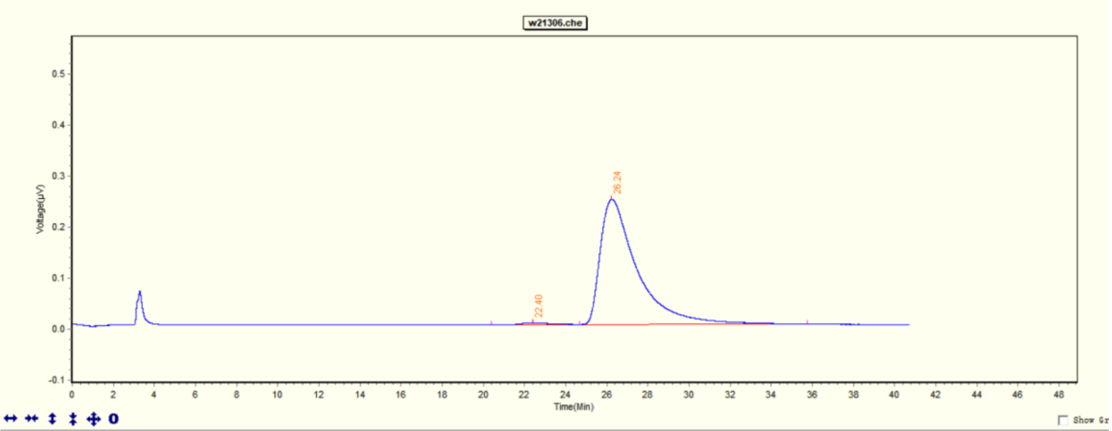

No.	Retention Time	Peak Area	Peak Height	Peak Area(%)	Peak Width	Peak Type
1	22.40	201376	1965	1.29%	4.286	BV
2	26.24	15396494	122744	98.71%	11.095	VB
Total		15,597,870	124,709	100.00%		

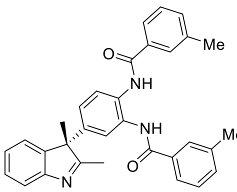

3c′

```
Sample Info       : IC-3 7/3 1mL/min
```

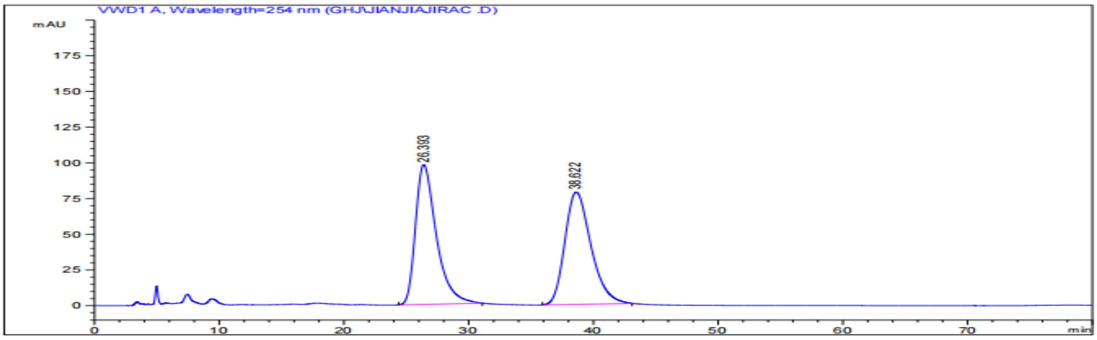


```
=====================================================================
                          Area Percent Report
=====================================================================

Sorted By             :        Signal
Multiplier            :        1.0000
Dilution              :        1.0000
Use Multiplier & Dilution Factor with ISTDs

Signal 1: VWD1 A, Wavelength=254 nm
```

Peak #	RetTime [min]	Type	Width [min]	Area mAU *s	Height [mAU]	Area %
1	26.393	BB	1.7408	1.13830e4	97.80228	49.8884
2	38.622	BB	2.1623	1.14340e4	78.56654	50.1116
Totals :				2.28170e4	176.36881	

```
Sample Info       : IC-3 7/3 1mL/min
```

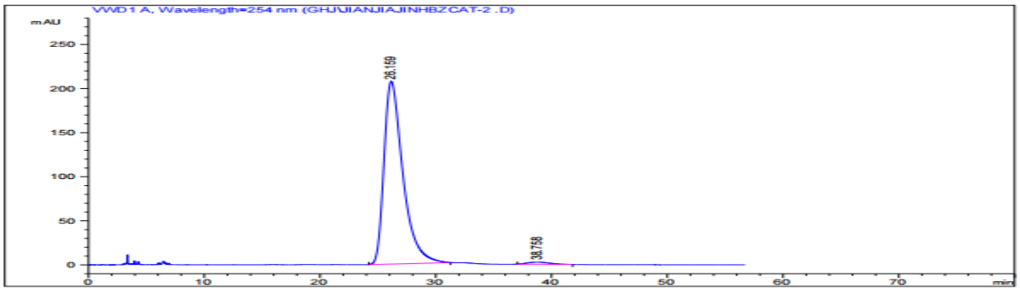


```
=====================================================================
                          Area Percent Report
=====================================================================

Sorted By             :        Signal
Multiplier            :        1.0000
Dilution              :        1.0000
Use Multiplier & Dilution Factor with ISTDs

Signal 1: VWD1 A, Wavelength=254 nm
```

Peak #	RetTime [min]	Type	Width [min]	Area mAU *s	Height [mAU]	Area %
1	26.159	BB	1.7092	2.35571e4	207.21872	98.6236
2	38.758	BB	1.5733	328.77118	2.45610	1.3764
Totals :				2.38858e4	209.67482	

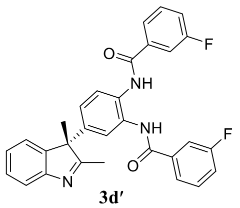

3d′

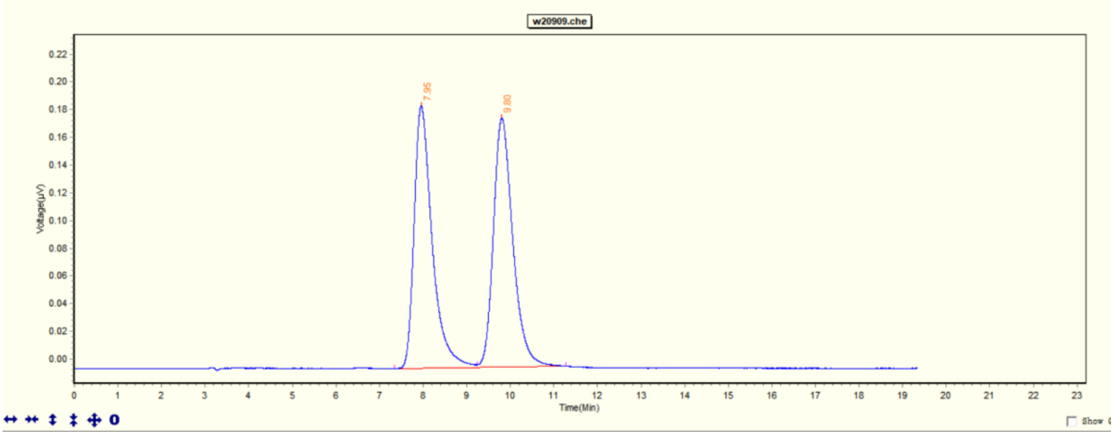

Integration Result | Calculation Result | TimeTable

No.	Retention Time	Peak Area	Peak Height	Peak Area(%)	Peak Width	Peak Type
1	7.95	2724486	94587	49.55%	1.896	BV
2	9.80	2774272	89833	50.45%	2.034	VB
Total		5,498,758	184,420	100.00%		

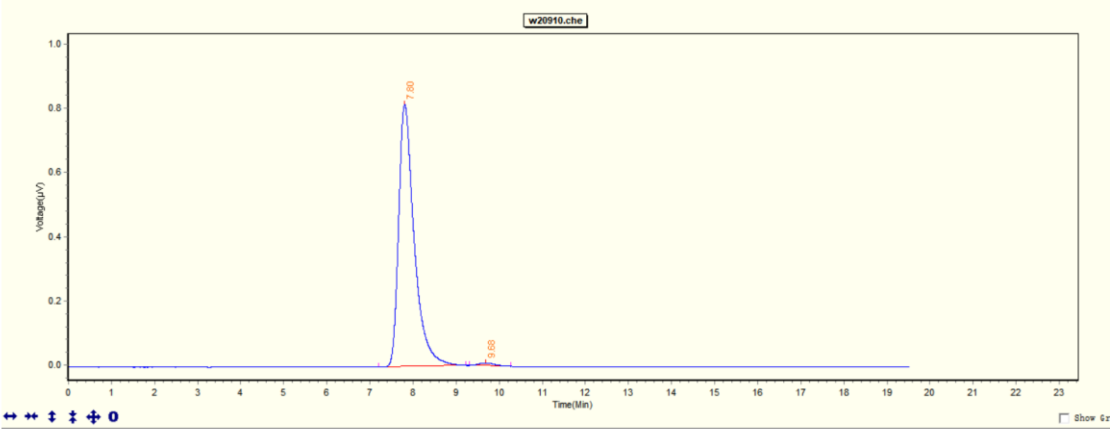

Integration Result | Calculation Result | TimeTable

No.	Retention Time	Peak Area	Peak Height	Peak Area(%)	Peak Width	Peak Type
1	7.80	10264769	408063	98.89%	2.025	BB
2	9.68	114880	4480	1.11%	0.962	BB
Total		10,379,649	412,543	100.00%		

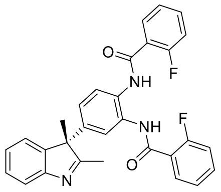

3e′

Sample Info : IC-3 7/3 1mL/min

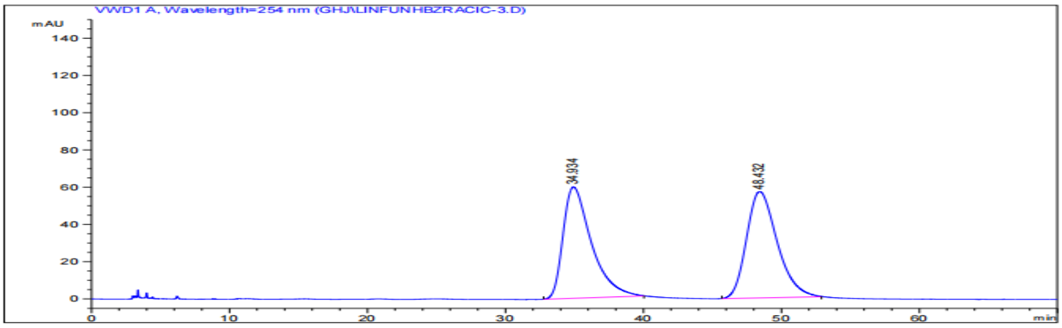

Area Percent Report

Sorted By : Signal
Multiplier : 1.0000
Dilution : 1.0000
Use Multiplier & Dilution Factor with ISTDs

Signal 1: VWD1 A, Wavelength=254 nm

Peak #	RetTime [min]	Type	Width [min]	Area mAU *s	Height [mAU]	Area %
1	34.934	BB	2.1060	8556.89648	59.75449	49.2552
2	48.432	BB	2.2305	8815.66602	57.06126	50.7448

Totals : 1.73726e4 116.81575

Sample Info : IC-3 7/3 1mL/min

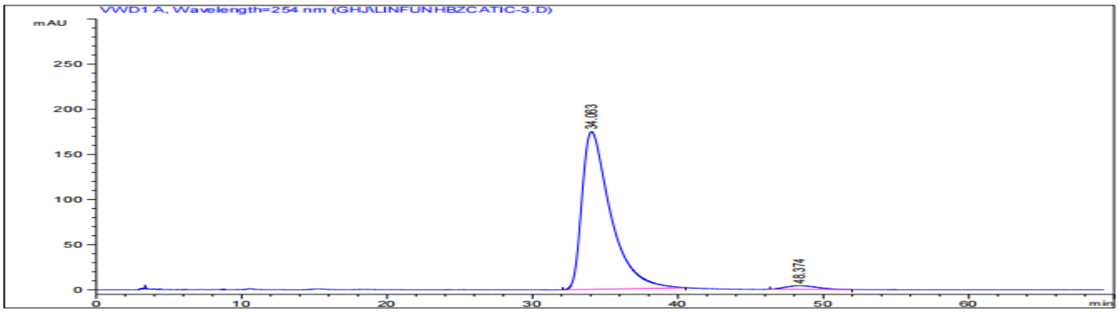

Area Percent Report

Sorted By : Signal
Multiplier : 1.0000
Dilution : 1.0000
Use Multiplier & Dilution Factor with ISTDs

Signal 1: VWD1 A, Wavelength=254 nm

Peak #	RetTime [min]	Type	Width [min]	Area mAU *s	Height [mAU]	Area %
1	34.063	BB	2.0020	2.39037e4	174.59727	97.5295
2	48.374	BB	1.7771	605.49225	4.03796	2.4705

Totals : 2.45092e4 178.63524

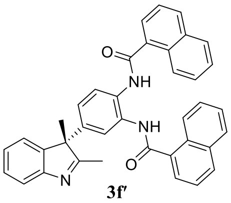

3f′

Sample Info : IC-3 7/3

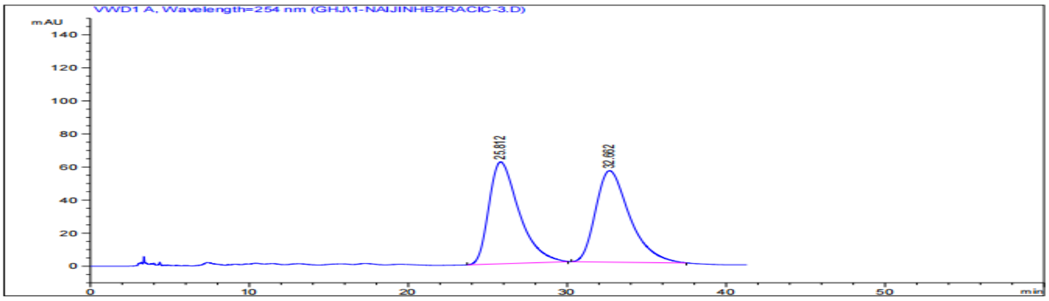

Area Percent Report

Sorted By : Signal
Multiplier : 1.0000
Dilution : 1.0000
Use Multiplier & Dilution Factor with ISTDs

Signal 1: VWD1 A, Wavelength=254 nm

Peak #	RetTime [min]	Type	Width [min]	Area mAU *s	Height [mAU]	Area %
1	25.812	BB	1.9788	8129.48438	61.68246	49.6707
2	32.662	BB	2.2476	8237.27148	55.34175	50.3293

Totals : 1.63668e4 117.02421

Sample Info : IC-3 7/3 1mL/min

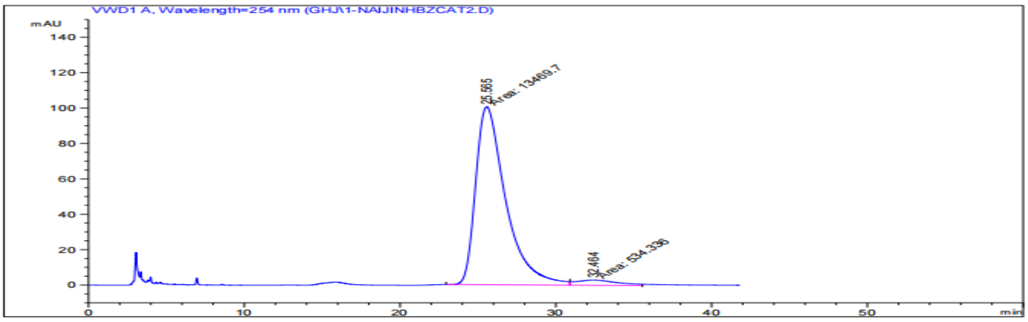

Area Percent Report

Sorted By : Signal
Multiplier : 1.0000
Dilution : 1.0000
Use Multiplier & Dilution Factor with ISTDs

Signal 1: VWD1 A, Wavelength=254 nm

Peak #	RetTime [min]	Type	Width [min]	Area mAU *s	Height [mAU]	Area %
1	25.565	MF	2.2335	1.34697e4	100.51509	96.1844
2	32.464	FM	3.0663	534.33630	2.90433	3.8156

Totals : 1.40041e4 103.41942

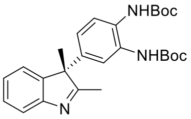

3g′

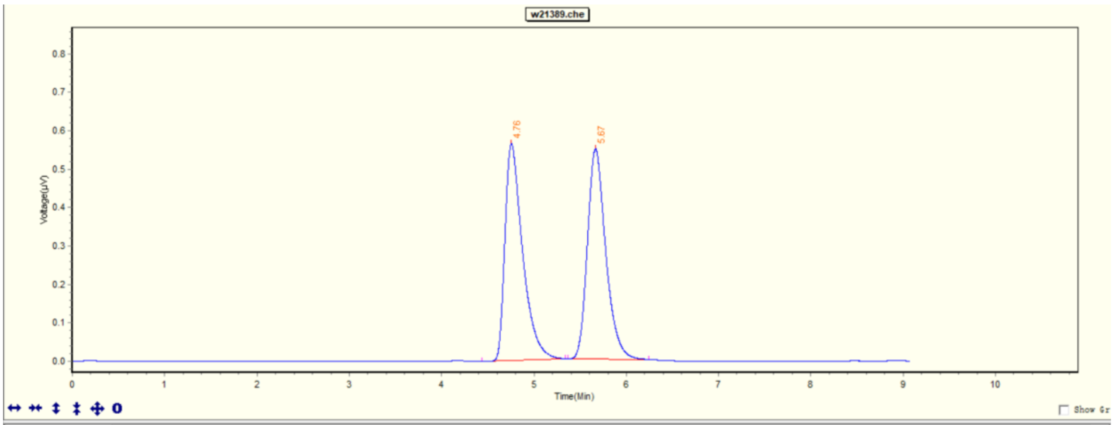

No.	Retention Time	Peak Area	Peak Height	Peak Area(%)	Peak Width	Peak Type
1	4.76	3730623	281544	49.74%	0.904	BB
2	5.67	3770138	273034	50.26%	0.877	BB
Total		7,500,761	554,578	100.00%		

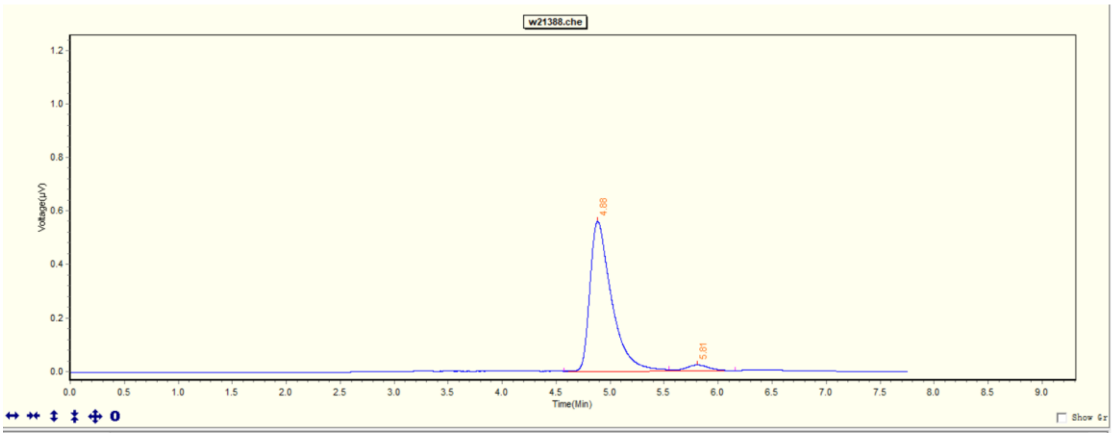

No.	Retention Time	Peak Area	Peak Height	Peak Area(%)	Peak Width	Peak Type
1	4.88	3882172	279666	96.05%	0.972	BV
2	5.81	159628	10762	3.95%	0.612	VB
Total		4,041,800	290,428	100.00%		

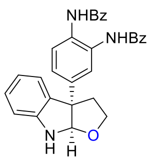

4a

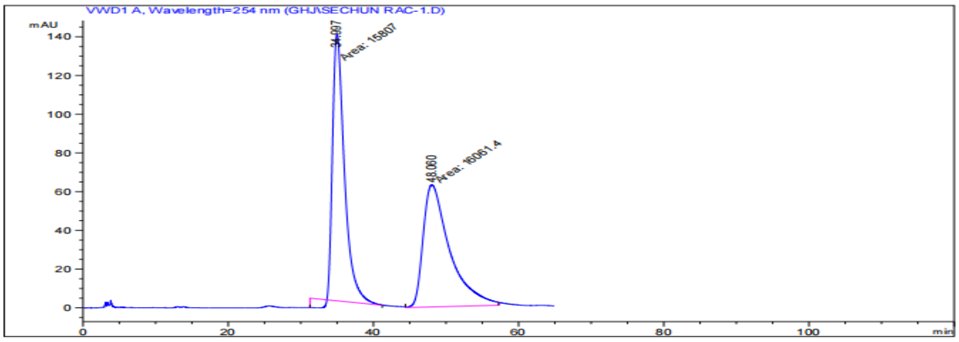


```
=====================================================================
                         Area Percent Report
=====================================================================

Sorted By             :      Signal
Multiplier            :      1.0000
Dilution              :      1.0000
Use Multiplier & Dilution Factor with ISTDs

Signal 1: VWD1 A, Wavelength=254 nm

Peak RetTime Type  Width     Area      Height     Area
  #   [min]        [min]   mAU   *s   [mAU  ]       %
----|-------|----|-------|----------|----------|--------|
   1  34.997 MM     1.9117 1.58070e4  137.80620  49.6008
   2  48.060 MM     4.2404 1.60614e4   63.12798  50.3992
```

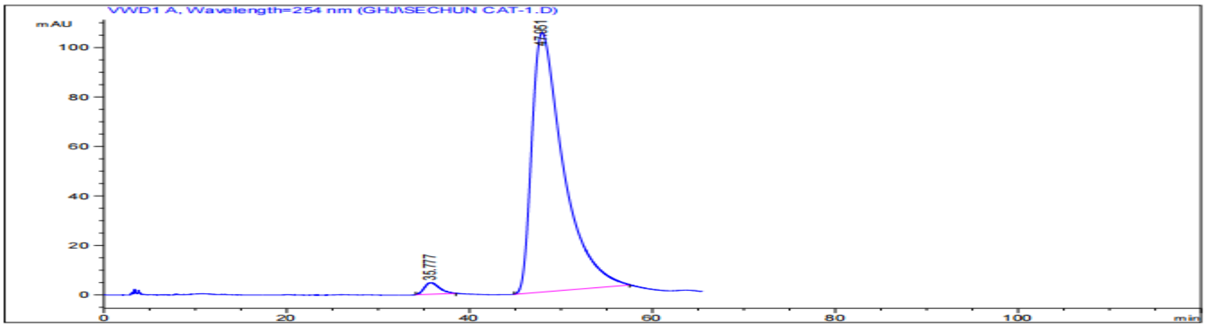


```
=====================================================================
                         Area Percent Report
=====================================================================

Sorted By             :      Signal
Multiplier            :      1.0000
Dilution              :      1.0000
Use Multiplier & Dilution Factor with ISTDs

Signal 1: VWD1 A, Wavelength=254 nm

Peak RetTime Type  Width     Area      Height     Area
  #   [min]        [min]   mAU   *s   [mAU  ]       %
----|-------|----|-------|----------|----------|--------|
   1  35.777 BB     1.3974  535.86786    4.65817   2.0884
   2  47.951 BB     3.4041 2.51234e4  104.85087  97.9116
```

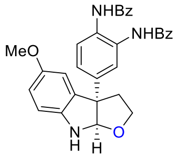

4b

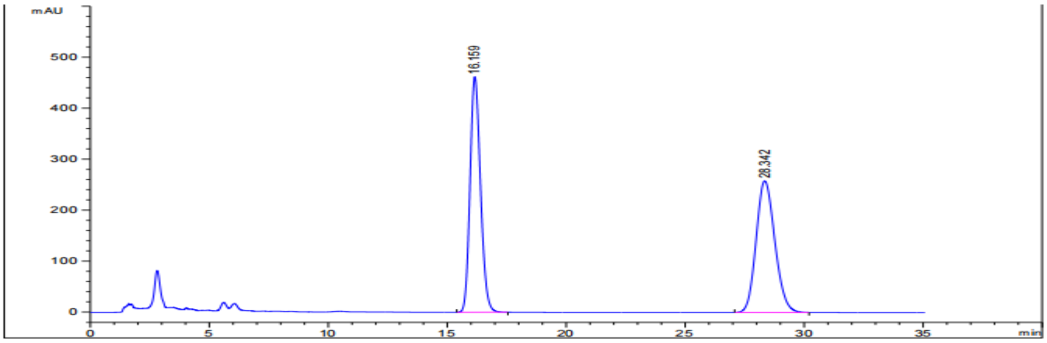

Area Percent Report

Sorted By : Signal
Multiplier : 1.0000
Dilution : 1.0000
Sample Amount : 1.00000 [ng/ul] (not used in calc.)
Use Multiplier & Dilution Factor with ISTDs

Signal 1: VWD1 A, Wavelength=254 nm

Peak #	RetTime [min]	Type	Width [min]	Area mAU *s	Height [mAU]	Area %
1	16.159	BB	0.4744	1.41161e4	462.02972	50.0066
2	28.342	BB	0.8478	1.41124e4	258.00735	49.9934

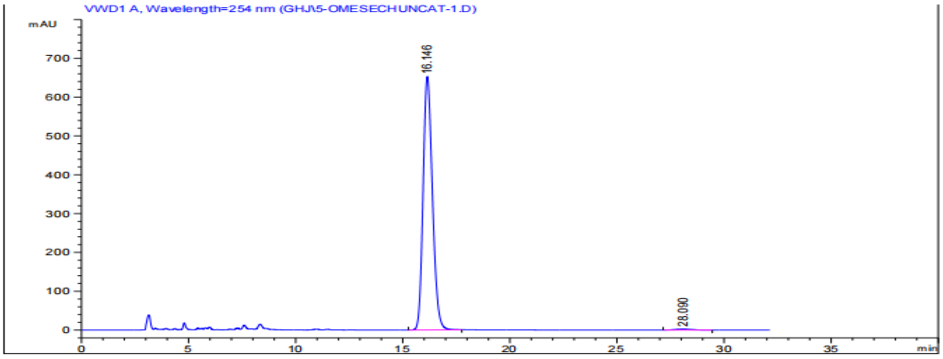

Area Percent Report

Sorted By : Signal
Multiplier : 1.0000
Dilution : 1.0000
Sample Amount : 1.00000 [ng/ul] (not used in calc.)
Use Multiplier & Dilution Factor with ISTDs

Signal 1: VWD1 A, Wavelength=254 nm

Peak #	RetTime [min]	Type	Width [min]	Area mAU *s	Height [mAU]	Area %
1	16.146	BB	0.4673	1.97775e4	652.38123	99.2334
2	28.090	BB	0.8013	152.79173	2.87183	0.7666

Totals : 1.99303e4 655.25306

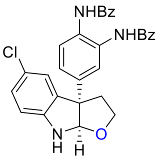

4c

Sample Info : IG-3 7/3 1mL/min

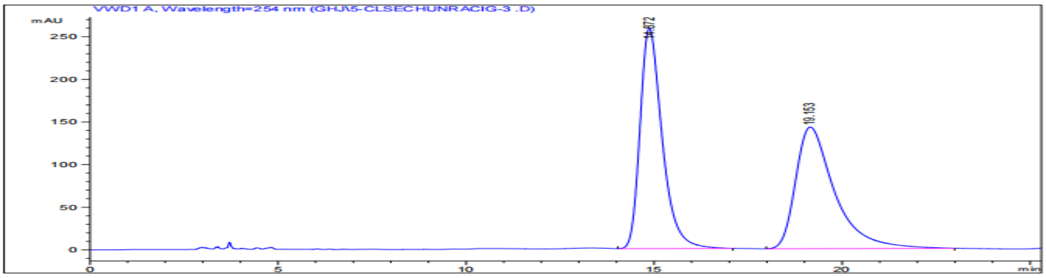

Area Percent Report

Sorted By : Signal
Multiplier : 1.0000
Dilution : 1.0000
Use Multiplier & Dilution Factor with ISTDs

Signal 1: VWD1 A, Wavelength=254 nm

Peak #	RetTime [min]	Type	Width [min]	Area mAU *s	Height [mAU]	Area %
1	14.872	VB	0.6229	1.05799e4	258.62711	50.7801
2	19.153	BB	1.0714	1.02548e4	142.69110	49.2199
Totals :				2.08347e4	401.31821	

Sample Info : IG-3 7/3 1mL/min

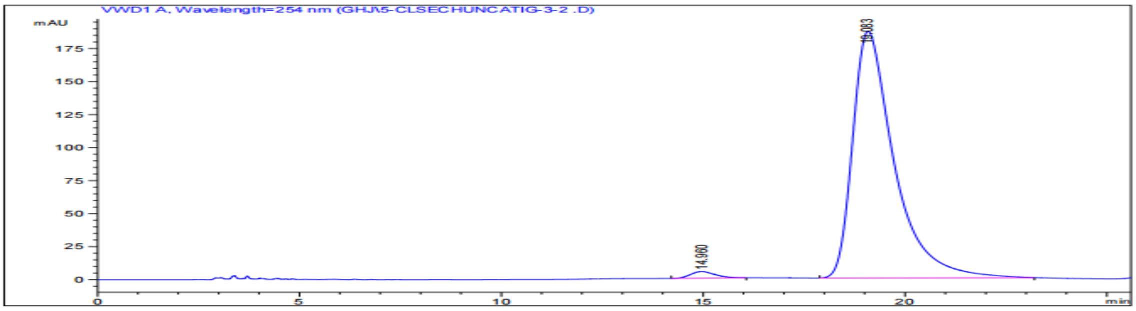

Area Percent Report

Sorted By : Signal
Multiplier : 1.0000
Dilution : 1.0000
Use Multiplier & Dilution Factor with ISTDs

Signal 1: VWD1 A, Wavelength=254 nm

Peak #	RetTime [min]	Type	Width [min]	Area mAU *s	Height [mAU]	Area %
1	14.960	BB	0.6408	210.70200	5.00812	1.5554
2	19.083	BB	1.0644	1.33361e4	186.80394	98.4446
Totals :				1.35468e4	191.81206	

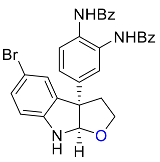

4d

Sample Info : IG-3 7/3 1mL/min

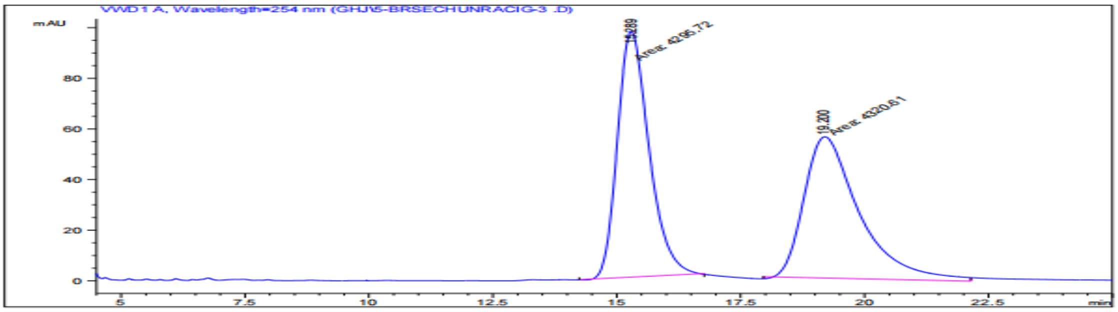

Area Percent Report

Sorted By : Signal
Multiplier : 1.0000
Dilution : 1.0000
Use Multiplier & Dilution Factor with ISTDs

Signal 1: VWD1 A, Wavelength=254 nm

Peak #	RetTime [min]	Type	Width [min]	Area mAU *s	Height [mAU]	Area %
1	15.289	MM	0.7369	4295.72119	97.15576	49.8555
2	19.200	MM	1.2909	4320.61426	55.78505	50.1445

Totals : 8616.33545 152.94081

Sample Info : IG-3 7/3 1mL/min

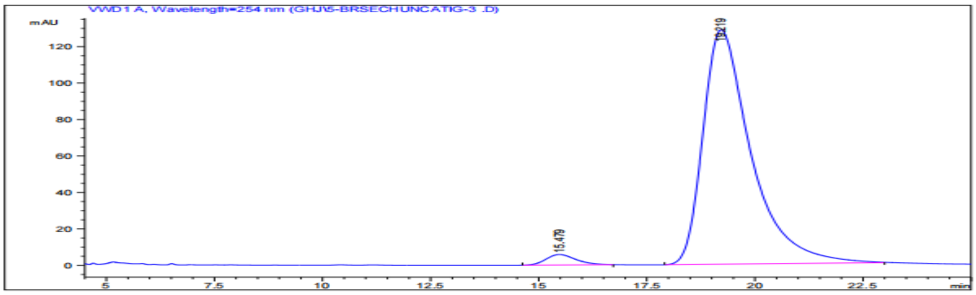

Area Percent Report

Sorted By : Signal
Multiplier : 1.0000
Dilution : 1.0000
Use Multiplier & Dilution Factor with ISTDs

Signal 1: VWD1 A, Wavelength=254 nm

Peak #	RetTime [min]	Type	Width [min]	Area mAU *s	Height [mAU]	Area %
1	15.479	BB	0.7228	278.16266	5.76790	2.7283
2	19.219	BB	1.1449	9917.39062	128.51248	97.2717

Totals : 1.01956e4 134.28039

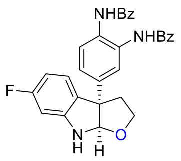

4f

Sample Info : Ib-3 7/3

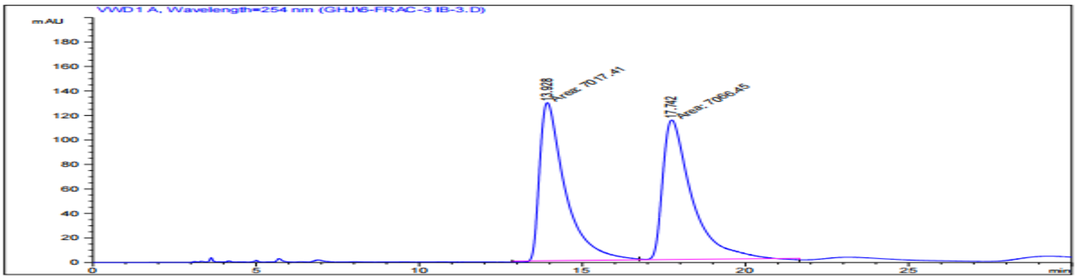

Area Percent Report

Sorted By : Signal
Multiplier : 1.0000
Dilution : 1.0000
Use Multiplier & Dilution Factor with ISTDs

Signal 1: VWD1 A, Wavelength=254 nm

Peak #	RetTime [min]	Type	Width [min]	Area mAU *s	Height [mAU]	Area %
1	13.928	MF	0.9062	7017.41455	129.05812	49.8259
2	17.742	FM	1.0338	7066.45361	113.92305	50.1741

Totals : 1.40839e4 242.98117

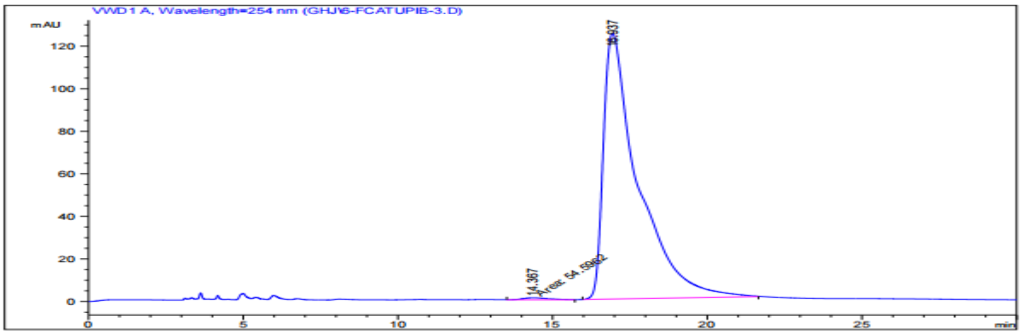

Area Percent Report

Sorted By : Signal
Multiplier : 1.0000
Dilution : 1.0000
Use Multiplier & Dilution Factor with ISTDs

Signal 1: VWD1 A, Wavelength=254 nm

Peak #	RetTime [min]	Type	Width [min]	Area mAU *s	Height [mAU]	Area %
1	14.367	MM	0.9731	54.59616	9.35052e-1	0.5583
2	16.937	BB	1.1041	9724.60840	124.81216	99.4417

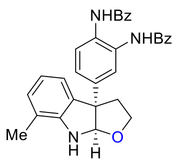

4g

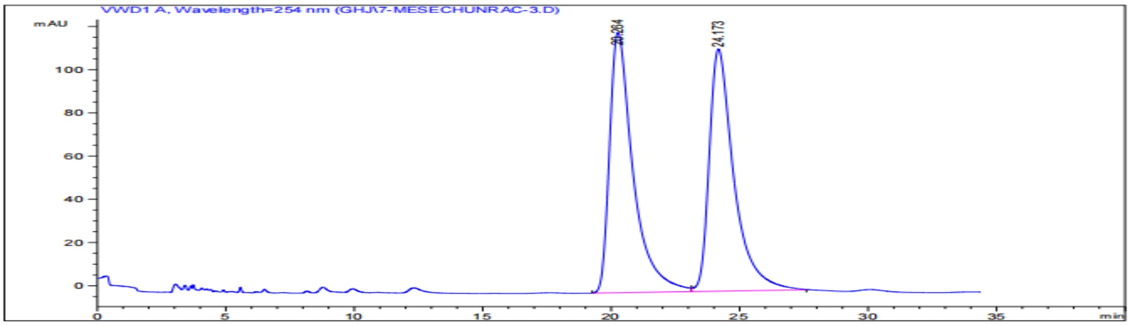


```
=====================================================================
                         Area Percent Report
=====================================================================

Sorted By             :      Signal
Multiplier            :      1.0000
Dilution              :      1.0000
Use Multiplier & Dilution Factor with ISTDs

Signal 1: VWD1 A, Wavelength=254 nm

Peak RetTime Type  Width     Area       Height     Area
  #   [min]        [min]   mAU   *s    [mAU  ]      %
----|-------|----|-------|----------|----------|--------|
  1  20.264 BB    0.9410 7670.27979  120.44926  50.6161
  2  24.173 BB    0.9964 7483.56348  112.01395  49.3839
```

```
Sample Info     : IF-3 7/3
```

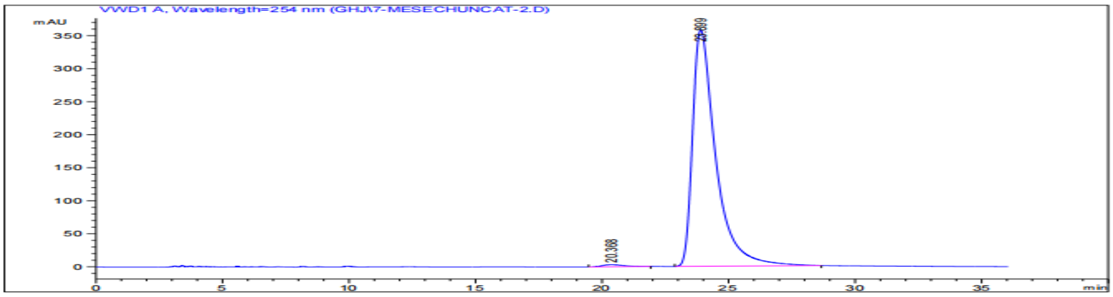


```
=====================================================================
                         Area Percent Report
=====================================================================

Sorted By             :      Signal
Multiplier            :      1.0000
Dilution              :      1.0000
Use Multiplier & Dilution Factor with ISTDs

Signal 1: VWD1 A, Wavelength=254 nm

Peak RetTime Type  Width     Area       Height     Area
  #   [min]        [min]   mAU   *s    [mAU  ]      %
----|-------|----|-------|----------|----------|--------|
  1  20.368 BB    0.8602  193.89809    3.19162   0.8432
  2  23.899 BB    0.9443 2.28018e4   357.84921  99.1568

Totals :                  2.29957e4  361.04084
```

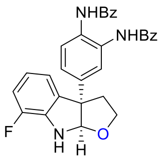

4h

Sample Info : IF-3 8/2

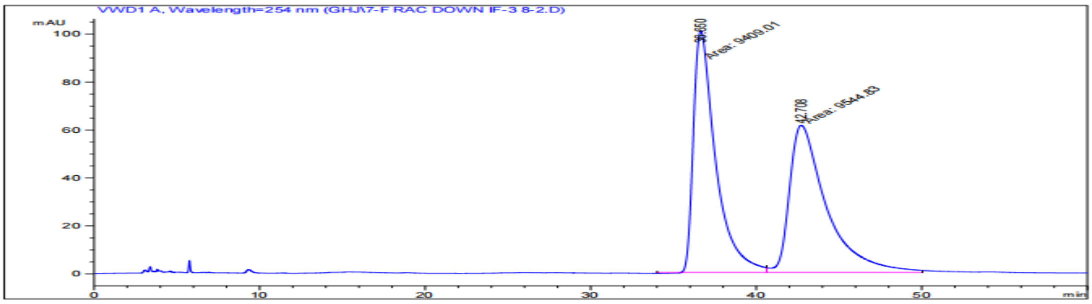

===

Area Percent Report

===

Sorted By : Signal
Multiplier : 1.0000
Dilution : 1.0000
Use Multiplier & Dilution Factor with ISTDs

Signal 1: VWD1 A, Wavelength=254 nm

Peak #	RetTime [min]	Type	Width [min]	Area mAU *s	Height [mAU]	Area %
1	36.650	MF	1.5566	9409.01367	100.74193	49.6417
2	42.708	FM	2.5899	9544.82715	61.42311	50.3583

Totals : 1.89538e4 162.16504

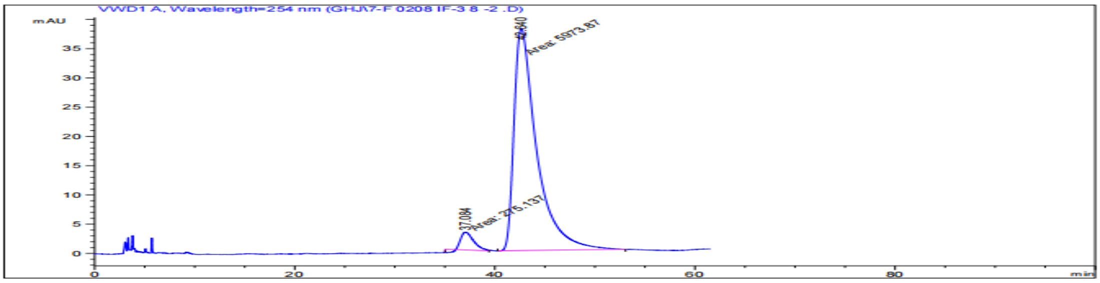

===

Area Percent Report

===

Sorted By : Signal
Multiplier : 1.0000
Dilution : 1.0000
Use Multiplier & Dilution Factor with ISTDs

Signal 1: VWD1 A, Wavelength=254 nm

Peak #	RetTime [min]	Type	Width [min]	Area mAU *s	Height [mAU]	Area %
1	37.084	MM	1.5013	275.13654	3.05433	4.4029
2	42.640	MM	2.6259	5973.86865	37.91597	95.5971

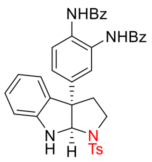

4i

Sample Info : IF-3 7:3

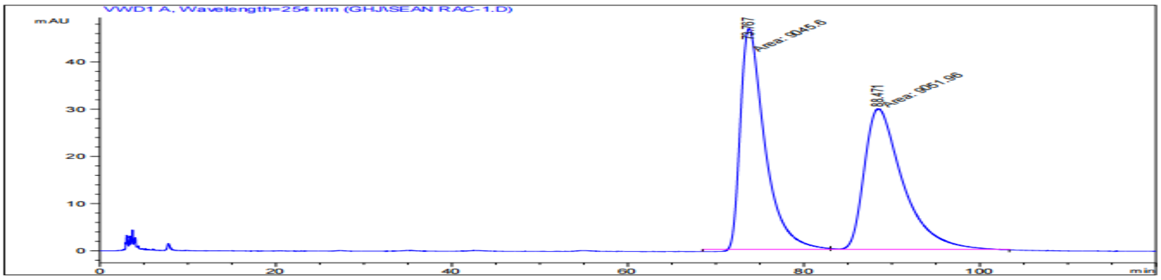


```
=====================================================================
                        Area Percent Report
=====================================================================

Sorted By             :      Signal
Multiplier            :      1.0000
Dilution              :      1.0000
Use Multiplier & Dilution Factor with ISTDs

Signal 1: VWD1 A, Wavelength=254 nm

Peak RetTime Type  Width     Area       Height     Area
  #   [min]        [min]   mAU   *s     [mAU  ]      %
----|-------|----|-------|----------|----------|--------|
   1  73.767 MF    3.2227 9045.60254   46.78126 49.9824
   2  88.471 FM    5.0616 9051.96289   29.80623 50.0176

Totals :                  1.80976e4   76.58749
```

Sample Info : IF-3 7:3

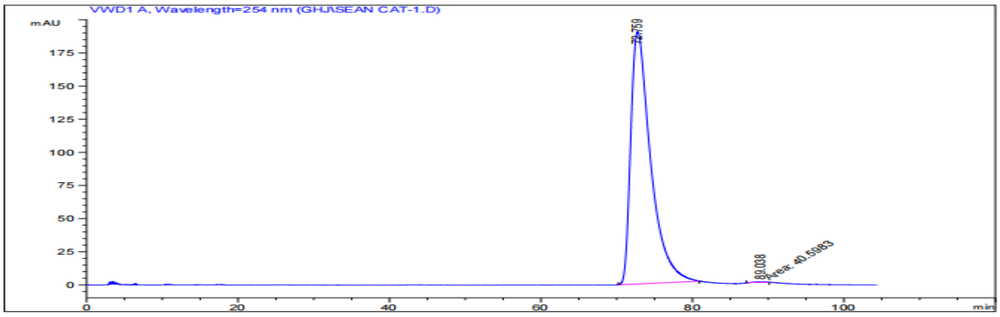


```
=====================================================================
                        Area Percent Report
=====================================================================

Sorted By             :      Signal
Multiplier            :      1.0000
Dilution              :      1.0000
Use Multiplier & Dilution Factor with ISTDs

Signal 1: VWD1 A, Wavelength=254 nm

Peak RetTime Type  Width     Area      Height     Area
  #   [min]        [min]   mAU   *s    [mAU  ]      %
----|-------|----|-------|----------|----------|--------|
   1  72.759 BB    2.6857 3.47739e4  190.38185 99.8834
   2  89.038 MM    1.6446   40.59833 4.11420e-1  0.1166

Totals :                  3.48145e4  190.79327
```

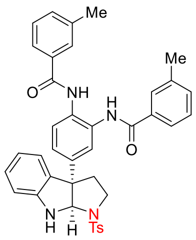

4j

Sample Info : IF 7/3 1mL/min

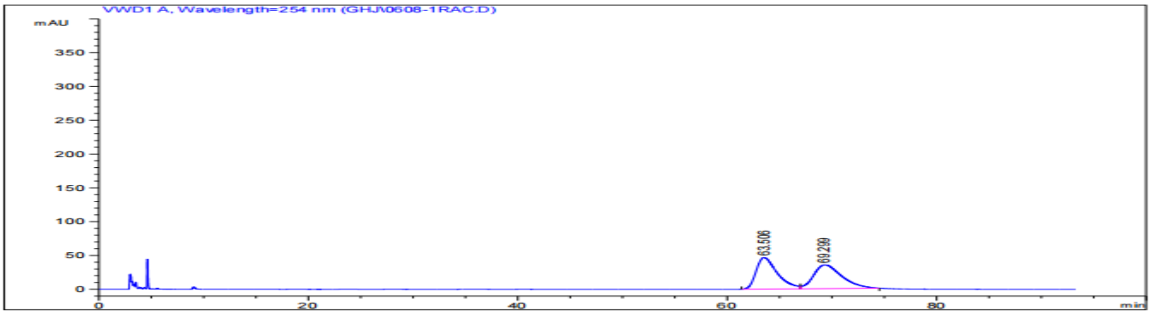

Area Percent Report

Sorted By : Signal
Multiplier : 1.0000
Dilution : 1.0000
Use Multiplier & Dilution Factor with ISTDs

Signal 1: VWD1 A, Wavelength=254 nm

Peak #	RetTime [min]	Type	Width [min]	Area mAU *s	Height [mAU]	Area %
1	63.506	BV	2.0569	6559.05176	46.68884	49.7497
2	69.299	VB	2.5093	6625.04590	35.14587	50.2503

Totals : 1.31841e4 81.83472

Sample Info : IF 7/3 1mL/min

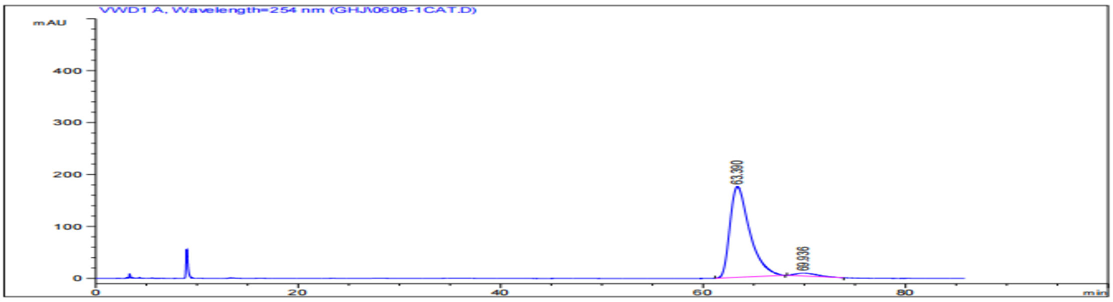

Area Percent Report

Sorted By : Signal
Multiplier : 1.0000
Dilution : 1.0000
Use Multiplier & Dilution Factor with ISTDs

Signal 1: VWD1 A, Wavelength=254 nm

Peak #	RetTime [min]	Type	Width [min]	Area mAU *s	Height [mAU]	Area %
1	63.390	BB	2.0035	2.37632e4	174.84128	96.6186
2	69.936	BB	1.7701	831.65991	5.54994	3.3814

Totals : 2.45949e4 180.39122

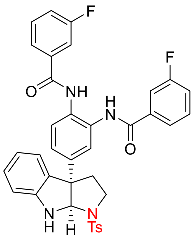

4k

Sample Info : IK 7/3

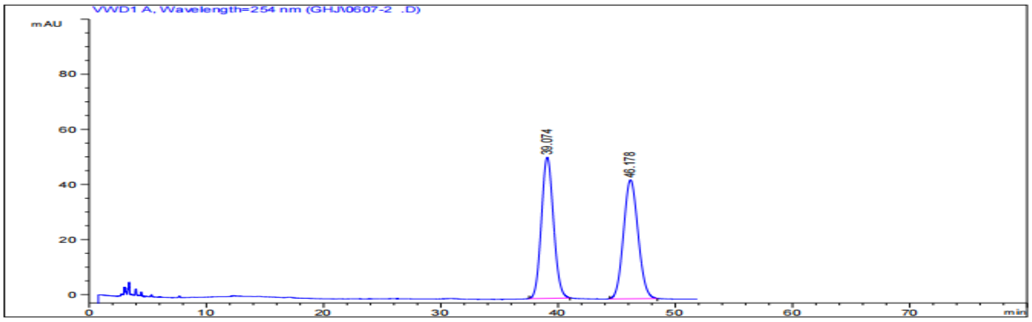

Area Percent Report

Sorted By : Signal
Multiplier : 1.0000
Dilution : 1.0000
Use Multiplier & Dilution Factor with ISTDs

Signal 1: VWD1 A, Wavelength=254 nm

Peak #	RetTime [min]	Type	Width [min]	Area mAU *s	Height [mAU]	Area %
1	39.074	BB	1.1141	3689.96436	51.22665	49.7266
2	46.178	BB	1.3402	3730.53271	43.10681	50.2734

Totals : 7420.49707 94.33345

Sample Info : IK 7/3

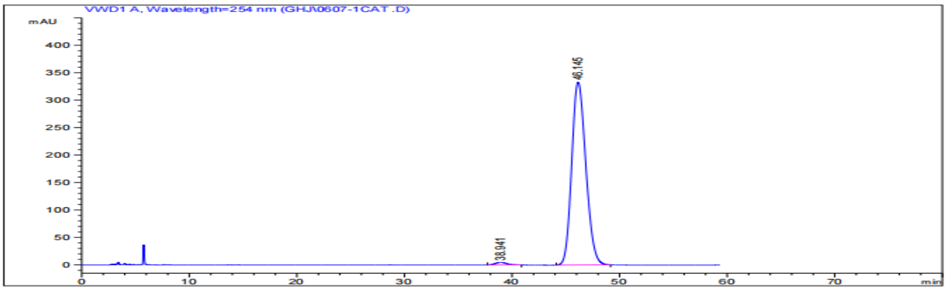

Area Percent Report

Sorted By : Signal
Multiplier : 1.0000
Dilution : 1.0000
Use Multiplier & Dilution Factor with ISTDs

Signal 1: VWD1 A, Wavelength=254 nm

Peak #	RetTime [min]	Type	Width [min]	Area mAU *s	Height [mAU]	Area %
1	38.941	BB	1.0938	352.47397	4.56848	1.1472
2	46.145	BB	1.4113	3.03722e4	332.79654	98.8528

Totals : 3.07246e4 337.36502

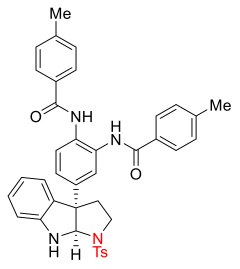

4l

Sample Info : IF 7/3

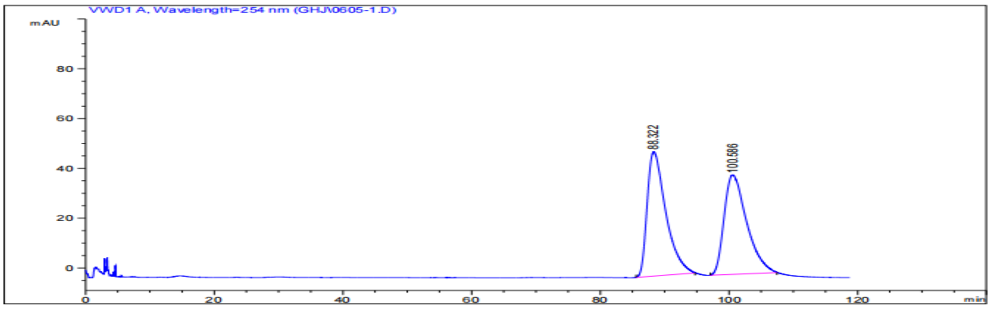


```
=====================================================================
                      Area Percent Report
=====================================================================

Sorted By             :      Signal
Multiplier            :      1.0000
Dilution              :      1.0000
Use Multiplier & Dilution Factor with ISTDs

Signal 1: VWD1 A, Wavelength=254 nm

Peak RetTime Type  Width     Area      Height     Area
  #   [min]        [min]  mAU    *s   [mAU   ]     %
----|-------|----|-------|----------|----------|--------|
   1  88.322 BB     2.8466 1.00713e4   49.90424  50.7764
   2 100.586 BB     3.1316 9763.27832  39.95802  49.2236

Totals :                   1.98345e4   89.86226
```

Sample Info : IF 7/3 1mL/min

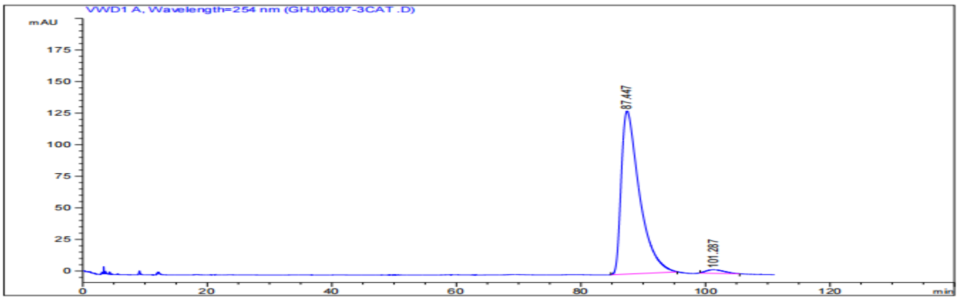


```
=====================================================================
                      Area Percent Report
=====================================================================

Sorted By             :      Signal
Multiplier            :      1.0000
Dilution              :      1.0000
Use Multiplier & Dilution Factor with ISTDs

Signal 1: VWD1 A, Wavelength=254 nm

Peak RetTime Type  Width     Area      Height     Area
  #   [min]        [min]  mAU    *s   [mAU   ]     %
----|-------|----|-------|----------|----------|--------|
   1  87.447 BB     2.7804 2.62601e4  128.96449  97.8065
   2 101.287 BB     2.3789  588.94305   2.89901   2.1935

Totals :                   2.68490e4  131.86350
```

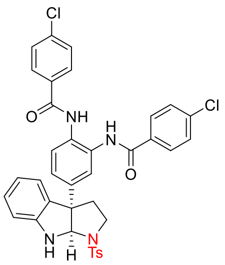

4m

Sample Info : IK 7/3

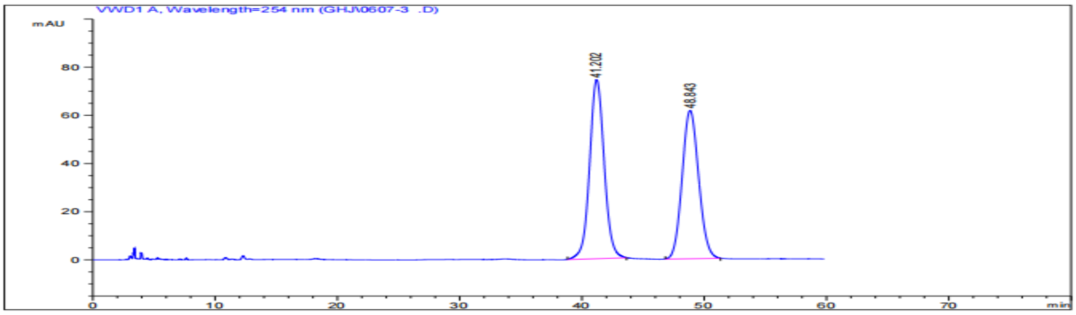


```
=====================================================================
                        Area Percent Report
=====================================================================

Sorted By             :      Signal
Multiplier            :      1.0000
Dilution              :      1.0000
Use Multiplier & Dilution Factor with ISTDs

Signal 1: VWD1 A, Wavelength=254 nm
```

Peak #	RetTime [min]	Type	Width [min]	Area mAU *s	Height [mAU]	Area %
1	41.202	BB	1.2730	6151.54004	74.30893	51.5323
2	48.843	BB	1.4514	5785.70508	61.58222	48.4677
Totals :				1.19372e4	135.89115	

Sample Info : IK 7/3

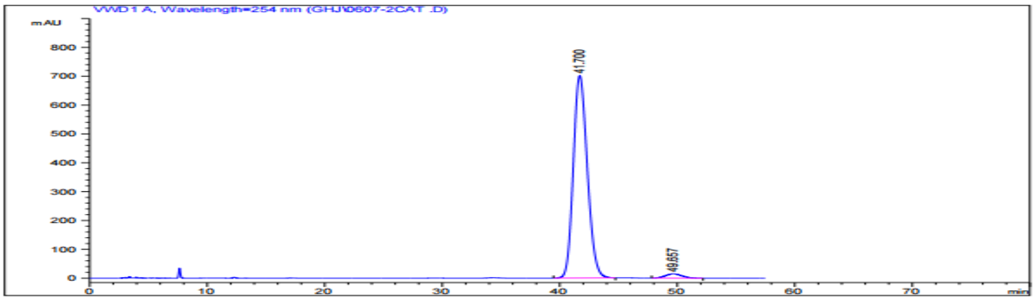


```
=====================================================================
                        Area Percent Report
=====================================================================

Sorted By             :      Signal
Multiplier            :      1.0000
Dilution              :      1.0000
Use Multiplier & Dilution Factor with ISTDs

Signal 1: VWD1 A, Wavelength=254 nm
```

Peak #	RetTime [min]	Type	Width [min]	Area mAU *s	Height [mAU]	Area %
1	41.700	BB	1.2949	5.80084e4	700.73273	97.5867
2	49.657	BB	1.4244	1434.54919	14.74987	2.4133
Totals :				5.94429e4	715.48259	

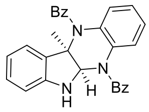

5a

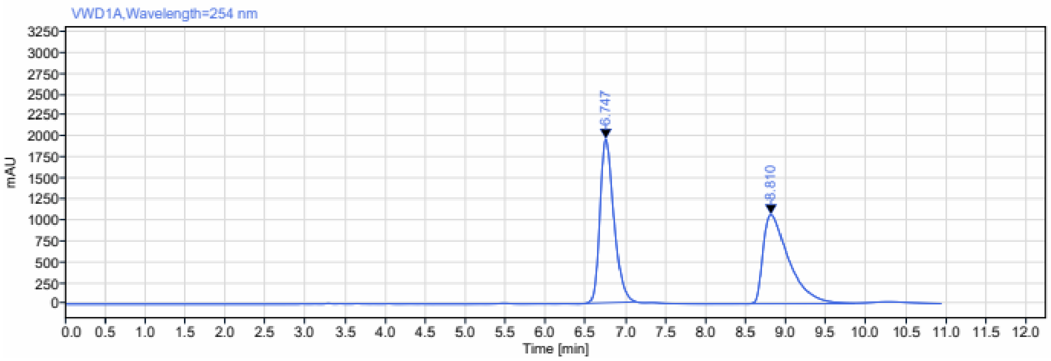

Signal:	VWD1A,Wavelength=254 nm					
**RT [min]**	**Type**	**Width [min]**	**Area**	**Height**	**Area%**	**Name**
6.747	BM m	0.75	23893.50	1951.31	49.86	
8.810	BV	1.35	24032.40	1062.40	50.14	
		**Sum**	**47925.90**			

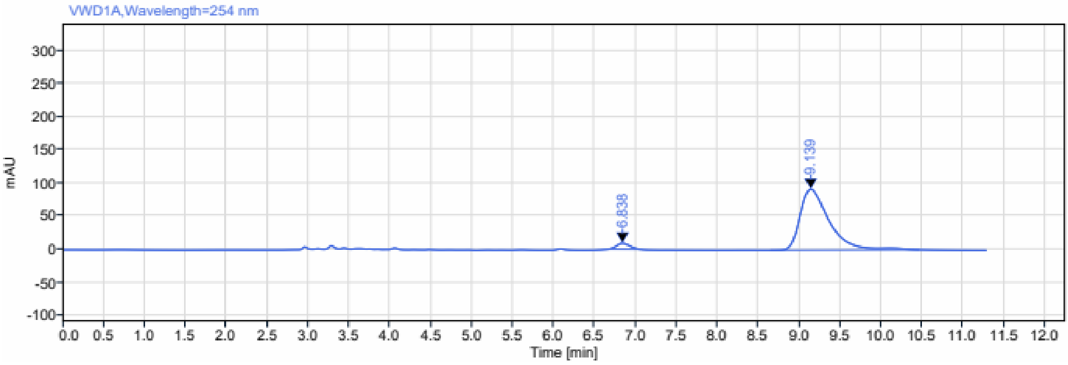

Signal:	VWD1A,Wavelength=254 nm					
**RT [min]**	**Type**	**Width [min]**	**Area**	**Height**	**Area%**	**Name**
6.838	MM m	0.37	96.04	8.69	4.07	
9.139	MM m	1.82	2261.78	91.71	95.93	
		**Sum**	**2357.82**			

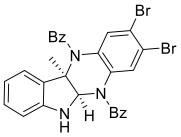

5b

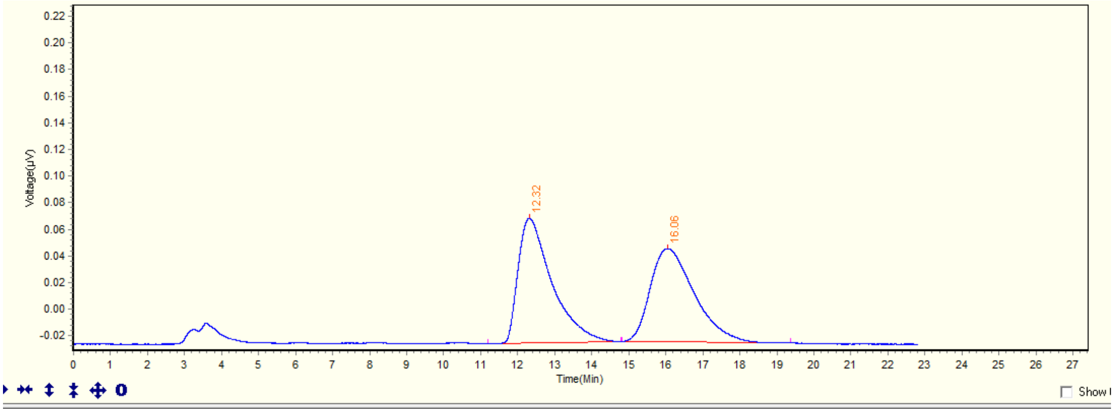

No.	Retention Time	Peak Area	Peak Height	Peak Area(%)	Peak Width	Peak Type
1	12.32	2956450	46844	50.63%	3.594	BB
2	16.06	2882629	35041	49.37%	4.554	BB
otal		5,839,079	81,885	100.00%		

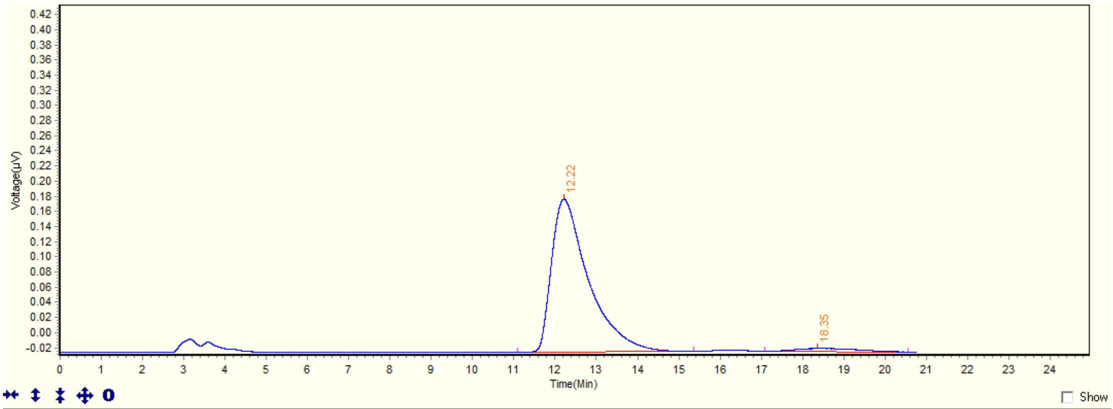

No.	Retention Time	Peak Area	Peak Height	Peak Area(%)	Peak Width	Peak Type
1	12.22	6296416	100952	96.77%	4.271	BB
2	18.35	210454	1994	3.23%	3.472	BB
il		6,506,870	102,946	100.00%		

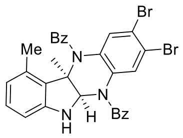

5c

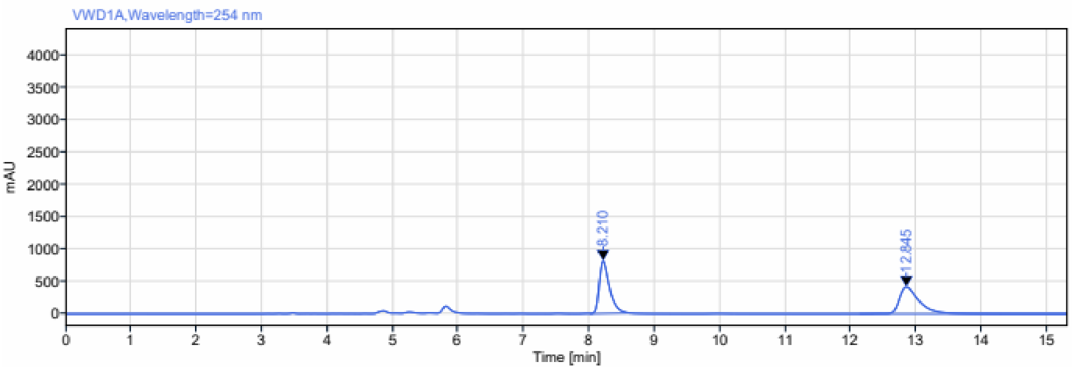

Signal: VWD1A,Wavelength=254 nm

RT [min]	Type	Width [min]	Area	Height	Area%	Name
8.210	VM m	0.61	8884.75	809.75	50.52	
12.845	BB	4.52	8703.39	411.68	49.48	
		**Sum**	**17588.14**			

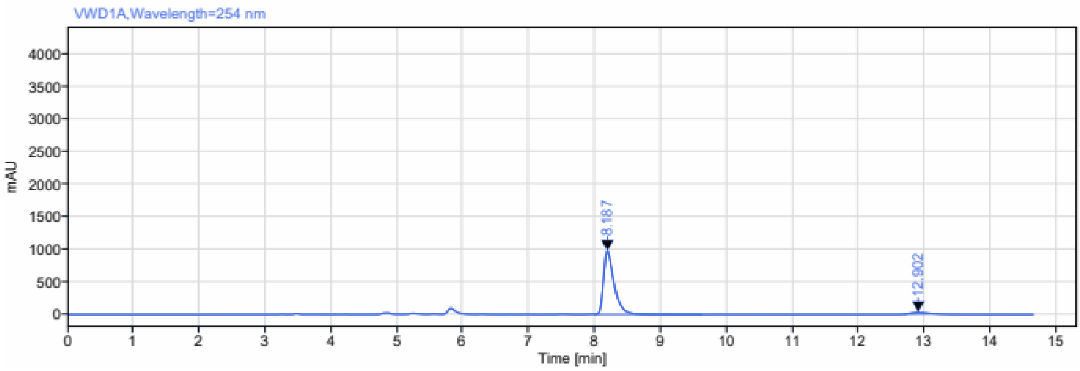

Signal: VWD1A,Wavelength=254 nm

RT [min]	Type	Width [min]	Area	Height	Area%	Name
8.187	VB	1.63	10925.81	973.13	96.59	
12.902	MM m	0.46	385.29	25.52	3.41	
		**Sum**	**11311.10**			

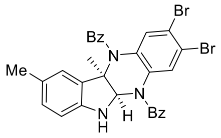

5d

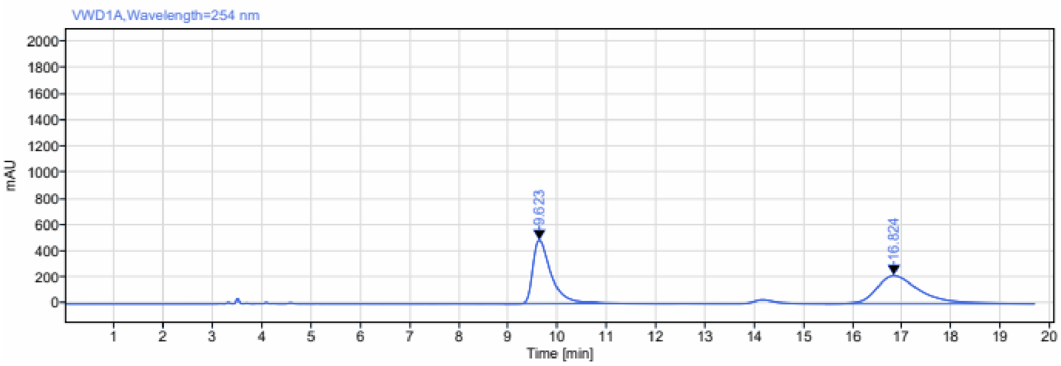

Signal: VWD1A,Wavelength=254 nm

RT [min]	Type	Width [min]	Area	Height	Area%	Name
9.623	BM m	2.33	12466.33	482.01	49.23	
16.824	BBA	4.26	12854.63	212.57	50.77	
		**Sum**	**25320.96**			

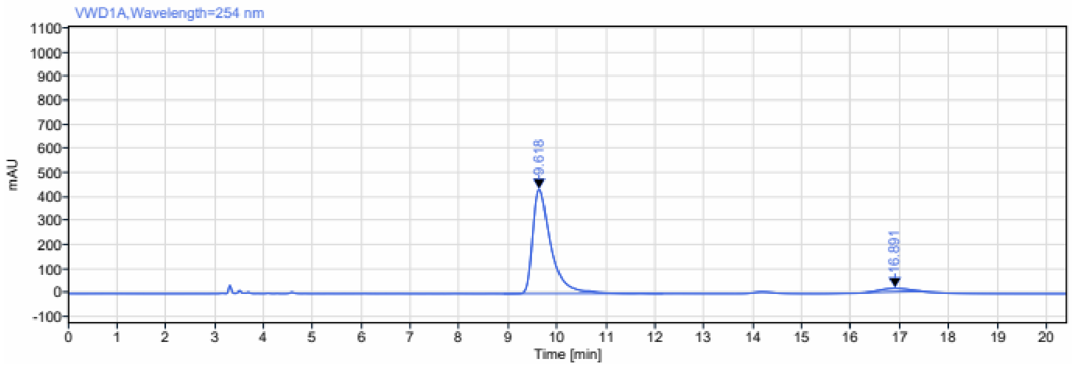

Signal: VWD1A,Wavelength=254 nm

RT [min]	Type	Width [min]	Area	Height	Area%	Name
9.618	BM m	3.26	11226.65	431.97	95.48	
16.891	MM m	1.12	531.25	13.80	4.52	
		**Sum**	**11757.89**			

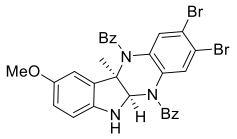

5e

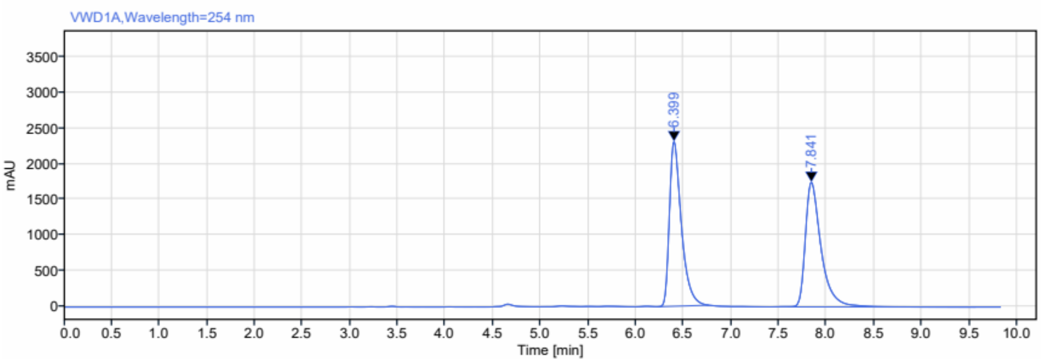

Signal:	VWD1A,Wavelength=254 nm					
**RT [min]**	**Type**	**Width [min]**	**Area**	**Height**	**Area%**	**Name**
6.399	VM m	0.54	20027.67	2298.25	49.98	
7.841	VBA	1.97	20046.97	1739.67	50.02	
		**Sum**	**40074.63**			

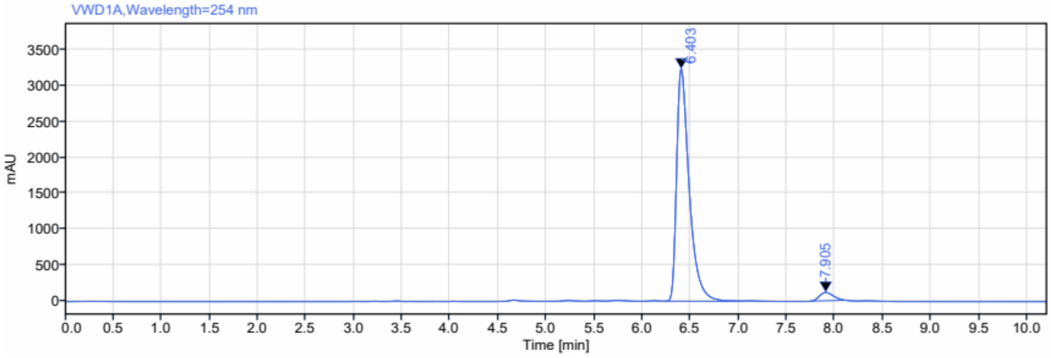

Signal:	VWD1A,Wavelength=254 nm					
**RT [min]**	**Type**	**Width [min]**	**Area**	**Height**	**Area%**	**Name**
6.403	BV	0.80	29404.53	3223.08	96.20	
7.905	MM m	0.36	1162.69	115.64	3.80	
		**Sum**	**30567.22**			

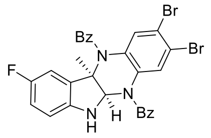

5f

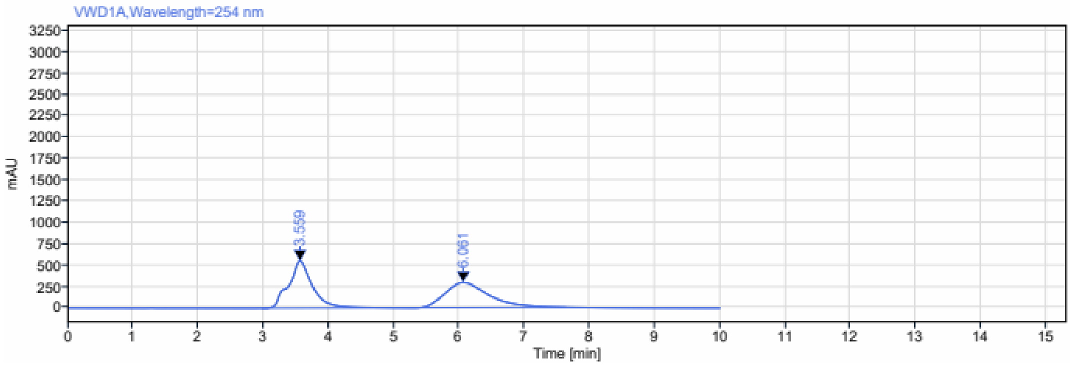

Signal: VWD1A,Wavelength=254 nm

RT [min]	Type	Width [min]	Area	Height	Area%	Name
3.559	VM m	2.33	14009.43	552.33	50.41	
6.061	MM m	2.33	13782.45	294.11	49.59	
		**Sum**	**27791.88**			

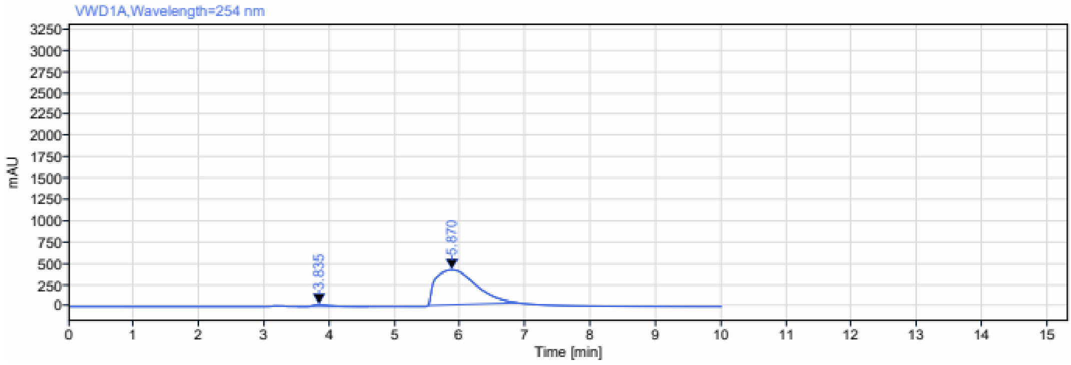

Signal: VWD1A,Wavelength=254 nm

RT [min]	Type	Width [min]	Area	Height	Area%	Name
3.835	MM m	1.09	401.70	23.53	2.25	
5.870	MM m	1.37	17468.52	410.10	97.75	
		**Sum**	**17870.22**			

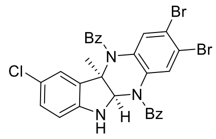

5g

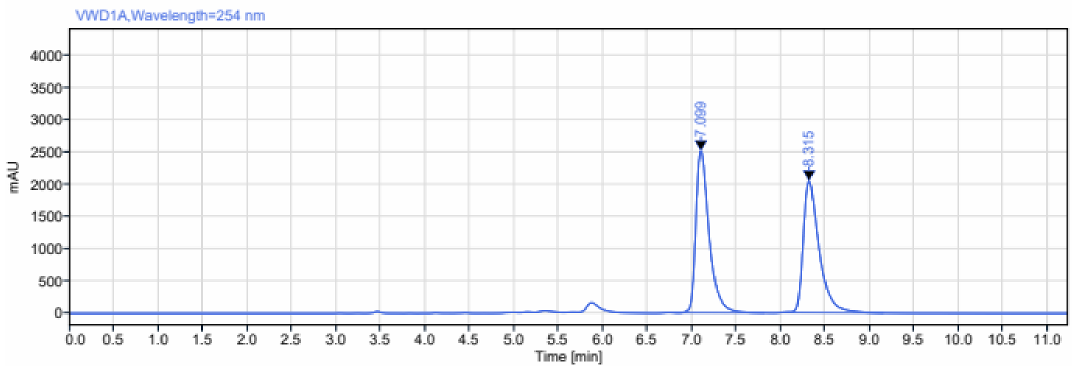

Signal: VWD1A,Wavelength=254 nm

RT [min]	Type	Width [min]	Area	Height	Area%	Name
7.099	MM m	1.02	25908.26	2504.88	50.36	
8.315	MM m	1.16	25535.46	2035.24	49.64	
		**Sum**	**51443.71**			

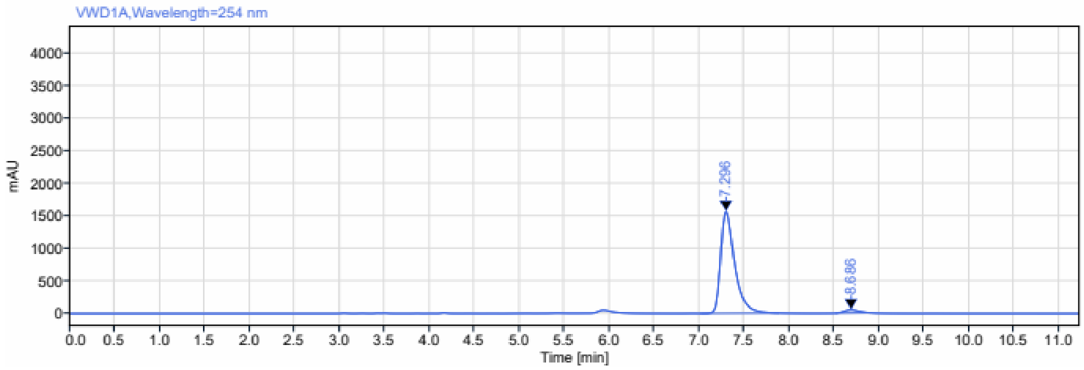

Signal: VWD1A,Wavelength=254 nm

RT [min]	Type	Width [min]	Area	Height	Area%	Name
7.296	MM m	0.87	16999.93	1561.69	96.98	
8.686	MM m	0.38	529.54	47.78	3.02	
		**Sum**	**17529.47**			

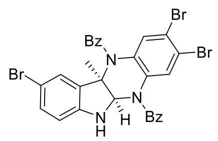

5h

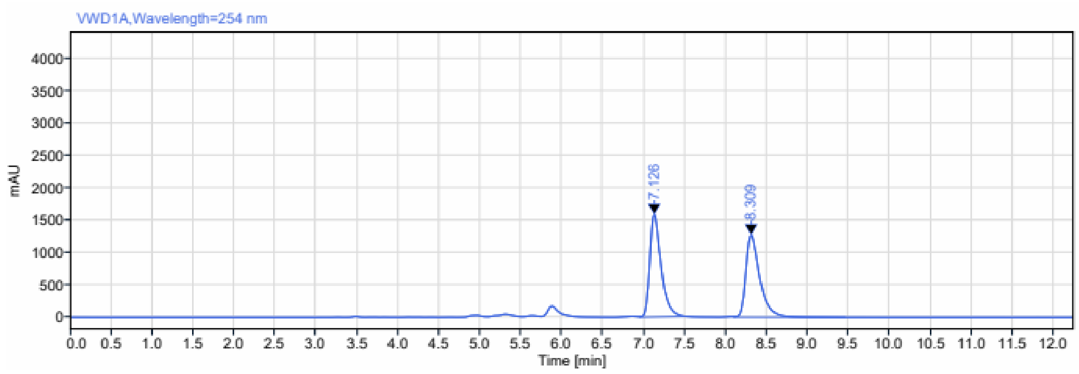

Signal: VWD1A,Wavelength=254 nm

RT [min]	Type	Width [min]	Area	Height	Area%	Name
7.126	VM m	0.57	15264.62	1580.28	50.32	
8.309	VB	1.37	15070.51	1267.93	49.68	
		**Sum**	**30335.13**			

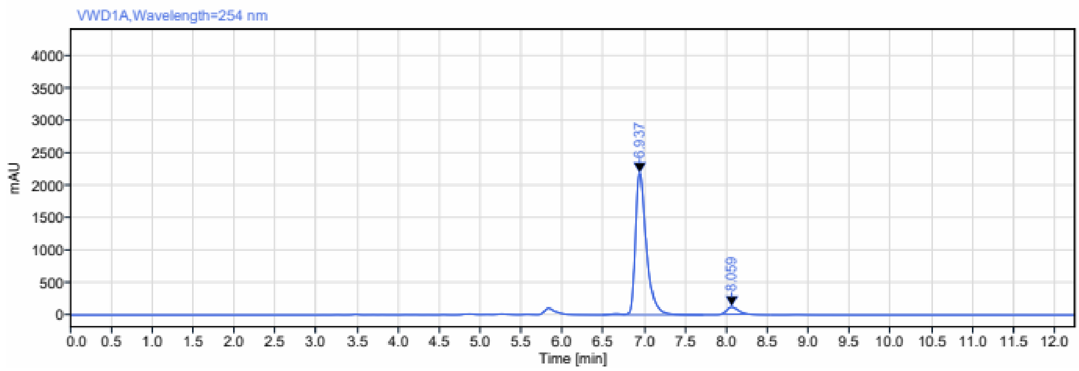

Signal: VWD1A,Wavelength=254 nm

RT [min]	Type	Width [min]	Area	Height	Area%	Name
6.937	MV m	1.22	20420.57	2181.28	94.98	
8.059	MM m	0.35	1079.38	111.97	5.02	
		**Sum**	**21499.96**			

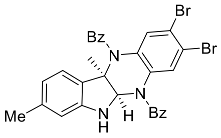

5i

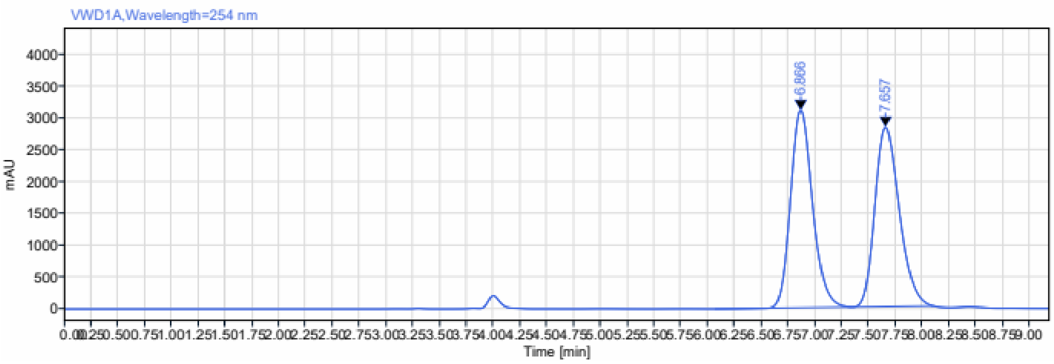

Signal: VWD1A,Wavelength=254 nm

RT [min]	Type	Width [min]	Area	Height	Area%	Name
6.866	BM m	1.28	44204.50	3099.63	49.70	
7.657	MM m	0.76	44738.80	2817.44	50.30	
		**Sum**	**88943.30**			

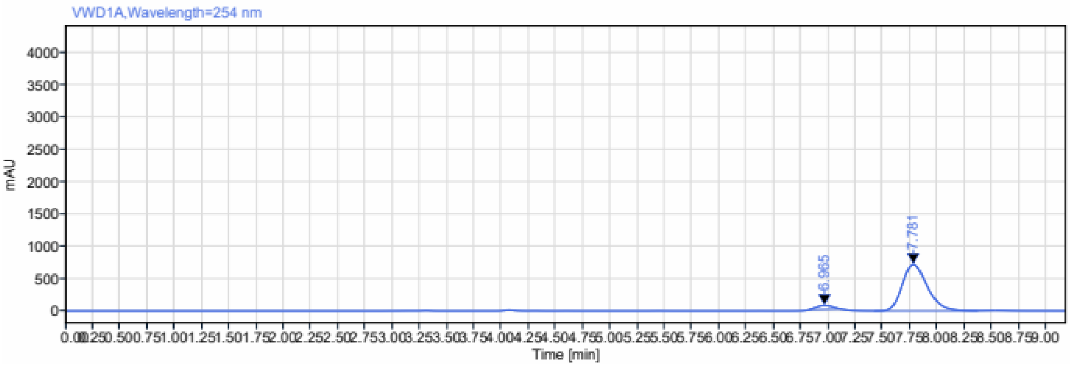

Signal: VWD1A,Wavelength=254 nm

RT [min]	Type	Width [min]	Area	Height	Area%	Name
6.965	MM m	0.29	635.13	62.61	5.17	
7.781	VV	0.95	11647.98	721.31	94.83	
		**Sum**	**12283.10**			

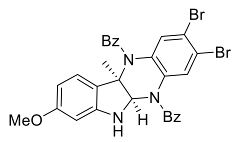

5j

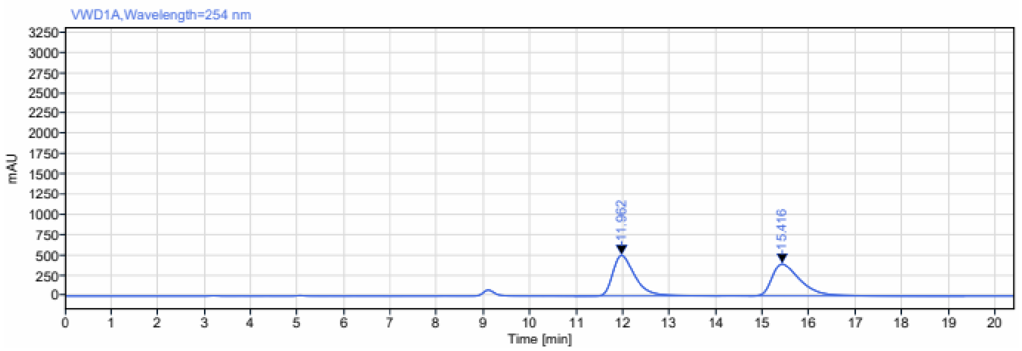

Signal: VWD1A,Wavelength=254 nm

RT [min]	Type	Width [min]	Area	Height	Area%	Name
11.962	BB	3.15	16337.74	500.43	49.71	
15.416	BB	4.64	16529.51	393.72	50.29	
		**Sum**	**32867.25**			

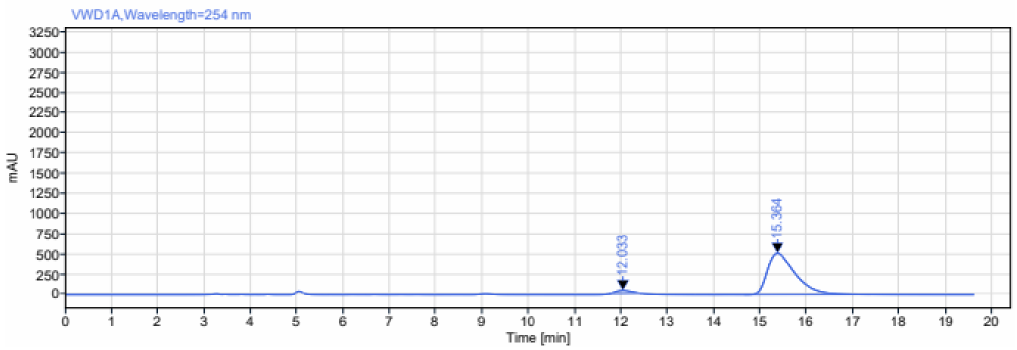

Signal: VWD1A,Wavelength=254 nm

RT [min]	Type	Width [min]	Area	Height	Area%	Name
12.033	MM m	0.71	954.17	39.72	4.25	
15.364	BM m	2.73	21516.75	513.03	95.75	
		**Sum**	**22470.92**			

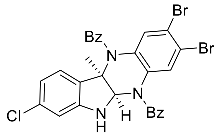

5k

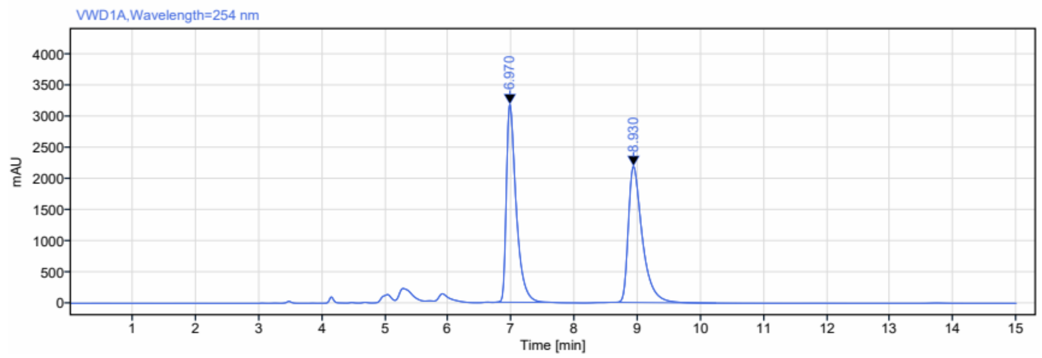

Signal: VWD1A,Wavelength=254 nm

RT [min]	Type	Width [min]	Area	Height	Area%	Name
6.970	MM m	1.03	34241.97	3171.11	50.45	
8.930	MM m	1.62	33630.27	2182.89	49.55	
		**Sum**	**67872.24**			

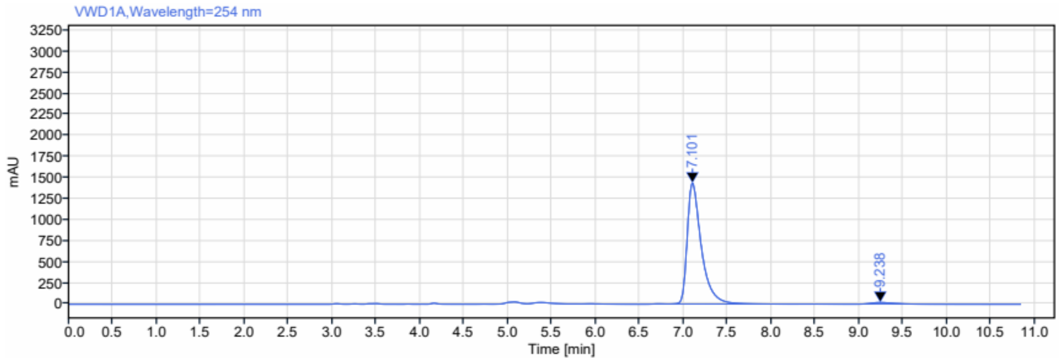

Signal: VWD1A,Wavelength=254 nm

RT [min]	Type	Width [min]	Area	Height	Area%	Name
7.101	MM m	1.36	15987.21	1433.67	98.52	
9.238	MM m	0.45	239.91	17.07	1.48	
		**Sum**	**16227.12**			

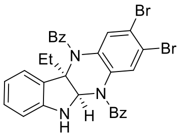

5l

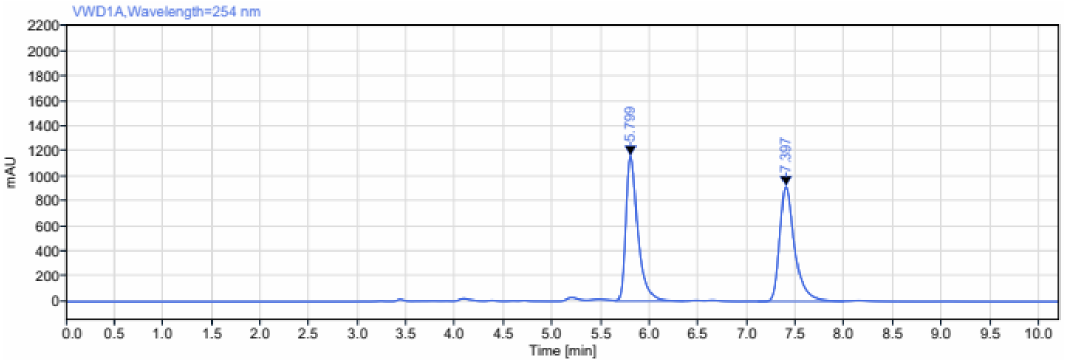

Signal: VWD1A,Wavelength=254 nm

RT [min]	Type	Width [min]	Area	Height	Area%	Name
5.799	VB	0.71	9914.45	1155.20	50.18	
7.397	BV	0.89	9842.37	915.43	49.82	
		**Sum**	**19756.82**			

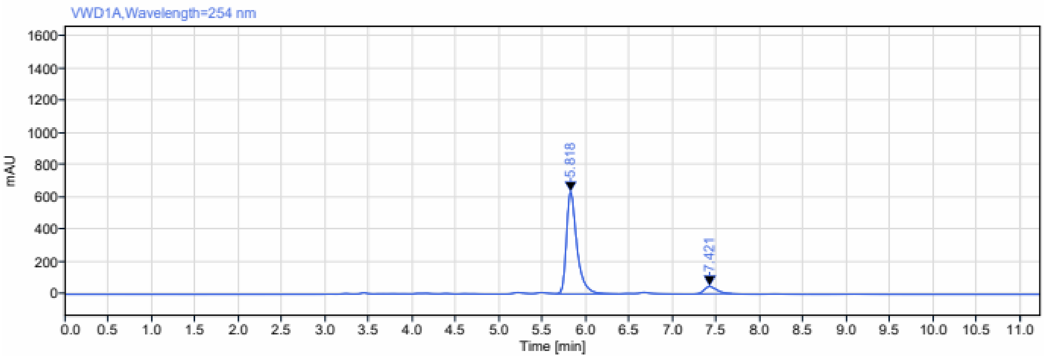

Signal: VWD1A,Wavelength=254 nm

RT [min]	Type	Width [min]	Area	Height	Area%	Name
5.818	VB	0.71	5378.11	630.24	92.01	
7.421	MM m	0.43	467.21	46.03	7.99	
		**Sum**	**5845.32**			

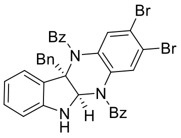

5m

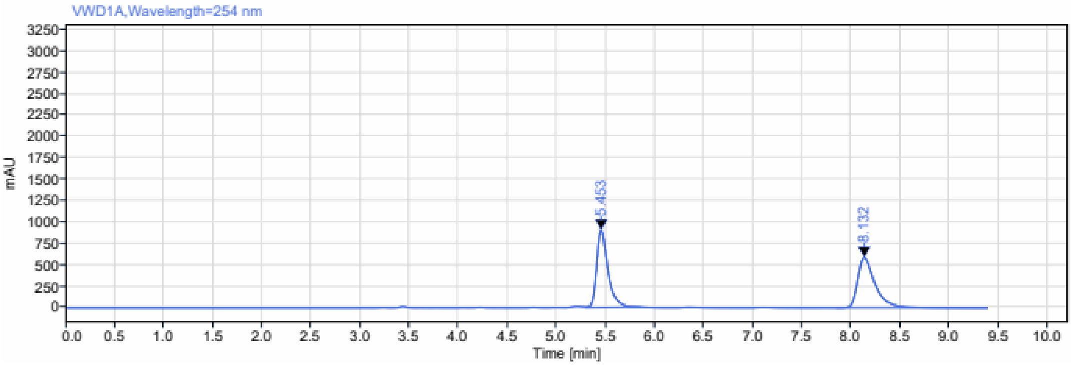

Signal: VWD1A,Wavelength=254 nm

RT [min]	Type	Width [min]	Area	Height	Area%	Name
5.453	VM m	0.57	7291.75	902.64	50.24	
8.132	VBA	1.54	7222.08	586.36	49.76	
		**Sum**	**14513.83**			

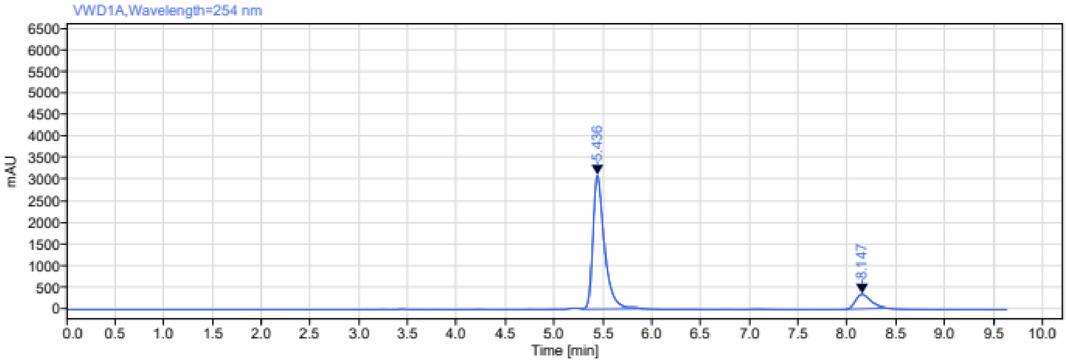

Signal: VWD1A,Wavelength=254 nm

RT [min]	Type	Width [min]	Area	Height	Area%	Name
5.436	VM m	0.64	25571.39	3088.46	87.69	
8.147	MM m	0.43	3591.07	330.49	12.31	
		**Sum**	**29162.46**			

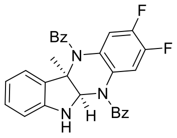

5n

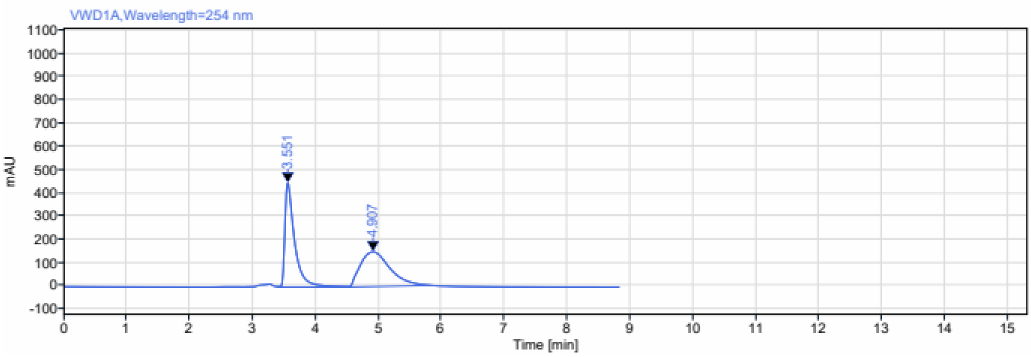

Signal: VWD1A,Wavelength=254 nm

RT [min]	Type	Width [min]	Area	Height	Area%	Name
3.551	VV	1.13	4848.65	448.26	49.67	
4.907	MM m	1.31	4912.57	149.90	50.33	
		**Sum**	**9761.22**			

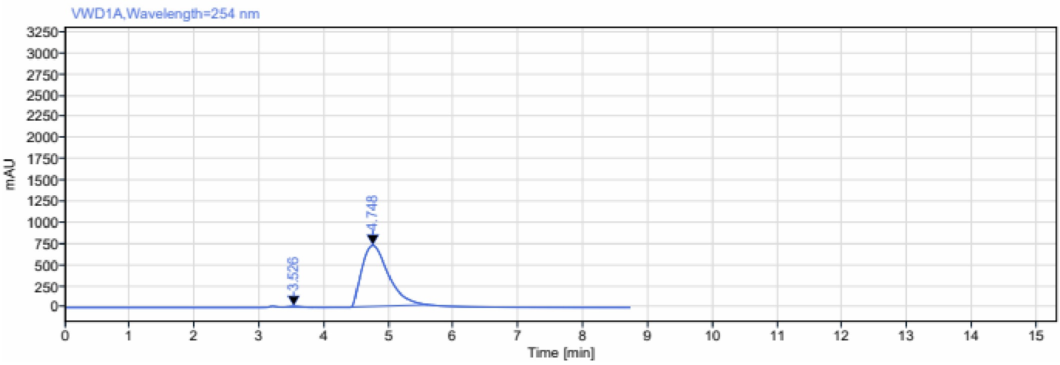

Signal: VWD1A,Wavelength=254 nm

RT [min]	Type	Width [min]	Area	Height	Area%	Name
3.526	MM m	0.48	129.85	11.11	0.62	
4.748	MM m	1.18	20655.61	720.98	99.38	
		**Sum**	**20785.46**			

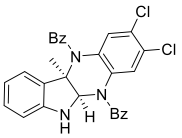

5o

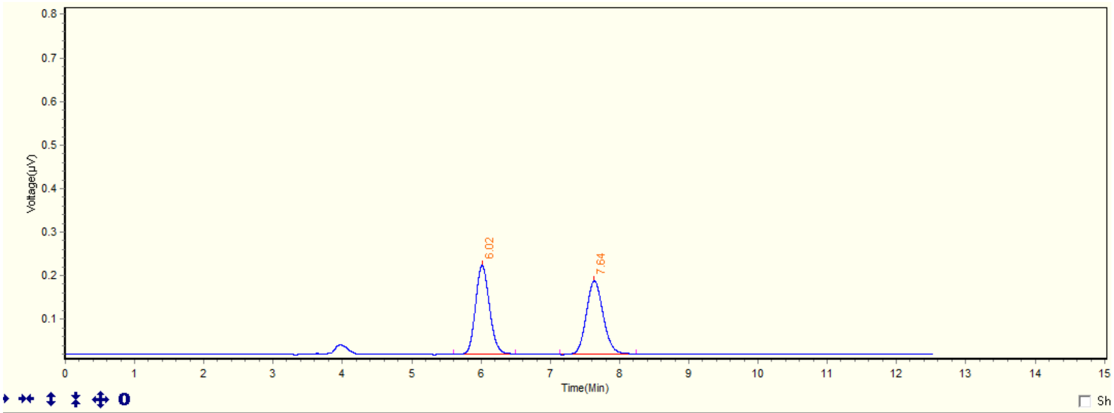

ntegration Result | Calculation Result | TimeTable

No.	Retention Time	Peak Area	Peak Height	Peak Area(%)	Peak Width	Peak Type
1	6.02	1401472	101197	49.62%	0.894	BB
2	7.64	1423155	84244	50.38%	1.103	BB
otal		2,824,627	185,441	100.00%		

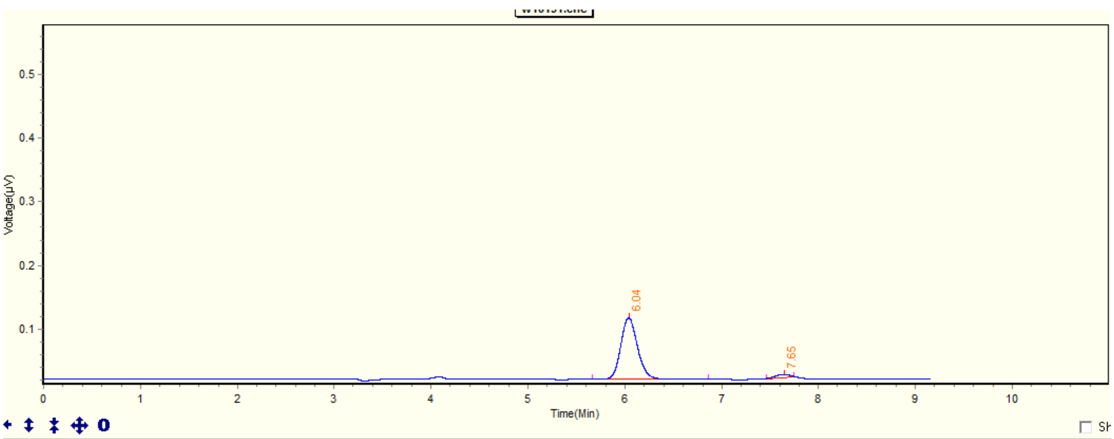

ration Result | Calculation Result | TimeTable

o.	Retention Time	Peak Area	Peak Height	Peak Area(%)	Peak Width	Peak Type
1	6.04	576642	48331	96.67%	1.194	BB
2	7.65	19845	1978	3.33%	0.285	BB
		596,487	50,309	100.00%		

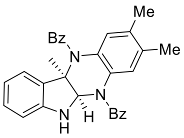

5p

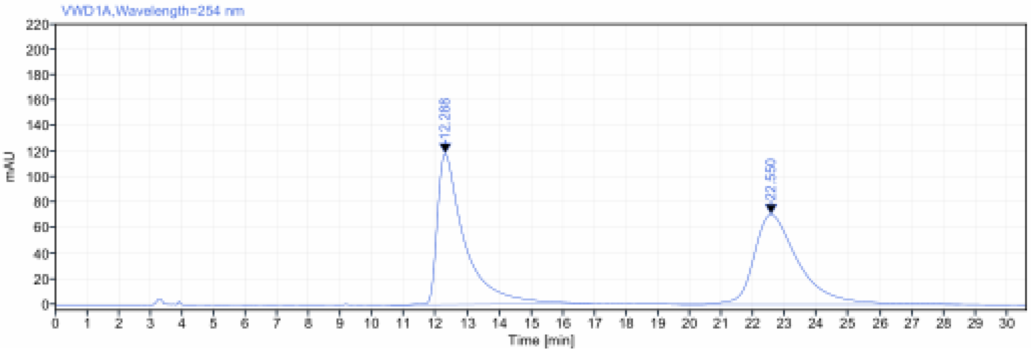

Signal: VWD1A,Wavelength=254 nm

RT [min]	Type	Width [min]	Area	Height	Area%	Name
12.288	MM m	6.31	7032.57	117.87	50.68	
22.550	MM m	8.90	6844.00	70.08	49.32	
		**Sum**	**13876.57**			

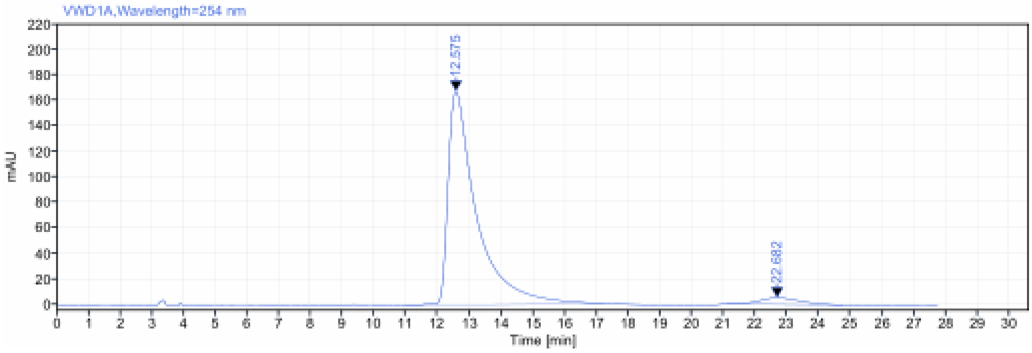

Signal: VWD1A,Wavelength=254 nm

RT [min]	Type	Width [min]	Area	Height	Area%	Name
12.575	MM m	6.38	10393.38	167.31	95.82	
22.682	MM m	3.66	453.85	5.06	4.18	
		**Sum**	**10847.23**			

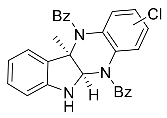

5q-up

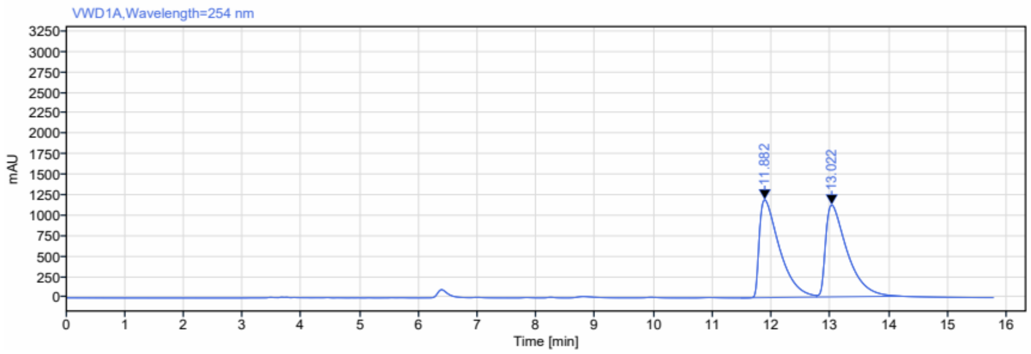

Signal: VWD1A,Wavelength=254 nm

RT [min]	Type	Width [min]	Area	Height	Area%	Name
11.882	BM m	1.29	28556.64	1192.97	49.91	
13.022	MM m	1.43	28655.21	1121.52	50.09	
		**Sum**	**57211.85**			

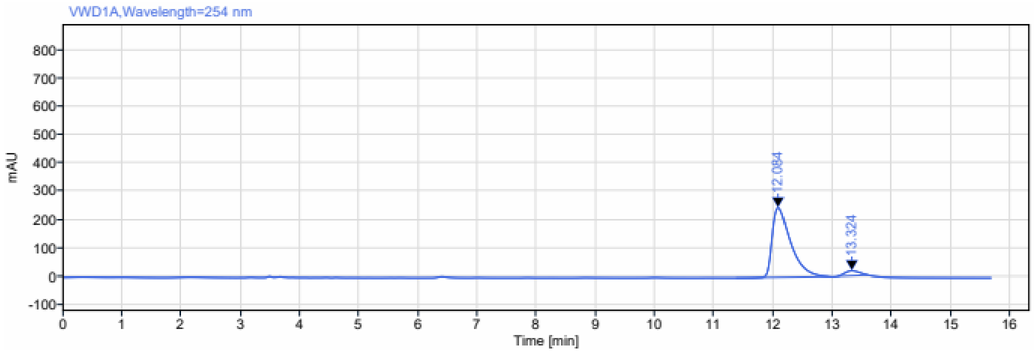

Signal: VWD1A,Wavelength=254 nm

RT [min]	Type	Width [min]	Area	Height	Area%	Name
12.084	BM m	1.56	5194.22	244.75	94.81	
13.324	MM m	0.51	284.29	16.66	5.19	
		**Sum**	**5478.52**			

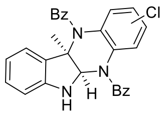

5q-down

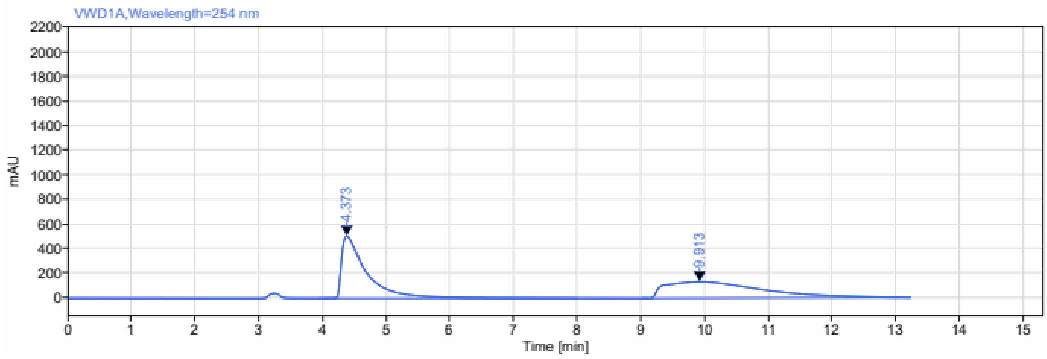

Signal: VWD1A,Wavelength=254 nm

RT [min]	Type	Width [min]	Area	Height	Area%	Name
4.373	BB	4.06	13819.49	505.94	50.03	
9.913	BM m	4.01	13801.54	133.07	49.97	
		**Sum**	**27621.03**			

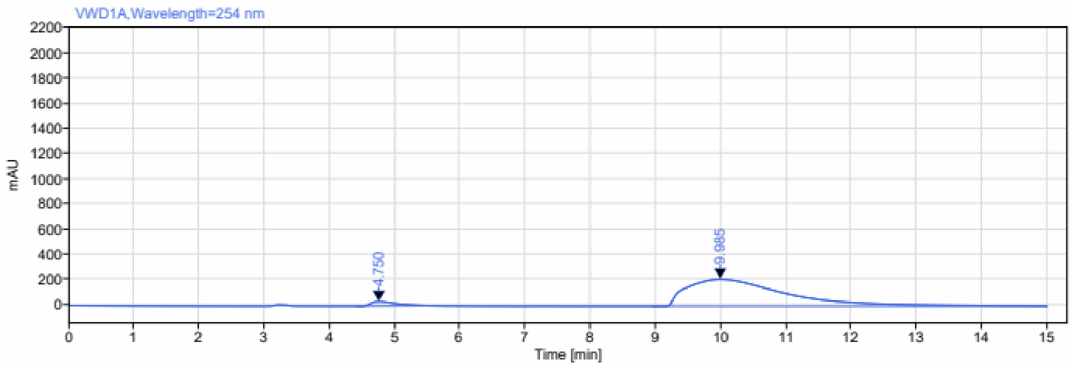

Signal: VWD1A,Wavelength=254 nm

RT [min]	Type	Width [min]	Area	Height	Area%	Name
4.750	MM m	1.16	964.46	35.91	4.04	
9.985	BBA	6.06	22909.04	214.48	95.96	
		**Sum**	**23873.50**			

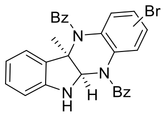

5r-up

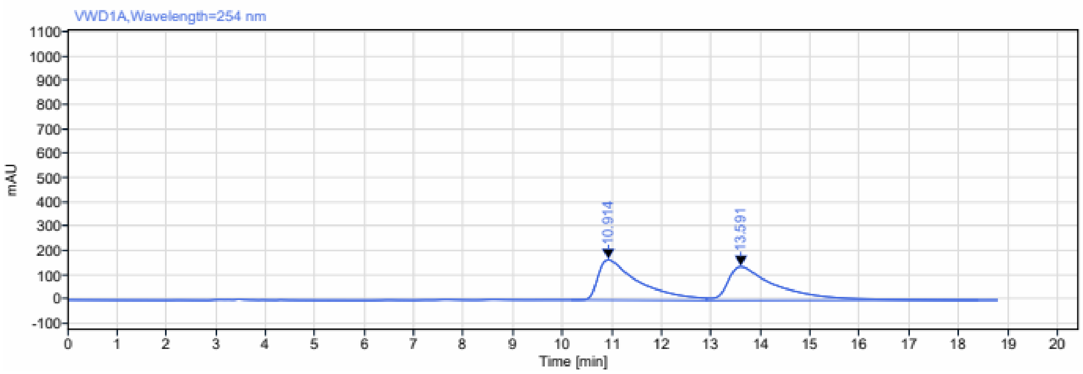

Signal: VWD1A,Wavelength=254 nm

RT [min]	Type	Width [min]	Area	Height	Area%	Name
10.914	BM m	2.73	9447.32	165.89	49.85	
13.591	MB m	5.48	9504.05	138.81	50.15	
		**Sum**	**18951.37**			

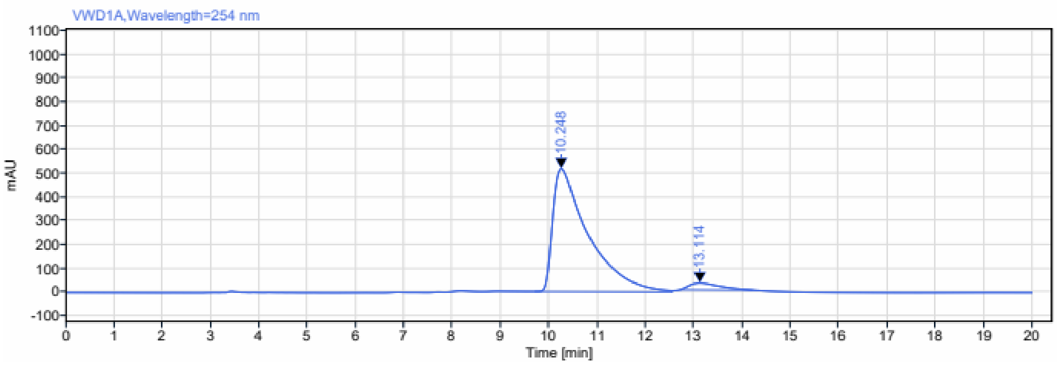

Signal: VWD1A,Wavelength=254 nm

RT [min]	Type	Width [min]	Area	Height	Area%	Name
10.248	BV	2.86	26360.99	516.12	95.51	
13.114	MM m	1.52	1238.82	28.30	4.49	
		**Sum**	**27599.81**			

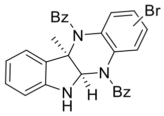

5r-down

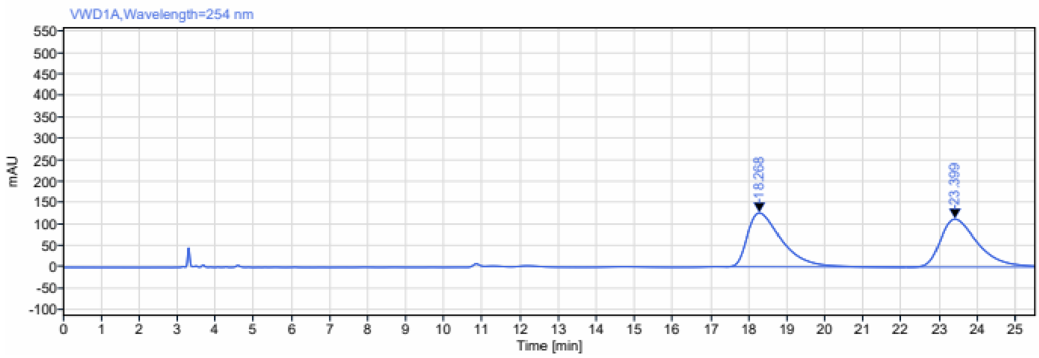

Signal: VWD1A,Wavelength=254 nm

RT [min]	Type	Width [min]	Area	Height	Area%	Name
18.268	BB	4.73	7858.15	125.77	50.70	
23.399	BBA	3.87	7642.38	111.83	49.30	
		**Sum**	**15500.53**			

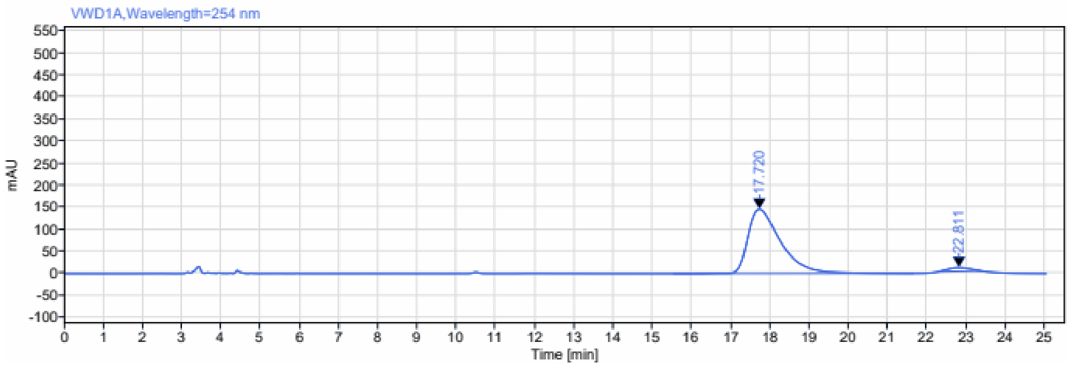

Signal: VWD1A,Wavelength=254 nm

RT [min]	Type	Width [min]	Area	Height	Area%	Name
17.720	BB	6.15	8448.01	145.44	96.49	
22.811	MM m	1.08	307.16	7.95	3.51	
		**Sum**	**8755.17**			

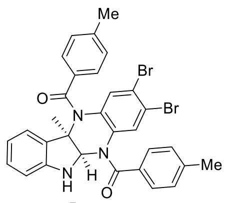

5s

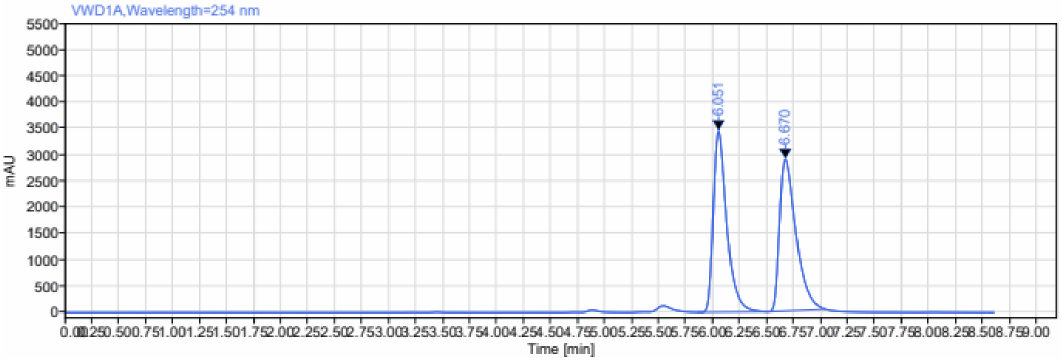

Signal: VWD1A,Wavelength=254 nm

RT [min]	Type	Width [min]	Area	Height	Area%	Name
6.051	MM m	0.66	30059.48	3448.21	49.52	
6.670	MM m	0.53	30646.18	2887.50	50.48	
		**Sum**	**60705.66**			

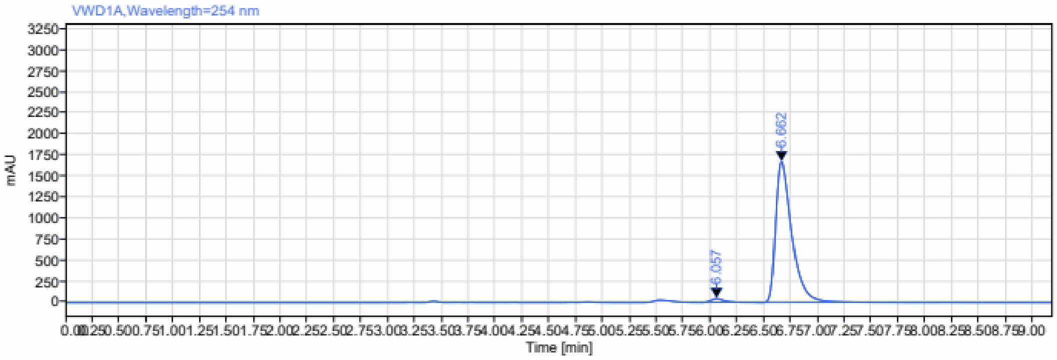

Signal: VWD1A,Wavelength=254 nm

RT [min]	Type	Width [min]	Area	Height	Area%	Name
6.057	MM m	0.34	359.84	44.84	2.00	
6.662	MM m	0.97	17588.61	1670.04	98.00	
		**Sum**	**17948.45**			

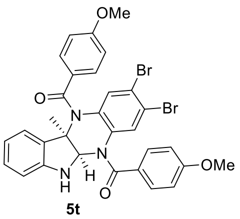

5t

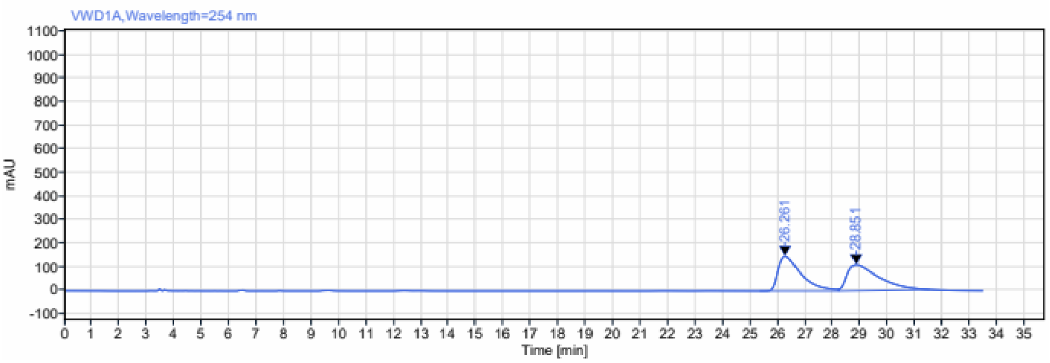

Signal: VWD1A,Wavelength=254 nm

RT [min]	Type	Width [min]	Area	Height	Area%	Name
26.261	MM m	2.87	8687.63	146.78	49.54	
28.851	MM m	3.31	8849.57	109.14	50.46	
		**Sum**	**17537.20**			

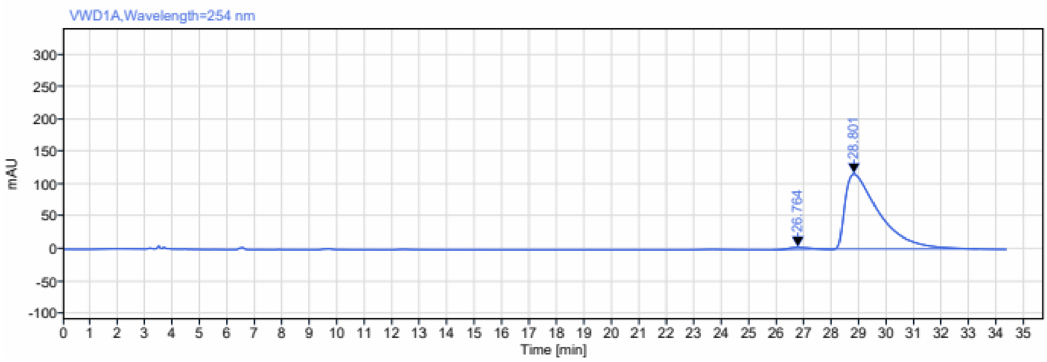

Signal: VWD1A,Wavelength=254 nm

RT [min]	Type	Width [min]	Area	Height	Area%	Name
26.764	MM m	1.76	151.68	3.14	1.50	
28.801	MM m	5.70	9950.37	115.71	98.50	
		**Sum**	**10102.05**			

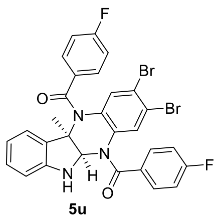

5u

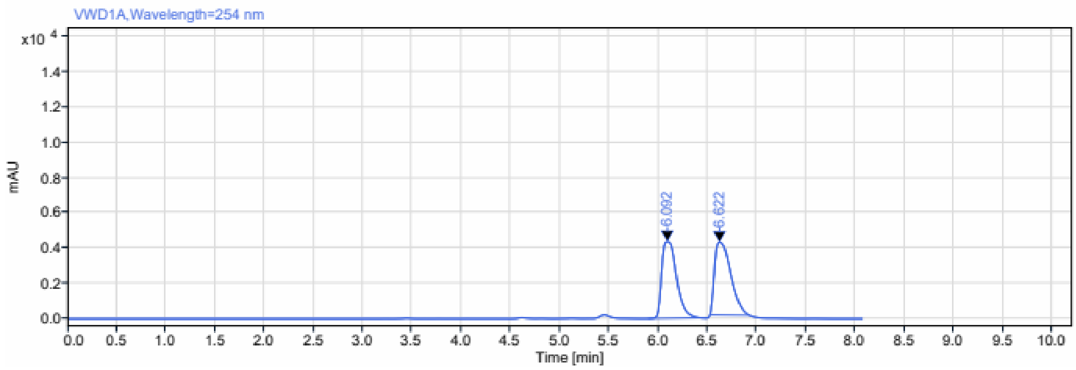

Signal: VWD1A,Wavelength=254 nm

RT [min]	Type	Width [min]	Area	Height	Area%	Name
6.092	MM m	0.61	45398.53	4345.88	49.10	
6.622	MM m	0.40	47069.14	4114.87	50.90	
		**Sum**	**92467.67**			

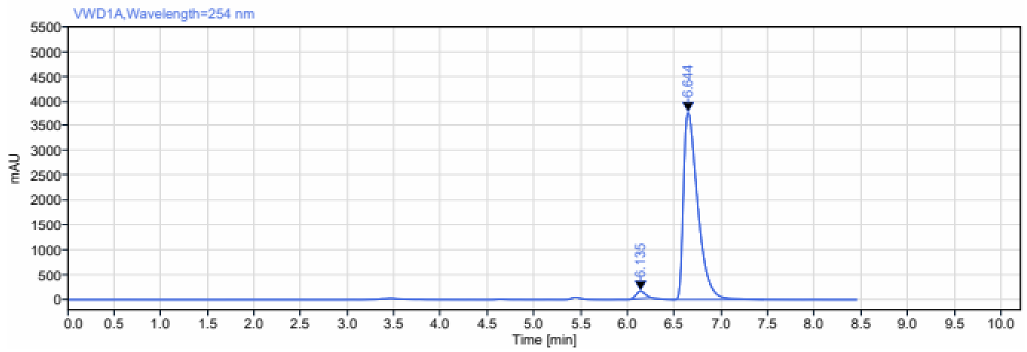

Signal: VWD1A,Wavelength=254 nm

RT [min]	Type	Width [min]	Area	Height	Area%	Name
6.135	MM m	0.23	993.65	147.20	2.45	
6.644	MM m	0.99	39496.30	3782.78	97.55	
		**Sum**	**40489.95**			

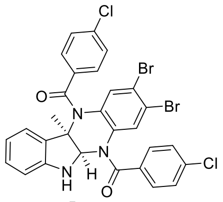

5v

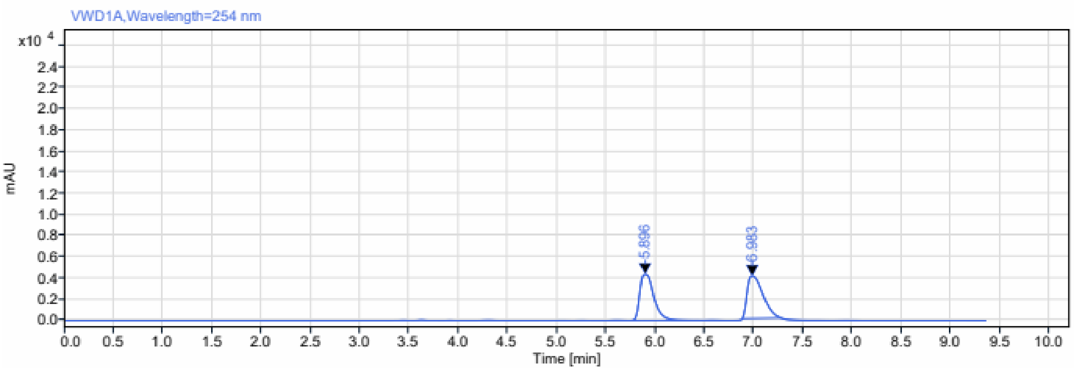

Signal: VWD1A,Wavelength=254 nm

RT [min]	Type	Width [min]	Area	Height	Area%	Name
5.896	MM m	0.93	43487.79	4364.28	49.35	
6.983	MM m	0.39	44636.17	4006.58	50.65	
		**Sum**	**88123.96**			

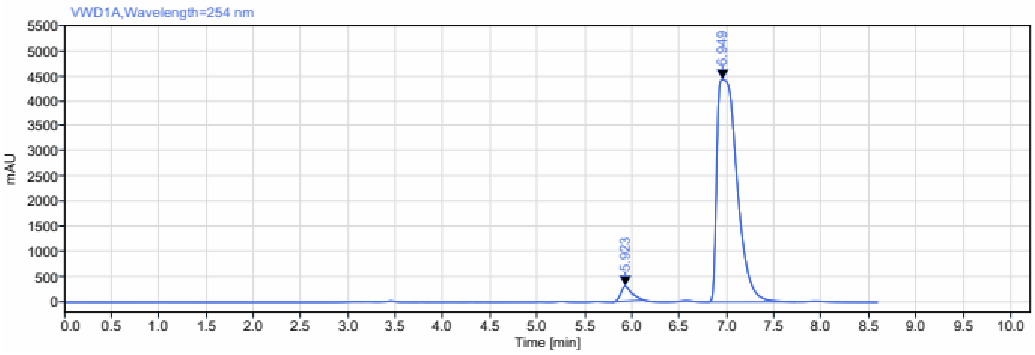

Signal: VWD1A,Wavelength=254 nm

RT [min]	Type	Width [min]	Area	Height	Area%	Name
5.923	MM m	0.33	2402.86	294.97	3.53	
6.949	MM m	0.93	65600.09	4427.09	96.47	
		**Sum**	**68002.95**			

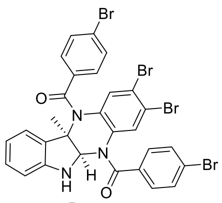

5w

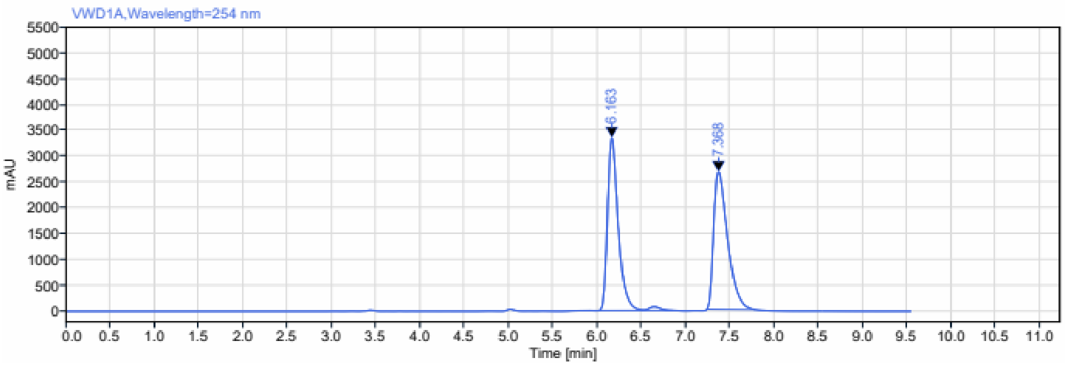

Signal: VWD1A,Wavelength=254 nm

RT [min]	Type	Width [min]	Area	Height	Area%	Name
6.163	MM m	0.94	28847.89	3347.53	49.69	
7.368	MM m	0.55	29207.51	2663.84	50.31	
		**Sum**	**58055.40**			

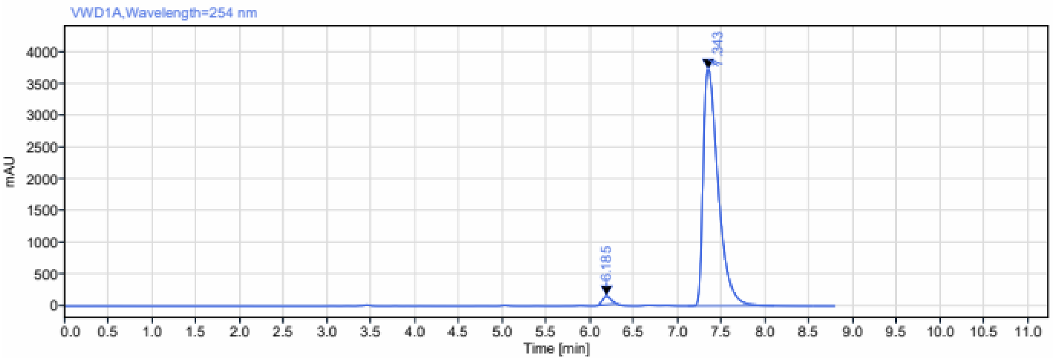

Signal: VWD1A,Wavelength=254 nm

RT [min]	Type	Width [min]	Area	Height	Area%	Name
6.185	MM m	0.22	865.05	129.71	1.94	
7.343	MM m	0.99	43638.53	3722.59	98.06	
		**Sum**	**44503.58**			

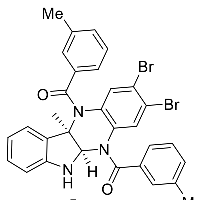

5x

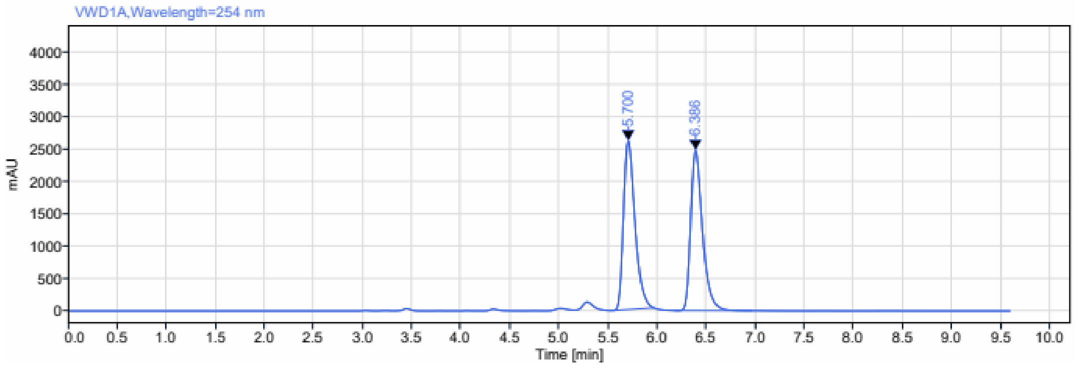

Signal: VWD1A,Wavelength=254 nm

RT [min]	Type	Width [min]	Area	Height	Area%	Name
5.700	MM m	0.40	21132.05	2600.86	50.50	
6.386	MM m	0.79	20716.92	2472.18	49.50	
		**Sum**	**41848.96**			

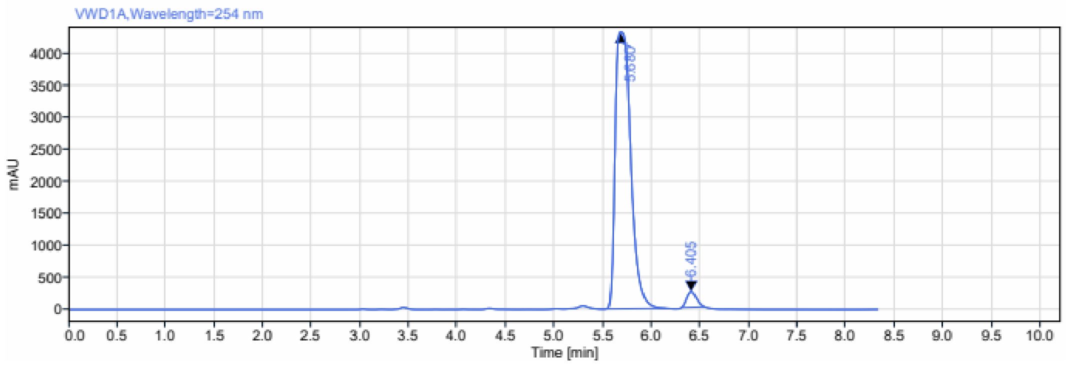

Signal: VWD1A,Wavelength=254 nm

RT [min]	Type	Width [min]	Area	Height	Area%	Name
5.680	MM m	0.70	48192.09	4321.17	96.60	
6.405	MM m	0.24	1697.57	241.14	3.40	
		**Sum**	**49889.66**			

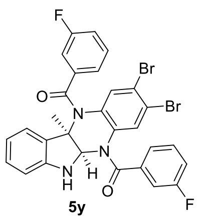

5y

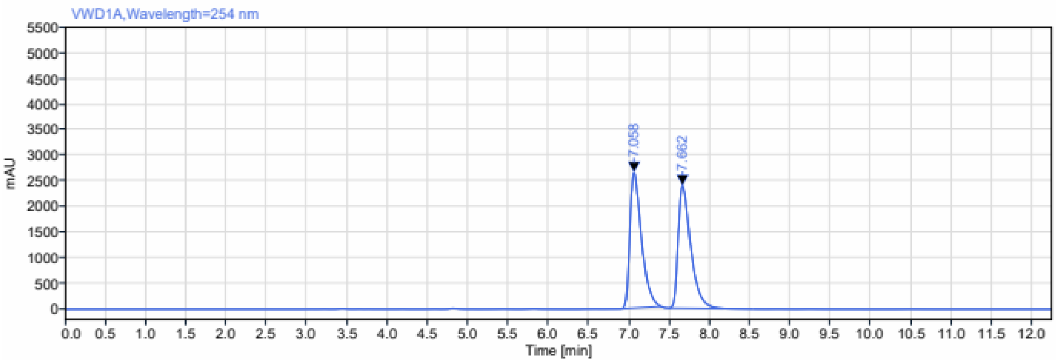

Signal: VWD1A,Wavelength=254 nm

RT [min]	Type	Width [min]	Area	Height	Area%	Name
7.058	MM m	0.50	26688.63	2650.31	50.63	
7.662	MM m	0.68	26022.57	2394.53	49.37	
		**Sum**	**52711.20**			

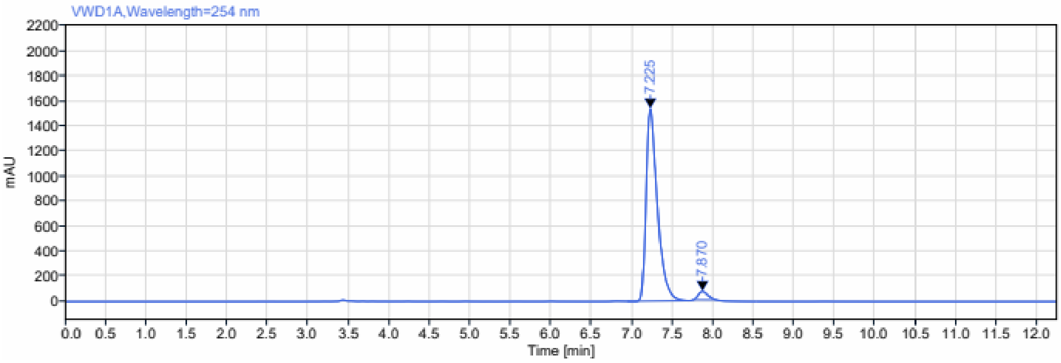

Signal: VWD1A,Wavelength=254 nm

RT [min]	Type	Width [min]	Area	Height	Area%	Name
7.225	MM m	0.74	14968.02	1532.75	96.55	
7.870	MM m	0.28	534.24	63.58	3.45	
		**Sum**	**15502.26**			

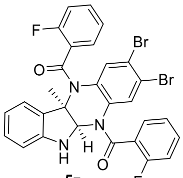

5z

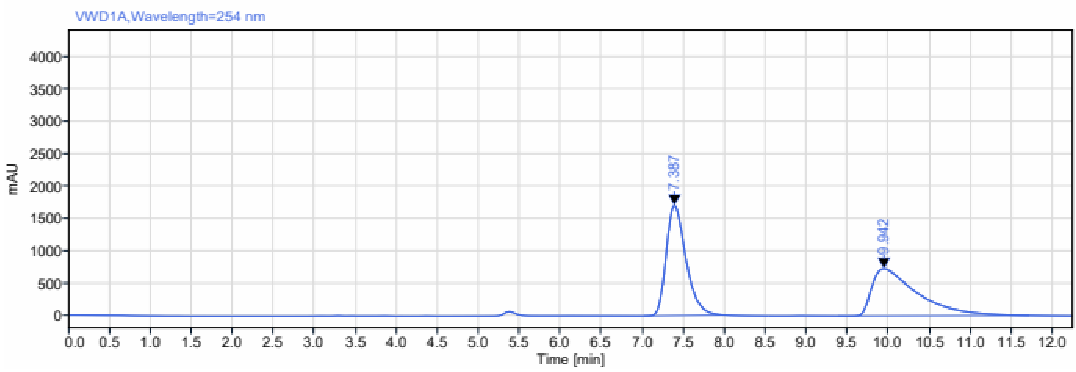

Signal: VWD1A,Wavelength=254 nm

RT [min]	Type	Width [min]	Area	Height	Area%	Name
7.387	MM m	0.99	28649.65	1699.17	50.28	
9.942	MM m	2.33	28325.17	728.34	49.72	
		**Sum**	**56974.82**			

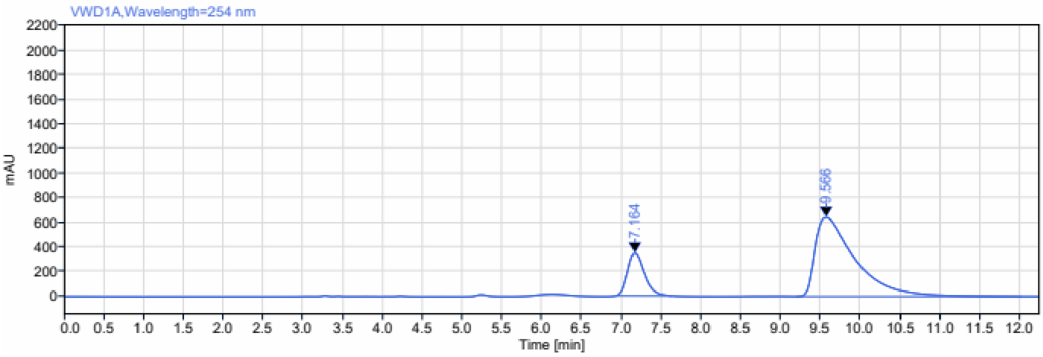

Signal: VWD1A,Wavelength=254 nm

RT [min]	Type	Width [min]	Area	Height	Area%	Name
7.164	MM m	0.70	5096.45	347.59	18.49	
9.566	MM m	2.83	22471.30	644.80	81.51	
		**Sum**	**27567.75**			

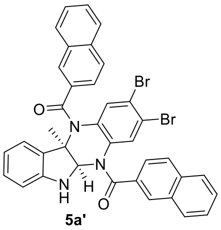

5a'

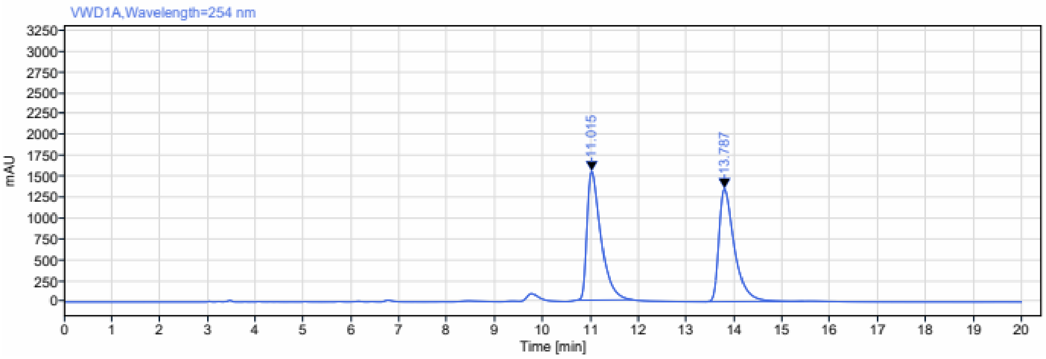

Signal: VWD1A,Wavelength=254 nm

RT [min]	Type	Width [min]	Area	Height	Area%	Name
11.015	MM m	1.19	31363.44	1537.23	50.51	
13.787	MM m	2.06	30729.31	1350.32	49.49	
		**Sum**	**62092.74**			

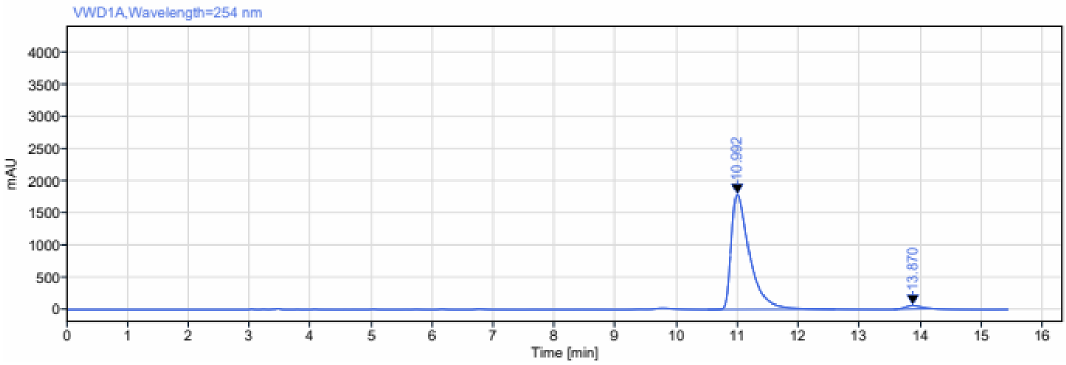

Signal: VWD1A,Wavelength=254 nm

RT [min]	Type	Width [min]	Area	Height	Area%	Name
10.992	MM m	2.10	37464.34	1784.75	97.48	
13.870	MM m	0.62	966.81	52.25	2.52	
		**Sum**	**38431.14**			

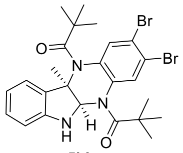

5b'

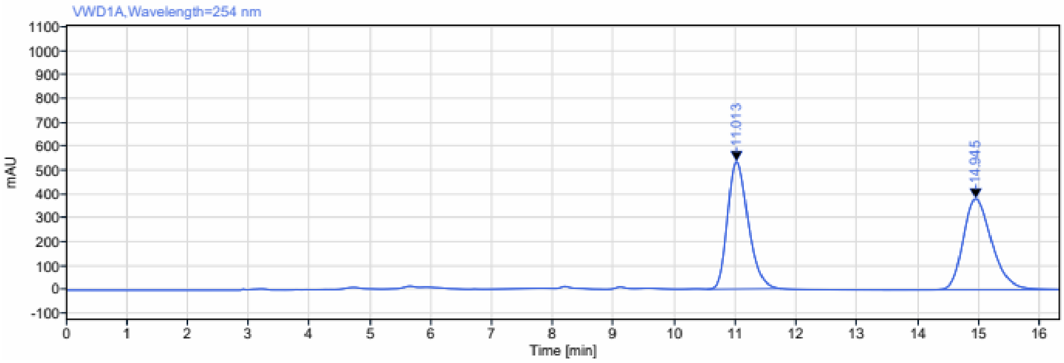

Signal: VWD1A,Wavelength=254 nm

RT [min]	Type	Width [min]	Area	Height	Area%	Name
11.013	VM m	1.22	12584.77	531.99	50.11	
14.945	MB m	3.69	12528.01	380.12	49.89	
		**Sum**	**25112.78**			

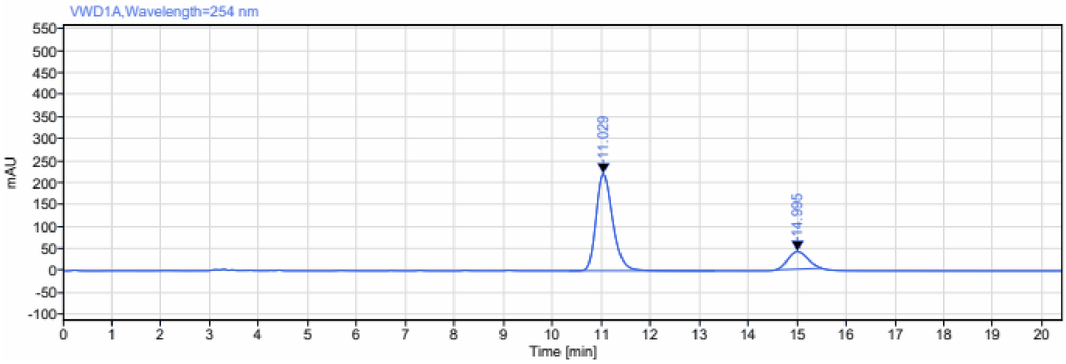

Signal: VWD1A,Wavelength=254 nm

RT [min]	Type	Width [min]	Area	Height	Area%	Name
11.029	BB	2.98	5251.10	220.41	82.56	
14.995	MM m	0.94	1109.49	39.60	17.44	
		**Sum**	**6360.59**			

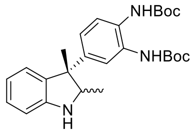

7

Sample Info : IE 8/2 1mL/min

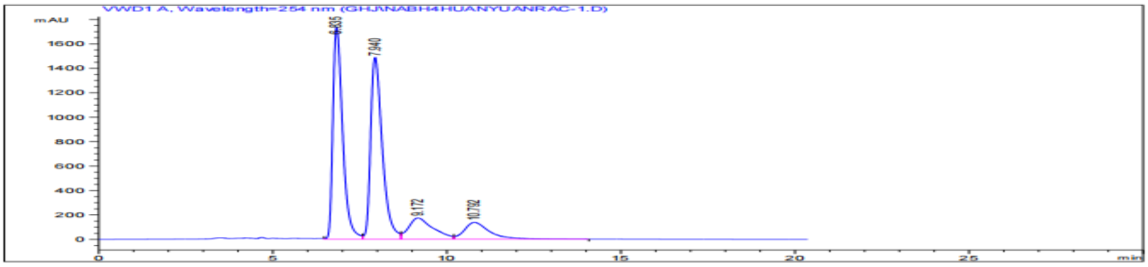

Area Percent Report

Sorted By : Signal
Multiplier : 1.0000
Dilution : 1.0000
Use Multiplier & Dilution Factor with ISTDs

Signal 1: VWD1 A, Wavelength=254 nm

Peak #	RetTime [min]	Type	Width [min]	Area mAU *s	Height [mAU]	Area %
1	6.835	VV	0.3004	3.38257e4	1733.52271	40.2868
2	7.940	VV	0.3571	3.47086e4	1485.09546	41.3383
3	9.172	VV	0.7013	8474.04883	173.36226	10.0927
4	10.792	VB	0.7510	6953.93506	136.64418	8.2822

Totals : 8.39623e4 3528.62460

Sample Info : IE 8/2 1mL/min

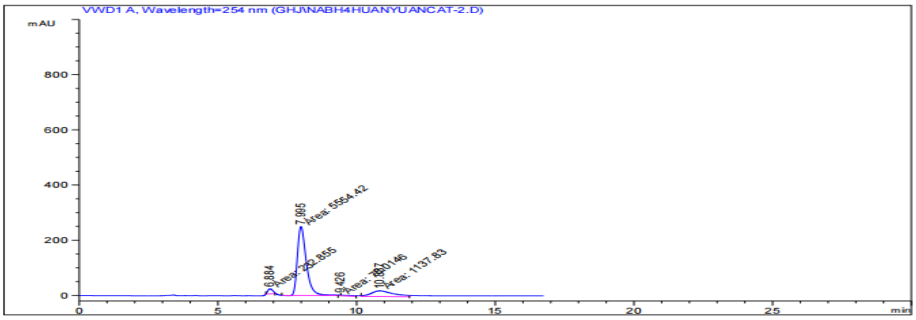

Area Percent Report

Sorted By : Signal
Multiplier : 1.0000
Dilution : 1.0000
Use Multiplier & Dilution Factor with ISTDs

Signal 1: VWD1 A, Wavelength=254 nm

Peak #	RetTime [min]	Type	Width [min]	Area mAU *s	Height [mAU]	Area %
1	6.884	MM	0.2106	232.85539	18.42425	3.3250
2	7.995	MM	0.3715	5554.41553	249.15637	79.3135
3	9.426	MM	0.5160	78.01457	2.51972	1.1140
4	10.837	MM	0.9292	1137.83276	20.40848	16.2475

Totals : 7003.11826 290.50882

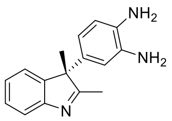

8

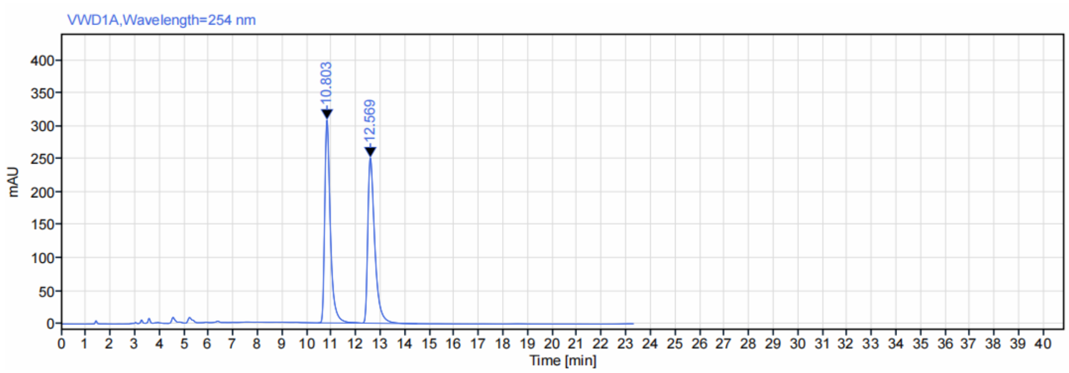

Signal: VWD1A,Wavelength=254 nm

RT [min]	Type	Width [min]	Area	Height	Area%	Name
10.803	BB	1.79	4922.92	307.93	49.88	
12.569	BB	2.25	4947.04	250.88	50.12	
		**Sum**	**9869.96**			

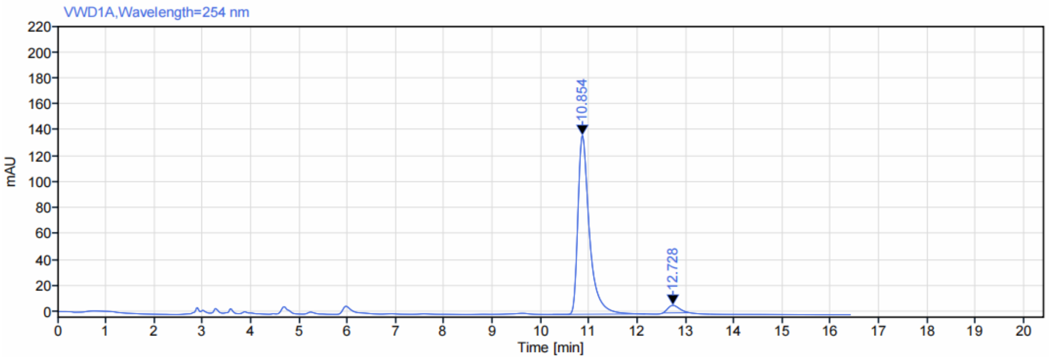

Signal: VWD1A,Wavelength=254 nm

RT [min]	Type	Width [min]	Area	Height	Area%	Name
10.854	MM m	1.92	2277.30	137.68	95.98	
12.728	MM m	0.56	95.40	5.77	4.02	
		**Sum**	**2372.70**			

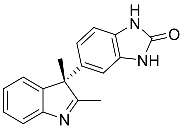

9

Sample Info : IG 7/3 1mL/min

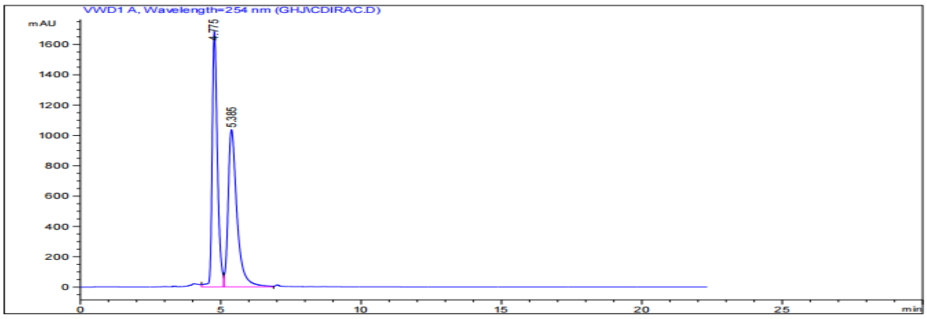

Area Percent Report

Sorted By : Signal
Multiplier : 1.0000
Dilution : 1.0000
Use Multiplier & Dilution Factor with ISTDs

Signal 1: VWD1 A, Wavelength=254 nm

Peak #	RetTime [min]	Type	Width [min]	Area mAU *s	Height [mAU]	Area %
1	4.775	VV	0.1973	2.19191e4	1680.32861	49.0136
2	5.385	VV	0.3279	2.28014e4	1036.98328	50.9864

Totals : 4.47205e4 2717.31189

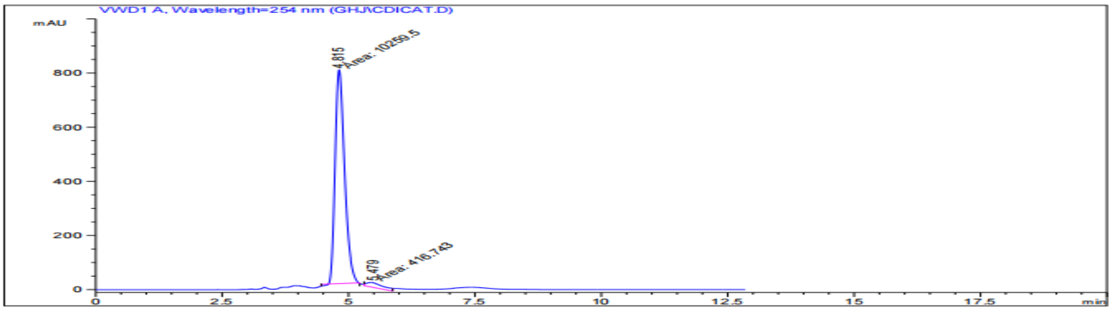

Area Percent Report

Sorted By : Signal
Multiplier : 1.0000
Dilution : 1.0000
Use Multiplier & Dilution Factor with ISTDs

Signal 1: VWD1 A, Wavelength=254 nm

Peak #	RetTime [min]	Type	Width [min]	Area mAU *s	Height [mAU]	Area %
1	4.815	MM	0.2168	1.02595e4	788.66791	96.0965
2	5.479	MM	0.4052	416.74323	17.14070	3.9035

Totals : 1.06763e4 805.80860

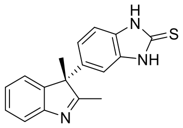

10

Sample Info : IG 7/3 1mL/min

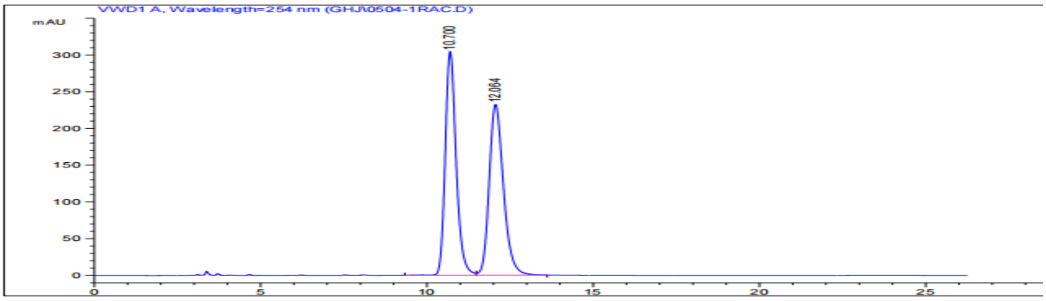

Area Percent Report

Sorted By : Signal
Multiplier : 1.0000
Dilution : 1.0000
Use Multiplier & Dilution Factor with ISTDs

Signal 1: VWD1 A, Wavelength=254 nm

Peak #	RetTime [min]	Type	Width [min]	Area mAU *s	Height [mAU]	Area %
1	10.700	BV	0.3492	6936.68701	304.01538	50.0429
2	12.064	VB	0.4570	6924.78662	232.27615	49.9571

Totals : 1.38615e4 536.29153

Sample Info : IG 7/3 1mL/min

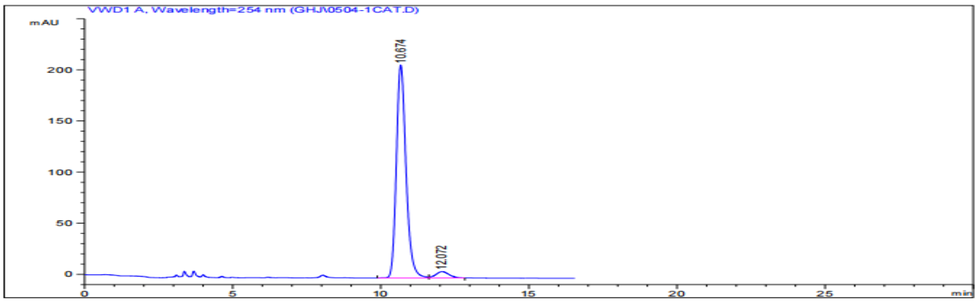

Area Percent Report

Sorted By : Signal
Multiplier : 1.0000
Dilution : 1.0000
Use Multiplier & Dilution Factor with ISTDs

Signal 1: VWD1 A, Wavelength=254 nm

Peak #	RetTime [min]	Type	Width [min]	Area mAU *s	Height [mAU]	Area %
1	10.674	BB	0.3479	4726.90186	208.16728	96.0380
2	12.072	BB	0.4692	195.00819	6.29483	3.9620

Totals : 4921.91005 214.46211

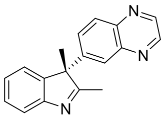

11

Sample Info : IC 7/3

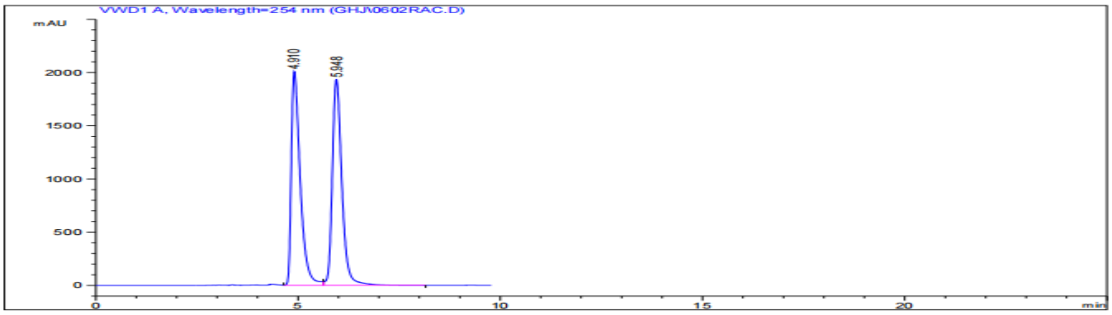

===
Area Percent Report
===

Sorted By : Signal
Multiplier : 1.0000
Dilution : 1.0000
Use Multiplier & Dilution Factor with ISTDs

Signal 1: VWD1 A, Wavelength=254 nm

Peak #	RetTime [min]	Type	Width [min]	Area mAU *s	Height [mAU]	Area %
1	4.910	VV	0.2318	3.08596e4	2007.02527	49.2306
2	5.948	VB	0.2516	3.18241e4	1934.53296	50.7694
Totals :				6.26837e4	3941.55823	

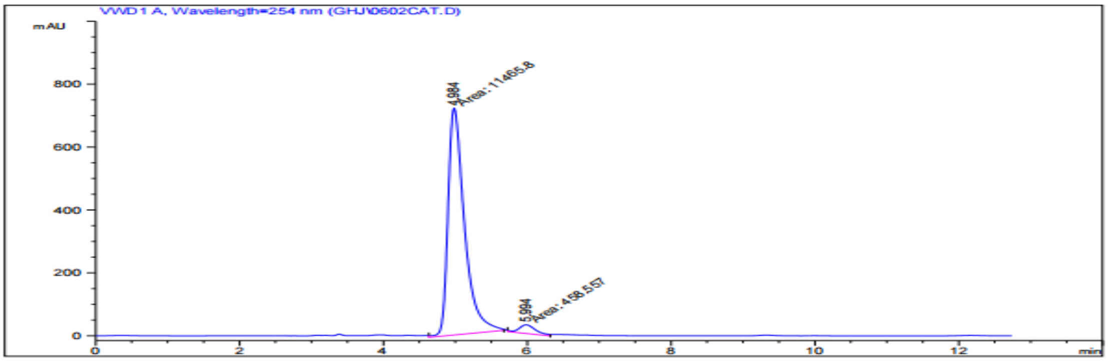

===
Area Percent Report
===

Sorted By : Signal
Multiplier : 1.0000
Dilution : 1.0000
Use Multiplier & Dilution Factor with ISTDs

Signal 1: VWD1 A, Wavelength=254 nm

Peak #	RetTime [min]	Type	Width [min]	Area mAU *s	Height [mAU]	Area %
1	4.984	MM	0.2652	1.14658e4	720.55005	96.1544
2	5.994	MM	0.2744	458.55710	27.85127	3.8456
Totals :				1.19243e4	748.40132	

7. References

[1] (a) Cheng, H.-G.; Lu, L.-Q.; Wang, T.; Yang, Q.-Q.; Liu, X.-P.; Li, Y.; Deng, Q.-H.; Chen, J.-R.; Xiao, W.-J. *Angew. Chem. Int. Ed.* **2013**, *52*, 3250-3254. (b) Han, L.; Liu, C.; Zhang, W.; Shi, X.-X.; You, S.-L. *Chem. Commun.*, **2014**, *50*, 1231-1233. (c) Kieffer, M.; Chuang, K. V.; Reisman, S. E. *J. Am. Chem. Soc.* **2013**, *135*, 5557 - 5560.
[2] (a) Adams, R.; Way, J. W. *J. Am. Chem. Soc.* **1954**, *76*, 2763-2769. (b) Abraham, C. J.; Paull, D. H.; Scerba, M. T.; Grebinski, J. W.; Lectka, T. *J. Am. Chem. Soc.* **2006**, *128*, 13370-13371. (c). Huang, R.-Y.; Luo, G.-Y.; Li, Y.-J.; Li, X.-Y.; Xue, W.; Jin, Z.-C.; Chi, Y.-G. *Org. Lett.* **2019**, *21*, 4340-4344.
